# LncSNHG1 Promoted CRC Proliferation through the miR-181b-5p/SMAD2 Axis

**DOI:** 10.1155/2022/4181730

**Published:** 2022-03-11

**Authors:** Qi Huang, Zhi Yang, Jin-hai Tian, Pei-dong You, Jia Wang, Rong Ma, Jingjing Yu, Xu Zhang, Jia Cao, Jie Cao, Li-bin Wang

**Affiliations:** ^1^The Biochip Research Center of General Hospital of Ningxia Medical University, Yinchuan 750004, China; ^2^Guangzhou First People's Hospital, Guangzhou 510180, China

## Abstract

**Objective:**

To investigate the effects of LncRNA SNHG1 on the proliferation, migration, and epithelial-mesenchymal transition (EMT) of colorectal cancer cells (CRCs).

**Methods:**

4 pairs of CRC tissue samples and their corresponding adjacent samples were analyzed by the human LncRNA microarray chip. The expression of LncSNHG1 in CRC cell lines was verified by qRT-PCR. Colony formation assays and CCK8 assays were applied to study the changes in cell proliferation. The transwell assay and wound healing experiments were used to verify the cell invasion and migration. EMT progression was confirmed finally.

**Results:**

LncSNHG1 was overexpressed both in CRC tissues and cell lines, while the miR-181b-5p expression was decreased in CRC cell lines. After knock-down of LncSNHG1, the proliferation, invasion, and migration of HT29 and SW620 cells were all decreased. Meanwhile, LncSNHG1 enhanced EMT progress through regulation of the miR-181b-5p/SMAD2 axis.

**Conclusion:**

LncSNHG1 promotes colorectal cancer cell proliferation and invasion through the miR-181b-5p/SMAD2 axis.

## 1. Introduction

Colorectal cancer (CRC) is one of the most common gastrointestinal malignant tumors and the second leading cause of cancer mortality worldwide [1]. In spite of multiple treatment such as surgery, radiotherapy, and chemotherapy, the 5-year subsistence rate of CRC remained around 55% while metastasis and recurrence are the leading causes of death [2, 3]. Accordingly, exploring the molecular mechanisms associated with the incidence of CRC may be of great help to seek effective treatment strategies and improve the prognosis. Although 80% of human genes may be transcribed into RNA, more than 98% are non-protein-coding RNAs (ncRNAs). Long noncoding RNAs (LncRNAs) are a kind of noncoding RNA which has about 200 nucleotides. LncRNAs have been improved to associated with diverse diseases such as Parkinson's disease and various cancers [4, 5]. Long noncoding RNA small nucleolar RNA host gene 1 (LncRNA SNHG1 and LncSNHG1), which localized at 11q12.3 region and has 11 exons, was reported to play an important role in enhancing cell proliferation, invasion, apoptosis, and epithelial-mesenchymal transition (EMT) in several cancers, including colorectal cancer, non-small-cell lung cancer, and gastric cancer [6–9]. The increased expression of LncSNHG1 is an effective marker in predicting a poor outcome in CRC patients [10]. LncRNAs have been reported to function through competition for microRNA (miRNA) binding, resulting in imposing posttranscriptional regulation in CRC cells [11].

MicroRNAs (miRNAs) are a group of noncoding RNAs which have 21–24 nucleotides in length. miRNAs often play their function in the posttranscriptional regulation through ceRNA with LncRNA [12]. The expression change of miRNA has been related with different cancers. Fridrichova et al. have reported that miRNAs are involved in regulating invasive processes, cell-cell adhesion junctions, cancer cell-extracellular matrix interactions, and cancer cell stem abilities in breast cancer [13]. Cao et al. proved that miRNA-124 and miR-552 regulate tumor cell proliferation and migration in hepatocellular carcinoma and CRC [14, 15]. miR-181b is a member of miR-181 family and positioned at chr9q3.33 [16]. It has been reported that miR-181b could inhibit the progression of human colon cancer cell proliferation [17]. However, the molecular mechanism by which miR-181b mediates CRC progression still needs further study.

In this study, we used TargetScan software to search the potential miRNA sites that complement the untranslated area (UTR) of the SNHG1 3 'end. miR-181 b-5p was upregulated more than 2 times with LncSNHG1 silencing. miR-181 b directly binds to the 3′ untranslated regions (UTRs) of both LncSNHG1 and SMAD2 in CRC cells. Furthermore, the TGF-*β*/SMAD2 pathway was activated abnormally and closely related to cell proliferation, EMT progress, chemoresistance, and poor prognosis in CRC [18].

Epithelial-mesenchymal transition (EMT) is defined as the transformation of an epithelial cell into mesenchymal cells. Accompanying the process was the loss of membrane E-cadherin expression and the gain of mesenchymal marker positivity [19]. EMT has been shown to be an essential process during CRC invasion and metastasis since 1995 [20]. Previous studies have shown the association between LncSNHG1 and EMT in different cancers. Liu et al. have shown that the overexpression of LncSNHG1 enhances the EMT process via the miR-15b/DCLK1/Notch1 axis and promotes the metastasis in GC cells [9]. LncSNHG1 also plays a vital role in the promotion of the cell cycle, migration, and EMT progression of hepatocellular carcinoma [21]. Although LncSNHG1 can indicate CRC deterioration by cooperating with miR-497/miR-195-5p to modify the EMT process, the molecular mechanism mediated by LncSNHG1 in CRC progression remains unknown [22].

Based on the abovementioned background, we assumed the LncSNHG1 promoted the CRC progress via miR-181b-5p binding. In this study, we aim to present in vitro results to certificate the change of LncSNHG1, miR-181b-5p, and SMAD2 in CRC to provide a potential application for the treatment of CRC.

## 2. Materials and Methods

### 2.1. Clinical Samples Collection

Approval for this research was given by the General Hospital of Ningxia Medical University Ethics Committee. All the patients involved have signed informed consent forms. We recollected 24 paired colorectal tissues and adjacent normal samples from the oncology surgery department of the General Hospital of Ningxia Medical University. All of the tissue specimens were confirmed for diagnosis based on hematoxylin-eosin and pathological examination. 4 pairs of cancer tissues and adjacent tissues were randomly selected for differentially expressed LncRNA screening (LncRNA chip hybridization, Biochip, China).

### 2.2. Microarray Analysis

4 pairs of cancer tissues and adjacent normal tissues were used for the microarray analysis. Human LncRNA Array v2 microarray (Beijing Capital Bio Biotechnology Corporation, China) has been used for LncRNA microarray profiling analysis. The LncRNA array data were analyzed by GeneSpring 13.0 (Agilent) software. Bioanalyzer 2100 (Agilent Technologies, USA) was used for the RNA integrity analysis. RNA digestion, amplification, and labelling were performed according to protocol. The microarray contained 162351 human LncRNA probes. With the filter criteria fold-change ≥2, *P*value < 0.05, fluorescence value ≥ 100, differentially expressed LncRNAs were detected and separated.

### 2.3. Cell Line and Cell Culture

The human colorectal cancer cell lines HCT116, HT29, LOVO, and SW620 and human colon epithelial cells NCM460 were purchased from the cell bank of the Chinese Academy of Sciences. The cells were cultured with high glucose DMEM medium (HyClone, Logan, USA) supplemented with 1% penicillin-streptomycin solution and 10% fetal bovine serum (Gibco, Australia). The cells were incubated at 37 °C with 5% CO_2_.

### 2.4. Plasmids and Cell Transfection

shRNA for LncSNHG1 and negative control shRNA-NC plasmid, miR-181b-5p inhibitor and negative control (NC) vector were all designed and synthesized from HanBio Company (China). HT29 and SW620 cells were cultured in 6-well plates. When the cell density reaches 70%–80% confluence, a 2.5 µg plasmid vector was transfected to the cells using Lipofectamine 3000 (Invitrogen, CA, USA) according to the manufacturer's protocol. Fluorescence microscope, qRT-PCR, and western blot assays were used for observing the cell transfection efficiency.

### 2.5. Real-Time Quantitative Reverse Transcription-PCR (qRT-PCR)

Following the manufacturer's protocol, the total RNA was extracted from tissues and cells by using TRIzol reagent (Invitrogen, CA, USA). cDNA was synthesized with the TaKaRa verse transcription kit (TaKaRa, Shanghai, China). The PCR amplification was performed with specific primer sat Prism 7900 system (ABI, USA). GAPDH served as internal control. Using 2^–ΔΔCt^ methods, we calculated the relative expression of each gene.

### 2.6. Dual-Luciferase Reporter Assay

Based on the TargetScan database, LncSNHG1 has binding sites with miR-181b-5p at the 3'-UTR. To identify it, the wt-Lnc SNHG1 and mut-LncSNHG1 luciferase expression vectors were constructed from GeneChem Co. and incubated into the vector and cells in a 24-well plate (5 × 10^3^ cells per well). When the cells fusioned to 80%, the wt-LncSNHG1 and mut-LncSNHG1 groups were transfected into miR-NC and miR-181b-5p, respectively. The double luciferase reporting experiment was carried out using the luciferase reporter kit. The ratio of luciferase activity was statistically analyzed. All the experiments was repeated three times.

### 2.7. Cell Migration and Invasion Assay

To determine the cell migration and invasion, wound healing assay was used. 1Х10^5^ HT29 and SW620 cells were seeded in a 6-well plate and transfected with an interfering vector for 48 h. The cell was wounded with a 200 µl pipet tip scraping across the monolayer. After that, the speed of the wound's recovery was photographed. The cell mobility was assessed by calculating the wound width.

### 2.8. Colony Formation Assay

After being transfected with an interfering or overexpressing vector, the HT29 and SW620 cells were seeded in a 6-well plate. The medium was exchanged every three days. Two weeks later, the cells were fixed with 100% methanol for 15 min. Then, it was stained with crystal violet for 10 min. The colony numbers of representative areas were observed and calculated. All experiments were performed three times.

### 2.9. Flow Cytometry

The HT29 and SW620 cells were seeded in a 6-well plate after being transfected with an interfering or overexpressing vector for 48 h. Then, the cells were digested with trypsin and 1 × 10^6^ cells were counted for analysis. The cells were washed with cold PBS three times. They were then collected and suspended into a single cell in a binding buffer. According to the instructions of the apoptosis detection kit (Best Bio, Shanghai, China), the cells were stained with Annexin V-APC for 10 min and propidium iodide (PI) for 10 min. A BD flow cytometer examined the samples, and FlowJo software (Tree Star Corporation, Ashland, OR, USA) analyzed the data.

### 2.10. Western Blot

The total protein of each group was extracted and the protein concentration was detected by the BCA protein reagent kit (Thermo Fisher Scientific, Inc.). The protein samples were separated with 10% SDS-PAGE and transferred to a PVDF membrane. The membranes were blocked with 5% defatted milk in TBST at 25°C for 1 h. The membrane was incubated with a specific primary antibody (1 : 1 000) at 4°C overnight. Then, the HRP-conjugated secondary antibodies were incubated at 25 °C for 2 h. After washing the membrane with TBST 3 times, the protein bands were detected and scanned by Bio Imaging Systems (BIO-RAD, CA, USA). The absorbance of the protein bands in each group was measured by Quantity One gel analysis software. The protein expression level is assessed by the ratio of the target band to the GAPDH band. Each protein sample was applied with 3 repeats.

### 2.11. Statistical Analysis

Statistical analysis was carried out using SPSS 20.0 software. The mean ± standard deviation (*x* ± *s*) was used to measure the data expression. The *T* test was performed between the two groups. A single factor variance analysis was used to analyze the variance between the two groups. *P*value < 0.05 indicated that the difference has the significance of statist.

## 3. Result

### 3.1. Expression Analysis of LncRNAs and Its Verification in CRC Tissues and Cells

**Figure 1:**
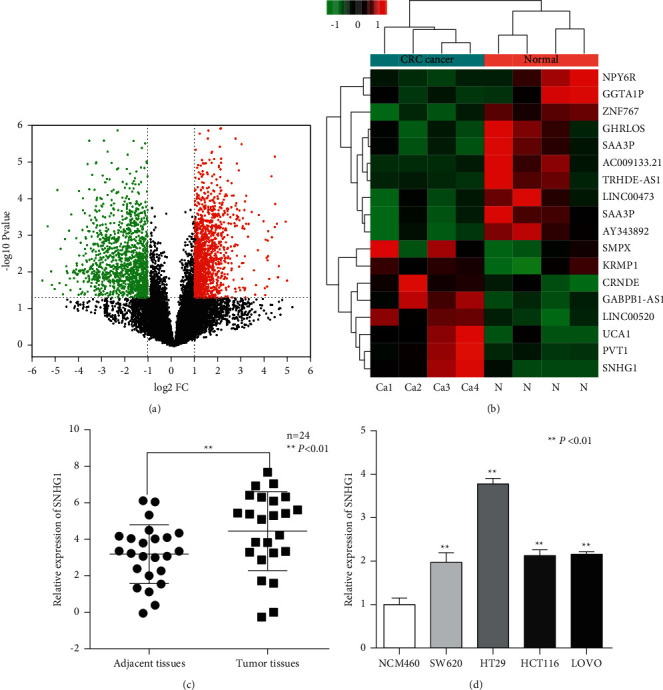
LncRNAs expression analysis in CRC tissues and cells. (a, b) Cluster and volcano plot analysis of different expression of LncRNAs between CRC tissues samples (CA1–CA4) and adjacent normal samples (N) based on the microarray profiles. The expression levels are presented in red and green, which indicated upregulated and downregulated LncRNAs. (C, D)   The expression of LncSNHG1 in CRC tissues (c) and cell lines (d) (^*∗*^*P* < 0.05 and ^*∗∗*^*P* < 0.01).

To screen for specific LncRNA in colorectal cancer, we selected 4 CRC tissue samples (CA1-CA4) and adjacent normal samples (AP1-AP4) for investigating the expression of LncRNAs by microarray profiles. The cluster analysis displayed the different expression of LncRNAs between the two groups (Figure 1(a)). Based on the different fluorescence signal values, the variation in LncRNA expression between the CRC and adjacent normal tissues is described in the volcano plot (Figure 1(b)). As illustrated in Figure 1, 13198 differentially expressed LncRNAs were separated. Taking the fold-change ≥5 or ≤ -5, the *P* value <0.01 and the processed signal ≥100 as screening criteria, 18 candidate LncRNAs were selected including 8 upregulation and 10 downregulation. Among them, LncSNHG1 was significantly highly expressed in CRC tissues.

To further verify the selected LncSNHG1 with CRC, qRT-PCR explored the LncSNHG1 expression in CRC tissues and cell lines. The results showed the expression of LncSNHG1 in 24CRC was 4.45 ± 2.11, which was higher than that in para cancerous tissues (Figure 1(c); (p) < 0.05). Compared with human normal colorectal cancer NCM460 cells, LncSNHG1 was highly expressed in SW620, HT29, HCT116, and LOVO cell lines (Figure 1(d); *P* < 0.05). Based on the results, we selected HT29 and SW620 cell lines for the following experiments.

### 3.2. LncSNHG1 Promoted CRC Cells Proliferation and Migration

**Figure 2:**
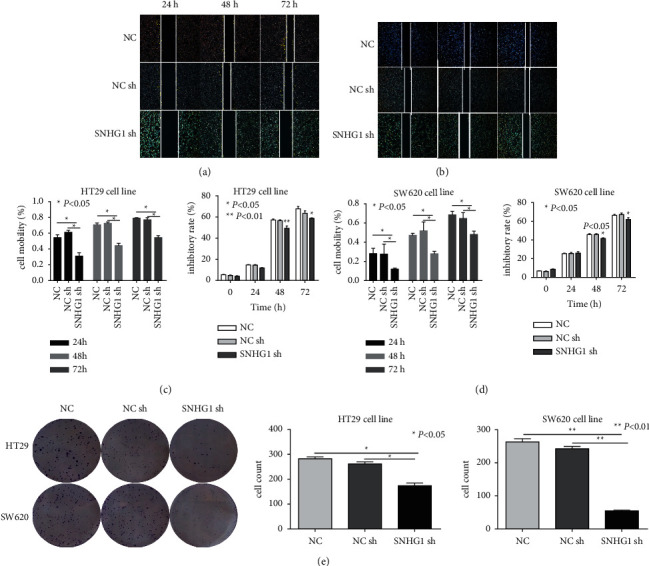
LncSNHG1 promoted CRC cells proliferation and migration. (A–D) The migration assays showed the cell mobility and inhibition rate of HT29 (A, C) and SW620 cells (B, D) after downregulation of lncSNHG1. (E) Colony formation assay showed the cell self-renewal in HT29 and SW620 cells after downregulation of SNHG1 (^∗^*P* < 0.05 and ^∗∗^*P* < 0.01).

To investigate the effect of LncSNHG1 on CRC cell growth, we constructed siRNA for LncSNHG1 and transfected it into HT29 and SW620 cells. After 48 h, all cell proliferation was significantly inhibited, while cell mobility was decreased (Figure 2(a)–2(d)). The results demonstrated that sh-SNHG1 had anticancer effects on CRC cells. The results of colony formation assays showed that the colony numbers were smaller and fewer when treated with sh-SNHG1 (Figure 2(e)). These results illustrated that LncSNHG1has the function of promoting CRC cell proliferation.

### 3.3. Downregulation of LncSNHG1 Influence the Cell Cycle and Promoted Apoptosis of HT29 and SW620 Cells

**Figure 3:**
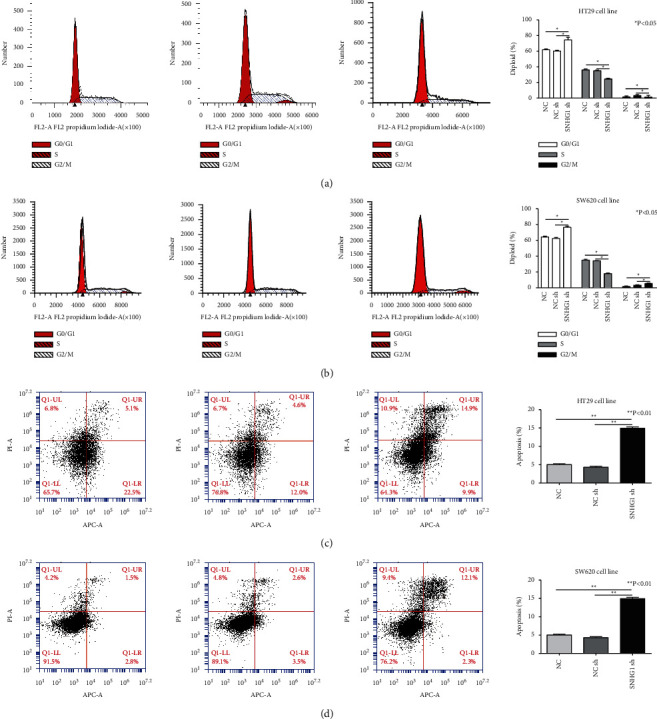
The effects of LncSNHG1 on HT29 and SW620 cell cycle and inhibited apoptosis. (A, B) The cell cycle changed after downregulation of LncSNHG1 in HT29 and SW620 cells. (C, D)  The cells apoptosis being promoted by downregulation of LncSNHG1 (^∗^*P* < 0.05 and ^∗∗^*P* < 0.01).

With the flow cytometry analysis, the effects of LncSNHG1 on the cell cycle and apoptosis were evaluated. The results showed that the G0/*G*1 phrases were all increased after being treated with sh-SNHG1 in HT29 and SW620 cells. Meanwhile, the cells' S phrases were all decreased. The results proved that inhibition of LncSNHG1 enhanced the cells G0/*G*1 accumulation (Figures 3(a) and 3(b)).

Compared with the control and NC groups, the apoptosis rate in the sh-SNHG1 group was significantly increased both in HT29 and SW620 cells. The results indicated that inhibition of LncSNHG1 promoted apoptosis of CRC cells (Figure 3(c) and 3(d)).

### 3.4. miR-181b-5pas ceRNA to LncSNHG1 in CRC Cells

**Figure 4:**
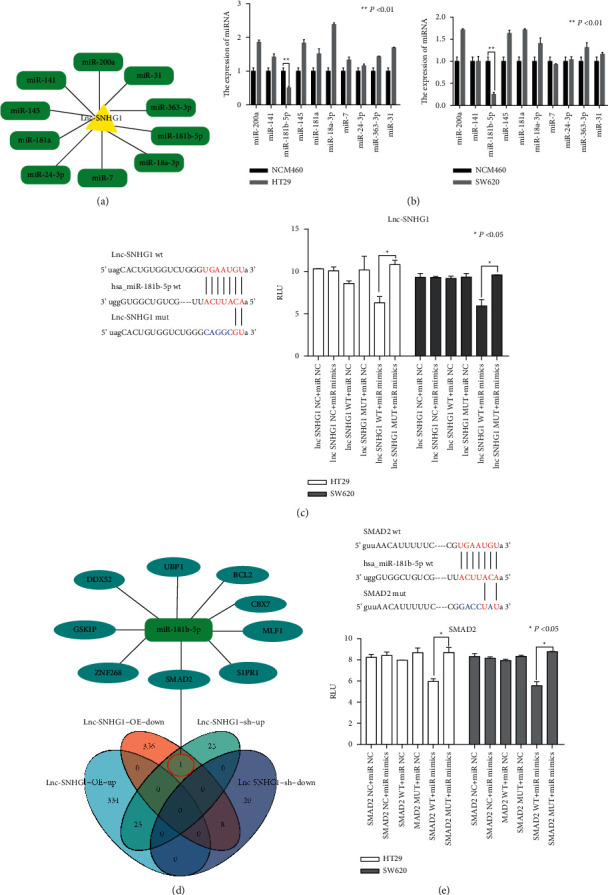
miR-181b-5pas ceRNA to LncSNHG1 in CRC cells. (A, B)  Target miRNA prediction and identify in HT29 and SW620 cells. (C) Luciferase reporter assays tested the direct combination between LncSNHG1 3'UTR and miR-181b-5p. (D, E) Prediction and identification of SMAD2 as miR-181b-5p target gene through RNA transcriptome sequence and luciferase reporter assay (^*∗∗*^*P* < 0.05).

A large body of research literature has proved that LncRNA is an important factor to regulate the expression of miRNA. In this study, we identified the target miRNA of LncSNHG1 through starBase database (http://starbase.sysu.edu.cn/) and searched for potential sites that complement the untranslated area (UTR) of theSNHG1 3 ′end with TargetScan software. Based on the bioinformatic analyses, we selected 10 miRNAs which matched to LncSNHG1 (Figure 4(a)). The expression of 10 miRNAs was measured in HT29 and SW620 cells after LncSNHG1 silencing. The results proved that the expression of miR-181b-5p was significantly decreased in the cell lines (Figure 4(b)). In order to confirm the binding site of LncSNHG1 to miR-181b-5p, luciferase reporter assays were used. We found that in the LncSNHG1-wt report vector transfer group, miR-181b-5p mimic could reduce the activity of luciferase. But there is no difference between LncSNHG1 mutation and the miR-181b-5p mimic group. The results proved that there was a direct combination between LncSNHG1 3'UTR and miR-181b-5p (Figure 4(c)).

To further identify the specific target genes that are regulated by LncSNHG1 in CRC, RNA transcriptome sequencing was carried out. Based on the results, the most highly expressed genes such as ATP6V1C2, CLDN2, SMAD2, ITGA2, FOSL1, FBXO34, and others are selected. qRT-PCR were used to verify these genes, the results showed that SMAD2 was high expressed in HT29 and SW620 cells (Figure 4(d)). Bioinformatics analysis showed that there were seven complementary bases in the miR-181b-5p and SMAD2 3'UTR regions. Luciferase reporter assays proved that of the wild type SMAD2 and the miR-181b-5p mimic group was significantly lower than that of the miR-NC group. But the luciferase activity between the miR-NC and miR-181b-5p mimic groups was no significant difference (Figure 4(e)).

### 3.5. LncSNHG1 Induced CRC EMT through miR-181b-5p/SMAD2

**Figure 5:**
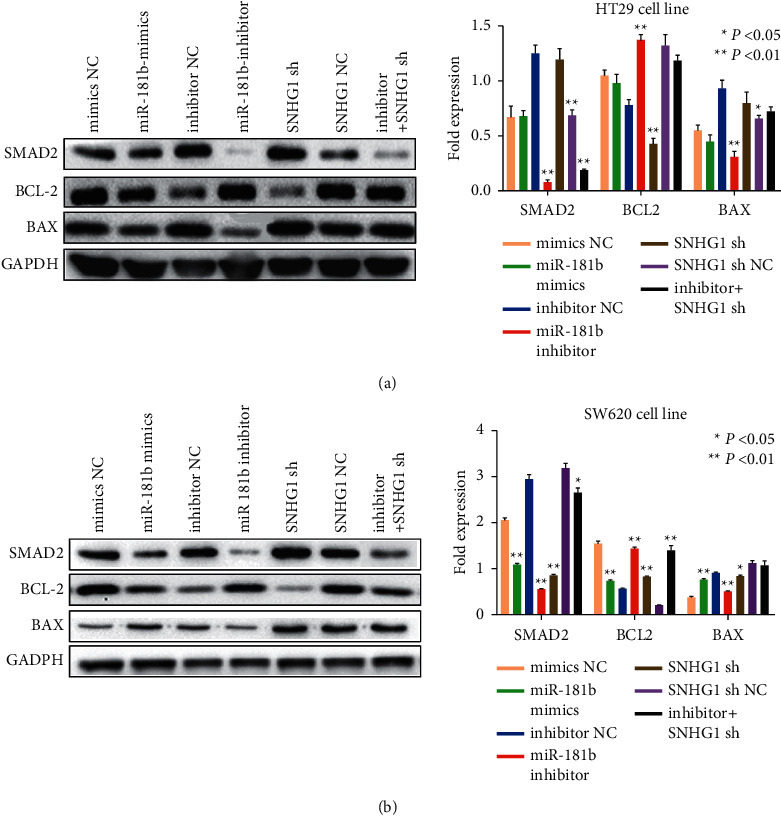
The relationship between LncSNHG1, miR-181b-5p, SMAD2, and apoptosis. (A, B) The expression of SMAD2 and apoptosis-related proteins Bcl-2 and Bax after treated with sh-SNHG1 and 181b-5p inhibitor in HT29 and SW620 cells.

To identify the relationship between LncSNHG1 and miR-181b-5p and the EMT regulated by the ceRNA in CRC, western blot was used. After transfecting miR-181b-5p mimics and inhibitor into the cells, SMAD2, BCL-2, and BAX were tested. The results proved that SMAD2 and the apoptosis related protein Bax were remarkably decreased in the miR-181b-5p inhibitor group, while the apoptosis stimulating protein Bcl-2 showed a reduction. When the cells were treated with sh-SNHG1, SMAD2 and Bax expression were increased. Combined treated the cells with sh-SNHG1 and 181b-5p inhibitor could reverse the expression meanwhile (Figure 5(a) and 5(b)).

EMT is a process which often occurs in different cancer oncogenesis. Previous research has proved that SMAD2 could induce EMT and influence CRC progression. To verify the results, we tested the EMT-related protein after being transfected with 181b-5p and sh-SNHG1. Western blot results proved that N-cadherin, vimentin, and slug was increased while E-cadherin was decreased in the 181b-5p inhibitor group as well as in the sh-SNHG1 group.

**Figure 6:**
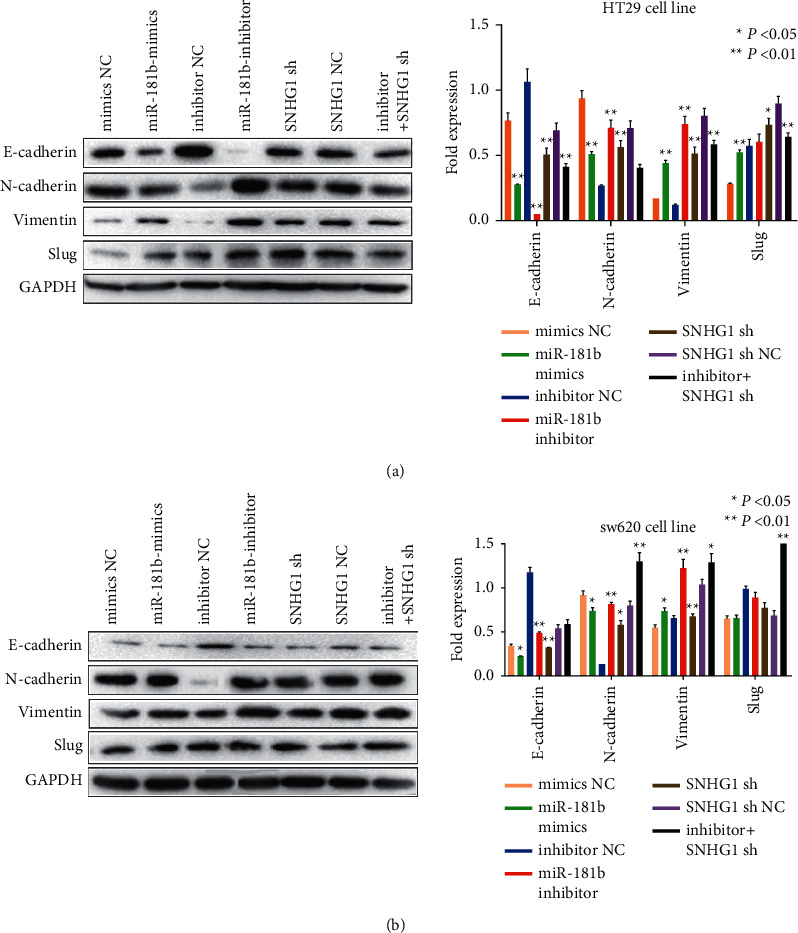
EMT induced by LncSNHG1 in CRC cells. (A, B) The different expression of EMT related protein after treated with 181b-5p and sh-SNHG1 in HT29 and SW620 cells.

When treated the cells with 181b-5p inhibitor combined with sh-SNHG1, all the protein expression would be reversed (Figure 6(a) and 6(b)). These results suggested that inhibiting miR-181b could reverse the anticancer effect of sh-SNHG1 on CRC cells through SMAD2.

## 4. Discussion

Due to the lack of effective methods for the early diagnosis of CRC and its unoptimistic survival rate, the study of the incidence and mechanism of CRC has been a hotspot these years. Plenty of LncRNAs and miRNAs have been indicated to be in association with CRC development, via kinds of pathways such as Wnt/*β*-catenin or TGF-*β*/Smad2/3 [11, 16, 23]. The essential finding of this research is that we identified LncSNHG1 plays an important role in proliferation, migration, invasion, and EMT progress by acting as a molecular sponge for miR-181b in CRC processing.

LncRNAs are transcribed by RNA polymerase II (PoII) but not translated into protein. Although they have been thought to be junk genes, LncRNAs have been identified as key regulatory elements in multiple physiological processes, such as cell cycle and proliferation, with the development of high-throughput technologies nowadays [24]. LncRNAs have been associated with a variety of malignant tumors progression [25]. Meanwhile, LncSNHG1 involvement in gene transcription, invasion, cell migration, metastasis, tumorigenesis, and chemoresistance of multiple cancers has been reported widely [26]. By analyzing the RNA-Seq and miR-Seq and corresponding clinical data of CRC from the TCGA database, A. Poursheikhani et al. reported that SNHG1 was one of the significant diagnostic LncRNAs in CRC development [23]. In our research, we utilized the Human LncRNA Microarray chip to determine that LncSNHG1 was upregulated in CRC tissue and was qualified by qRT-PCR. This finding was consistent with previous studies, while the relationship between poor prognosis of CRC patients and high level LncSNHG1 had been reported [10, 11, 27–29]. In our gain-of-function and loss-of-function experiments, LncSNHG1 was verified to enhance proliferation, migration, EMT progress, cell cycle progression, and inhibition of apoptosis in CRC cells. These results which were in accordance with forepassed literature identified that LncSNHG1 might serve as an oncogene in CRC development [7, 22, 29].

Most LncRNAs are involved in gene regulation mechanisms by acting as competing endogenous RNAs (ceRNAs) by miRNA recognition elements (MREs) [30, 31]. A couple of miRNAs (such as miR-154-5p and miR‐137) mediate the part of LncSNHG1 in modulating CRC development have been demonstrated [10, 29]. The miR-181b family had been implicated in the progression of kinds of malignant tumor such as glioblastoma, osteosarcoma, pancreatic cancer, breast cancer, and colorectal cancer [14, 15, 32–36]. Interestingly, the miR-181b family may act as oncogenes [32, 33] or tumor suppressors [36, 37] in different cancer types. Moreover, whether the miR-181b family in cancer tissue is upregulated or downregulated in prostate cancer [38–40] and gastric carcinoma [41, 42] remains a controversy. The function of miR-181b depend on not only the type of tumor but also the cellular circumstances may be the cause [43, 44]. A similar situation emerged in the research studies about the miR-181b family in CRC. miR-181b in CRC has been reported to be an oncomiRNA and the probable mechanism could be that association with the mutation status of the p53 gene [45], Warburg effect promotion [46], cylindromatosis (CYLD) expression suppression, PDCD4 suppression [35],and the NF-*κ*B signaling pathway [15] inhibition. But LUN-DE ZHAO and others and Si Chen, et al. showed that miR-181b could act as a tumor suppressor in CRC by targeting RASSF1A [34] and regulating TUSC3 [14], respectively. In our research, we verified that LncSNHG1 positively regulated proliferation, migration, invasion, and EMT by competing with miR-181b-5p in CRC cells. The result is similar with the previous one [14], and we are the only two reports in LncRNA-miR181b-CRC while the first study figured out the LncRNA-miR181b-EMT in CRC development until now. Every cloud has a silver lining; the different effects miR-181b would exert in CRC development might be decided by upstream factors such as LncRNAs. Further studies on the function of the miR-181b family in CRC processing are needed.

LncSNHG1 can act as an oncogene by regulating Wnt/*β*-catenin signaling, the PI3K/AKT signaling pathway, and the HIF-1*α*/VEGF signal pathway in different tumor progression [28, 47, 48]. In our research, the positive correlation between LncSNHG1 and SMAD2 was verified by the in vitro assays. The TGF‐*β* was demonstrated to act as both tumor suppressor (in the early stage) and progression promoter (in the advanced stage) by multifaceted impacts in cancer progression [49]. Smad2 is located at 18q21. It belongs to the Smad superfamily that transmit signals of TGF-*β* from receptors on cell membranes to the nucleus [50]. The role of TGF-*β*/Smad2 in colon cancer has been reported in various studies. Xinke Wang, et al. reported that LncRNA SNHG6 was a promoter in CRC progression by activating TGF-*β*/Smad signaling pathway [51]. While Xuning Shen, et al. proved the role of LncRNA TUG1 in CRC metastasis by TGF-*β* promotion [52]. In the current study, we report the function of LncRNA SNHG1 in TGF-*β*/SMAD2 signal way regulation in cancer development, which was similar with the previous study [51], indicating that the LncRNA SNHG family may regulate CRC progression in a similar way. Although EMT via the TGF-*β*/Smad pathway is a fundamental process for cancer metastasis and chemoresistance in CRC [17, 51], the literature about LncRNA SNHG1 in EMT is limited. Liu ZQ and others discovered the mechanism of SNHG1 in promoting EMT in gastric cancer cells [9]. Meng XF and others proved SNHG1 could mediate the proliferation, invasion, and EMT of prostate cancer by regulating miR-195-5p expression [53]. In our research, compared with the cells transfected with sh-SNHG1, the E-cadherin was reduced and the N-cadherin and vimentin were increased. These results suggested that inhibiting miR-181b-5p could reverse the anticancer effect of silent SNHG1 on CRC cells.

In brief, this study first proved that LncSNHG1 regulated the biological behavior of miR-181b-5p-miediated cells in colorectal cancer. Targeting the LncSNHG1 may present a promising therapeutic target for CRC, while more detailed research is still needed in near future [54, 55].

## Data Availability

The colorectal cancer LncRNA expression array data used to support the findings of this study are included within the supplementary information file (s).

## Supplementary Materials

Supplementary Materials Colorectal cancer differentially expressed LncRNAs.
