# Supplementary material for: LncSNHG1 Promoted CRC Proliferation through the miR-181b-5p/SMAD2 Axis
Source: J Oncol. 2022 Mar 11;2022:4181730. doi: 10.1155/2022/4181730 (PMC8933095; doi:10.1155/2022/4181730)
Supplement: Supplementary Materials — Colorectal cancer differentially expressed LncRNAs. [file 4181730.f1.pdf]

Fold Change cut-off: **2.0**
# P-value cut-off: **0.05**
Condition pairs: **Ca_vs_N**

Column A: ProbeName it represents probe name.
Column B: P-value, P-value calculated from paired t-test.
Column C: FDR, FDR is calculated from Benjamini Hochberg FDR.
Column D: Fold Change, the absolute ratio (no log scale) of normalized intensities between two conditions.
Column E: Regulation, it depicts which group has greater or lower intensity values wrt other group.
Column F ~ X: Annotations, include type, seqname, GeneSymbol, source, RNAlength, chrom, strand, txStart, txEnd, Xhyb, Sequence
Column Y, Z: Raw Intensity of each group (averaged raw intensities of replicate samples).
Column AA, AB: Normalized Intensity of each group (averaged normalized intensities of replicate samples, log2 transformed).
Column AC ~ AN: Raw Intensity of each sample.
Column AO ~ AZ: Normalized Intensity of each sample (log2 transformed).

Note:
Column I: source, the source of LncRNA is collected from.
RefSeq: transcripts collected from RefSeq database;
UCSC_knowngene: UCSC known genes annotated as "non-coding", "near-coding" or "antisense" (http://genome.ucsc.edu/cgi-bin/hgTa
GENCODE: the GENCODE project (http://www.gencodegenes.org/);
pseudogene: pseudogene tagged by GENCODE;
RNAdb: RNAdb2.0 (http://research.imb.uq.edu.au/rnadb/);
NRED: NRED (http://jsm-research.imb.uq.edu.au/nred/cgi-bin/ncrnadb.pl);
UCR: "ultra-conserved region" among human, mouse and rat (http://users.soe.ucsc.edu/~jill/ultra.html);
LincRNAs identified by Khalil et al: lincRNA identified by searching for regions of chromatin methylation (H3K4me3 and H3K36me3
LincRNAs identified by Cabili et al: lincRNA identified by RNA sequencing results and public database information (Cabili et al. 2011

Columns R ~ X: the relationship of LncRNA and its nearby coding gene and the coordinate of the coding gene, including relationship
"exon sense-overlapping": the LncRNA's exon is overlapping a coding transcript exon on the same genomic strand;
"intronic": the LncRNA is overlapping the intron of a coding transcript on the same genomic strand;
"natural antisense": the LncRNA is transcribed from the antisense strand and overlapping with a coding transcript;
"non-overlapping antisense": the LncRNA is transcribed from the antisense strand without sharing overlapping exons;
"bidirectional": the LncRNA is oriented head to head to a coding transcript within 1000 bp;

	P-value and FDR		Fold change and Regulation		
**ProbeName**	**P-value**	**FDR**	**Fold Change**	**Regulation**	**type**
ASHGA5P048339	0.007751341	0.055296161	2.3111905	up	noncoding
ASHGA5P022755	0.019265156	0.084591728	2.1117804	up	noncoding
ASHGA5P025811	0.009535972	0.060604099	2.359045	up	noncoding
ASHGA5P036006	0.033871172	0.116094281	6.8546918	up	noncoding
ASHGA5P031308	0.007618346	0.054807128	4.8948916	up	noncoding
ASHGA5P031308	0.007618346	0.054807128	4.8948916	up	noncoding
ASHGA5P053098	0.002261405	0.031214119	2.1295967	up	noncoding
ASHGA5P044704	0.047013662	0.139917379	2.2288412	up	noncoding
ASHGA5P052532	0.002638213	0.03325916	2.3669846	up	noncoding
ASHGA5P031045	0.03507684	0.11853671	2.6912156	up	noncoding
ASHGA5P016725	0.047808496	0.141601172	3.3080024	up	noncoding
ASHGA5P057014	0.006439967	0.051060618	3.0052361	up	noncoding
ASHGA5P057014	0.006439967	0.051060618	3.0052361	up	noncoding
ASHGA5P057014	0.006439967	0.051060618	3.0052361	up	noncoding
ASHGA5P058383	0.025109046	0.098439971	2.828986	up	noncoding
ASHGA5P029189	0.011239526	0.065435608	3.7370768	up	noncoding
ASHGA5P029189	0.011239526	0.065435608	3.7370768	up	noncoding
ASHGA5P029189	0.011239526	0.065435608	3.7370768	up	noncoding
ASHGA5P028767	0.037740113	0.123372227	2.0600623	up	noncoding
ASHGA5P043319	0.049820831	0.145340561	2.5839818	up	noncoding
ASHGA5P031317	0.016529192	0.078449075	2.1525162	up	noncoding
ASHGA5P031044	0.013792508	0.07198363	2.5825664	up	noncoding
ASHGA5P044460	0.014786507	0.074317791	5.2358818	up	noncoding
ASHGA5P036879	0.002009657	0.029606317	3.446467	up	noncoding

ɛ, EntrezID, relationship, Associated_gene_acc, Associated_gene_name, Associated_protein_name, Associated_gene_strand, Ass

ables/);

3) outside of known protein-coding loci (Guttman et al. 2009; Khalil et al. 2009);
l).

), Associated_gene_acc, Associated_gene_name, Associated_protein_name, Associated_gene_strand, Associated_gene_start and

seqname	GeneSymbol	source	RNAlength	chrom	strand
ENST00000528781	RP11-672A2.6	GENCODE	831	chr11	-
ENST00000562227	ZNF192P1	GENCODE	1113	chr6	+
NR_038209	LOC100616530	RefSeq	377	chr8	+
ENST00000450072	AC017099.3	GENCODE	587	chr2	+
ENST00000454068	GAS5	GENCODE	688	chr1	-
ENST00000454068	GAS5	GENCODE	688	chr1	-
uc003tuh.3	CCT6P1	UCSC_knowngene	1675	chr7	+
ENST00000511897	RP11-87E22.2	GENCODE	3879	chr8	+
ENST00000504756	RP11-423H2.1	GENCODE	489	chr5	+
ENST00000565783	RP11-673P17.2	GENCODE	499	chr16	-
ENST00000433505	RP11-426L16.8	GENCODE	767	chr1	-
TCONS_00011960	XLOC_005473	LincRNAs identified	462	chr6	+
TCONS_00011960	XLOC_005473	LincRNAs identified	462	chr6	+
TCONS_00011960	XLOC_005473	LincRNAs identified	462	chr6	+
uc002lal.1	LOC647946	UCSC_knowngene	2306	chr18	-
NR_046321	GPX2	RefSeq	1310	chr14	-
NR_046321	GPX2	RefSeq	1310	chr14	-
NR_046321	GPX2	RefSeq	1310	chr14	-
ENST00000443576	RP11-141M1.1	GENCODE	466	chr13	+
NR_046216	MGC27345	RefSeq	10079	chr7	-
ENST00000563408	RP11-264L1.4	GENCODE	719	chr16	-
ENST00000443373	AC002331.1	GENCODE	294	chr16	-
ENST00000577199	RP11-1C8.6	GENCODE	777	chr8	-
NR_024470	LOC100127888	RefSeq	1440	chr20	-

ociated_gene_start and Associated_gene_end.

Associated_gene_end.

Annotations					
**txStart**	**txEnd**	**Xhyb**	**Sequence**	**EntrezID**	**relationship**
76336505	76337878		AATTTGTTCTGATCCTAAGGTCACTA		intergenic
28130400	28134936		ACATGAACAGTACCTGCTACATAGG		intergenic
96281063	96822371	NR_038205	ATTCAAGGATTAT	100616530	intronic antisense
98280679	98292184		GCCCTCTCCAAGGAGGCCGGCCCTG		bidirectional
173833048	173836953		GCATGCGTGACCT	60674	bidirectional
173833048	173836953		GCATGCGTGACCT	60674	bidirectional
65216091	65228662	NR_003110, uc003tu	TTTCTTTTCTTCCT	643253	intergenic
22735496	22745535		GGTTAGCTTTTCACTGTGACCCTTAT		intronic antisense
177309391	177310001		CTTTAGACAAGTATGTTTCTGTTTTC		intergenic
27078248	27079179		TTTTCTGGGAGACTCTGGCTGCTGGA		natural antisense
113362791	113392437		GCGCTTAATGCAACGCGAATGAAAT		intergenic
136871938	136872729		AAGTAGCCAGAAACAGATTATTGGC		natural antisense
136871938	136872729		AAGTAGCCAGAAACAGATTATTGGC		natural antisense
136871938	136872729		AAGTAGCCAGAAACAGATTATTGGC		natural antisense
36786887	37380282		ACATTTTTGGCGC	647946	intron sense-overlapp
65405869	65409623		CCATCTCTTCAGC	2877	exon sense-overlappi
65405869	65409623		CCATCTCTTCAGC	2877	intronic antisense
65405869	65409623		CCATCTCTTCAGC	2877	intronic antisense
33907982	33909403		GCTTTGTCTCCTGTGAGGGACTTTTC		intronic antisense
127937737	127947816		GTGATCCTTTGCT	157247	intergenic
78529822	78540465		AGACTGTGGTTATCCGTTTAAAGATC		intronic antisense
26596075	26606134		TATTTATTTGCTTCGAGTCATCCGGA		intergenic
104395305	104396082		TGTCTGATATTCTCTACCAAATGGTA		intergenic
61294378	61297973		AGAACTGACATA	100127888	natural antisense

Associated_gene_acc	ssociated_gene_nan	sociated_protein_na	ssociated_gene_stran	ssociated_gene_star	Associated_gene_enc
NM_177965	C8orf37	protein C8orf37	-	96257140	96281462
NM_005735	ACTR1B	beta-centractin	-	98272401	98280561
NM_001122770	ZBTB37	zinc finger and BTB	+	173837492	173855774
NM_032522	ZBTB37	zinc finger and BTB	+	173837492	173842778
NM_144962	PEBP4	phosphatidylethanola	-	22570764	22785421
NM_001145545	C16orf82	protein TNT	+	27078218	27080487
NM_001198616	MAP7	ensconsin isoform 6	-	136663418	136871957
NM_001198617	MAP7	ensconsin isoform 7	-	136663418	136871957
NM_003980	MAP7	ensconsin isoform 3	-	136663418	136871957
ENST00000321566	AC011139.1		-	36914835	36915639
NM_002083	GPX2	glutathione peroxidas	-	65405869	65409623
NM_001202558	CHURC1-FNTB	CHURC1-FNTB pro	+	65381078	65529373
NM_001202559	CHURC1-FNTB	CHURC1-FNTB pro	+	65381078	65529373
NM_001243476	STARD13	stAR-related lipid tra	-	33677271	34250932
NM_016373	WWOX	WW domain-contain	+	78133326	79246564
NM_016354	SLCO4A1	solute carrier organic	+	61273796	61303647

Ca_vs_N 2.0 fold up regulated LncRNAs					
Group--Raw Intensity		Group--Normalized Intensity			
group-Ca(raw)	group-N(raw)	roup-Ca(normalized	group-N(normalized	D653416ca, Ca](raw	D906882ca, Ca](raw
521.236287	145.618854	8.606321	7.397685	701.23004	708.5937
35.909392	11.69552	4.77936	3.7009	41.489788	39.38305
58.959329	18.396287	5.532493	4.29429	74.237045	65.81788
2978.525267	482.50618	10.791963	8.014871	2854.056	4203.8975
338.514105	25.655149	7.13181	4.840533	574.50964	972.5552
338.514105	25.655149	7.13181	4.840533	574.50964	972.5552
450.98248	134.334785	8.380854	7.290274	567.7949	664.931
27.828951	10.271292	4.44439	3.288096	39.244167	30.575663
177.755123	43.596736	6.988593	5.745543	242.10977	324.24713
268.722891	55.662834	7.231483	5.803225	462.68167	560.0925
111.561014	24.018175	6.127749	4.401789	72.85013	21.392817
301.147738	76.12397	7.851259	6.263781	336.76633	374.6509
301.147738	76.12397	7.851259	6.263781	336.76633	374.6509
301.147738	76.12397	7.851259	6.263781	336.76633	374.6509
35.728591	8.746354	4.66884	3.168555	52.461937	60.489105
733.2356	120.201241	9.030063	7.128153	866.2029	742.7116
733.2356	120.201241	9.030063	7.128153	866.2029	742.7116
733.2356	120.201241	9.030063	7.128153	866.2029	742.7116
43.075468	15.397533	5.06804	4.025352	62.486214	37.331673
44.521967	15.785481	5.073989	3.704394	26.654587	38.05219
100.387207	25.676739	5.821051	4.715027	138.38448	11.686821
25.604005	8.300992	4.311216	2.942411	22.408657	12.41473
72.564953	10.714251	5.68193	3.293498	68.462944	26.004223
129.379177	20.19021	6.381002	4.595884	108.863716	134.41585

				Raw Intensity	
**D896032ca, Ca](raw**	**D907028ca, Ca](raw**	**D907020ca, Ca](raw**	**D907993ca, Ca](raw**	**[D653416N, N](raw)**	**[D906882N, N](raw)**
413.7801	424.84366	440.93668	438.03354	150.22527	59.176517
20.136517	49.170868	41.8576	23.418531	9.9137335	6.5364704
48.38091	60.914238	48.229584	56.176315	17.203497	7.693875
1036.6749	686.7432	4489.353	4600.427	611.4182	105.03289
60.51464	266.76495	28.13043	128.60977	35.467472	37.7445
60.51464	266.76495	28.13043	128.60977	35.467472	37.7445
349.00513	294.79095	421.9564	407.4165	160.45369	92.50319
24.848732	32.173683	23.462017	16.669443	4.9999995	4.9999995
104.163216	111.7233	138.20421	146.08311	46.951527	36.258213
124.60716	39.888096	245.65598	179.41194	61.298462	40.15523
92.18123	75.00262	213.32877	194.61052	8.819644	9.113771
271.45782	222.0042	287.44858	314.5586	81.26021	22.791517
271.45782	222.0042	287.44858	314.5586	81.26021	22.791517
271.45782	222.0042	287.44858	314.5586	81.26021	22.791517
35.474648	32.96204	17.422508	15.56131	7.9417644	4.9999995
826.628	1051.5957	278.1006	634.1748	71.1748	58.766407
826.628	1051.5957	278.1006	634.1748	71.1748	58.766407
826.628	1051.5957	278.1006	634.1748	71.1748	58.766407
32.06283	54.47896	31.355173	40.73796	8.303433	6.0995855
32.59347	73.52329	37.887123	58.421143	6.9330144	8.510804
250.86983	81.437935	43.772835	76.17134	29.109201	8.593746
29.891024	32.169693	21.502033	35.237892	4.9999995	4.9999995
42.51814	125.97678	67.45905	104.96858	8.114567	7.2433486
65.94291	316.6986	39.270664	111.08332	22.374916	19.501812

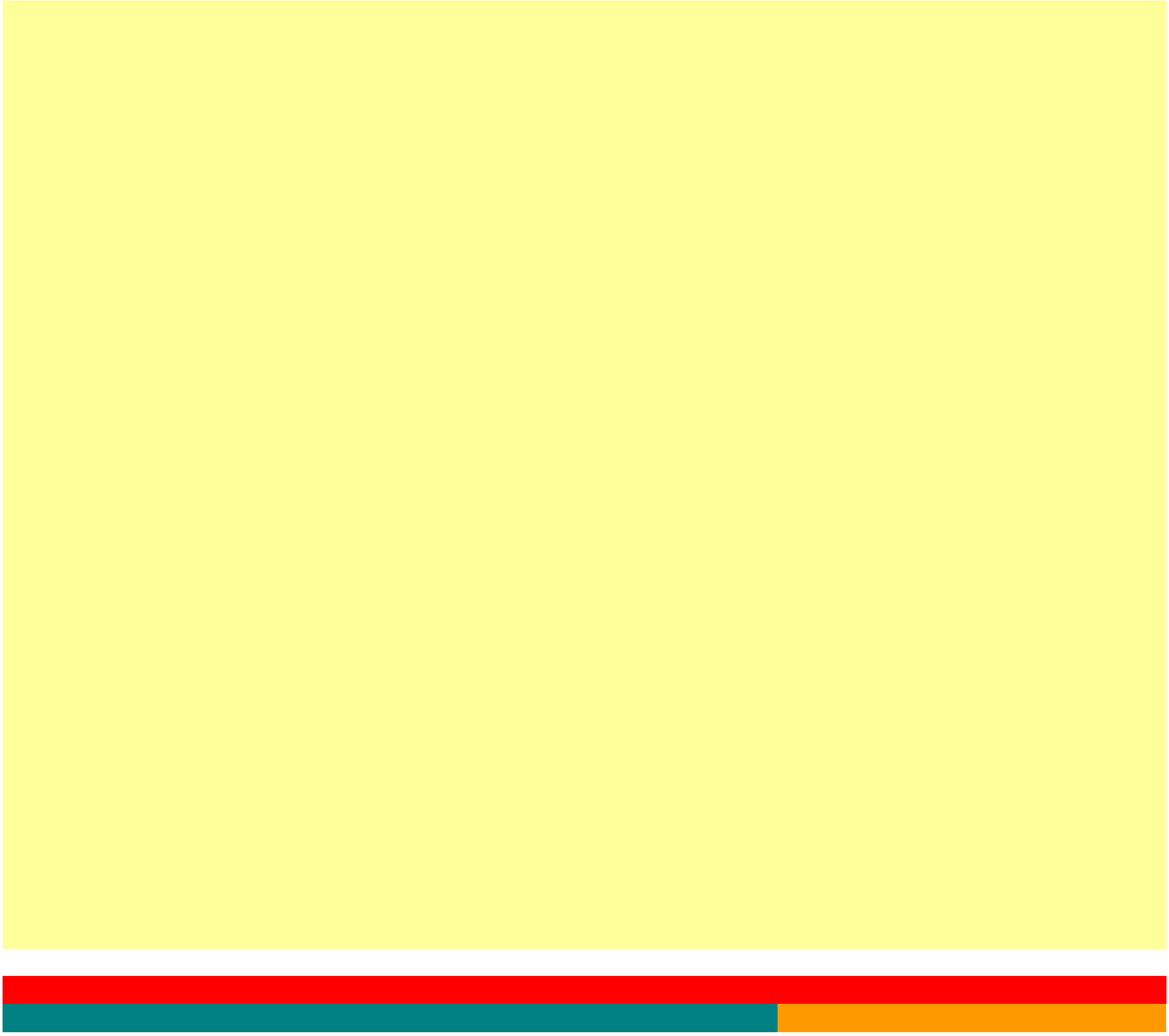

[D896032N, N](raw)	[D907028N, N](raw)	[D907020N, N](raw)	[D907993N, N](raw)	3416ca, Ca](normal	6882ca, Ca](normal
260.0895	135.14519	165.20227	103.87438	8.983642	9.145328
21.121117	11.793231	14.04992	6.7586465	4.912071	5.1963964
40.88422	17.270199	11.833471	15.492462	5.7586894	5.8674517
1886.0549	63.087734	175.0605	54.382854	10.994306	11.60719
6.893377	25.721725	18.35774	29.746078	8.691224	9.583754
6.893377	25.721725	18.35774	29.746078	8.691224	9.583754
236.90973	78.85111	153.86691	83.42408	8.672012	9.054823
18.938002	10.473421	16.001307	6.2150245	4.8327775	4.868408
59.219543	40.592308	46.46869	32.090134	7.4686575	8.060068
141.0243	21.620937	44.088657	25.78942	8.375926	8.818264
10.615991	71.12097	10.09442	34.344254	5.730055	4.3837647
168.43176	38.851326	100.585495	44.82351	7.942898	8.257078
168.43176	38.851326	100.585495	44.82351	7.942898	8.257078
168.43176	38.851326	100.585495	44.82351	7.942898	8.257078
15.327759	5.920527	12.46464	5.823436	5.245638	5.7565713
119.5573	110.42678	221.3739	139.90826	9.29452	9.214978
119.5573	110.42678	221.3739	139.90826	9.29452	9.214978
119.5573	110.42678	221.3739	139.90826	9.29452	9.214978
25.677794	20.025187	22.463808	9.815391	5.509778	5.128319
54.473125	11.180479	8.337253	5.2782083	4.2801223	5.152038
59.859257	20.091463	20.11817	16.288599	6.658198	3.604756
4.9999995	13.414929	16.391022	4.9999995	4.023713	3.6852622
31.18494	4.9999995	7.74265	4.9999995	5.636147	4.6521797
10.739248	30.758354	19.547043	18.219889	6.3198314	6.837971

			Normalized Intensity		
6032ca, Ca](normal	7028ca, Ca](normal	7020ca, Ca](normal	7993ca, Ca](normal	53416N, N](normali	06882N, N](normali
8.350713	8.422825	8.526192	8.209225	7.469465	6.7379923
4.0103726	5.227714	5.229281	4.1003222	3.531339	3.4656787
5.2791033	5.552244	5.420487	5.316983	4.3421073	3.7043612
9.641417	9.1190405	11.829823	11.560001	9.539011	7.560299
5.608146	7.741766	4.685355	6.4806123	5.382128	6.080709
5.608146	7.741766	4.685355	6.4806123	5.382128	6.080709
8.107872	7.8884397	8.462235	8.099743	7.573084	7.3820157
4.3236876	4.563595	4.44253	3.6353405	2.3467326	2.4279919
6.386155	6.4606647	6.8895445	6.666469	5.782229	6.0284534
6.6394205	4.897938	7.6961527	6.961197	6.1719832	6.17163
6.2053905	5.8704934	7.4954977	7.0812917	3.353395	3.9634771
7.7516103	7.483323	7.923947	7.748699	6.568746	5.3382306
7.7516103	7.483323	7.923947	7.748699	6.568746	5.3382306
7.7516103	7.483323	7.923947	7.748699	6.568746	5.3382306
4.8332005	4.6008844	4.0373206	3.5394244	3.189217	2.4279919
9.342271	9.722648	7.874619	8.731342	6.384901	6.7269187
9.342271	9.722648	7.874619	8.731342	6.384901	6.7269187
9.342271	9.722648	7.874619	8.731342	6.384901	6.7269187
4.690253	5.3797708	4.8281054	4.872012	3.259294	3.3587208
4.715403	5.8396645	5.0914106	5.3652983	2.9880579	3.8610795
7.6389804	5.9904175	5.292761	5.7411933	5.093396	3.875915
4.593603	4.563217	4.323081	4.678421	2.3467326	2.4279919
5.0878735	6.6453767	5.876195	6.193809	3.223689	3.6186695
5.735646	7.9841433	5.136381	6.2720404	4.7211723	5.1111193

96032N, N](normali	07028N, N](normali	07020N, N](normali	07993N, N](normalized)
7.97	7.4473476	7.599537	7.161766
4.438443	3.6711743	4.0836406	3.0151227
5.3510427	4.2540455	3.8401752	4.2740088
10.821028	6.277798	7.693179	6.1979117
2.9527395	4.876491	4.4687223	5.282406
2.9527395	4.876491	4.4687223	5.282406
7.8361297	6.625823	7.4934654	6.8311253
4.2917852	3.4934525	4.273472	2.8951416
5.8745117	5.5816936	5.8076115	5.3987575
7.095615	4.6008844	5.731102	5.0481358
3.5091064	6.469005	3.6134408	5.502307
7.344956	5.5159173	6.906013	5.9088225
7.344956	5.5159173	6.906013	5.9088225
7.344956	5.5159173	6.906013	5.9088225
4.002629	2.6742423	3.9158492	2.8013995
6.8613634	7.132372	8.043253	7.6201086
6.8613634	7.132372	8.043253	7.6201086
6.8613634	7.132372	8.043253	7.6201086
4.7061467	4.4840164	4.761637	3.5822957
5.7595925	3.5931666	3.3527057	2.671759
5.890485	4.4885716	4.594253	4.347541
2.350709	3.8679411	4.308338	2.352752
4.9771304	2.3408759	3.247869	2.352752
3.5252707	5.152258	4.5525107	4.512973

ASHGA5P020789	0.00167851	0.027439569	2.7639348	up	noncoding
ASHGA5P044770	0.00208465	0.030144699	2.2903466	up	noncoding
ASHGA5P045216	0.004102171	0.040926621	2.6104313	up	noncoding
ASHGA5P027638	0.001203738	0.023279887	4.6842796	up	noncoding
ASHGA5P032023	0.049850251	0.145360175	2.6579183	up	noncoding
ASHGA5P044836	0.00393204	0.040316445	2.8560585	up	noncoding
ASHGA5P029706	0.002155772	0.030562731	3.1999148	up	noncoding
ASHGA5P029706	0.002155772	0.030562731	3.1999148	up	noncoding
ASHGA5P035715	0.000128767	0.010460795	2.742712	up	noncoding
ASHGA5P035715	0.000128767	0.010460795	2.742712	up	noncoding
ASHGA5P035715	0.000128767	0.010460795	2.742712	up	noncoding
ASHGA5P016824	0.027365906	0.103219977	3.2415092	up	noncoding
ASHGA5P038596	0.024930451	0.097992075	3.5367341	up	noncoding
ASHGA5P053097	0.001028699	0.02179407	2.5260713	up	noncoding
ASHGA5P042967	0.008061411	0.056491889	6.0861372	up	noncoding
ASHGA5P049003	0.011697709	0.066606252	3.2784796	up	noncoding
ASHGA5P049003	0.011697709	0.066606252	3.2784796	up	noncoding
ASHGA5P048122	0.008270553	0.056920478	2.7510408	up	noncoding
ASHGA5P056899	0.024566717	0.097243838	5.5359803	up	noncoding
ASHGA5P053487	0.006673787	0.051928192	2.8806486	up	noncoding
ASHGA5P046701	0.002375545	0.031849965	2.3502404	up	noncoding
ASHGA5P047683	0.014887653	0.074671223	2.4901395	up	noncoding
ASHGA5P042961	0.000275979	0.012375459	13.666813	up	noncoding
ASHGA5P028575	0.013703699	0.071739124	3.2992187	up	noncoding
ASHGA5P018536	0.016819376	0.078960288	31.8043519	up	noncoding
ASHGA5P050997	0.00020401	0.01146874	8.8592287	up	noncoding
ASHGA5P041934	5.05122E-05	0.007392592	4.3340308	up	noncoding
ASHGA5P056162	0.013628042	0.071680418	2.0274044	up	noncoding
ASHGA5P050888	0.003174736	0.036481439	2.4979224	up	noncoding
ASHGA5P053694	0.008772501	0.05835635	2.7886075	up	noncoding
ASHGA5P019742	0.005528331	0.047315963	2.3134817	up	noncoding
ASHGA5P037444	0.040896403	0.129245177	2.1581883	up	noncoding
ASHGA5P037444	0.040896403	0.129245177	2.1581883	up	noncoding
ASHGA5P037444	0.040896403	0.129245177	2.1581883	up	noncoding
ASHGA5P037444	0.040896403	0.129245177	2.1581883	up	noncoding
ASHGA5P038421	0.022859541	0.093651074	2.0412295	up	noncoding
ASHGA5P045335	0.000311642	0.012874016	4.3141789	up	noncoding
ASHGA5P036375	2.18615E-05	0.005043652	2.5921713	up	noncoding
ASHGA5P042233	0.000559579	0.016101946	8.4451224	up	noncoding
ASHGA5P028913	0.04915931	0.144049178	2.2662041	up	noncoding
ASHGA5P026827	0.020367233	0.087543191	2.7534815	up	noncoding
ASHGA5P026642	0.006774947	0.052211732	3.0251255	up	noncoding
ASHGA5P026642	0.006774947	0.052211732	3.0251255	up	noncoding
ASHGA5P052151	0.003558921	0.038481895	3.8070484	up	noncoding
ASHGA5P044524	0.00059047	0.016411961	21.9687645	up	noncoding
ASHGA5P015612	0.021127449	0.089499513	2.3630509	up	noncoding
ASHGA5P029373	0.007792186	0.055480763	2.4587446	up	noncoding
ASHGA5P049522	0.000981435	0.021189195	4.4297651	up	noncoding
ASHGA5P056323	0.00405098	0.040655911	2.2951802	up	noncoding
ASHGA5P056323	0.00405098	0.040655911	2.2951802	up	noncoding
ASHGA5P056323	0.00405098	0.040655911	2.2951802	up	noncoding
ASHGA5P022469	0.015705932	0.076574828	2.2471936	up	noncoding
ASHGA5P045076	0.000453269	0.015001215	2.1097113	up	noncoding
ASHGA5P032987	0.002706646	0.033801833	2.9635411	up	noncoding
ASHGA5P052919	0.001850001	0.028874356	4.4171837	up	noncoding
ASHGA5P030886	0.005605051	0.047487927	2.0961711	up	noncoding
ASHGA5P030886	0.005605051	0.047487927	2.0961711	up	noncoding
ASHGA5P030886	0.005605051	0.047487927	2.0961711	up	noncoding
ASHGA5P027402	0.005271594	0.046214425	2.5169958	up	noncoding
ASHGA5P027402	0.005271594	0.046214425	2.5169958	up	noncoding
ASHGA5P036237	0.018322759	0.082120309	2.0496357	up	noncoding
ASHGA5P031281	0.00094294	0.020844644	3.4828476	up	noncoding

ENST00000519861	RP11-383J24.5	GENCODE	359	chr8	-
ENST00000563059	KB-1836B5.1	GENCODE	911	chr8	+
ENST00000426946	RP11-220I1.2	GENCODE	944	chr9	-
ENST00000535720	RP11-173P15.3	GENCODE	583	chr12	-
ENST00000419190	RP11-465N4.4	GENCODE	788	chr1	-
TCONS_00002142	XLOC_000363	LincRNAs identified	264	chr1	+
ENST00000556926	RP11-356K23.2	GENCODE	458	chr14	+
ENST00000556926	RP11-356K23.2	GENCODE	458	chr14	+
ENST00000414538	AC092687.5	GENCODE	565	chr2	+
ENST00000414538	AC092687.5	GENCODE	565	chr2	+
ENST00000414538	AC092687.5	GENCODE	565	chr2	+
ENST00000434703	AC005152.2	GENCODE	3032	chr17	-
ENST00000421870	EIF2B5-AS1	GENCODE	571	chr3	-
ENST00000419314	CCT6P3	GENCODE	3702	chr7	+
ENST00000446246	AC004593.3	GENCODE	798	chr7	-
ENST00000570072	RP11-304L19.1	GENCODE	600	chr16	+
ENST00000570072	RP11-304L19.1	GENCODE	600	chr16	+
CD642394		LincRNAs identified	752	chr3	-
TCONS_00010362	XLOC_004859	LincRNAs identified	282	chr5	-
ENST00000519854	RP11-37B2.1	GENCODE	284	chr8	-
ENST00000455777	RP13-507I23.1	GENCODE	284	chrX	+
uc001kyd.1	AK094859	UCSC_knowngene	2743	chr10	+
ENST00000523608	RP1-170O19.17	GENCODE	757	chr7	-
ENST00000443679	LINC00345	GENCODE	285	chr13	-
ENST00000454712	RP11-431J24.2	GENCODE	454	chrX	-
ENST00000430481	RP5-881L22.5	GENCODE	370	chr20	-
ENST00000431043	SNHG5	GENCODE	998	chr6	-
TCONS_00027578	XLOC_013095	LincRNAs identified	1145	chr19	+
ENST00000444990	AC093642.5	GENCODE	557	chr2	+
ENST00000452436	AC129778.2	pseudogene	321	chr9	-
uc011ckd.2	NBLA00301	UCSC_knowngene	4252	chr4	+
NR_038880	GCFC1-AS1	RefSeq	2946	chr21	+
NR_038880	GCFC1-AS1	RefSeq	2946	chr21	+
NR_038880	GCFC1-AS1	RefSeq	2946	chr21	+
NR_038880	GCFC1-AS1	RefSeq	2946	chr21	+
uc003ene.2	FAM86HP	UCSC_knowngene	1886	chr3	-
ENST00000433644	RP11-435O5.2	GENCODE	739	chr9	-
ENST00000420509	AC007383.3	GENCODE	752	chr2	+
ENST00000454882	RP3-380B8.4	GENCODE	412	chr6	+
ENST00000417102	CLYBL-IT1	GENCODE	374	chr13	+
TCONS_00011825	XLOC_005311	LincRNAs identified	283	chr6	+
ENST00000529215	CTD-2562J17.7	GENCODE	506	chr11	+
ENST00000529215	CTD-2562J17.7	GENCODE	506	chr11	+
TCONS_00015168	XLOC_007214	LincRNAs identified	1349	chr8	-
uc003ysa.2	CCAT1	UCSC_knowngene	2613	chr8	-
ENST00000421927	MCM3AP-AS1	GENCODE	2280	chr21	+
ENST00000564585	RP11-982M15.8	GENCODE	3816	chr14	-
ENST00000460249	C17orf76-AS1	GENCODE	773	chr17	+
TCONS_00002486	XLOC_001036	LincRNAs identified	875	chr1	-
TCONS_00002486	XLOC_001036	LincRNAs identified	875	chr1	-
TCONS_00002486	XLOC_001036	LincRNAs identified	875	chr1	-
NR_039992	LINC00645	RefSeq	3555	chr14	+
NR_015445	BREA2	RefSeq	1002	chr8	+
TCONS_00029124	XLOC_014070	LincRNAs identified	531	chr21	-
uc003qvy.1	AL832737	UCSC_knowngene	3867	chr6	-
ENST00000562485	RP11-20I23.8	GENCODE	9768	chr16	-
ENST00000562485	RP11-20I23.8	GENCODE	9768	chr16	-
ENST00000562485	RP11-20I23.8	GENCODE	9768	chr16	-
ENST00000489520	RPSAP52	GENCODE	1144	chr12	-
ENST00000489520	RPSAP52	GENCODE	1144	chr12	-
TCONS_00026864	XLOC_012912	LincRNAs identified	487	chr19	+
ENST00000422207	GAS5	GENCODE	643	chr1	-

125485048	125486595		CATCTTTGAGCAAAAGGAGAGAGG(	bidirectional
37920398	37921309		GCTTACAACCTGATCAGGAGACGAC	intergenic
37112914	37115487	ENST00000426358	GCTGCATTTCAAAATTGAACTTTAG(	intergenic
121136066	121137344		AAATAAAGAATCCCACCCCTTTAAC	natural antisense
201969221	201979481		AGAGATTCAGCAACAAAGAGGCCTA	intronic antisense
117647168	117647504		TGTCCTCATGAGGCTTAGCATCTAA(	intergenic
89821403	89822915		ACAATTGTAACCCTGTAAAGCTGAT	intronic antisense
89821403	89822915		ACAATTGTAACCCTGTAAAGCTGAT	intronic antisense
10729291	10744956		CAAACATAACTTTAAATCGATCATC(	natural antisense
10729291	10744956		CAAACATAACTTTAAATCGATCATC(	natural antisense
10729291	10744956		CAAACATAACTTTAAATCGATCATC(	natural antisense
70067183	70112157		AGGTAAAGCGCAGCCAAAAACAATC	intergenic
184264501	184274706		CCAGGGGCCCATGACTGTAGTCACC	intronic antisense
64498731	64535091		CAGGACTTGTATA 643180	intergenic
29239155	29248586		GGACTAGTACTCCTCATCTGAGTAA	intronic antisense
2141436	2145018		AATGCTTCCTGTTTTGCTGTCTCTCG	natural antisense
2141436	2145018		AATGCTTCCTGTTTTGCTGTCTCTCG	natural antisense
163907665	163908407		GTGTTTCACCAATGCTCTTCAGTTAT	intergenic
67804064	67816089	TCONS_00010945	TGGAGGAGAGATAAGGAGTTCATAT	intergenic
90629221	90635433	ENST00000520306	TTTGAAGAAGGCAAGCTTCATTAAA	intergenic
149129099	149131017		CTCAGCGTTAATTTGTAAGCCTTTG(	intergenic
106061961	106064704		CCCAAGGCCAAGTTTAATACTGTGC	intergenic
27278861	27280847		TGTTTTCGCTTCTCTTTGGTTAAAAA	intergenic
53058295	53058815		CATAAACTTATGTTTGCAAAGCACC	intergenic
16171342	16183244		GGGAAGAGGAGTTGGATTTGGGAA(	natural antisense
42979361	42980742		CACTTCTCAGTTCTGAGAGCCGAGG	natural antisense
86386845	86388451		CTTTTACATATGTTGTAATGTTTGTA	intergenic
45240861	45249208		ACTTCCAGATGGAATTTCGTTTCCCT	intergenic
243030829	243058523		CACAAAAGAAGTAAGTATTCTATTA	intergenic
42853630	42856292		TTCATTTACCCTTTGGTCGGAACGGTGAGTGAGTCACA	
174451608	174462981	ENST00000514673,	ACTCCAGAAAGA(79804	bidirectional
34100424	34105224		AGTACTAGTCACT 100506215	bidirectional
34100424	34105224		AGTACTAGTCACT 100506215	bidirectional
34100424	34105224		AGTACTAGTCACT 100506215	bidirectional
34100424	34105224		AGTACTAGTCACT 100506215	bidirectional
129816624	129822720	ENST00000427587,	GGGATGTAGCGA 729375	natural antisense
98177780	98189078		TTTCAATAGTCCAGACAGCTTCCAA(	intergenic
206949328	206951288		TTCCTCTCCGCCGGAGCTGTCCAGA	natural antisense
5851738	5870453		GCCCACACATAAGCTGAAGTATTGC	intergenic
100426278	100427543		CCTGAGAATGTTTTCTGCCACTCAA	intron sense-overlapp
47824776	47838098		ATGGGAAATGGTGTTGGATCAGCAC	intergenic
74971170	74972069		TGTGTAAAGATGAATTTAACCCTAG	natural antisense
74971170	74972069		TGTGTAAAGATGAATTTAACCCTAG	natural antisense
128153590	128197980		TCAGCCTCCCTGGACTGTTACATAA	intergenic
128219628	128231333		AGGAAGGCTCGCTAGAATACTTTGT	intergenic
47649160	47662913		GAGTGCGAAGCA 114044	natural antisense
105127456	105131895		GACAGGAAAACGTAATGTTATAAAC	intergenic
16342640	16344880	ENST00000492250	TTTTCTGAAGATA 125144	intergenic
154671124	154675050		TAAAATACTCAAAATCCTAGAAGGT	exon sense-overlappi
154671124	154675050		TAAAATACTCAAAATCCTAGAAGGT	exon sense-overlappi
154671124	154675050		TAAAATACTCAAAATCCTAGAAGGT	exon sense-overlappi
28081793	28108842		AATGTAAACAGA(100505967	intergenic
144779284	144780583		CTGCAATATTCTT 286076	intergenic
38411520	38414294		AATTCAGAGACCATGAAGTGAAACT	intergenic
168080305	168096970		GATGGACACAGAATCCTGAACTATG	intergenic
2604068	2616295		TCTCCATTTTTGGCATAACTAAGGC(	natural antisense
2604068	2616295		TCTCCATTTTTGGCATAACTAAGGC(	natural antisense
2604068	2616295		TCTCCATTTTTGGCATAACTAAGGC(	natural antisense
66151799	66220754		TCTTGCACGGACT 204010	intronic antisense
66151799	66220754		TCTTGCACGGACT 204010	intronic antisense
1261353	1262238		TCAAAGATGGGAGGAAAGGCGAGG(	intergenic
173833041	173834044	ENST00000412059,	GATGGGAGTGAT' 60674	intergenic

NM_007218	RNF139	E3 ubiquitin-protein	+	125487007	125500859
NM_014730	MLEC	malectin precursor	+	121124948	121139667
NM_020216	RNPEP	aminopeptidase B	+	201951765	201975275
NM_001085471	FOXN3	forkhead box protein	-	89622515	90085494
NM_005197	FOXN3	forkhead box protein	-	89622515	89883454
NM_001261392	NOL10	nucleolar protein 10	-	10710891	10830113
NM_001261394	NOL10	nucleolar protein 10	-	10710891	10830113
NM_024894	NOL10	nucleolar protein 10	-	10710891	10830113
ENST00000444495	EIF2B5	eukaryotic translatior	+	183853163	184402546
NM_004067	CHN2	beta-chimaerin isofoı	+	29234120	29553944
NM_000296	PKD1	polycystin-1 isoform	-	2138710	2185899
NM_001009944	PKD1	polycystin-1 isoform	-	2138710	2185899
NM_005314	GRPR	gastrin-releasing pep	+	16141423	16171641
NM_178491	R3HDML	peptidase inhibitor R	+	42965625	42979875
TTTTCCAAAATCCCTCCCCATT					
NM_021973	HAND2	heart- and neural cres	-	174447651	174451378
NM_001160302	SYNJ1	synaptojanin-1 isofoı	-	34001068	34100250
NM_001160306	SYNJ1	synaptojanin-1 isofoı	-	34001068	34100250
NM_003895	SYNJ1	synaptojanin-1 isofoı	-	34001068	34100351
NM_203446	SYNJ1	synaptojanin-1 isofoı	-	34001068	34100351
NM_001136152	ALG1L2	putative glycosyltran	+	129800673	129817233
NM_017759	INO80D	INO80 complex subu	-	206858444	206950906
NM_206808	CLYBL	citrate lyase subunit l	+	100258918	100549388
NM_004041	ARRB1	beta-arrestin-1 isofor	-	74971165	75062875
NM_020251	ARRB1	beta-arrestin-1 isofor	-	74971165	75062875
NM_003906	MCM3AP	80 kDa MCM3-assoc	-	47655047	47705236
NM_001204087	KCNN3	small conductance ca	-	154669941	154842754
NM_002249	KCNN3	small conductance ca	-	154669941	154842754
NM_170782	KCNN3	small conductance ca	-	154669941	154832338
NM_001261816	PDPK1	3-phosphoinositide-d	+	2587964	2653191
NM_002613	PDPK1	3-phosphoinositide-d	+	2587964	2653191
NM_031268	PDPK1	3-phosphoinositide-d	+	2587964	2653191
NM_003483	HMGA2	high mobility group p	+	66218239	66360071
NM_003484	HMGA2	high mobility group p	+	66218239	66309307

1252.796727	262.525294	9.753727	8.287003	861.8564	628.0028
150.933928	41.148897	6.848699	5.653134	159.3981	127.43641
30.964935	7.979785	4.597665	3.213377	46.639114	28.424562
416.558685	57.01679	8.230183	6.002356	596.93335	552.93634
289.704707	87.133784	7.782154	6.371857	284.63934	246.38773
46.118671	12.709844	5.122739	3.608714	39.98905	39.37692
2736.6989	489.703955	10.90292	9.224887	2179.9688	1128.833
2736.6989	489.703955	10.90292	9.224887	2179.9688	1128.833
84.408864	19.545019	6.018928	4.563325	76.840034	104.2975
84.408864	19.545019	6.018928	4.563325	76.840034	104.2975
84.408864	19.545019	6.018928	4.563325	76.840034	104.2975
87.515493	19.869387	6.037445	4.34078	97.546	114.12531
121.851631	12.05868	5.639105	3.816687	213.04868	365.142
256.958597	67.154073	7.629384	6.292489	251.67517	237.67331
110.247376	12.224558	6.234939	3.629412	82.93091	113.58381
5622.429067	913.36139	11.776481	10.063454	7117.032	11283.5625
5622.429067	913.36139	11.776481	10.063454	7117.032	11283.5625
23.555232	6.183385	4.139886	2.679908	38.79897	22.817408
80.580348	5.942437	5.090341	2.621502	139.64807	14.021721
193.650589	37.209461	6.831187	5.304794	92.968376	99.91898
113.041969	31.221171	6.398968	5.16616	94.84593	50.225235
1152.654867	340.493157	9.775169	8.458942	1162.0889	1139.1597
2893.344209	79.859348	8.517815	4.74521	89.89551	116.93899
47.884782	8.07107	4.89109	3.168965	66.752945	49.853775
2244.606106	12.242546	8.755789	3.764637	4453.1816	105.857124
169.89079	11.285549	6.932612	3.785431	216.05371	266.06107
1755.961983	255.308592	10.363214	8.247504	1839.2551	1574.528
30.635803	12.054478	4.610554	3.59092	36.145473	25.799778
316.776365	73.591512	7.806624	6.485896	270.95404	243.57918
324.694445	64.963747	7.808699	6.329154	195.40427	177.18216
168.365298	51.082545	7.024097	5.814031	174.82643	154.99406
114.495731	28.919695	6.2543	5.14448	152.0358	32.890064
114.495731	28.919695	6.2543	5.14448	152.0358	32.890064
114.495731	28.919695	6.2543	5.14448	152.0358	32.890064
114.495731	28.919695	6.2543	5.14448	152.0358	32.890064
970.860557	328.350646	9.48662	8.457181	1051.5289	1116.6976
64.509825	9.312461	5.563767	3.454681	72.02557	94.993256
19.97982	5.590855	3.986813	2.612652	21.155407	18.285019
427.467372	28.74874	8.183641	5.105523	567.73303	374.55658
28.628572	13.497291	4.404381	3.224103	32.47304	19.244263
33.708019	9.711667	4.719659	3.258402	39.18232	44.36817
2938.775267	695.900737	11.077279	9.480284	3295.9956	3822.5562
2938.775267	695.900737	11.077279	9.480284	3295.9956	3822.5562
202.400201	31.338249	7.13503	5.206357	166.04391	202.39459
4016.349183	131.431215	11.471286	7.013904	5166.988	5681.437
27.64921	8.507625	4.431145	3.190495	26.978218	41.515892
64.882303	16.608411	5.596261	4.298339	57.737877	47.594387
388.77416	52.692212	8.124631	5.977401	481.05786	698.84406
468.613823	137.474601	8.458878	7.26027	564.4654	500.70444
468.613823	137.474601	8.458878	7.26027	564.4654	500.70444
468.613823	137.474601	8.458878	7.26027	564.4654	500.70444
22.135826	7.790851	4.121544	2.95342	28.64463	22.236095
268.330752	78.971117	7.690023	6.612977	256.9652	302.4342
35.072775	7.84852	4.70434	3.137018	31.828264	17.82773
49.76435	8.437647	5.210027	3.066901	34.06683	20.077078
63.844793	20.357945	5.55712	4.489363	78.43266	75.74527
63.844793	20.357945	5.55712	4.489363	78.43266	75.74527
63.844793	20.357945	5.55712	4.489363	78.43266	75.74527
278.457837	64.220164	7.658888	6.327186	292.8634	131.23592
278.457837	64.220164	7.658888	6.327186	292.8634	131.23592
71.24371	21.744371	5.735521	4.700153	79.87659	115.9175
10086.76757	1631.226683	12.829926	11.029658	9223.982	7960.182

971.829	2321.5935	958.11536	1775.3833	234.64746	95.335815
185.13202	93.57077	177.7822	162.28407	29.249651	45.067474
35.686764	28.95411	24.807653	21.277409	8.059631	7.113857
527.67773	314.2007	229.96259	277.6414	55.62727	21.35382
318.66547	396.3472	187.2161	304.9724	66.58109	18.004858
38.26995	28.982182	68.20511	61.888813	8.257802	4.9999995
2531.6353	3360.7314	2842.4204	4376.6045	528.8294	292.76566
2531.6353	3360.7314	2842.4204	4376.6045	528.8294	292.76566
70.30065	58.492767	98.02491	98.49732	19.217611	17.47701
70.30065	58.492767	98.02491	98.49732	19.217611	17.47701
70.30065	58.492767	98.02491	98.49732	19.217611	17.47701
52.881264	113.4631	50.807274	96.27001	6.220891	11.866141
25.196224	11.966658	63.16222	52.594006	13.993921	18.052893
227.0514	205.91647	308.0949	311.34033	65.51407	37.77775
36.007156	208.19832	70.08443	150.67963	16.132147	9.480113
1393.2355	4482.05	4288.5767	5170.1177	669.89166	512.4419
1393.2355	4482.05	4288.5767	5170.1177	669.89166	512.4419
30.56769	17.301245	10.994911	20.851168	4.9999995	4.9999995
263.31268	21.962345	18.468557	26.068714	4.9999995	4.9999995
83.45571	506.9604	75.36392	303.23615	28.126629	33.324963
105.895195	139.77751	119.253334	168.25461	34.179127	13.674662
1140.644	1126.2333	1062.7208	1285.0825	363.91476	72.05472
33.812572	10629.885	509.65518	5979.878	6.43878	6.3414607
92.83226	4.9999995	43.628654	29.241058	10.57146	9.259715
8223.55	89.92107	331.7198	263.40704	8.40451	13.903118
152.961	194.32687	67.11926	122.82283	9.54514	11.766289
1998.2858	1885.6049	1305.323	1932.7751	239.04916	119.361694
37.07647	32.05904	22.75026	29.983799	11.51545	4.9999995
250.11699	548.3152	139.38113	448.31165	67.53691	35.959774
193.72708	279.10226	532.0155	570.7354	55.113293	63.560345
174.16595	118.94802	188.50255	198.75478	47.958557	20.517653
236.97214	65.194725	93.99248	105.889175	25.944609	24.9272
236.97214	65.194725	93.99248	105.889175	25.944609	24.9272
236.97214	65.194725	93.99248	105.889175	25.944609	24.9272
236.97214	65.194725	93.99248	105.889175	25.944609	24.9272
795.61584	570.0902	1255.1499	1036.0809	282.84735	119.078575
40.263344	33.72821	71.28912	74.75945	10.446461	11.8515415
18.97806	31.160698	15.010378	15.28936	4.9999995	5.250387
730.26373	456.84735	127.654274	307.74927	23.125238	31.758312
43.167435	42.431366	10.95874	23.49659	18.809702	4.9999995
32.911682	37.840893	21.636522	26.308529	9.208185	4.9999995
2506.1365	1955.8143	3331.292	2720.857	737.06793	245.54492
2506.1365	1955.8143	3331.292	2720.857	737.06793	245.54492
116.80039	88.813774	365.98608	274.36246	40.178585	47.292843
4095.8293	1999.1709	3501.1543	3653.5156	183.53401	305.80127
19.733019	28.989588	23.16328	25.515266	11.149279	4.9999995
59.115604	116.60001	33.847458	74.39848	15.172	9.082616
258.1584	296.74118	254.37856	343.4649	49.079567	34.978073
460.67508	578.07764	296.18854	411.57184	156.61925	57.531384
460.67508	578.07764	296.18854	411.57184	156.61925	57.531384
460.67508	578.07764	296.18854	411.57184	156.61925	57.531384
17.508371	30.374304	13.653338	20.39822	4.9999995	4.9999995
209.89352	316.43933	220.27896	303.9733	76.445015	54.983242
46.419	21.333427	50.40979	42.61844	9.047537	7.2090716
56.130714	60.335476	62.49464	65.48136	10.664081	4.9999995
91.108055	29.157742	56.89219	51.732838	19.235474	9.651351
91.108055	29.157742	56.89219	51.732838	19.235474	9.651351
91.108055	29.157742	56.89219	51.732838	19.235474	9.651351
392.5064	179.1634	326.37994	348.59796	56.57129	51.176537
392.5064	179.1634	326.37994	348.59796	56.57129	51.176537
51.297977	45.946953	65.67704	68.7462	15.78692	16.243258
9779.11	14155.442	6946.5234	12455.366	1249.8411	1167.5133

378.38202	241.81427	365.8475	259.1247	9.289892	8.977501
46.88204	34.513958	56.992275	34.187984	6.857848	6.764355
4.9999995	12.718191	8.527787	6.459244	5.086096	4.7713084
106.36039	43.728916	67.745834	47.28451	8.747626	8.801858
131.83757	29.032957	164.14182	113.20441	7.695909	7.679269
19.988197	6.128808	23.426125	13.45813	4.8611603	5.196118
857.9394	531.4233	316.53287	410.7331	10.614377	9.791146
857.9394	531.4233	316.53287	410.7331	10.614377	9.791146
23.442646	13.401679	17.61738	26.113789	5.8094444	6.49028
23.442646	13.401679	17.61738	26.113789	5.8094444	6.49028
23.442646	13.401679	17.61738	26.113789	5.8094444	6.49028
30.970837	10.846929	36.01606	23.295467	6.159145	6.613652
13.00847	8.62468	11.676869	6.995246	7.285737	8.2166
118.80669	66.06052	75.90135	38.864056	7.518346	7.630635
26.413712	9.002286	4.9999995	7.319089	5.922473	6.606063
686.04443	581.71735	2029.8358	1000.2372	12.312792	13.010322
686.04443	581.71735	2029.8358	1000.2372	12.312792	13.010322
8.353088	4.9999995	8.747227	4.9999995	4.814247	4.4714456
4.9999995	9.357974	4.9999995	6.2966475	6.6725907	3.8417947
8.944802	82.62437	28.585585	41.650417	6.0912414	6.429155
44.737114	22.19475	42.834194	29.70718	6.1217422	5.5164785
689.75134	183.80014	477.22638	256.2116	9.706196	9.802824
4.9999995	319.2478	9.341758	132.78629	6.036092	6.6469007
5.810785	4.9999995	12.78446	4.9999995	5.601547	5.5054727
4.9999995	12.53985	21.718386	11.88941	11.635123	6.5086913
9.691657	10.899209	13.736268	12.07473	7.3039308	7.791562
433.5139	196.67534	335.2143	208.03716	10.365895	10.231895
25.192402	10.28695	11.48306	8.849005	4.7122507	4.6408205
84.52888	91.19934	88.56417	73.76	7.623695	7.661812
47.561317	104.45039	64.85993	54.237206	7.161021	7.2278786
109.316	54.34469	38.80315	35.555218	6.996277	7.039657
40.0551	31.10068	29.27591	22.214668	6.7922	4.9628887
40.0551	31.10068	29.27591	22.214668	6.7922	4.9628887
40.0551	31.10068	29.27591	22.214668	6.7922	4.9628887
40.0551	31.10068	29.27591	22.214668	6.7922	4.9628887
808.1593	199.49414	344.09665	216.42786	9.565301	9.775599
4.9999995	10.988438	7.9824924	9.605833	5.710286	6.356907
4.9999995	8.294748	4.9999995	4.9999995	3.9436824	4.1855907
23.505817	50.150192	22.244946	21.707937	8.671812	8.256527
42.174046	4.9999995	4.9999995	4.9999995	4.560942	4.2503815
17.422232	4.9999995	14.8093195	6.8302665	4.8295135	5.354347
1902.2445	429.97327	502.7358	357.838	11.205994	11.487032
1902.2445	429.97327	502.7358	357.838	11.205994	11.487032
29.58647	22.9609	30.536951	17.473742	6.9186983	7.409607
32.195744	133.66492	49.778625	83.612724	11.842582	12.013407
13.18505	6.7592854	8.019631	6.9325047	4.2987247	5.2664766
18.896067	15.086981	22.58691	18.825891	5.39022	5.445795
54.836437	34.411884	86.11454	56.73277	8.431667	9.125894
289.1936	114.13418	126.37401	80.99518	8.661674	8.657427
289.1936	114.13418	126.37401	80.99518	8.661674	8.657427
289.1936	114.13418	126.37401	80.99518	8.661674	8.657427
13.171728	10.412422	4.9999995	8.160959	4.387575	4.438697
90.11311	77.36656	109.185684	65.73309	7.547325	7.969258
8.64123	4.9999995	12.19328	4.9999995	4.533784	4.15385
7.603711	5.181579	17.17651	4.9999995	4.6282687	4.304326
38.6149	14.789759	25.807999	14.048188	5.839066	6.0556455
38.6149	14.789759	25.807999	14.048188	5.839066	6.0556455
38.6149	14.789759	25.807999	14.048188	5.839066	6.0556455
84.18552	73.78892	65.73061	53.868107	7.739571	6.8024464
84.18552	73.78892	65.73061	53.868107	7.739571	6.8024464
13.391112	28.575354	26.859716	29.609869	5.867154	6.6345744
1237.8507	1819.9514	2463.3796	1848.824	12.686368	12.506136

9.553047	10.847548	9.652062	10.2023115	8.127777	7.431106
7.211899	6.1961746	7.2457833	6.816137	5.1000314	6.340822
4.8407536	4.3960357	4.519867	3.9719284	3.2127426	3.5922976
8.688299	7.973553	7.5989847	7.5707755	6.0354953	5.2479434
7.980965	8.313351	7.3146706	7.708761	6.2935014	4.990901
4.9453607	4.3975544	5.8923397	5.4439034	3.2519693	2.4279919
10.957084	11.374494	11.187539	11.492882	9.32295	9.056586
10.957084	11.374494	11.187539	11.492882	9.32295	9.056586
5.8233075	5.489619	6.3996673	6.1012526	4.5017676	4.9488564
5.8233075	5.489619	6.3996673	6.1012526	4.5017676	4.9488564
5.8233075	5.489619	6.3996673	6.1012526	4.5017676	4.9488564
5.4061484	6.4877257	5.4888678	6.0691323	2.837554	4.3593707
4.3429213	2.973595	5.788062	5.227714	4.032938	4.99504
7.5030527	7.371356	8.019993	7.732921	6.2712216	6.0823936
4.8544374	7.3872867	5.930689	6.7086825	4.244479	4.0217915
10.074331	11.782421	11.764259	11.714763	9.673226	9.866077
10.074331	11.782421	11.764259	11.714763	9.673226	9.866077
4.626699	3.5683482	3.4124196	3.946155	2.3467326	2.4279919
7.7092113	3.9568033	4.114166	4.2474794	2.3467326	2.4279919
6.0656867	8.670306	6.030626	7.7001095	5.0491295	5.8961296
6.408125	6.8006306	6.675176	6.8716574	5.326611	4.576132
9.782292	9.813189	9.798503	9.748009	8.77046	7.0156975
4.7650294	13.0087	8.737953	11.912215	2.8840306	3.4165633
6.214041	2.3221264	5.2877893	4.4155617	3.6284132	3.987037
12.6373005	6.1379814	8.119947	7.4956903	3.280585	4.605299
6.9265094	7.283399	5.8689103	6.4213634	3.473377	4.3487344
10.606932	10.558912	10.080638	10.335009	8.152884	7.747083
4.896984	4.558445	4.399888	4.4549365	3.753536	2.4279919
7.6343865	8.777067	6.902346	8.240439	6.3137927	6.014154
7.28013	7.807702	8.802414	8.5730505	6.021134	6.847015
7.1137023	6.5618067	7.324194	7.1089425	5.8165784	5.1892786
7.565162	5.6542716	6.3439355	6.2073436	4.9392123	5.469978
7.565162	5.6542716	6.3439355	6.2073436	4.9392123	5.469978
7.565162	5.6542716	6.3439355	6.2073436	4.9392123	5.469978
7.565162	5.6542716	6.3439355	6.2073436	4.9392123	5.469978
9.286069	8.835735	10.022337	9.434678	8.403776	7.7437453
5.015009	4.634572	5.9524164	5.7134132	3.612193	4.3572674
3.9265957	4.5179296	3.8354495	3.5116293	2.3467326	3.1219954
9.159653	8.52151	6.7729	7.719443	4.7729096	5.825639
5.1083484	4.994153	3.4077873	4.104673	4.467273	2.4279919
4.728513	4.813502	4.331605	4.2604713	3.4162364	2.4279919
10.946661	10.606144	11.407322	10.810523	9.828565	8.792931
10.946661	10.606144	11.407322	10.810523	9.828565	8.792931
6.549296	6.1198745	8.260652	7.552053	5.563854	6.412642
11.64623	10.633326	11.46652	11.225648	7.768665	9.117244
3.9817667	4.3981028	4.4240303	4.2177706	3.7086937	2.4279919
5.5727687	6.5321217	4.9315906	5.70507	4.1588993	3.9583216
7.6788616	7.89756	7.7494173	7.864388	5.8500137	5.971114
8.495224	8.855517	7.9683747	8.11505	7.5343547	6.6984277
8.495224	8.855517	7.9683747	8.11505	7.5343547	6.6984277
8.495224	8.855517	7.9683747	8.11505	7.5343547	6.6984277
3.8092117	4.4760723	3.7015316	3.916177	2.3467326	2.4279919
7.397959	7.983368	7.5375977	7.7046285	6.4802055	6.632056
5.215388	3.908677	5.47989	4.93445	3.3929594	3.6124694
5.495415	5.5366125	5.7727785	5.5227637	3.6408224	2.4279919
6.1921954	4.4081416	5.64297	5.204701	4.503066	4.0511146
6.1921954	4.4081416	5.64297	5.204701	4.503066	4.0511146
6.1921954	4.4081416	5.64297	5.204701	4.503066	4.0511146
8.2645235	7.161613	8.100489	7.884687	6.058771	6.5258894
8.2645235	7.161613	8.100489	7.884687	6.058771	6.5258894
5.3637314	5.1170425	5.8397994	5.590824	4.211194	4.839772
12.930331	13.453012	12.442215	12.961493	10.586285	11.067009

8.514635	8.340542	8.752054	8.555906
5.5498486	5.3328195	6.098575	5.4967046
2.350709	3.7853856	3.385948	2.953177
6.6984277	5.69662	6.348827	5.9868197
6.998948	5.067354	7.590277	7.2901635
4.365098	2.720789	4.823522	4.062913
9.687518	9.512049	8.548897	9.221321
9.687518	9.512049	8.548897	9.221321
4.5808525	3.8667085	4.412364	5.069403
4.5808525	3.8667085	4.412364	5.069403
4.5808525	3.8667085	4.412364	5.069403
4.9682894	3.5465562	5.445795	4.887112
3.7794375	3.2048185	3.8201752	3.0677135
6.8519626	6.350757	6.5072217	5.691375
4.745681	3.2656574	2.3618152	3.1370463
9.366946	9.649572	11.2515955	10.57331
9.366946	9.649572	11.2515955	10.57331
3.1921983	2.3408759	3.4188986	2.352752
2.350709	3.324197	2.3618152	2.9175668
3.280585	6.6954885	5.113602	5.793828
5.4796605	4.6418257	5.6926494	5.280081
9.376487	7.912108	9.1374	8.541501
2.350709	8.767781	3.509996	7.5421805
2.7522485	2.3408759	3.952465	2.352752
2.350709	3.7653742	4.7130036	3.872849
3.3890648	3.5546107	4.0515842	3.8952172
8.715908	8.01892	8.6244755	8.225754
4.67796	3.4654038	3.7958965	3.4247332
6.36874	6.8419237	6.722024	6.654739
5.57109	7.054309	6.2873197	6.194059
6.7363176	6.04195	5.5462823	5.5537786
5.321369	5.1697507	5.149868	4.816699
5.321369	5.1697507	5.149868	4.816699
5.321369	5.1697507	5.149868	4.816699
5.321369	5.1697507	5.149868	4.816699
9.6058235	8.041357	8.660897	8.28749
2.350709	3.5682375	3.2902184	3.5494623
2.350709	3.1419067	2.3618152	2.352752
4.5842423	5.919529	4.7470345	4.7837806
5.3939104	2.3408759	2.3618152	2.352752
4.1748376	2.3408759	4.1608634	3.0296052
10.831475	9.19941	9.207486	9.021839
10.831475	9.19941	9.207486	9.021839
4.9061356	4.6980534	5.2080727	4.4493856
5.021933	7.429501	5.911343	6.834736
3.7980187	2.857306	3.2973657	3.0535913
4.2881074	4.0541277	4.7693167	4.561262
5.767784	5.326723	6.682615	6.266157
8.123905	7.1851745	7.2259994	6.793761
8.123905	7.1851745	7.2259994	6.793761
8.123905	7.1851745	7.2259994	6.793761
3.7969644	3.4840775	2.3618152	3.3029366
6.4606647	6.5964704	7.026403	6.482063
3.2374547	2.3408759	3.8855953	2.352752
3.0686224	2.5365648	4.37465	2.352752
5.275046	4.0225964	4.9588814	4.1254764
5.275046	4.0225964	4.9588814	4.1254764
5.275046	4.0225964	4.9588814	4.1254764
6.3618455	6.525436	6.306364	6.1848073
6.3618455	6.525436	6.306364	6.1848073
3.8166988	5.0429378	5.016523	5.273795
10.216973	11.335681	11.518818	11.453185

ASHGA5P046133	0.008990825	0.05919444	11.7718565	up	noncoding
ASHGA5P029325	0.002797269	0.034179323	2.0786741	up	noncoding
ASHGA5P055104	0.029429593	0.107331823	2.4708344	up	noncoding
ASHGA5P050598	0.01306539	0.069928519	2.0562954	up	noncoding
ASHGA5P033854	0.00755719	0.054639687	2.3729655	up	noncoding
ASHGA5P021723	0.000398132	0.014223693	2.7579139	up	noncoding
ASHGA5P039775	0.000541156	0.015984419	7.0899433	up	noncoding
ASHGA5P015696	0.034654006	0.117788629	2.6271273	up	noncoding
ASHGA5P015696	0.034654006	0.117788629	2.6271273	up	noncoding
ASHGA5P033709	0.013799809	0.07198363	2.2973529	up	noncoding
ASHGA5P033709	0.013799809	0.07198363	2.2973529	up	noncoding
ASHGA5P033709	0.013799809	0.07198363	2.2973529	up	noncoding
ASHGA5P033709	0.013799809	0.07198363	2.2973529	up	noncoding
ASHGA5P033709	0.013799809	0.07198363	2.2973529	up	noncoding
ASHGA5P033709	0.013799809	0.07198363	2.2973529	up	noncoding
ASHGA5P033709	0.013799809	0.07198363	2.2973529	up	noncoding
ASHGA5P033709	0.013799809	0.07198363	2.2973529	up	noncoding
ASHGA5P027910	0.000966602	0.021093715	2.5212881	up	noncoding
ASHGA5P015290	0.013102828	0.070034815	2.7472528	up	noncoding
ASHGA5P000495	0.007206028	0.053475878	3.0629955	up	noncoding
ASHGA5P015671	0.006916746	0.05278746	24.8156757	up	noncoding
ASHGA5P054912	5.46488E-07	0.001647562	3.1559354	up	noncoding
ASHGA5P054912	5.46488E-07	0.001647562	3.1559354	up	noncoding
ASHGA5P054912	5.46488E-07	0.001647562	3.1559354	up	noncoding
ASHGA5P055706	0.000288681	0.012492602	3.8466476	up	noncoding
ASHGA5P025952	0.00440548	0.04209439	2.1195039	up	noncoding
ASHGA5P019729	0.027814619	0.104253625	3.8454599	up	noncoding
ASHGA5P047429	0.0371552	0.12233581	2.2516338	up	noncoding
ASHGA5P047429	0.0371552	0.12233581	2.2516338	up	noncoding
ASHGA5P022588	0.000623162	0.016906436	3.8974371	up	noncoding
ASHGA5P053562	0.000557797	0.016097331	3.0426166	up	noncoding
ASHGA5P044594	0.001461681	0.025539378	2.3279752	up	noncoding
ASHGA5P046353	0.000413793	0.014498655	3.0875077	up	noncoding
ASHGA5P006991	5.27433E-05	0.007400226	2.9141016	up	noncoding
ASHGA5P038317	0.002054702	0.029861322	3.7482316	up	noncoding
ASHGA5P022446	0.001268127	0.023779671	2.9983689	up	noncoding
ASHGA5P022446	0.001268127	0.023779671	2.9983689	up	noncoding
ASHGA5P020832	0.007114641	0.053286323	3.280314	up	noncoding
ASHGA5P020832	0.007114641	0.053286323	3.280314	up	noncoding
ASHGA5P044940	0.018639342	0.083001898	2.3522933	up	noncoding
ASHGA5P049045	0.002876416	0.034719588	2.0727459	up	noncoding
ASHGA5P049045	0.002876416	0.034719588	2.0727459	up	noncoding
ASHGA5P049045	0.002876416	0.034719588	2.0727459	up	noncoding
ASHGA5P041021	0.001556104	0.026510436	3.0809507	up	noncoding
ASHGA5P053406	0.000431208	0.014669266	4.3437683	up	noncoding
ASHGA5P026974	0.001230775	0.023565796	3.6980251	up	noncoding
ASHGA5P045405	0.013918895	0.07221887	4.0411521	up	noncoding
ASHGA5P046198	0.002114507	0.030299337	3.3298833	up	noncoding
ASHGA5P046198	0.002114507	0.030299337	3.3298833	up	noncoding
ASHGA5P026668	0.031469451	0.111346021	2.3128638	up	noncoding
ASHGA5P026668	0.031469451	0.111346021	2.3128638	up	noncoding
ASHGA5P026668	0.031469451	0.111346021	2.3128638	up	noncoding
ASHGA5P026668	0.031469451	0.111346021	2.3128638	up	noncoding
ASHGA5P015097	0.015707767	0.076574828	2.370623	up	noncoding
ASHGA5P015768	0.048845665	0.14359352	3.602627	up	noncoding
ASHGA5P045312	0.037561066	0.122993662	2.1085795	up	noncoding
ASHGA5P019889	0.00018175	0.011439647	2.775264	up	noncoding
ASHGA5P032024	0.033719484	0.115849652	2.0050818	up	noncoding
ASHGA5P030748	0.012857912	0.069419086	3.0416767	up	noncoding
ASHGA5P057077	0.009653623	0.060964991	2.0448765	up	noncoding
ASHGA5P037807	0.03527683	0.118945997	3.2878697	up	noncoding
ASHGA5P053428	0.00106613	0.022073729	2.5005624	up	noncoding

NR_045617	SMPX	RefSeq	1053	chrX	-
ENST00000500370	SNHG10	GENCODE	1531	chr14	-
uc009ywt.1	AK313893	UCSC_knowngene	741	chr11	-
ENST00000452037	AC104655.3	GENCODE	515	chr2	+
uc001icl.1	BC043220	UCSC_knowngene	755	chr1	-
ENST00000539233	C11orf95	GENCODE	351	chr11	-
AA151944		LincRNAs identified	518	chr13	+
ENST00000422703	MLK7-AS1	GENCODE	447	chr2	-
ENST00000422703	MLK7-AS1	GENCODE	447	chr2	-
ENST00000573177	RP11-874J12.1	GENCODE	1388	chr18	+
ENST00000573177	RP11-874J12.1	GENCODE	1388	chr18	+
ENST00000573177	RP11-874J12.1	GENCODE	1388	chr18	+
ENST00000573177	RP11-874J12.1	GENCODE	1388	chr18	+
ENST00000573177	RP11-874J12.1	GENCODE	1388	chr18	+
ENST00000573177	RP11-874J12.1	GENCODE	1388	chr18	+
ENST00000573177	RP11-874J12.1	GENCODE	1388	chr18	+
ENST00000573177	RP11-874J12.1	GENCODE	1388	chr18	+
ENST00000537346	DENND5B-AS1	GENCODE	1106	chr12	+
ENST00000418874	AP006748.1	GENCODE	346	chr21	-
chr7:149967450-150	chr7:149967450-150	LincRNAs identified	34300	chr7	-
ENST00000422438	RP11-431J24.2	GENCODE	392	chrX	-
ENST00000537869	SNHG1	GENCODE	1038	chr11	-
ENST00000537869	SNHG1	GENCODE	1038	chr11	-
ENST00000537869	SNHG1	GENCODE	1038	chr11	-
ENST00000567788	RP11-474D1.3	GENCODE	4592	chr12	-
NR_047594	CENPJ	RefSeq	5246	chr13	-
ENST00000502082	RP11-382A18.1	GENCODE	1420	chr8	-
NR_073073	TFAM	RefSeq	5386	chr10	+
NR_073073	TFAM	RefSeq	5386	chr10	+
NR_034039	SORD	RefSeq	2898	chr15	+
uc003ysl.3	PVT1	UCSC_knowngene	1738	chr8	+
ENST00000524499	CTD-3065J16.9	GENCODE	1264	chr8	-
ENST00000435597	RP11-1114A5.4	GENCODE	3018	chrX	-
NR_036659	ZNFX1-AS1	RefSeq	516	chr20	+
ENST00000497543	FAM86DP	GENCODE	2152	chr3	-
ENST00000556874	RP11-72M17.1	GENCODE	1206	chr14	-
ENST00000556874	RP11-72M17.1	GENCODE	1206	chr14	-
NR_036633	VAPB	RefSeq	7764	chr20	+
NR_036633	VAPB	RefSeq	7764	chr20	+
TCONS_00015497	XLOC_007210	LincRNAs identified	327	chr8	-
NR_040252	ANKS3	RefSeq	2667	chr16	-
NR_040252	ANKS3	RefSeq	2667	chr16	-
NR_040252	ANKS3	RefSeq	2667	chr16	-
ENST00000423102	PMCHL1	pseudogene	261	chr5	+
ENST00000442067	GAS5	GENCODE	1114	chr1	-
ENST00000547042	RP4-816N1.7	GENCODE	287	chr12	-
TCONS_00000867	XLOC_000095	LincRNAs identified	263	chr1	+
ENST00000412242	KDM5C-IT1	GENCODE	755	chrX	-
ENST00000412242	KDM5C-IT1	GENCODE	755	chrX	-
ENST00000526730	RP11-7I15.4	GENCODE	277	chr11	+
ENST00000526730	RP11-7I15.4	GENCODE	277	chr11	+
ENST00000526730	RP11-7I15.4	GENCODE	277	chr11	+
ENST00000526730	RP11-7I15.4	GENCODE	277	chr11	+
uc001vgk.2	THSD1P1	UCSC_knowngene	2751	chr13	-
ENST00000423332	CYP4Z2P	pseudogene	1512	chr1	-
ENST00000429700	RP11-82L18.2	GENCODE	585	chr9	-
ENST00000504719	PVT1	GENCODE	754	chr8	+
ENST00000450026	AFG3L1P	GENCODE	494	chr16	+
ENST00000579581	RP11-697E2.7	pseudogene	588	chr15	+
TCONS_00012677	XLOC_005635	LincRNAs identified	335	chr6	-
NR_026919	LOC150197	RefSeq	2206	chr22	+
ENST00000449589	GAS5	GENCODE	712	chr1	-

21724089	21776278		AAATAAGTATCA 23676	exon sense-overlappi
95998633	96001137		CTTATATCCTGAGACAGAACAGTAA	natural antisense
96122322	96123063		CGCCAAAGAGGTTTGTAATCATGAA	exon sense-overlappi
105760580	105761370		CTCTGGGCAGAGGCCACGCGGGGAC	intergenic
247337903	247338870		CTCACATGATCTATTTGAGGTCTTG	intergenic
63531311	63533434	ENST00000433688,	GGGCCTGGGGAGGAGGCTGAAGGA	exon sense-overlappi
20180452	20180928		ACCTGAGGCTGGGCAAGGGGAATAC	intergenic
174031457	174136348		TGCCTGGGAGGT 339751	intronic antisense
174031457	174136348		TGCCTGGGAGGT 339751	intronic antisense
3602997	3604385		AGTTTTAGCAAAAGCAGCAACAAAT	intronic antisense
3602997	3604385		AGTTTTAGCAAAAGCAGCAACAAAT	intronic antisense
3602997	3604385		AGTTTTAGCAAAAGCAGCAACAAAT	intronic antisense
3602997	3604385		AGTTTTAGCAAAAGCAGCAACAAAT	intronic antisense
3602997	3604385		AGTTTTAGCAAAAGCAGCAACAAAT	intronic antisense
3602997	3604385		AGTTTTAGCAAAAGCAGCAACAAAT	intronic antisense
3602997	3604385		AGTTTTAGCAAAAGCAGCAACAAAT	intronic antisense
3602997	3604385		AGTTTTAGCAAAAGCAGCAACAAAT	intronic antisense
31744245	31768600		GAGGGCCTCTTC 100874249	bidirectional
42948061	42953246		TGTCTCATCTGCACCTGGGCACGTG	intergenic
150336517	150370817		ATAATTAAGAATTCAGGAGATGTTT	intergenic
16171063	16175952		CCTCCATCAAATATTCCTGAAAACA	natural antisense
62619462	62622932	ENST00000537068,	GGACTGGCTGTC 23642	bidirectional
62619462	62622932	ENST00000537068,	GGACTGGCTGTC 23642	bidirectional
62619462	62622932	ENST00000537068,	GGACTGGCTGTC 23642	bidirectional
130517998	130529602		TTCCCTGTATCTAAATTTTAGCAAGT	intergenic
25456411	25497027		ACATCCAGCTCT 55835	exon sense-overlappi
128302061	128494384		TTAAAAAAAGCTTTGTGCTGATGAA	intronic antisense
60144902	60158990		TTTTTCTGGCTCA 7019	exon sense-overlappi
60144902	60158990		TTTTTCTGGCTCA 7019	exon sense-overlappi
45315301	45367287		TGGGATCATCGG 6652	exon sense-overlappi
128902834	129113499	ENST00000504719,	AAAGATGCCCCT 5820	intronic antisense
145132904	145134168		GTATGTCTGTTTATCTCTTGCCACTT	natural antisense
135923089	135929956		CAGTGCTGAGAAATATGAGGTTCTG	intergenic
47895178	47905795	ENST00000428008	TTTGTGTGTGGTT 441951	bidirectional
75470702	75484199	ENST00000491583	GGGGCAGACGCG 692099	intergenic
66697050	66976112		TCTCGTCCAGTGTGACATTCTTCAG	intronic antisense
66697050	66976112		TCTCGTCCAGTGTGACATTCTTCAG	intronic antisense
56964174	57026156		GGAATCATCGAT 9217	exon sense-overlappi
56964174	57026156		GGAATCATCGAT 9217	exon sense-overlappi
127630407	127634583		TTTTAGAGCTGGGATGGATTTTCCA	intergenic
4746510	4784378		TAACGCACGTTCT 124401	exon sense-overlappi
4746510	4784378		TAACGCACGTTCT 124401	exon sense-overlappi
4746510	4784378		TAACGCACGTTCT 124401	natural antisense
22142481	22143012	ENST00000415808,	GAGAAACTGGGG 5369	
173833768	173836181	ENST00000416952,	TGAAACAGTAGT 60674	intergenic
2906301	2913104		AAGGAGAATTTGTCCTTGCGATCCA	natural antisense
24348318	24350190		ATAAAAGTTGATGAAGCTGTAGCAC	intergenic
53241589	53243861		GGCTAAATTGTGTGTTAGAAACTCT	intron sense-overlapp
53241589	53243861		GGCTAAATTGTGTGTTAGAAACTCT	intron sense-overlapp
77733978	77734767		CGAAAGGAGAGTGATCCCAGCAGG	intronic antisense
77733978	77734767		CGAAAGGAGAGTGATCCCAGCAGG	intronic antisense
77733978	77734767		CGAAAGGAGAGTGATCCCAGCAGG	intronic antisense
77733978	77734767		CGAAAGGAGAGTGATCCCAGCAGG	intronic antisense
52741846	52768575	ENST00000416599,	GAGCTGACTGAA 374500	intergenic
47308849	47366106		CTATGCCTTCATA 163720	
91703801	91724849		AGCTCTCAGGTCACTTCTGGCTTCC	intron sense-overlapp
128806778	128903221	ENST00000522963,	ACCCCTAAGGGC 5820	intergenic
90038996	90044363	ENST00000423742	TTAACAGCATGA 172	natural antisense
90818360	90818948		TGGTAATTTACCCCGTGAAGTACACTCAACCTTCAACC	
20331467	20332657		TAATAGAGCTATTCCTCACTCTCCC	intergenic
20193854	20196060		GGACATTTATCAC 150197	exon sense-overlappi
173833153	173837127	ENST00000416952,	CCATTGGATGTG 60674	bidirectional

NM_014332	SMPX	small muscular prote	-	21724089	21776278
ENST00000331334	GLRX5	glutaredoxin 5 [Sour	+	96000929	96011055
NM_024725	CCDC82	coiled-coil domain-c	-	96085928	96123083
NM_001144936	C11orf95	uncharacterized prote	-	63527363	63536113
NM_016653	ZAK	mitogen-activated pro	+	173940564	174132737
NM_133646	ZAK	mitogen-activated pro	+	173940564	174091873
NM_001003809	DLGAP1	disks large-associate	-	3496029	3845358
NM_001242761	DLGAP1	disks large-associate	-	3502142	4455266
NM_001242762	DLGAP1	disks large-associate	-	3496029	3874767
NM_001242763	DLGAP1	disks large-associate	-	3496029	3874767
NM_001242764	DLGAP1	disks large-associate	-	3496029	3874253
NM_001242765	DLGAP1	disks large-associate	-	3502142	3845358
NM_001242766	DLGAP1	disks large-associate	-	3496029	3845358
NM_004746	DLGAP1	disks large-associate	-	3496029	4455266
NM_144973	DENND5B	DENN domain-conta	-	31535156	31743952
NM_005314	GRPR	gastrin-releasing pep	+	16141423	16171641
NM_001012662	SLC3A2	4F2 cell-surface anti	+	62623483	62656355
NM_001012664	SLC3A2	4F2 cell-surface anti	+	62623483	62656355
NM_002394	SLC3A2	4F2 cell-surface anti	+	62623483	62656355
NM_018451	CENPJ	centromere protein J	-	25456411	25497027
NM_001159542	POU5F1B	putative POU domai	+	128427856	128429441
NM_001270782	TFAM	transcription factor A	+	60144902	60158990
NM_003201	TFAM	transcription factor A	+	60144902	60158990
NM_003104	SORD	sorbitol dehydrogena	+	45315301	45367287
ENST00000539634	TMEM75	transmembrane prote	-	128959125	128960591
NM_019037	EXOSC4	exosome complex co	+	145133521	145135551
NM_021035	ZNFX1	NFX1-type zinc fing	-	47862438	47894756
NM_001024218	GPHN	gephyrin isoform 2	+	66974124	67648525
NM_020806	GPHN	gephyrin isoform 1	+	66974124	67648525
NM_001195677	VAPB	vesicle-associated me	+	56964174	57026156
NM_004738	VAPB	vesicle-associated me	+	56964174	57026156
NM_001242929	ANKS3	ankyrin repeat and S	-	4746510	4784378
NM_133450	ANKS3	ankyrin repeat and S	-	4746510	4784163
NM_139170	C16orf71	uncharacterized prote	+	4784288	4799397
NM_002014	FKBP4	peptidyl-prolyl cis-tr	+	2904107	2914587
NM_001146702	KDM5C	lysine-specific demet	-	53220502	53254604
NM_004187	KDM5C	lysine-specific demet	-	53220502	53254604
NM_001203260	NDUFC2-KCTD14	NDUFC2-KCTD14 p	-	77726760	77791265
NM_001203261	NDUFC2-KCTD14	NDUFC2-KCTD14 p	-	77726760	77791265
NM_001203262	NDUFC2-KCTD14	NDUFC2-KCTD14 p	-	77726760	77791265
NM_023930	KCTD14	BTB/POZ domain-co	-	77726760	77734320
NM_016848	SHC3	SHC-transforming pr	-	91620685	91793682
NM_145039	CENPBD1	CENPB DNA-bindin	-	90036182	90039240
TTCATGCCAAGCCCCGCTGTCA					
ENST00000423736	AC007663.1	Em:AC006547.7 pro	+	20194099	20194706
NM_001122770	ZBTB37	zinc finger and BTB	+	173837492	173855774

193.50021	5.497516	6.142829	2.585559	375.07416	38.02748
61.789583	19.305665	5.562189	4.506525	64.46585	83.22551
50.109423	18.398614	5.218266	3.913267	51.55842	26.24899
46.395022	15.125728	5.185904	4.145857	54.521076	51.19112
38.277186	10.58105	4.672685	3.425993	26.068085	16.59449
413.52509	91.198744	8.284008	6.820431	431.67288	557.85815
4533.412417	329.692318	11.506861	8.681087	3964.2446	4976.288
48.223051	10.167999	4.856377	3.462891	23.209707	19.124321
48.223051	10.167999	4.856377	3.462891	23.209707	19.124321
81.420695	23.065923	5.896903	4.69693	87.37382	55.524643
81.420695	23.065923	5.896903	4.69693	87.37382	55.524643
81.420695	23.065923	5.896903	4.69693	87.37382	55.524643
81.420695	23.065923	5.896903	4.69693	87.37382	55.524643
81.420695	23.065923	5.896903	4.69693	87.37382	55.524643
81.420695	23.065923	5.896903	4.69693	87.37382	55.524643
81.420695	23.065923	5.896903	4.69693	87.37382	55.524643
81.420695	23.065923	5.896903	4.69693	87.37382	55.524643
210.84899	50.462572	7.211775	5.877614	309.6198	299.76157
26.950785	7.639889	4.404308	2.946319	31.11921	17.990705
908.6792	211.520507	9.4108	7.795857	1148.8248	1084.12
649.905942	5.071188	7.046042	2.412862	1247.1539	41.455643
35295.024	6514.60715	14.674772	13.016704	38133.566	26421.146
35295.024	6514.60715	14.674772	13.016704	38133.566	26421.146
35295.024	6514.60715	14.674772	13.016704	38133.566	26421.146
401.197882	54.53392	7.850373	5.906771	638.0737	1002.6972
58.127653	19.227146	5.462318	4.378591	47.46198	36.300156
61.504814	9.486939	5.212792	3.269636	101.07288	97.10215
23.294835	8.622724	4.146599	2.975627	28.068836	38.785828
23.294835	8.622724	4.146599	2.975627	28.068836	38.785828
195.623483	31.527309	7.177672	5.215147	205.92928	324.00504
232.127705	44.793273	7.378391	5.773078	186.2318	154.46869
1469.490028	374.561695	10.067473	8.848397	1290.4369	832.20667
510.548848	97.076997	8.572263	6.94582	457.76242	336.869
3296.466167	611.482657	11.164933	9.621882	3909.2944	5344.067
436.086925	61.933528	8.156973	6.250763	627.0881	960.65466
239.530098	45.480465	7.363177	5.779	318.26108	447.5427
239.530098	45.480465	7.363177	5.779	318.26108	447.5427
58.680176	13.019402	5.472931	3.759097	76.610794	89.37592
58.680176	13.019402	5.472931	3.759097	76.610794	89.37592
50.427592	12.824626	5.09409	3.860022	65.53986	107.36382
165.79517	52.956429	7.016241	5.964698	172.01569	192.74422
165.79517	52.956429	7.016241	5.964698	172.01569	192.74422
165.79517	52.956429	7.016241	5.964698	172.01569	192.74422
34.922679	7.717003	4.637247	3.013872	19.984411	41.80718
1065.8027	148.136635	9.629541	7.510594	1128.0826	1196.3378
46.222071	8.490223	5.153827	3.267072	52.96162	68.09419
228.322218	25.976891	6.964549	4.949782	362.5893	581.42865
242.116718	46.684717	7.310424	5.574953	270.23535	368.70798
242.116718	46.684717	7.310424	5.574953	270.23535	368.70798
73.773163	17.884516	5.607278	4.397598	105.037094	73.90427
73.773163	17.884516	5.607278	4.397598	105.037094	73.90427
73.773163	17.884516	5.607278	4.397598	105.037094	73.90427
73.773163	17.884516	5.607278	4.397598	105.037094	73.90427
231.637077	61.528602	7.407647	6.162381	191.74554	114.5608
66.769513	12.382673	5.576433	3.727384	52.52818	19.946407
58.980414	16.100954	5.353325	4.277054	107.284615	84.50338
80.997372	17.902226	5.909396	4.436771	65.5435	51.824738
59.405074	23.273972	5.545419	4.541758	65.41221	60.50814
1475.970417	335.637843	9.963951	8.359084	1307.4318	431.3474
315.971278	105.475909	7.917946	6.885932	397.78214	386.79968
134.27164	35.802259	6.64363	4.926477	94.10796	75.68579
825.74361	203.031225	9.278482	7.956229	801.2823	829.60156

628.30304	51.90251	22.127108	45.566963	4.9999995	7.9851007
46.57303	42.373077	67.09458	67.00545	17.581356	12.358489
78.47563	30.323256	52.797836	61.252407	25.080442	8.090246
56.626587	40.794445	34.450146	40.78676	10.580701	12.896919
21.43933	90.429504	19.168657	55.96305	10.50451	4.9999995
287.7151	370.7171	360.72073	472.46658	73.78375	87.023155
2928.9592	1293.5687	7656.922	6380.492	249.97137	264.6478
18.790335	28.807175	112.62296	86.783806	13.11815	4.9999995
18.790335	28.807175	112.62296	86.783806	13.11815	4.9999995
102.247375	35.650723	102.1504	105.57721	18.54983	12.109049
102.247375	35.650723	102.1504	105.57721	18.54983	12.109049
102.247375	35.650723	102.1504	105.57721	18.54983	12.109049
102.247375	35.650723	102.1504	105.57721	18.54983	12.109049
102.247375	35.650723	102.1504	105.57721	18.54983	12.109049
102.247375	35.650723	102.1504	105.57721	18.54983	12.109049
102.247375	35.650723	102.1504	105.57721	18.54983	12.109049
102.247375	35.650723	102.1504	105.57721	18.54983	12.109049
274.94254	86.89063	158.53113	135.34827	63.927128	61.069927
38.947624	29.011154	19.797714	24.838305	4.9999995	4.9999995
705.2297	654.9577	960.8973	898.0457	183.8524	68.40395
2433.9512	35.83055	69.88887	71.15549	5.427133	4.9999995
35182.41	32780.11	38222.402	41030.51	6768.6494	3233.3318
35182.41	32780.11	38222.402	41030.51	6768.6494	3233.3318
35182.41	32780.11	38222.402	41030.51	6768.6494	3233.3318
165.65045	118.865524	232.32968	249.57074	73.52752	113.111206
51.930447	47.504604	86.11431	79.45442	14.72946	6.0665293
105.05042	20.240002	21.866482	23.696949	8.366385	9.458083
21.417742	16.471212	17.40726	17.618132	10.77374	4.9999995
21.417742	16.471212	17.40726	17.618132	10.77374	4.9999995
118.44699	138.11862	177.81229	209.42868	29.490196	21.27087
189.45558	458.83124	134.95522	268.8237	37.758896	32.919254
1583.02	2022.2769	1190.028	1898.9717	312.99838	199.52718
604.4068	678.0396	389.13397	597.0813	97.76855	84.60902
1852.2235	3595.9915	2009.3009	3067.9197	564.0029	672.2401
284.86203	211.90894	265.43076	266.57706	59.82941	64.865425
158.6369	249.5596	89.643906	173.5364	39.437	59.09107
158.6369	249.5596	89.643906	173.5364	39.437	59.09107
55.610413	49.21062	37.355457	43.917854	14.45565	6.3851333
55.610413	49.21062	37.355457	43.917854	14.45565	6.3851333
23.806942	24.668499	42.42699	38.75944	13.966959	9.60096
147.07736	145.52489	154.78131	182.62755	44.043987	28.284166
147.07736	145.52489	154.78131	182.62755	44.043987	28.284166
147.07736	145.52489	154.78131	182.62755	44.043987	28.284166
13.280659	34.16982	46.301426	53.99258	5.401293	4.9999995
1024.0413	1251.6981	646.6386	1148.0178	118.57551	90.4957
31.010374	41.60133	41.526787	42.138123	12.297108	5.375254
97.402985	172.60867	39.572296	116.331406	25.741201	28.068846
127.88959	61.856686	341.8901	282.1206	55.112892	32.5592
127.88959	61.856686	341.8901	282.1206	55.112892	32.5592
132.72073	18.738182	51.431194	60.807507	17.212166	10.71681
132.72073	18.738182	51.431194	60.807507	17.212166	10.71681
132.72073	18.738182	51.431194	60.807507	17.212166	10.71681
132.72073	18.738182	51.431194	60.807507	17.212166	10.71681
270.6839	354.42593	167.72984	290.67645	64.762405	38.095966
76.52652	61.311428	91.80653	98.49801	16.067297	20.87161
48.97654	21.761593	46.23188	45.124474	16.065222	9.943064
68.784546	146.81067	51.97729	101.04349	16.866161	12.470498
55.48839	71.68754	42.053974	61.28019	17.011951	7.4844155
2279.19	2061.681	1090.1465	1686.0258	361.87622	79.18389
267.80173	261.9244	290.05515	291.46457	105.481186	52.323505
118.3938	218.78432	126.51539	172.14258	50.65581	9.061884
761.5928	1073.6698	571.1064	917.2088	179.47456	85.19255

4.9999995	4.9999995	4.9999995	4.9999995	8.086212	5.150998
30.256271	15.122211	22.961292	17.554373	5.5516243	6.1831684
52.209957	5.8535695	14.15747	4.9999995	5.222305	4.663017
12.802802	28.826996	12.828519	12.81843	5.3016605	5.5434933
14.059541	20.168295	6.1027164	7.651237	4.246353	4.0633783
58.599426	133.23413	102.472084	92.07992	8.291409	8.813948
207.92429	354.50818	509.00067	392.1016	11.455366	11.8192625
12.44121	10.95663	10.562269	8.929734	4.0732503	4.243498
12.44121	10.95663	10.562269	8.929734	4.0732503	4.243498
14.509579	21.682926	46.601578	24.942577	5.99547	5.6437745
14.509579	21.682926	46.601578	24.942577	5.99547	5.6437745
14.509579	21.682926	46.601578	24.942577	5.99547	5.6437745
14.509579	21.682926	46.601578	24.942577	5.99547	5.6437745
14.509579	21.682926	46.601578	24.942577	5.99547	5.6437745
14.509579	21.682926	46.601578	24.942577	5.99547	5.6437745
14.509579	21.682926	46.601578	24.942577	5.99547	5.6437745
14.509579	21.682926	46.601578	24.942577	5.99547	5.6437745
77.56689	41.369778	31.538446	27.30326	7.8185415	7.9555635
5.543768	4.9999995	14.118321	11.177248	4.502668	4.1658506
542.2288	149.40291	166.1676	159.06738	9.69575	9.733742
4.9999995	4.9999995	4.9999995	4.9999995	9.810356	5.263874
9989.955	5463.639	7792.0737	5839.994	14.810216	14.296818
9989.955	5463.639	7792.0737	5839.994	14.810216	14.296818
9989.955	5463.639	7792.0737	5839.994	14.810216	14.296818
36.20144	41.347225	36.810307	26.20582	8.844283	9.623875
33.151485	20.755278	24.912983	15.74714	5.111353	5.0903254
4.9999995	4.9999995	23.167637	5.929528	6.2100544	6.3850203
20.762564	4.9999995	4.9999995	5.2000413	4.3573346	5.177353
20.762564	4.9999995	4.9999995	5.2000413	4.3573346	5.177353
49.6806	20.986607	40.721214	27.014364	7.232936	8.059328
30.629087	72.17086	49.191887	46.089653	7.086796	7.0354357
477.91806	322.53397	594.49854	339.89404	9.867495	9.370539
100.38469	109.71939	99.35092	90.62941	8.362253	8.1130085
479.2018	830.1682	535.64844	587.6345	11.436981	11.929546
41.167824	68.72714	90.275345	46.736023	8.818264	9.5666065
26.837868	71.55732	32.10244	43.85709	7.8591027	8.495712
26.837868	71.55732	32.10244	43.85709	7.8591027	8.495712
25.36345	4.9999995	15.959638	10.95254	5.8045845	6.2767053
25.36345	4.9999995	15.959638	10.95254	5.8045845	6.2767053
22.323439	6.6169143	14.141722	10.297759	5.573363	6.528861
65.49032	38.476227	85.20919	56.234684	6.9740424	7.3472104
65.49032	38.476227	85.20919	56.234684	6.9740424	7.3472104
65.49032	38.476227	85.20919	56.234684	6.9740424	7.3472104
4.9999995	8.121324	12.818281	9.961119	3.861624	5.275325
161.3518	132.74504	228.66548	156.98628	9.664968	9.866973
13.985979	7.2753615	5.8389206	6.168716	5.258837	5.9166517
37.12191	15.0019	29.2459	20.681587	8.039865	8.87384
79.73476	11.29239	72.57795	28.831112	7.6201086	8.231701
79.73476	11.29239	72.57795	28.831112	7.6201086	8.231701
21.480764	15.030591	27.339083	15.52768	6.269996	6.024085
21.480764	15.030591	27.339083	15.52768	6.269996	6.024085
21.480764	15.030591	27.339083	15.52768	6.269996	6.024085
21.480764	15.030591	27.339083	15.52768	6.269996	6.024085
98.073784	36.101154	84.93642	47.20188	7.1320114	6.6186795
5.3371377	16.05418	4.9999995	10.965811	5.247462	4.2966757
18.557201	18.522451	18.620619	14.897168	6.3002176	6.2051883
14.663639	25.500809	16.02292	21.889326	5.5734725	5.55934
58.20099	19.956444	22.55059	14.439439	5.570228	5.756935
697.57666	104.72119	512.0775	258.3916	9.886148	8.445166
235.47246	89.02538	81.00886	69.54406	8.180854	8.303292
97.49439	10.86684	29.630056	17.104572	6.1090384	6.053564
249.72124	243.39584	249.6204	210.78276	9.185014	9.365079

8.928796	5.3031244	4.362803	5.0250416	2.3467326	3.7604706
5.2218676	4.991579	5.8678384	5.5570536	4.3732934	4.422317
5.978817	4.4731064	5.5403647	5.431983	4.8916016	3.7830913
5.5088496	4.9320064	4.9554305	4.873985	3.629726	4.486372
4.1049676	6.1459613	4.1632395	5.312208	3.6199841	2.4279919
7.83588	8.213706	8.237763	8.311342	6.4335265	7.295902
11.139539	10.019149	12.584982	12.022866	8.223052	8.910387
3.9139345	4.3887844	6.5992675	5.919529	3.9402096	2.4279919
3.9139345	4.3887844	6.5992675	5.919529	3.9402096	2.4279919
6.3596253	4.720988	6.459258	6.202302	4.4477262	4.392129
6.3596253	4.720988	6.459258	6.202302	4.4477262	4.392129
6.3596253	4.720988	6.459258	6.202302	4.4477262	4.392129
6.3596253	4.720988	6.459258	6.202302	4.4477262	4.392129
6.3596253	4.720988	6.459258	6.202302	4.4477262	4.392129
6.3596253	4.720988	6.459258	6.202302	4.4477262	4.392129
6.3596253	4.720988	6.459258	6.202302	4.4477262	4.392129
6.3596253	4.720988	6.459258	6.202302	4.4477262	4.392129
7.766587	6.0869474	7.0874557	6.555554	6.2325907	6.7872677
4.9681315	4.399888	4.2074895	4.181823	2.3467326	2.4279919
9.108781	9.042444	9.6568985	9.227187	7.7709255	6.9471784
10.905268	4.7279334	5.9258494	5.64297	2.6430278	2.4279919
14.802069	14.694532	14.810216	14.634781	13.028245	12.568294
14.802069	14.694532	14.810216	14.634781	13.028245	12.568294
14.802069	14.694532	14.810216	14.634781	13.028245	12.568294
7.0398436	6.5606337	7.6142225	7.419379	6.428829	7.671328
5.3796864	5.170342	6.223396	5.798806	4.1118026	3.3502028
6.398073	3.819745	4.3463387	4.1175203	3.2710438	4.017671
4.102395	3.4909449	4.0361457	3.7154195	3.654105	2.4279919
4.102395	3.4909449	4.0361457	3.7154195	3.654105	2.4279919
6.5696297	6.784023	7.2464523	7.1736655	5.1134753	5.2426343
7.2440968	8.527583	6.8536906	7.5227437	5.4746904	5.8790426
10.26295	10.647209	9.953668	10.302975	8.559115	8.499752
8.870395	9.097352	8.352535	8.638033	6.843725	7.253504
10.497016	11.467794	10.681693	10.976569	9.414182	10.2493
7.8198905	7.415657	7.8095045	7.511914	6.140405	6.874795
6.97556	7.6539993	6.2779746	6.916716	5.5354834	6.735208
6.97556	7.6539993	6.2779746	6.916716	5.5354834	6.735208
5.4820085	5.228952	5.071197	4.97414	4.0826287	3.4278843
5.4820085	5.228952	5.071197	4.97414	4.0826287	3.4278843
4.26493	4.142945	5.2490444	4.805395	4.029748	4.0417104
6.874795	6.858004	7.057122	6.9862714	5.6937175	5.6589184
6.874795	6.858004	7.057122	6.9862714	5.6937175	5.6589184
6.874795	6.858004	7.057122	6.9862714	5.6937175	5.6589184
3.4040575	4.6556425	5.364009	5.2628264	2.6362064	2.4279919
9.625287	9.971427	9.070833	9.57776	7.126667	7.349266
4.647783	4.963566	5.2184324	4.917691	3.8497374	3.159215
6.2840014	7.10376	5.147186	6.3386407	4.9297	5.6483765
6.67738	5.5751166	8.162547	7.5956917	6.0210204	5.864086
6.67738	5.5751166	8.162547	7.5956917	6.0210204	5.864086
6.727361	3.694447	5.506139	5.4216394	4.3429213	4.211325
6.727361	3.694447	5.506139	5.4216394	4.3429213	4.211325
6.727361	3.694447	5.506139	5.4216394	4.3429213	4.211325
6.727361	3.694447	5.506139	5.4216394	4.3429213	4.211325
7.747083	8.145644	7.1652217	7.637244	6.252406	6.093305
5.942403	5.561391	6.3092628	6.1014056	4.23793	5.216565
5.2987275	3.9411843	5.3619757	5.012658	4.2375298	4.0952086
5.794189	6.8703704	5.5208178	6.1381874	4.3094945	4.4379225
5.478944	5.8002954	5.2337437	5.432369	4.3245263	3.6656382
10.810523	10.673766	9.840263	10.12784	8.762093	7.157763
7.729767	7.7181187	7.9366636	7.6389804	6.959223	6.5582266
6.568569	7.462455	6.762277	6.9058785	5.8996315	3.9552395
9.222115	9.747093	8.899571	9.252018	7.7407985	7.264973

2.350709	2.3408759	2.3618152	2.352752
4.934734	4.05844	4.7940626	4.4563026
5.6973753	2.6602614	4.0945215	2.352752
3.759234	5.057914	3.9569266	3.9849677
3.8839915	4.4939203	2.927049	3.203024
5.8593426	7.423303	6.934781	6.9757285
7.651756	8.924494	9.224349	9.152482
3.721424	3.564649	3.6832945	3.439778
3.721424	3.564649	3.6832945	3.439778
3.9250717	4.606222	5.812874	4.99756
3.9250717	4.606222	5.812874	4.99756
3.9250717	4.606222	5.812874	4.99756
3.9250717	4.606222	5.812874	4.99756
3.9250717	4.606222	5.812874	4.99756
3.9250717	4.606222	5.812874	4.99756
3.9250717	4.606222	5.812874	4.99756
3.9250717	4.606222	5.812874	4.99756
6.243641	5.6087933	5.2541614	5.139229
2.6942596	2.3408759	4.0907717	3.777281
9.033111	7.596813	7.6121087	7.8150063
2.350709	2.3408759	2.3618152	2.352752
13.24231	12.895115	13.24231	13.123952
13.24231	12.895115	13.24231	13.123952
13.24231	12.895115	13.24231	13.123952
5.181766	5.6084337	5.474874	5.075396
5.062258	4.539161	4.910662	4.2974625
2.350709	2.3408759	4.807745	2.82977
4.414891	2.3408759	2.3618152	2.6540806
4.414891	2.3408759	2.3618152	2.6540806
5.632655	4.5590525	5.620453	5.12261
4.9511337	6.4932923	5.891892	5.9484196
8.853938	8.779891	9.450233	8.947453
6.6120257	7.1223617	6.890214	6.9530897
8.859375	10.16732	9.294022	9.747093
5.361157	6.411529	6.747841	5.968849
4.768921	6.4801035	5.2785006	5.8757806
4.768921	6.4801035	5.2785006	5.8757806
4.687648	2.3408759	4.2696924	3.7458546
4.687648	2.3408759	4.2696924	3.7458546
4.5132084	2.8253298	4.092942	3.657192
6.013214	5.501737	6.6669846	6.253614
6.013214	5.501737	6.6669846	6.253614
6.013214	5.501737	6.6669846	6.253614
2.350709	3.1107988	3.9557266	3.601798
7.283399	7.417538	8.093078	7.793617
3.8780541	2.9558587	2.8746185	2.8849473
5.216419	4.044402	5.1485267	4.711269
6.282127	3.6078851	6.44596	5.2286367
6.282127	3.6078851	6.44596	5.2286367
4.460197	4.0479074	5.045829	4.2774057
4.460197	4.0479074	5.045829	4.2774057
4.460197	4.0479074	5.045829	4.2774057
4.460197	4.0479074	5.045829	4.2774057
6.5756803	5.4061484	6.6632814	5.983465
2.6534348	4.1469755	2.3618152	3.7475839
4.265582	4.3616095	4.486859	4.215534
3.94055	4.862039	4.275234	4.795387
5.849493	4.47856	4.767035	4.165296
9.392478	7.058901	9.231916	8.551354
7.8266177	6.8089333	6.595763	6.5668297
6.5673504	3.5495846	5.1675377	4.4195194
7.9117007	8.349012	8.221882	8.249008

ASHGA5P053428	0.00106613	0.022073729	2.5005624	up	noncoding
ASHGA5P028909	0.008164325	0.056774037	2.5653112	up	noncoding
ASHGA5P017314	7.4385E-05	0.008283845	4.2673309	up	noncoding
ASHGA5P017314	7.4385E-05	0.008283845	4.2673309	up	noncoding
ASHGA5P017314	7.4385E-05	0.008283845	4.2673309	up	noncoding
ASHGA5P017314	7.4385E-05	0.008283845	4.2673309	up	noncoding
ASHGA5P037205	0.00646773	0.051079512	2.0038158	up	noncoding
ASHGA5P044291	0.000760308	0.018587813	3.0324053	up	noncoding
ASHGA5P043947	0.028692464	0.105667631	2.3361488	up	noncoding
ASHGA5P044812	0.000649252	0.017104871	2.2750788	up	noncoding
ASHGA5P023027	0.003396129	0.037609468	2.263611	up	noncoding
ASHGA5P023027	0.003396129	0.037609468	2.263611	up	noncoding
ASHGA5P043168	0.043713437	0.134107741	2.4237495	up	noncoding
ASHGA5P032708	0.000214693	0.01146874	4.8906477	up	noncoding
ASHGA5P048186	1.09802E-05	0.004010044	2.580733	up	noncoding
ASHGA5P048186	1.09802E-05	0.004010044	2.580733	up	noncoding
ASHGA5P048186	1.09802E-05	0.004010044	2.580733	up	noncoding
ASHGA5P018975	0.009806122	0.061453355	2.7206794	up	noncoding
ASHGA5P001407	0.000205649	0.01146874	4.1006512	up	noncoding
ASHGA5P001407	0.000205649	0.01146874	4.1006512	up	noncoding
ASHGA5P001407	0.000205649	0.01146874	4.1006512	up	noncoding
ASHGA5P001407	0.000205649	0.01146874	4.1006512	up	noncoding
ASHGA5P001407	0.000205649	0.01146874	4.1006512	up	noncoding
ASHGA5P047569	0.010279032	0.062647807	2.3350852	up	noncoding
ASHGA5P050994	0.002245065	0.031214119	3.0277744	up	noncoding
ASHGA5P056711	0.005723094	0.048087879	3.7674974	up	noncoding
ASHGA5P028830	3.87651E-06	0.00279577	4.2629855	up	noncoding
ASHGA5P043957	0.00476573	0.043854347	2.0343845	up	noncoding
ASHGA5P018335	0.014165152	0.073080363	2.1123849	up	noncoding
ASHGA5P047146	0.02063656	0.088218874	2.4173759	up	noncoding
ASHGA5P047146	0.02063656	0.088218874	2.4173759	up	noncoding
ASHGA5P019438	0.021010287	0.089265357	2.0465866	up	noncoding
ASHGA5P019438	0.021010287	0.089265357	2.0465866	up	noncoding
ASHGA5P019438	0.021010287	0.089265357	2.0465866	up	noncoding
ASHGA5P019438	0.021010287	0.089265357	2.0465866	up	noncoding
ASHGA5P031667	0.006856277	0.052487971	2.3190393	up	noncoding
ASHGA5P031667	0.006856277	0.052487971	2.3190393	up	noncoding
ASHGA5P031667	0.006856277	0.052487971	2.3190393	up	noncoding
ASHGA5P035229	0.01953095	0.085195149	3.4446936	up	noncoding
ASHGA5P037934	0.003457169	0.037897493	2.0521959	up	noncoding
ASHGA5P008083	0.020704802	0.088426356	2.7967891	up	noncoding
ASHGA5P008083	0.020704802	0.088426356	2.7967891	up	noncoding
ASHGA5P008083	0.020704802	0.088426356	2.7967891	up	noncoding
ASHGA5P008083	0.020704802	0.088426356	2.7967891	up	noncoding
ASHGA5P035924	0.047887063	0.141761103	2.1330735	up	noncoding
ASHGA5P035924	0.047887063	0.141761103	2.1330735	up	noncoding
ASHGA5P035924	0.047887063	0.141761103	2.1330735	up	noncoding
ASHGA5P035924	0.047887063	0.141761103	2.1330735	up	noncoding
ASHGA5P033946	0.004958278	0.044652877	3.450651	up	noncoding
ASHGA5P046096	5.71112E-07	0.001647562	4.4274091	up	noncoding
ASHGA5P053422	0.000721097	0.018055047	5.1603983	up	noncoding
ASHGA5P053422	0.000721097	0.018055047	5.1603983	up	noncoding
ASHGA5P047956	0.004259838	0.041423336	3.6169862	up	noncoding
ASHGA5P048191	0.030603896	0.109741627	8.9504249	up	noncoding
ASHGA5P046995	0.000511648	0.015567285	2.260796	up	noncoding
ASHGA5P028991	0.003726844	0.039190735	2.3083878	up	noncoding
ASHGA5P028991	0.003726844	0.039190735	2.3083878	up	noncoding
ASHGA5P028991	0.003726844	0.039190735	2.3083878	up	noncoding
ASHGA5P030271	0.019926546	0.08624871	2.0537034	up	noncoding
ASHGA5P026905	0.02833334	0.105047356	2.9864757	up	noncoding
ASHGA5P041839	0.0022536	0.031214119	3.0884072	up	noncoding
ASHGA5P020689	0.000448271	0.014950157	3.1609469	up	noncoding

ENST00000449589	GAS5	GENCODE	712	chr1	-
ENST00000562781	RP11-122A8.4	GENCODE	807	chr13	+
NR_037669	GGCT	RefSeq	1051	chr7	-
NR_037669	GGCT	RefSeq	1051	chr7	-
NR_037669	GGCT	RefSeq	1051	chr7	-
NR_037669	GGCT	RefSeq	1051	chr7	-
ENST00000416641	AP000432.2	GENCODE	516	chr21	-
ENST00000534670	RP11-33I11.2	GENCODE	301	chr8	-
uc022amu.1	BC065735	UCSC_knowngene	1437	chr7	+
ENST00000521612	RP11-110G21.1	GENCODE	423	chr8	+
NR_045815	ATF7IP2	RefSeq	3451	chr16	+
NR_045815	ATF7IP2	RefSeq	3451	chr16	+
TCONS_00018358	XLOC_008643	LincRNAs identified	413	chr10	+
TCONS_00029193	XLOC_013852	LincRNAs identified	557	chr21	+
uc001nvs.3	SNHG1	UCSC_knowngene	1365	chr11	-
uc001nvs.3	SNHG1	UCSC_knowngene	1365	chr11	-
uc001nvs.3	SNHG1	UCSC_knowngene	1365	chr11	-
NR_051969	MRPS2	RefSeq	1719	chr9	+
NR_072998	PTK7	RefSeq	4040	chr6	+
NR_072998	PTK7	RefSeq	4040	chr6	+
NR_072998	PTK7	RefSeq	4040	chr6	+
NR_072998	PTK7	RefSeq	4040	chr6	+
NR_072998	PTK7	RefSeq	4040	chr6	+
TCONS_00014180	XLOC_006188	LincRNAs identified	313	chr7	+
NR_038337	LOC100505783	RefSeq	1743	chr20	+
TCONS_00007988	XLOC_003455	LincRNAs identified	372	chr4	+
NR_046551	DLEU7-AS1	RefSeq	1824	chr13	+
TCONS_00014730	XLOC_006818	LincRNAs identified	278	chr8	+
ENST00000452249	AC003075.4	GENCODE	441	chr7	-
NR_033847	FLJ37035	RefSeq	2527	chr10	-
NR_033847	FLJ37035	RefSeq	2527	chr10	-
NR_046443	CEP41	RefSeq	6410	chr7	-
NR_046443	CEP41	RefSeq	6410	chr7	-
NR_046443	CEP41	RefSeq	6410	chr7	-
NR_046443	CEP41	RefSeq	6410	chr7	-
uc002dyu.3	ZNF785	UCSC_knowngene	1475	chr16	+
uc002dyu.3	ZNF785	UCSC_knowngene	1475	chr16	+
uc002dyu.3	ZNF785	UCSC_knowngene	1475	chr16	+
NR_046111	LOC440894	RefSeq	2334	chr2	-
ENST00000441558	MORC2-AS1	GENCODE	425	chr22	+
NR_049767	SLC7A8	RefSeq	3236	chr14	-
NR_049767	SLC7A8	RefSeq	3236	chr14	-
NR_049767	SLC7A8	RefSeq	3236	chr14	-
NR_049767	SLC7A8	RefSeq	3236	chr14	-
ENST00000434786	ZNF638-IT1	GENCODE	827	chr2	+
ENST00000434786	ZNF638-IT1	GENCODE	827	chr2	+
ENST00000434786	ZNF638-IT1	GENCODE	827	chr2	+
ENST00000434786	ZNF638-IT1	GENCODE	827	chr2	+
TCONS_00004010	XLOC_001836	LincRNAs identified	347	chr2	+
uc011mhl.1	AK097803	UCSC_knowngene	1681	chrX	-
ENST00000458220	GAS5	GENCODE	469	chr1	-
ENST00000458220	GAS5	GENCODE	469	chr1	-
TCONS_00013475	XLOC_006123	LincRNAs identified	2028	chr7	+
ENST00000447028	AP003774.1	GENCODE	672	chr11	-
ENST00000455699	RP11-80H5.7	GENCODE	667	chr10	-
ENST00000555983	RP11-203M5.4	GENCODE	334	chr14	-
ENST00000555983	RP11-203M5.4	GENCODE	334	chr14	-
ENST00000555983	RP11-203M5.4	GENCODE	334	chr14	-
ENST00000562894	CTD-2313J17.5	GENCODE	1736	chr15	-
BF969023		LincRNAs identified	778	chr7	-
ENST00000562471	RP11-7K24.3	GENCODE	2022	chr6	-
ENST00000518528	PVT1	GENCODE	624	chr8	+

173833153	173837127	ENST00000416952,	CCATTGGATGTG(60674	bidirectional
99740072	99740879		TTACCTTGGCTCTCTAAGCTGTATCT	intergenic
30536236	30544457		CCGCCTGCAGGC/ 79017	exon sense-overlappi
30536236	30544457		CCGCCTGCAGGC/ 79017	exon sense-overlappi
30536236	30544457		CCGCCTGCAGGC/ 79017	exon sense-overlappi
30536236	30544457		CCGCCTGCAGGC/ 79017	exon sense-overlappi
19031593	19032702		GAGCTAACATTATATGTCTCAGCAT(	intergenic
61721293	61722165		GCTTACAACAGAATCTACAAGGACA	intronic antisense
140405000	140406437		ATGGATTTGAATTAGTGAATATAAA	exon sense-overlappi
52812293	52824289		GACTGGTATTCTGTTATCCACAAGA(	bidirectional
10522724	10577495		GAGCCTCTAAGT/ 80063	exon sense-overlappi
10522724	10577495		GAGCCTCTAAGT/ 80063	exon sense-overlappi
122368119	122571581		AAGACAGATGGTGATGGATCAGAG(	intergenic
9825743	9826389	NR_046235	CCGGGGGACCGGCCGCGACTGCGG(	intergenic
62619459	62623217	ENST00000541416,	CTTGCTTAGCCGT 23642	bidirectional
62619459	62623217	ENST00000541416,	CTTGCTTAGCCGT 23642	bidirectional
62619459	62623217	ENST00000541416,	CTTGCTTAGCCGT 23642	bidirectional
138392476	138396519		CATGGATAGGAT' 51116	exon sense-overlappi
43044005	43129458		ACCACGCTGGGG. 5754	exon sense-overlappi
43044005	43129458		ACCACGCTGGGG. 5754	exon sense-overlappi
43044005	43129458		ACCACGCTGGGG. 5754	exon sense-overlappi
43044005	43129458		ACCACGCTGGGG. 5754	exon sense-overlappi
43044005	43129458		ACCACGCTGGGG. 5754	exon sense-overlappi
99597122	99609247		CATGCCCGGCCCTCCCTTTCTTTTGT	intergenic
42839725	42854667		AAGTGAAGAGGT 100505783	bidirectional
11315541	11352918		ATAACAAGGTGGACCTAAAACTGG(	intergenic
51381990	51424041		TGTCTGTCTGTG(100874074	intronic antisense
62718282	62722504		GTTCCCACAAAACAATTCAGATTGT(	intergenic
17325902	17338070		CATTTAACAGTGTTGTGAACCACTT(	bidirectional
127393858	127408062		CAGACTCGGGCA. 399821	bidirectional
127393858	127408062		CAGACTCGGGCA. 399821	bidirectional
130033611	130081051		GGAAATCCAGTG' 95681	exon sense-overlappi
130033611	130081051		GGAAATCCAGTG' 95681	exon sense-overlappi
130033611	130081051		GGAAATCCAGTG' 95681	exon sense-overlappi
130033611	130081051		GGAAATCCAGTG' 95681	exon sense-overlappi
30583575	30595168		TAAAAGCAAATGCCATGGTCTGCAG	natural antisense
30583575	30595168		TAAAAGCAAATGCCATGGTCTGCAG	natural antisense
30583575	30595168		TAAAAGCAAATGCCATGGTCTGCAG	natural antisense
111133764	111142102	NR_027244, NR_04(	ATGTGCTTGGTGT 440894	intergenic
31318435	31328436		TATTAGCTCATCT 150291	natural antisense
23594503	23624667		AACTTTCTGAATT 23428	exon sense-overlappi
23594503	23624667		AACTTTCTGAATT 23428	exon sense-overlappi
23594503	23624667		AACTTTCTGAATT 23428	exon sense-overlappi
23594503	23624667		AACTTTCTGAATT 23428	exon sense-overlappi
71601067	71602662		GGAATAAAAATG 100507113	intron sense-overlapp
71601067	71602662		GGAATAAAAATG 100507113	intron sense-overlapp
71601067	71602662		GGAATAAAAATG 100507113	intron sense-overlapp
71601067	71602662		GGAATAAAAATG 100507113	intron sense-overlapp
215674994	215698897		ATAAAAAGCGCTGTTTTGACTGCTG'	intergenic
3782438	3799884	NR_034031	ATCTCGACCTGCCAAGTATACAGCT.	intergenic
173835353	173837125	ENST00000421068	GAAAATGTGAAA 60674	bidirectional
173835353	173837125	ENST00000421068	GAAAATGTGAAA 60674	bidirectional
63831931	63836925		ATCGTGATAGTCATGTGATATAAAT'	intergenic
64092521	64096976		TGGAAACACCCAGCTCTCCACCAAG	intergenic
91454051	91457685		GATATTTTCTGACTTGGGGATTAAT.	intergenic
20789321	20790303		GCACAACTACTACTTGAAACTCAAA	intron sense-overlapp
20789321	20790303		GCACAACTACTACTTGAAACTCAAA	intron sense-overlapp
20789321	20790303		GCACAACTACTACTTGAAACTCAAA	intron sense-overlapp
93322986	93324722		GTACATTTAAAGAGGCAATGCTACT	intron sense-overlapp
127188015	127188758		GCCTTTGGTTTTTATCAGCAGAGCT(	intergenic
42059975	42061997		GAACCCAGTCACTTTGCTGTTTTTCT	intergenic
128808065	128903206		ATTCCACTTACG(5820	intergenic

NM_032522	ZBTB37	zinc finger and BTB	+	173837492	173842778
NM_001199815	GGCT	gamma-glutamylcycl	-	30536236	30544457
NM_001199816	GGCT	gamma-glutamylcycl	-	30536236	30544457
NM_001199817	GGCT	gamma-glutamylcycl	-	30536236	30544457
NM_024051	GGCT	gamma-glutamylcycl	-	30536236	30544457
NM_017780	CHD7	chromodomain-helica	+	61591323	61780586
NM_004546	NDUFB2	NADH dehydrogenas	+	140396480	140406446
NM_052937	PCMTD1	protein-L-isoaspartat	-	52730139	52811735
NM_001256160	ATF7IP2	activating transcriptio	+	10479911	10577495
NM_024997	ATF7IP2	activating transcriptio	+	10479911	10577495
NM_001012662	SLC3A2	4F2 cell-surface antig	+	62623483	62656355
NM_001012664	SLC3A2	4F2 cell-surface antig	+	62623483	62656355
NM_002394	SLC3A2	4F2 cell-surface antig	+	62623483	62656355
NM_016034	MRPS2	28S ribosomal protei	+	138392476	138396519
NM_001270398	PTK7	inactive tyrosine-prot	+	43044551	43129458
NM_002821	PTK7	inactive tyrosine-prot	+	43044005	43129458
NM_152880	PTK7	inactive tyrosine-prot	+	43044005	43129458
NM_152881	PTK7	inactive tyrosine-prot	+	43044005	43129458
NM_152882	PTK7	inactive tyrosine-prot	+	43044005	43129458
NM_016470	C20orf111	uncharacterized prote	-	42824580	42839546
NM_198989	DLEU7	leukemia-associated	-	51286758	51417885
NM_001621	AHR	aryl hydrocarbon rec	+	17338275	17385775
NM_001202438	C10orf137	erythroid differentiat	+	127408083	127452712
NM_015608	C10orf137	erythroid differentiat	+	127408083	127452712
NM_001257158	CEP41	centrosomal protein (	-	130033611	130081051
NM_001257159	CEP41	centrosomal protein (	-	130033611	130081051
NM_001257160	CEP41	centrosomal protein (	-	130058874	130081051
NM_018718	CEP41	centrosomal protein (	-	130033611	130081051
NM_001024683	ZNF688	zinc finger protein 68	-	30581018	30583728
NM_145271	ZNF688	zinc finger protein 68	-	30581018	30583728
NM_152458	ZNF785	zinc finger protein 78	-	30591993	30597092
NM_014941	MORC2	MORC family CW-ty	-	31322599	31364187
NM_001267036	SLC7A8	large neutral amino a	-	23594503	23624667
NM_001267037	SLC7A8	large neutral amino a	-	23594503	23623659
NM_012244	SLC7A8	large neutral amino a	-	23594503	23652869
NM_182728	SLC7A8	large neutral amino a	-	23594503	23623659
NM_001014972	ZNF638	zinc finger protein 63	+	71558884	71662191
NM_001252612	ZNF638	zinc finger protein 63	+	71558884	71662191
NM_001252613	ZNF638	zinc finger protein 63	+	71558884	71662191
NM_014497	ZNF638	zinc finger protein 63	+	71558884	71662191
NM_001122770	ZBTB37	zinc finger and BTB	+	173837492	173855774
NM_032522	ZBTB37	zinc finger and BTB	+	173837492	173842778
NM_021178	CCNB1IP1	E3 ubiquitin-protein	-	20779526	20801471
NM_182849	CCNB1IP1	E3 ubiquitin-protein	-	20779526	20801471
NM_182852	CCNB1IP1	E3 ubiquitin-protein	-	20779526	20801471
ENST00000557398	FAM174B	family with sequence	-	93160677	93353028

825.74361	203.031225	9.278482	7.956229	801.2823	829.60156
1701.1838	395.091987	10.160354	8.80122	1559.7391	472.0801
12459.3775	1640.16715	13.179714	11.08638	12612.404	12825.018
12459.3775	1640.16715	13.179714	11.08638	12612.404	12825.018
12459.3775	1640.16715	13.179714	11.08638	12612.404	12825.018
12459.3775	1640.16715	13.179714	11.08638	12612.404	12825.018
17.046493	6.197176	3.758696	2.755946	21.663828	16.364216
31.726295	8.62177	4.629715	3.029252	40.688763	24.014835
1653.54529	443.805953	10.139641	8.915509	1348.6833	1176.5383
697.548048	179.279405	9.005878	7.819962	661.48035	800.53845
152.479415	42.004561	6.86576	5.687134	184.1532	189.74252
152.479415	42.004561	6.86576	5.687134	184.1532	189.74252
23.687829	7.253683	4.154574	2.877333	18.19171	23.637138
2532.772883	276.338843	10.766395	8.47637	3453.0452	4195.4473
202.088267	47.430938	7.213393	5.845612	212.11034	255.74612
202.088267	47.430938	7.213393	5.845612	212.11034	255.74612
202.088267	47.430938	7.213393	5.845612	212.11034	255.74612
220.96924	47.092114	7.271167	5.8272	252.5663	391.6752
65.241598	10.807662	5.656222	3.620369	63.60374	38.817352
65.241598	10.807662	5.656222	3.620369	63.60374	38.817352
65.241598	10.807662	5.656222	3.620369	63.60374	38.817352
65.241598	10.807662	5.656222	3.620369	63.60374	38.817352
65.241598	10.807662	5.656222	3.620369	63.60374	38.817352
3726.469917	1075.235358	11.387588	10.164113	4462.998	3947.2114
56.991465	11.834765	5.430553	3.832295	67.28365	52.315914
56.310564	7.841496	4.906133	2.992526	84.50533	133.12787
431.952717	61.556308	8.342922	6.251058	503.77542	582.11163
27.826795	9.945997	4.469963	3.445371	29.016083	28.462967
1040.751158	278.29057	9.546267	8.467394	1199.7701	652.85565
27.732859	7.652417	4.42545	3.152008	23.348965	22.455671
27.732859	7.652417	4.42545	3.152008	23.348965	22.455671
112.02536	35.563069	6.334668	5.301449	69.98947	96.229675
112.02536	35.563069	6.334668	5.301449	69.98947	96.229675
112.02536	35.563069	6.334668	5.301449	69.98947	96.229675
112.02536	35.563069	6.334668	5.301449	69.98947	96.229675
167.574742	49.350798	7.01194	5.798413	147.37535	159.58298
167.574742	49.350798	7.01194	5.798413	147.37535	159.58298
167.574742	49.350798	7.01194	5.798413	147.37535	159.58298
590.740377	117.377984	8.631553	6.847177	750.7848	1174.9979
14.300388	5.194731	3.512839	2.47567	11.935401	15.454501
121.495326	44.525064	6.54342	5.059649	141.32957	99.31362
121.495326	44.525064	6.54342	5.059649	141.32957	99.31362
121.495326	44.525064	6.54342	5.059649	141.32957	99.31362
121.495326	44.525064	6.54342	5.059649	141.32957	99.31362
26.815522	12.668986	4.389839	3.296905	37.281967	24.363056
26.815522	12.668986	4.389839	3.296905	37.281967	24.363056
26.815522	12.668986	4.389839	3.296905	37.281967	24.363056
26.815522	12.668986	4.389839	3.296905	37.281967	24.363056
31.630308	6.149413	4.459768	2.672899	33.32889	15.375878
129.938343	18.059789	6.556907	4.410444	131.01065	151.528
197.008993	24.082748	7.212518	4.845036	205.30688	200.8989
197.008993	24.082748	7.212518	4.845036	205.30688	200.8989
128.000104	16.957933	6.20771	4.352922	53.717533	61.22733
19585.21367	4717.65173	13.568235	10.406279	18195.207	26315.326
38.464026	12.501376	4.719597	3.542766	37.31334	10.8439
34.521923	10.703985	4.782125	3.575239	40.613953	31.35504
34.521923	10.703985	4.782125	3.575239	40.613953	31.35504
34.521923	10.703985	4.782125	3.575239	40.613953	31.35504
30.309166	8.995198	4.415139	3.376911	35.90104	55.8697
31.190591	7.937926	4.554173	2.975729	27.81531	32.371056
1082.393838	198.874326	9.571645	7.944782	708.8701	916.28467
50.27095	11.701212	5.257758	3.597401	41.295135	31.63467

761.5928	1073.6698	571.1064	917.2088	179.47456	85.19255
2635.4375	2277.1072	1252.1665	2010.5724	470.7644	157.2321
13075.977	10097.15	12489.326	13656.39	1629.3751	1633.6287
13075.977	10097.15	12489.326	13656.39	1629.3751	1633.6287
13075.977	10097.15	12489.326	13656.39	1629.3751	1633.6287
13075.977	10097.15	12489.326	13656.39	1629.3751	1633.6287
13.424468	22.479576	11.192372	17.154497	8.0925	4.9999995
43.480194	24.260883	29.372347	28.54075	7.251314	4.9999995
1132.5747	2958.4392	822.35504	2482.6812	432.5055	97.46549
491.33533	429.3413	906.7261	895.86676	174.85469	126.0046
168.07314	104.52512	122.68971	145.6928	37.710308	28.80987
168.07314	104.52512	122.68971	145.6928	37.710308	28.80987
16.604858	15.674851	38.638176	29.380241	11.758828	4.9999995
2595.845	1134.5177	1900.6708	1917.1113	296.8642	337.05075
176.80753	88.62874	235.1849	244.05197	54.830853	51.690655
176.80753	88.62874	235.1849	244.05197	54.830853	51.690655
176.80753	88.62874	235.1849	244.05197	54.830853	51.690655
76.57335	176.00739	200.75426	228.23894	46.319977	29.2676
63.780384	91.10264	53.67909	80.46638	6.760025	6.187459
63.780384	91.10264	53.67909	80.46638	6.760025	6.187459
63.780384	91.10264	53.67909	80.46638	6.760025	6.187459
63.780384	91.10264	53.67909	80.46638	6.760025	6.187459
63.780384	91.10264	53.67909	80.46638	6.760025	6.187459
4356.679	1697.1542	3908.9146	3985.8623	1279.6835	785.37354
62.890537	73.29779	23.24101	62.91989	16.3573	11.470211
20.081306	62.443455	5.423503	32.281918	13.035229	14.01375
349.1288	442.31284	292.98676	421.40085	78.187164	76.21745
25.979181	39.1264	20.63496	23.74118	11.06412	5.8285155
1713.2244	1098.1117	637.69714	942.84796	240.0785	181.72707
28.313442	43.94608	15.447758	32.88524	11.806369	10.387799
28.313442	43.94608	15.447758	32.88524	11.806369	10.387799
56.897568	189.33176	95.84055	163.86314	24.570518	50.983227
56.897568	189.33176	95.84055	163.86314	24.570518	50.983227
56.897568	189.33176	95.84055	163.86314	24.570518	50.983227
56.897568	189.33176	95.84055	163.86314	24.570518	50.983227
129.3733	219.3759	140.63643	209.10449	37.12461	18.60728
129.3733	219.3759	140.63643	209.10449	37.12461	18.60728
129.3733	219.3759	140.63643	209.10449	37.12461	18.60728
221.53992	384.80814	480.23685	532.07465	169.64392	74.54604
11.21088	15.63277	13.675068	17.893711	5.1750097	4.9999995
171.83505	115.23037	83.82773	117.435616	69.1461	6.3400064
171.83505	115.23037	83.82773	117.435616	69.1461	6.3400064
171.83505	115.23037	83.82773	117.435616	69.1461	6.3400064
171.83505	115.23037	83.82773	117.435616	69.1461	6.3400064
32.36777	22.900667	16.407646	27.572025	18.047607	4.9999995
32.36777	22.900667	16.407646	27.572025	18.047607	4.9999995
32.36777	22.900667	16.407646	27.572025	18.047607	4.9999995
32.36777	22.900667	16.407646	27.572025	18.047607	4.9999995
67.25724	14.197629	32.664803	26.957405	4.9999995	4.9999995
92.57207	55.008816	179.86555	169.64497	16.450148	16.433687
174.1298	277.13382	104.297295	220.28726	25.13636	15.814638
174.1298	277.13382	104.297295	220.28726	25.13636	15.814638
49.05279	322.2778	54.906803	226.81837	13.041719	18.338121
12869.161	3774.906	31341.264	25015.418	6038.5444	433.13522
44.204994	20.467611	60.52738	57.426933	9.625355	4.9999995
29.693241	43.554787	26.59059	35.32393	7.53261	9.102055
29.693241	43.554787	26.59059	35.32393	7.53261	9.102055
29.693241	43.554787	26.59059	35.32393	7.53261	9.102055
23.954208	12.148489	32.314346	21.667215	9.459971	9.30306
14.475799	44.224705	23.683372	44.573303	8.164168	4.9999995
491.27686	1631.438	1165.104	1581.3894	196.50238	113.838394
44.12847	85.780106	38.339745	60.447575	10.133159	4.9999995

249.72124	243.39584	249.6204	210.78276	9.185014	9.365079
771.93	210.2297	461.10617	299.28955	10.142613	8.573693
1546.2723	1660.5417	1988.9777	1382.2074	13.172068	13.186595
1546.2723	1660.5417	1988.9777	1382.2074	13.172068	13.186595
1546.2723	1660.5417	1988.9777	1382.2074	13.172068	13.186595
1546.2723	1660.5417	1988.9777	1382.2074	13.172068	13.186595
4.9999995	4.9999995	7.0521727	7.038385	3.9766154	4.045346
18.39262	4.9999995	11.08669	4.9999995	4.883735	4.542522
875.41876	409.02667	511.0799	337.3394	9.932707	9.8462105
227.89053	173.04446	219.28384	154.59831	8.89629	9.313969
40.693848	47.206123	60.84315	36.764065	7.06817	7.3247967
40.693848	47.206123	60.84315	36.764065	7.06817	7.3247967
8.969156	7.7941136	4.9999995	4.9999995	3.7158005	4.5212617
243.69601	236.75803	303.62207	240.042	11.276378	11.602852
51.035896	26.919378	60.107094	40.001755	7.277364	7.732756
51.035896	26.919378	60.107094	40.001755	7.277364	7.732756
51.035896	26.919378	60.107094	40.001755	7.277364	7.732756
63.99704	41.79404	64.296364	36.87766	7.5219345	8.319926
8.511949	13.076821	11.09667	19.21305	5.533642	5.178852
8.511949	13.076821	11.09667	19.21305	5.533642	5.178852
8.511949	13.076821	11.09667	19.21305	5.533642	5.178852
8.511949	13.076821	11.09667	19.21305	5.533642	5.178852
8.511949	13.076821	11.09667	19.21305	5.533642	5.178852
2719.3608	648.52856	598.2824	420.18335	11.638906	11.534537
12.49518	8.662658	11.551481	10.47176	5.6117983	5.5721846
4.9999995	4.9999995	4.9999995	4.9999995	5.94795	6.8243346
62.256153	55.456326	53.901375	43.319378	8.493938	8.8755
15.443761	13.102739	9.236846	4.9999995	4.4060326	4.773078
288.6321	324.06927	355.75095	279.48553	9.751583	9.028195
7.593409	4.9999995	5.520022	5.6069036	4.0836406	4.4502254
7.593409	4.9999995	5.520022	5.6069036	4.0836406	4.4502254
9.011126	56.612213	34.516193	37.68514	5.6704035	6.3725333
9.011126	56.612213	34.516193	37.68514	5.6704035	6.3725333
9.011126	56.612213	34.516193	37.68514	5.6704035	6.3725333
9.011126	56.612213	34.516193	37.68514	5.6704035	6.3725333
44.51141	44.940342	93.29019	57.630955	6.7483788	7.076006
44.51141	44.940342	93.29019	57.630955	6.7483788	7.076006
44.51141	44.940342	93.29019	57.630955	6.7483788	7.076006
286.77954	38.769627	83.001434	51.527344	9.085781	9.844387
5.9933796	4.9999995	4.9999995	4.9999995	3.0915568	3.9699037
133.44121	21.447947	18.825071	17.95005	6.6916122	6.4187746
133.44121	21.447947	18.825071	17.95005	6.6916122	6.4187746
133.44121	21.447947	18.825071	17.95005	6.6916122	6.4187746
133.44121	21.447947	18.825071	17.95005	6.6916122	6.4187746
36.152893	4.9999995	6.8134203	4.9999995	4.7549224	4.5640707
36.152893	4.9999995	6.8134203	4.9999995	4.7549224	4.5640707
36.152893	4.9999995	6.8134203	4.9999995	4.7549224	4.5640707
36.152893	4.9999995	6.8134203	4.9999995	4.7549224	4.5640707
4.9999995	4.9999995	10.878571	6.017909	4.596338	3.9625843
19.406027	9.698637	26.809206	19.561028	6.586121	7.0080676
27.769058	17.214935	34.97294	23.588556	7.228572	7.4005375
27.769058	17.214935	34.97294	23.588556	7.228572	7.4005375
12.06175	26.392262	14.537931	17.375818	5.278879	5.77373
20749.883	144.43178	699.832	240.08398	13.695072	14.284758
23.606876	4.9999995	20.58204	11.193989	4.7567625	3.5049899
17.915028	16.75135	5.970731	6.9521337	4.8806825	4.9009495
17.915028	16.75135	5.970731	6.9521337	4.8806825	4.9009495
17.915028	16.75135	5.970731	6.9521337	4.8806825	4.9009495
4.9999995	8.54564	14.164751	7.497767	4.7030597	5.652783
15.009358	4.9999995	9.454034	4.9999995	4.345584	4.942521
294.18243	235.8638	181.8476	171.01135	9.000512	9.504933
7.6389136	22.217678	10.789061	14.428459	4.905142	4.910768

9.222115	9.747093	8.899571	9.252018	7.7407985	7.264973
11.007731	10.828189	10.020022	10.389878	9.14473	8.145644
13.375594	12.941439	13.278634	13.123952	10.972676	11.56372
13.375594	12.941439	13.278634	13.123952	10.972676	11.56372
13.375594	12.941439	13.278634	13.123952	10.972676	11.56372
13.375594	12.941439	13.278634	13.123952	10.972676	11.56372
3.4193892	3.99515	3.437369	3.678304	3.2188282	2.4279919
5.1194477	4.1164303	4.7363424	4.3798122	3.0516148	2.4279919
9.772156	11.193256	9.419062	10.674453	9.020102	7.461958
8.590928	8.437454	9.573069	9.223558	7.702283	7.826035
7.0632067	6.357002	6.717967	6.6634164	5.4737206	5.686953
7.0632067	6.357002	6.717967	6.6634164	5.4737206	5.686953
3.7368288	3.4152188	5.116016	4.422317	3.7850182	2.4279919
10.984496	9.824013	10.588401	10.322233	8.479207	9.258244
7.1370745	6.1149616	7.6329484	7.3852534	6.0119452	6.5408387
7.1370745	6.1149616	7.6329484	7.3852534	6.0119452	6.5408387
7.1370745	6.1149616	7.6329484	7.3852534	6.0119452	6.5408387
5.9435587	7.1363106	7.4132276	7.2920423	5.761828	5.710879
5.6903105	6.1562243	5.5615935	5.816707	2.9538639	3.380406
5.6903105	6.1562243	5.5615935	5.816707	2.9538639	3.380406
5.6903105	6.1562243	5.5615935	5.816707	2.9538639	3.380406
5.6903105	6.1562243	5.5615935	5.816707	2.9538639	3.380406
5.6903105	6.1562243	5.5615935	5.816707	2.9538639	3.380406
11.745006	10.419105	11.625542	11.362432	10.616147	10.476458
5.6672378	5.8350544	4.4284105	5.468632	4.2623553	4.310388
4.0064034	5.5890093	2.511003	4.558096	3.9295127	4.6194925
8.108717	8.479794	7.9522905	8.147293	6.510647	7.0997066
4.3896694	4.867869	4.26334	4.1197896	3.6976042	3.2838042
10.374961	9.776578	9.048699	9.2975855	8.161489	8.3553
4.5133567	5.0476193	3.8734958	4.5843635	3.7902727	4.1622868
4.5133567	5.0476193	3.8734958	4.5843635	3.7902727	4.1622868
5.5177927	7.2434807	6.370657	6.833143	4.8611603	6.5200653
5.5177927	7.2434807	6.370657	6.833143	4.8611603	6.5200653
5.5177927	7.2434807	6.370657	6.833143	4.8611603	6.5200653
5.5177927	7.2434807	6.370657	6.833143	4.8611603	6.5200653
6.693942	7.4663982	6.9156675	7.1712484	5.450808	5.0393906
6.693942	7.4663982	6.9156675	7.1712484	5.450808	5.0393906
6.693942	7.4663982	6.9156675	7.1712484	5.450808	5.0393906
7.468946	8.268391	8.644871	8.47694	7.651517	7.0639057
3.16072	3.4102354	3.7047482	3.7398686	2.5838494	2.4279919
7.0961413	6.515286	6.1856904	6.353018	6.344431	3.4162364
7.0961413	6.515286	6.1856904	6.353018	6.344431	3.4162364
7.0961413	6.515286	6.1856904	6.353018	6.344431	3.4162364
7.0961413	6.515286	6.1856904	6.353018	6.344431	3.4162364
4.705545	4.02563	3.95918	4.3296833	4.4112144	2.4279919
4.705545	4.02563	3.95918	4.3296833	4.4112144	2.4279919
4.705545	4.02563	3.95918	4.3296833	4.4112144	2.4279919
4.705545	4.02563	3.95918	4.3296833	4.4112144	2.4279919
5.7634716	3.2532878	4.886387	4.2965393	2.3467326	2.4279919
6.2101474	5.3941956	7.261121	6.8817887	4.2717	4.8537564
7.1133122	7.7986	6.4917765	7.242311	4.8944025	4.8016486
7.1133122	7.7986	6.4917765	7.242311	4.8944025	4.8016486
5.301098	8.011875	5.5957165	7.284961	3.93022	5.0198116
13.349262	11.538073	14.559727	13.982518	12.856338	9.628041
5.1468153	3.8366914	5.729191	5.34313	3.488935	2.4279919
4.583099	5.0343432	4.6118298	4.681844	3.1078713	3.961549
4.583099	5.0343432	4.6118298	4.681844	3.1078713	3.961549
4.583099	5.0343432	4.6118298	4.681844	3.1078713	3.961549
4.27495	2.9949439	4.8701854	3.9949098	3.4596407	3.99287
3.5303166	5.0560074	4.4563026	4.994309	3.2322395	2.4279919
8.590663	10.367367	9.923487	10.042907	7.864052	7.682559
5.1439953	6.067602	5.1070495	5.41199	3.5656352	2.4279919

7.9117007	8.349012	8.221882	8.249008
9.540466	8.121112	9.090699	8.764672
10.53186	11.214787	11.215674	11.019561
10.53186	11.214787	11.215674	11.019561
10.53186	11.214787	11.215674	11.019561
10.53186	11.214787	11.215674	11.019561
2.350709	2.3408759	3.1192646	3.0780046
4.2551594	2.3408759	3.7471201	2.352752
9.719757	9.126838	9.2286625	8.935734
7.774795	7.813798	8.030203	7.772655
5.3448844	5.8199954	6.191412	5.605837
5.3448844	5.8199954	6.191412	5.605837
3.2858248	3.050597	2.3618152	2.352752
7.8780603	8.306798	8.4931555	8.442755
5.667463	4.947808	6.173862	5.7317557
5.667463	4.947808	6.173862	5.7317557
5.667463	4.947808	6.173862	5.7317557
5.9823585	5.6239634	6.273701	5.610468
3.2147503	3.82942	3.7484207	4.5953503
3.2147503	3.82942	3.7484207	4.5953503
3.2147503	3.82942	3.7484207	4.5953503
3.2147503	3.82942	3.7484207	4.5953503
3.2147503	3.82942	3.7484207	4.5953503
11.358265	9.810787	9.46278	9.26024
3.7250936	3.2128968	3.8028843	3.6801534
2.350709	2.3408759	2.3618152	2.352752
5.9459763	6.0743933	6.0185604	5.857064
4.0126276	3.8323848	3.4930515	2.352752
8.12084	8.788202	8.711066	8.667468
3.0675511	2.3408759	2.800991	2.750073
3.0675511	2.3408759	2.800991	2.750073
3.2910297	6.109677	5.3836217	5.643138
3.2910297	6.109677	5.3836217	5.643138
3.2910297	6.109677	5.3836217	5.643138
3.2910297	6.109677	5.3836217	5.643138
5.4737206	5.740714	6.7972956	6.2885485
5.4737206	5.740714	6.7972956	6.2885485
5.4737206	5.740714	6.7972956	6.2885485
8.110173	5.5130763	6.629179	6.115211
2.7867377	2.3408759	2.3618152	2.352752
7.0150757	4.5892787	4.5012565	4.4916153
7.0150757	4.5892787	4.5012565	4.4916153
7.0150757	4.5892787	4.5012565	4.4916153
7.0150757	4.5892787	4.5012565	4.4916153
5.178852	2.3408759	3.0697432	2.352752
5.178852	2.3408759	3.0697432	2.352752
5.178852	2.3408759	3.0697432	2.352752
5.178852	2.3408759	3.0697432	2.352752
2.350709	2.3408759	3.7196598	2.8514273
4.3253593	3.3757267	5.0138154	4.6223073
4.815949	4.247795	5.4017715	4.908648
4.815949	4.247795	5.4017715	4.908648
3.6791968	4.913923	4.1334805	4.440899
14.27892	7.547723	9.683542	8.443109
4.5898705	2.3408759	4.6292048	3.7797172
4.2156496	4.2087736	2.9000905	3.0575004
4.2156496	4.2087736	2.9000905	3.0575004
4.2156496	4.2087736	2.9000905	3.0575004
2.350709	3.1897757	4.0952086	3.1732607
3.9723628	2.3408759	3.5281546	2.352752
8.14705	8.299048	7.7542386	7.9217434
3.0746772	4.64349	3.7086086	4.164003

ASHGA5P015436	0.010252899	0.062647807	2.0902879	up	noncoding
ASHGA5P015436	0.010252899	0.062647807	2.0902879	up	noncoding
ASHGA5P050237	0.000142128	0.01066938	2.3097321	up	noncoding
ASHGA5P000074	0.013710383	0.071739124	2.4234183	up	noncoding
ASHGA5P036086	0.012810114	0.069277826	4.5445675	up	noncoding
ASHGA5P032843	0.032597157	0.113617438	2.7061136	up	noncoding
ASHGA5P035875	0.036365146	0.120940502	2.2344407	up	noncoding
ASHGA5P042992	0.026720197	0.102009237	3.3727194	up	noncoding
ASHGA5P042992	0.026720197	0.102009237	3.3727194	up	noncoding
ASHGA5P057927	0.028296362	0.104997102	2.0423313	up	noncoding
ASHGA5P046415	0.000757172	0.018563586	3.2987685	up	noncoding
ASHGA5P044513	0.002951313	0.035133618	2.7100801	up	noncoding
ASHGA5P056802	0.009123956	0.059505105	3.6781161	up	noncoding
ASHGA5P030874	0.030508161	0.109579945	2.2714207	up	noncoding
ASHGA5P030874	0.030508161	0.109579945	2.2714207	up	noncoding
ASHGA5P032809	0.02060528	0.088106916	2.8314136	up	noncoding
ASHGA5P022044	0.007162456	0.053397886	7.5916499	up	noncoding
ASHGA5P041189	0.008699746	0.058118065	2.3753054	up	noncoding
ASHGA5P041189	0.008699746	0.058118065	2.3753054	up	noncoding
ASHGA5P041189	0.008699746	0.058118065	2.3753054	up	noncoding
ASHGA5P051267	0.026661106	0.101899297	2.8254555	up	noncoding
ASHGA5P051267	0.026661106	0.101899297	2.8254555	up	noncoding
ASHGA5P047797	0.040943416	0.129322917	2.1161484	up	noncoding
ASHGA5P028100	0.015331165	0.075787682	2.0836718	up	noncoding
ASHGA5P052634	0.000363467	0.013588019	2.3897516	up	noncoding
ASHGA5P019912	0.034011842	0.116461123	2.1379758	up	noncoding
ASHGA5P048846	0.010428192	0.063044889	2.8300143	up	noncoding
ASHGA5P043501	0.000122736	0.010164781	3.5583795	up	noncoding
ASHGA5P047776	0.002583449	0.032855929	3.6666333	up	noncoding
ASHGA5P044346	0.004213678	0.041275918	5.4155744	up	noncoding
ASHGA5P021486	0.047907502	0.141797358	2.7635082	up	noncoding
ASHGA5P021486	0.047907502	0.141797358	2.7635082	up	noncoding
ASHGA5P021486	0.047907502	0.141797358	2.7635082	up	noncoding
ASHGA5P021486	0.047907502	0.141797358	2.7635082	up	noncoding
ASHGA5P021486	0.047907502	0.141797358	2.7635082	up	noncoding
ASHGA5P021486	0.047907502	0.141797358	2.7635082	up	noncoding
ASHGA5P045509	0.025585823	0.099699462	2.9112901	up	noncoding
ASHGA5P015035	3.29657E-06	0.002593655	8.1472203	up	noncoding
ASHGA5P048324	0.004845522	0.044259175	3.3572189	up	noncoding
ASHGA5P037069	0.007138524	0.053350909	2.3519455	up	noncoding
ASHGA5P037069	0.007138524	0.053350909	2.3519455	up	noncoding
ASHGA5P037069	0.007138524	0.053350909	2.3519455	up	noncoding
ASHGA5P037069	0.007138524	0.053350909	2.3519455	up	noncoding
ASHGA5P036158	0.006046957	0.049581613	3.8140123	up	noncoding
ASHGA5P036914	0.044787079	0.135994806	2.3963826	up	noncoding
ASHGA5P029948	0.037618802	0.123112847	2.4463559	up	noncoding
ASHGA5P022604	0.003321461	0.037316455	3.02233	up	noncoding
ASHGA5P021124	0.002049313	0.029808035	2.5271184	up	noncoding
ASHGA5P031778	0.002021734	0.029606317	5.9189865	up	noncoding
ASHGA5P035667	0.002468712	0.032298511	2.0797308	up	noncoding
ASHGA5P044557	0.007660217	0.054952989	2.2094682	up	noncoding
ASHGA5P031306	0.003991383	0.040472675	2.2649687	up	noncoding
ASHGA5P031306	0.003991383	0.040472675	2.2649687	up	noncoding
ASHGA5P048259	0.000868903	0.01986768	2.2106774	up	noncoding
ASHGA5P048259	0.000868903	0.01986768	2.2106774	up	noncoding
ASHGA5P048259	0.000868903	0.01986768	2.2106774	up	noncoding
ASHGA5P026748	0.039959444	0.127780027	2.1582883	up	noncoding
ASHGA5P041623	0.013025716	0.069856339	2.3539673	up	noncoding
ASHGA5P043928	0.000386817	0.014011107	3.2534005	up	noncoding
ASHGA5P048981	0.000204465	0.01146874	2.2958989	up	noncoding
ASHGA5P048981	0.000204465	0.01146874	2.2958989	up	noncoding
ASHGA5P048981	0.000204465	0.01146874	2.2958989	up	noncoding

NR_026879	FAM185A	RefSeq	2055	chr7	+
NR_026879	FAM185A	RefSeq	2055	chr7	+
ENST00000575148	APOC1P1	GENCODE	560	chr19	+
AV648285		LincRNAs identified	390	chr12	-
uc002tji.1	AK123617	UCSC_knowngene	3471	chr2	+
ENST00000428642	RP11-496N12.6	GENCODE	397	chr1	-
TCONS_00026909	XLOC_012981	LincRNAs identified	600	chr19	+
ENST00000439852	NPSR1-AS1	GENCODE	710	chr7	-
ENST00000439852	NPSR1-AS1	GENCODE	710	chr7	-
TCONS_00023843	XLOC_011616	LincRNAs identified	3947	chr15	-
ENST00000399966	WASIR1	GENCODE	1054	chrX	-
ENST00000530778	RP11-383J24.5	GENCODE	1767	chr8	-
TCONS_00008755	XLOC_003455	LincRNAs identified	210	chr4	+
ENST00000565937	RP11-304L19.3	GENCODE	596	chr16	-
ENST00000565937	RP11-304L19.3	GENCODE	596	chr16	-
TCONS_00029013	XLOC_013936	LincRNAs identified	815	chr21	+
ENST00000547779	AL589743.1	GENCODE	617	chr14	+
ENST00000457548	RP11-98D18.1	GENCODE	386	chr1	+
ENST00000457548	RP11-98D18.1	GENCODE	386	chr1	+
ENST00000457548	RP11-98D18.1	GENCODE	386	chr1	+
ENST00000566814	CTA-445C9.14	GENCODE	2032	chr22	+
ENST00000566814	CTA-445C9.14	GENCODE	2032	chr22	+
ENST00000525893	RP13-317D12.3	GENCODE	664	chr11	-
TCONS_00017110	XLOC_007908	LincRNAs identified	863	chrX	+
ENST00000455957	HCG17	GENCODE	350	chr6	-
ENST00000506340	CTC-338M12.4	GENCODE	687	chr5	+
ENST00000558687	RP11-752G15.6	GENCODE	558	chr15	+
TCONS_00014700	XLOC_006798	LincRNAs identified	248	chr8	+
TCONS_00013639	XLOC_006301	LincRNAs identified	463	chr7	+
uc003yao.3	BC062758	UCSC_knowngene	1142	chr8	-
NR_073039	MDK	RefSeq	821	chr11	+
NR_073039	MDK	RefSeq	821	chr11	+
NR_073039	MDK	RefSeq	821	chr11	+
NR_073039	MDK	RefSeq	821	chr11	+
NR_073039	MDK	RefSeq	821	chr11	+
NR_073039	MDK	RefSeq	821	chr11	+
NR_027440	LOC100272217	RefSeq	2159	chr9	-
ENST00000415883	RP11-346D19.1	GENCODE	597	chr6	+
ENST00000533740	RP11-939C17.4	GENCODE	386	chr11	-
ENST00000445073	RP11-399E6.1	GENCODE	709	chr1	+
ENST00000445073	RP11-399E6.1	GENCODE	709	chr1	+
ENST00000445073	RP11-399E6.1	GENCODE	709	chr1	+
ENST00000445073	RP11-399E6.1	GENCODE	709	chr1	+
uc002tte.3	BX648270	UCSC_knowngene	4566	chr2	+
TCONS_00025358	XLOC_012177	LincRNAs identified	1210	chr17	+
ENST00000526635	CTD-2339L15.1	GENCODE	419	chr15	-
ENST00000559518	RP11-561C5.5	GENCODE	553	chr15	-
ENST00000525656	CTD-2313N18.4	pseudogene	427	chr11	-
ENST00000571340	RP11-44F14.8	GENCODE	2142	chr16	+
ENST00000400768	AC114730.8	GENCODE	472	chr2	-
uc003yvm.3	DQ574852	UCSC_knowngene	4833	chr8	-
ENST00000432536	GAS5	GENCODE	841	chr1	-
ENST00000432536	GAS5	GENCODE	841	chr1	-
ENST00000532296	RP5-901A4.1	GENCODE	739	chr11	-
ENST00000532296	RP5-901A4.1	GENCODE	739	chr11	-
ENST00000532296	RP5-901A4.1	GENCODE	739	chr11	-
uc001pgr.3	BC040646	UCSC_knowngene	1952	chr11	+
TCONS_00020555	XLOC_009868	LincRNAs identified	2469	chr12	+
TCONS_00018637	XLOC_008986	LincRNAs identified	1817	chr10	-
ENST00000568106	LA16c-358B7.3	GENCODE	3107	chr16	-
ENST00000568106	LA16c-358B7.3	GENCODE	3107	chr16	-
ENST00000568106	LA16c-358B7.3	GENCODE	3107	chr16	-

102389398	102449672		GACACTGCAGGT 222234	exon sense-overlappi
102389398	102449672		GACACTGCAGGT 222234	exon sense-overlappi
45430060	45434450	NR_028412	GATTCAGGTTGT 342	intergenic
102601068	102601512		TGATACCATCTCCCAGACAGAAGCA	intergenic
113963502	113966973		CTAATTTATGGCATGCATATCATAT	intergenic
225634944	225653045		TCTGTAGCATTTTCTTTCTGGCAAC	intergenic
15945758	15946461	NR_015379	AATTTTCTTTAAGGTGTCCACGCAG	intergenic
34606334	34797884		GGCTTCTTTAAC 404744	intronic antisense
34606334	34797884		GGCTTCTTTAAC 404744	intronic antisense
102142909	102154530		CCTTATGCAAAGTGTGAACTTGGCC	intergenic
155244287	155246502	ENST00000329244	GGCAGTGTATTTGTAATGGTACATA	intergenic
125485050	125486817		GATCTTCAGAAATATAAATTAGCTA	bidirectional
11338388	11344494	TCONS_00007986	ACAAGGTGGACCTAAAACTGGGTG	intergenic
2144830	2147027		TTTATCTTGGTCTCAGAGCAGTGAG	intron sense-overlapp
2144830	2147027		TTTATCTTGGTCTCAGAGCAGTGAG	intron sense-overlapp
38666995	38668774		AGAATATCACGTTTTTCTGAAGTTT	intergenic
19650054	19662608	ENST00000548107	GAACAGGTAGCTCTCAACATCTCTT	intergenic
151673501	151680862		ACACCCCAGTCCTCATGGTGTGCCT	natural antisense
151673501	151680862		ACACCCCAGTCCTCATGGTGTGCCT	natural antisense
151673501	151680862		ACACCCCAGTCCTCATGGTGTGCCT	natural antisense
26908502	26910534		TGATGAAAAGATTCAAGAGGCAATT	bidirectional
26908502	26910534		TGATGAAAAGATTCAAGAGGCAATT	bidirectional
462929	463899		GGCGCTTGGGGAGGCGAGGCTACA	intronic antisense
287991	288942		GTGAGTTGCTGAGATTTCATTTTTA	intergenic
30201815	30202254		TTACTTTAAGAGACACTTACTGATG	intergenic
180673524	180684245	ENST00000505151	TCCATACAAGCTTGCAGAGTCAAGA	natural antisense
83419317	83423789		CTAGGACTCTGTGGCTGATTTATAA	intergenic
55291717	55293397		AATCTGGACTGCACTGAAACCTGGA	intergenic
154795153	154806834	NR_028090, TCON	GGACCCGGCTATGCCGTGACGGAAT	intergenic
76135660	76190696		GACCTGTGGATGTTTTTGAAAAGT	intergenic
46403204	46405387		CAGCGAGCGAAT 4192	exon sense-overlappi
46403204	46405387		CAGCGAGCGAAT 4192	exon sense-overlappi
46403204	46405387		CAGCGAGCGAAT 4192	exon sense-overlappi
46403204	46405387		CAGCGAGCGAAT 4192	exon sense-overlappi
46403204	46405387		CAGCGAGCGAAT 4192	exon sense-overlappi
46403204	46405387		CAGCGAGCGAAT 4192	exon sense-overlappi
133452736	133454881		CATGCAGTTATA 100272217	bidirectional
113852319	113864995		CTCAGGCCTTCATATTCAGGACATG	intergenic
75217981	75219436		GCAGCTTAGACACGTTTTTGTACCT	intron sense-overlapp
41708246	41750533		GAAGGATTAGGTGACAGCTAACACT	bidirectional
41708246	41750533		GAAGGATTAGGTGACAGCTAACACT	bidirectional
41708246	41750533		GAAGGATTAGGTGACAGCTAACACT	bidirectional
41708246	41750533		GAAGGATTAGGTGACAGCTAACACT	bidirectional
132442469	132457442		TAGAAGCAGACGTCTACTCACTGTC	intergenic
33640547	33652439	TCONS_00025360,	CAAGAAGCTACCCTCTCCCCACCAG	intergenic
40979920	40987296	ENST00000533146,	AGACGGGAGCTCGAGAGCTTGATCC	natural antisense
85813534	85873459		AGAGACCATGATCATAAATGTGGA	intergenic
71511708	71515691		CCTGCGTGTGCTGTGAACTCTCTGCAGCTTACATGAGC	
53420855	53422997		TTCTTCTTCAGGTAACCACAGAGTC	intergenic
242674129	242674913		CTCAGGGCCCGGAGGGGAAGGGCA	natural antisense
141531042	141535875	ENST00000560295	TTGGGGCAGGTGATTAAATTTATAT	intergenic
173834996	173836827		CACTCTTTAAAA 60674	bidirectional
173834996	173836827		CACTCTTTAAAA 60674	bidirectional
67792276	67797928		CGACCTTGGATTACAGCCCCTGGAG	intronic antisense
67792276	67797928		CGACCTTGGATTACAGCCCCTGGAG	intronic antisense
67792276	67797928		CGACCTTGGATTACAGCCCCTGGAG	intronic antisense
101978616	101980568		CGTTTACCCCTCTCAAGTGAGTTTTT	intergenic
105789467	105806934		TAAAAGATGCCATAAAACACCAAGT	exon sense-overlappi
128591476	128593410		TCACTGAATGCATAAATAAGTGTAG	intergenic
1369255	1372828	ENST00000567829	CATTTAGAAGTTCCTGTATTCCTAAT	natural antisense
1369255	1372828	ENST00000567829	CATTTAGAAGTTCCTGTATTCCTAAT	natural antisense
1369255	1372828	ENST00000567829	CATTTAGAAGTTCCTGTATTCCTAAT	natural antisense

NM_001145268	FAM185A	protein FAM185A is	+	102389398	102449672
NM_001145269	FAM185A	protein FAM185A is	+	102389398	102449672
NM_207172	NPSR1	neuropeptide S recep	+	34697896	34889590
NM_207173	NPSR1	neuropeptide S recep	+	34697896	34917944
NM_007218	RNF139	E3 ubiquitin-protein	+	125487007	125500859
NM_000296	PKD1	polycystin-1 isoform	-	2138710	2185899
NM_001009944	PKD1	polycystin-1 isoform	-	2138710	2185899
NM_001172648	CELF3	CUGBP Elav-like fai	-	151672533	151688792
NM_001172649	CELF3	CUGBP Elav-like fai	-	151672533	151688882
NM_007185	CELF3	CUGBP Elav-like fai	-	151672533	151689290
NM_001008697	TFIP11	tuftelin-interacting pi	-	26887893	26908437
NM_012143	TFIP11	tuftelin-interacting pi	-	26887893	26908437
NM_030783	PTDSS2	phosphatidylserine sy	+	450279	491387
NM_032765	TRIM52	tripartite motif-conta	-	180683385	180688119
NM_001012333	MDK	midkine isoform a pr	+	46403260	46405387
NM_001012334	MDK	midkine isoform a pr	+	46402617	46405387
NM_001270550	MDK	midkine isoform a pr	+	46402333	46405387
NM_001270551	MDK	midkine isoform a pr	+	46403260	46405387
NM_001270552	MDK	midkine isoform b pr	+	46403204	46405387
NM_002391	MDK	midkine isoform a pr	+	46403204	46405387
NM_003934	FUBP3	far upstream element	+	133454959	133513739
NM_030792	GDPD5	glycerophosphodieste	-	75145684	75236599
NM_001172218	SCMH1	polycomb protein SC	-	41492870	41707815
NM_001172219	SCMH1	polycomb protein SC	-	41492870	41707815
NM_001172220	SCMH1	polycomb protein SC	-	41492870	41707815
NM_012236	SCMH1	polycomb protein SC	-	41492870	41707815
ENST00000527860	RAD51	RAD51 homolog (S.	+	40986971	41011084

TGGTGAAACATGAAGAAAATGG

NM_152783	D2HGDH	D-2-hydroxyglutarate	+	242674029	242708231
NM_001122770	ZBTB37	zinc finger and BTB	+	173837492	173855774
NM_032522	ZBTB37	zinc finger and BTB	+	173837492	173842778
NM_000694	ALDH3B1	aldehyde dehydrogen	+	67777789	67796743
NM_001030010	ALDH3B1	aldehyde dehydrogen	+	67777789	67796743
NM_001161473	ALDH3B1	aldehyde dehydrogen	+	67776047	67796743
ENST00000549893	C12orf75	chromosome 12 oper	+	105724487	105789875
NM_003345	UBE2I	SUMO-conjugating e	+	1359627	1377019
NM_194259	UBE2I	SUMO-conjugating e	+	1359627	1377019
NM_194260	UBE2I	SUMO-conjugating e	+	1359153	1377019

346.266703	107.138255	8.035894	6.972193	353.86102	378.95926
346.266703	107.138255	8.035894	6.972193	353.86102	378.95926
343.710677	89.624598	8.026478	6.818752	272.44348	273.00272
124.647425	34.772559	6.553799	5.276755	200.4598	140.40388
6760.4677	845.605998	11.955957	9.771814	3535.7131	2990.9255
58.695334	11.738331	5.233151	3.796928	53.379612	13.520318
192.135802	43.318886	6.882382	5.722468	117.77188	58.20801
39.11296	8.517532	4.658474	2.904562	50.783867	21.73978
39.11296	8.517532	4.658474	2.904562	50.783867	21.73978
28.223901	10.746576	4.438757	3.40854	47.300552	27.053778
321.68663	67.67886	7.907279	6.185352	254.23315	192.3494
1419.131975	299.382986	9.885963	8.447628	888.9171	580.43445
48.088594	6.374432	4.652667	2.7737	64.30769	118.01172
18.615582	6.165261	3.828326	2.644731	22.141197	29.683802
18.615582	6.165261	3.828326	2.644731	22.141197	29.683802
46.731851	12.962867	4.994591	3.493068	74.18125	68.12562
346.843452	19.707601	7.080284	4.155871	554.6668	24.897516
230.33664	51.419437	7.26662	6.018507	276.34976	455.05133
230.33664	51.419437	7.26662	6.018507	276.34976	455.05133
230.33664	51.419437	7.26662	6.018507	276.34976	455.05133
51.794021	11.876868	5.097587	3.599104	32.775726	15.676092
51.794021	11.876868	5.097587	3.599104	32.775726	15.676092
15176.741	3812.989267	13.361293	12.279852	15972.216	6778.487
36.006793	10.429454	4.627346	3.568218	17.99864	15.949097
30.516561	7.656189	4.348697	3.091836	29.340395	59.275566
1666.102733	443.13812	10.157724	9.061478	1215.9543	1377.4307
100.231548	22.721382	6.206792	4.705983	110.627594	148.63329
34.87764	6.867285	4.791788	2.960567	40.59864	36.441055
28.29421	5.69285	4.398568	2.524112	25.424408	36.076214
3391.771823	650.158058	11.097978	8.660864	3356.8394	762.23944
154.003608	45.1063	6.49049	5.023989	221.89519	231.95541
154.003608	45.1063	6.49049	5.023989	221.89519	231.95541
154.003608	45.1063	6.49049	5.023989	221.89519	231.95541
154.003608	45.1063	6.49049	5.023989	221.89519	231.95541
154.003608	45.1063	6.49049	5.023989	221.89519	231.95541
154.003608	45.1063	6.49049	5.023989	221.89519	231.95541
1102.145905	191.87626	9.346265	7.804606	600.77716	424.59378
54.467108	5	5.389787	2.363479	56.711884	45.265575
86.611943	15.95745	6.022029	4.274763	73.9053	40.488808
186.309886	58.736099	5.454415	4.22056	15.978388	30.879232
186.309886	58.736099	5.454415	4.22056	15.978388	30.879232
186.309886	58.736099	5.454415	4.22056	15.978388	30.879232
186.309886	58.736099	5.454415	4.22056	15.978388	30.879232
118.704667	17.255534	5.985529	4.05422	182.45856	29.165442
3704.687667	1086.905003	11.337313	10.076455	4072.6567	5112.2383
1337.066262	306.362212	9.705023	8.414389	1703.1927	2662.9014
69.866203	14.573725	5.702136	4.106474	66.64668	96.73173
1405.994967	314.273338	9.986417	8.648924	1784.2507	1908.652
305.436261	31.938817	7.763768	5.198418	262.06418	417.0382
44.52916	13.090026	5.032959	3.976563	60.281357	46.448742
8033.8631	1988.525683	12.511582	11.367883	8203.098	5295.7764
186.205738	50.62065	7.130663	5.951172	220.29279	291.27023
186.205738	50.62065	7.130663	5.951172	220.29279	291.27023
183.807701	49.53617	7.007604	5.863116	147.701	178.84517
183.807701	49.53617	7.007604	5.863116	147.701	178.84517
183.807701	49.53617	7.007604	5.863116	147.701	178.84517
29.17438	10.577439	4.500821	3.390933	30.172066	40.24891
340.835782	103.868676	7.982102	6.747008	239.02365	217.84044
30.315091	6.427552	4.590171	2.888222	31.27622	34.514767
53.002679	14.808531	5.379473	4.180414	57.191807	56.350597
53.002679	14.808531	5.379473	4.180414	57.191807	56.350597
53.002679	14.808531	5.379473	4.180414	57.191807	56.350597

304.79907	462.5309	227.56334	349.88663	122.3487	66.4578
304.79907	462.5309	227.56334	349.88663	122.3487	66.4578
311.84042	462.99313	311.35226	430.63205	85.85692	74.23825
96.64033	90.52086	120.5216	99.33808	27.953266	16.52167
3851.9216	16633.463	3098.358	10452.425	1065.393	226.18578
103.488525	23.643805	87.3709	70.768845	7.839989	9.874224
130.59367	91.86561	419.0184	335.35724	32.64027	26.228584
72.75005	6.1959777	44.554344	38.653744	5.150827	4.9999995
72.75005	6.1959777	44.554344	38.653744	5.150827	4.9999995
32.214436	24.024157	18.883062	19.86742	10.788859	4.9999995
283.84024	403.61087	336.62967	459.45645	54.58209	17.095383
1082.345	2657.6572	1106.4357	2199.0024	271.7451	94.787735
12.702939	61.302433	5.5991173	26.607662	4.9999995	10.275211
15.059778	13.29531	18.60628	12.907128	4.9999995	4.9999995
15.059778	13.29531	18.60628	12.907128	4.9999995	4.9999995
37.322998	14.00723	53.73545	33.018555	7.7956247	5.8980513
1033.1859	279.44748	38.107327	150.75569	29.271082	16.05737
101.92033	274.99924	89.79452	183.90466	51.87742	45.147312
101.92033	274.99924	89.79452	183.90466	51.87742	45.147312
101.92033	274.99924	89.79452	183.90466	51.87742	45.147312
56.267315	110.67873	22.828913	72.53735	11.736539	4.9999995
56.267315	110.67873	22.828913	72.53735	11.736539	4.9999995
19791.803	9326.016	20274.287	18917.637	4427.127	3360.738
23.018381	32.658855	69.36989	57.045895	5.9888844	7.390107
9.555431	45.595646	12.8335085	26.49882	6.6870766	9.859689
860.0778	3095.4407	1108.0386	2339.6743	437.05942	216.31766
43.562286	94.970146	85.286194	118.30978	23.897524	14.772349
38.41688	39.34754	23.878378	30.583344	4.9999995	7.6180043
20.294289	46.93649	11.829129	29.204731	4.9999995	4.9999995
6131.2173	3144.7168	3059.594	3896.024	974.16504	45.89544
178.14412	15.372448	148.71048	127.944	69.15253	9.391938
178.14412	15.372448	148.71048	127.944	69.15253	9.391938
178.14412	15.372448	148.71048	127.944	69.15253	9.391938
178.14412	15.372448	148.71048	127.944	69.15253	9.391938
178.14412	15.372448	148.71048	127.944	69.15253	9.391938
178.14412	15.372448	148.71048	127.944	69.15253	9.391938
697.89954	2795.21	470.14835	1624.2466	231.95319	88.91058
46.2219	39.5209	59.183147	79.89924	4.9999995	4.9999995
89.97916	134.26768	75.73731	105.2934	15.499039	20.588722
10.187419	24.658937	617.5644	418.59094	6.0977483	4.9999995
10.187419	24.658937	617.5644	418.59094	6.0977483	4.9999995
10.187419	24.658937	617.5644	418.59094	6.0977483	4.9999995
10.187419	24.658937	617.5644	418.59094	6.0977483	4.9999995
323.67365	98.83592	25.169573	52.924854	26.323046	16.344238
2466.5962	1729.4514	4913.275	3933.9084	970.54755	271.38785
607.151	380.29227	1492.8265	1176.0337	383.4048	171.7509
32.35147	77.26741	58.97022	87.22971	13.18324	10.208008
1359.038	705.9148	1326.3328	1351.7815	284.89874	213.09543
106.922935	395.41687	294.86383	356.31155	40.476555	21.379118
33.46286	24.608637	71.91247	30.460896	16.532475	10.210861
11036.168	7283.3726	7372.5796	9012.184	1872.076	1816.7577
144.84769	142.94762	136.67838	181.19772	45.16879	45.73275
144.84769	142.94762	136.67838	181.19772	45.16879	45.73275
88.852066	101.23329	312.8788	273.33588	33.849525	34.17237
88.852066	101.23329	312.8788	273.33588	33.849525	34.17237
88.852066	101.23329	312.8788	273.33588	33.849525	34.17237
22.923773	38.843315	17.761559	25.096655	7.517471	4.9999995
282.16162	400.34915	415.29736	490.34247	100.51733	24.975584
21.969374	38.46149	25.79797	29.870728	4.9999995	5.5741963
42.607723	47.43941	52.499905	61.92663	15.479751	15.680587
42.607723	47.43941	52.499905	61.92663	15.479751	15.680587
42.607723	47.43941	52.499905	61.92663	15.479751	15.680587

192.42099	69.36629	118.42542	73.81033	8.011523	8.273411
192.42099	69.36629	118.42542	73.81033	8.011523	8.273411
88.49907	123.73177	80.65391	84.76767	7.6315513	7.8267465
73.976654	30.62848	25.131899	34.423386	7.195143	6.9045844
2081.254	499.4565	699.14166	502.20505	11.310052	11.124279
11.671291	13.59491	17.501278	9.948296	5.2715163	3.7964973
44.89012	61.786823	51.86175	42.50577	6.428829	5.7064657
22.42696	8.527407	4.9999995	4.9999995	5.199905	4.40574
22.42696	8.527407	4.9999995	4.9999995	5.199905	4.40574
23.012903	5.8083587	11.02706	8.842278	5.1058517	4.7052555
98.04707	120.191505	44.001587	72.155525	7.533695	7.34399
485.9145	281.6888	402.3711	259.79068	9.327154	8.871387
4.9999995	7.9713836	4.9999995	4.9999995	5.548351	6.659801
6.5003886	10.491181	4.9999995	4.9999995	4.0054483	4.827268
6.5003886	10.491181	4.9999995	4.9999995	4.0054483	4.827268
24.129383	4.9999995	29.954142	4.9999995	5.7578397	5.9179115
47.460957	11.3426285	4.9999995	9.113569	8.633799	4.593457
58.45848	56.75231	50.579704	45.701397	7.6581407	8.522633
58.45848	56.75231	50.579704	45.701397	7.6581407	8.522633
58.45848	56.75231	50.579704	45.701397	7.6581407	8.522633
19.200012	6.6084275	15.845809	12.87042	4.5737348	3.9892693
19.200012	6.6084275	15.845809	12.87042	4.5737348	3.9892693
5499.8867	3998.6775	2689.835	2901.6714	13.498958	12.26291
9.099886	17.311638	14.923919	7.862289	3.7005663	4.014574
4.9999995	12.37547	4.9999995	7.0149016	4.41924	5.727525
643.4007	295.65274	665.7997	400.5985	9.775087	10.059771
38.467606	16.49959	28.201694	14.4895315	6.34217	6.9815273
9.326692	8.548959	5.710058	4.9999995	4.8798804	5.0948715
9.157105	4.9999995	4.9999995	4.9999995	4.210272	5.082851
2141.655	109.27738	415.42917	214.52632	11.228771	9.241855
135.67657	9.458891	33.30753	13.650342	7.344956	7.599537
135.67657	9.458891	33.30753	13.650342	7.344956	7.599537
135.67657	9.458891	33.30753	13.650342	7.344956	7.599537
135.67657	9.458891	33.30753	13.650342	7.344956	7.599537
135.67657	9.458891	33.30753	13.650342	7.344956	7.599537
135.67657	9.458891	33.30753	13.650342	7.344956	7.599537
360.19867	144.79207	185.2152	140.18785	8.755931	8.422825
4.9999995	4.9999995	4.9999995	4.9999995	5.364252	5.378251
17.103006	18.176012	10.63842	13.739499	5.7517214	5.2322044
4.9999995	5.0066395	230.72392	100.58829	3.524983	4.8820934
4.9999995	5.0066395	230.72392	100.58829	3.524983	4.8820934
4.9999995	5.0066395	230.72392	100.58829	3.524983	4.8820934
4.9999995	5.0066395	230.72392	100.58829	3.524983	4.8820934
37.528175	12.111379	5.597945	5.628423	7.0534673	4.803363
3063.0037	583.32117	1018.3271	614.84265	11.501801	11.8569565
674.3337	169.65721	264.2941	174.73256	10.264538	10.955659
23.310534	15.80126	11.871569	13.0677395	5.599554	6.3789563
391.62833	312.07056	391.99377	291.9532	10.317698	10.484809
56.202408	30.868906	23.92097	18.784945	7.57663	8.397927
13.026451	13.14024	15.997768	9.632362	5.4540415	5.412939
1979.2458	2045.4551	2384.0444	1833.5751	12.5049	11.911033
32.82399	49.166138	79.791725	51.040504	7.334412	7.9157505
32.82399	49.166138	79.791725	51.040504	7.334412	7.9157505
50.66184	37.808826	92.42743	48.297028	6.7508183	7.2410746
50.66184	37.808826	92.42743	48.297028	6.7508183	7.2410746
50.66184	37.808826	92.42743	48.297028	6.7508183	7.2410746
14.282123	20.830042	10.459549	5.3754506	4.4596076	5.223886
152.93567	50.944874	191.2597	102.578896	7.4483156	7.5088086
4.9999995	5.7033973	8.12804	9.15968	4.5105553	5.02689
17.336758	15.493008	12.868431	11.99265	5.376331	5.6640077
17.336758	15.493008	12.868431	11.99265	5.376331	5.6640077
17.336758	15.493008	12.868431	11.99265	5.376331	5.6640077

7.916737	8.538454	7.5851855	7.890055	7.1670766	6.909728
7.916737	8.538454	7.5851855	7.890055	7.1670766	6.909728
7.948268	8.539523	8.03266	8.1801195	6.652263	7.05838
6.270905	6.147895	6.6913843	6.1128807	5.041163	4.8613305
11.548703	13.700135	11.304102	12.748472	10.359538	8.676701
6.37757	4.074767	6.2432046	5.6353493	3.1669352	4.0853806
6.7071934	6.167836	8.449653	7.8343163	5.259838	5.5485444
5.8713107	2.3547673	5.317281	4.8018427	2.5790408	2.4279919
5.8713107	2.3547673	5.317281	4.8018427	2.5790408	2.4279919
4.696985	4.1003222	4.1440926	3.8800344	3.65762	2.4279919
7.8128533	8.342337	8.141571	8.26923	6.0056114	4.9143815
9.708847	11.04531	9.855436	10.507644	8.348115	7.422022
3.3363662	5.5612903	2.5341594	4.2760344	2.3467326	4.1450634
3.5868278	3.1390417	4.1257625	3.2856052	2.3467326	2.4279919
3.5868278	3.1390417	4.1257625	3.2856052	2.3467326	2.4279919
4.907662	3.2298486	5.563854	4.5904274	3.1604223	3.3055475
9.637011	7.8088465	5.0987654	6.7098274	5.101483	4.823029
6.3549604	7.7867117	6.280999	6.996277	5.931111	6.3430276
6.3549604	7.7867117	6.280999	6.996277	5.931111	6.3430276
6.3549604	7.7867117	6.280999	6.996277	5.931111	6.3430276
5.4985404	6.447755	4.403946	5.6722775	3.7833061	2.4279919
5.4985404	6.447755	4.403946	5.6722775	3.7833061	2.4279919
14.025061	12.826483	13.979705	13.574642	12.427867	12.627403
4.2135506	4.586977	5.9131503	5.335258	2.7844732	3.6458886
2.9478207	5.105668	3.6215298	4.270399	2.9366338	4.0831256
9.394972	11.263188	9.856511	10.596813	9.037142	8.615323
5.1215997	6.218216	6.2097416	6.367498	4.8217707	4.6949077
4.9492784	4.87635	4.467674	4.482672	2.3467326	3.6890357
4.0234346	5.151302	3.5095649	4.413984	2.3467326	2.4279919
12.22285	11.2831745	11.286409	11.324808	10.244089	6.36659
7.149307	3.3805084	6.995343	6.4732866	6.3448944	4.007168
7.149307	3.3805084	6.995343	6.4732866	6.3448944	4.007168
7.149307	3.3805084	6.995343	6.4732866	6.3448944	4.007168
7.149307	3.3805084	6.995343	6.4732866	6.3448944	4.007168
7.149307	3.3805084	6.995343	6.4732866	6.3448944	4.007168
7.149307	3.3805084	6.995343	6.4732866	6.3448944	4.007168
9.087545	11.116988	8.618181	10.076117	8.110966	7.322574
5.2087345	4.882836	5.6976986	5.806952	2.3467326	2.4279919
6.172302	6.739757	6.038169	6.198021	4.1863685	5.1956816
3.033061	4.1422615	9.004969	8.139122	2.8110275	2.4279919
3.033061	4.1422615	9.004969	8.139122	2.8110275	2.4279919
3.033061	4.1422615	9.004969	8.139122	2.8110275	2.4279919
3.033061	4.1422615	9.004969	8.139122	2.8110275	2.4279919
8.002997	6.2765837	4.539805	5.2369585	4.9601407	4.846613
10.923324	10.442846	11.959332	11.339621	10.239256	8.951307
8.87462	8.2481365	10.27154	9.615644	8.843277	8.268785
4.704607	5.909313	5.6930423	5.9273405	3.947702	4.134368
10.047306	9.155938	10.09799	9.814762	8.4162855	8.59319
6.42191	8.309002	7.9627295	7.914412	5.574485	5.249404
4.7517548	4.138937	5.964012	4.4760723	4.2790318	4.1348953
13.104774	12.481129	12.532224	12.535432	11.182822	11.71647
6.8536906	6.835222	6.8698378	6.9750648	5.727311	6.3622093
6.8536906	6.835222	6.8698378	6.9750648	5.727311	6.3622093
6.155646	6.3121314	8.039865	7.5460916	5.311153	5.9355555
6.155646	6.3121314	8.039865	7.5460916	5.311153	5.9355555
6.155646	6.3121314	8.039865	7.5460916	5.311153	5.9355555
4.207841	4.855729	4.062228	4.1956325	3.1045434	2.4279919
7.8034234	8.330698	8.438243	8.363123	6.8869505	5.472029
4.1435394	4.8383203	4.5726643	4.4490542	2.3467326	3.215198
5.090735	5.167788	5.5333614	5.444613	4.1845884	4.788298
5.090735	5.167788	5.5333614	5.444613	4.1845884	4.788298
5.090735	5.167788	5.5333614	5.444613	4.1845884	4.788298

7.5374146	6.4260893	7.1370745	6.6557727
7.5374146	6.4260893	7.1370745	6.6557727
6.43744	7.316149	6.591478	6.856805
6.1821127	5.147186	4.922719	5.5060205
10.972676	9.414182	9.680368	9.52742
3.6388113	3.8882163	4.4018717	3.6003551
5.4862375	6.2474074	5.9668646	5.825919
4.519209	3.186564	2.3618152	2.352752
4.519209	3.186564	2.3618152	2.352752
4.5556087	2.6497142	3.7381823	3.422123
6.5748835	7.264973	5.728891	6.6233706
8.880659	8.577859	8.896685	8.560425
2.350709	3.085128	2.3618152	2.352752
2.8833127	3.4957795	2.3618152	2.352752
2.8833127	3.4957795	2.3618152	2.352752
4.617343	2.3408759	5.1814675	2.352752
5.5663614	3.6138785	2.3618152	3.4686584
5.856167	6.113735	5.9314475	5.9355555
5.856167	6.113735	5.9314475	5.9355555
5.856167	6.113735	5.9314475	5.9355555
4.31089	2.823288	4.2569375	3.9922087
4.31089	2.823288	4.2569375	3.9922087
12.391281	12.491043	11.642957	12.098563
3.3045406	4.256682	4.1721888	3.2455347
2.350709	3.7457561	2.3618152	3.0729787
9.272681	8.654312	9.606584	9.182827
5.268574	4.1867805	5.092321	4.1715426
3.3397624	3.1904263	2.844695	2.352752
3.3145049	2.3408759	2.3618152	2.352752
11.023864	7.1154013	8.941606	8.273631
7.0405917	3.3380566	5.33018	4.083042
7.0405917	3.3380566	5.33018	4.083042
7.0405917	3.3380566	5.33018	4.083042
7.0405917	3.3380566	5.33018	4.083042
7.0405917	3.3380566	5.33018	4.083042
7.0405917	3.3380566	5.33018	4.083042
8.437454	7.5509963	7.78195	7.623695
2.350709	2.3408759	2.3618152	2.352752
4.1503015	4.328668	3.6924956	4.09506
2.350709	2.5112703	8.106773	7.1155906
2.350709	2.5112703	8.106773	7.1155906
2.350709	2.5112703	8.106773	7.1155906
2.350709	2.5112703	8.106773	7.1155906
5.232718	3.713131	2.8187363	2.7539785
11.543254	9.653299	10.251916	9.819699
9.342271	7.7793503	8.299912	7.9527364
4.57357	4.122824	3.8444438	4.015938
8.565757	8.738177	8.854871	8.725263
5.8019886	5.1575456	4.8491797	4.5579066
3.7814782	3.836934	4.2733855	3.5536509
10.890528	11.498745	11.473399	11.445333
5.049529	5.8878803	6.577146	6.1029553
5.049529	5.8878803	6.577146	6.1029553
5.657776	5.473324	6.7811813	6.0197062
5.657776	5.473324	6.7811813	6.0197062
5.657776	5.473324	6.7811813	6.0197062
3.9051957	4.545908	3.668214	2.6937456
7.2052975	5.9441776	7.8291187	7.1444726
2.350709	2.6249855	3.3150547	3.476653
4.1675897	4.0945215	3.9621627	3.8853211
4.1675897	4.0945215	3.9621627	3.8853211
4.1675897	4.0945215	3.9621627	3.8853211

ASHGA5P048981	0.000204465	0.01146874	2.2958989	up	noncoding
ASHGA5P021093	0.005574791	0.047417224	2.8424497	up	noncoding
ASHGA5P033391	0.048676541	0.143360998	6.1281088	up	noncoding
ASHGA5P047633	0.030285573	0.109120103	2.6965832	up	noncoding
ASHGA5P045741	0.027033501	0.102614663	3.2588764	up	noncoding
ASHGA5P042632	3.82242E-05	0.006301164	3.3345175	up	noncoding
ASHGA5P027317	0.026203038	0.100833346	8.571684	up	noncoding
ASHGA5P041578	0.00140473	0.025092339	2.1698533	up	noncoding
ASHGA5P056051	0.009997501	0.06206579	2.0646679	up	noncoding
ASHGA5P056051	0.009997501	0.06206579	2.0646679	up	noncoding
ASHGA5P056051	0.009997501	0.06206579	2.0646679	up	noncoding
ASHGA5P056051	0.009997501	0.06206579	2.0646679	up	noncoding
ASHGA5P056051	0.009997501	0.06206579	2.0646679	up	noncoding
ASHGA5P056051	0.009997501	0.06206579	2.0646679	up	noncoding
ASHGA5P056051	0.009997501	0.06206579	2.0646679	up	noncoding
ASHGA5P056051	0.009997501	0.06206579	2.0646679	up	noncoding
ASHGA5P056051	0.009997501	0.06206579	2.0646679	up	noncoding
ASHGA5P056051	0.009997501	0.06206579	2.0646679	up	noncoding
ASHGA5P057819	0.042794127	0.132584608	2.5138637	up	noncoding
ASHGA5P020318	0.003953378	0.040405952	2.1904876	up	noncoding
ASHGA5P041951	0.001898031	0.029176753	3.3717891	up	noncoding
ASHGA5P051875	0.035108278	0.118596641	2.0809214	up	noncoding
ASHGA5P014885	0.000194962	0.01146874	2.4539935	up	noncoding
ASHGA5P014885	0.000194962	0.01146874	2.4539935	up	noncoding
ASHGA5P014885	0.000194962	0.01146874	2.4539935	up	noncoding
ASHGA5P020158	0.040841188	0.129117831	2.3478234	up	noncoding
ASHGA5P020158	0.040841188	0.129117831	2.3478234	up	noncoding
ASHGA5P017564	0.003800588	0.039493479	2.3001728	up	noncoding
ASHGA5P017564	0.003800588	0.039493479	2.3001728	up	noncoding
ASHGA5P017564	0.003800588	0.039493479	2.3001728	up	noncoding
ASHGA5P017564	0.003800588	0.039493479	2.3001728	up	noncoding
ASHGA5P017564	0.003800588	0.039493479	2.3001728	up	noncoding
ASHGA5P020443	0.003054512	0.035781836	3.3201845	up	noncoding
ASHGA5P029901	0.000141747	0.01066938	22.3343717	up	noncoding
ASHGA5P042342	0.002459793	0.032230552	2.3726938	up	noncoding
ASHGA5P032716	5.16342E-05	0.007392592	2.5753693	up	noncoding
ASHGA5P032732	0.000162001	0.011182026	3.6858791	up	noncoding
ASHGA5P017959	0.003596311	0.038615726	2.0361312	up	noncoding
ASHGA5P017959	0.003596311	0.038615726	2.0361312	up	noncoding
ASHGA5P017959	0.003596311	0.038615726	2.0361312	up	noncoding
ASHGA5P038130	0.032935545	0.114267657	4.0639157	up	noncoding
ASHGA5P038130	0.032935545	0.114267657	4.0639157	up	noncoding
ASHGA5P045175	0.046038937	0.138218388	2.4021598	up	noncoding
ASHGA5P053393	0.000537448	0.015963419	2.897849	up	noncoding
ASHGA5P038627	0.016373796	0.078125296	3.2426947	up	noncoding
ASHGA5P038411	0.010899394	0.064586652	2.8580065	up	noncoding
ASHGA5P021484	0.000881467	0.02004903	3.0887676	up	noncoding
ASHGA5P021484	0.000881467	0.02004903	3.0887676	up	noncoding
ASHGA5P021484	0.000881467	0.02004903	3.0887676	up	noncoding
ASHGA5P032711	0.027260811	0.103003137	9.0804344	up	noncoding
ASHGA5P043447	0.005807882	0.048450721	2.0771654	up	noncoding
ASHGA5P043447	0.005807882	0.048450721	2.0771654	up	noncoding
ASHGA5P043447	0.005807882	0.048450721	2.0771654	up	noncoding
ASHGA5P027219	0.041482069	0.130029045	2.3979538	up	noncoding
ASHGA5P036948	0.003065967	0.035804635	2.7464213	up	noncoding
ASHGA5P036948	0.003065967	0.035804635	2.7464213	up	noncoding
ASHGA5P025986	0.00043508	0.014669266	3.2007553	up	noncoding
ASHGA5P025986	0.00043508	0.014669266	3.2007553	up	noncoding
ASHGA5P025986	0.00043508	0.014669266	3.2007553	up	noncoding
ASHGA5P025986	0.00043508	0.014669266	3.2007553	up	noncoding
ASHGA5P040852	0.000437307	0.014669266	3.3576113	up	noncoding
ASHGA5P040852	0.000437307	0.014669266	3.3576113	up	noncoding

ENST00000568106	LA16c-358B7.3	GENCODE	3107	chr16	-
ENST00000524962	RP11-680F20.6	GENCODE	507	chr11	+
NR_040050	LOC100507351	RefSeq	4232	chr17	+
ENST00000443919	RP11-316M21.7	GENCODE	578	chr10	+
ENST00000439342	RP11-318K12.2	GENCODE	305	chr9	+
uc003qdy.1	SNORA33	UCSC_knowngene	2045	chr6	+
ENST00000512916	HOXC-AS5	GENCODE	1408	chr12	-
ENST00000358442	RP11-423H2.1	GENCODE	515	chr5	+
ENST00000445805	RP4-594I10.2	GENCODE	420	chr1	-
ENST00000445805	RP4-594I10.2	GENCODE	420	chr1	-
ENST00000445805	RP4-594I10.2	GENCODE	420	chr1	-
ENST00000445805	RP4-594I10.2	GENCODE	420	chr1	-
ENST00000445805	RP4-594I10.2	GENCODE	420	chr1	-
ENST00000445805	RP4-594I10.2	GENCODE	420	chr1	-
ENST00000445805	RP4-594I10.2	GENCODE	420	chr1	-
ENST00000445805	RP4-594I10.2	GENCODE	420	chr1	-
ENST00000445805	RP4-594I10.2	GENCODE	420	chr1	-
ENST00000445805	RP4-594I10.2	GENCODE	420	chr1	-
TCONS_00022189	XLOC_010411	LincRNAs identified	259	chr13	+
ENST00000511488	FAM86EP	GENCODE	1209	chr4	-
ENST00000564541	RP13-44L19.2	GENCODE	2268	chr6	-
uc003ggd.1	C4orf10	UCSC_knowngene	2766	chr4	+
NR_047677	TARS	RefSeq	2714	chr5	+
NR_047677	TARS	RefSeq	2714	chr5	+
NR_047677	TARS	RefSeq	2714	chr5	+
ENST00000508878	CTC-228N24.3	GENCODE	968	chr5	-
ENST00000508878	CTC-228N24.3	GENCODE	968	chr5	-
ENST00000443162	AC004854.4	GENCODE	188	chr7	+
ENST00000443162	AC004854.4	GENCODE	188	chr7	+
ENST00000443162	AC004854.4	GENCODE	188	chr7	+
ENST00000443162	AC004854.4	GENCODE	188	chr7	+
ENST00000443162	AC004854.4	GENCODE	188	chr7	+
ENST00000513652	RP11-472B18.1	GENCODE	1532	chr4	+
BX104325		LincRNAs identified	451	chr1	-
ENST00000440874	TRIM31-AS1	GENCODE	549	chr6	+
ENST00000582866	RP11-498C9.15	GENCODE	2682	chr17	-
ENST00000574080	RP11-388C12.2	GENCODE	2450	chr17	-
ENST00000447623	RP11-263K19.4	GENCODE	541	chr1	+
ENST00000447623	RP11-263K19.4	GENCODE	541	chr1	+
ENST00000447623	RP11-263K19.4	GENCODE	541	chr1	+
ENST00000414844	PLCL2-AS1	GENCODE	277	chr3	-
ENST00000414844	PLCL2-AS1	GENCODE	277	chr3	-
ENST00000441360	RP4-781K5.5	GENCODE	933	chr1	+
ENST00000434796	GAS5	GENCODE	575	chr1	-
ENST00000418353	RP11-135A1.2	GENCODE	430	chr3	-
uc003emd.1	RPL32P3	UCSC_knowngene	2836	chr3	-
ENST00000533253	CTD-2523D13.2	GENCODE	523	chr11	+
ENST00000533253	CTD-2523D13.2	GENCODE	523	chr11	+
ENST00000533253	CTD-2523D13.2	GENCODE	523	chr11	+
ENST00000438158	RP11-400N13.3	GENCODE	562	chr1	-
ENST00000430825	AC005534.9	GENCODE	514	chr7	-
ENST00000430825	AC005534.9	GENCODE	514	chr7	-
ENST00000430825	AC005534.9	GENCODE	514	chr7	-
ENST00000552634	RP11-474P2.4	GENCODE	562	chr12	-
ENST00000451443	PLCB1-IT1	GENCODE	251	chr20	+
ENST00000451443	PLCB1-IT1	GENCODE	251	chr20	+
NR_047691	PIGT	RefSeq	2206	chr20	+
NR_047691	PIGT	RefSeq	2206	chr20	+
NR_047691	PIGT	RefSeq	2206	chr20	+
NR_047691	PIGT	RefSeq	2206	chr20	+
uc003lzi.3	BC035392	UCSC_knowngene	1269	chr5	-
uc003lzi.3	BC035392	UCSC_knowngene	1269	chr5	-

1369255	1372828	ENST00000567829	CATTTAGAAGTTCCTGTATTCCTAA		natural antisense
125821760	125825832		ACCTCCAGCTGGGATATCCAGCTCC		natural antisense
75543022	75561103		ATCTCCTTCCCAC	100507351	intergenic
101949792	101950504		TATATCCCTGAGTAACGGCAGATAA		intronic antisense
66742303	66759357	ENST00000446288,	AGGTTACTTGTTTATTAGTAGTAGT		intergenic
133136445	133138490		GGGATGAAGGTT	594839	exon sense-overlappi
54329111	54333427		GATACCCAGCTC	100874366	natural antisense
177309443	177348388		ATCAAAACAACCTCATTGTTCTCAA		intergenic
24863137	24865371		GACTATATTCTTAAAGAATAAAAGT		natural antisense
24863137	24865371		GACTATATTCTTAAAGAATAAAAGT		natural antisense
24863137	24865371		GACTATATTCTTAAAGAATAAAAGT		natural antisense
24863137	24865371		GACTATATTCTTAAAGAATAAAAGT		natural antisense
24863137	24865371		GACTATATTCTTAAAGAATAAAAGT		natural antisense
24863137	24865371		GACTATATTCTTAAAGAATAAAAGT		natural antisense
24863137	24865371		GACTATATTCTTAAAGAATAAAAGT		natural antisense
24863137	24865371		GACTATATTCTTAAAGAATAAAAGT		natural antisense
24863137	24865371		GACTATATTCTTAAAGAATAAAAGT		natural antisense
24863137	24865371		GACTATATTCTTAAAGAATAAAAGT		natural antisense
64201007	64209643	TCONS_00021811, '	CTTTAATCTGAGAATCTTACGTCTT		intergenic
3944493	3957121	NR_024253	GCAGAGGCCGGCCAGGCCAGGATC		intergenic
96023058	96025326		ATTTATTGAGTATCTTTCATTATATT		bidirectional
2936625	2943596	ENST00000505731	CGCCGGTCCTGC	317648	natural antisense
33440881	33468196		GTGGAATTAGCC	6897	exon sense-overlappi
33440881	33468196		GTGGAATTAGCC	6897	exon sense-overlappi
33440881	33468196		GTGGAATTAGCC	6897	exon sense-overlappi
127359718	127418652		TTGGCAGAACTGTTTGAAAATGCTA		bidirectional
127359718	127418652		TTGGCAGAACTGTTTGAAAATGCTA		bidirectional
44888014	44889167		GTTTGGGGAGGTTAAAATTTGGAG		bidirectional
44888014	44889167		GTTTGGGGAGGTTAAAATTTGGAG		bidirectional
44888014	44889167		GTTTGGGGAGGTTAAAATTTGGAG		bidirectional
44888014	44889167		GTTTGGGGAGGTTAAAATTTGGAG		bidirectional
44888014	44889167		GTTTGGGGAGGTTAAAATTTGGAG		bidirectional
39481874	39483525		AGAGTGGCTGATGACAAAACCTTG		intergenic
109073813	109074249		TGAACCTGGAACTGCTTATTCCTTT		intergenic
30073016	30082501		TAGAGACACTACTAATTGTTCCAGC		natural antisense
79836300	79838982		GCCTAAGGTAGGCCAGAGTTAATTT		intergenic
80631872	80634364		GTTGTTGCGTTTTCTAGTTTGTCCA		intron sense-overlapp
155166658	155170137		AGTCCCACAGGCTTACCATCTGAGT		natural antisense
155166658	155170137		AGTCCCACAGGCTTACCATCTGAGT		natural antisense
155166658	155170137		AGTCCCACAGGCTTACCATCTGAGT		natural antisense
17084267	17085684		TATTATGGTCAGTACCTGAAAGCTC		natural antisense
17084267	17085684		TATTATGGTCAGTACCTGAAAGCTC		natural antisense
234796016	234802850		AGTACCATTTGCAAATGGCTCTTGC		intergenic
173833117	173834039	ENST00000422183,	CTGATATTCTGC	60674	intergenic
193848404	193848990		CCCTGAAATGGAATTTCAACGTGTG		intergenic
129109997	129118282		GAGTTAAGAGCA	132241	intergenic
119600292	119610333		AACAACGTGGGTTAATATTAATGCC		bidirectional
119600292	119610333		AACAACGTGGGTTAATATTAATGCC		bidirectional
119600292	119610333		AACAACGTGGGTTAATATTAATGCC		bidirectional
222215046	222238105		TGGTCAGTGCTCAACAAATGCCCCT		intergenic
156461645	156469257		CAGTTCGCATAGACTTTTCATGCAG		natural antisense
156461645	156469257		CAGTTCGCATAGACTTTTCATGCAG		natural antisense
156461645	156469257		CAGTTCGCATAGACTTTTCATGCAG		natural antisense
46778015	46780774		CTAATTTGTTCATACAATGGATATC		intergenic
8229371	8237564		GGCTGAGCTCCTACTAAAAAGAAG		intron sense-overlapp
8229371	8237564		GGCTGAGCTCCTACTAAAAAGAAG		intron sense-overlapp
44044706	44054885		GCCCCATCAGTG	51604	exon sense-overlappi
44044706	44054885		GCCCCATCAGTG	51604	exon sense-overlappi
44044706	44054885		GCCCCATCAGTG	51604	exon sense-overlappi
44044706	44054885		GCCCCATCAGTG	51604	exon sense-overlappi
162909609	162921064		GTTCTCCAGCTTAAGATCTTCTATC		natural antisense
162909609	162921064		GTTCTCCAGCTTAAGATCTTCTATC		natural antisense

NM_194261	UBE2I	SUMO-conjugating e	+	1359153	1377019
ENST00000392693	CDON	Cdon homolog (mous	-	125825690	125933187
NM_001278	CHUK	inhibitor of nuclear fa	-	101948123	101989344
NM_001016	RPS12	40S ribosomal protei	+	133135707	133138703
NM_017410	HOXC13	homeobox protein Ho	+	54332575	54340328
NM_001251977	RCAN3	calcipressin-3 isoforr	+	24829386	24863510
NM_001251978	RCAN3	calcipressin-3 isoforr	+	24834083	24863510
NM_001251979	RCAN3	calcipressin-3 isoforr	+	24828840	24863510
NM_001251980	RCAN3	calcipressin-3 isoforr	+	24840803	24863510
NM_001251981	RCAN3	calcipressin-3 isoforr	+	24829386	24863510
NM_001251982	RCAN3	calcipressin-3 isoforr	+	24840803	24863510
NM_001251983	RCAN3	calcipressin-3 isoforr	+	24829386	24863510
NM_001251984	RCAN3	calcipressin-3 isoforr	+	24828840	24863510
NM_001251985	RCAN3	calcipressin-3 isoforr	+	24840803	24863510
NM_013441	RCAN3	calcipressin-3 isoforr	+	24829386	24863510
NM_024641	MANEA	glycoprotein endo-alj	+	96025372	96057328
NM_003703	NOP14	nucleolar protein 14	-	2939663	2965118
NM_001258437	TARS	threonine--tRNA liga	+	33440801	33468196
NM_001258438	TARS	threonine--tRNA liga	+	33440881	33468196
NM_152295	TARS	threonine--tRNA liga	+	33440881	33468196
NM_001046	SLC12A2	solute carrier family	+	127419482	127525380
NM_001256461	SLC12A2	solute carrier family	+	127419482	127525380
NM_012412	H2AFV	histone H2A.V isofo	-	44873212	44887725
NM_138635	H2AFV	histone H2A.V isofo	-	44866487	44887725
NM_201436	H2AFV	histone H2A.V isofo	-	44873212	44887725
NM_201516	H2AFV	histone H2A.V isofo	-	44873212	44887725
NM_201517	H2AFV	histone H2A.V isofo	-	44873212	44887725
NM_007028	TRIM31	E3 ubiquitin-protein	-	30070673	30080867
NM_006822	RAB40B	ras-related protein Ra	-	80614942	80656598
NM_001252607	THBS3	thrombospondin-3 is	-	155165378	155177772
NM_001252608	THBS3	thrombospondin-3 is	-	155165378	155177772
NM_007112	THBS3	thrombospondin-3 is	-	155165378	155177772
NM_001144382	PLCL2	inactive phospholipa	+	16926451	17132098
NM_015184	PLCL2	inactive phospholipa	+	16974581	17132098
NM_002855	PVRL1	poliovirus receptor-r	-	119531702	119599435
NM_203285	PVRL1	poliovirus receptor-r	-	119508807	119599435
NM_203286	PVRL1	poliovirus receptor-r	-	119545098	119599435
NM_001184996	RNF32	RING finger protein	+	156433440	156469820
NM_001184997	RNF32	RING finger protein	+	156435670	156469820
NM_030936	RNF32	RING finger protein	+	156433352	156469820
NM_015192	PLCB1	1-phosphatidylinosito	+	8113295	8865547
NM_182734	PLCB1	1-phosphatidylinosito	+	8113295	8865547
NM_001184728	PIGT	GPI transamidase coi	+	44044706	44054885
NM_001184729	PIGT	GPI transamidase coi	+	44044706	44054885
NM_001184730	PIGT	GPI transamidase coi	+	44044706	44054885
NM_015937	PIGT	GPI transamidase coi	+	44044706	44054885
NM_001142556	HMMR	hyaluronan mediated	+	162887516	162918953
NM_001142557	HMMR	hyaluronan mediated	+	162887516	162918953

53.002679	14.808531	5.379473	4.180414	57.191807	56.350597
725.149293	166.593357	9.019224	7.512089	760.1759	895.5393
13913.92328	3901.25205	13.015125	10.399683	14799.326	15748.559
25.989447	6.516574	4.123042	2.69191	27.607443	62.43328
57.091145	10.586371	5.153815	3.44944	22.354574	13.399021
4992.828983	831.415178	11.820347	10.082869	4756.1865	5660.7437
12815.84767	2872.390787	13.015047	9.915468	12378.04	14972.114
2951.627117	761.860788	11.050694	9.933096	2712.6838	3740.832
757.592543	222.188453	9.072708	8.026798	863.9332	367.95926
757.592543	222.188453	9.072708	8.026798	863.9332	367.95926
757.592543	222.188453	9.072708	8.026798	863.9332	367.95926
757.592543	222.188453	9.072708	8.026798	863.9332	367.95926
757.592543	222.188453	9.072708	8.026798	863.9332	367.95926
757.592543	222.188453	9.072708	8.026798	863.9332	367.95926
757.592543	222.188453	9.072708	8.026798	863.9332	367.95926
757.592543	222.188453	9.072708	8.026798	863.9332	367.95926
757.592543	222.188453	9.072708	8.026798	863.9332	367.95926
757.592543	222.188453	9.072708	8.026798	863.9332	367.95926
1055.486328	213.684608	9.233861	7.903954	1507.7194	2377.3938
277.071353	81.063309	7.668492	6.53724	259.78964	373.2355
50.230419	11.300688	5.186052	3.432538	45.965942	17.821533
90.87965	28.407851	5.980678	4.923456	120.767075	113.67522
1457.765417	351.515405	10.073516	8.778384	1195.2446	1200.9938
1457.765417	351.515405	10.073516	8.778384	1195.2446	1200.9938
1457.765417	351.515405	10.073516	8.778384	1195.2446	1200.9938
63.934159	23.862327	5.629061	4.397737	72.832	55.042553
63.934159	23.862327	5.629061	4.397737	72.832	55.042553
27.23401	8.157851	4.296176	3.094434	37.716415	19.93985
27.23401	8.157851	4.296176	3.094434	37.716415	19.93985
27.23401	8.157851	4.296176	3.094434	37.716415	19.93985
27.23401	8.157851	4.296176	3.094434	37.716415	19.93985
27.23401	8.157851	4.296176	3.094434	37.716415	19.93985
71.268863	13.860363	5.726076	3.994813	72.265884	105.91966
1814.886967	47.619901	10.139159	5.657965	1363.6049	319.6461
306.414867	89.950876	7.857977	6.611451	335.7221	297.01297
2454.0264	584.172547	10.810767	9.445987	2327.0664	1495.437
45.979578	8.64409	5.033973	3.151964	54.212616	21.427958
47.733701	15.821629	5.232224	4.206394	53.3272	42.421227
47.733701	15.821629	5.232224	4.206394	53.3272	42.421227
47.733701	15.821629	5.232224	4.206394	53.3272	42.421227
4410.473908	716.519497	11.263295	9.240425	5499.054	9106.442
4410.473908	716.519497	11.263295	9.240425	5499.054	9106.442
135.12299	27.515007	6.337948	5.073615	202.77968	60.4639
6904.48915	1283.601707	12.225917	10.690934	5145.8647	5101.1387
2926.842625	481.019348	10.720898	9.023705	1622.7238	902.48645
722.222835	155.78504	8.947381	7.432372	517.9645	473.65863
70.730118	15.44687	5.712767	4.085735	60.785583	47.934345
70.730118	15.44687	5.712767	4.085735	60.785583	47.934345
70.730118	15.44687	5.712767	4.085735	60.785583	47.934345
172.035638	7.038586	6.096683	2.913922	271.10635	6.309858
647.000322	179.903402	8.791574	7.736957	720.7398	434.022
647.000322	179.903402	8.791574	7.736957	720.7398	434.022
647.000322	179.903402	8.791574	7.736957	720.7398	434.022
53.305199	15.227301	5.288143	4.026339	34.086403	63.726273
73.912489	17.697355	5.809137	4.351584	66.95798	65.01002
73.912489	17.697355	5.809137	4.351584	66.95798	65.01002
233.065127	48.240218	7.474265	5.795853	291.14664	293.75064
233.065127	48.240218	7.474265	5.795853	291.14664	293.75064
233.065127	48.240218	7.474265	5.795853	291.14664	293.75064
233.065127	48.240218	7.474265	5.795853	291.14664	293.75064
35.743354	8.217428	4.761431	3.013996	28.848679	30.931328
35.743354	8.217428	4.761431	3.013996	28.848679	30.931328

42.607723	47.43941	52.499905	61.92663	15.479751	15.680587
549.77856	313.6906	997.0619	834.6495	186.89818	84.88416
8069.3203	2372.5144	23510.957	18982.863	4465.2495	381.24875
16.008781	14.471288	23.212759	12.203132	6.7814054	4.9999995
37.755577	58.68411	106.3977	103.95589	4.9999995	10.518751
3685.8757	6359.674	3609.788	5884.706	770.88	695.4192
8738.767	3422.01	21329.447	16054.708	3781.437	309.89276
1538.725	3446.9834	2602.3945	3668.144	686.7355	459.09335
1201.367	862.627	451.20206	798.46674	174.92607	73.91312
1201.367	862.627	451.20206	798.46674	174.92607	73.91312
1201.367	862.627	451.20206	798.46674	174.92607	73.91312
1201.367	862.627	451.20206	798.46674	174.92607	73.91312
1201.367	862.627	451.20206	798.46674	174.92607	73.91312
1201.367	862.627	451.20206	798.46674	174.92607	73.91312
1201.367	862.627	451.20206	798.46674	174.92607	73.91312
1201.367	862.627	451.20206	798.46674	174.92607	73.91312
1201.367	862.627	451.20206	798.46674	174.92607	73.91312
1201.367	862.627	451.20206	798.46674	174.92607	73.91312
394.5876	213.13037	1048.2712	791.8156	246.54926	136.69704
128.58218	343.50894	232.66066	324.6512	49.677906	64.15546
79.76172	73.123764	30.968155	53.7414	11.077641	4.9999995
52.32461	30.941002	121.48569	106.0843	37.701458	22.200912
1197.2769	1312.9236	1853.3809	1986.7727	323.2191	293.51712
1197.2769	1312.9236	1853.3809	1986.7727	323.2191	293.51712
1197.2769	1312.9236	1853.3809	1986.7727	323.2191	293.51712
79.455185	71.66245	36.413544	68.19922	23.6171	4.9999995
79.455185	71.66245	36.413544	68.19922	23.6171	4.9999995
49.29739	26.370785	9.707961	20.37166	10.838692	4.9999995
49.29739	26.370785	9.707961	20.37166	10.838692	4.9999995
49.29739	26.370785	9.707961	20.37166	10.838692	4.9999995
49.29739	26.370785	9.707961	20.37166	10.838692	4.9999995
49.29739	26.370785	9.707961	20.37166	10.838692	4.9999995
31.508762	70.33921	68.45055	79.12911	19.00467	11.923931
2183.7908	3200.6365	1343.9204	2477.7231	25.307428	22.370539
366.4298	179.1341	330.86133	329.3289	88.78903	27.759644
2879.552	2593.8005	2262.6038	3165.6987	587.46747	251.72017
89.123474	55.75417	21.555641	33.803608	11.0762205	4.9999995
33.754818	60.319305	46.963337	49.61632	14.558818	7.5885377
33.754818	60.319305	46.963337	49.61632	14.558818	7.5885377
33.754818	60.319305	46.963337	49.61632	14.558818	7.5885377
1509.6631	765.15485	5487.5503	4094.9792	977.4438	282.93655
1509.6631	765.15485	5487.5503	4094.9792	977.4438	282.93655
351.98676	84.722855	50.542824	60.24192	21.546219	22.774055
5366.2305	12031.494	4632.858	9149.349	972.3495	882.10364
2225.3416	7106.154	1125.8514	4578.4985	541.6649	125.77546
413.58704	1383.3875	474.56784	1070.1715	143.65489	44.92849
74.113754	39.232834	103.44841	98.86578	7.709773	7.7057214
74.113754	39.232834	103.44841	98.86578	7.709773	7.7057214
74.113754	39.232834	103.44841	98.86578	7.709773	7.7057214
506.66623	37.455982	111.09499	99.58042	4.9999995	5.2530656
1000.11224	889.5731	211.92659	625.6282	207.41066	110.82166
1000.11224	889.5731	211.92659	625.6282	207.41066	110.82166
1000.11224	889.5731	211.92659	625.6282	207.41066	110.82166
33.814972	89.876434	30.924183	67.40293	16.60677	5.4488463
44.057205	105.49558	62.33907	99.61508	17.88791	26.805218
44.057205	105.49558	62.33907	99.61508	17.88791	26.805218
231.45479	185.95593	162.33284	233.74992	53.225773	27.388329
231.45479	185.95593	162.33284	233.74992	53.225773	27.388329
231.45479	185.95593	162.33284	233.74992	53.225773	27.388329
231.45479	185.95593	162.33284	233.74992	53.225773	27.388329
21.349968	63.77368	27.395817	42.160652	4.9999995	4.9999995
21.349968	63.77368	27.395817	42.160652	4.9999995	4.9999995

17.336758	15.493008	12.868431	11.99265	5.376331	5.6640077
381.3377	94.47174	147.23903	104.72933	9.1064625	9.46992
17406.717	239.98418	648.7905	265.52237	13.394678	13.479052
12.318039	4.9999995	4.9999995	4.9999995	4.3335614	5.798473
4.9999995	19.225203	6.139235	17.635038	4.020041	3.7846107
851.89197	813.558	1069.1802	787.5617	11.734996	12.005853
12307.131	130.26776	526.2262	179.39	13.140427	13.406784
930.59094	839.25867	905.71967	749.7666	10.919979	11.453988
295.9519	235.83542	323.35202	229.15219	9.291906	8.229831
295.9519	235.83542	323.35202	229.15219	9.291906	8.229831
295.9519	235.83542	323.35202	229.15219	9.291906	8.229831
295.9519	235.83542	323.35202	229.15219	9.291906	8.229831
295.9519	235.83542	323.35202	229.15219	9.291906	8.229831
295.9519	235.83542	323.35202	229.15219	9.291906	8.229831
295.9519	235.83542	323.35202	229.15219	9.291906	8.229831
295.9519	235.83542	323.35202	229.15219	9.291906	8.229831
295.9519	235.83542	323.35202	229.15219	9.291906	8.229831
295.9519	235.83542	323.35202	229.15219	9.291906	8.229831
456.5829	105.32313	214.15892	122.7964	10.0934725	10.785746
31.923	113.297386	133.28667	94.03943	7.563295	8.251866
25.89481	7.760083	12.178781	5.8928156	5.062407	4.1532216
59.515423	12.864538	21.537909	16.626865	6.465655	6.607797
344.9279	236.79865	579.25616	331.3735	9.74544	9.871557
344.9279	236.79865	579.25616	331.3735	9.74544	9.871557
344.9279	236.79865	579.25616	331.3735	9.74544	9.871557
59.961613	17.60582	20.81137	16.178062	5.7290196	5.6347795
59.961613	17.60582	20.81137	16.178062	5.7290196	5.6347795
10.731818	7.5137496	4.9999995	9.86285	4.773218	4.2965393
10.731818	7.5137496	4.9999995	9.86285	4.773218	4.2965393
10.731818	7.5137496	4.9999995	9.86285	4.773218	4.2965393
10.731818	7.5137496	4.9999995	9.86285	4.773218	4.2965393
10.731818	7.5137496	4.9999995	9.86285	4.773218	4.2965393
16.2406	8.698692	19.234833	8.059453	5.716444	6.509448
29.867727	114.99821	30.155855	63.01965	9.947801	8.041696
157.91692	53.7929	131.80232	79.64444	7.9381137	7.9442644
990.66754	443.776	761.7388	469.6653	10.710357	10.167695
16.880672	6.149128	5.76865	6.98987	5.294453	4.386631
19.9792	19.745579	13.09513	19.962507	5.2699757	5.295855
19.9792	19.745579	13.09513	19.962507	5.2699757	5.295855
19.9792	19.745579	13.09513	19.962507	5.2699757	5.295855
2189.1833	186.73819	477.21124	185.6039	11.918303	12.692435
2189.1833	186.73819	477.21124	185.6039	11.918303	12.692435
35.075424	36.306023	23.717775	25.670547	7.210077	5.755888
1062.2369	1578.9879	1768.9764	1436.9559	11.836764	11.854132
1042.2255	345.32373	494.89423	336.23227	10.1974325	9.483771
307.1615	128.89345	168.30505	141.76686	8.533712	8.580712
12.9312	15.583692	28.324207	20.426624	5.466432	5.455357
12.9312	15.583692	28.324207	20.426624	5.466432	5.455357
12.9312	15.583692	28.324207	20.426624	5.466432	5.455357
4.9999995	12.0049515	4.9999995	9.973499	7.624617	2.832375
335.4812	161.29129	141.32378	123.09182	9.02409	8.454784
335.4812	161.29129	141.32378	123.09182	9.02409	8.454784
335.4812	161.29129	141.32378	123.09182	9.02409	8.454784
30.52682	13.307809	14.161841	11.311721	4.628864	5.8234944
8.621654	25.971197	10.11073	16.787422	5.6060705	5.8498836
8.621654	25.971197	10.11073	16.787422	5.6060705	5.8498836
85.852715	33.31486	56.694847	32.964787	7.731335	7.9285445
85.852715	33.31486	56.694847	32.964787	7.731335	7.9285445
85.852715	33.31486	56.694847	32.964787	7.731335	7.9285445
85.852715	33.31486	56.694847	32.964787	7.731335	7.9285445
6.108042	19.773014	7.482897	5.9406147	4.3974247	4.88498
6.108042	19.773014	7.482897	5.9406147	4.3974247	4.88498

5.090735	5.167788	5.5333614	5.444613	4.1845884	4.788298
8.745526	7.9707837	9.701546	9.121104	7.791901	7.259163
12.613105	10.872356	14.151086	13.580471	12.4406595	9.445841
3.683047	3.2871265	4.4266796	3.209366	2.959929	2.4279919
4.9250574	5.493587	6.5181217	6.1814694	2.3467326	4.181725
11.484203	12.292572	11.515345	11.889115	9.8901825	10.293598
12.757199	11.400835	14.03268	13.352356	12.17855	9.132697
10.219658	11.413313	11.0663395	11.2308855	9.715997	9.711583
9.862339	9.430864	8.5582695	9.063037	7.70298	7.052748
9.862339	9.430864	8.5582695	9.063037	7.70298	7.052748
9.862339	9.430864	8.5582695	9.063037	7.70298	7.052748
9.862339	9.430864	8.5582695	9.063037	7.70298	7.052748
9.862339	9.430864	8.5582695	9.063037	7.70298	7.052748
9.862339	9.430864	8.5582695	9.063037	7.70298	7.052748
9.862339	9.430864	8.5582695	9.063037	7.70298	7.052748
9.862339	9.430864	8.5582695	9.063037	7.70298	7.052748
9.862339	9.430864	8.5582695	9.063037	7.70298	7.052748
9.862339	9.430864	8.5582695	9.063037	7.70298	7.052748
8.273411	7.424325	9.776927	9.049282	8.203857	7.942898
6.6842723	8.101908	7.6162686	7.793345	5.8680654	6.8609214
6.002259	5.8310738	4.8101873	5.257166	3.6997714	2.4279919
5.391615	4.505154	6.703521	6.210328	5.4731145	5.301784
9.8578415	10.037063	10.55558	10.373614	8.604717	9.059559
9.8578415	10.037063	10.55558	10.373614	8.604717	9.059559
9.8578415	10.037063	10.55558	10.373614	8.604717	9.059559
5.9956584	5.799695	5.0341306	5.5810823	4.80267	2.4279919
5.9956584	5.799695	5.0341306	5.5810823	4.80267	2.4279919
5.307632	4.253227	3.232346	3.9140952	3.6656382	2.4279919
5.307632	4.253227	3.232346	3.9140952	3.6656382	2.4279919
5.307632	4.253227	3.232346	3.9140952	3.6656382	2.4279919
5.307632	4.253227	3.232346	3.9140952	3.6656382	2.4279919
5.307632	4.253227	3.232346	3.9140952	3.6656382	2.4279919
4.6699743	5.7703047	5.896803	5.7934823	4.484425	4.36691
10.742585	11.312136	10.118137	10.6726	4.9066978	5.312208
8.176049	7.161021	8.11556	7.8128533	6.701433	5.6299725
11.116395	10.999118	10.850793	11.020242	9.472317	8.833116
6.1588383	5.4160337	4.326622	4.6212587	3.699278	2.4279919
4.7626176	5.5361476	5.385187	5.143562	4.093274	3.6832945
4.7626176	5.5361476	5.385187	5.143562	4.093274	3.6832945
4.7626176	5.5361476	5.385187	5.143562	4.093274	3.6832945
10.19199	9.272262	12.108326	11.396457	10.248859	9.008374
10.19199	9.272262	12.108326	11.396457	10.248859	9.008374
8.121112	6.0508866	5.482541	5.407181	4.6673965	5.3373027
12.022866	13.212936	11.877131	12.551671	10.240652	10.661263
10.771082	12.443952	9.877325	11.551827	9.355125	7.8232293
8.3500595	10.111395	8.63079	9.477616	7.398412	6.3345537
5.896246	4.8711433	6.480447	6.106975	3.1414146	3.7069004
5.896246	4.8711433	6.480447	6.106975	3.1414146	3.7069004
5.896246	4.8711433	6.480447	6.106975	3.1414146	3.7069004
8.629734	4.7966757	6.5785966	6.1180987	2.3467326	3.1226819
9.595597	9.476601	7.4853125	8.713057	7.9416356	7.640181
9.595597	9.476601	7.4853125	8.713057	7.9416356	7.640181
9.595597	9.476601	7.4853125	8.713057	7.9416356	7.640181
4.765168	6.1372123	4.8083024	5.565819	4.2866316	3.1826048
5.1403947	6.371144	5.768847	6.1184826	4.397764	5.578876
5.1403947	6.371144	5.768847	6.1184826	4.397764	5.578876
7.530159	7.2172213	7.114332	7.323999	5.971114	5.610603
7.530159	7.2172213	7.114332	7.323999	5.971114	5.610603
7.530159	7.2172213	7.114332	7.323999	5.971114	5.610603
7.530159	7.2172213	7.114332	7.323999	5.971114	5.610603
4.097916	5.62074	4.6486554	4.918872	2.3467326	2.4279919
4.097916	5.62074	4.6486554	4.918872	2.3467326	2.4279919

4.1675897	4.0945215	3.9621627	3.8853211
8.525056	6.892417	7.4298134	7.174183
14.026723	8.328192	9.568935	8.587746
3.7080941	2.3408759	2.3618152	2.352752
2.350709	4.419416	2.9345014	4.4635553
9.676732	10.1362505	10.320815	10.179638
13.523036	7.393526	9.269134	7.995866
9.807134	10.182105	10.067049	10.11471
8.155564	8.298145	8.577614	8.373737
8.155564	8.298145	8.577614	8.373737
8.155564	8.298145	8.577614	8.373737
8.155564	8.298145	8.577614	8.373737
8.155564	8.298145	8.577614	8.373737
8.155564	8.298145	8.577614	8.373737
8.155564	8.298145	8.577614	8.373737
8.155564	8.298145	8.577614	8.373737
8.155564	8.298145	8.577614	8.373737
8.155564	8.298145	8.577614	8.373737
8.796121	7.068897	7.996295	7.415657
5.0098743	7.17352	7.301441	7.0096207
4.719424	3.0451915	3.8839915	2.8188586
5.881412	3.8041415	4.700565	4.379718
8.375731	8.307229	9.411084	8.911987
8.375731	8.307229	9.411084	8.911987
8.375731	8.307229	9.411084	8.911987
5.8925796	4.280236	4.646239	4.3367057
5.8925796	4.280236	4.646239	4.3367057
3.5240734	2.998682	2.3618152	3.5884035
3.5240734	2.998682	2.3618152	3.5884035
3.5240734	2.998682	2.3618152	3.5884035
3.5240734	2.998682	2.3618152	3.5884035
3.5240734	2.998682	2.3618152	3.5884035
4.081836	3.218621	4.532228	3.2848558
4.917691	7.196063	5.1921396	6.422993
7.2542863	6.02673	7.2850866	6.7711973
9.888025	9.239677	9.805144	9.437645
4.1352634	2.7256498	2.8579519	3.0656493
4.364566	4.460566	3.9857454	4.650916
4.364566	4.460566	3.9857454	4.650916
4.364566	4.460566	3.9857454	4.650916
11.0612545	7.9381137	9.137076	8.048873
11.0612545	7.9381137	9.137076	8.048873
5.1400228	5.4146647	4.8404107	5.0418954
9.9888525	11.143431	11.040394	11.071013
9.963567	8.884542	9.184741	8.931026
8.209225	7.37786	7.633297	7.6408815
3.7714834	4.1033344	5.0995426	4.691737
3.7714834	4.1033344	5.0995426	4.691737
3.7714834	4.1033344	5.0995426	4.691737
2.350709	3.6973567	2.3618152	3.6042337
8.335113	7.7054634	7.380066	7.419286
8.335113	7.7054634	7.380066	7.419286
8.335113	7.7054634	7.380066	7.419286
4.9453607	3.8559897	4.094766	3.792684
3.2332416	4.8894477	3.6160347	4.3941407
3.2332416	4.8894477	3.6160347	4.3941407
6.392637	5.272189	6.0912414	5.437332
6.392637	5.272189	6.0912414	5.437332
6.392637	5.272189	6.0912414	5.437332
6.392637	5.272189	6.0912414	5.437332
2.809821	4.4631004	3.2034223	2.8329086
2.809821	4.4631004	3.2034223	2.8329086

ASHGA5P040852	0.000437307	0.014669266	3.3576113	up	noncoding
ASHGA5P040852	0.000437307	0.014669266	3.3576113	up	noncoding
ASHGA5P056671	0.001221249	0.023487681	2.6023138	up	noncoding
ASHGA5P045578	0.027724654	0.104047281	2.5366342	up	noncoding
ASHGA5P043096	0.004555005	0.042992804	2.0481031	up	noncoding
ASHGA5P026856	0.017092634	0.079591954	2.0057179	up	noncoding
ASHGA5P050614	0.010112809	0.062335956	3.9771806	up	noncoding
ASHGA5P058887	0.017827709	0.081055899	3.9794573	up	noncoding
ASHGA5P018381	0.018618725	0.082980206	2.2535105	up	noncoding
ASHGA5P046099	0.001401793	0.025091657	2.4535336	up	noncoding
ASHGA5P035262	0.028298081	0.104997102	2.4160471	up	noncoding
ASHGA5P054839	8.3884E-05	0.008738697	2.2739623	up	noncoding
ASHGA5P018694	0.000169723	0.011182026	3.0175993	up	noncoding
ASHGA5P000083	0.000483359	0.015384974	9.3371283	up	noncoding
ASHGA5P054138	0.00123762	0.023574374	3.7838473	up	noncoding
ASHGA5P034505	0.004791855	0.043918141	17.7722075	up	noncoding
ASHGA5P030919	0.022918508	0.093781666	2.0969046	up	noncoding
ASHGA5P044450	3.81857E-05	0.006301164	6.0784827	up	noncoding
ASHGA5P026007	0.002204926	0.030923045	3.8948482	up	noncoding
ASHGA5P016092	0.033380079	0.115144552	2.1888198	up	noncoding
ASHGA5P033553	0.029416565	0.107329537	2.264317	up	noncoding
ASHGA5P028160	0.000336542	0.013239107	2.4375562	up	noncoding
ASHGA5P055790	0.006471107	0.051079512	2.6662233	up	noncoding
ASHGA5P028953	0.013652392	0.071680418	2.0724724	up	noncoding
ASHGA5P052758	0.005645773	0.047739466	3.0051376	up	noncoding
ASHGA5P052472	0.044712612	0.135944249	2.5889303	up	noncoding
ASHGA5P016013	0.000687451	0.017500829	2.0327487	up	noncoding
ASHGA5P049029	0.032589587	0.113617438	4.1744088	up	noncoding
ASHGA5P054711	0.008651336	0.057996114	2.7481611	up	noncoding
ASHGA5P027698	0.001249834	0.023668906	4.6341222	up	noncoding
ASHGA5P057962	0.000192136	0.01146874	3.0272469	up	noncoding
ASHGA5P028638	0.001263752	0.023779671	2.3072418	up	noncoding
ASHGA5P034136	0.017482488	0.080423645	2.162035	up	noncoding
ASHGA5P047547	0.000424667	0.014669266	30.5139325	up	noncoding
ASHGA5P037472	0.003511235	0.038218567	4.8391859	up	noncoding
ASHGA5P037472	0.003511235	0.038218567	4.8391859	up	noncoding
ASHGA5P037472	0.003511235	0.038218567	4.8391859	up	noncoding
ASHGA5P037472	0.003511235	0.038218567	4.8391859	up	noncoding
ASHGA5P037472	0.003511235	0.038218567	4.8391859	up	noncoding
ASHGA5P043572	0.037005958	0.122123188	2.7176467	up	noncoding
ASHGA5P043572	0.037005958	0.122123188	2.7176467	up	noncoding
ASHGA5P049676	0.031117643	0.110685904	2.0011685	up	noncoding
ASHGA5P034511	0.000253	0.012149764	2.1748536	up	noncoding
ASHGA5P034511	0.000253	0.012149764	2.1748536	up	noncoding
ASHGA5P048255	0.003467668	0.037964495	2.6679569	up	noncoding
ASHGA5P040901	1.72001E-05	0.004580259	7.5035904	up	noncoding
ASHGA5P040901	1.72001E-05	0.004580259	7.5035904	up	noncoding
ASHGA5P031049	0.000978523	0.021189195	5.3717679	up	noncoding
ASHGA5P038623	0.030147772	0.108872954	5.1480403	up	noncoding
ASHGA5P056009	0.007258029	0.053618963	3.0977102	up	noncoding
ASHGA5P056009	0.007258029	0.053618963	3.0977102	up	noncoding
ASHGA5P045501	0.008738673	0.058198417	3.0596875	up	noncoding
ASHGA5P017795	0.000966207	0.021093715	4.4865692	up	noncoding
ASHGA5P048735	0.016546921	0.078495124	2.3783474	up	noncoding
ASHGA5P050941	0.029989435	0.108456761	2.5130975	up	noncoding
ASHGA5P048283	0.000116959	0.009923756	3.1676038	up	noncoding
ASHGA5P048283	0.000116959	0.009923756	3.1676038	up	noncoding
ASHGA5P046027	0.048419328	0.142802321	2.1644721	up	noncoding
ASHGA5P045253	0.010734312	0.064157533	4.5164737	up	noncoding
ASHGA5P043156	0.00171844	0.027747135	2.3188509	up	noncoding
ASHGA5P038688	0.001689118	0.027504186	2.6081425	up	noncoding
ASHGA5P049904	0.041052297	0.129441514	3.661103	up	noncoding

uc003lzi.3	BC035392	UCSC_knowngene	1269	chr5	-
uc003lzi.3	BC035392	UCSC_knowngene	1269	chr5	-
TCONS_00007073	XLOC_002996	LincRNAs identified	211	chr3	+
TCONS_00015184	XLOC_007240	LincRNAs identified	337	chr8	-
TCONS_00019744	XLOC_009537	LincRNAs identified	423	chr11	-
TCONS_00011856	XLOC_005347	LincRNAs identified	313	chr6	+
uc002tfs.4	LOC151009	UCSC_knowngene	3608	chr2	-
uc.403+	uc.403	UCR	206	chr16	+
ENST00000452793	RP3-395M20.8	GENCODE	569	chr1	-
ENST00000451781	RP11-706O15.5	GENCODE	490	chrX	-
ENST00000449819	AC093901.1	GENCODE	458	chr2	-
uc001nhg.1	LOC646813	UCSC_knowngene	648	chr11	+
ENST00000456563	RP11-706O15.3	GENCODE	793	chrX	-
AW673701		LincRNAs identified	514	chr11	-
ENST00000424306	RP11-308D16.4	GENCODE	2222	chrX	+
uc002nbr.3	UCA1	UCSC_knowngene	1413	chr19	+
ENST00000570409	RP11-461A8.4	GENCODE	1068	chr16	-
ENST00000520268	KB-1562D12.1	GENCODE	491	chr8	-
NR_052852	MARCKSL1	RefSeq	1337	chr1	-
uc010fjx.1	LINC00116	UCSC_knowngene	1260	chr2	-
ENST00000582697	RP11-958F21.1	GENCODE	386	chr18	-
ENST00000449572	RP11-426L16.8	GENCODE	704	chr1	-
uc001veb.2	DLEU2	UCSC_knowngene	722	chr13	-
ENST00000422615	DCUN1D2-AS1	GENCODE	421	chr13	+
NR_024403	LOC730101	RefSeq	3784	chr6	+
ENST00000524034	CTB-12O2.1	GENCODE	399	chr5	+
ENST00000425797	DTX2P1	pseudogene	1652	chr7	+
ENST00000573315	LINC00514	GENCODE	491	chr16	+
TCONS_00006377	XLOC_002996	LincRNAs identified	6659	chr3	+
ENST00000538806	RP11-474D1.3	GENCODE	4551	chr12	-
TCONS_00024435	XLOC_011752	LincRNAs identified	618	chr16	+
ENST00000449881	UGGT2-IT1	GENCODE	463	chr13	-
NR_027301	LOC148189	RefSeq	2085	chr19	-
NR_038220	LOC170425	RefSeq	2685	chr10	+
ENST00000454980	AP000695.4	GENCODE	649	chr21	+
ENST00000454980	AP000695.4	GENCODE	649	chr21	+
ENST00000454980	AP000695.4	GENCODE	649	chr21	+
ENST00000454980	AP000695.4	GENCODE	649	chr21	+
ENST00000454980	AP000695.4	GENCODE	649	chr21	+
ENST00000504773	RP11-510N19.5	GENCODE	987	chr1	+
ENST00000504773	RP11-510N19.5	GENCODE	987	chr1	+
NR_033465	NT5C3L	RefSeq	1654	chr17	-
uc002ndl.1	AK095546	UCSC_knowngene	2675	chr19	+
uc002ndl.1	AK095546	UCSC_knowngene	2675	chr19	+
ENST00000531806	FAM86C2P	GENCODE	1814	chr11	-
ENST00000514846	RP11-1334A24.4	GENCODE	543	chr5	-
ENST00000514846	RP11-1334A24.4	GENCODE	543	chr5	-
ENST00000501520	RP11-57A19.2	GENCODE	2481	chr16	-
NR_033944	LOC647323	RefSeq	1625	chr3	-
NR_029435	FLJ31306	RefSeq	2457	chr14	-
NR_029435	FLJ31306	RefSeq	2457	chr14	-
ENST00000436510	RP11-65J3.3	GENCODE	1846	chr9	-
ENST00000445836	TMEM191A	GENCODE	671	chr22	+
uc001bql.3	SNHG12	UCSC_knowngene	1080	chr1	-
uc002wuz.3	ZNF337	UCSC_knowngene	4664	chr20	+
uc001opt.1	AK125463	UCSC_knowngene	2576	chr11	-
uc001opt.1	AK125463	UCSC_knowngene	2576	chr11	-
BC034527R		LincRNAs identified	1077	chr1	+
uc004aej.3	BC065763	UCSC_knowngene	1242	chr9	-
uc022agj.1	PMS2P3	UCSC_knowngene	1262	chr7	-
ENST00000438698	AC034193.5	pseudogene	534	chr3	+
TCONS_00002579	XLOC_001192	LincRNAs identified	262	chr1	-

162909609	162921064		GTTCTCCAGCTTAAGATCTTCTATCT	natural antisense
162909609	162921064		GTTCTCCAGCTTAAGATCTTCTATCT	natural antisense
195367105	195377054		TAAAAATTATTACGGTGAACAGTCT	intron sense-overlapp
142208033	142209262		ACCACCAAAGTGAAGGTGAGTTTAC	intergenic
103713661	103715301	TCONS_00020124	TTCTTCTGGTTTTTGTGTTTGTGGCT	intergenic
74364303	74365877		TTTAGGCGGAAAAACAGCAATGGA/	intergenic
111132685	111142113	NR_027244, uc010fj	ATATTGAGCGAA(151009	intergenic
54323854	54324060		GTGAAGCGTAGACAAGAATTTAATC	intergenic
2485679	2488470		GGAAGCCCAGCGTGGGCTCTCCCGC	natural antisense
3811332	3823750	ENST00000432445,	AGATGGTGCTATCTGAGAAAGCCAA	intergenic
118940596	118943962	ENST00000449075	GAACATTAAATCCGACACTTGTACT.	intergenic
50368317	50379803	ENST00000532521,	TGTGTTTCAGGCT646813	intergenic
3772389	3785176	ENST00000457936	AGAGTCAGCACGCGGTGGCCTGGGT	intergenic
2228859	2229381		TTACAACTGGGGCAGGCTGACAACC	intergenic
135991553	136075814		TTTTAGTACCACTAACATTTTTTGGT	intergenic
15939756	15946230	TCONS_00026909	AACATTATTCCTT652995	intergenic
3700636	3701704		AACTCACAGAGCACCATCAGATCCT	intronic antisense
102473404	102504727		AATTCAAAAGCCTGGCACAGTTGGG	natural antisense
32799429	32801840		CGGAGCGGAGGA65108	exon sense-overlappi
110969791	110975365	ENST00000426713	TTACAGTCGGAC(205251	intergenic
22534170	22567629		TGTTTGGATTTGGGAATGTAAGGAC	intergenic
113362911	113392401	ENST00000401018,	CGCAGACCAGACCCGCCCCGCCTCT	intergenic
50623324	50656139	ENST00000449579	TTTTACCAAGGT/8847	bidirectional
114110943	114116670		GGGTACCACAAGAGGATGGAAAGA.	natural antisense
52529198	52533951		GTGTTTCCAGAT(730101	intergenic
151338458	151650006	ENST00000518431,	CCACTGGTCTTAAAGACAGACCTGC	intergenic
76607933	76633625		AACGGCAATAGATGGAAGTCTGCAGTGTCCCTCCTGCA	
3051300	3052017		CTTCATTCTTTGT283875	intergenic
195370820	195377621		TGTGGCTTCGTCATCATTATCCATGC	intron sense-overlapp
130517998	130529602	ENST00000567788	AAGGTGAAGTCAGAGGCTAAAAATC	intergenic
64294312	64295136		TCAAAATAAACCCATCTTGCATCAG	intergenic
96567600	96568330		CATAGGGAGACAGACAATAAGTGAT	intron sense-overlapp
28281400	28284848		CATGATGTTTTCT148189	intergenic
86039735	86054415		TCTGACTCTGGA(170425	intergenic
37818119	37833050		CCACTGACCAAACTCCCAAGTCACT	natural antisense
37818119	37833050		CCACTGACCAAACTCCCAAGTCACT	natural antisense
37818119	37833050		CCACTGACCAAACTCCCAAGTCACT	natural antisense
37818119	37833050		CCACTGACCAAACTCCCAAGTCACT	natural antisense
37818119	37833050		CCACTGACCAAACTCCCAAGTCACT	natural antisense
201980497	201984785		TTTAATGATCAGACCCCAGTCACAG	exon sense-overlappi
201980497	201984785		TTTAATGATCAGACCCCAGTCACAG	exon sense-overlappi
39981333	39992523		TCAGTGGTTCTCT115024	exon sense-overlappi
16210476	16213151		CTCCCAAATTTGGTGCTTGTTCCTTA	exon sense-overlappi
16210476	16213151		CTCCCAAATTTGGTGCTTGTTCCTTA	exon sense-overlappi
67559118	67572793		CTAGGAGACACA645332	intergenic
176869803	176871581		GACATGTGGCAGCTTACGTTGGACA	natural antisense
176869803	176871581		GACATGTGGCAGCTTACGTTGGACA	natural antisense
28296205	28303385		GACATAAATGTGATAAAATGTTGAC	bidirectional
193675160	193721448		CAAGTTTCAGTG/647323	intergenic
58732082	58764855		TAAAGAAAAGCA379025	intronic antisense
58732082	58764855		TAAAGAAAAGCA379025	intronic antisense
132192930	132209569		GCCACAAAAACATCATCAGTAGGTA	intergenic
21057128	21058793		CGAGTGAGCGCC84222	intergenic
28905049	28908366	uc001bqn.3, uc001bc	TCAGACAGGTTT(85028	natural antisense
25604680	25658708		CGCTTCTCACGTAAGTGTCTCTTTA/	natural antisense
70219515	70244568		GCATTCCAGGAATTCTGGGAGTCAT	natural antisense
70219515	70244568		GCATTCCAGGAATTCTGGGAGTCAT	natural antisense
211713915	211714910		TCCGAGAGCTGCTCTCCCATTTCCC/	intergenic
66688085	66699951		CTGAAGAAGAACAGAGCTCAAGAAT	intergenic
75140252	75157453	ENST00000418756,	AGGGGCCGGGAG5387	intergenic
10029587	10036015		AGTCTCCTATACAAATAATGTGTTTATGTTTTTTGACAC	
212605053	212606095		GATGCCCGGCTTGCTGGAGGATCAA	intergenic

NM_012484	HMMR	hyaluronan mediated	+	162887516	162918953
NM_012485	HMMR	hyaluronan mediated	+	162887516	162918953
ENST00000381954	MUC20	mucin 20, cell surfac	+	195343315	195460421
NM_003820	TNFRSF14	tumor necrosis factor	+	2487804	2495267
ENST00000407479	DNASE1	deoxyribonuclease I	+	3692938	3708095
NM_024915	GRHL2	grainyhead-like prote	+	102504667	102681952
NM_023009	MARCKSL1	MARCKS-related pr	-	32799429	32801840
ENST00000378180	DLEU1	deleted in lymphocyt	+	50656306	50679433
NM_001014283	DCUN1D2	DCN1-like protein 2	-	114110133	114145023

AAAACCATCTATGAGAGAAGACG

ENST00000381954	MUC20	mucin 20, cell surfac	+	195343315	195460421
NM_020121	UGGT2	UDP-glucose:glycop	-	96453835	96705736
NM_001146077	CLDN14	claudin-14	-	37832919	37948867
NM_001146078	CLDN14	claudin-14	-	37832919	37915117
NM_001146079	CLDN14	claudin-14	-	37832919	37852388
NM_012130	CLDN14	claudin-14	-	37832919	37838739
NM_144492	CLDN14	claudin-14	-	37832919	37852388
NM_001114309	ELF3	ETS-related transcrip	+	201979689	201986315
NM_004433	ELF3	ETS-related transcrip	+	201979689	201986315
NM_052935	NT5C3L	cytosolic 5'-nucleotic	-	39981333	39992523
NM_001145160	TPM4	tropomyosin alpha-4	+	16178316	16213813
NM_003290	TPM4	tropomyosin alpha-4	+	16187134	16213813
NM_001004106	GRK6	G protein-coupled re	+	176853686	176869850
NM_002082	GRK6	G protein-coupled re	+	176853686	176869850
NM_001024401	SBK1	serine/threonine-prot	+	28303839	28335170
NM_002788	PSMA3	proteasome subunit a	+	58711522	58738727
NM_152132	PSMA3	proteasome subunit a	+	58711522	58738727
NM_017846	TRNAU1AP	tRNA selenocysteine	+	28879528	28905057
NM_015655	ZNF337	zinc finger protein 33	-	25654850	25677469
NM_003626	PPFIA1	liprin-alpha-1 isoform	+	70116805	70230607
NM_177423	PPFIA1	liprin-alpha-1 isoform	+	70116805	70224598

CATACTGCCAGCAGGTATGTTG

35.743354	8.217428	4.761431	3.013996	28.848679	30.931328
35.743354	8.217428	4.761431	3.013996	28.848679	30.931328
89.333248	23.571685	6.09764	4.717845	79.254486	62.160133
1644.71054	443.894467	10.180597	8.837682	1799.4796	2131.417
16.575387	5.690177	3.703537	2.669249	27.071032	15.587858
22.524351	8.517995	4.168552	3.164433	26.288408	26.254074
44.5375	7.765787	4.957296	2.96555	63.811043	83.88483
84.286278	12.144058	5.769577	3.777005	40.43009	52.89717
402.177147	117.749594	8.211492	7.039318	479.1638	496.6275
292.103047	72.940665	7.752999	6.458138	302.593	388.77658
72.743854	19.381447	5.604343	4.331694	49.289764	73.08426
890.372632	231.224205	9.351324	8.166116	1001.13684	1198.0511
724.678337	140.897117	9.045275	7.451874	734.732	859.53107
210.394465	12.663372	7.091837	3.868858	171.92708	202.08257
340.269048	55.383155	7.74581	5.825956	335.6176	127.40499
8503.295788	127.278092	10.876458	6.724907	2056.0425	143.12509
36.972593	13.099757	4.869868	3.801606	38.336845	34.391212
68.606253	7.448274	5.589311	2.985599	38.605637	32.41418
252.517477	41.560853	7.569564	5.607997	307.28113	311.13727
50.018812	15.221065	5.199738	4.069585	57.471268	85.22201
104.114252	30.090408	6.212406	5.03333	88.29154	65.18645
3036.799417	801.78622	11.138062	9.852626	3225.2925	3208.1794
74.286039	18.763335	5.818878	4.40408	85.41546	87.7056
38.193177	15.071641	4.898896	3.847543	38.92149	20.45905
54.535814	10.735258	5.145301	3.55787	119.90071	83.328125
111.325748	17.252755	5.757733	4.385376	40.94472	64.71338
366.183722	116.028485	8.122691	7.099259	379.43777	372.69214
1233.225955	104.872228	8.598573	6.537001	2352.4934	4120.9424
171.706517	47.625787	7.067689	5.609223	176.7731	153.90637
116.469383	9.579358	5.432949	3.220653	198.75078	394.23196
339.502457	68.186235	8.010211	6.412205	295.61853	236.96039
230.413064	58.967725	7.359937	6.153768	194.59143	150.87617
56.490585	17.948676	5.43081	4.31842	55.31866	43.085728
612.607975	15.382745	8.789092	3.857696	713.3598	531.1806
76.021759	9.473908	5.690136	3.415371	55.72007	28.205908
76.021759	9.473908	5.690136	3.415371	55.72007	28.205908
76.021759	9.473908	5.690136	3.415371	55.72007	28.205908
76.021759	9.473908	5.690136	3.415371	55.72007	28.205908
76.021759	9.473908	5.690136	3.415371	55.72007	28.205908
122.479975	30.494795	6.357588	4.91523	100.90679	61.512558
122.479975	30.494795	6.357588	4.91523	100.90679	61.512558
86.529162	25.708518	5.985448	4.984606	68.6536	32.70743
127.6219	35.602934	6.54532	5.424402	143.69469	204.22728
127.6219	35.602934	6.54532	5.424402	143.69469	204.22728
667.34513	151.310647	8.963216	7.547481	844.57745	813.09937
1176.035712	78.448371	9.374669	6.467087	921.7869	1458.1765
1176.035712	78.448371	9.374669	6.467087	921.7869	1458.1765
552.783603	53.803954	8.49138	6.065983	678.0652	1062.6821
74.126109	6.086488	4.967065	2.603042	136.91422	10.44712
1083.642423	231.97131	9.64036	8.009158	901.8561	979.4404
1083.642423	231.97131	9.64036	8.009158	901.8561	979.4404
128.366218	38.610105	6.561264	4.94788	192.15558	134.51709
202.191623	30.122075	7.275726	5.110113	246.0883	254.50757
574.907075	164.44393	8.743764	7.493805	619.4312	385.91397
51.73774	14.491109	5.274452	3.944986	39.98769	42.810726
214.94364	40.476353	7.309282	5.64589	253.22597	357.76944
214.94364	40.476353	7.309282	5.64589	253.22597	357.76944
1080.94565	330.216707	9.572644	8.458629	1054.4691	997.0217
1727.092292	175.449777	9.958234	7.783037	2020.9138	3116.4175
5512.67895	1486.409325	11.955734	10.742324	6564.956	7562.579
61.516322	15.343968	5.554322	4.171299	69.69849	80.015335
213.181127	62.887276	7.10782	5.235542	245.99568	253.16605

21.349968	63.77368	27.395817	42.160652	4.9999995	4.9999995
21.349968	63.77368	27.395817	42.160652	4.9999995	4.9999995
97.75098	126.14557	61.56931	109.11901	25.193968	9.814333
1187.5463	717.63684	2114.0188	1918.1647	447.87534	154.23895
15.33202	15.349431	12.93442	13.177559	4.9999995	5.402302
23.374538	19.576914	19.00107	20.651102	9.316266	4.9999995
22.676037	22.978474	35.370087	38.50453	9.018608	4.9999995
22.55525	130.94618	113.33327	145.55571	8.299268	11.271199
276.6647	226.87265	488.36923	445.365	99.04918	54.141685
189.36174	169.8839	365.77036	336.2327	53.17378	78.4128
16.56826	74.30254	104.72871	118.48959	12.45331	19.677275
723.01025	478.3789	957.8532	983.8055	266.65582	192.60036
555.73517	371.7967	947.63293	878.64215	116.76311	103.1199
102.90752	474.43735	65.0383	245.97397	12.267082	16.83908
543.61053	575.617	85.33707	374.0271	45.461735	10.850509
4240.729	386.44214	24688.797	19504.639	40.858986	33.167015
43.14393	28.44066	37.408657	40.114254	12.85729	4.9999995
48.180386	135.24219	55.814735	101.38039	4.9999995	4.9999995
315.10446	152.30045	205.42587	223.85568	33.731365	19.939058
23.15095	41.353237	46.29913	46.616276	21.839361	12.68461
71.02745	228.63449	64.36708	107.1785	29.182713	14.994508
3850.4094	2423.658	2560.599	2952.6582	1076.2179	463.2567
66.36852	42.982265	67.89449	95.3499	16.38625	12.39504
48.888992	46.200993	38.133812	36.554726	21.74287	4.9999995
35.742836	20.599539	37.24178	30.401894	13.714491	6.461871
11.0045805	295.19147	58.485905	197.61443	16.43115	14.4272
336.5605	247.2013	424.33032	436.8803	106.467316	54.112778
342.68726	85.93062	252.71477	244.58728	159.80539	257.72598
184.0559	193.88408	138.1988	183.42085	49.899536	17.75549
21.710176	15.201671	36.61296	32.30875	12.39952	23.426628
309.51688	442.29	327.96976	424.65918	61.04462	52.895264
218.87082	109.427414	371.39212	337.32043	60.73437	47.482346
49.296852	39.22972	69.68234	82.33021	17.30963	7.067106
806.5918	254.28299	714.69366	655.539	24.617731	32.629646
62.956486	42.82045	136.32782	130.09982	4.9999995	7.744493
62.956486	42.82045	136.32782	130.09982	4.9999995	7.744493
62.956486	42.82045	136.32782	130.09982	4.9999995	7.744493
62.956486	42.82045	136.32782	130.09982	4.9999995	7.744493
62.956486	42.82045	136.32782	130.09982	4.9999995	7.744493
111.43712	269.06726	41.59275	150.36337	14.767531	5.4955564
111.43712	269.06726	41.59275	150.36337	14.767531	5.4955564
97.31874	131.95213	73.92448	114.61859	24.171225	22.271128
63.713238	146.50258	78.98239	128.61122	36.10927	41.604836
63.713238	146.50258	78.98239	128.61122	36.10927	41.604836
740.1899	438.29596	581.9954	585.9127	107.43762	110.137
174.86769	2432.772	481.81848	1586.7927	58.17524	69.56645
174.86769	2432.772	481.81848	1586.7927	58.17524	69.56645
290.2151	645.7134	159.76642	480.2594	56.811165	54.38839
230.09245	15.527152	21.199497	30.576218	4.9999995	4.9999995
704.33704	1396.0251	948.2408	1571.9551	268.27924	90.21822
704.33704	1396.0251	948.2408	1571.9551	268.27924	90.21822
147.722	61.344448	140.41689	94.0413	52.99515	12.40872
213.08072	180.88579	134.56601	184.02135	24.94623	12.45655
734.6096	666.35535	386.53513	656.5972	116.4925	55.919193
28.404747	79.85288	42.033714	77.33668	9.362847	6.605767
154.71037	139.22707	174.94151	209.78748	42.835114	32.84622
154.71037	139.22707	174.94151	209.78748	42.835114	32.84622
717.3736	540.3303	1774.5244	1401.9548	319.77353	131.0913
845.1896	229.50085	2327.5186	1823.0134	164.42448	198.95477
4867.5547	3208.631	5601.558	5270.795	1995.8896	926.8179
41.780792	44.23447	65.818535	67.55031	14.75055	9.308372
158.21764	39.70247	297.41318	284.59174	99.604385	24.104704

6.108042	19.773014	7.482897	5.9406147	4.3974247	4.88498
6.108042	19.773014	7.482897	5.9406147	4.3974247	4.88498
44.664013	20.8659	21.716894	19.175	5.8557734	5.7929482
1193.6039	290.841	351.28687	225.52074	10.329566	10.644848
7.712011	6.0267496	4.9999995	4.9999995	4.304737	3.9817667
12.9897	4.9999995	10.20149	8.600516	4.2599893	4.66343
15.519981	6.607282	5.4488506	4.9999995	5.5380483	6.195458
20.820597	16.713737	6.2615814	9.497968	4.874274	5.5854106
266.93237	95.86615	104.89329	85.61489	8.426655	8.644871
39.066635	69.0768	113.993484	83.92049	7.79063	8.309243
7.150571	47.321815	9.099247	20.586464	5.162209	6.0075507
335.0273	152.85	263.87793	176.33382	9.499339	9.868406
130.33287	125.81471	221.80368	147.54843	9.05374	9.408349
4.9999995	14.67985	13.03613	14.15809	6.973092	7.4085836
112.08621	72.58059	42.408134	48.91175	7.9376025	6.7640977
53.175644	65.32799	398.45743	172.68149	10.52566	6.9299755
13.51466	14.550781	17.658741	15.01707	4.7947555	5.02265
5.036817	9.986807	10.96512	8.700901	4.8057127	4.9445496
36.77008	39.697624	69.51012	49.71687	7.8095045	8.006871
25.34028	5.833809	13.465489	12.16284	5.3826704	6.217394
67.296196	25.21676	24.810112	19.042162	6.011455	5.8543725
1696.9365	540.7616	551.99774	481.54688	11.177897	11.228771
35.366592	22.508287	15.172562	10.751279	5.9628596	6.2514596
31.012033	12.090981	10.153841	10.4301195	4.8180795	4.325995
18.250767	4.9999995	12.030701	8.953717	6.4550276	6.1855717
11.376429	25.46444	16.36605	19.45126	4.8932486	5.8440833
150.79462	86.46706	186.27174	112.057396	8.107108	8.249466
21.47085	111.6025	28.37472	50.25393	10.724892	11.586438
88.72244	24.101582	80.10692	25.168756	7.0121365	7.0299687
4.9999995	4.9999995	6.6499987	4.9999995	7.183602	8.327551
60.17635	69.70817	96.10765	69.185356	7.755659	7.6258564
78.25968	36.70279	80.59834	50.028824	7.155577	7.0015287
28.882566	25.823494	16.363249	12.246011	5.323874	5.316849
14.501229	10.34913	4.9999995	5.198735	9.009434	8.743496
10.338938	15.375312	11.1682	7.2165084	5.336431	4.761637
10.338938	15.375312	11.1682	7.2165084	5.336431	4.761637
10.338938	15.375312	11.1682	7.2165084	5.336431	4.761637
10.338938	15.375312	11.1682	7.2165084	5.336431	4.761637
10.338938	15.375312	11.1682	7.2165084	5.336431	4.761637
31.240969	36.545258	54.531273	40.38818	6.2065506	5.780081
31.240969	36.545258	54.531273	40.38818	6.2065506	5.780081
16.821096	32.938984	27.287365	30.761312	5.6405935	4.954466
29.311617	53.86818	19.14773	33.575974	6.7152367	7.422526
29.311617	53.86818	19.14773	33.575974	6.7152367	7.422526
152.99402	128.99031	231.36476	176.94017	9.260468	9.340039
32.054016	164.45927	51.685104	94.750145	9.380393	10.135849
32.054016	164.45927	51.685104	94.750145	9.380393	10.135849
67.31034	46.69796	56.619213	40.996655	8.935019	9.711216
4.9999995	4.9999995	11.518931	4.9999995	6.6431293	3.4550548
480.2759	143.4264	263.21802	146.41008	9.345069	9.592757
480.2759	143.4264	263.21802	146.41008	9.345069	9.592757
121.14499	12.685861	16.1788	16.247107	7.135289	6.8391075
41.332455	25.928501	46.229435	29.839281	7.4875226	7.7255597
209.70927	95.270836	322.27097	187.00081	8.801858	8.299278
24.364336	7.2382727	22.266954	17.108479	4.8609715	5.308319
49.94914	31.464169	45.150177	40.613297	7.5271125	8.194462
49.94914	31.464169	45.150177	40.613297	7.5271125	8.194462
851.4444	245.14005	255.55846	178.2925	9.569232	9.616721
96.02811	200.26047	205.07706	187.95377	10.498606	11.190278
3060.5527	686.86395	1404.1372	844.1946	12.209661	12.426967
24.17912	13.331732	16.555067	13.938968	5.663678	6.1291175
214.59908	7.131518	19.094799	12.78917	7.486947	7.7181187

4.097916	5.62074	4.6486554	4.918872	2.3467326	2.4279919
4.097916	5.62074	4.6486554	4.918872	2.3467326	2.4279919
6.289446	6.646807	5.75281	6.248054	4.897938	4.0767684
9.844387	9.180233	10.761244	10.323305	9.068845	8.119947
3.6132596	3.3773136	3.6318192	3.3123264	2.3467326	3.168814
4.2373147	3.7661543	4.152366	3.932056	3.4335835	2.4279919
4.1914296	4.0302577	4.9917154	4.7968655	3.3867471	2.4279919
4.182831	6.70329	6.6100464	6.661609	3.2587466	4.2857423
7.776466	7.520152	8.67129	8.229517	6.8646913	6.607797
7.2429748	7.0795565	8.259016	7.8365755	5.969233	7.142981
3.7337983	5.855495	6.497099	6.3699036	3.8676736	5.127019
9.146963	8.587312	9.650996	9.354929	8.320385	8.449027
8.761304	8.217877	9.637581	9.192798	7.105855	7.5370455
6.36954	8.575204	5.8269167	7.3976846	3.8455462	4.8898206
8.731722	8.850399	6.210328	7.980708	5.7361293	4.2291527
11.699583	8.27385	14.204054	13.625623	5.587536	5.888458
5.1078234	4.368171	5.073363	4.8524427	3.9104486	2.4279919
5.2729254	6.7515287	5.617491	6.143657	2.3467326	2.4279919
7.965569	6.924188	7.4430404	7.268209	5.3053064	5.1464767
4.224234	4.9537234	5.3638916	5.056514	4.685957	4.4622335
5.8381834	7.5319133	5.8137054	6.224807	5.097228	4.719104
11.547944	10.906386	11.049557	10.917817	10.375759	9.725263
5.7448416	5.0133963	5.8850656	6.0556455	4.2650223	4.42722
5.2965736	5.1258764	5.099798	4.7270536	4.680293	2.4279919
4.843294	3.8473296	5.0666924	4.4738917	4.0050807	3.4465315
3.13718	7.890339	5.682056	7.0994887	4.2694855	4.660921
8.060904	7.6424513	8.471642	8.204572	6.971765	6.6061606
8.081514	6.0692787	7.740992	7.388323	7.5659614	8.86651
7.201823	7.280886	6.8894434	6.9918776	5.8755355	4.972652
4.1229396	3.3622644	5.0413094	4.5600276	3.8609853	5.379029
7.935874	8.479584	8.106307	8.157988	6.1655693	6.5743484
7.451929	6.4281397	8.280339	7.842108	6.1595507	6.416201
5.3075356	4.8710284	5.9211416	5.8444333	4.3512177	3.583119
9.303602	7.681485	9.218643	8.777891	4.8641167	5.867154
5.669308	5.0083966	6.8659334	6.499109	2.3467326	3.7152045
5.669308	5.0083966	6.8659334	6.499109	2.3467326	3.7152045
5.669308	5.0083966	6.8659334	6.499109	2.3467326	3.7152045
5.669308	5.0083966	6.8659334	6.499109	2.3467326	3.7152045
5.669308	5.0083966	6.8659334	6.499109	2.3467326	3.7152045
6.481742	7.7514086	5.220528	6.7052193	4.1154904	3.1954978
6.481742	7.7514086	5.220528	6.7052193	4.1154904	3.1954978
6.2826185	6.713787	6.002713	6.3185124	4.837557	5.3061304
5.6876426	6.8662877	6.0994687	6.48076	5.40983	6.224098
5.6876426	6.8662877	6.0994687	6.48076	5.40983	6.224098
9.18208	8.464981	8.923181	8.608549	6.983787	7.6308746
7.120654	10.912378	8.650681	10.048056	6.099095	6.9684153
7.120654	10.912378	8.650681	10.048056	6.099095	6.9684153
7.8492575	9.021839	7.0967593	8.334188	6.065835	6.615508
7.5240226	3.3972692	4.3011312	4.4817843	2.3467326	2.4279919
9.106663	10.124751	9.639024	10.0338955	8.327092	7.345213
9.106663	10.124751	9.639024	10.0338955	8.327092	7.345213
6.879807	5.5634136	6.913404	6.036565	5.9632554	4.429617
7.419056	7.176334	6.848785	6.997098	4.883735	4.435931
9.168936	9.071902	8.34033	8.78028	7.1029854	6.657811
4.5200667	5.9611406	5.2330494	5.7631674	3.4402542	3.4813995
6.9389825	6.795329	7.2237377	7.176069	5.6542716	5.8755355
6.9389825	6.795329	7.2237377	7.176069	5.6542716	5.8755355
9.132697	8.755491	10.495647	9.866077	8.591352	7.8851233
9.372145	7.537774	10.907656	10.242946	7.609612	8.495712
11.898183	11.3147135	12.142589	11.74229	11.277311	10.734393
5.0649405	5.056514	5.843215	5.5684667	4.1140037	3.9939806
6.971159	4.8891463	7.972278	7.609274	6.8731723	5.4233265

2.809821	4.4631004	3.2034223	2.8329086
2.809821	4.4631004	3.2034223	2.8329086
5.4780974	4.549365	4.712892	4.592008
10.16732	8.6294	8.690093	8.350485
3.0874476	2.697931	2.3618152	2.352752
3.7762213	2.3408759	3.6295815	3.3783438
4.020419	2.823179	2.782209	2.352752
4.419416	4.2057467	2.959929	3.5324502
8.005969	6.91579	6.969453	6.8722057
5.288601	6.419374	7.087643	6.8409977
2.9955173	5.8244696	3.4711406	4.704344
8.332223	7.6304564	8.297893	7.9667106
6.982648	7.34035	8.047441	7.697902
2.350709	4.0111647	3.9788797	4.1370273
6.769919	6.501544	5.679334	6.039655
5.7238626	6.332508	8.881201	7.935874
3.8309355	3.9980068	4.416343	4.2259126
2.5910902	3.4215405	3.7299023	3.3963394
5.202394	5.5477166	6.38396	6.0621257
4.685957	2.6548626	4.0228925	3.9056056
6.0530887	4.8463974	4.9033103	4.5808525
10.668611	9.536282	9.341576	9.468268
5.150168	4.665109	4.1976686	3.7192945
4.969976	3.7099097	3.6227787	3.6743095
4.244957	2.3408759	3.8657537	3.444022
3.603506	4.859937	4.304893	4.6135163
7.185799	6.765874	7.7901154	7.275838
4.4593306	7.149307	5.1025853	6.078312
6.440709	4.772693	6.5832157	5.0105305
2.350709	2.3408759	3.0395663	2.352752
5.8973746	6.4347696	6.8429418	6.5582266
6.255379	5.4292765	6.5900054	6.0721936
4.873985	4.881957	4.3045616	3.9156823
3.9242282	3.4754248	2.3618152	2.6534348
3.4754248	4.083042	3.7578416	3.1139834
3.4754248	4.083042	3.7578416	3.1139834
3.4754248	4.083042	3.7578416	3.1139834
3.4754248	4.083042	3.7578416	3.1139834
3.4754248	4.083042	3.7578416	3.1139834
4.979194	5.422139	6.033459	5.7456017
4.979194	5.422139	6.033459	5.7456017
4.1310453	5.25502	5.0422177	5.335664
4.8934426	6.028906	4.524849	5.4652863
4.8934426	6.028906	4.524849	5.4652863
7.206245	7.3796406	8.110435	7.9739037
5.0158744	7.7346745	5.962536	7.0219293
5.0158744	7.7346745	5.962536	7.0219293
6.053357	5.802692	6.090138	5.768367
2.350709	2.3408759	3.79919	2.352752
8.863318	7.5375977	8.29456	7.6871657
8.863318	7.5375977	8.29456	7.6871657
6.8803277	3.782194	4.2886376	4.3432484
5.3666897	4.887112	5.7994223	5.2877893
7.6636105	6.9052825	8.572605	8.060533
4.631235	2.9482548	4.7484694	4.420302
5.638393	5.1869454	5.7657437	5.754452
5.638393	5.1869454	5.7657437	5.754452
9.675767	8.359835	8.2552805	7.984416
6.5472045	8.046786	7.9314446	8.067465
11.541893	9.893059	10.712136	10.295151
4.6203313	3.85964	4.3248324	4.1150074
7.6891384	2.9273627	4.519997	3.980256

ASHGA5P045311	0.013825445	0.07198363	2.5073394	up	noncoding
ASHGA5P037726	0.036708206	0.121581005	2.156068	up	noncoding
ASHGA5P055763	6.37423E-05	0.007893824	3.611404	up	noncoding
ASHGA5P029632	0.000155496	0.011056858	2.7827625	up	noncoding
ASHGA5P037994	0.013389521	0.070917754	2.4246319	up	noncoding
ASHGA5P031393	0.021757707	0.091077182	4.4473114	up	noncoding
ASHGA5P027059	0.032129545	0.112554199	3.1101796	up	noncoding
ASHGA5P031130	0.0004765	0.015255446	2.306828	up	noncoding
ASHGA5P043361	0.00017198	0.011182026	5.9949398	up	noncoding
ASHGA5P043361	0.00017198	0.011182026	5.9949398	up	noncoding
ASHGA5P043361	0.00017198	0.011182026	5.9949398	up	noncoding
ASHGA5P043361	0.00017198	0.011182026	5.9949398	up	noncoding
ASHGA5P033014	0.001377148	0.024808723	2.4859323	up	noncoding
ASHGA5P033380	0.0002102	0.01146874	3.4541684	up	noncoding
ASHGA5P033380	0.0002102	0.01146874	3.4541684	up	noncoding
ASHGA5P028926	0.03182747	0.112017423	3.9463565	up	noncoding
ASHGA5P022981	0.01612757	0.077717436	2.1123187	up	noncoding
ASHGA5P053588	0.000843704	0.019498573	3.4628862	up	noncoding
ASHGA5P018993	0.046839374	0.139710963	2.2944481	up	noncoding
ASHGA5P022793	0.037808035	0.123545266	3.3375087	up	noncoding
ASHGA5P057875	0.020864253	0.088884901	2.0397438	up	noncoding
ASHGA5P019333	5.78897E-05	0.007736798	2.0630134	up	noncoding
ASHGA5P045847	6.16573E-05	0.007847253	4.3784336	up	noncoding
ASHGA5P000402	0.001623434	0.027071307	2.5585251	up	noncoding
ASHGA5P030704	0.004883818	0.044366328	2.1401259	up	noncoding
ASHGA5P023163	0.036221777	0.120725876	2.4160678	up	noncoding
ASHGA5P041069	0.004865324	0.044314367	2.4015762	up	noncoding
ASHGA5P041069	0.004865324	0.044314367	2.4015762	up	noncoding
ASHGA5P021844	0.000234398	0.011897921	2.9975855	up	noncoding
ASHGA5P021844	0.000234398	0.011897921	2.9975855	up	noncoding
ASHGA5P021844	0.000234398	0.011897921	2.9975855	up	noncoding
ASHGA5P030044	0.014787132	0.074317791	2.6043575	up	noncoding
ASHGA5P030044	0.014787132	0.074317791	2.6043575	up	noncoding
ASHGA5P030044	0.014787132	0.074317791	2.6043575	up	noncoding
ASHGA5P030044	0.014787132	0.074317791	2.6043575	up	noncoding
ASHGA5P030044	0.014787132	0.074317791	2.6043575	up	noncoding
ASHGA5P051828	0.001549201	0.026466815	3.6392324	up	noncoding
ASHGA5P040127	0.00280232	0.034179323	2.0379068	up	noncoding
ASHGA5P045185	0.002217407	0.031027561	5.4211114	up	noncoding
ASHGA5P025670	0.000635973	0.017037535	2.2520048	up	noncoding
ASHGA5P042197	0.018830222	0.083422655	2.789138	up	noncoding
ASHGA5P042197	0.018830222	0.083422655	2.789138	up	noncoding
ASHGA5P030534	0.001680394	0.027439569	2.5835416	up	noncoding
ASHGA5P014191	0.02224917	0.092228003	2.0231116	up	noncoding
ASHGA5P039618	0.004222777	0.041294942	2.194141	up	noncoding
ASHGA5P039278	0.00281339	0.03419731	2.022336	up	noncoding
ASHGA5P019434	0.001911125	0.02921028	2.4731443	up	noncoding
ASHGA5P019434	0.001911125	0.02921028	2.4731443	up	noncoding
ASHGA5P048185	0.000285994	0.012492602	3.5032379	up	noncoding
ASHGA5P048185	0.000285994	0.012492602	3.5032379	up	noncoding
ASHGA5P048185	0.000285994	0.012492602	3.5032379	up	noncoding
ASHGA5P049224	0.00077113	0.018599313	18.0466494	up	noncoding
ASHGA5P053397	0.00225597	0.031214119	3.3583506	up	noncoding
ASHGA5P053397	0.00225597	0.031214119	3.3583506	up	noncoding
ASHGA5P014621	0.000101942	0.009142535	4.0173029	up	noncoding
ASHGA5P014621	0.000101942	0.009142535	4.0173029	up	noncoding
ASHGA5P014621	0.000101942	0.009142535	4.0173029	up	noncoding
ASHGA5P036933	0.012370774	0.068149498	2.3446608	up	noncoding
ASHGA5P038097	0.023051357	0.094014124	2.7029897	up	noncoding
ASHGA5P016309	0.016099042	0.07768932	2.7899708	up	noncoding
ASHGA5P016309	0.016099042	0.07768932	2.7899708	up	noncoding
ASHGA5P055227	0.014174401	0.07308451	2.308871	up	noncoding

ENST00000456944	RP11-82L18.2	GENCODE	583	chr9	-
uc003bcm.3	BC038245	UCSC_knowngene	1076	chr22	-
uc001uzl.3	BC025370	UCSC_knowngene	978	chr13	-
ENST00000553791	RP11-718G2.5	GENCODE	448	chr14	+
NR_040535	LOC100506472	RefSeq	3566	chr22	+
ENST00000568031	RP11-863P13.7	GENCODE	1313	chr16	-
DB308297		LincRNAs identified	526	chr8	-
ENST00000567422	RP11-23E10.4	pseudogene	509	chr16	-
NR_033999	LOC100129148	RefSeq	463	chr7	-
NR_033999	LOC100129148	RefSeq	463	chr7	-
NR_033999	LOC100129148	RefSeq	463	chr7	-
NR_033999	LOC100129148	RefSeq	463	chr7	-
ENST00000581019	CTD-2349P21.5	GENCODE	789	chr17	+
NR_040017	RNF157-AS1	RefSeq	2177	chr17	+
NR_040017	RNF157-AS1	RefSeq	2177	chr17	+
ENST00000435024	LINC00460	GENCODE	631	chr13	+
ENST00000567053	RP11-578F21.10	pseudogene	138	chr15	+
ENST00000523031	RP13-582O9.5	GENCODE	689	chr8	-
ENST00000463567	ZNF767	GENCODE	3520	chr7	-
ENST00000562920	RP11-23P13.6	GENCODE	400	chr15	+
TCONS_00022963	XLOC_010798	LincRNAs identified	257	chr14	+
ENST00000478666	FAM86DP	GENCODE	1068	chr3	-
uc004aus.1	BC042913	UCSC_knowngene	1164	chr9	+
chr3:45182150-4519	chr3:45182150-4519	LincRNAs identified	14250	chr3	+
uc010bmd.3	AGSK1	UCSC_knowngene	2405	chr15	+
ENST00000571970	RP11-95P2.1	GENCODE	753	chr16	-
ENST00000569313	CTD-2636A23.2	GENCODE	643	chr5	+
ENST00000569313	CTD-2636A23.2	GENCODE	643	chr5	+
ENST00000542112	SNHG1	GENCODE	780	chr11	-
ENST00000542112	SNHG1	GENCODE	780	chr11	-
ENST00000542112	SNHG1	GENCODE	780	chr11	-
ENST00000429648	RP11-21N7.2	GENCODE	301	chr1	-
ENST00000429648	RP11-21N7.2	GENCODE	301	chr1	-
ENST00000429648	RP11-21N7.2	GENCODE	301	chr1	-
ENST00000429648	RP11-21N7.2	GENCODE	301	chr1	-
ENST00000429648	RP11-21N7.2	GENCODE	301	chr1	-
ENST00000397644	RP11-513G11.1	GENCODE	416	chr3	-
ENST00000514752	SLC7A11-AS1	GENCODE	764	chr4	+
ENST00000437601	RP4-781K5.8	GENCODE	3574	chr1	+
uc001nhf.1	LOC646813	UCSC_knowngene	609	chr11	+
uc003qxa.1	AK128656	UCSC_knowngene	2322	chr6	-
uc003qxa.1	AK128656	UCSC_knowngene	2322	chr6	-
ENST00000558593	CTD-3110H11.2	GENCODE	507	chr15	+
ENST00000578492	RP13-104F24.2	GENCODE	2732	chr17	-
ENST00000514727	PPP1R14BP3	pseudogene	442	chr4	-
uc021xjv.1	BC040733	UCSC_knowngene	1061	chr3	+
ENST00000484076	RP11-18H7.1	GENCODE	969	chr3	+
ENST00000484076	RP11-18H7.1	GENCODE	969	chr3	+
ENST00000545308	SNHG1	GENCODE	646	chr11	-
ENST00000545308	SNHG1	GENCODE	646	chr11	-
ENST00000545308	SNHG1	GENCODE	646	chr11	-
ENST00000558031	CRNDE	GENCODE	464	chr16	-
ENST00000412059	GAS5	GENCODE	979	chr1	-
ENST00000412059	GAS5	GENCODE	979	chr1	-
NR_024533	ALG3	RefSeq	1450	chr3	-
NR_024533	ALG3	RefSeq	1450	chr3	-
NR_024533	ALG3	RefSeq	1450	chr3	-
NR_033917	LOC728228	RefSeq	692	chr20	+
NR_027007	LOC440944	RefSeq	1890	chr3	-
ENST00000429139	MIR181A2HG	GENCODE	662	chr9	+
ENST00000429139	MIR181A2HG	GENCODE	662	chr9	+
NR_046986	KIRREL3-AS2	RefSeq	500	chr11	+

91703518	91724213		CTGGTGACCTTTCTACTGATTGCAT		intron sense-overlapp
42760534	42765180		GTTTTCAGGTTTCTGCCTGACATTT		intergenic
44974385	44980021		TGCTTTTGAACGTGAAAGGAGAAA		intergenic
70742012	70758576		AATCTGGAATATTTCAAGATAAGGT		intergenic
39828164	39833133		GCAAAGATTTAA	100506472	exon sense-overlappi
88210903	88212216		TAATATCCTGATTTCAGTGGACTTG		intergenic
90904271	90904797		CATTTTGAGTGTTGTTGCAGTATTT		intergenic
33365496	33373402		GCTCCTGAAGTTCTTGTTTGTGTTGGACTGCTGGGTTT		
139102208	139112272		CTCTGGACCTGG	100129148	natural antisense
139102208	139112272		CTCTGGACCTGG	100129148	natural antisense
139102208	139112272		CTCTGGACCTGG	100129148	natural antisense
139102208	139112272		CTCTGGACCTGG	100129148	natural antisense
29056486	29057409		AGTTAATGTTCGGATCAACATCATT		intergenic
74136636	74150729		GTCAGGAACACC	100507218	natural antisense
74136636	74150729		GTCAGGAACACC	100507218	natural antisense
107028910	107030565		GACGAGGCATGT	728192	intergenic
29033747	29037148		AATATAAACATGTATGCAAACGAGCTTGTCAGAGAGC		
144362330	144363019		ATCACAAGTCTGTTAAGGTTGGAGA		intergenic
149244244	149321818		GTCCCCTAAGAC	79970	intergenic
42187382	42188141		GTGTGATGTTCTCAACGGCAGCTAA		intergenic
36690499	36691569		TTGAGAGCAGGAACTGATAACCTCT		intergenic
75471569	75484197	ENST00000489609,	TCATCAAAAAGA	692099	intergenic
97317350	97330409	ENST00000452148,	GAGAGGAAGAAGTTACATTGTCGT		natural antisense
45207146	45221396		AAGATGTTTGGAAGAAAACATGAC		intergenic
82944792	82975798	ENST00000335068,	AAAGACACCTGT	80154	intergenic
4296374	4301499	NR_039999	TTGCCTGTTCTAGAAGCTTGTATCA		intergenic
43289496	43290941		ATTGGAGATTAGTACCTCTGCTTTC		natural antisense
43289496	43290941		ATTGGAGATTAGTACCTCTGCTTTC		natural antisense
62619729	62623386		AGATTCGTTCTC	23642	bidirectional
62619729	62623386		AGATTCGTTCTC	23642	bidirectional
62619729	62623386		AGATTCGTTCTC	23642	bidirectional
155279828	155280381		TTTTCTTGGGCATAAACATCTGAAT		intronic antisense
155279828	155280381		TTTTCTTGGGCATAAACATCTGAAT		natural antisense
155279828	155280381		TTTTCTTGGGCATAAACATCTGAAT		natural antisense
155279828	155280381		TTTTCTTGGGCATAAACATCTGAAT		natural antisense
155279828	155280381		TTTTCTTGGGCATAAACATCTGAAT		natural antisense
194019234	194023820	ENST00000414120,	GTGCTATGCCGCGTCAAAAGAGTAT		intergenic
139026872	139090561		CTGTGTCTTCTCA	641364	natural antisense
234861147	234867390	NR_038856	CCAACAATCATTCTTTCTCGCATAT		intergenic
50368317	50379803		GAGACATTAATG	646813	intergenic
170060912	170063234		CCTTTAAACTTGACGGATGCATTTA		exon sense-overlappi
170060912	170063234		CCTTTAAACTTGACGGATGCATTTA		exon sense-overlappi
50648667	50659636	NR_024490, uc001z	TGGAATGGGACTAGAGAAGCAATT		intergenic
62745780	62777744	ENST00000400873	GACATACCAGCTTTCTCCCTTTGTC		intergenic
140036086	140036528	NM_001031801, NM	ATTTCTGGTCTGCTGGACAAGATCCGGGGCATGCAGA		
197371037	197373038		GCAATCTACTTATGTCCAAGGTGAA		intergenic
119813741	119855630		ACAGAACAAAGTGAGTGGAGGACA		bidirectional
119813741	119855630		ACAGAACAAAGTGAGTGGAGGACA		bidirectional
62621913	62622960		TATGAATAAGCT	23642	bidirectional
62621913	62622960		TATGAATAAGCT	23642	bidirectional
62621913	62622960		TATGAATAAGCT	23642	bidirectional
54952965	54957691		TTTTTCTTTTTAA	643911	intergenic
173833038	173837125	ENST00000454813,	TTATCCTAAACT	60674	bidirectional
173833038	173837125	ENST00000454813,	TTATCCTAAACT	60674	bidirectional
183960116	183966759		AGGGTGGCATGT	10195	exon sense-overlappi
183960116	183966759		AGGGTGGCATGT	10195	exon sense-overlappi
183960116	183966759		AGGGTGGCATGT	10195	natural antisense
4173736	4176600		GATTCTCCGTGA	728228	intergenic
9430536	9439174		GAAAATATGAAA	440944	bidirectional
127420745	127460910		GTTCAGGACTACTTCGGTTCTTTTA		intronic antisense
127420745	127460910		GTTCAGGACTACTTCGGTTCTTTTA		intronic antisense
126810641	126814986		GCCTTGTCCCTAC	100874251	intronic antisense

NM_016848	SHC3	SHC-transforming pr	-	91620685	91793682
NM_153497	TAB1	TGF-beta-activated k	+	39795758	39833132

[AGGAGTTATTCTTTTTATCTG

NM_001244584	C7orf55-LUC7L2	C7orf55-LUC7L2 pr	+	139025877	139108203
NM_001244585	LUC7L2	putative RNA-bindin	+	139025104	139108203
NM_001270643	LUC7L2	putative RNA-bindin	+	139044591	139108203
NM_016019	LUC7L2	putative RNA-bindin	+	139044591	139108203
NM_001454	FOXJ1	forkhead box protein	-	74132414	74137380
NM_052916	RNF157	RING finger protein	-	74138533	74236390

AATTGAAAAGCTGCAGGCGGGTG

NM_003837	FBP2	fructose-1,6-bisphosp	-	97321002	97356075
NM_001098272	HMGCS1	hydroxymethylglutar	-	43287571	43313614
NM_002130	HMGCS1	hydroxymethylglutar	-	43287571	43313614
NM_001012662	SLC3A2	4F2 cell-surface anti	+	62623483	62656355
NM_001012664	SLC3A2	4F2 cell-surface anti	+	62623483	62656355
NM_002394	SLC3A2	4F2 cell-surface anti	+	62623483	62656355
NM_001242825	FDPS	farnesyl pyrophospha	+	155278538	155290457
NM_001135821	FDPS	farnesyl pyrophospha	+	155278704	155290457
NM_001135822	FDPS	farnesyl pyrophospha	+	155278538	155290457
NM_001242824	FDPS	farnesyl pyrophospha	+	155278538	155290457
NM_002004	FDPS	farnesyl pyrophospha	+	155278538	155290457
NM_014331	SLC7A11	cystine/glutamate tra	-	139085247	139163503
NM_001202550	WDR27	WD repeat-containin	-	169857302	170102159
NM_182552	WDR27	WD repeat-containin	-	169857302	170102159

AGCTGAGCACACCCCAGAAGAAG

NM_001146156	GSK3B	glycogen synthase ki	-	119540801	119813264
NM_002093	GSK3B	glycogen synthase ki	-	119540801	119813264
NM_001012662	SLC3A2	4F2 cell-surface anti	+	62623483	62656355
NM_001012664	SLC3A2	4F2 cell-surface anti	+	62623483	62656355
NM_002394	SLC3A2	4F2 cell-surface anti	+	62623483	62656355
NM_001122770	ZBTB37	zinc finger and BTB	+	173837492	173855774
NM_032522	ZBTB37	zinc finger and BTB	+	173837492	173842778
NM_001006941	ALG3	dol-P-Man:Man(5)G	-	183960116	183967313
NM_005787	ALG3	dol-P-Man:Man(5)G	-	183960116	183966759
NM_138345	VWA5B2	von Willebrand facto	+	183948316	183960117
NM_001080517	SETD5	SET domain-contain	+	9439402	9519838
NM_001489	NR6A1	nuclear receptor subf	-	127284702	127533576
NM_033334	NR6A1	nuclear receptor subf	-	127284702	127533576
NM_001161707	KIRREL3	kin of IRRE-like prot	-	126300836	126870766

1040.651992	211.75648	9.411789	8.085632	1503.1417	1941.9781
150.935791	40.640862	6.751616	5.643213	216.51558	119.9591
1422.246783	260.152998	10.002752	8.150192	1342.7349	1159.7252
52.063556	11.952199	5.241928	3.765411	37.105717	16.6258
25.603438	7.600985	4.158084	2.880319	49.97546	8.55285
123.800505	16.928058	6.383766	4.230833	126.7496	128.78178
471.473488	78.570577	8.031843	6.394845	194.03473	91.10134
842.06797	216.989242	9.316395	8.110485	900.4693	915.2186
502.63212	34.816228	7.772775	5.18903	798.4984	1438.778
502.63212	34.816228	7.772775	5.18903	798.4984	1438.778
502.63212	34.816228	7.772775	5.18903	798.4984	1438.778
502.63212	34.816228	7.772775	5.18903	798.4984	1438.778
91.553634	23.225485	6.13149	4.817703	101.04339	120.5165
113.998215	21.305172	6.204566	4.416228	68.9523	18.64984
113.998215	21.305172	6.204566	4.416228	68.9523	18.64984
112.707217	16.030412	5.880828	3.900306	83.87451	12.779148
511.323123	138.989773	8.509151	7.430324	407.71472	542.5382
266.455152	51.15093	7.671084	5.879109	233.65384	235.82794
2176.22405	633.309688	10.5235	9.325353	2239.501	2493.2463
162.173762	32.062348	6.817402	5.07863	161.82901	170.5464
18.075022	6.594224	3.834393	2.806005	22.226925	23.239782
641.642827	176.73412	8.859587	7.814834	637.851	890.23083
2773.171233	375.460775	10.917862	8.787447	3267.0154	2529.036
155.788733	43.326232	6.925077	5.569765	186.4678	166.85725
10112.111	2653.818683	12.820762	11.723066	9979.395	13258.532
64.46809	25.561374	5.665127	4.392466	62.89299	47.736553
48.37697	15.90732	5.23074	3.966758	45.539516	28.361303
48.37697	15.90732	5.23074	3.966758	45.539516	28.361303
24.93385	5.735289	4.296158	2.712357	28.271288	26.968954
24.93385	5.735289	4.296158	2.712357	28.271288	26.968954
24.93385	5.735289	4.296158	2.712357	28.271288	26.968954
191.367452	57.330887	7.217238	5.836311	196.58112	221.04942
191.367452	57.330887	7.217238	5.836311	196.58112	221.04942
191.367452	57.330887	7.217238	5.836311	196.58112	221.04942
191.367452	57.330887	7.217238	5.836311	196.58112	221.04942
191.367452	57.330887	7.217238	5.836311	196.58112	221.04942
57.235961	10.778695	5.406198	3.542563	63.462086	25.170422
18.294802	6.004981	3.833503	2.806415	17.146624	25.98442
209.331521	19.400347	6.967446	4.528857	325.38428	309.40436
764.017777	200.162077	9.11911	7.9479	875.5744	1007.7526
26.114081	6.979532	4.252647	2.772827	56.0507	23.607018
26.114081	6.979532	4.252647	2.772827	56.0507	23.607018
232.301277	51.70712	7.341661	5.972311	269.96786	461.26532
1073.793818	357.539752	9.633754	8.617178	1201.2804	923.54346
5222.738533	1292.158433	11.829721	10.696065	6122.101	6997.644
22.639128	8.343019	4.134003	3.117981	20.916672	13.347911
493.538982	118.236518	8.465878	7.159532	362.11575	380.01108
493.538982	118.236518	8.465878	7.159532	362.11575	380.01108
390.994727	69.202001	8.209996	6.401307	421.19617	314.16855
390.994727	69.202001	8.209996	6.401307	421.19617	314.16855
390.994727	69.202001	8.209996	6.401307	421.19617	314.16855
1573.854447	53.787365	9.974668	5.801009	1218.1853	308.80548
11626.30567	1978.552317	13.027916	11.280163	11000.318	8719.194
11626.30567	1978.552317	13.027916	11.280163	11000.318	8719.194
95.36472	15.402231	6.215373	4.209145	92.93633	106.73032
95.36472	15.402231	6.215373	4.209145	92.93633	106.73032
95.36472	15.402231	6.215373	4.209145	92.93633	106.73032
210.57565	41.233394	6.787628	5.558248	339.56528	592.112
906.041238	281.448288	9.415469	7.980913	862.0877	690.8251
107.068955	21.603293	6.119022	4.638772	142.37143	213.09825
107.068955	21.603293	6.119022	4.638772	142.37143	213.09825
986.085578	245.662301	9.345168	8.13798	1042.53	1134.4802

1021.743	357.96695	689.1698	729.9124	193.81013	252.10959
275.2881	98.82596	89.72919	105.296814	31.666756	32.860043
1450.8539	730.6404	1992.195	1857.3313	243.50214	87.124115
56.78644	72.52953	55.99419	73.33966	7.3367887	5.6573777
39.449646	23.634468	15.365139	16.643068	5.297188	4.9999995
208.4493	29.283642	127.43228	122.10643	10.13496	8.8325205
284.01874	1116.9996	301.74188	840.94464	81.88325	23.646336
750.12006	905.2151	640.9992	940.38556	175.11067	169.73965
95.98184	388.36432	47.263153	246.90701	54.34106	67.18685
95.98184	388.36432	47.263153	246.90701	54.34106	67.18685
95.98184	388.36432	47.263153	246.90701	54.34106	67.18685
95.98184	388.36432	47.263153	246.90701	54.34106	67.18685
74.458725	61.851963	84.85659	106.594635	24.59682	16.757032
106.8888	152.20961	146.06381	191.22493	8.788076	5.0068173
106.8888	152.20961	146.06381	191.22493	8.788076	5.0068173
122.3504	27.099625	232.26389	197.87573	6.5741005	6.265749
228.15108	802.3153	390.82224	696.3972	148.03731	100.84601
226.68108	359.29953	219.62292	323.6456	44.51372	24.702955
1522.0673	720.7289	3221.4082	2860.3926	748.6003	258.0758
158.67865	46.15737	243.43318	192.39796	23.170609	6.503676
17.869669	17.923971	12.02822	15.161568	4.9999995	4.9999995
337.41165	423.08868	823.4283	737.8465	172.866	189.49434
3473.046	1025.6407	3352.3	2991.9893	307.31564	199.435
158.35538	142.7472	136.62657	143.6782	43.844856	25.722551
6101.351	8154.715	10962.267	12216.406	2694.738	1354.8893
75.09598	67.12277	59.180332	74.779915	35.22195	5.083777
57.511875	44.565125	50.12643	64.15757	14.364501	4.9999995
57.511875	44.565125	50.12643	64.15757	14.364501	4.9999995
22.146978	17.410955	26.63162	28.173307	4.9999995	6.2616725
22.146978	17.410955	26.63162	28.173307	4.9999995	6.2616725
22.146978	17.410955	26.63162	28.173307	4.9999995	6.2616725
158.54224	187.85191	175.20741	208.97261	74.09805	21.545378
158.54224	187.85191	175.20741	208.97261	74.09805	21.545378
158.54224	187.85191	175.20741	208.97261	74.09805	21.545378
158.54224	187.85191	175.20741	208.97261	74.09805	21.545378
158.54224	187.85191	175.20741	208.97261	74.09805	21.545378
92.33842	71.02013	35.45313	55.971577	7.8770094	7.4456563
13.956819	14.043661	18.033058	20.60423	4.9999995	5.331788
305.94382	174.48065	25.313812	115.462204	19.843748	24.812908
590.84796	388.4696	848.4497	873.0124	240.38818	157.749
19.380522	21.155582	14.611749	21.878918	4.9999995	4.9999995
19.380522	21.155582	14.611749	21.878918	4.9999995	4.9999995
108.36612	202.7969	140.97333	210.43813	59.50132	58.95743
843.41815	710.5013	1458.0734	1305.9462	337.94202	136.6342
4200.414	2211.3608	6273.5674	5531.344	1172.5321	816.7846
25.432402	37.71492	14.899751	23.52311	5.9845214	4.9999995
347.18134	877.90076	333.6875	660.33746	100.7786	55.45244
347.18134	877.90076	333.6875	660.33746	100.7786	55.45244
484.92905	270.52847	403.87457	451.27155	53.086838	56.439415
484.92905	270.52847	403.87457	451.27155	53.086838	56.439415
484.92905	270.52847	403.87457	451.27155	53.086838	56.439415
2104.7295	2386.8716	1235.8998	2188.635	44.070038	35.95779
11679.888	17205.934	7037.106	14115.394	1422.5919	1128.1028
11679.888	17205.934	7037.106	14115.394	1422.5919	1128.1028
74.03448	99.34806	80.78832	118.35081	15.423068	13.1439
74.03448	99.34806	80.78832	118.35081	15.423068	13.1439
74.03448	99.34806	80.78832	118.35081	15.423068	13.1439
51.73742	102.41029	72.17394	105.45497	52.242012	65.92103
1000.86487	969.14996	763.2176	1150.1022	411.345	92.06969
25.737349	65.92589	93.35802	101.92279	24.017742	19.252762
25.737349	65.92589	93.35802	101.92279	24.017742	19.252762
498.95593	323.10864	1727.0693	1190.3694	299.50467	160.82173

175.55469	214.29091	247.46797	187.30559	10.086569	10.504383
40.011024	64.30053	35.58702	39.4198	7.3067083	6.679782
403.1685	117.89281	464.65155	244.57887	9.927773	9.827123
12.740811	15.507429	16.194138	14.27665	4.7482605	4.0658145
16.344902	4.9999995	4.9999995	8.963822	5.1800513	3.2088838
15.543469	34.080894	11.548129	21.428375	6.5360494	6.7782764
157.46921	54.14872	99.268036	55.007908	7.1501884	6.3026276
205.97876	258.00485	287.2007	205.90082	9.342271	9.502642
17.831673	35.56803	11.21361	22.756147	9.178617	10.115532
17.831673	35.56803	11.21361	22.756147	9.178617	10.115532
17.831673	35.56803	11.21361	22.756147	9.178617	10.115532
17.831673	35.56803	11.21361	22.756147	9.178617	10.115532
26.089176	30.093754	25.03811	16.778019	6.209666	6.6858573
28.700191	36.832615	26.438053	22.065277	5.649657	4.210272
28.700191	36.832615	26.438053	22.065277	5.649657	4.210272
45.815857	15.130148	9.094078	13.302541	5.938537	3.7204206
195.5508	118.07882	157.6051	113.8206	8.211056	8.774062
84.48712	38.842094	73.620735	40.738956	7.415809	7.6201086
1728.8364	308.3771	468.768	287.20053	10.6541	10.861384
39.948444	53.006626	31.318022	38.426712	6.882111	7.1775985
9.513489	4.9999995	8.406435	6.645421	4.0114455	4.4967422
153.96652	154.0975	229.26135	160.71901	8.843602	9.461089
506.13968	201.67282	661.27094	376.93057	11.196217	10.876938
99.352196	31.859398	25.67859	33.4998	7.089572	7.141159
4122.705	2390.3896	3055.697	2304.4932	12.800134	13.251185
73.3053	12.27902	17.569958	9.908241	5.518408	5.449081
26.870548	9.52076	23.046412	16.641699	5.050141	4.768037
26.870548	9.52076	23.046412	16.641699	5.050141	4.768037
4.9999995	5.1417427	8.008319	4.9999995	4.368171	4.6998987
4.9999995	5.1417427	8.008319	4.9999995	4.368171	4.6998987
4.9999995	5.1417427	8.008319	4.9999995	4.368171	4.6998987
144.62715	29.651913	39.1774	34.88543	7.1689568	7.5320888
144.62715	29.651913	39.1774	34.88543	7.1689568	7.5320888
144.62715	29.651913	39.1774	34.88543	7.1689568	7.5320888
144.62715	29.651913	39.1774	34.88543	7.1689568	7.5320888
144.62715	29.651913	39.1774	34.88543	7.1689568	7.5320888
11.90386	20.501863	4.9999995	11.943779	5.530241	4.607878
4.9999995	6.794838	5.2745876	8.628676	3.6272967	4.6513805
16.940948	28.21457	10.507231	16.082674	7.886812	7.9993567
297.7907	142.5979	220.814	141.63268	9.305759	9.630097
13.78618	4.9999995	8.091012	4.9999995	5.3450427	4.518482
13.78618	4.9999995	8.091012	4.9999995	5.3450427	4.518482
64.91044	56.57136	42.226543	28.075628	7.618684	8.541501
865.67737	268.5383	305.7924	230.65422	9.755366	9.513567
1521.857	1066.3477	1866.3464	1309.0828	12.088738	12.3176
16.02713	8.894947	5.434771	8.716743	3.9282544	3.7777586
117.56249	134.13528	177.07051	124.419785	8.0386095	8.2795515
117.56249	134.13528	177.07051	124.419785	8.0386095	8.2795515
67.356674	53.167313	111.90309	73.258675	8.259554	8.019411
67.356674	53.167313	111.90309	73.258675	8.259554	8.019411
67.356674	53.167313	111.90309	73.258675	8.259554	8.019411
34.236214	133.33778	15.409161	59.713207	9.776578	7.9972873
1568.6355	2218.3577	3249.5735	2284.0525	12.955497	12.631751
1568.6355	2218.3577	3249.5735	2284.0525	12.955497	12.631751
18.085112	10.469611	20.095343	15.19635	6.090537	6.5200653
18.085112	10.469611	20.095343	15.19635	6.090537	6.5200653
18.085112	10.469611	20.095343	15.19635	6.090537	6.5200653
36.58815	50.427402	17.437988	24.78378	7.954182	8.896972
778.2206	82.68021	201.5145	122.85973	9.290684	9.107395
26.11518	10.566531	33.56497	16.102571	6.702884	7.480156
26.11518	10.566531	33.56497	16.102571	6.702884	7.480156
422.1397	122.961136	334.55637	133.9902	9.551316	9.796671

9.622358	8.159922	9.165044	8.932458	7.845251	8.8365755
7.768841	6.276408	6.279671	6.198286	5.216419	5.876034
10.12784	9.206102	10.661901	10.26577	8.185551	7.298137
5.5134783	5.8187313	5.620864	5.684422	3.067297	3.237613
4.98702	4.073824	3.86529	3.633437	2.6147757	2.4279919
7.38896	4.4154334	6.77054	6.4133368	3.5659158	3.9177082
7.813798	9.801983	7.9894986	9.13296	6.5800357	5.3954463
9.200793	9.502182	9.05618	9.294303	7.705233	8.253856
6.262135	8.281423	5.3946185	7.404324	6.000153	6.924452
6.262135	8.281423	5.3946185	7.404324	6.000153	6.924452
6.262135	8.281423	5.3946185	7.404324	6.000153	6.924452
6.262135	8.281423	5.3946185	7.404324	6.000153	6.924452
5.901005	5.5746965	6.2010074	6.21671	4.862563	4.883322
6.421521	6.923353	6.9702573	7.0523367	3.3477747	3.0492284
6.421521	6.923353	6.9702573	7.0523367	3.3477747	3.0492284
6.6129704	4.2974625	7.613503	7.102073	2.9126093	3.3989067
7.5105324	9.332134	8.357673	8.869449	7.447483	7.5075865
7.500532	8.16674	7.533904	7.7894106	5.7077007	5.4579983
10.20178	9.184741	11.361345	10.877653	9.851195	8.868647
6.9759765	5.1246614	7.683593	7.06047	4.7756405	3.4580486
3.8407645	3.6248217	3.5320184	3.5005684	2.3467326	2.4279919
8.0651655	8.41722	9.422249	8.948195	7.681485	8.421829
11.395753	9.68511	11.417775	10.935378	8.530296	8.499079
6.972909	6.8336496	6.8693295	6.6438427	5.6865883	5.5198627
12.213274	12.640489	13.083119	12.936368	11.715728	11.282167
5.9124017	5.699424	5.697114	5.7143316	5.372783	3.0758946
5.5315475	5.06874	5.4713063	5.4946685	4.0726523	2.4279919
5.5315475	5.06874	5.4713063	5.4946685	4.0726523	2.4279919
4.1564403	3.5771267	4.6145225	4.360787	2.3467326	3.398107
4.1564403	3.5771267	4.6145225	4.360787	2.3467326	3.398107
4.1564403	3.5771267	4.6145225	4.360787	2.3467326	3.398107
6.975276	7.23111	7.22584	7.1701584	6.43744	5.2606497
6.975276	7.23111	7.22584	7.1701584	6.43744	5.2606497
6.975276	7.23111	7.22584	7.1701584	6.43744	5.2606497
6.975276	7.23111	7.22584	7.1701584	6.43744	5.2606497
6.975276	7.23111	7.22584	7.1701584	6.43744	5.2606497
6.2071757	5.784296	4.99504	5.312555	3.1750174	3.6577806
3.4764614	3.2339826	4.082072	3.9298244	2.3467326	3.1480198
7.919527	7.1222258	4.54796	6.328794	4.545515	5.4637485
8.844283	8.282152	9.468268	9.184099	8.164367	8.151084
3.9550972	3.8944898	3.7963645	4.0064034	2.3467326	2.4279919
3.9550972	3.8944898	3.7963645	4.0064034	2.3467326	2.4279919
6.440709	7.346869	6.9201193	7.182083	6.13182	6.732543
9.368111	9.16518	10.229125	9.771173	8.6693	7.9418917
11.687833	10.783394	12.291324	11.809436	10.4938545	10.532251
4.356322	4.8093066	3.8252258	4.107153	2.7836428	2.4279919
8.100062	9.46065	8.126455	8.78994	6.889906	6.64497
8.100062	9.46065	8.126455	8.78994	6.889906	6.64497
8.571802	7.7596226	8.40225	8.247339	5.9658046	6.673623
8.571802	7.7596226	8.40225	8.247339	5.9658046	6.673623
8.571802	7.7596226	8.40225	8.247339	5.9658046	6.673623
10.6862755	10.880724	10.006871	10.500271	5.6945286	6.013834
13.18249	13.763353	12.464699	13.169705	10.769582	11.017909
13.18249	13.763353	12.464699	13.169705	10.769582	11.017909
5.894843	6.2844825	6.1341906	6.368118	4.17912	4.5157228
5.894843	6.2844825	6.1341906	6.368118	4.17912	4.5157228
5.894843	6.2844825	6.1341906	6.368118	4.17912	4.5157228
5.3757563	6.329414	5.969233	6.200208	5.942523	6.9000835
9.596456	9.602419	9.313969	9.58189	8.941606	7.3743935
4.3745246	5.671168	6.3342543	6.151148	4.829273	5.0921373
4.3745246	5.671168	6.3342543	6.151148	4.829273	5.0921373
8.61194	8.014741	10.459863	9.6364765	8.493663	8.177273

7.4080367	8.153408	8.207653	8.062866
5.319903	6.3073635	5.4279847	5.711576
8.612217	7.2367826	9.0998955	8.468567
3.7524185	4.0957503	4.290621	4.148764
4.0910144	2.3408759	2.3618152	3.4454384
4.0224533	5.3123045	3.8023777	4.764236
7.251147	6.035989	6.8887963	6.217654
7.6353045	8.441366	8.41455	8.212599
4.2084394	5.384291	3.7621272	4.8547153
4.2084394	5.384291	3.7621272	4.8547153
4.2084394	5.384291	3.7621272	4.8547153
4.2084394	5.384291	3.7621272	4.8547153
4.728639	5.1221275	4.916835	4.3927336
4.8657374	5.4349394	4.9928145	4.806872
4.8657374	5.4349394	4.9928145	4.806872
5.517534	4.0596166	3.4697275	4.0434446
7.5621157	7.239038	7.5286636	7.2970543
6.3679366	5.5157127	6.465309	5.759997
10.6966505	8.7208395	9.111173	8.703615
5.316615	6.003877	5.2436233	5.6739764
3.3620586	2.3408759	3.363959	2.994415
7.2148886	7.6431437	8.096182	7.831473
8.937437	8.056215	9.598484	9.103171
6.5937204	5.2045507	4.9528174	5.461048
11.985458	11.719634	11.847445	11.787963
6.170115	3.7339466	4.4078546	3.5942008
4.7704973	3.3477747	4.800864	4.3807707
4.7704973	3.3477747	4.800864	4.3807707
2.350709	2.5302544	3.2955859	2.352752
2.350709	2.5302544	3.2955859	2.352752
2.350709	2.5302544	3.2955859	2.352752
7.1342673	5.0994406	5.5615935	5.524474
7.1342673	5.0994406	5.5615935	5.524474
7.1342673	5.0994406	5.5615935	5.524474
7.1342673	5.0994406	5.5615935	5.524474
7.1342673	5.0994406	5.5615935	5.524474
3.6618996	4.51931	2.3618152	3.8795578
2.350709	2.8647172	2.7444959	3.3838146
4.1396093	5.021034	3.6750293	4.3282075
8.164114	7.5288925	8.038947	7.639994
3.8592794	2.3408759	3.309332	2.352752
3.8592794	2.3408759	3.309332	2.352752
6.002713	6.108493	5.6717625	5.186533
9.700099	8.50676	8.501679	8.383337
10.51125	10.572428	11.124279	10.942325
4.063998	3.252387	2.779829	3.4000354
6.838156	7.4346385	7.708908	7.440611
6.838156	7.4346385	7.708908	7.440611
6.0537663	6.009107	7.0620756	6.6434684
6.0537663	6.009107	7.0620756	6.6434684
6.0537663	6.009107	7.0620756	6.6434684
5.109831	7.424783	4.2188616	6.344214
10.553689	11.616222	11.9475	11.776076
10.553689	11.616222	11.9475	11.776076
4.231201	3.492522	4.5930915	4.2432156
4.231201	3.492522	4.5930915	4.2432156
4.231201	3.492522	4.5930915	4.2432156
5.1957746	5.928275	4.395814	4.98702
9.551316	6.6970463	7.9045277	7.4165874
4.7294884	3.5078642	5.3424053	4.3314667
4.7294884	3.5078642	5.3424053	4.3314667
8.675404	7.3042655	8.619994	7.5572824

ASHGA5P055227	0.014174401	0.07308451	2.308871	up	noncoding
ASHGA5P052791	1.73181E-06	0.002141141	2.3389504	up	noncoding
ASHGA5P006992	2.23046E-05	0.005043652	2.5624044	up	noncoding
ASHGA5P017466	0.027582768	0.103745399	2.5959087	up	noncoding
ASHGA5P000098	0.036741272	0.121643972	2.0922378	up	noncoding
ASHGA5P037175	0.023286972	0.094538063	2.0978609	up	noncoding
ASHGA5P048066	0.034959566	0.118302079	2.0187558	up	noncoding
ASHGA5P048066	0.034959566	0.118302079	2.0187558	up	noncoding
ASHGA5P048066	0.034959566	0.118302079	2.0187558	up	noncoding
ASHGA5P048066	0.034959566	0.118302079	2.0187558	up	noncoding
ASHGA5P054139	0.016170767	0.077740057	2.009129	up	noncoding
ASHGA5P023469	0.00145462	0.025519097	2.8726983	up	noncoding
ASHGA5P022759	0.001567153	0.02659241	4.199494	up	noncoding
ASHGA5P046564	0.003044364	0.03574339	4.3678983	up	noncoding
ASHGA5P038419	0.004102356	0.040926621	5.0955087	up	noncoding
ASHGA5P021955	0.003805208	0.039493479	2.5972844	up	noncoding
ASHGA5P021955	0.003805208	0.039493479	2.5972844	up	noncoding
ASHGA5P029459	0.03570412	0.119652008	2.2164791	up	noncoding
ASHGA5P029459	0.03570412	0.119652008	2.2164791	up	noncoding
ASHGA5P029459	0.03570412	0.119652008	2.2164791	up	noncoding
ASHGA5P025926	0.007127347	0.053290385	2.5206135	up	noncoding
ASHGA5P025926	0.007127347	0.053290385	2.5206135	up	noncoding
ASHGA5P000062	0.00251199	0.032544932	2.6272492	up	noncoding
ASHGA5P018969	0.024808312	0.097770279	3.0249263	up	noncoding
ASHGA5P018969	0.024808312	0.097770279	3.0249263	up	noncoding
ASHGA5P018969	0.024808312	0.097770279	3.0249263	up	noncoding
ASHGA5P018969	0.024808312	0.097770279	3.0249263	up	noncoding
ASHGA5P018969	0.024808312	0.097770279	3.0249263	up	noncoding
ASHGA5P018969	0.024808312	0.097770279	3.0249263	up	noncoding
ASHGA5P018969	0.024808312	0.097770279	3.0249263	up	noncoding
ASHGA5P018969	0.024808312	0.097770279	3.0249263	up	noncoding
ASHGA5P018969	0.024808312	0.097770279	3.0249263	up	noncoding
ASHGA5P030066	0.026066529	0.100598785	2.8944209	up	noncoding
ASHGA5P050681	0.030833452	0.110301777	3.5301728	up	noncoding
ASHGA5P023235	0.004229569	0.04130748	2.1081891	up	noncoding
ASHGA5P031379	0.01592209	0.077147596	3.8693967	up	noncoding
ASHGA5P031078	0.029618265	0.107770137	3.9005455	up	noncoding
ASHGA5P037297	0.002019053	0.029606317	4.017933	up	noncoding
ASHGA5P053474	0.011740245	0.066707739	3.9089815	up	noncoding
ASHGA5P027285	0.041193939	0.129607517	2.802375	up	noncoding
ASHGA5P029243	0.002579848	0.032855824	2.0079173	up	noncoding
ASHGA5P054526	0.000525905	0.015826428	2.1034483	up	noncoding
ASHGA5P044075	0.037439047	0.122766907	2.0669376	up	noncoding
ASHGA5P054404	0.004357079	0.041854505	2.035865	up	noncoding
ASHGA5P031047	0.000396697	0.014216192	3.4052334	up	noncoding
ASHGA5P031047	0.000396697	0.014216192	3.4052334	up	noncoding
ASHGA5P021447	0.010277075	0.062647807	3.2131948	up	noncoding
ASHGA5P018116	9.90834E-05	0.009080764	2.514708	up	noncoding
ASHGA5P037062	0.004388664	0.041991917	2.1547889	up	noncoding
ASHGA5P023010	0.001467631	0.025593099	2.3856369	up	noncoding
ASHGA5P040796	0.003274786	0.036994062	2.6335498	up	noncoding
ASHGA5P041570	0.01732642	0.080081956	2.8256161	up	noncoding
ASHGA5P014167	0.023370236	0.094712107	2.0112535	up	noncoding
ASHGA5P053820	0.008978393	0.059181641	3.4436344	up	noncoding
ASHGA5P053820	0.008978393	0.059181641	3.4436344	up	noncoding
ASHGA5P052517	0.021783837	0.091164516	4.7145691	up	noncoding
ASHGA5P029211	0.014825133	0.074487146	2.3607025	up	noncoding
ASHGA5P029097	0.004126121	0.041046245	3.8116125	up	noncoding
ASHGA5P036778	0.00043846	0.014679519	3.2833108	up	noncoding
ASHGA5P021811	0.000603925	0.016566293	4.364568	up	noncoding
ASHGA5P031856	0.002265129	0.031214119	2.4708826	up	noncoding
ASHGA5P046097	0.021593674	0.090652411	3.0041508	up	noncoding

NR_046986	KIRREL3-AS2	RefSeq	500	chr11	+
ENST00000433843	SNHG5	GENCODE	388	chr6	-
NR_036658	ZNFX1-AS1	RefSeq	959	chr20	+
ENST00000442010	HMGB3P22	GENCODE	597	chr5	+
BC042589		RNAdb	3223	chr6	+
ENST00000449500	CTD-3184A7.4	GENCODE	801	chr20	+
ENST00000532022	RP11-390K5.3	GENCODE	332	chr11	-
ENST00000532022	RP11-390K5.3	GENCODE	332	chr11	-
ENST00000532022	RP11-390K5.3	GENCODE	332	chr11	-
ENST00000532022	RP11-390K5.3	GENCODE	332	chr11	-
ENST00000438602	RP11-308D16.4	GENCODE	582	chrX	+
NR_034100	LOC400643	RefSeq	1751	chr18	-
ENST00000562298	RP11-368I7.2	GENCODE	931	chr16	+
ENST00000561542	RP11-305E6.4	GENCODE	3241	chr10	-
ENST00000509201	RP11-93K22.6	GENCODE	466	chr3	-
ENST00000545424	RP11-695J4.2	GENCODE	475	chr12	+
ENST00000545424	RP11-695J4.2	GENCODE	475	chr12	+
ENST00000555294	RP11-298I3.4	GENCODE	589	chr14	+
ENST00000555294	RP11-298I3.4	GENCODE	589	chr14	+
ENST00000555294	RP11-298I3.4	GENCODE	589	chr14	+
NR_046402	POLD1	RefSeq	3361	chr19	+
NR_046402	POLD1	RefSeq	3361	chr19	+
AK124533		NRED	4378	chr9	-
NR_033746	DMKN	RefSeq	1054	chr19	-
NR_033746	DMKN	RefSeq	1054	chr19	-
NR_033746	DMKN	RefSeq	1054	chr19	-
NR_033746	DMKN	RefSeq	1054	chr19	-
NR_033746	DMKN	RefSeq	1054	chr19	-
NR_033746	DMKN	RefSeq	1054	chr19	-
NR_033746	DMKN	RefSeq	1054	chr19	-
NR_033746	DMKN	RefSeq	1054	chr19	-
NR_033746	DMKN	RefSeq	1054	chr19	-
TCONS_00008011	XLOC_003475	LincRNAs identified	413	chr4	+
uc002tyw.3	BC032407	UCSC_knowngene	1746	chr2	-
ENST00000575202	CTD-2377D24.8	GENCODE	618	chr17	+
ENST00000563280	RP11-463O9.5	GENCODE	319	chr16	-
ENST00000457405	RP1-10C16.1	GENCODE	418	chr1	-
ENST00000457669	AP000696.2	GENCODE	1133	chr21	-
ENST00000518880	RP11-363E6.3	GENCODE	620	chr8	-
TCONS_00011758	XLOC_005220	LincRNAs identified	319	chr6	+
ENST00000556072	RP11-488C13.5	GENCODE	1677	chr14	-
uc001kfm.3	BC069782	UCSC_knowngene	1227	chr10	+
NR_026817	LOC148696	RefSeq	4241	chr1	+
TCONS_00010945	XLOC_004859	LincRNAs identified	387	chr5	-
ENST00000567108	CTD-3203P2.1	GENCODE	1684	chr16	-
ENST00000567108	CTD-3203P2.1	GENCODE	1684	chr16	-
ENST00000534477	CTC-497E21.4	GENCODE	888	chr11	-
ENST00000449614	AC005077.5	pseudogene	213	chr7	+
NR_034009	SRSF6	RefSeq	4023	chr20	+
ENST00000567665	RP11-345M22.1	GENCODE	416	chr16	+
ENST00000414422	RP11-277L2.2	GENCODE	769	chr1	+
TCONS_00020532	XLOC_009846	LincRNAs identified	614	chr12	+
ENST00000434828	FER1L4	GENCODE	1964	chr20	-
uc004bos.3	AB074162	UCSC_knowngene	2239	chr9	+
uc004bos.3	AB074162	UCSC_knowngene	2239	chr9	+
ENST00000510155	RP11-844P9.2	GENCODE	544	chr5	-
ENST00000554634	RP1-261D10.2	GENCODE	464	chr14	-
ENST00000556537	RP11-407N17.4	GENCODE	530	chr14	-
ENST00000439232	SNORA71B	GENCODE	136	chr20	-
ENST00000541383	RP11-173P15.3	GENCODE	575	chr12	-
TCONS_00006568	XLOC_003195	LincRNAs identified	243	chr3	-
ENST00000424415	RP11-706O15.5	GENCODE	2194	chrX	-

126810641	126814986		GCCTTGTCCCTAC	100874251	intronic antisense
86387046	86387917	ENST00000431043	AAGTTGCAACGATTTCTGTAAGTGG		intergenic
47894714	47905795	NR_036659	TATAAAATTGAA	441951	natural antisense
179106032	179121650		AAGAGTCCGGAATTTTCCAAGAAGT		natural antisense
26172304	26175512		GCAGCATTTGGGTGGAAGGACGTAC		intergenic
62258579	62260177		GTGTCATTTATCTGAGTTTGCCTATC		bidirectional
47144654	47152352		TTGGAAGCTTTTACAATAAAGATTT		intronic antisense
47144654	47152352		TTGGAAGCTTTTACAATAAAGATTT		intronic antisense
47144654	47152352		TTGGAAGCTTTTACAATAAAGATTT		intronic antisense
47144654	47152352		TTGGAAGCTTTTACAATAAAGATTT		intronic antisense
136075645	136103789		CCCCTAATTACATAATTTTAAGCAT		intergenic
6925472	6929868		CGAGGCTAAGGA	400643	intergenic
89782935	89784572		TTTTCCCTGCAAGCCATGGGCCTCT		natural antisense
30591754	30594995		CAGAACACTCTGATTTGGTTACATG		intergenic
129672947	129688137		CAATTAGAGGATTTGCTAGTACGTA		intergenic
14720683	14772232		CCTCTCCGAGTTTTGCGGCCCAAGG		natural antisense
14720683	14772232		CCTCTCCGAGTTTTGCGGCCCAAGG		natural antisense
23452106	23467632		CCTGAAGGAAAAGGAAACGTGGTAC		intronic antisense
23452106	23467632		CCTGAAGGAAAAGGAAACGTGGTAC		intronic antisense
23452106	23467632		CCTGAAGGAAAAGGAAACGTGGTAC		intronic antisense
50887579	50921275		TGGCCGAGAGGA	5424	exon sense-overlappi
50887579	50921275		TGGCCGAGAGGA	5424	exon sense-overlappi
32954488	32958868		GTCAAGTTACTTGCTCACAATCATA		intergenic
35988118	36001412		GCTGCAGTGGGT	93099	exon sense-overlappi
35988118	36001412		GCTGCAGTGGGT	93099	exon sense-overlappi
35988118	36001412		GCTGCAGTGGGT	93099	exon sense-overlappi
35988118	36001412		GCTGCAGTGGGT	93099	exon sense-overlappi
35988118	36001412		GCTGCAGTGGGT	93099	exon sense-overlappi
35988118	36001412		GCTGCAGTGGGT	93099	exon sense-overlappi
35988118	36001412		GCTGCAGTGGGT	93099	exon sense-overlappi
35988118	36001412		GCTGCAGTGGGT	93099	exon sense-overlappi
35988118	36001412		GCTGCAGTGGGT	93099	exon sense-overlappi
24667010	24670951		TTAAACATGTCAACATCTCAACAAT		intergenic
156877046	157111432	ENST00000428651	CTTGTTTCAGGTAATGTAACCAGAT		intergenic
46760728	46781844		TTCTCTACAGGTATTGACTGCTGCC		intergenic
86598750	86601367		ACTAGAAGGCAATGGGGCGTGCCAC		natural antisense
168732602	168743728		CATTCATTCACCATTGATGTATTTGT		intergenic
38004978	38009331		ATGGGTTTATCTTTTGGGACCAAGA		intergenic
82192105	82193681		CGGGAAGTGTGGTTTCTAAACTAAA		intronic antisense
27677988	27680876		TCCCAATGTTCTTGTCAGTGCACTTT		intergenic
77248082	77253067		CCCAGGAGGGGAGGAGGGGTTGCT		natural antisense
90667927	90669154		TATTATGAAGGTATTGTGGGTTTTC		exon sense-overlappi
207991723	207995941		ATACCCATCTCCC	148696	natural antisense
67804064	67814933		ATGTTGGCTTCATCCTCATGCCGGT		intergenic
27224630	27226314		AGACAACGTTTGTTATTATTACTGC		natural antisense
27224630	27226314		AGACAACGTTTGTTATTATTACTGC		natural antisense
13001757	13011086	ENST00000532541	AGCAGCATGCCAAAATTGGAAGAAC		intergenic
75803050	75804891	ENST00000437494	CATCATTCAGGTCACTGTCGATGCCTTGCCAACCTCTG		
42086503	42092244		AATCTGAACTAG	6431	exon sense-overlappi
79832430	79832987		TGAGAATCAAACAAAACCACGGTCA		intergenic
149605639	149633208	ENST00000418974,	ACGGGGATCCAGAAGGCGTTGTCGC		intergenic
96506540	96545912		TTTTATTTCACTCTCAAGTAATGGCT		intergenic
34146506	34152457	ENST00000400465,	ATCTCTGCCAATC	80307	intergenic
127420690	127422929		CTTACGTAACAGTTCAGGACTACTT		intronic antisense
127420690	127422929		CTTACGTAACAGTTCAGGACTACTT		intronic antisense
175600355	175602396		TCTTCCTCCCAGATACCAACAAGCC		intergenic
71773886	71778076		GAGACTGAAATGGAAGTCTCTTAGT		intergenic
39644088	39645084		CTAGGCCTTTTAACCTATGATGTAA		natural antisense
37053842	37053978		CGCTTCCCCGTGT	26776	intergenic
121134926	121137304		TCTCCCATACATTCCCTATGGTGGT		natural antisense
104536325	104626299		ATCTTTGGAGATTGGAGAGTCCACC		intergenic
3809478	3820041		CTCAGTACATCTTAATAAGGAATTA		intergenic

NM_032531	KIRREL3	kin of IRRE-like prot	-	126293395	126870766
NM_021035	ZNFX1	NFX1-type zinc fing	-	47862438	47894756
NM_001164444	CBY3	protein chibby homo	-	179105558	179107975
NM_012384	GMEB2	glucocorticoid modu	-	62218954	62258381
NM_001003676	C11orf49	UPF0705 protein C1	+	46958250	47183798
NM_001003677	C11orf49	UPF0705 protein C1	+	46958250	47183798
NM_001003678	C11orf49	UPF0705 protein C1	+	46958250	47185931
NM_024113	C11orf49	UPF0705 protein C1	+	46958250	47183798
NM_004913	C16orf7	uncharacterized prote	-	89773540	89787394
NM_004963	GUCY2C	heat-stable enterotox	-	14765567	14849519
NM_024829	PLBD1	phospholipase B-like	-	14656596	14720791
NM_001130706	C14orf93	uncharacterized prote	-	23456109	23479360
NM_001130708	C14orf93	uncharacterized prote	-	23456109	23479360
NM_021944	C14orf93	uncharacterized prote	-	23456109	23479360
NM_001256849	POLD1	DNA polymerase del	+	50887579	50921275
NM_002691	POLD1	DNA polymerase del	+	50887579	50921275
NM_001035516	DMKN	dermokine isoform 1	-	35988118	35992813
NM_001126056	DMKN	dermokine isoform 3	-	35988118	36004560
NM_001126057	DMKN	dermokine isoform 5	-	35994205	36004560
NM_001126058	DMKN	dermokine isoform 4	-	35994205	36004560
NM_001126059	DMKN	dermokine isoform 6	-	35988118	36001391
NM_001190347	DMKN	dermokine isoform 8	-	35988118	36004560
NM_001190348	DMKN	dermokine isoform 9	-	35994205	36004560
NM_001190349	DMKN	dermokine isoform 1	-	35994205	36004560
NM_033317	DMKN	dermokine isoform 2	-	35988118	36004560
NM_005251	FOXC2	forkhead box protein	+	86600856	86602537
NM_001444	FABP5	fatty acid-binding pr	+	82192717	82197012
NM_014909	VASH1	vasohibin-1	+	77228234	77249363
NM_020799	STAMBPL1	AMSH-like protease	+	90639943	90683259
ENST00000415882	C1orf132	chromosome 1 open	-	207990581	208042495
NM_001145348	KDM8	lysine-specific demet	+	27215295	27233089
NM_024773	KDM8	lysine-specific demet	+	27214806	27233089
AGGAAATGACAGACTCAATGCC					
NM_006275	SRSF6	serine/arginine-rich s	+	42086503	42092244
NM_001489	NR6A1	nuclear receptor subf	-	127284702	127533576
NM_033334	NR6A1	nuclear receptor subf	-	127284702	127533576
NM_002687	PNN	pinin	+	39644386	39652422
NM_014730	MLEC	malectin precursor	+	121124948	121139667

986.085578	245.662301	9.345168	8.13798	1042.53	1134.4802
2558.055917	625.85145	10.894719	9.668858	2730.5415	2982.3787
23852.69983	5038.554233	14.065494	12.707996	27743.605	38039.332
975.485882	222.636472	9.393129	8.01689	1424.8195	1123.4471
306.520983	116.422056	7.752838	6.687791	246.3304	112.5434
1815.555128	477.956663	10.326049	9.257129	2057.2632	1890.1562
28.069366	8.019058	4.133706	3.120239	39.4618	68.380035
28.069366	8.019058	4.133706	3.120239	39.4618	68.380035
28.069366	8.019058	4.133706	3.120239	39.4618	68.380035
28.069366	8.019058	4.133706	3.120239	39.4618	68.380035
215.220152	56.514217	7.097979	6.091408	117.879875	83.259735
101.580003	21.733392	6.158838	4.636431	127.561485	117.81549
53.020394	7.907083	5.234155	3.16394	71.39202	100.331184
345.164403	51.260843	7.961076	5.834137	266.11646	154.82495
102.881242	15.184208	6.318066	3.96884	85.444084	89.09856
40.19943	12.543235	4.965964	3.58896	43.00435	30.261692
40.19943	12.543235	4.965964	3.58896	43.00435	30.261692
131.617312	30.064929	6.260329	5.112059	131.0398	223.35098
131.617312	30.064929	6.260329	5.112059	131.0398	223.35098
131.617312	30.064929	6.260329	5.112059	131.0398	223.35098
235.332203	58.299868	7.44407	6.110295	270.28287	384.64868
235.332203	58.299868	7.44407	6.110295	270.28287	384.64868
49.735129	12.263361	5.268043	3.87449	55.296707	51.15861
658.708072	80.220791	7.838148	6.241248	94.33742	48.566
658.708072	80.220791	7.838148	6.241248	94.33742	48.566
658.708072	80.220791	7.838148	6.241248	94.33742	48.566
658.708072	80.220791	7.838148	6.241248	94.33742	48.566
658.708072	80.220791	7.838148	6.241248	94.33742	48.566
658.708072	80.220791	7.838148	6.241248	94.33742	48.566
658.708072	80.220791	7.838148	6.241248	94.33742	48.566
658.708072	80.220791	7.838148	6.241248	94.33742	48.566
658.708072	80.220791	7.838148	6.241248	94.33742	48.566
56.23083	11.966345	5.165914	3.63264	84.0701	65.45948
79.269913	10.877438	5.324532	3.504793	146.89969	129.50049
112.841412	35.560279	6.465492	5.389488	132.6253	111.5879
197.582672	20.772113	6.243329	4.29122	383.52927	576.2493
540.44021	62.563541	8.141171	6.177495	739.8548	800.8052
83.172722	13.023733	5.897817	3.891364	64.94563	59.72509
572.517225	89.159235	8.62505	6.658257	492.44672	465.2801
121.71997	29.430102	6.402319	4.915669	95.20561	132.64342
8855.634067	2355.413967	12.617501	11.611801	9936.424	11273.55
107.905316	35.829039	6.362311	5.289555	102.03078	81.335205
2037.376967	723.919182	10.528661	9.481167	2726.6743	2363.6418
18.178234	7.040994	3.854086	2.828444	25.069014	13.154799
349.162835	69.019241	8.04156	6.273806	292.99838	237.08041
349.162835	69.019241	8.04156	6.273806	292.99838	237.08041
37.137832	8.761411	4.829609	3.1456	54.686607	44.13648
45.228217	11.849331	5.149319	3.818929	48.978367	36.662575
472.348412	135.605502	8.451084	7.343537	486.49686	464.8079
118.39563	29.208653	6.399369	5.144995	151.46591	150.76512
32.594548	8.388413	4.670682	3.273673	29.79771	21.464613
82.521485	16.45759	5.80618	4.307615	73.668365	42.568703
81.761967	22.525429	5.776509	4.768414	64.784515	21.905218
122.173125	20.344358	6.364508	4.580576	132.80942	242.53362
122.173125	20.344358	6.364508	4.580576	132.80942	242.53362
47799.00033	9926.752633	14.945834	12.708708	52791.91	68116.625
122.061607	30.877328	6.445437	5.206221	163.51358	149.65501
52.080488	11.495805	5.319484	3.389083	45.906273	59.273453
1751.140433	299.664802	10.301133	8.585982	2328.5806	2147.7583
49.820987	8.406751	5.279074	3.153235	64.02232	59.129242
230.585415	62.494301	7.431128	6.126102	272.02258	325.47226
2974.829398	418.11623	10.470387	8.88343	2312.1667	2078.7761

498.95593	323.10864	1727.0693	1190.3694	299.50467	160.82173
2370.4734	2550.6335	1970.0472	2744.2612	635.36145	562.5668
12578.276	26271.248	15865.64	22618.098	4895.868	5927.562
1538.2521	423.57	664.26324	678.56335	169.73747	150.9996
334.97726	229.25272	449.42285	466.59927	35.635693	23.847216
2287.75	2265.7605	700.66797	1691.7329	366.4485	344.47787
14.512231	9.540442	17.11775	19.403936	9.76212	10.31269
14.512231	9.540442	17.11775	19.403936	9.76212	10.31269
14.512231	9.540442	17.11775	19.403936	9.76212	10.31269
14.512231	9.540442	17.11775	19.403936	9.76212	10.31269
125.07681	312.4039	143.51282	509.18777	40.392063	31.27434
143.05528	31.744501	108.07917	81.22409	18.249258	26.057463
28.13813	32.597588	38.098335	47.565105	10.464631	5.2547693
370.05997	511.85583	289.13644	478.99277	61.17973	33.22788
85.05429	104.1619	126.1869	127.34172	15.04287	12.825401
49.571213	26.608234	44.32206	47.42903	11.359479	4.9999995
49.571213	26.608234	44.32206	47.42903	11.359479	4.9999995
25.767282	36.020428	204.27048	169.2549	33.766792	39.441757
25.767282	36.020428	204.27048	169.2549	33.766792	39.441757
25.767282	36.020428	204.27048	169.2549	33.766792	39.441757
146.50844	176.38208	207.89055	226.2806	46.870697	29.041555
146.50844	176.38208	207.89055	226.2806	46.870697	29.041555
50.976402	60.583485	25.405659	54.98991	11.90801	14.519171
112.629814	2014.3718	483.4104	1198.933	35.484898	14.69463
112.629814	2014.3718	483.4104	1198.933	35.484898	14.69463
112.629814	2014.3718	483.4104	1198.933	35.484898	14.69463
112.629814	2014.3718	483.4104	1198.933	35.484898	14.69463
112.629814	2014.3718	483.4104	1198.933	35.484898	14.69463
112.629814	2014.3718	483.4104	1198.933	35.484898	14.69463
112.629814	2014.3718	483.4104	1198.933	35.484898	14.69463
112.629814	2014.3718	483.4104	1198.933	35.484898	14.69463
112.629814	2014.3718	483.4104	1198.933	35.484898	14.69463
79.61333	9.398762	54.523506	44.319805	8.242886	8.495557
130.48831	4.9999995	32.76704	30.963947	13.945042	5.611807
94.4154	112.11148	104.13567	122.17272	25.557316	15.711138
134.65024	33.849995	25.376907	31.840322	28.483822	54.604263
655.9612	27.991459	566.3016	451.727	66.42396	35.352684
66.43496	167.60161	39.46733	100.86171	10.332039	6.522034
533.16187	1053.1881	208.94331	682.08325	81.252525	129.17073
34.89431	192.2102	106.24571	169.12057	17.48662	7.5453544
7764.5654	11006.138	4296.286	8856.841	2200.5496	2182.6794
129.5288	69.53427	137.15721	127.845634	34.59984	12.53208
1699.624	1166.7565	2141.7654	2125.7998	838.9857	269.73187
16.093105	24.073898	14.825129	15.853459	7.856269	4.9999995
317.58542	380.7785	368.02542	498.50888	42.053974	25.012312
317.58542	380.7785	368.02542	498.50888	42.053974	25.012312
22.68794	45.019047	27.35802	28.938896	8.667067	4.9999995
52.65046	58.76161	29.44338	44.872913	9.07682	8.701623
494.8697	677.6556	255.53775	454.72266	157.56067	86.97143
133.47832	39.00241	126.3522	109.30982	29.610641	23.384468
48.325897	28.38169	33.912876	33.684505	7.4549127	6.356764
44.83047	42.56264	156.68013	134.8186	17.988304	14.932191
85.28845	47.49074	145.95387	125.14901	20.681026	13.656351
34.10172	96.42672	102.41439	124.75288	21.86063	15.976801
34.10172	96.42672	102.41439	124.75288	21.86063	15.976801
26061.094	15175.965	65176.508	59471.9	12677.972	2001.5061
185.78122	50.81661	87.08084	95.522385	24.932943	20.625626
28.268513	65.17436	51.116272	62.744057	5.4826345	4.9999995
2261.3508	1453.0748	915.2701	1400.808	296.75708	218.31334
50.597664	39.32463	37.650913	48.201153	8.066454	4.9999995
179.55289	136.40372	247.43558	222.62546	81.66079	44.05952
2164.388	145.39949	6168.4834	4979.7627	367.25748	292.2382

422.1397	122.961136	334.55637	133.9902	9.551316	9.796671
745.14435	666.25494	633.7981	511.98306	10.926253	11.119517
4050.3113	6304.981	4375.4937	4677.1094	14.313887	14.860018
179.8152	127.3927	466.74054	241.13332	10.004446	9.782856
57.884815	106.56191	306.4071	168.1956	7.4896693	6.595354
458.30408	627.36176	591.28284	479.86493	10.528252	10.471233
12.26024	5.7793	4.9999995	4.9999995	4.8407536	5.921891
12.26024	5.7793	4.9999995	4.9999995	4.8407536	5.921891
12.26024	5.7793	4.9999995	4.9999995	4.8407536	5.921891
12.26024	5.7793	4.9999995	4.9999995	4.8407536	5.921891
55.121746	77.69056	79.6368	54.969795	6.430396	6.183736
21.791182	8.383744	31.333408	24.585295	6.5457187	6.6573205
6.019308	9.332702	11.371091	4.9999995	5.697596	6.4337006
104.472595	30.968912	48.95867	28.757269	7.5985193	7.0377994
30.629889	17.830671	4.9999995	9.77642	5.9633613	6.272367
20.527338	5.606031	23.09368	9.67288	4.9670706	4.853403
20.527338	5.606031	23.09368	9.67288	4.9670706	4.853403
26.617893	17.328949	46.313793	16.92039	6.586653	7.546888
26.617893	17.328949	46.313793	16.92039	6.586653	7.546888
26.617893	17.328949	46.313793	16.92039	6.586653	7.546888
68.046295	55.150906	96.05718	54.632572	7.620288	8.294148
68.046295	55.150906	96.05718	54.632572	7.620288	8.294148
16.446484	8.573568	12.314439	9.818494	5.323045	5.5427933
44.07205	87.5648	175.2169	124.291466	6.1135817	5.474477
44.07205	87.5648	175.2169	124.291466	6.1135817	5.474477
44.07205	87.5648	175.2169	124.291466	6.1135817	5.474477
44.07205	87.5648	175.2169	124.291466	6.1135817	5.474477
44.07205	87.5648	175.2169	124.291466	6.1135817	5.474477
44.07205	87.5648	175.2169	124.291466	6.1135817	5.474477
44.07205	87.5648	175.2169	124.291466	6.1135817	5.474477
44.07205	87.5648	175.2169	124.291466	6.1135817	5.474477
44.07205	87.5648	175.2169	124.291466	6.1135817	5.474477
6.8917613	4.9999995	25.895939	17.271929	5.9416113	5.859637
14.94285	5.172073	20.368504	5.224351	6.7440724	6.7857003
38.077488	49.73304	45.289864	38.992825	6.6038446	6.581672
6.47347	13.398559	13.723762	7.948802	8.123682	8.859691
104.40038	39.058723	83.964935	46.180565	9.064061	9.314474
22.290077	11.91547	14.21414	12.86864	5.562163	5.7370043
23.320051	127.864395	82.60539	90.74232	8.462374	8.552278
38.448246	24.625364	60.10765	28.367378	6.1259975	6.8184953
2072.362	3108.634	2330.3545	2237.9043	12.792551	13.0087
57.541977	22.145449	55.8486	32.306286	6.225027	6.151743
2088.5542	368.10547	438.05615	340.0817	10.924857	10.77782
11.845951	7.543743	4.9999995	4.9999995	4.1882877	3.7578416
51.201355	122.950325	92.32084	80.57664	7.7407985	7.626748
51.201355	122.950325	92.32084	80.57664	7.7407985	7.626748
19.288187	6.7831235	5.8404236	6.9896655	5.3061304	5.3481426
12.965761	17.808426	9.724709	12.81865	5.1535683	5.103252
236.82533	102.226524	130.93199	99.117065	8.447027	8.55081
36.581486	19.874178	37.014523	28.786625	6.7864685	6.9998484
10.0477495	14.795968	5.957477	5.7176075	4.44253	4.3878975
17.202297	20.520988	18.183619	9.918143	5.747101	5.300892
23.7032	24.306946	29.68697	23.118078	5.558642	4.4180727
30.649494	12.429778	24.217726	16.931719	6.6056657	7.656527
30.649494	12.429778	24.217726	16.931719	6.6056657	7.656527
35907.297	1575.3015	5284.4707	2113.9685	15.287802	15.672249
33.057964	23.082308	49.60963	33.955498	6.895493	6.9915404
4.9999995	14.33546	27.080616	12.076121	5.0606174	5.727311
383.5252	370.22733	261.8406	267.32526	10.710994	10.6541
14.40903	6.9154725	9.836611	6.2129416	5.5429316	5.724901
120.264984	33.68064	60.16227	35.137604	7.6293216	8.065734
431.13135	148.67268	874.67834	394.71933	10.702623	10.606144

8.61194	8.014741	10.459863	9.6364765	8.493663	8.177273
10.8700485	10.980642	10.647209	10.824647	9.601699	9.993444
13.311917	14.370472	13.676494	13.860174	12.583225	13.4268055
10.219233	8.418165	9.10502	8.829055	7.6525607	8.091201
8.053862	7.5356736	8.55184	8.29063	5.3905077	5.4100704
10.818191	10.820469	9.185588	10.132558	8.779891	9.289892
3.5339692	2.6422672	4.0166245	3.8467276	3.510912	4.151476
3.5339692	2.6422672	4.0166245	3.8467276	3.510912	4.151476
3.5339692	2.6422672	4.0166245	3.8467276	3.510912	4.151476
3.5339692	2.6422672	4.0166245	3.8467276	3.510912	4.151476
6.6452403	7.965822	6.944784	8.417893	5.5721846	5.8055353
6.838156	4.5430684	6.5406857	5.8280783	4.424577	5.5375266
4.5048423	4.5843635	5.0983343	5.086096	3.6147182	3.1238182
8.184247	8.681705	7.9328475	8.331337	6.169254	5.8916407
6.0943522	6.3524423	6.7597075	6.466164	4.143899	4.4783015
5.315003	4.2693567	5.309368	5.081581	3.7364175	2.4279919
5.315003	4.2693567	5.309368	5.081581	3.7364175	2.4279919
4.376024	4.737487	7.4365597	6.878362	5.306449	6.144624
4.376024	4.737487	7.4365597	6.878362	5.306449	6.144624
4.376024	4.737487	7.4365597	6.878362	5.306449	6.144624
6.8698378	7.140625	7.4592423	7.2802796	5.780302	5.699424
6.8698378	7.140625	7.4592423	7.2802796	5.780302	5.699424
5.355456	5.5437784	4.553592	5.2895956	3.8028843	4.670077
6.495697	10.642509	8.656863	9.645759	5.3832984	4.687362
6.495697	10.642509	8.656863	9.645759	5.3832984	4.687362
6.495697	10.642509	8.656863	9.645759	5.3832984	4.687362
6.495697	10.642509	8.656863	9.645759	5.3832984	4.687362
6.495697	10.642509	8.656863	9.645759	5.3832984	4.687362
6.495697	10.642509	8.656863	9.645759	5.3832984	4.687362
6.495697	10.642509	8.656863	9.645759	5.3832984	4.687362
6.495697	10.642509	8.656863	9.645759	5.3832984	4.687362
6.495697	10.642509	8.656863	9.645759	5.3832984	4.687362
5.99955	2.6198082	5.5865307	4.9883494	3.2479994	3.8580766
6.705492	2.3221264	4.8911276	4.4986725	4.0276976	3.224631
6.238665	6.466601	6.4881744	6.4139967	4.9204407	4.7917657
6.747841	4.640364	4.551584	4.536811	5.0666924	6.621921
8.992563	4.3415647	8.885673	8.248692	6.288945	5.9847794
5.746375	7.061634	5.1430926	6.136636	3.5961955	3.4625905
8.705203	9.725263	7.4661613	8.839019	6.568569	7.862587
4.8101873	7.266332	6.5156918	6.8772097	4.3659554	3.6758711
12.546705	13.0815	11.769413	12.506136	11.426614	11.982133
6.6954885	5.75281	6.876506	6.472292	5.3447123	4.44536
10.362165	9.87117	10.776549	10.459407	10.023644	8.942398
3.690878	4.1038914	3.8186889	3.5649295	3.169776	2.4279919
7.9755254	8.250004	8.270824	8.38546	5.626088	5.4751754
7.9755254	8.250004	8.270824	8.38546	5.626088	5.4751754
4.1926	5.084915	4.646643	4.3992205	3.3250613	2.4279919
5.399629	5.4965315	4.7389097	5.004026	3.3960164	3.8959959
8.600178	9.09678	7.755804	8.255904	7.542578	7.2939515
6.7345743	4.863475	6.7611275	6.2507205	5.1188383	5.376331
5.2783346	4.3641796	4.9349747	4.6161747	3.0909386	3.4198887
5.1676702	4.9990125	7.072974	6.549432	4.4064465	4.711655
6.0981045	5.1697507	6.9697366	6.4447474	4.6084595	4.5739746
4.777419	6.2418895	6.464509	6.441036	4.686498	4.817898
4.777419	6.2418895	6.464509	6.441036	4.686498	4.817898
14.397183	13.531816	15.557471	15.228483	13.882639	11.8518305
7.2163563	5.274757	6.2366867	6.0577903	4.8829436	5.1986766
4.5116596	5.6535916	5.4985404	5.465186	2.657188	2.4279919
10.801527	10.18626	9.589073	9.864846	8.478647	8.625574
5.345134	4.8751407	5.083185	5.1031523	3.2137773	2.4279919
7.1586914	6.7629538	7.7086353	7.261433	6.5755105	6.30748
10.729852	6.856805	12.271666	11.655234	8.78267	9.0532875

8.675404	7.3042655	8.619994	7.5572824
9.483771	9.846637	9.536282	9.551316
11.961581	13.098996	12.388632	12.7887335
7.442043	7.360831	9.105436	8.4492655
5.8434753	7.0837464	8.502987	7.895961
8.801858	9.764435	9.443367	9.463334
3.7017384	2.6427412	2.3618152	2.352752
3.7017384	2.6427412	2.3618152	2.352752
3.7017384	2.6427412	2.3618152	2.352752
3.7017384	2.6427412	2.3618152	2.352752
5.776781	6.6042094	6.5738	6.21594
4.479794	3.1575496	5.244028	4.9751134
2.7912567	3.3200235	3.781071	2.352752
6.670253	5.1633906	5.8860703	5.224211
4.9512362	4.3008986	2.3618152	3.5768867
4.4002595	2.6052425	4.8035316	3.5603154
4.4002595	2.6052425	4.8035316	3.5603154
4.7561965	4.2585983	5.8021927	4.4042945
4.7561965	4.2585983	5.8021927	4.4042945
4.7561965	4.2585983	5.8021927	4.4042945
6.0669155	6.0669155	6.8421793	6.206035
6.0669155	6.0669155	6.8421793	6.206035
4.0976577	3.1957858	3.897755	3.5827823
5.4592195	6.7837963	7.6949286	7.438882
5.4592195	6.7837963	7.6949286	7.438882
5.4592195	6.7837963	7.6949286	7.438882
5.4592195	6.7837963	7.6949286	7.438882
5.4592195	6.7837963	7.6949286	7.438882
5.4592195	6.7837963	7.6949286	7.438882
5.4592195	6.7837963	7.6949286	7.438882
5.4592195	6.7837963	7.6949286	7.438882
5.4592195	6.7837963	7.6949286	7.438882
2.952317	2.3408759	4.963566	4.4330034
3.9665592	2.5355656	4.6136804	2.6606245
5.2522335	5.906407	5.769356	5.696725
2.8779914	3.8663142	4.0500336	3.2643685
6.669389	5.5233006	6.646015	5.9525433
4.5112634	3.6858318	4.1006956	3.991607
4.574152	7.3672194	6.622568	6.9544463
5.267334	4.8084636	6.1739955	5.202394
10.962376	12.119435	11.437958	11.74229
5.833062	4.6384826	6.068378	5.4073334
10.978442	8.980464	9.013856	8.948195
3.6547575	3.003573	2.3618152	2.352752
5.671432	7.3039308	6.7799	6.7863116
5.671432	7.3039308	6.7799	6.7863116
4.316754	2.8630996	2.8752835	3.0654106
3.7741036	4.2990413	3.5633066	3.9851072
7.835102	7.019902	7.276976	7.0927143
5.195106	4.4706144	5.4826636	5.226414
3.434793	4.023637	2.8970428	2.7757387
4.1564403	4.521924	4.453799	3.595424
4.595487	4.786169	5.1691947	4.8771996
4.9523196	3.752511	4.8692756	4.404952
4.9523196	3.752511	4.8692756	4.404952
15.066691	11.140424	12.653332	11.657332
5.058316	4.7076597	5.904049	5.4856815
2.350709	3.9721143	5.0308814	3.8956127
8.535077	8.99023	8.287911	8.598453
3.9162943	2.888086	3.5786424	2.8946195
6.869166	5.2931037	6.1753726	5.5359774
8.703615	7.588686	10.011737	9.160585

ASHGA5P054920	4.33314E-05	0.00669663	3.9365715	up	noncoding
ASHGA5P054920	4.33314E-05	0.00669663	3.9365715	up	noncoding
ASHGA5P054920	4.33314E-05	0.00669663	3.9365715	up	noncoding
ASHGA5P044990	0.006976892	0.052947146	2.0755174	up	noncoding
ASHGA5P043028	0.025911715	0.100359338	3.6736276	up	noncoding
ASHGA5P027749	0.012072842	0.067365838	2.8079336	up	noncoding
ASHGA5P047729	0.003734465	0.03919943	2.4678895	up	noncoding
ASHGA5P047028	0.000893083	0.020125432	4.0765863	up	noncoding
ASHGA5P027386	2.62101E-05	0.005249161	4.3066997	up	noncoding
ASHGA5P027647	0.002103004	0.03028922	2.0381234	up	noncoding
ASHGA5P027647	0.002103004	0.03028922	2.0381234	up	noncoding
ASHGA5P048737	0.030251264	0.109041885	2.3024075	up	noncoding
ASHGA5P045021	0.040580077	0.128872335	2.0796945	up	noncoding
ASHGA5P049569	0.012424882	0.068295423	2.141212	up	noncoding
ASHGA5P015118	0.007503929	0.054436508	2.0250994	up	noncoding
ASHGA5P048208	0.018787698	0.083353796	2.7656626	up	noncoding
ASHGA5P025833	0.004330748	0.041714476	2.0882304	up	noncoding
ASHGA5P025833	0.004330748	0.041714476	2.0882304	up	noncoding
ASHGA5P025833	0.004330748	0.041714476	2.0882304	up	noncoding
ASHGA5P025833	0.004330748	0.041714476	2.0882304	up	noncoding
ASHGA5P048465	0.001576938	0.026655485	2.1805408	up	noncoding
ASHGA5P056376	0.043868804	0.134396669	3.128841	up	noncoding
ASHGA5P050634	0.000733646	0.018192952	2.182906	up	noncoding
ASHGA5P022188	0.024606604	0.097329913	2.5821043	up	noncoding
ASHGA5P047584	0.030024918	0.108497141	2.3140133	up	noncoding
ASHGA5P039023	0.016408985	0.07821393	2.4179316	up	noncoding
ASHGA5P056135	0.002918326	0.034973658	2.4660172	up	noncoding
ASHGA5P046499	0.004914871	0.044432255	4.4191507	up	noncoding
ASHGA5P037342	0.000258809	0.012173186	3.5033705	up	noncoding
ASHGA5P051695	0.000158556	0.011066341	2.1379806	up	noncoding
ASHGA5P035648	0.001968108	0.029367232	2.3655591	up	noncoding
ASHGA5P035648	0.001968108	0.029367232	2.3655591	up	noncoding
ASHGA5P035230	0.003642234	0.038724469	2.8755849	up	noncoding
ASHGA5P018933	0.000197821	0.01146874	3.2231065	up	noncoding
ASHGA5P018933	0.000197821	0.01146874	3.2231065	up	noncoding
ASHGA5P018933	0.000197821	0.01146874	3.2231065	up	noncoding
ASHGA5P018933	0.000197821	0.01146874	3.2231065	up	noncoding
ASHGA5P018933	0.000197821	0.01146874	3.2231065	up	noncoding
ASHGA5P016690	0.010622863	0.063796341	2.0626029	up	noncoding
ASHGA5P015537	0.005811063	0.048450721	2.8671803	up	noncoding
ASHGA5P043828	2.48233E-05	0.005188131	19.8894671	up	noncoding
ASHGA5P056495	0.003800056	0.039493479	2.342502	up	noncoding
ASHGA5P032534	8.69644E-05	0.008766146	3.9934355	up	noncoding
ASHGA5P043271	0.008252248	0.056900112	2.7106875	up	noncoding
ASHGA5P021475	0.012356809	0.068094238	3.7987237	up	noncoding
ASHGA5P057140	0.015310837	0.075783608	2.0865125	up	noncoding
ASHGA5P040524	0.016417117	0.07821393	2.2414036	up	noncoding
ASHGA5P035743	0.005651733	0.047766529	2.0981149	up	noncoding
ASHGA5P053899	0.01152501	0.066150897	2.3034589	up	noncoding
ASHGA5P028787	0.004748126	0.043793775	3.639398	up	noncoding
ASHGA5P042798	0.001238993	0.023574374	2.8055259	up	noncoding
ASHGA5P042798	0.001238993	0.023574374	2.8055259	up	noncoding
ASHGA5P015520	0.010427798	0.063044889	2.2519529	up	noncoding
ASHGA5P015520	0.010427798	0.063044889	2.2519529	up	noncoding
ASHGA5P015520	0.010427798	0.063044889	2.2519529	up	noncoding
ASHGA5P045496	0.002326801	0.031698104	2.0718169	up	noncoding
ASHGA5P043843	0.000292412	0.012559206	7.1084816	up	noncoding
ASHGA5P026546	0.000486867	0.015406196	10.0792354	up	noncoding
ASHGA5P032627	0.007464447	0.054241022	2.7083683	up	noncoding
ASHGA5P033262	0.007419999	0.054076953	2.6334871	up	noncoding
ASHGA5P033262	0.007419999	0.054076953	2.6334871	up	noncoding
ASHGA5P050119	0.014485908	0.073639339	2.173554	up	noncoding

ENST00000544983	SNHG1	GENCODE	870	chr11	-
ENST00000544983	SNHG1	GENCODE	870	chr11	-
ENST00000544983	SNHG1	GENCODE	870	chr11	-
ENST00000518865	HAS2-AS1	GENCODE	577	chr8	+
ENST00000456114	AC005537.2	GENCODE	636	chr7	-
ENST00000513358	RP11-253E3.3	GENCODE	3485	chr12	+
NR_026941	CASC2	RefSeq	2407	chr10	+
uc010qoz.1	NR_026754	UCSC_knowngene	686	chr10	-
uc001srm.3	AK024134	UCSC_knowngene	2486	chr12	-
ENST00000536910	RP11-87C12.4	GENCODE	434	chr12	-
ENST00000536910	RP11-87C12.4	GENCODE	434	chr12	-
uc001bqn.3	SNHG12	UCSC_knowngene	1641	chr1	-
ENST00000519880	RP11-89K10.1	GENCODE	574	chr8	+
ENST00000443508	RP11-344E13.3	GENCODE	1216	chr17	+
ENST00000416860	RP3-395M20.8	GENCODE	3522	chr1	-
ENST00000534819	AP003068.18	GENCODE	2354	chr11	-
NR_038396	LOC100506469	RefSeq	1651	chr19	-
NR_038396	LOC100506469	RefSeq	1651	chr19	-
NR_038396	LOC100506469	RefSeq	1651	chr19	-
NR_038396	LOC100506469	RefSeq	1651	chr19	-
ENST00000366405	AP006216.11	GENCODE	598	chr11	-
TCONS_00003814	XLOC_001616	LincRNAs identified	448	chr2	+
ENST00000420330	AC009404.2	GENCODE	3090	chr2	+
ENST00000551847	RP11-722P11.4	GENCODE	588	chr12	-
ENST00000439157	RP11-94M14.2	GENCODE	784	chr10	+
ENST00000464612	SNHG12	GENCODE	719	chr1	-
NR_001459	SNHG10	RefSeq	1341	chr14	-
ENST00000452501	RP5-1172N10.2	GENCODE	835	chrX	+
uc002zcj.2	AX747730	UCSC_knowngene	2538	chr21	-
NR_033289	GK5	RefSeq	10938	chr3	-
ENST00000567819	RP11-27M15.1	GENCODE	4000	chr2	-
ENST00000567819	RP11-27M15.1	GENCODE	4000	chr2	-
NR_046112	LOC440894	RefSeq	1092	chr2	-
NR_027676	BRCA1	RefSeq	7128	chr17	-
NR_027676	BRCA1	RefSeq	7128	chr17	-
NR_027676	BRCA1	RefSeq	7128	chr17	-
NR_027676	BRCA1	RefSeq	7128	chr17	-
NR_027676	BRCA1	RefSeq	7128	chr17	-
ENST00000433005	AC003075.4	GENCODE	540	chr7	-
ENST00000421140	NSUN5P1	pseudogene	1535	chr7	+
ENST00000419422	RP11-132A1.4	GENCODE	705	chr7	+
TCONS_00004869	XLOC_001616	LincRNAs identified	228	chr2	+
TCONS_00029032	XLOC_013952	LincRNAs identified	443	chr21	+
AF143879		LincRNAs identified	581	chr16	+
ENST00000533039	AC005152.2	GENCODE	435	chr17	-
TCONS_00013654	XLOC_006311	LincRNAs identified	328	chr7	+
ENST00000503007	CTD-2201I18.1	GENCODE	956	chr5	-
ENST00000452342	RP11-644K8.1	GENCODE	573	chr2	+
NR_027231	PPP2R3B-AS1	RefSeq	1532	chrX	+
uc001uyq.1	AK054970	UCSC_knowngene	3681	chr13	+
ENST00000431459	RP3-436N22.3	GENCODE	677	chr1	+
ENST00000431459	RP3-436N22.3	GENCODE	677	chr1	+
ENST00000453011	RP11-421L21.3	GENCODE	794	chr1	+
ENST00000453011	RP11-421L21.3	GENCODE	794	chr1	+
ENST00000453011	RP11-421L21.3	GENCODE	794	chr1	+
ENST00000428643	HMGA1P4	GENCODE	334	chr9	-
ENST00000470135	RP5-884M6.1	GENCODE	673	chr7	+
ENST00000423403	RP11-436K8.1	GENCODE	2344	chr1	-
ENST00000533996	AC005152.2	GENCODE	1001	chr17	-
NR_038415	BZRAP1-AS1	RefSeq	2404	chr17	+
NR_038415	BZRAP1-AS1	RefSeq	2404	chr17	+
TCONS_00013645	XLOC_006301	LincRNAs identified	2020	chr7	+

62622153	62623230	ENST00000540904,	CCAGGGTGAATA	23642	bidirectional
62622153	62623230	ENST00000540904,	CCAGGGTGAATA	23642	bidirectional
62622153	62623230	ENST00000540904,	CCAGGGTGAATA	23642	bidirectional
122651532	122655691		CTCGCCTCTTGTA	594842	natural antisense
43011865	43084204		GACTAGCATTAGCTGTCCTGATTAA		intergenic
3150602	3154116		CAGAATGAAAACGAGATACTTTTCC		intergenic
119806331	119859647	uc009xzb.3	GTTTGGCAGTGT	255082	bidirectional
99477216	99477902		ATCCACTGTTTA	100270710	natural antisense
63326429	63328915		GCCTGCCAAACATACCAAACACTAT		exon sense-overlappi
122337853	122338900		AAATGAGCCCCAAGAAGAGATCCT		intronic antisense
122337853	122338900		AAATGAGCCCCAAGAAGAGATCCT		intronic antisense
28905049	28908366	uc001bql.3, uc001bq	GTGCTTTAAGGT	85028	natural antisense
127570119	127725660		GCTGCAAAACCAGGTGACATGAAA		intronic antisense
20824357	20833359		TCTAGTCTACATACAATGAAAAGGA		intergenic
2481358	2488450		AGGAGGGAAACTGAGGCATGGCAA		natural antisense
64941587	64943941		TTTACAATTAACATCTTAAAGGAGT		intergenic
36031639	36036931		CATTGGAAAGGT	100506469	natural antisense
36031639	36036931		CATTGGAAAGGT	100506469	natural antisense
36031639	36036931		CATTGGAAAGGT	100506469	natural antisense
36031639	36036931		CATTGGAAAGGT	100506469	natural antisense
116645825	116646592		GAAAAAGCTGCTTATTTTGTGCGTT		intergenic
107384141	107394766	TCONS_00003813	CTCTACCATGTCTTTTACACACACT		intergenic
118591512	118599234		GGAATTTAACCTCTCAAAAATGCTA		intergenic
48877069	48894744		TGTCTCGGAGAAATAAGGAACAAA		natural antisense
92214454	92290873		AATGAAAAAGTATCACAGCTGAGA		intergenic
28905049	28907522	uc001bqm.3, uc001b	GGACCAAATGAT	85028	natural antisense
95999248	96001209	NR_003138	ATCAGGAAAGGT	283596	natural antisense
41134991	41136031		TTTTGCACACTAATTAATAAACTAC		intergenic
44263542	44266080		CTCTGTTCCTGTTGAATCATTCTTCG		intron sense-overlapp
141876368	141944449		CCATTGAGCCAG	256356	exon sense-overlappi
241522116	241526116		AACCATTTTAGGCTTTGCTGCAACC		bidirectional
241522116	241526116		AACCATTTTAGGCTTTGCTGCAACC		bidirectional
111133764	111142102	NR_046110, NR_04	CGTAGACCGCGC	440894	intergenic
41196311	41277340		TGTCCTTTATGA	672	exon sense-overlappi
41196311	41277340		TGTCCTTTATGA	672	exon sense-overlappi
41196311	41277340		TGTCCTTTATGA	672	exon sense-overlappi
41196311	41277340		TGTCCTTTATGA	672	exon sense-overlappi
41196311	41277340		TGTCCTTTATGA	672	exon sense-overlappi
17325898	17338981		GCCAGTACAGTGTTGTGAACCACTT		natural antisense
75039650	75045876	ENST00000457352,	TGGATCCTTCCTGCAGTGGCTCGGGTGAGATGGTATGC		
100951626	100954266		ACTCAATTCAAATATTTAATGGGTT		intergenic
107318305	107384278	TCONS_00003813	AAGTTGGAGTGGGCCAGGATTCCCC		intergenic
43474002	43475216		TGGAGGAACTTCTTTACTCCCTTGT		intergenic
25271823	25272384		TTTTCCTTAAAATCCCTGGATCTAG		intergenic
70078052	70085261		GGACACAGGATAGCCAAAAACAAT		intergenic
156774469	156774876	TCONS_00013655	GCACACGTAAGCTGTGTGTGTATCA		intergenic
79348196	79378227		GACAGACTAGCTAGAAGGGCTTTCT		intronic antisense
20251894	20254257		AATGTCTTCAATGAAAAAACAGCCA		bidirectional
281384	282054		TTCTAAGGGAAT	283981	intergenic
42813345	42817026		CATCTTTCCCAAAGGGCACTGCCTT		intergenic
173524389	173581911		AATCCAGAAGAAGCCAAGTGATCTA		intronic antisense
173524389	173581911		AATCCAGAAGAAGCCAAGTGATCTA		natural antisense
101491433	101546118	ENST00000421013	CCTAAGCCTGGTTCCTGAATATATC		bidirectional
101491433	101546118	ENST00000421013	CCTAAGCCTGGTTCCTGAATATATC		bidirectional
101491433	101546118	ENST00000421013	CCTAAGCCTGGTTCCTGAATATATC		bidirectional
131425412	131425857	NM_002131, NM_1	TTTGTCCCAGCC	100506080	intergenic
106415521	106421388		ATCCTAGGCTCTCCAAATGGGAGT		intergenic
61125302	61291256		GCAGCAAGTTTTTGCCACACTGTAA		intergenic
70072626	70081642		CTCACTTATTGGTGTGTCCTTACTG		intergenic
56414562	56431088	ENST00000582348,	AATTCTCCCCTTA	100506779	natural antisense
56414562	56431088	ENST00000582348,	AATTCTCCCCTTA	100506779	natural antisense
154795168	154797394	NR_028090	TTAATACTACACAGCGGCGATTCTA		intergenic

NM_001012662	SLC3A2	4F2 cell-surface anti	+	62623483	62656355
NM_001012664	SLC3A2	4F2 cell-surface anti	+	62623483	62656355
NM_002394	SLC3A2	4F2 cell-surface anti	+	62623483	62656355
NM_005328	HAS2	hyaluronan synthase	-	122625270	122653630
NM_014904	RAB11FIP2	rab11 family-interact	-	119764426	119806114
NM_031484	MARVELD1	MARVEL domain-c	+	99473464	99477909
NM_020700	PPM1H	protein phosphatase	-	63037762	63328665
NM_001261400	PSMD9	26S proteasome non-	+	122326636	122355771
NM_002813	PSMD9	26S proteasome non-	+	122326636	122355771
NM_017846	TRNAU1AP	tRNA selenocysteine	+	28879528	28905057
NM_174911	FAM84B	protein FAM84B	-	127564682	127570711
NM_003820	TNFRSF14	tumor necrosis factor	+	2487804	2495267
NM_001242597	TMEM147	transmembrane prote	+	36036501	36038429
NM_001242598	TMEM147	transmembrane prote	+	36036501	36038429
NM_014364	GAPDHS	glyceraldehyde-3-ph	+	36024313	36036221
NM_032635	TMEM147	transmembrane prote	+	36036501	36038429
NM_152319	C12orf54	uncharacterized prot	+	48876285	48890297
NM_017846	TRNAU1AP	tRNA selenocysteine	+	28879528	28905057
ENST00000331334	GLRX5	glutaredoxin 5 [Sour	+	96000929	96011055
NM_033661	WDR4	tRNA (guanine-N(7)	-	44263189	44299693
NM_001039547	GK5	putative glycerol kin	-	141876368	141944449
NM_023083	CAPN10	calpain-10 isoform a	+	241526132	241538526
NM_023085	CAPN10	calpain-10 isoform c	+	241526132	241538526
NM_007294	BRCA1	breast cancer type 1	-	41196311	41277500
NM_007297	BRCA1	breast cancer type 1	-	41196311	41277468
NM_007298	BRCA1	breast cancer type 1	-	41196311	41276132
NM_007299	BRCA1	breast cancer type 1	-	41196311	41277468
NM_007300	BRCA1	breast cancer type 1	-	41196311	41277500
NM_001621	AHR	aryl hydrocarbon rec	+	17338275	17385775
CGAGCAGACAGCTGGAGGATCC					
NM_003248	THBS4	thrombospondin-4 pr	+	79331169	79379107
NM_014713	LAPTM4A	lysosomal-associated	-	20232410	20251789
NM_198493	ANKRD45	ankyrin repeat domai	-	173577474	173639001
NM_178527	SLC9C2	sodium/hydrogen exc	-	173469603	173572233
NM_001077394	DPH5	diphthine synthase is	-	101455179	101491362
NM_001077395	DPH5	diphthine synthase is	-	101455179	101491362
NM_015958	DPH5	diphthine synthase is	-	101455179	101491362
NM_003168	SUPT4H1	transcription elongati	-	56422538	56429563
NM_017763	RNF43	E3 ubiquitin-protein	-	56431037	56494931

180.83488	30.070663	7.10871	5.13177	182.84227	152.143
180.83488	30.070663	7.10871	5.13177	182.84227	152.143
180.83488	30.070663	7.10871	5.13177	182.84227	152.143
91.175996	26.328714	5.95214	4.898669	83.1811	135.25002
117.609744	11.465251	5.335665	3.45846	14.486328	4.9999995
1538.427683	333.677995	10.162105	8.672596	1336.8551	1103.5818
189.197688	45.819809	7.142056	5.838778	220.39314	128.05861
372.77688	60.05476	8.056258	6.028896	513.48016	288.01288
657.95404	95.745707	8.955364	6.848781	583.4789	528.96326
507.086297	162.625005	8.594234	7.566992	605.621	501.05075
507.086297	162.625005	8.594234	7.566992	605.621	501.05075
2530.22405	711.632577	10.841438	9.638295	2769.029	1977.3496
48.813225	15.811591	5.253369	4.196997	51.120865	45.313805
24.92473	8.436517	4.282833	3.184405	15.423272	21.952578
777.750358	241.378474	9.184056	8.166063	832.94855	1013.3278
29.540164	8.026326	4.498291	3.030666	35.4947	19.008764
75.975198	24.057233	5.894445	4.832164	73.84069	80.75858
75.975198	24.057233	5.894445	4.832164	73.84069	80.75858
75.975198	24.057233	5.894445	4.832164	73.84069	80.75858
75.975198	24.057233	5.894445	4.832164	73.84069	80.75858
17.8702	6.318612	3.827542	2.702856	14.168272	15.19049
85.050788	12.316379	5.529681	3.884053	83.78983	154.9158
196.87908	57.29755	7.203834	6.077584	159.077	190.41745
335.896222	112.802439	7.995954	6.627407	419.26553	403.36816
75.058325	29.243765	5.854903	4.644506	102.06548	74.50735
172.471417	44.74099	6.994506	5.720733	224.61816	259.58057
795.406537	203.514692	9.192968	7.890785	843.0954	1026.2498
74.666721	10.205061	5.720218	3.576449	85.6937	27.13005
679.577615	114.329295	8.929848	7.121104	558.5814	777.2592
640.594948	211.578949	8.722837	7.626588	933.80945	564.97644
85.188503	24.095109	6.022231	4.78005	70.18255	64.00482
85.188503	24.095109	6.022231	4.78005	70.18255	64.00482
71.471889	16.419471	5.727387	4.203532	98.386765	116.41841
418.285403	81.335883	8.320208	6.631756	426.12958	411.9262
418.285403	81.335883	8.320208	6.631756	426.12958	411.9262
418.285403	81.335883	8.320208	6.631756	426.12958	411.9262
418.285403	81.335883	8.320208	6.631756	426.12958	411.9262
418.285403	81.335883	8.320208	6.631756	426.12958	411.9262
1223.842268	334.41145	9.785354	8.740888	1389.0798	770.47095
429.854458	100.00922	8.337453	6.817821	467.67944	601.96857
1509.096197	43.281994	9.840425	5.526493	1962.9951	3584.4502
279.135482	72.610818	7.680527	6.452477	385.7617	360.79395
80.85338	14.897524	5.845598	3.847968	73.09489	21.519772
1516.939075	328.215323	9.983196	8.544537	921.80945	944.8064
100.129091	19.492326	6.240952	4.315437	92.21349	129.14684
321.343382	83.941865	7.734266	6.673173	165.28824	135.85593
95.331405	29.976119	6.207867	5.043465	84.87381	114.5318
24.404573	8.050207	4.271463	3.20237	29.570974	28.668177
631.988117	134.699277	8.521364	7.317562	263.1319	231.50162
3967.356933	589.089415	11.341603	9.477903	5004.433	1758.6951
44.455897	9.914282	5.049132	3.56086	77.218956	53.757744
44.455897	9.914282	5.049132	3.56086	77.218956	53.757744
77.733414	20.31152	5.80823	4.637053	97.53706	130.54538
77.733414	20.31152	5.80823	4.637053	97.53706	130.54538
77.733414	20.31152	5.80823	4.637053	97.53706	130.54538
69440.07717	19801.78183	15.66342	14.612523	67264.32	79390.15
148.318122	13.436497	6.741353	3.911812	116.191414	50.710587
192.503927	10.062512	6.717738	3.384424	59.68862	81.30137
45.547844	12.003396	5.153216	3.715792	47.56322	41.021297
46.976064	13.030311	5.161377	3.764403	66.94955	41.11641
46.976064	13.030311	5.161377	3.764403	66.94955	41.11641
409.899307	119.51398	8.256198	7.136142	422.81134	519.02527

217.1326	113.51922	204.64363	214.72856	25.991026	20.767662
217.1326	113.51922	204.64363	214.72856	25.991026	20.767662
217.1326	113.51922	204.64363	214.72856	25.991026	20.767662
33.238007	42.163578	136.16057	117.0627	19.83546	36.848076
23.989109	274.6824	122.40533	265.0953	6.6143966	4.9999995
1376.1947	1592.6344	1729.8309	2091.4692	315.04553	427.18103
280.1957	224.0028	106.49737	176.03851	49.95746	32.42284
621.37146	187.517	307.53616	318.74362	61.96737	40.931206
629.7271	589.24524	772.6881	843.62164	82.04427	75.50834
436.6052	571.3488	442.02316	485.86887	175.78577	76.53575
436.6052	571.3488	442.02316	485.86887	175.78577	76.53575
3350.1558	3033.2134	1512.9178	2538.6787	477.60068	252.68404
58.185776	59.215923	28.971676	50.071304	11.84207	19.213337
30.738647	37.418404	21.324707	22.69077	7.90533	9.453952
576.9785	569.4409	856.0207	817.7857	239.45712	118.887886
45.15507	19.637293	25.12087	32.824284	4.9999995	4.9999995
57.918995	79.91902	74.647255	88.76665	24.201523	20.634953
57.918995	79.91902	74.647255	88.76665	24.201523	20.634953
57.918995	79.91902	74.647255	88.76665	24.201523	20.634953
57.918995	79.91902	74.647255	88.76665	24.201523	20.634953
25.557781	18.45012	18.57412	15.28042	5.5480933	4.9999995
4.9999995	138.76532	39.188484	88.645294	9.041055	18.519518
113.46336	261.12607	173.3937	283.7969	44.92138	33.411736
300.92877	226.97661	335.39072	329.44754	152.3677	33.3652
69.51607	50.675888	85.86268	67.72248	34.25397	8.325015
168.33998	157.4906	85.49397	139.30522	38.447598	25.906195
671.62225	421.60147	870.4372	939.4331	160.0924	89.64734
149.19432	71.72653	47.21085	67.044876	9.372247	9.140191
317.5048	559.97296	872.39343	991.7539	108.09972	76.94586
1308.3344	287.22406	338.9389	410.28644	320.1174	89.292694
58.057606	119.302025	83.93463	115.64939	20.246746	9.506423
58.057606	119.302025	83.93463	115.64939	20.246746	9.506423
50.915565	49.22191	53.854916	60.03377	19.434578	9.145717
396.1455	436.21747	323.58273	515.71094	78.844574	57.823776
396.1455	436.21747	323.58273	515.71094	78.844574	57.823776
396.1455	436.21747	323.58273	515.71094	78.844574	57.823776
396.1455	436.21747	323.58273	515.71094	78.844574	57.823776
396.1455	436.21747	323.58273	515.71094	78.844574	57.823776
1992.0605	1274.479	740.65436	1176.309	308.96332	218.26926
343.48306	450.98138	308.4711	406.5432	120.323784	42.599117
417.46988	1052.4663	858.7519	1178.4438	65.63167	85.69997
229.91965	134.12589	291.9034	272.3083	66.0147	55.280388
117.097565	98.57164	70.0986	104.73781	8.202189	4.9999995
871.6435	3147.6846	1068.158	2147.5325	291.71207	82.8703
69.66507	132.8509	58.52127	118.376976	11.799821	5.55714
192.67014	590.21124	296.63684	547.3979	84.72529	43.72237
77.81656	111.93309	73.33651	109.49666	14.037879	17.159573
17.710218	30.248411	19.115107	21.11455	10.31269	11.07226
275.59125	399.54913	1405.8331	1216.3217	112.004295	53.35271
7824.3745	3917.4458	1954.1317	3345.0615	476.78085	249.55103
46.1333	31.872068	26.519575	31.233742	10.32165	9.938426
46.1333	31.872068	26.519575	31.233742	10.32165	9.938426
51.329147	85.97687	32.26762	68.74441	16.110529	17.535566
51.329147	85.97687	32.26762	68.74441	16.110529	17.535566
51.329147	85.97687	32.26762	68.74441	16.110529	17.535566
42778.598	57473.18	83381.445	86352.77	19923.291	10868.388
148.94994	172.5744	180.38971	221.09268	11.924299	7.314021
36.17393	359.27698	251.19432	367.38834	6.576742	4.9999995
41.011833	63.932007	28.507198	51.251507	8.771919	5.313991
46.827217	64.053856	22.657846	40.251503	13.436309	4.9999995
46.827217	64.053856	22.657846	40.251503	13.436309	4.9999995
310.75473	551.0306	260.51932	395.25458	118.33965	56.049164

30.574865	18.859343	54.780624	29.450455	7.0573153	7.012863
30.574865	18.859343	54.780624	29.450455	7.0573153	7.012863
30.574865	18.859343	54.780624	29.450455	7.0573153	7.012863
8.234385	27.913115	39.397995	25.743252	5.9258494	6.847015
5.902166	27.174564	8.516062	15.584317	3.3818736	2.3499577
140.1456	482.3006	307.22974	330.16547	9.920096	9.756766
47.118816	45.072422	50.937077	49.410236	7.334942	6.7713237
75.765854	19.505306	112.4942	49.664623	8.519726	7.899537
106.78001	61.60821	158.37155	90.16186	8.712161	8.734421
303.38013	163.27548	149.26431	107.50859	8.767781	8.658075
303.38013	163.27548	149.26431	107.50859	8.767781	8.658075
791.2411	460.224	1530.067	757.97864	10.948408	10.53186
5.7927155	22.278292	15.651379	20.091755	5.2087345	5.3796864
13.937241	9.322579	4.9999995	4.9999995	3.473651	4.42137
433.7217	175.48299	279.4798	201.24135	9.239677	9.643044
4.9999995	12.701419	10.538219	9.91832	4.6869926	4.235627
35.096867	14.941842	30.603638	18.864578	5.750132	6.142038
35.096867	14.941842	30.603638	18.864578	5.750132	6.142038
35.096867	14.941842	30.603638	18.864578	5.750132	6.142038
35.096867	14.941842	30.603638	18.864578	5.750132	6.142038
9.925563	7.4380164	4.9999995	4.9999995	3.3514404	3.9459188
11.398189	14.24421	10.22779	10.467509	5.936878	7.0388107
51.516277	109.169266	39.77293	64.99371	6.855126	7.3300815
335.4761	46.05294	53.071682	56.481014	8.253093	8.3535595
87.905815	12.483612	20.08167	12.41251	6.2259893	6.032944
55.389153	36.570026	76.03991	36.093056	7.360155	7.755804
259.07434	137.81052	360.803	213.66055	9.257543	9.659721
6.791268	19.146008	8.474061	8.306592	5.967749	4.70875
136.93471	78.07656	174.99043	110.92849	8.645444	9.272681
571.5282	87.32202	119.82334	81.39004	9.39818	8.832876
29.614586	25.100039	37.75484	22.348022	5.674142	5.8288918
29.614586	25.100039	37.75484	22.348022	5.674142	5.8288918
30.35411	11.15995	16.153307	12.269162	6.170934	6.639543
101.47321	61.63271	120.17716	68.063866	8.275042	8.381755
101.47321	61.63271	120.17716	68.063866	8.275042	8.381755
101.47321	61.63271	120.17716	68.063866	8.275042	8.381755
101.47321	61.63271	120.17716	68.063866	8.275042	8.381755
101.47321	61.63271	120.17716	68.063866	8.275042	8.381755
362.6533	394.92578	395.15454	326.5025	9.968546	9.258819
173.29825	60.26799	130.7862	72.779976	8.395008	8.9183035
38.28265	26.29105	26.107653	17.67897	10.459863	11.386386
114.21007	68.62345	87.36004	44.17626	8.132907	8.202782
23.156528	26.224173	9.2559	17.546356	5.735957	4.3904557
548.2862	343.49237	399.95132	302.97968	9.380915	9.545704
22.65059	14.33523	41.589203	21.02197	6.076598	6.7824187
119.43838	104.8186	73.36551	77.58104	6.911137	6.8527837
19.744463	31.32882	62.12702	35.45896	5.954436	6.6181297
9.440879	6.5167637	4.9999995	5.958649	4.429617	4.782638
167.65651	126.66384	206.338	142.18031	7.583443	7.5958595
726.70026	532.35925	948.3699	600.7752	11.794888	10.375759
10.41178	10.591329	12.65914	5.5633664	5.816922	5.605269
10.41178	10.591329	12.65914	5.5633664	5.816922	5.605269
17.957142	25.46832	25.446932	19.35063	6.1588383	6.7959785
17.957142	25.46832	25.446932	19.35063	6.1588383	6.7959785
17.957142	25.46832	25.446932	19.35063	6.1588383	6.7959785
30705.084	17006.344	23686.672	16620.912	15.633382	15.928686
13.711262	27.526144	8.078949	12.06431	6.4076834	5.532311
7.595521	13.06983	18.060938	10.07204	5.441412	6.1508594
17.11611	10.873049	20.951912	8.993396	5.114172	5.2504067
19.541462	16.419224	9.219217	14.565652	5.605556	5.253018
19.541462	16.419224	9.219217	14.565652	5.605556	5.253018
195.05408	91.848694	155.39217	100.40012	8.266052	8.706419

7.4418373	6.4885564	7.438882	7.212807	4.941611	5.2089453
7.4418373	6.4885564	7.438882	7.212807	4.941611	5.2089453
7.4418373	6.4885564	7.438882	7.212807	4.941611	5.2089453
4.7415357	4.9852037	6.865146	6.34809	4.5450134	6.0508866
4.2774057	7.7835183	6.714956	7.506278	2.921372	2.4279919
10.060663	10.335355	10.462447	10.437304	8.572818	9.604408
7.793617	7.4981923	6.518916	6.9353433	5.8783264	5.8579593
8.904858	7.228176	8.015696	7.7695537	6.1891227	6.200208
8.932674	8.882537	9.333313	9.137076	6.5830436	7.084866
8.4220705	8.839325	8.529139	8.349012	7.7106805	7.108122
8.4220705	8.839325	8.529139	8.349012	7.7106805	7.108122
11.336502	11.233106	10.291988	10.706762	9.167047	8.839019
5.5477166	5.507521	4.719424	5.1571293	3.79319	5.089304
4.6352534	4.795754	4.3115187	4.059448	3.1822972	4.017111
8.810124	8.832589	9.484455	9.094446	8.15608	7.740559
5.177009	3.7711673	4.5374103	4.581539	2.3467326	2.4279919
5.541789	5.961829	6.016682	5.9542017	4.8394785	5.199183
5.541789	5.961829	6.016682	5.9542017	4.8394785	5.199183
5.541789	5.961829	6.016682	5.9542017	4.8394785	5.199183
5.541789	5.961829	6.016682	5.9542017	4.8394785	5.199183
4.365098	3.6699626	4.1219177	3.510912	2.671759	2.4279919
2.3258038	6.7914143	5.1340356	5.9511466	3.3905108	5.032044
6.5075173	7.714591	7.210077	7.6056113	5.7195506	5.900133
7.8992696	7.5216265	8.135006	7.813172	7.4905844	5.898205
5.8094444	5.27034	6.21836	5.57234	5.3291874	3.8263674
7.065048	6.9781785	6.213038	6.5948143	5.4992824	5.529325
9.025662	8.411921	9.510809	9.292154	7.569208	7.335156
6.892893	5.801633	5.3921776	5.5581074	3.4431586	3.9676578
7.9750853	8.805205	9.513727	9.366946	6.9931664	7.1162405
9.995662	7.8504696	8.1506815	8.109154	8.59319	7.3283076
5.545344	6.5665956	6.187197	6.331216	4.5739746	4.025434
5.545344	6.5665956	6.187197	6.331216	4.5739746	4.025434
5.3541613	5.229281	5.5673723	5.4030333	4.517703	3.9683623
8.281262	8.45911	8.087202	8.436878	6.5251675	6.7055874
8.281262	8.45911	8.087202	8.436878	6.5251675	6.7055874
8.281262	8.45911	8.087202	8.436878	6.5251675	6.7055874
8.281262	8.45911	8.087202	8.436878	6.5251675	6.7055874
8.281262	8.45911	8.087202	8.436878	6.5251675	6.7055874
10.600171	9.997466	9.270403	9.616721	8.535715	8.625251
8.085681	8.50734	8.021917	8.096471	7.143567	6.25911
8.361902	9.72453	9.489901	9.61997	6.273701	7.274063
7.522901	6.73819	7.946139	7.540243	6.2818007	6.6394205
6.5527864	6.2731137	5.9309254	6.1903496	3.2397518	2.4279919
9.410206	11.284947	9.806539	10.470865	8.450581	7.2255673
5.812	6.723474	5.682771	6.3684483	3.7895691	3.2103212
7.2716055	8.884542	7.97	8.515529	6.6284337	6.2961993
5.9670444	6.463563	5.9904175	6.253614	4.037688	4.9212966
3.8263674	4.468565	4.1595173	3.9620757	3.5931666	4.261097
7.7705693	8.329194	10.181695	9.667421	7.043127	6.586653
12.564281	11.587498	10.635328	11.091865	9.165044	8.819457
5.205726	4.5491076	4.6070275	4.5107374	3.594371	4.094766
5.205726	4.5491076	4.6070275	4.5107374	3.594371	4.094766
5.3647127	6.070762	4.8686223	5.5904655	4.242664	4.954302
5.3647127	6.070762	4.8686223	5.5904655	4.242664	4.954302
5.3647127	6.070762	4.8686223	5.5904655	4.242664	4.954302
15.083024	15.646477	15.917739	15.771212	14.52811	14.303534
6.8907437	7.1033864	7.264512	7.249484	3.804501	3.6306033
4.8608136	8.166241	7.732921	7.954182	2.9142737	2.4279919
5.0403814	5.625095	4.7007823	5.1884604	3.3443246	3.142225
5.230659	5.628236	4.3940125	4.85678	3.9753897	2.4279919
5.230659	5.628236	4.3940125	4.85678	3.9753897	2.4279919
7.9430966	8.783311	7.7836957	8.054611	7.1229963	6.661609

4.9483643	4.3894362	6.0394397	5.2628264
4.9483643	4.3894362	6.0394397	5.2628264
4.9483643	4.3894362	6.0394397	5.2628264
3.1732607	5.006796	5.570228	5.045829
2.7705443	4.9631624	3.3842592	4.2834272
7.083676	9.360057	8.50676	8.907859
5.556664	5.746815	5.9409275	6.0519753
6.2136497	4.440398	7.069037	6.060962
6.7027397	6.2405453	7.5370455	6.9444456
8.192692	7.725996	7.4491997	7.215264
8.192692	7.725996	7.4491997	7.215264
9.574456	9.29452	10.827551	10.127174
2.7476184	4.6481557	4.2401876	4.6635265
3.8744755	3.3179784	2.3618152	2.352752
8.716484	7.8325357	8.377428	8.173292
2.350709	3.783199	3.6797721	3.5955896
5.140481	4.0383453	5.210716	4.5647826
5.140481	4.0383453	5.210716	4.5647826
5.140481	4.0383453	5.210716	4.5647826
5.140481	4.0383453	5.210716	4.5647826
3.4172828	2.9855325	2.3618152	2.352752
3.6064384	3.9621627	3.6339662	3.6791968
5.6787343	7.11353	5.5854106	6.4681454
8.334832	5.782071	5.9981976	6.260553
6.426432	3.758215	4.592008	3.934825
5.7845125	5.4237294	6.510322	5.577226
7.9639835	7.476079	8.732428	8.2678585
2.9334874	4.4124527	3.374927	3.3270118
7.0523367	6.6101685	7.6928453	7.2618694
9.104666	6.779653	7.153521	6.800193
4.907283	4.838899	5.508935	4.8257737
4.907283	4.838899	5.508935	4.8257737
4.9389505	3.591111	4.286369	3.9186964
6.6268044	6.2408614	7.1584487	6.5336685
6.6268044	6.2408614	7.1584487	6.5336685
6.6268044	6.2408614	7.1584487	6.5336685
6.6268044	6.2408614	7.1584487	6.5336685
6.6268044	6.2408614	7.1584487	6.5336685
8.4484215	9.075708	8.867599	8.892635
7.390444	6.203667	7.2755623	6.6345744
5.260334	4.908648	4.9746823	4.4675274
6.797867	6.409382	6.702343	5.884047
4.5643773	4.9037623	3.4965007	4.4554214
9.048474	8.87404	8.885891	8.78267
4.5336795	3.9719284	5.651573	4.73555
6.8601804	7.06087	6.4603744	6.732978
4.348615	5.1814675	6.2221513	5.5495715
3.353395	2.8061018	2.3618152	2.8386428
7.338382	7.3527207	7.9408245	7.6436634
9.448594	9.515962	10.137314	9.781049
3.484622	3.5114553	3.9380352	2.7419126
3.484622	3.5114553	3.9380352	2.7419126
4.219835	4.860328	4.9398108	4.6053796
4.219835	4.860328	4.9398108	4.6053796
4.219835	4.860328	4.9398108	4.6053796
14.848997	14.481314	14.881725	14.631461
3.8503883	4.9836664	3.3075917	3.8941212
3.0679133	3.829095	4.44475	3.6225193
4.15174	3.5504122	4.6562467	3.4498057
4.3335614	4.18026	3.4906552	4.178557
4.3335614	4.18026	3.4906552	4.178557
7.5585427	6.8534713	7.5092316	7.1109986

ASHGA5P020761	0.000326356	0.013106479	3.5909488	up	noncoding
ASHGA5P032302	0.00361608	0.038659181	2.0337598	up	noncoding
ASHGA5P033751	0.024967477	0.098084897	2.8377016	up	noncoding
ASHGA5P029834	0.013965155	0.072372115	2.8647469	up	noncoding
ASHGA5P038154	0.001656819	0.027211232	2.0453571	up	noncoding
ASHGA5P053871	0.00240466	0.031940985	2.7583027	up	noncoding
ASHGA5P020270	0.003380319	0.037497733	2.0798694	up	noncoding
ASHGA5P058787	0.027168392	0.102915644	16.3258507	up	noncoding
ASHGA5P020212	0.001432535	0.025327629	2.2029247	up	noncoding
ASHGA5P014601	0.009777337	0.061358967	2.2900153	up	noncoding
ASHGA5P018688	0.00063439	0.017037535	8.3673191	up	noncoding
ASHGA5P038962	0.000983368	0.021190872	2.7449262	up	noncoding
ASHGA5P036150	0.00335989	0.037423637	2.4034216	up	noncoding
ASHGA5P014592	0.006825268	0.05243611	2.1242504	up	noncoding
ASHGA5P031079	0.046244386	0.138653053	3.54109	up	noncoding
ASHGA5P018893	0.002080118	0.030104322	2.1754037	up	noncoding
ASHGA5P033707	0.00013393	0.010633934	4.2428513	up	noncoding
ASHGA5P033707	0.00013393	0.010633934	4.2428513	up	noncoding
ASHGA5P033707	0.00013393	0.010633934	4.2428513	up	noncoding
ASHGA5P033707	0.00013393	0.010633934	4.2428513	up	noncoding
ASHGA5P033707	0.00013393	0.010633934	4.2428513	up	noncoding
ASHGA5P033707	0.00013393	0.010633934	4.2428513	up	noncoding
ASHGA5P033707	0.00013393	0.010633934	4.2428513	up	noncoding
ASHGA5P033707	0.00013393	0.010633934	4.2428513	up	noncoding
ASHGA5P023344	0.00327018	0.036994062	2.9736007	up	noncoding
ASHGA5P031297	0.000641329	0.017037535	4.2764071	up	noncoding
ASHGA5P031297	0.000641329	0.017037535	4.2764071	up	noncoding
ASHGA5P019262	0.001316849	0.024205782	2.6385896	up	noncoding
ASHGA5P014826	0.003303647	0.037195092	3.0458737	up	noncoding
ASHGA5P051208	0.010201044	0.062583107	2.0992737	up	noncoding
ASHGA5P034997	0.000135467	0.01066938	6.9610936	up	noncoding
ASHGA5P046609	0.037154537	0.12233581	2.2310653	up	noncoding
ASHGA5P045761	0.032419657	0.113226764	2.9704575	up	noncoding
ASHGA5P028678	0.004683784	0.043516706	2.5778979	up	noncoding
ASHGA5P038916	0.023453944	0.094851476	2.0534159	up	noncoding
ASHGA5P022540	0.000168476	0.011182026	3.0095913	up	noncoding
ASHGA5P057661	0.01261375	0.068765796	2.2841049	up	noncoding
ASHGA5P037190	0.041213229	0.129607517	3.7464411	up	noncoding
ASHGA5P026751	0.038361935	0.124649282	2.0480592	up	noncoding
ASHGA5P035797	0.000276842	0.012382074	2.4151655	up	noncoding
ASHGA5P029350	0.002497826	0.032458616	2.994991	up	noncoding
ASHGA5P044556	0.004491299	0.04262056	2.5231497	up	noncoding
ASHGA5P044201	1.25934E-06	0.001921139	4.3241656	up	noncoding
ASHGA5P028572	0.00099745	0.02136839	3.5062174	up	noncoding
ASHGA5P050560	0.037299144	0.122530061	2.5466391	up	noncoding
ASHGA5P050560	0.037299144	0.122530061	2.5466391	up	noncoding
ASHGA5P050560	0.037299144	0.122530061	2.5466391	up	noncoding
ASHGA5P050560	0.037299144	0.122530061	2.5466391	up	noncoding
ASHGA5P044550	0.012820679	0.069304541	2.7172429	up	noncoding
ASHGA5P017547	0.011809563	0.066828817	2.4095549	up	noncoding
ASHGA5P017547	0.011809563	0.066828817	2.4095549	up	noncoding
ASHGA5P017547	0.011809563	0.066828817	2.4095549	up	noncoding
ASHGA5P017736	6.68713E-05	0.007985351	2.9989223	up	noncoding
ASHGA5P041202	0.001083178	0.022256512	2.9346585	up	noncoding
ASHGA5P016010	0.001379538	0.024821649	2.3705062	up	noncoding
ASHGA5P042986	0.000269532	0.012302262	5.3938367	up	noncoding
ASHGA5P042986	0.000269532	0.012302262	5.3938367	up	noncoding
ASHGA5P042986	0.000269532	0.012302262	5.3938367	up	noncoding
ASHGA5P056420	0.008612892	0.057872881	2.5909803	up	noncoding
ASHGA5P044152	0.014252965	0.073184388	2.5945167	up	noncoding
ASHGA5P039790	0.024889627	0.097963763	2.6099004	up	noncoding
ASHGA5P035922	0.009187003	0.059691382	2.5551076	up	noncoding

ENST00000519481	PVT1	GENCODE	542	chr8	+
ENST00000582718	AC010761.8	GENCODE	899	chr17	-
ENST00000581724	RP11-21G15.1	GENCODE	1908	chr18	+
ENST00000546968	RP11-44N21.1	GENCODE	965	chr14	+
NR_038320	LOC100507634	RefSeq	2048	chr1	+
NR_051970	MRPS2	RefSeq	1661	chr9	+
ENST00000510692	RP11-423H2.1	GENCODE	551	chr5	+
uc.247+	uc.247	UCR	361	chr9	+
ENST00000509713	RP11-553P9.2	GENCODE	284	chr4	+
ENST00000411727	RP11-33A14.1	GENCODE	2100	chr3	-
ENST00000456532	RP5-1158E12.3	GENCODE	375	chrX	-
ENST00000485898	RP11-18H7.1	GENCODE	545	chr3	+
NR_002909	SNHG3	RefSeq	2238	chr1	+
ENST00000411666	RP11-319I23.2	GENCODE	318	chr10	+
ENST00000366408	RP11-375F2.2	GENCODE	1129	chr1	-
ENST00000484945	FAM86DP	GENCODE	2238	chr3	-
uc010wzb.2	BX647607	UCSC_knowngene	2932	chr18	+
uc010wzb.2	BX647607	UCSC_knowngene	2932	chr18	+
uc010wzb.2	BX647607	UCSC_knowngene	2932	chr18	+
uc010wzb.2	BX647607	UCSC_knowngene	2932	chr18	+
uc010wzb.2	BX647607	UCSC_knowngene	2932	chr18	+
uc010wzb.2	BX647607	UCSC_knowngene	2932	chr18	+
uc010wzb.2	BX647607	UCSC_knowngene	2932	chr18	+
uc010wzb.2	BX647607	UCSC_knowngene	2932	chr18	+
NR_003697	SNHG15	RefSeq	837	chr7	-
ENST00000436656	GAS5	GENCODE	822	chr1	-
ENST00000436656	GAS5	GENCODE	822	chr1	-
NR_027139	SPIN3	RefSeq	1721	chrX	-
ENST00000413755	RP4-564F22.2	GENCODE	648	chr20	-
ENST00000359859	TMEM191A	GENCODE	736	chr22	+
ENST00000457457	AC016735.1	GENCODE	237	chr2	-
ENST00000416381	LL0XNC01-157D4.1	GENCODE	2012	chrX	+
ENST00000429567	RP11-470P21.2	GENCODE	443	chr9	+
ENST00000426991	ANKRD10-IT1	GENCODE	380	chr13	-
ENST00000462011	RP11-201E8.1	GENCODE	2305	chr3	+
ENST00000561384	CTD-2008A1.2	GENCODE	1045	chr15	-
TCONS_00020514	XLOC_009831	LincRNAs identified	4970	chr12	+
ENST00000457565	AP001464.4	GENCODE	323	chr21	-
ENST00000528717	RP11-315O6.1	GENCODE	692	chr11	+
TCONS_00003222	XLOC_002007	LincRNAs identified	535	chr2	-
ENST00000553270	RP11-1029J19.4	GENCODE	539	chr14	-
ENST00000560295	CTA-204B4.6	GENCODE	9346	chr8	-
ENST00000519738	RP11-150O12.1	GENCODE	733	chr8	-
uc001vgl.1	THSD1P1	UCSC_knowngene	3493	chr13	-
uc002sro.3	BC066991	UCSC_knowngene	1946	chr2	+
uc002sro.3	BC066991	UCSC_knowngene	1946	chr2	+
uc002sro.3	BC066991	UCSC_knowngene	1946	chr2	+
uc002sro.3	BC066991	UCSC_knowngene	1946	chr2	+
ENST00000518973	RP11-238K6.1	GENCODE	4171	chr8	-
ENST00000442967	AC017096.1	GENCODE	560	chr2	+
ENST00000442967	AC017096.1	GENCODE	560	chr2	+
ENST00000442967	AC017096.1	GENCODE	560	chr2	+
ENST00000445226	RP11-7O11.3	GENCODE	294	chr1	-
ENST00000389897	RP11-98D18.9	GENCODE	992	chr1	+
ENST00000425771	GAS5	GENCODE	242	chr1	-
NR_024466	LSM5	RefSeq	3022	chr7	-
NR_024466	LSM5	RefSeq	3022	chr7	-
NR_024466	LSM5	RefSeq	3022	chr7	-
TCONS_00004137	XLOC_001980	LincRNAs identified	1213	chr2	-
TCONS_00014734	XLOC_006819	LincRNAs identified	566	chr8	+
NR_037888	LOC100507266	RefSeq	2481	chr4	+
uc010fdz.3	OR7E91P	UCSC_knowngene	1287	chr2	+

128902922	129008896		TGGGCTTGAGAA' 5820	intronic antisense
27048504	27049895		TGCATTTTCTTTCTCTTGAAGGGCTC	natural antisense
10726629	10728537		CGAATCTGTTCTGTGGAAAATGCAT	intronic antisense
105561527	105565341		GAGATAAACTTTATGTGACCTTGGT	intergenic
55681080	55683128		CTAAACTGATGT/ 100507634	bidirectional
138392476	138396519		CCTGCCGTAGAT1 51116	exon sense-overlappi
177259752	177309517		GAAGGAACATGTCAAAGAAAACAA	intergenic
969153	969514		TTATTGCCCAGTGCTTTACGAGTTA	intergenic
136013548	136018804		CCGTGTCAAGCTCTTCCTCTTGTGA	intergenic
178051326	178103184		CAATGACTTATTGTTGTTGTTTGTTT	intronic antisense
45707508	45710920		CTGCGCGCAGGTGGCAGTGTAGAAC	intergenic
119814476	119817634		TAATATGGTTGTTACTACAAAGTTTA	intergenic
28832454	28837404	NR_036473	CCAGTCCCATCC(8420	exon sense-overlappi
120117056	120118715	ENST00000445161	CATCCAAAGATAATACTACCTTCAG	intergenic
168873142	169056243		TAATCCTCTTTTAAGAAGAAGGGGT	intergenic
75470710	75484108	ENST00000427587,	CTCCTGCCACAG(692099	intergenic
3594445	3597377		CTTGTCAGATGTCACTTTTCAGCAA(	intronic antisense
3594445	3597377		CTTGTCAGATGTCACTTTTCAGCAA(	intronic antisense
3594445	3597377		CTTGTCAGATGTCACTTTTCAGCAA(	intronic antisense
3594445	3597377		CTTGTCAGATGTCACTTTTCAGCAA(	intronic antisense
3594445	3597377		CTTGTCAGATGTCACTTTTCAGCAA(	intronic antisense
3594445	3597377		CTTGTCAGATGTCACTTTTCAGCAA(	intronic antisense
3594445	3597377		CTTGTCAGATGTCACTTTTCAGCAA(	intronic antisense
3594445	3597377		CTTGTCAGATGTCACTTTTCAGCAA(	intronic antisense
45022626	45026259	ENST00000580458,	AAGCAGTCTTTG1 285958	intergenic
173833038	173837127	ENST00000416952,	GAAATCTTTTAG/ 60674	bidirectional
173833038	173837127	ENST00000416952,	GAAATCTTTTAG/ 60674	bidirectional
57002802	57021988		TGGGCATAAGGT 169981	exon sense-overlappi
37050838	37063996		AAGACACTGGCGGGAGTGTAACATC	intergenic
21055219	21055955		GATGGTGACCCT(84222	intergenic
43228494	43233200	TCONS_00004225	TGGCCTGGAACCTAAGACTTGTCCA	intergenic
102094835	102121752		GTTTACTTGTTTGTCTGTGATAAATA	intergenic
72087381	72109429		GCCACACAAATAATGCATTTCTCAC	intronic antisense
111547405	111551519		GTAGCTTCTCATTTCTCTGTTTCTTG	intron sense-overlapp
99316907	99342664		TGGATATTACTTAGCTTAATAGTTC(	intergenic
45119397	45176892	ENST00000558556,	CTAAAACCAGGTGATCGTGTTGCCA	intergenic
91069064	91078196		AAGAAACAATGAAAGCCACAAGTT1	intergenic
14371996	14389013	ENST00000446288	GTAGTAGTTTTGCATCTATGGGATT(	intergenic
102323649	102332739		CCTGGGTTGGGCTACTACTGCCTAT(	natural antisense
20251894	20254219		TTTCATTTATCCTTAATCCTTTTGCC	intergenic
102095320	102098867		TAAGTAGCTGGTGTCTGGCAAGAGC	intergenic
141530254	141539600		ACCTCCTTACATAACCCCTAACATG(	intergenic
37308280	37411701		TCAGACTGAACTGGCAGACACTGCA	intergenic
52741846	52768602	ENST00000416599	CTGCTATGGTTG1 374500	intergenic
86831129	86845887		CTGGAAAAATTAAGCAGGTGTCCTA	intronic antisense
86831129	86845887		CTGGAAAAATTAAGCAGGTGTCCTA	natural antisense
86831129	86845887		CTGGAAAAATTAAGCAGGTGTCCTA	natural antisense
86831129	86845887		CTGGAAAAATTAAGCAGGTGTCCTA	natural antisense
138821686	139095813		TGTGTCATATTCCAGCTCTCAGAGG(	intergenic
200322422	200324615		TTATTAGTTGCGTTGAGTCATCAGG(	intronic antisense
200322422	200324615		TTATTAGTTGCGTTGAGTCATCAGG(	intronic antisense
200322422	200324615		TTATTAGTTGCGTTGAGTCATCAGG(	natural antisense
44410043	44411958	ENST00000412378,	ATTTCCGGAGGGAACATCCCAAAAC	bidirectional
151763317	151766878	ENST00000533481	TTTCTGGAAAACATGAAATAGGCTT	natural antisense
173835004	173836178		AGTTGAAATGTT1 60674	intergenic
32524944	32530475		ATGGTAAAATGT(23658	exon sense-overlappi
32524944	32530475		ATGGTAAAATGT(23658	exon sense-overlappi
32524944	32530475		ATGGTAAAATGT(23658	exon sense-overlappi
9344858	9346352		TGACTCAAGTCTCTTAAGGAAATTC	intergenic
63924579	63925933		AAATGCTGGAGGTAAGAGCTTTGGC	intergenic
4543857	4712664		CTGCATCTTTTTT 100507266	natural antisense
71251204	71257060	NR_002185, NR_02	TCCATCCTTTTTC 79315	intergenic

ENST00000539634	TMEM75	transmembrane prote	-	128959125	128960591
NM_000984	RPL23A	60S ribosomal protei	+	27046999	27051374
NM_022068	PIEZO2	piezo-type mechanos	-	10670243	11148761
NM_015306	USP24	ubiquitin carboxyl-te	-	55532031	55681039
NM_016034	MRPS2	28S ribosomal protei	+	138392476	138396519
ENST00000437510	KCNMB2	potassium large cond	+	177990719	178546150
NM_001048199	RCC1	regulator of chromos	+	28832454	28865708
NM_001003809	DLGAP1	disks large-associate	-	3496029	3845358
NM_001242761	DLGAP1	disks large-associate	-	3502142	4455266
NM_001242762	DLGAP1	disks large-associate	-	3496029	3874767
NM_001242763	DLGAP1	disks large-associate	-	3496029	3874767
NM_001242764	DLGAP1	disks large-associate	-	3496029	3874253
NM_001242765	DLGAP1	disks large-associate	-	3502142	3845358
NM_001242766	DLGAP1	disks large-associate	-	3496029	3845358
NM_004746	DLGAP1	disks large-associate	-	3496029	4455266
NM_001122770	ZBTB37	zinc finger and BTB	+	173837492	173855774
NM_032522	ZBTB37	zinc finger and BTB	+	173837492	173842778
NM_001010862	SPIN3	spindlin-3	-	57017263	57021988
NM_001163	APBA1	amyloid beta A4 prec	-	72042448	72287275
NM_017664	ANKRD10	ankyrin repeat domai	-	111530886	111567416
NM_052932	TMEM123	porimin precursor	-	102267055	102323775
NM_001198954	RNF103-CHMP3	RNF103-CHMP3 pro	-	86730552	86948245
NM_001198951	RNF103	E3 ubiquitin-protein	-	86830515	86851000
NM_001198952	RNF103	E3 ubiquitin-protein	-	86843539	86851000
NM_005667	RNF103	E3 ubiquitin-protein	-	86830515	86851000
NM_001172517	SATB2	DNA-binding protein	-	200134222	200329831
NM_015265	SATB2	DNA-binding protein	-	200134222	200335989
NM_001172509	SATB2	DNA-binding protein	-	200134222	200322819
NM_014652	IPO13	importin-13	+	44412477	44433694
ENST00000368822	TDRKH	tudor and KH domai	-	151745969	151763892
NM_001130710	LSM5	U6 snRNA-associate	-	32524944	32534870
NM_001139499	LSM5	U6 snRNA-associate	-	32524944	32534870
NM_012322	LSM5	U6 snRNA-associate	-	32524944	32530023
ENST00000507908	STX18	syntaxin 18 [Source:]	-	4426899	4544073

836.514728	129.864258	9.03652	7.192155	505.18335	378.03052
87.489687	28.864709	6.081648	5.057498	97.927086	94.02874
60.202827	10.704328	5.147191	3.642469	68.27582	112.0711
949.38715	264.827876	9.485556	7.967148	1050.2535	859.8863
32.873893	10.954887	4.673632	3.641279	37.753407	29.155264
227.441718	48.904981	7.35533	5.891549	245.46184	386.1449
481.512523	132.675847	8.44557	7.389077	380.17163	535.4661
1966.87597	184.38394	9.82043	5.791343	1589.3552	2147.3623
33.947156	10.905805	4.743489	3.604069	38.922237	34.812763
59.327736	18.137539	5.520439	4.325081	85.74362	56.309418
97.342739	9.431364	6.222347	3.157581	86.228836	85.556335
830.296093	169.080802	9.131996	7.675229	529.00037	466.9964
2384.336383	577.588245	10.744199	9.479109	2341.3035	3153.543
45.844269	12.774654	4.993935	3.906981	74.10812	76.15251
250.812137	32.423743	6.919065	5.094871	345.8599	245.14085
411.905472	109.598237	8.230152	7.108868	398.85773	570.9746
32.739294	5.322697	4.64493	2.559896	38.420845	32.467857
32.739294	5.322697	4.64493	2.559896	38.420845	32.467857
32.739294	5.322697	4.64493	2.559896	38.420845	32.467857
32.739294	5.322697	4.64493	2.559896	38.420845	32.467857
32.739294	5.322697	4.64493	2.559896	38.420845	32.467857
32.739294	5.322697	4.64493	2.559896	38.420845	32.467857
32.739294	5.322697	4.64493	2.559896	38.420845	32.467857
32.739294	5.322697	4.64493	2.559896	38.420845	32.467857
936.326183	185.013568	9.411399	7.839188	1240.7761	1347.5784
913.435357	130.899879	9.412524	7.316125	918.9117	981.72144
913.435357	130.899879	9.412524	7.316125	918.9117	981.72144
188.2541	45.664229	7.196969	5.797202	205.89844	165.08519
133.805927	24.882243	6.53762	4.930764	149.11726	207.64505
111.472028	33.346912	6.40367	5.33378	165.9263	82.69084
761.971405	63.916325	9.063842	6.264528	668.7228	426.64426
39.623277	11.278988	4.746718	3.588985	46.45542	29.849094
406.065853	134.810156	8.259907	6.689222	406.24155	473.4276
584.682132	145.580207	8.658392	7.292197	566.17096	216.42099
26.108835	7.465683	4.053614	3.015588	41.487553	57.57049
307.957783	62.336545	7.866223	6.276656	354.74094	415.45114
85.527377	25.257029	6.011344	4.819715	99.003685	135.81952
49.450386	7.970721	4.923924	3.018403	23.071936	9.796665
1137.067583	337.936195	9.714034	8.679777	1328.8146	1249.182
20.48518	6.045375	3.951572	2.67945	24.751976	13.592128
110.587801	20.141164	6.213126	4.630575	144.92719	225.7964
20088.79967	4372.049233	13.842653	12.507427	21927.998	12805.145
34.993987	5.798708	4.781251	2.66883	32.191254	25.478743
128.424107	29.274214	6.606376	4.796461	146.1815	85.86514
29.285924	8.842498	4.288205	2.93961	64.92344	34.382328
29.285924	8.842498	4.288205	2.93961	64.92344	34.382328
29.285924	8.842498	4.288205	2.93961	64.92344	34.382328
29.285924	8.842498	4.288205	2.93961	64.92344	34.382328
30.966804	8.19138	4.563122	3.120978	35.623245	37.74295
1690.203543	452.298735	10.238618	8.969852	2069.2354	2245.0667
1690.203543	452.298735	10.238618	8.969852	2069.2354	2245.0667
1690.203543	452.298735	10.238618	8.969852	2069.2354	2245.0667
261.996325	53.14698	7.636064	6.05162	251.81653	292.3002
68.329467	15.734986	5.748231	4.195038	61.606358	58.470306
321.897778	81.051374	7.893586	6.648391	288.7039	251.7086
87.907721	13.637705	6.084824	3.653513	92.66182	72.78077
87.907721	13.637705	6.084824	3.653513	92.66182	72.78077
87.907721	13.637705	6.084824	3.653513	92.66182	72.78077
44.400163	12.516578	5.086873	3.713375	42.623695	36.98664
21.450793	6.697419	4.072775	2.697309	23.25398	20.073362
473.106722	111.86184	8.338178	6.954183	580.64703	492.2459
703.620282	144.722189	8.770584	7.4172	532.9557	184.37671

616.279	1944.0717	382.1763	1193.3475	71.75444	54.03355
86.95536	58.191143	88.33726	99.498535	22.398779	13.753479
14.611287	101.94587	12.54428	51.768604	8.374387	9.391659
1128.3287	758.4317	840.9652	1058.4575	274.3712	55.689526
49.572735	34.288918	19.65385	26.819185	16.709618	9.799296
101.01511	175.72478	224.73595	231.56773	53.02958	34.129288
245.96413	746.662	378.97128	601.84	116.2659	122.39691
569.6472	126.64312	4791.099	2577.149	240.71153	70.22773
41.931755	25.704584	29.626913	32.684685	10.251381	5.317704
58.724525	41.365227	60.364418	53.459206	12.19194	6.9922247
83.16804	72.485435	123.137886	133.4799	11.79304	4.9999995
551.50134	1534.5552	543.31055	1356.4127	140.31696	73.84108
1291.216	2906.609	1917.8693	2695.4775	428.26108	348.39545
49.8272	30.99699	17.489193	26.491604	13.979941	12.339061
345.49246	4.9999995	325.26944	238.11017	39.109463	15.785018
224.85512	258.9052	513.50934	504.33084	122.90632	132.2966
40.092106	16.93531	32.21416	36.305485	5.3666525	4.9999995
40.092106	16.93531	32.21416	36.305485	5.3666525	4.9999995
40.092106	16.93531	32.21416	36.305485	5.3666525	4.9999995
40.092106	16.93531	32.21416	36.305485	5.3666525	4.9999995
40.092106	16.93531	32.21416	36.305485	5.3666525	4.9999995
40.092106	16.93531	32.21416	36.305485	5.3666525	4.9999995
40.092106	16.93531	32.21416	36.305485	5.3666525	4.9999995
40.092106	16.93531	32.21416	36.305485	5.3666525	4.9999995
887.836	819.1762	528.7182	793.8722	166.18831	229.28925
812.1985	1205.8599	575.2104	986.7102	107.11341	84.575714
812.1985	1205.8599	575.2104	986.7102	107.11341	84.575714
213.24818	200.17767	144.529	200.58612	39.089176	35.614876
58.06603	59.712025	178.8266	149.4686	22.88791	28.749172
143.50508	85.98903	89.366325	101.35459	25.674273	18.78721
889.4203	376.56747	1157.1407	1053.3329	44.17524	43.96116
94.95693	27.976532	16.355467	22.146217	7.649676	7.907024
336.53735	273.5584	459.94904	486.68118	51.57739	34.713696
894.02454	807.6349	344.4446	679.3968	185.91156	55.693592
13.21485	7.5580926	16.38717	20.434855	9.390466	10.214792
227.56606	312.06683	228.96149	308.96024	58.60884	42.855465
69.98766	57.635395	75.33459	75.38341	24.311165	12.6011
31.625261	44.352028	84.85564	103.000786	7.2114406	7.616641
1303.364	1252.987	626.2331	1061.8248	239.64209	186.83691
15.934309	40.232445	12.402941	15.997279	8.209423	4.9999995
34.453594	88.35486	77.36935	92.62541	17.339079	22.90287
29523.848	20760.9	15045.005	20469.902	3923.671	3508.8
29.76082	33.80381	46.840374	41.888924	5.5034714	4.9999995
192.27019	131.0838	84.543915	130.6001	36.933002	9.436285
22.104607	8.904611	25.960289	19.44027	10.59582	4.9999995
22.104607	8.904611	25.960289	19.44027	10.59582	4.9999995
22.104607	8.904611	25.960289	19.44027	10.59582	4.9999995
22.104607	8.904611	25.960289	19.44027	10.59582	4.9999995
17.46114	22.48445	38.30351	34.18553	10.487001	4.9999995
1401.927	809.67206	1818.8499	1796.4702	447.5918	176.50122
1401.927	809.67206	1818.8499	1796.4702	447.5918	176.50122
1401.927	809.67206	1818.8499	1796.4702	447.5918	176.50122
192.0003	189.08392	337.3753	309.4017	42.9286	51.577305
60.54177	82.62115	66.41173	80.32549	14.011793	11.356819
303.67667	528.219	189.31342	369.76508	77.27944	51.479935
125.81034	91.49616	60.79145	83.905785	14.363599	5.6327343
125.81034	91.49616	60.79145	83.905785	14.363599	5.6327343
125.81034	91.49616	60.79145	83.905785	14.363599	5.6327343
42.16571	27.605673	61.433765	55.585495	8.798902	10.5014305
15.7268	18.339588	29.958755	21.352276	4.9999995	4.9999995
660.0791	574.73566	125.56612	405.36652	124.46359	48.696472
817.5101	286.29388	1289.8186	1110.7667	117.33602	51.639034

89.860695	251.53954	137.4392	174.55812	8.497623	8.268949
35.8889	21.653921	45.453545	34.039627	6.1648808	6.3426485
14.767902	15.991221	6.226704	9.474096	5.632655	6.589115
521.50244	73.73736	459.17712	204.48961	9.563779	9.410531
15.245411	10.87136	7.8538	5.2498355	4.774437	4.80267
58.895226	33.83976	67.97535	45.560684	7.484921	8.300746
122.12621	121.52842	185.81757	127.92007	8.110435	8.755491
757.50525	4.9999995	27.487204	5.371927	10.166045	10.6527195
20.530073	8.191839	9.531383	11.612451	4.818328	5.037894
25.525162	18.044476	29.647434	16.424	5.9681554	5.663059
18.940012	4.9999995	4.9999995	10.855131	5.9762087	6.221996
182.46591	212.9865	236.21425	168.66011	8.561582	8.5586
522.707	532.1921	1032.0411	601.93274	10.717249	11.208283
20.003258	8.034113	10.22693	12.064621	5.756035	6.0636916
68.608986	13.748491	33.161797	24.128704	7.9818645	7.671328
117.47238	98.74463	99.30269	86.8668	8.183573	8.848739
5.3019505	4.9999995	6.267582	4.9999995	4.798562	4.9461308
5.3019505	4.9999995	6.267582	4.9999995	4.798562	4.9461308
5.3019505	4.9999995	6.267582	4.9999995	4.798562	4.9461308
5.3019505	4.9999995	6.267582	4.9999995	4.798562	4.9461308
5.3019505	4.9999995	6.267582	4.9999995	4.798562	4.9461308
5.3019505	4.9999995	6.267582	4.9999995	4.798562	4.9461308
5.3019505	4.9999995	6.267582	4.9999995	4.798562	4.9461308
5.3019505	4.9999995	6.267582	4.9999995	4.798562	4.9461308
105.921486	280.25812	163.46057	164.96367	9.799103	10.03167
135.98909	107.45928	223.96794	126.29384	9.375414	9.5945215
135.98909	107.45928	223.96794	126.29384	9.375414	9.5945215
49.4664	35.357975	71.72345	42.733498	7.232593	7.1254773
19.961082	32.397495	26.63965	18.65815	6.764688	7.444423
37.70992	44.78749	32.831448	40.29113	6.91716	6.174479
50.966618	59.203846	107.205284	77.9858	8.915527	8.427277
19.574883	4.9999995	17.9929	9.549443	5.0787997	4.83372
64.0267	31.30729	438.30292	188.93294	8.206002	8.579478
316.92752	97.41734	137.98595	79.54528	8.665215	7.5003695
10.188839	4.9999995	4.9999995	4.9999995	4.9119267	5.6922636
84.79743	61.923885	66.52263	59.31102	8.016042	8.39285
50.838333	25.607439	20.94156	17.242577	6.1781454	6.8519626
4.9999995	17.996248	4.9999995	4.9999995	4.0655284	3.3751245
284.9839	338.76553	594.89044	382.4983	9.91113	9.925661
5.451557	7.6112695	4.9999995	4.9999995	4.171854	3.8023777
19.355122	23.476904	22.200127	15.572879	6.7255154	7.562858
4508.6274	5255.198	4693.1274	4342.8716	13.968516	13.18249
4.9999995	4.9999995	7.363721	6.9250584	4.547674	4.623199
75.99122	13.65938	20.658619	18.96678	6.7364388	6.2267313
22.459167	4.9999995	4.9999995	4.9999995	5.5615935	5.0220647
22.459167	4.9999995	4.9999995	4.9999995	5.5615935	5.0220647
22.459167	4.9999995	4.9999995	4.9999995	5.5615935	5.0220647
22.459167	4.9999995	4.9999995	4.9999995	5.5615935	5.0220647
13.93196	5.697343	7.0501475	6.981827	4.6922035	5.1426387
1052.2054	309.71152	428.949	298.83347	10.536766	10.713436
1052.2054	309.71152	428.949	298.83347	10.536766	10.713436
1052.2054	309.71152	428.949	298.83347	10.536766	10.713436
43.04674	60.33093	70.848015	50.15029	7.5190434	7.9217434
14.52427	10.4024	27.598804	16.515827	5.4874926	5.710718
112.423805	79.44754	88.61884	77.058685	7.720291	7.7092113
36.524765	11.16819	4.9999995	9.136941	6.085165	6.002578
36.524765	11.16819	4.9999995	9.136941	6.085165	6.002578
36.524765	11.16819	4.9999995	9.136941	6.085165	6.002578
30.970058	8.111739	8.969066	7.748272	4.9528174	5.1142926
13.249782	6.934732	4.9999995	4.9999995	4.0762877	4.3039985
222.58066	65.72781	134.83911	74.8634	8.705375	8.63079
180.07262	170.35199	194.69731	154.23616	8.571802	7.285914

8.894337	10.595957	8.321607	9.64065	6.3971715	6.604294
6.1269917	5.480972	6.258562	6.1158304	4.7231193	4.585809
3.5454535	6.321778	3.5886724	5.205475	3.273707	4.006923
9.764	9.26024	9.454134	9.46065	8.360149	6.651796
5.3151608	4.661604	4.19898	4.2889414	4.294446	4.074335
6.3390436	7.1333995	7.564148	7.30972	5.964088	5.932051
7.6118402	9.235538	8.308131	8.651986	7.0992684	7.78406
8.793183	6.654048	11.920902	10.73568	8.168179	6.9815273
5.069659	4.213138	4.7473097	4.5746036	3.58381	3.144012
5.5609856	4.954214	5.7259383	5.25028	3.8373413	3.5682375
6.060545	5.8176103	6.7237515	6.533968	3.7890573	2.4279919
8.749801	10.269084	8.832876	9.820034	7.3613663	7.051773
9.972047	11.164158	10.606932	10.796526	9.003152	9.3150625
5.3222795	4.5089884	4.042925	4.2696924	4.031591	4.4201493
8.0946	2.3221264	8.0933895	7.3510795	5.521658	4.7992864
7.4899263	7.7054634	8.748426	8.404782	7.174183	7.8976955
5.008727	3.5339692	4.865244	4.716948	2.6300874	2.4279919
5.008727	3.5339692	4.865244	4.716948	2.6300874	2.4279919
5.008727	3.5339692	4.865244	4.716948	2.6300874	2.4279919
5.008727	3.5339692	4.865244	4.716948	2.6300874	2.4279919
5.008727	3.5339692	4.865244	4.716948	2.6300874	2.4279919
5.008727	3.5339692	4.865244	4.716948	2.6300874	2.4279919
5.008727	3.5339692	4.865244	4.716948	2.6300874	2.4279919
5.008727	3.5339692	4.865244	4.716948	2.6300874	2.4279919
9.428208	9.363692	8.79198	9.05374	7.6226	8.694846
9.311349	9.920622	8.912669	9.360567	6.9798174	7.2533555
9.311349	9.920622	8.912669	9.360567	6.9798174	7.2533555
7.4197674	7.327385	6.9564524	7.12014	5.520661	5.998509
5.545674	5.520396	7.254458	6.6960816	4.7558317	5.6835895
6.8414755	6.0714536	6.2740397	6.1434126	4.926239	5.05281
9.430864	8.236768	9.915837	9.4567795	5.698315	6.3046255
6.2479644	4.3412256	3.955556	4.0230412	3.1308587	3.7448554
8.060533	7.7751064	8.586667	8.351657	5.9241323	5.9573283
9.441317	9.341576	8.171133	8.830743	7.786166	6.651963
3.3966525	2.4451997	3.957456	3.9181871	3.447054	4.135071
7.50675	7.9639835	7.5943546	7.72336	6.1091213	6.267876
5.816922	5.4655304	6.0297003	5.7258043	4.844914	4.454622
4.673996	5.060999	6.2007833	6.167113	3.0429091	3.6886053
9.9883175	9.97336	9.021271	9.464466	8.158214	8.398788
3.6750293	4.910768	3.5722787	3.5771267	3.2412121	2.4279919
4.793169	6.1118073	6.0700817	6.015326	4.3543115	5.345284
14.557114	14.03268	13.594554	13.720564	12.236178	12.683071
4.586735	4.639167	5.381213	4.909521	2.662697	2.4279919
7.268386	6.7052193	6.196307	6.5051737	5.444116	4.0140476
4.153712	2.5621135	4.580166	3.849578	3.632964	2.4279919
4.153712	2.5621135	4.580166	3.849578	3.632964	2.4279919
4.153712	2.5621135	4.580166	3.849578	3.632964	2.4279919
4.153712	2.5621135	4.580166	3.849578	3.632964	2.4279919
3.8056703	3.9955504	5.1060743	4.636594	3.6180618	2.4279919
10.082535	9.345686	10.533146	10.220142	9.066629	8.305659
10.082535	9.345686	10.533146	10.220142	9.066629	8.305659
10.082535	9.345686	10.533146	10.220142	9.066629	8.305659
7.2651157	7.2413	8.1443205	7.72486	5.658372	6.5372953
5.6091385	6.012456	5.8547955	5.814785	4.035068	4.2967763
7.9133534	8.726166	7.3294683	7.9630256	6.495064	6.534383
6.651796	6.1626234	5.735355	5.871429	4.07252	3.2301643
6.651796	6.1626234	5.735355	5.871429	4.07252	3.2301643
6.651796	6.1626234	5.735355	5.871429	4.07252	3.2301643
5.0758295	4.3236876	5.749824	5.3047853	3.3494494	4.179638
3.654105	3.6609128	4.7650294	3.9763172	2.3467326	2.4279919
9.001109	8.848739	6.7508183	8.0922365	7.191397	6.4540367
9.322625	7.844609	10.064374	9.53418	7.111969	6.539441

6.4582605	8.401984	7.3399906	7.951232
5.1691947	4.6034703	5.7743945	5.489002
3.9514565	4.141452	2.9529572	3.528316
8.980853	6.524727	9.084449	8.200914
3.9939806	3.5501282	3.2686865	2.6661005
5.866482	5.302248	6.353612	5.9308124
6.8937016	7.283399	7.7867117	7.487323
9.509842	2.3408759	5.054576	2.6930597
4.4006376	3.1229177	3.538158	3.834876
4.6980534	4.3175263	5.168108	4.3612223
4.2922525	2.3408759	2.3618152	3.733494
7.4622574	8.142045	8.13574	7.898191
8.984613	9.514803	10.271769	9.785257
4.3666906	3.0954673	3.6336277	3.894363
6.0774426	3.9040053	5.323045	4.9437895
6.8368106	6.9654326	6.8895445	6.8895445
2.6458135	2.3408759	2.9618556	2.352752
2.6458135	2.3408759	2.9618556	2.352752
2.6458135	2.3408759	2.9618556	2.352752
2.6458135	2.3408759	2.9618556	2.352752
2.6458135	2.3408759	2.9618556	2.352752
2.6458135	2.3408759	2.9618556	2.352752
2.6458135	2.3408759	2.9618556	2.352752
2.6458135	2.3408759	2.9618556	2.352752
6.691194	8.570687	7.5843334	7.871467
7.0427375	7.093479	8.062209	7.465149
7.0427375	7.093479	8.062209	7.465149
5.62692	5.373929	6.4281397	5.8350544
4.3632574	5.228952	5.0058284	4.547125
5.2382026	5.736881	5.3066463	5.7419
5.6655035	6.1775827	7.000608	6.740534
4.3367057	2.3408759	4.4402013	3.5404134
5.9831204	5.180651	9.0156355	8.074465
8.259554	6.941948	7.3447433	6.7688084
3.4559624	2.3408759	2.3618152	2.352752
6.373998	6.2511463	6.3251686	6.3326244
5.662679	4.8692756	4.6556425	4.4311585
2.350709	4.313629	2.3618152	2.352752
8.099115	8.851284	9.450988	9.120272
2.675927	3.017004	2.3618152	2.352752
4.321811	4.735638	4.744484	4.281919
12.118268	12.845834	12.487051	12.674161
2.350709	2.3408759	3.1792796	3.051425
6.21671	3.8956127	4.6343026	4.5739746
4.5212617	2.3408759	2.3618152	2.352752
4.5212617	2.3408759	2.3618152	2.352752
4.5212617	2.3408759	2.3618152	2.352752
4.5212617	2.3408759	2.3618152	2.352752
3.8737085	2.623707	3.1187534	3.0636473
9.977817	8.72662	8.981082	8.761304
9.977817	8.72662	8.981082	8.761304
9.977817	8.72662	8.981082	8.761304
5.4234524	6.2056823	6.409084	6.075832
3.92721	3.4819586	5.0602994	4.3689175
6.776666	6.6380076	6.722672	6.723552
5.1931844	3.5921187	2.3618152	3.4712727
5.1931844	3.5921187	2.3618152	3.4712727
5.1931844	3.5921187	2.3618152	3.4712727
4.968224	3.1089723	3.4504693	3.2234952
3.8030305	2.8915336	2.3618152	2.352752
7.740992	6.3430276	7.318266	6.67738
7.4448066	7.786166	7.851634	7.769183

ASHGA5P032274	0.013032318	0.069856339	2.3337862	up	noncoding
ASHGA5P026228	0.033493999	0.115418598	2.4718196	up	noncoding
ASHGA5P036099	2.14807E-05	0.005043652	2.3995986	up	noncoding
ASHGA5P043445	0.004303512	0.041611144	2.8611717	up	noncoding
ASHGA5P054840	0.010165093	0.062488634	2.3276176	up	noncoding
ASHGA5P020601	0.001826841	0.02866799	2.2510834	up	noncoding
ASHGA5P044381	0.001737545	0.027815258	2.5550818	up	noncoding
ASHGA5P017889	0.017168631	0.079799097	2.9037718	up	noncoding
ASHGA5P017889	0.017168631	0.079799097	2.9037718	up	noncoding
ASHGA5P017889	0.017168631	0.079799097	2.9037718	up	noncoding
ASHGA5P017889	0.017168631	0.079799097	2.9037718	up	noncoding
ASHGA5P015403	0.011394598	0.065655489	2.7828953	up	noncoding
ASHGA5P015191	2.52666E-05	0.005206419	2.4887292	up	noncoding
ASHGA5P050656	0.012665523	0.068811258	2.4902912	up	noncoding
ASHGA5P043479	1.26335E-05	0.004261616	2.4016576	up	noncoding
ASHGA5P034812	0.012978822	0.069832275	2.013277	up	noncoding
ASHGA5P038136	0.001393964	0.025029174	2.430003	up	noncoding
ASHGA5P033306	0.000647953	0.017096671	2.7261633	up	noncoding
ASHGA5P000036	0.005414185	0.046810253	2.1060934	up	noncoding
ASHGA5P047996	0.005746881	0.048170833	8.2507483	up	noncoding
ASHGA5P036258	1.10965E-05	0.004010044	2.6559998	up	noncoding
ASHGA5P041295	0.00238863	0.031905667	2.2322738	up	noncoding
ASHGA5P043490	0.000472078	0.015197114	5.5270853	up	noncoding
ASHGA5P023709	0.006940886	0.052901715	2.1290861	up	noncoding
ASHGA5P033399	0.000384067	0.014011107	7.6429028	up	noncoding
ASHGA5P015330	0.000511744	0.015567285	2.583859	up	noncoding
ASHGA5P042183	0.000554952	0.016097331	3.3573274	up	noncoding
ASHGA5P030284	0.023210506	0.09436573	4.3668823	up	noncoding
ASHGA5P037898	0.008927988	0.058937658	2.0899839	up	noncoding
ASHGA5P030309	0.001645143	0.027211232	4.1743029	up	noncoding
ASHGA5P031058	0.000319877	0.012936329	3.6730517	up	noncoding
ASHGA5P031058	0.000319877	0.012936329	3.6730517	up	noncoding
ASHGA5P028122	0.011277818	0.065455073	2.2637814	up	noncoding
ASHGA5P018325	0.028251748	0.104954123	6.7333094	up	noncoding
ASHGA5P039131	0.008251199	0.056900112	2.4947726	up	noncoding
ASHGA5P056248	0.041786307	0.130815553	2.6018198	up	noncoding
ASHGA5P054984	0.004133046	0.041062628	3.1553104	up	noncoding
ASHGA5P044811	0.000596161	0.016430598	2.1608636	up	noncoding
ASHGA5P047804	0.01137833	0.065649171	2.1714267	up	noncoding
ASHGA5P045129	0.018184254	0.081962493	2.5564556	up	noncoding
ASHGA5P036921	0.013670431	0.071680418	3.0996092	up	noncoding
ASHGA5P056533	0.006149681	0.050044579	3.0818061	up	noncoding
ASHGA5P042878	0.003487684	0.038135018	2.4067762	up	noncoding
ASHGA5P035813	0.002284109	0.031352605	2.3294987	up	noncoding
ASHGA5P035813	0.002284109	0.031352605	2.3294987	up	noncoding
ASHGA5P035813	0.002284109	0.031352605	2.3294987	up	noncoding
ASHGA5P035813	0.002284109	0.031352605	2.3294987	up	noncoding
ASHGA5P016554	7.48133E-05	0.008283845	3.2803919	up	noncoding
ASHGA5P016554	7.48133E-05	0.008283845	3.2803919	up	noncoding
ASHGA5P016554	7.48133E-05	0.008283845	3.2803919	up	noncoding
ASHGA5P014628	0.007296089	0.053739578	2.0946521	up	noncoding
ASHGA5P049689	2.7424E-05	0.005394108	2.0514272	up	noncoding
ASHGA5P044274	0.008764831	0.058350177	2.1444457	up	noncoding
ASHGA5P043050	0.000562061	0.016101946	4.2267441	up	noncoding
ASHGA5P032571	3.29085E-05	0.005933463	5.4093962	up	noncoding
ASHGA5P028564	0.017007109	0.079410859	3.2122168	up	noncoding
ASHGA5P019960	0.034552007	0.117566482	2.0350908	up	noncoding
ASHGA5P019960	0.034552007	0.117566482	2.0350908	up	noncoding
ASHGA5P049516	0.004307973	0.041611144	2.6845665	up	noncoding
ASHGA5P053120	0.011042448	0.064989371	2.4086061	up	noncoding
ASHGA5P053120	0.011042448	0.064989371	2.4086061	up	noncoding
ASHGA5P053120	0.011042448	0.064989371	2.4086061	up	noncoding

BE174175R		LincRNAs identified	327	chr10	-
ENST00000528194	RP11-222N13.1	GENCODE	546	chr11	+
uc021vmx.1	DKFZp434K1323	UCSC_knowngene	507	chr2	+
ENST00000439364	RP5-982E9.1	GENCODE	597	chr7	-
uc010rib.1	LOC646813	UCSC_knowngene	709	chr11	+
ENST00000517363	RP11-150O12.1	GENCODE	199	chr8	-
ENST00000520306	RP11-37B2.1	GENCODE	421	chr8	-
ENST00000446789	MCF2L-AS1	GENCODE	687	chr13	-
ENST00000446789	MCF2L-AS1	GENCODE	687	chr13	-
ENST00000446789	MCF2L-AS1	GENCODE	687	chr13	-
ENST00000446789	MCF2L-AS1	GENCODE	687	chr13	-
ENST00000419950	TMEM191A	GENCODE	663	chr22	+
NR_003606	ZNFX1-AS1	RefSeq	872	chr20	+
ENST00000438109	CYP4F30P	GENCODE	2250	chr2	+
TCONS_00018263	XLOC_008543	LincRNAs identified	301	chr10	+
uc021vcr.1	BC011243	UCSC_knowngene	964	chr19	+
ENST00000436123	AC098971.2	GENCODE	2964	chr3	-
ENST00000583416	RP11-583F2.2	GENCODE	2474	chr17	+
AK022421		NRED	2134	chr7	-
ENST00000531363	RP11-1L12.3	GENCODE	745	chr11	-
TCONS_00026882	XLOC_012945	LincRNAs identified	414	chr19	+
ENST00000427306	EPB41L4A-AS1	GENCODE	1367	chr5	+
ENST00000455866	AC092171.2	GENCODE	753	chr7	+
HMlincRNA1579-	HMlincRNA1579	LincRNAs identified	2404	chr3	-
ENST00000576963	RP11-353N14.2	GENCODE	1788	chr17	+
ENST00000426828	CCT6P3	GENCODE	2238	chr7	+
uc003qvx.1	AK127120	UCSC_knowngene	4518	chr6	-
ENST00000555332	CTD-2536I1.1	GENCODE	1147	chr15	-
ENST00000428818	TTC28-AS1	GENCODE	706	chr22	+
ENST00000560221	RP11-35O15.1	GENCODE	334	chr15	-
NR_046290	LOC100289092	RefSeq	535	chr16	-
NR_046290	LOC100289092	RefSeq	535	chr16	-
ENST00000544089	RP11-71J4.2	GENCODE	476	chr12	+
ENST00000452050	AC002454.1	GENCODE	407	chr7	+
ENST00000477099	RP11-418B12.1	GENCODE	749	chr3	+
TCONS_00001416	XLOC_000734	LincRNAs identified	1381	chr1	-
uc001okf.1	AK057681	UCSC_knowngene	1429	chr11	+
ENST00000520357	RP11-110G21.1	GENCODE	575	chr8	+
ENST00000567508	RP1-39G22.7	GENCODE	1541	chr1	-
TCONS_00000997	XLOC_000263	LincRNAs identified	311	chr1	+
ENST00000566366	RP1-118J21.25	GENCODE	1196	chr1	+
TCONS_00005961	XLOC_002592	LincRNAs identified	205	chr3	+
ENST00000412266	AC006028.9	GENCODE	3288	chr7	-
ENST00000419425	AC006369.2	GENCODE	548	chr2	+
ENST00000419425	AC006369.2	GENCODE	548	chr2	+
ENST00000419425	AC006369.2	GENCODE	548	chr2	+
ENST00000419425	AC006369.2	GENCODE	548	chr2	+
NR_049728	PSMG1	RefSeq	1033	chr21	-
NR_049728	PSMG1	RefSeq	1033	chr21	-
NR_049728	PSMG1	RefSeq	1033	chr21	-
ENST00000429621	LINC00114	GENCODE	827	chr21	-
TCONS_00020001	XLOC_009294	LincRNAs identified	227	chr11	+
TCONS_00001318	XLOC_000600	LincRNAs identified	565	chr1	+
ENST00000582727	SNHG15	GENCODE	1039	chr7	-
ENST00000451396	PROX1-AS1	GENCODE	476	chr1	-
ENST00000532104	GUCY1B2	GENCODE	1912	chr13	-
NR_033859	LOC574538	RefSeq	2907	chr12	-
NR_033859	LOC574538	RefSeq	2907	chr12	-
ENST00000497774	C17orf76-AS1	GENCODE	1662	chr17	+
NR_026912	ABHD11	RefSeq	1667	chr7	-
NR_026912	ABHD11	RefSeq	1667	chr7	-
NR_026912	ABHD11	RefSeq	1667	chr7	-

112287106	112287405		ATATAAATGTTATTAGAGTGAATGA	intergenic
15932494	15940087		AATACTAAATAACAGGCTCTGGCAT	intergenic
114341204	114341711	NR_003659, uc010b	GAGCGGCGGCGTTAGGACCCGGAG(	intergenic
156396311	156398461	ENST00000418309	CTTTTCAGGGGCGATTTTATGGAGC	intergenic
50375273	50379802	uc001nhe.2	CCACATTTTGGTA 646813	intergenic
37333471	37411585		TTCCTGCTCTAGTAGCCAGTCCTGC(	intergenic
90629157	90633104		TTATAAACCTTAGAATTAAGAAGTT	intergenic
113621822	113623138		CCGCGCCCAGCATTCCAGCGTCCAA	intronic antisense
113621822	113623138		CCGCGCCCAGCATTCCAGCGTCCAA	intronic antisense
113621822	113623138		CCGCGCCCAGCATTCCAGCGTCCAA	natural antisense
113621822	113623138		CCGCGCCCAGCATTCCAGCGTCCAA	natural antisense
21057339	21058894		ATTCCAGGAGCA(84222	intergenic
47894714	47905795		CTCCTGACGAAA 441951	natural antisense
131437622	131443436	uc002trt.3	ATGAAAATTCTG 100132708	intergenic
89190751	89209410	ENST00000413722,	ATTTGATAGTACATTTTGATAGTACA	intergenic
58379176	58380140		TTGAACACACATGCTACCAAGCTCT	intergenic
24137759	24144714		CCATGTTTTAATCACCTTTGGGTCTC	intergenic
62939379	62947172		CATGGAGAGAAAGCTGTGATTCTTT	intergenic
98765273	98767719		GGAAGAAAAAGCAGGATAAGAAAA	intergenic
27238698	27241649		ACAACTTGACATCTGCAGAAATGGG	intergenic
8008905	8014249		TGAAGACGCTCAGGCCTTTGTCATT	intergenic
111496225	111497999		CCAGGGAAATAA 114915	intergenic
5459476	5462753		CCCCTCTTAAATGGAGTTAAAATGG	intronic antisense
33273197	33275601		GGCATTCTAAACACGGAAAACAGCT	intergenic
77797250	77801503		CCTTATCCAAACAGTTCACACGAAG	intergenic
64498737	64535091	ENST00000419314	TTGCCTCCAGCA 643180	intergenic
168067523	168079903		CTATGGTTTGTGCTGTGCTGTGCACA	intergenic
95819689	95832714		ACTCCTGCAATGAATTATATGCATA	intergenic
28315444	28320951	NR_026962	CATCTGTGGATT(284900	intronic antisense
99190179	99190600		CGCCCTTAATGTGGTCCGGTTTCAT(	intergenic
28890277	28891242	NR_046287, NR_04	GAATCAGGCCAC 100289092	natural antisense
28890277	28891242	NR_046287, NR_04	GAATCAGGCCAC 100289092	natural antisense
68738443	68743739		GGTCTAACACCTGTGTGTTCTCTCT(	intergenic
92465802	92546437		AGTCGGCTCTCCGCACGTGTCCGCG	natural antisense
163192854	163197153		TTTTGGATCCACTATAAGGATCCAA	intergenic
23608314	23610576		TGATTTTATGATTTCGTGACTGATG(	intergenic
67064098	67065527		TCTCTTTTGATTTATTATTTGAGACG	intron sense-overlapp
52812274	52825698		GGCGAGCCGGGAAAATATTGCTACT	bidirectional
40722098	40723639		CTTGAATTTCTCAACCCTGCCTAAAT	bidirectional
77685226	77686671		GGTAGACGCAGGATATTGACCTTTT	intergenic
40254647	40255843		CTCGAAGCAAGTGTATAATGCCCCC	bidirectional
18626906	18628395		ACTGGCAAAGAAACTCCTGAGGTGA	intergenic
2757466	2762622		CAGTCTCGAATCTCAAAAAATTACC	intergenic
37827278	37873841		GTTAACAGTGATTTCTGGCTCAGTG	natural antisense
37827278	37873841		GTTAACAGTGATTTCTGGCTCAGTG	natural antisense
37827278	37873841		GTTAACAGTGATTTCTGGCTCAGTG	natural antisense
37827278	37873841		GTTAACAGTGATTTCTGGCTCAGTG	natural antisense
40547371	40555440		CGCGGAAGAGCA 8624	exon sense-overlappi
40547371	40555440		CGCGGAAGAGCA 8624	exon sense-overlappi
40547371	40555440		CGCGGAAGAGCA 8624	exon sense-overlappi
40110944	40119384	ENST00000411989,	GCTGTTGATGGT(400866	intergenic
118662486	118663730		GTGCGCGGGGGGCGCTGCGCGGTG(	intergenic
228688982	228697976		TAAAAACTGTTGATATTAGGATGTA	intergenic
45023213	45026273		TACTATTTTGGGA 285958	intergenic
214139236	214159496		CAGTTTGTCCATT 100505832	intronic antisense
51590243	51654998	ENST00000389600	GAGTACTTGGCT 2974	intergenic
246576	258332		GGGACACCAGTT 574538	natural antisense
246576	258332		GGGACACCAGTT 574538	natural antisense
16342324	16345309	ENST00000480811	ACTTATTCGACTC 125144	intergenic
73150426	73153190		ATACCCTGAGGG 83451	exon sense-overlappi
73150426	73153190		ATACCCTGAGGG 83451	exon sense-overlappi
73150426	73153190		ATACCCTGAGGG 83451	exon sense-overlappi

ENST00000375608	MCF2L	MCF.2 cell line deriv	+	113548691	113751175
ENST00000442652	MCF2L	MCF.2 cell line deriv	+	113556506	113750999
ENST00000375604	MCF2L	MCF.2 cell line deriv	+	113622756	113752862
ENST00000397030	MCF2L	MCF.2 cell line deriv	+	113622813	113754053
NM_021035	ZNFX1	NFX1-type zinc fing	-	47862438	47894756
NM_001080495	TNRC18	trinucleotide repeat-c	-	5346422	5463177
ENST00000415296	PITPNB	phosphatidylinositol	-	28256183	28316122
NM_004320	ATP2A1	sarcoplasmic/endopla	+	28889808	28915830
NM_173201	ATP2A1	sarcoplasmic/endopla	+	28889808	28915830
NM_001145306	CDK6	cyclin-dependent kin	-	92234234	92465941
NM_207354	ANKRD13D	ankyrin repeat domai	+	67056761	67069955
NM_052937	PCMTD1	protein-L-isoaspartat	-	52730139	52811735
NM_005857	ZMPSTE24	CAAX prenyl protea	+	40723721	40759856
NM_001720	BMP8B	bone morphogenetic	-	40223902	40254533
NM_001270436	CDC42EP3	cdc42 effector protei	-	37869024	37899678
NM_001270437	CDC42EP3	cdc42 effector protei	-	37869024	37899342
NM_001270438	CDC42EP3	cdc42 effector protei	-	37869024	37899342
NM_006449	CDC42EP3	cdc42 effector protei	-	37869024	37899342
NM_001261824	PSMG1	proteasome assembly	-	40547371	40555440
NM_003720	PSMG1	proteasome assembly	-	40547371	40555440
NM_203433	PSMG1	proteasome assembly	-	40547371	40555440
ENST00000471129	PROX1	prospero homeobox 1	+	214156523	214170568
NM_001170738	IQSEC3	IQ motif and SEC7 d	+	176048	287625
NM_015232	IQSEC3	IQ motif and SEC7 d	+	186541	280494
NM_001145364	ABHD11	abhydrolase domain-	-	73150424	73153184
NM_148912	ABHD11	abhydrolase domain-	-	73150426	73153184
NM_148913	ABHD11	abhydrolase domain-	-	73150426	73153184

1329.955418	361.53067	9.930067	8.707395	1601.4019	1739.5458
445.276447	111.283554	8.203889	6.898316	528.7224	695.40814
3034.432933	762.625168	11.141429	9.878636	3281.7903	3506.8638
210.964235	52.557859	7.327287	5.810681	225.10786	271.2139
20.162021	7.344223	4.002053	2.783199	27.12701	22.817219
46.299478	14.050899	5.161185	3.990565	34.58941	44.485054
150.338079	32.112756	6.525848	5.172478	81.49933	75.33894
980.38266	216.146199	9.38881	7.850882	726.4145	554.242
980.38266	216.146199	9.38881	7.850882	726.4145	554.242
980.38266	216.146199	9.38881	7.850882	726.4145	554.242
980.38266	216.146199	9.38881	7.850882	726.4145	554.242
235.205378	47.109923	7.258463	5.781876	161.26784	142.00903
21978.81067	4819.445	13.947762	12.632353	24894.436	35036.36
30.722069	7.852914	4.282838	2.966523	45.953136	29.143328
1167.448547	293.516787	9.778483	8.514452	1015.00714	1029.9496
3941.94275	1144.142883	11.483934	10.474388	4771.251	5491.596
25213.91433	5595.1612	14.144924	12.863966	28737.672	32746.303
30.573913	7.482187	4.444376	2.997504	16.13018	13.318779
23.662342	7.949929	4.138592	3.064023	20.04726	10.484849
1441.923788	58.422798	8.722754	5.678229	2055.73	98.3946
59.377213	14.318955	5.538855	4.1296	62.749947	61.487556
279.61623	73.163753	7.670967	6.512453	357.37045	482.36276
89.709799	11.159549	6.100347	3.633828	90.06372	64.777664
16.11718	5.681187	3.67887	2.588635	20.079998	19.046434
529.721438	43.791991	8.598883	5.664763	544.4301	354.9969
1192.409867	268.899213	9.792261	8.422734	1087.6128	818.0177
331.5599	59.376694	7.971684	6.22437	327.76126	219.40788
623.375908	115.068995	8.6893	6.562697	768.9481	858.0023
974.152182	262.687451	9.387463	8.323971	995.14514	524.23505
288.618951	34.384891	7.481459	5.419924	408.60266	689.40356
837.701293	128.747445	9.197579	7.3206	1148.493	1110.7358
837.701293	128.747445	9.197579	7.3206	1148.493	1110.7358
141.961502	45.544503	6.771616	5.592882	207.1011	149.92656
1518.026322	246.281562	9.693945	6.942629	1687.0659	2443.2346
28.974866	8.585585	4.500863	3.181954	30.80489	37.24317
74.216363	19.38507	5.720068	4.340547	53.632484	36.376713
25.454263	6.11921	4.310384	2.652602	26.705494	19.898668
791.736525	229.368692	9.185055	8.073447	828.3746	609.34796
753.960578	229.812658	9.127368	8.008725	917.1815	641.0678
42.652494	11.114258	4.995213	3.641068	56.88232	70.10694
1241.223967	295.638065	9.867602	8.235515	1186.909	1195.8832
36.115291	8.803379	4.799251	3.175475	46.115757	47.547455
917.664225	220.010382	9.407647	8.140545	969.8376	646.2019
49.257797	13.847146	5.207556	3.987537	68.59293	63.981853
49.257797	13.847146	5.207556	3.987537	68.59293	63.981853
49.257797	13.847146	5.207556	3.987537	68.59293	63.981853
49.257797	13.847146	5.207556	3.987537	68.59293	63.981853
5993.09745	1019.052458	12.110625	10.396756	6156.4175	6072.67
5993.09745	1019.052458	12.110625	10.396756	6156.4175	6072.67
5993.09745	1019.052458	12.110625	10.396756	6156.4175	6072.67
137.26089	39.293083	6.630357	5.563647	103.09748	109.4076
92.971775	29.140447	6.188008	5.15138	106.3231	89.47074
197.713305	61.279523	7.255472	6.154867	240.26672	244.40237
1279.825283	180.467212	9.87677	7.797223	1423.542	1816.065
109.87296	13.24967	6.388508	3.953041	83.80144	74.68716
33.058856	6.693243	4.428912	2.745342	30.45847	4.9999995
21.507207	7.590157	4.028227	3.003134	20.14874	10.251008
21.507207	7.590157	4.028227	3.003134	20.14874	10.251008
625.682182	142.118657	8.838611	7.413922	727.59094	978.3546
71.068103	19.993156	5.778909	4.51071	78.51296	93.258896
71.068103	19.993156	5.778909	4.51071	78.51296	93.258896
71.068103	19.993156	5.778909	4.51071	78.51296	93.258896

860.28314	960.88837	1454.5087	1363.1046	298.9457	142.5112
242.0109	124.92671	574.2852	506.30533	145.47972	64.68541
3332.7305	2656.5542	2487.5288	2941.13	910.13995	494.28055
203.49438	239.87253	117.94589	208.15085	72.19224	45.510113
18.98744	18.62608	15.73826	17.676119	7.555797	4.9999995
29.770924	59.187218	50.13387	59.630394	10.593299	6.12574
58.447044	319.24402	74.25408	293.24506	22.774574	28.143404
923.89886	1983.2797	522.1938	1172.2671	100.30231	78.387825
923.89886	1983.2797	522.1938	1172.2671	100.30231	78.387825
923.89886	1983.2797	522.1938	1172.2671	100.30231	78.387825
923.89886	1983.2797	522.1938	1172.2671	100.30231	78.387825
160.28375	635.19434	124.388	188.08931	45.088425	19.762156
12034.852	23803.846	14313.35	21790.02	4699.3984	5795.8896
68.55266	7.747082	15.904981	17.03123	14.31066	4.9999995
988.17694	1248.392	1189.2919	1533.8737	243.25824	173.1483
3059.1328	3097.67	3389.642	3842.3647	833.663	576.0943
16092.811	15643.95	27362.781	30699.969	5323.0996	4700.526
22.491407	59.272198	28.125792	44.105125	6.6169887	4.9999995
29.344158	35.557533	12.574901	33.96535	4.9999995	4.9999995
4170.3184	26.617426	1273.8289	1026.6534	71.28038	12.62886
46.47746	71.54467	44.718975	69.28467	12.359639	12.707201
221.58408	191.10924	192.03197	233.23888	69.4225	61.988735
86.01168	128.652	57.08171	111.67202	7.673823	6.629148
12.428449	14.104529	14.770011	16.27366	5.402384	4.9999995
689.00903	706.9403	295.1037	587.8486	35.73966	29.534042
1330.5804	1656.8707	902.734	1358.6436	284.7434	193.36621
356.34854	265.10266	391.6928	429.04626	57.120777	50.121014
288.39658	192.01547	929.2214	703.6716	98.022255	24.82633
1434.5348	1462.2296	396.2094	1032.5591	267.88815	124.923325
115.75953	214.97292	103.453674	199.52136	30.87958	36.84887
1186.625	387.2021	573.2229	619.92896	139.16519	121.398186
1186.625	387.2021	573.2229	619.92896	139.16519	121.398186
126.50126	138.12648	115.64002	114.473595	56.671936	28.312984
668.9699	152.15533	2405.992	1750.7402	182.31816	36.129242
30.780901	34.233814	15.80272	24.9837	7.514625	4.9999995
76.673386	150.59225	39.810966	88.21238	22.83889	6.9715424
26.665487	16.746517	30.545652	32.16376	7.96792	4.9999995
1093.543	434.60654	889.33325	895.2138	192.3265	99.65457
1019.16284	789.95496	463.13577	693.2606	294.59848	143.03802
28.837305	38.574093	25.579136	35.93517	11.39972	5.1541333
1188.0292	1488.1279	947.6417	1440.7528	310.01056	65.96216
28.228142	25.155676	40.090584	29.554129	8.284737	4.9999995
1171.2856	1096.3833	606.7161	1015.56085	182.85864	185.7518
41.761093	27.555107	55.665047	37.99075	14.935382	17.42183
41.761093	27.555107	55.665047	37.99075	14.935382	17.42183
41.761093	27.555107	55.665047	37.99075	14.935382	17.42183
41.761093	27.555107	55.665047	37.99075	14.935382	17.42183
5646.8555	6293.6714	4790.1743	6998.796	937.3036	912.07025
5646.8555	6293.6714	4790.1743	6998.796	937.3036	912.07025
5646.8555	6293.6714	4790.1743	6998.796	937.3036	912.07025
71.2654	249.66644	102.40021	187.72821	31.033947	21.285696
94.98199	106.694176	77.804245	82.5564	27.538502	24.027985
173.59216	176.80869	171.27495	179.93494	49.396	25.725016
1024.9548	1324.8738	837.6441	1251.872	145.09618	179.82257
95.32167	109.85318	141.81279	153.76152	14.05433	6.6749835
57.20181	25.942774	37.45171	42.29837	4.9999995	4.9999995
24.371359	32.205303	12.287771	29.779062	8.1803055	6.5249386
24.371359	32.205303	12.287771	29.779062	8.1803055	6.5249386
387.5921	579.9324	465.8288	614.79425	126.26629	96.31196
63.81292	77.5813	42.90803	70.33451	23.931679	8.132209
63.81292	77.5813	42.90803	70.33451	23.931679	8.132209
63.81292	77.5813	42.90803	70.33451	23.931679	8.132209

718.7078	288.75046	436.57776	283.6911	10.178893	10.362165
254.86128	61.09716	83.90404	57.673717	8.560154	9.117746
1318.3896	566.8731	740.92175	545.14606	11.2037945	11.353091
111.12081	27.665346	33.99452	24.864128	7.365525	7.816457
16.50954	4.9999995	4.9999995	4.9999995	4.308839	4.471334
12.705611	25.718529	14.288867	14.873351	4.65056	5.3575344
12.46276	61.49335	22.068546	45.7339	5.897513	6.0480924
117.99268	254.6518	440.675	304.86758	9.037142	8.805644
117.99268	254.6518	440.675	304.86758	9.037142	8.805644
117.99268	254.6518	440.675	304.86758	9.037142	8.805644
117.99268	254.6518	440.675	304.86758	9.037142	8.805644
67.48276	48.297283	64.29517	37.733746	6.8753314	6.9186983
3572.3367	6064.687	4240.6533	4543.705	14.17012	14.7291355
11.27942	4.9999995	6.5274043	4.9999995	5.0617476	4.8021708
359.85098	267.55756	429.96417	287.32147	9.515246	9.663292
1687.0348	981.1602	1582.2458	1204.6592	11.740653	11.97067
5780.2866	6994.8413	5607.015	5165.1987	14.370472	14.579334
5.556342	15.190182	5.5099425	7.0196667	3.5401654	3.7736626
5.586136	12.18911	8.21045	11.71388	3.8650498	3.4615943
133.48238	14.067228	80.59243	38.48551	10.52497	6.4059496
12.432272	17.357979	12.96898	18.087658	5.51504	5.779569
82.52173	61.75922	98.94163	64.3487	8.022528	8.6043415
21.17967	7.565054	12.27018	11.639421	6.039561	5.845935
8.4478445	5.2368975	4.9999995	4.9999995	3.8672132	4.2381277
22.219873	87.00156	36.765594	51.49122	8.603849	8.186546
336.0805	249.46971	320.51062	229.22484	9.613865	9.346241
60.08896	78.73616	57.862206	52.331047	7.899537	7.521781
390.3477	45.252575	76.64488	55.320232	9.127946	9.406958
423.58148	251.37372	291.42892	216.92911	9.488339	8.723316
27.72032	43.53675	37.64749	29.676336	8.21464	9.104666
155.81142	93.258125	160.52736	102.32439	9.694508	9.767113
155.81142	93.258125	160.52736	102.32439	9.694508	9.767113
103.2248	33.06769	17.70512	34.284485	7.240632	6.993594
1083.148	33.012825	106.20969	36.871456	10.2493	10.831475
15.676701	8.288789	7.342129	7.6912684	4.489123	5.124469
35.726383	8.622001	24.29338	17.858221	5.277318	5.092699
4.9999995	8.747341	4.9999995	4.9999995	4.2824736	4.2934694
318.7354	159.70248	380.38983	225.40337	9.232642	8.935298
437.1325	96.48863	270.2748	137.34352	9.371874	9.004969
17.240002	7.270744	14.63407	10.986879	5.3685813	5.9547
553.5972	119.1494	467.89557	257.2135	9.73489	9.866453
17.814095	5.3849607	9.021603	7.3148794	5.066824	5.443587
199.49454	294.4221	243.41315	214.12206	9.450233	9.014611
20.866032	6.106446	10.7211485	13.032039	5.638851	5.8282285
20.866032	6.106446	10.7211485	13.032039	5.638851	5.8282285
20.866032	6.106446	10.7211485	13.032039	5.638851	5.8282285
20.866032	6.106446	10.7211485	13.032039	5.638851	5.8282285
958.8808	1026.9312	1323.4216	955.7073	12.096656	12.106145
958.8808	1026.9312	1323.4216	955.7073	12.096656	12.106145
958.8808	1026.9312	1323.4216	955.7073	12.096656	12.106145
55.392868	48.677788	42.462776	36.905422	6.2408614	6.5534205
41.70348	31.947245	25.796171	23.829298	6.2862306	6.2790275
107.430695	61.65771	66.978546	56.48917	7.456524	7.666481
93.570335	270.95245	204.31194	189.0498	10.003053	10.423735
18.199287	14.933701	14.49179	11.143931	5.9369946	6.036209
4.9999995	8.434877	11.72458	4.9999995	4.472847	2.3499577
15.167062	5.6686344	4.9999995	4.9999995	3.8721473	3.431062
15.167062	5.6686344	4.9999995	4.9999995	3.8721473	3.431062
152.541	86.92263	250.83525	139.83481	9.040924	9.589903
26.258944	16.15326	27.051327	18.431519	5.8414736	6.3328805
26.258944	16.15326	27.051327	18.431519	5.8414736	6.3328805
26.258944	16.15326	27.051327	18.431519	5.8414736	6.3328805

9.395338	9.589903	10.226981	9.827123	8.489643	8.005969
7.591375	6.6335535	8.909478	8.41103	7.416786	6.870746
11.329662	11.043038	11.007272	10.911717	10.1455	9.811741
7.3562946	7.5992785	6.660993	7.165174	6.404771	6.355981
3.92721	3.684724	3.8993936	3.720819	3.1130807	2.4279919
4.5871224	5.506925	5.4714303	5.3935366	3.6324975	3.3657851
5.5545783	7.996295	6.010092	7.648516	4.7492137	5.6513157
9.48543	10.625169	8.773653	9.6058235	6.884366	7.1422005
9.48543	10.625169	8.773653	9.6058235	6.884366	7.1422005
9.48543	10.625169	8.773653	9.6058235	6.884366	7.1422005
9.48543	10.625169	8.773653	9.6058235	6.884366	7.1422005
6.990489	8.998642	6.7375274	7.0300875	5.7245784	5.1335692
13.236744	14.227712	13.510657	13.812202	12.509256	13.388846
5.7898073	2.459827	3.9156823	3.6677918	4.0676994	2.4279919
9.577352	9.968546	9.953018	9.993444	8.184559	8.279015
11.193256	11.264572	11.431436	11.303015	10.010733	10.028292
13.688972	13.588936	14.333352	14.308476	12.688867	13.098996
4.17781	5.508582	4.6849294	4.9811063	2.9221995	2.4279919
4.5678964	4.717102	3.592714	4.6271977	2.3467326	2.4279919
11.676552	4.269783	10.040541	9.418731	6.3876615	4.4573
5.2184324	5.7963104	5.321207	5.6025724	3.8564725	4.4648232
7.469465	7.2572775	7.3502674	7.3219213	6.350757	6.8075266
6.1110935	6.6782455	5.6478267	6.2794204	3.134729	3.4860427
3.3072598	3.2423792	3.8142054	3.6040325	2.6363752	2.4279919
9.067061	9.157744	7.9637036	8.614396	5.3947167	5.7241726
10.0166855	10.388451	9.5666065	9.821717	8.415353	8.455578
8.139521	7.735101	8.360454	8.173707	6.0756764	6.495697
7.840444	7.264751	9.612837	8.882866	6.84785	5.464513
10.110342	10.198489	8.376562	9.427729	8.325664	7.811635
6.5360494	7.43797	6.48076	7.114668	5.183704	6.050988
9.842656	8.276898	8.905201	8.699101	7.349468	7.7707605
9.842656	8.276898	8.905201	8.699101	7.349468	7.7707605
6.659038	6.7841897	6.6358724	6.3163724	6.06201	5.6606035
9.020351	6.9225497	10.957084	10.182909	7.7611227	6.0224094
4.6373506	4.658831	3.9052913	4.190112	3.1034155	2.4279919
5.9457617	6.906183	5.15489	5.9435587	4.7536535	3.5651326
4.425802	3.5157945	4.7915974	4.553167	3.1947105	2.4279919
9.720829	8.454127	9.544226	9.223209	7.8323364	7.4943223
9.619337	9.311349	8.596036	8.860646	8.466291	8.009429
4.541626	4.843514	4.5610785	4.70178	3.7419677	3.0947804
9.845725	10.220142	9.638157	9.900243	8.541823	6.900912
4.5098515	4.176882	5.1659017	4.4324594	3.2564945	2.4279919
9.827123	9.774209	8.9801445	9.399561	7.763898	8.388277
5.0643563	4.3214145	5.6138725	4.7786136	4.1312904	4.9431906
5.0643563	4.3214145	5.6138725	4.7786136	4.1312904	4.9431906
5.0643563	4.3214145	5.6138725	4.7786136	4.1312904	4.9431906
5.0643563	4.3214145	5.6138725	4.7786136	4.1312904	4.9431906
12.105225	12.27947	11.920088	12.156163	10.187893	10.710006
12.105225	12.27947	11.920088	12.156163	10.187893	10.710006
12.105225	12.27947	11.920088	12.156163	10.187893	10.710006
5.841633	7.6550393	6.4639854	7.0272036	5.1889553	5.244028
6.2487144	6.388566	6.0768285	5.8486814	5.0198116	5.419451
7.1099005	7.14412	7.1909933	6.964814	5.8589325	5.5199423
9.626983	10.047306	9.447347	9.712195	7.4115043	8.338058
6.2521772	6.4339237	6.9296117	6.7421327	4.040629	3.4989576
5.523938	4.2283254	5.075079	4.9233227	2.3467326	2.4279919
4.2969522	4.5650153	3.5596676	4.4445186	3.2352588	3.463159
4.2969522	4.5650153	3.5596676	4.4445186	3.2352588	3.463159
8.249008	8.860327	8.60511	8.686392	7.2126503	7.44591
5.6912537	5.9152474	5.265888	5.626709	4.8239346	3.7896497
5.6912537	5.9152474	5.265888	5.626709	4.8239346	3.7896497
5.6912537	5.9152474	5.265888	5.626709	4.8239346	3.7896497

9.430864	8.619258	9.007913	8.6907215
7.9416356	6.226834	6.644643	6.2892513
10.300857	9.609529	9.764	9.640189
6.7576475	4.992227	5.361358	4.992101
4.1026797	2.3408759	2.3618152	2.352752
3.7482593	4.87635	4.107762	4.2127376
3.722822	6.236862	4.737559	5.9370975
6.8434854	8.422825	9.024214	8.788202
6.8434854	8.422825	9.024214	8.788202
6.8434854	8.422825	9.024214	8.788202
6.8434854	8.422825	9.024214	8.788202
6.056382	5.858295	6.273612	5.6448193
11.782421	13.044908	12.318564	12.75012
3.59337	2.3408759	3.016451	2.352752
8.435932	8.498161	8.984613	8.704435
10.659363	10.436133	10.882229	10.829578
12.468296	13.256198	12.746854	12.9245825
2.6975646	4.0644636	2.7988324	3.0739717
2.7043612	3.7232053	3.3326278	3.849219
7.0156975	3.9421318	6.589743	5.676843
3.7201068	4.2608533	3.9732928	4.5020523
6.333911	6.2466826	6.8842144	6.4516253
4.44253	3.007125	3.8938975	3.8386447
3.2080941	2.5447836	2.3618152	2.352752
4.5074472	6.77469	5.473324	6.114225
8.338058	8.388046	8.564828	8.374539
5.8950615	6.6237035	6.118871	6.1372123
8.560154	5.7524366	6.5235806	6.227646
8.678753	8.401184	8.435512	8.291078
4.812683	5.6895695	5.5040965	5.2785006
7.2330317	6.8736258	7.5562115	7.1405044
7.2330317	6.8736258	7.5562115	7.1405044
6.652729	5.2621775	4.4195194	5.5002513
10.015636	5.259212	6.98722	5.610174
4.034582	3.139344	3.1742296	3.212164
5.1628685	3.203964	4.873744	4.4839215
2.350709	3.2276337	2.3618152	2.352752
8.266052	7.6891384	8.809521	8.349313
8.728037	6.925351	8.328635	7.5946083
4.1603127	2.954668	4.1440926	3.7505887
9.061951	7.2531204	9.1084795	8.546806
4.2072234	2.5660427	3.458692	3.1364045
7.5899186	8.648746	8.181606	8.270824
4.4234266	2.7149887	3.7008774	4.0114455
4.4234266	2.7149887	3.7008774	4.0114455
4.4234266	2.7149887	3.7008774	4.0114455
4.4234266	2.7149887	3.7008774	4.0114455
9.84461	10.506499	10.631641	10.499889
9.84461	10.506499	10.631641	10.499889
9.84461	10.506499	10.631641	10.499889
5.78483	5.8707657	5.681609	5.6116915
5.3785915	5.2083826	4.958237	4.923807
6.71343	6.2416773	6.334403	6.260819
6.5130377	8.519536	7.9261293	8.075073
4.2407823	4.0373206	4.128972	3.771582
2.350709	3.1671808	3.8266878	2.352752
3.9877596	2.6180596	2.3618152	2.352752
3.9877596	2.6180596	2.3618152	2.352752
7.201956	6.773463	8.230258	7.619292
4.7376976	4.1562896	5.028653	4.528037
4.7376976	4.1562896	5.028653	4.528037
4.7376976	4.1562896	5.028653	4.528037

ASHGA5P035316	0.003732342	0.03919943	9.0096127	up	noncoding
ASHGA5P029195	0.014985811	0.074989017	2.4653166	up	noncoding
ASHGA5P029195	0.014985811	0.074989017	2.4653166	up	noncoding
ASHGA5P042177	0.025236058	0.098803647	2.0409332	up	noncoding
ASHGA5P042177	0.025236058	0.098803647	2.0409332	up	noncoding
ASHGA5P044133	8.93149E-06	0.004010044	2.5035938	up	noncoding
ASHGA5P033860	0.001552334	0.026472272	2.1227728	up	noncoding
ASHGA5P054913	0.000526842	0.015826428	2.0673705	up	noncoding
ASHGA5P054913	0.000526842	0.015826428	2.0673705	up	noncoding
ASHGA5P054913	0.000526842	0.015826428	2.0673705	up	noncoding
ASHGA5P057291	0.000148634	0.01076444	4.5051681	up	noncoding
ASHGA5P036826	0.016266019	0.07799128	2.6639382	up	noncoding
ASHGA5P036826	0.016266019	0.07799128	2.6639382	up	noncoding
ASHGA5P036826	0.016266019	0.07799128	2.6639382	up	noncoding
ASHGA5P052416	0.000567686	0.01610992	3.9022052	up	noncoding
ASHGA5P052416	0.000567686	0.01610992	3.9022052	up	noncoding
ASHGA5P015833	0.000218654	0.01146874	5.3549971	up	noncoding
ASHGA5P015833	0.000218654	0.01146874	5.3549971	up	noncoding
ASHGA5P015833	0.000218654	0.01146874	5.3549971	up	noncoding
ASHGA5P027679	0.042085485	0.131395683	16.5454554	up	noncoding
ASHGA5P037417	0.012648784	0.06880509	2.2802399	up	noncoding
ASHGA5P037417	0.012648784	0.06880509	2.2802399	up	noncoding
ASHGA5P037417	0.012648784	0.06880509	2.2802399	up	noncoding
ASHGA5P037417	0.012648784	0.06880509	2.2802399	up	noncoding
ASHGA5P037417	0.012648784	0.06880509	2.2802399	up	noncoding
ASHGA5P037417	0.012648784	0.06880509	2.2802399	up	noncoding
ASHGA5P037417	0.012648784	0.06880509	2.2802399	up	noncoding
ASHGA5P037417	0.012648784	0.06880509	2.2802399	up	noncoding
ASHGA5P037417	0.012648784	0.06880509	2.2802399	up	noncoding
ASHGA5P037417	0.012648784	0.06880509	2.2802399	up	noncoding
ASHGA5P036782	0.008056913	0.056483235	2.0959492	up	noncoding
ASHGA5P036782	0.008056913	0.056483235	2.0959492	up	noncoding
ASHGA5P043018	0.000467732	0.015177128	6.6138405	up	noncoding
ASHGA5P043018	0.000467732	0.015177128	6.6138405	up	noncoding
ASHGA5P043018	0.000467732	0.015177128	6.6138405	up	noncoding
ASHGA5P043018	0.000467732	0.015177128	6.6138405	up	noncoding
ASHGA5P025851	0.002013153	0.029606317	3.5687454	up	noncoding
ASHGA5P025851	0.002013153	0.029606317	3.5687454	up	noncoding
ASHGA5P044029	0.040249244	0.128419201	2.0190485	up	noncoding
ASHGA5P044203	1.06596E-05	0.004010044	3.3489264	up	noncoding
ASHGA5P021040	0.005082291	0.045256951	2.1552209	up	noncoding
ASHGA5P031377	0.005100157	0.04534084	3.2388311	up	noncoding
ASHGA5P021045	0.011216107	0.065344868	2.9561737	up	noncoding
ASHGA5P021045	0.011216107	0.065344868	2.9561737	up	noncoding
ASHGA5P021045	0.011216107	0.065344868	2.9561737	up	noncoding
ASHGA5P021045	0.011216107	0.065344868	2.9561737	up	noncoding
ASHGA5P020868	0.004823018	0.044076887	2.9082254	up	noncoding
ASHGA5P032880	0.007606816	0.05476971	2.5417335	up	noncoding
ASHGA5P053402	0.000381961	0.013977512	4.0736694	up	noncoding
ASHGA5P053402	0.000381961	0.013977512	4.0736694	up	noncoding
ASHGA5P053486	0.002242285	0.031214119	3.0913827	up	noncoding
ASHGA5P030942	0.026588029	0.101816856	2.0983836	up	noncoding
ASHGA5P053021	0.014431414	0.073620202	3.4047199	up	noncoding
ASHGA5P053021	0.014431414	0.073620202	3.4047199	up	noncoding
ASHGA5P047680	0.004121103	0.041046245	17.2528194	up	noncoding
ASHGA5P029059	0.006411628	0.050954488	3.4559258	up	noncoding
ASHGA5P020906	0.000736594	0.018239926	2.7974182	up	noncoding
ASHGA5P052828	0.005097905	0.04534084	2.004114	up	noncoding
ASHGA5P020976	0.000437128	0.014669266	2.5590118	up	noncoding
ASHGA5P047949	9.09949E-06	0.004010044	2.5181329	up	noncoding
ASHGA5P032840	0.036317448	0.120888213	4.2484427	up	noncoding
ASHGA5P014151	0.007230429	0.053515377	3.5335285	up	noncoding

ENST00000446213	AC133785.1	GENCODE	1273	chr2	-
ENST00000555850	RP11-862P13.1	GENCODE	514	chr14	-
ENST00000555850	RP11-862P13.1	GENCODE	514	chr14	-
ENST00000416770	RPS6KA2-IT1	GENCODE	579	chr6	-
ENST00000416770	RPS6KA2-IT1	GENCODE	579	chr6	-
uc011kxs.1	DQ587039	UCSC_knowngene	309	chr8	-
ENST00000579506	RP11-202D1.3	GENCODE	384	chr18	+
ENST00000545688	SNHG1	GENCODE	610	chr11	-
ENST00000545688	SNHG1	GENCODE	610	chr11	-
ENST00000545688	SNHG1	GENCODE	610	chr11	-
TCONS_00015426	XLOC_007053	LincRNAs identified	418	chr8	-
uc002xti.1	AK098067	UCSC_knowngene	2884	chr20	-
uc002xti.1	AK098067	UCSC_knowngene	2884	chr20	-
uc002xti.1	AK098067	UCSC_knowngene	2884	chr20	-
NR_003141	SNHG4	RefSeq	1100	chr5	+
NR_003141	SNHG4	RefSeq	1100	chr5	+
NR_024534	ALG3	RefSeq	1513	chr3	-
NR_024534	ALG3	RefSeq	1513	chr3	-
NR_024534	ALG3	RefSeq	1513	chr3	-
AA558434		LincRNAs identified	556	chrX	-
ENST00000456904	AP001442.2	GENCODE	430	chr21	+
ENST00000456904	AP001442.2	GENCODE	430	chr21	+
ENST00000456904	AP001442.2	GENCODE	430	chr21	+
ENST00000456904	AP001442.2	GENCODE	430	chr21	+
ENST00000456904	AP001442.2	GENCODE	430	chr21	+
ENST00000456904	AP001442.2	GENCODE	430	chr21	+
ENST00000456904	AP001442.2	GENCODE	430	chr21	+
ENST00000456904	AP001442.2	GENCODE	430	chr21	+
ENST00000456904	AP001442.2	GENCODE	430	chr21	+
ENST00000456904	AP001442.2	GENCODE	430	chr21	+
ENST00000570096	RP4-616B8.4	GENCODE	187	chr20	-
ENST00000570096	RP4-616B8.4	GENCODE	187	chr20	-
ENST00000415237	AC004988.1	GENCODE	1771	chr7	-
ENST00000415237	AC004988.1	GENCODE	1771	chr7	-
ENST00000415237	AC004988.1	GENCODE	1771	chr7	-
ENST00000415237	AC004988.1	GENCODE	1771	chr7	-
NR_038975	MIR181A2HG	RefSeq	617	chr9	+
NR_038975	MIR181A2HG	RefSeq	617	chr9	+
NR_027387	LOC100128822	RefSeq	1438	chr7	+
ENST00000517714	RP11-150O12.4	GENCODE	551	chr8	-
ENST00000523789	EXTL3-AS1	GENCODE	542	chr8	-
TCONS_00006773	XLOC_003406	LincRNAs identified	4887	chr3	-
ENST00000523905	RP11-347C18.3	GENCODE	709	chr8	+
ENST00000523905	RP11-347C18.3	GENCODE	709	chr8	+
ENST00000523905	RP11-347C18.3	GENCODE	709	chr8	+
ENST00000523905	RP11-347C18.3	GENCODE	709	chr8	+
ENST00000521122	PVT1	GENCODE	221	chr8	+
ENST00000578757	C17orf76-AS1	GENCODE	920	chr17	+
uc001gjk.3	GAS5	UCSC_knowngene	1696	chr1	-
uc001gjk.3	GAS5	UCSC_knowngene	1696	chr1	-
ENST00000524166	RP11-37B2.1	GENCODE	836	chr8	-
ENST00000565648	RP11-473I1.5	GENCODE	731	chr16	-
uc003tab.3	BC038570	UCSC_knowngene	1428	chr7	-
uc003tab.3	BC038570	UCSC_knowngene	1428	chr7	-
NR_038940	LOC100505839	RefSeq	2023	chr10	+
ENST00000548631	RP11-1103G16.1	GENCODE	579	chr14	-
ENST00000521815	RP11-255B23.4	GENCODE	367	chr8	-
ENST00000520891	RP3-399L15.3	GENCODE	603	chr6	+
ENST00000522963	PVT1	GENCODE	568	chr8	+
TCONS_00013467	XLOC_006118	LincRNAs identified	4569	chr7	+
ENST00000577743	RP11-401O9.3	GENCODE	641	chr17	+
NR_027349	MIR17HG	RefSeq	927	chr13	+

131593010	131594567		TCATGATCATCTGCTCTAAACACGT		bidirectional
67435754	67436533		GATACTTCACTGTCATAAAGCAAGA		intronic antisense
67435754	67436533		GATACTTCACTGTCATAAAGCAAGA		intronic antisense
166874150	166878871		ATCATGAGACTG	100874353	intron sense-overlapp
166874150	166878871		ATCATGAGACTG	100874353	intron sense-overlapp
12281921	12282230	ENST00000427587,	TTTCAGCCTTGCTAAACTGTCTGAA		intergenic
51094840	51104092		GCACAGTGATTGACAATCTCCTTTC		intergenic
62620220	62622932		ATAAAAAATGGT	23642	bidirectional
62620220	62622932		ATAAAAAATGGT	23642	bidirectional
62620220	62622932		ATAAAAAATGGT	23642	bidirectional
37264166	37270206		CAATATCATGATTCCTCAGCAGTGC		intergenic
45979227	45982111		GGCAATTCAGACAGGTACAGATCTT		intron sense-overlapp
45979227	45982111		GGCAATTCAGACAGGTACAGATCTT		intron sense-overlapp
45979227	45982111		GGCAATTCAGACAGGTACAGATCTT		intron sense-overlapp
138609440	138615317		GATAAGTAACGT	724102	exon sense-overlappi
138609440	138615317		GATAAGTAACGT	724102	exon sense-overlappi
183960116	183966759	NM_001006941	CCTGGCGGAGAC	10195	exon sense-overlappi
183960116	183966759	NM_001006941	CCTGGCGGAGAC	10195	exon sense-overlappi
183960116	183966759	NM_001006941	CCTGGCGGAGAC	10195	natural antisense
125395367	125395925		AGACTACATGATTATGAGATATCTC		intergenic
27306611	27307584		CACAACTAAATTTACTGAAGTCAGG		intronic antisense
27306611	27307584		CACAACTAAATTTACTGAAGTCAGG		intronic antisense
27306611	27307584		CACAACTAAATTTACTGAAGTCAGG		intronic antisense
27306611	27307584		CACAACTAAATTTACTGAAGTCAGG		intronic antisense
27306611	27307584		CACAACTAAATTTACTGAAGTCAGG		intronic antisense
27306611	27307584		CACAACTAAATTTACTGAAGTCAGG		intronic antisense
27306611	27307584		CACAACTAAATTTACTGAAGTCAGG		intronic antisense
27306611	27307584		CACAACTAAATTTACTGAAGTCAGG		intronic antisense
27306611	27307584		CACAACTAAATTTACTGAAGTCAGG		intronic antisense
27306611	27307584		CACAACTAAATTTACTGAAGTCAGG		intronic antisense
37590567	37590754		GCACTGGGCGTTGAAGCAGTGCTTT		bidirectional
37590567	37590754		GCACTGGGCGTTGAAGCAGTGCTTT		bidirectional
40577725	40586527		CAGGGGATGAATATGTTCATTACCT		intronic antisense
40577725	40586527		CAGGGGATGAATATGTTCATTACCT		intronic antisense
40577725	40586527		CAGGGGATGAATATGTTCATTACCT		intronic antisense
40577725	40586527		CAGGGGATGAATATGTTCATTACCT		intronic antisense
127420714	127460907	ENST00000429139	CTTTTACTGGATC	100379345	intronic antisense
127420714	127460907	ENST00000429139	CTTTTACTGGATC	100379345	intronic antisense
152161208	152162630	ENST00000564834	GGACGTTTATAT	100128822	intergenic
37458054	37483391		AAGAGCCTCTCTTTGGGAAATCTGA		intergenic
28555728	28558047		TTGGGCTAAGGATAACTTCCTCTAC		intronic antisense
197374633	197381275	TCONS_00007073,	TGCTGATTGATTGATGAGACTTCTT		intergenic
95962264	95963624		CAGAACCAAGAATGTGGCAGAAAG		intron sense-overlapp
95962264	95963624		CAGAACCAAGAATGTGGCAGAAAG		intron sense-overlapp
95962264	95963624		CAGAACCAAGAATGTGGCAGAAAG		intron sense-overlapp
95962264	95963624		CAGAACCAAGAATGTGGCAGAAAG		intron sense-overlapp
128866973	128902987		TTCGTATTTTGCC	5820	intergenic
16342351	16344523	ENST00000497774	GTTAAGAAATGT	125144	intergenic
173833038	173837125	ENST00000442067	TTTTAAGTGTTGT	60674	bidirectional
173833038	173837125	ENST00000442067	TTTTAAGTGTTGT	60674	bidirectional
90623584	90629386		CACAGTGCTAGTAAGTAATGAATGT		intergenic
9199690	9201031		TGTCCTCTCTTAAATACATAGGACA		intronic antisense
29553839	29603273		TAATTACCCTAGAACATAAATTTCA		natural antisense
29553839	29603273		TAATTACCCTAGAACATAAATTTCA		natural antisense
105506536	105515167		GGGGATTATGAT	100505839	intronic antisense
30919565	30943276		TGCTGAGATTCCCATTGGTTTCCCA		intergenic
128197879	128215467		CTATCCCAAGATAACACTAACAAAG		intergenic
114292659	114316979	ENST00000521888	TCCCGCCCAAGCCGACCACGTGCCG		intronic antisense
128808058	128903041	uc003ysj.2	TCTCCATTATTTG	5820	intergenic
63170710	63176135		AAATCATAGAGAGTCATTGGCCAAT		intergenic
10223708	10244775		CCTGCTTATCTCTAAAGAGTAAGTA		natural antisense
92000073	92006829		CCTCTAAAAGGC	407975	intergenic

ENST00000409359	ARHGEF4	Rho guanine nucleoti	+	131595027	131704803
NM_001024218	GPHN	gephyrin isoform 2	+	66974124	67648525
NM_020806	GPHN	gephyrin isoform 1	+	66974124	67648525
NM_001006932	RPS6KA2	ribosomal protein S6	-	166822853	167275771
NM_021135	RPS6KA2	ribosomal protein S6	-	166822853	167040726
NM_001012662	SLC3A2	4F2 cell-surface antig	+	62623483	62656355
NM_001012664	SLC3A2	4F2 cell-surface antig	+	62623483	62656355
NM_002394	SLC3A2	4F2 cell-surface antig	+	62623483	62656355
NM_012408	ZMYND8	protein kinase C-binc	-	45838380	45985474
NM_183047	ZMYND8	protein kinase C-binc	-	45838380	45985474
NM_183048	ZMYND8	protein kinase C-binc	-	45838380	45985474
NM_001194954	MATR3	matrin-3 isoform a	+	138609440	138667366
NM_199189	MATR3	matrin-3 isoform a	+	138609440	138667366
NM_001006941	ALG3	dol-P-Man:Man(5)G	-	183960116	183967313
NM_005787	ALG3	dol-P-Man:Man(5)G	-	183960116	183966759
NM_138345	VWA5B2	von Willebrand facto	+	183948316	183960117
NM_000484	APP	amyloid beta A4 prot	-	27252860	27543138
NM_001136016	APP	amyloid beta A4 prot	-	27252860	27512708
NM_001136129	APP	amyloid beta A4 prot	-	27252860	27543138
NM_001136130	APP	amyloid beta A4 prot	-	27252860	27543138
NM_001136131	APP	amyloid beta A4 prot	-	27252860	27543446
NM_001204301	APP	amyloid beta A4 prot	-	27252860	27543138
NM_001204302	APP	amyloid beta A4 prot	-	27252860	27543138
NM_001204303	APP	amyloid beta A4 prot	-	27252860	27543138
NM_201413	APP	amyloid beta A4 prot	-	27252860	27543138
NM_201414	APP	amyloid beta A4 prot	-	27252860	27543138
NM_001190809	DHX35	probable ATP-depen	+	37590980	37668366
NM_021931	DHX35	probable ATP-depen	+	37590980	37668366
NM_001193311	C7orf10	caiB/baiF CoA-trans	+	40174574	40900366
NM_001193312	C7orf10	caiB/baiF CoA-trans	+	40174574	40900366
NM_001193313	C7orf10	caiB/baiF CoA-trans	+	40174574	40900366
NM_024728	C7orf10	caiB/baiF CoA-trans	+	40174574	40900366
NM_001489	NR6A1	nuclear receptor subf	-	127284702	127533576
NM_033334	NR6A1	nuclear receptor subf	-	127284702	127533576
ENST00000523149	EXTL3	exostoses (multiple)-	+	28480245	28608420
ENST00000396111	NDUFAF6	NADH dehydrogena	+	95908043	96070938
ENST00000396113	NDUFAF6	NADH dehydrogena	+	95908040	96070938
ENST00000519136	NDUFAF6	NADH dehydrogena	+	95908064	96059259
ENST00000523378	NDUFAF6	NADH dehydrogena	+	95907994	96059318
NM_001122770	ZBTB37	zinc finger and BTB	+	173837492	173855774
NM_032522	ZBTB37	zinc finger and BTB	+	173837492	173842778
NM_014117	C16orf72	UPF0472 protein C1	+	9185536	9213555
NM_001039936	CHN2	beta-chimaerin isofo	+	29519485	29553944
NM_004067	CHN2	beta-chimaerin isofo	+	29234120	29553944
NM_014631	SH3PXD2A	SH3 and PX domain-	-	105353783	105615164
ENST00000522371	HDAC2	histone deacetylase 2	-	114277792	114332472
NM_003802	MYH13	myosin-13	-	10204182	10276322

93.480313	5	5.534945	2.363479	42.933525	15.904529
22.019373	7.126396	4.134129	2.832356	24.610182	28.143469
22.019373	7.126396	4.134129	2.832356	24.610182	28.143469
21.30333	7.367176	4.020672	2.991443	21.566395	17.27053
21.30333	7.367176	4.020672	2.991443	21.566395	17.27053
8389.057717	1880.605633	12.590497	11.266496	8902.202	9725.854
41.67152	12.197282	4.958388	3.872438	51.139862	21.71797
646.486125	185.219512	8.824397	7.776601	541.1535	430.04773
646.486125	185.219512	8.824397	7.776601	541.1535	430.04773
646.486125	185.219512	8.824397	7.776601	541.1535	430.04773
45.525424	7.598625	5.175184	3.003603	43.49231	37.691826
350.858437	103.69279	7.978467	6.564906	463.03198	306.68716
350.858437	103.69279	7.978467	6.564906	463.03198	306.68716
350.858437	103.69279	7.978467	6.564906	463.03198	306.68716
131.327196	20.178154	6.488457	4.524167	91.306206	135.04918
131.327196	20.178154	6.488457	4.524167	91.306206	135.04918
66.843167	8.558783	5.71047	3.289584	63.038857	76.83882
66.843167	8.558783	5.71047	3.289584	63.038857	76.83882
66.843167	8.558783	5.71047	3.289584	63.038857	76.83882
1022.06504	15.881386	7.437836	3.389473	1261.3989	4.9999995
51.947701	16.207372	5.360857	4.171671	47.420174	47.219654
51.947701	16.207372	5.360857	4.171671	47.420174	47.219654
51.947701	16.207372	5.360857	4.171671	47.420174	47.219654
51.947701	16.207372	5.360857	4.171671	47.420174	47.219654
51.947701	16.207372	5.360857	4.171671	47.420174	47.219654
51.947701	16.207372	5.360857	4.171671	47.420174	47.219654
51.947701	16.207372	5.360857	4.171671	47.420174	47.219654
51.947701	16.207372	5.360857	4.171671	47.420174	47.219654
51.947701	16.207372	5.360857	4.171671	47.420174	47.219654
51.947701	16.207372	5.360857	4.171671	47.420174	47.219654
292.945022	82.769246	7.754662	6.687059	355.44043	245.18834
292.945022	82.769246	7.754662	6.687059	355.44043	245.18834
66.910227	6.678513	5.53327	2.807782	63.42931	13.052479
66.910227	6.678513	5.53327	2.807782	63.42931	13.052479
66.910227	6.678513	5.53327	2.807782	63.42931	13.052479
66.910227	6.678513	5.53327	2.807782	63.42931	13.052479
58.602426	9.120491	5.140452	3.305035	80.20778	132.41603
58.602426	9.120491	5.140452	3.305035	80.20778	132.41603
4299.714117	1120.67505	11.515618	10.501942	3621.2363	2779.3738
22.51272	5.165932	4.179919	2.43622	23.387978	20.08071
148.442563	47.593618	6.856971	5.749135	172.24217	168.19722
6355.994133	920.662175	11.858491	10.163018	3511.2317	3083.6301
48.246573	13.033233	5.210467	3.646736	62.35257	57.944843
48.246573	13.033233	5.210467	3.646736	62.35257	57.944843
48.246573	13.033233	5.210467	3.646736	62.35257	57.944843
48.246573	13.033233	5.210467	3.646736	62.35257	57.944843
84.833912	18.954445	5.97619	4.43605	73.14876	52.3169
1454.025067	324.436357	9.991207	8.645394	1789.2196	2594.845
6564.263	891.099812	12.188371	10.162042	5624.518	4858.849
6564.263	891.099812	12.188371	10.162042	5624.518	4858.849
158.60827	23.635196	6.35946	4.731208	56.46914	67.885925
27.918839	10.454915	4.465808	3.396529	28.689968	17.975882
188.022202	39.683615	6.816145	5.048609	285.4579	84.19487
188.022202	39.683615	6.816145	5.048609	285.4579	84.19487
1201.395742	32.826749	9.151669	5.042908	591.03625	38.4936
90.733747	16.792863	5.902426	4.113354	154.01028	89.73399
50.406596	11.40343	5.279727	3.795631	54.21869	51.4567
399.374542	133.12689	8.257369	7.254405	348.49265	365.92175
17.904217	5.242012	3.809106	2.45352	19.356592	15.16749
20.654976	5.769641	4.02929	2.696936	23.64496	24.438745
45.411602	6.186866	4.67939	2.592456	46.86515	4.9999995
607.5465	97.936165	8.742862	6.921752	866.33	912.1824

56.3463	23.536892	226.9073	195.25333	4.9999995	4.9999995
18.71308	22.33346	18.661325	19.65472	4.9999995	4.9999995
18.71308	22.33346	18.661325	19.65472	4.9999995	4.9999995
13.416318	15.528892	27.954607	32.08324	8.071006	4.9999995
13.416318	15.528892	27.954607	32.08324	8.071006	4.9999995
7272.2163	6442.216	8427.12	9564.738	1750.9393	1510.4337
40.67345	74.767555	23.91309	37.81719	14.47827	7.4999285
581.9693	319.82742	996.0362	1009.8826	137.4715	103.39741
581.9693	319.82742	996.0362	1009.8826	137.4715	103.39741
581.9693	319.82742	996.0362	1009.8826	137.4715	103.39741
42.511337	47.316437	53.759983	48.380653	7.366087	4.9999995
580.05774	309.65134	166.57216	279.15024	147.31837	30.890738
580.05774	309.65134	166.57216	279.15024	147.31837	30.890738
580.05774	309.65134	166.57216	279.15024	147.31837	30.890738
38.677002	112.99137	198.0664	211.87302	12.37475	9.673317
38.677002	112.99137	198.0664	211.87302	12.37475	9.673317
50.97804	72.98302	60.96391	76.256355	8.391361	5.534046
50.97804	72.98302	60.96391	76.256355	8.391361	5.534046
50.97804	72.98302	60.96391	76.256355	8.391361	5.534046
2649.4885	1528.491	4.9999995	683.01184	4.9999995	4.9999995
55.71165	58.026413	41.41226	61.896053	11.187442	9.199626
55.71165	58.026413	41.41226	61.896053	11.187442	9.199626
55.71165	58.026413	41.41226	61.896053	11.187442	9.199626
55.71165	58.026413	41.41226	61.896053	11.187442	9.199626
55.71165	58.026413	41.41226	61.896053	11.187442	9.199626
55.71165	58.026413	41.41226	61.896053	11.187442	9.199626
55.71165	58.026413	41.41226	61.896053	11.187442	9.199626
55.71165	58.026413	41.41226	61.896053	11.187442	9.199626
55.71165	58.026413	41.41226	61.896053	11.187442	9.199626
55.71165	58.026413	41.41226	61.896053	11.187442	9.199626
468.68402	165.90831	254.79205	267.65698	74.01574	54.42824
468.68402	165.90831	254.79205	267.65698	74.01574	54.42824
88.54673	72.23098	69.10701	95.09485	5.1202173	4.9999995
88.54673	72.23098	69.10701	95.09485	5.1202173	4.9999995
88.54673	72.23098	69.10701	95.09485	5.1202173	4.9999995
88.54673	72.23098	69.10701	95.09485	5.1202173	4.9999995
13.229952	23.650534	51.683292	50.42697	9.984784	8.078903
13.229952	23.650534	51.683292	50.42697	9.984784	8.078903
4079.4548	7542.8335	2048.233	5727.1533	1206.9851	1074.2717
25.86623	22.513414	24.510967	18.71702	4.9999995	4.9999995
124.80628	138.44899	145.73271	141.22801	47.080177	21.437443
3536.031	15703.441	2426.444	9875.187	916.3089	429.8667
41.293224	29.874746	52.27559	45.738464	20.212767	4.9999995
41.293224	29.874746	52.27559	45.738464	20.212767	4.9999995
41.293224	29.874746	52.27559	45.738464	20.212767	4.9999995
41.293224	29.874746	52.27559	45.738464	20.212767	4.9999995
70.661095	154.47083	54.859097	103.54679	13.013978	17.443794
959.80475	1298.537	833.61615	1248.1279	294.3003	234.96364
6463.189	9854.905	4314.11	8270.007	721.317	629.74835
6463.189	9854.905	4314.11	8270.007	721.317	629.74835
44.1767	440.88623	57.489166	284.74246	17.479078	16.99504
26.702272	41.971214	23.81603	28.357666	14.084179	4.9999995
481.99048	91.40249	73.19089	111.89658	38.740063	7.3127403
481.99048	91.40249	73.19089	111.89658	38.740063	7.3127403
1094.6561	1568.62	1830.1239	2085.4446	12.62307	16.36195
42.268364	27.207708	131.11864	100.0635	27.224863	14.457039
33.822685	39.3613	68.23471	55.34549	12.065369	8.917721
424.5385	364.9275	408.02182	484.34503	170.08841	75.70478
11.30732	20.827433	14.727421	26.039047	6.452072	4.9999995
23.22903	18.691462	12.882501	21.04316	5.998743	5.3945346
78.211716	15.459639	65.535835	61.39727	4.9999995	4.9999995
735.7998	317.16623	386.41223	427.38834	84.174446	94.87456

4.9999995	4.9999995	4.9999995	4.9999995	4.9644055	4.0103726
12.990769	5.328015	4.9999995	9.439593	4.163686	4.7584667
12.990769	5.328015	4.9999995	9.439593	4.163686	4.7584667
12.18428	6.1039963	7.3911967	5.45258	3.9699037	4.114166
12.18428	6.1039963	7.3911967	5.45258	3.9699037	4.114166
2082.0479	1585.8143	2661.453	1692.9456	12.640489	12.786555
11.882131	15.512499	11.70105	12.109811	5.209098	4.4042945
199.50027	140.75511	349.56735	180.62543	8.59467	8.438907
199.50027	140.75511	349.56735	180.62543	8.59467	8.438907
199.50027	140.75511	349.56735	180.62543	8.59467	8.438907
4.9999995	12.721012	9.479905	6.024745	4.9853845	5.14075
257.56638	29.390167	103.7782	53.212883	8.377992	7.988911
257.56638	29.390167	103.7782	53.212883	8.377992	7.988911
257.56638	29.390167	103.7782	53.212883	8.377992	7.988911
15.544708	24.532639	30.965668	27.977844	6.0604076	6.8449945
15.544708	24.532639	30.965668	27.977844	6.0604076	6.8449945
13.015921	6.199467	12.21724	5.9946613	5.521658	6.074525
13.015921	6.199467	12.21724	5.9946613	5.521658	6.074525
13.015921	6.199467	12.21724	5.9946613	5.521658	6.074525
4.9999995	52.430595	4.9999995	22.857723	9.825914	2.3499577
8.456173	17.444998	31.605783	19.350208	5.109831	5.4350786
8.456173	17.444998	31.605783	19.350208	5.109831	5.4350786
8.456173	17.444998	31.605783	19.350208	5.109831	5.4350786
8.456173	17.444998	31.605783	19.350208	5.109831	5.4350786
8.456173	17.444998	31.605783	19.350208	5.109831	5.4350786
8.456173	17.444998	31.605783	19.350208	5.109831	5.4350786
8.456173	17.444998	31.605783	19.350208	5.109831	5.4350786
8.456173	17.444998	31.605783	19.350208	5.109831	5.4350786
8.456173	17.444998	31.605783	19.350208	5.109831	5.4350786
8.456173	17.444998	31.605783	19.350208	5.109831	5.4350786
96.82903	87.914185	104.59483	78.83345	8.018048	7.671642
96.82903	87.914185	104.59483	78.83345	8.018048	7.671642
8.60255	11.052171	4.9999995	5.296138	5.529325	3.7482593
8.60255	11.052171	4.9999995	5.296138	5.529325	3.7482593
8.60255	11.052171	4.9999995	5.296138	5.529325	3.7482593
8.60255	11.052171	4.9999995	5.296138	5.529325	3.7482593
4.9999995	4.9999995	18.153198	8.506061	5.872989	6.8170834
4.9999995	4.9999995	18.153198	8.506061	5.872989	6.8170834
1425.9568	806.70807	1349.8768	860.25183	11.338854	11.0246725
5.9955926	4.9999995	4.9999995	4.9999995	4.0875463	4.3044343
93.732315	45.55535	46.18479	31.571632	6.975384	7.157064
1450.175	1059.9391	877.46155	790.2218	11.303015	11.176275
25.26781	7.292191	11.449231	8.977402	5.507208	5.70024
25.26781	7.292191	11.449231	8.977402	5.507208	5.70024
25.26781	7.292191	11.449231	8.977402	5.507208	5.70024
25.26781	7.292191	11.449231	8.977402	5.507208	5.70024
7.1945205	32.5263	21.927217	21.62086	5.736808	5.5724835
324.4311	231.68892	562.89	298.34418	10.322714	10.911717
777.96857	972.1451	1344.9619	900.45795	11.961581	11.790485
777.96857	972.1451	1344.9619	900.45795	11.961581	11.790485
13.286149	51.420334	15.369093	27.261484	5.357065	5.911976
19.919231	6.028212	7.713092	9.984779	4.390099	4.164884
88.411385	9.460082	69.15324	25.024178	7.7013855	6.200468
88.411385	9.460082	69.15324	25.024178	7.7013855	6.200468
10.206281	67.93023	41.479736	48.35923	8.734058	5.1675377
29.153067	6.1721544	16.861748	6.8883066	6.809992	6.2835765
10.196399	14.683931	9.53767	13.019489	5.2947197	5.550072
264.42377	101.24852	103.69087	83.60499	7.9930224	8.2185335
4.9999995	4.9999995	4.9999995	4.9999995	3.8165076	3.9438212
8.224569	4.9999995	4.9999995	4.9999995	4.1048446	4.5681486
4.9999995	12.1212	4.9999995	4.9999995	5.0930414	2.3499577
66.65602	155.02255	94.810074	92.07934	9.294816	9.497339

5.500935	4.0679245	7.5799675	7.086062	2.3467326	2.4279919
3.9066975	3.9826758	4.12796	3.86529	2.3467326	2.4279919
3.9066975	3.9826758	4.12796	3.86529	2.3467326	2.4279919
3.4182634	3.3974843	4.6756616	4.5485506	3.2149792	2.4279919
3.4182634	3.3974843	4.6756616	4.5485506	3.2149792	2.4279919
12.453372	12.307928	12.736248	12.618389	11.081052	11.439903
5.030363	5.86506	4.469638	4.771877	4.085673	3.668214
8.823429	7.998871	9.699494	9.391014	7.329967	7.5403786
8.823429	7.998871	9.699494	9.391014	7.329967	7.5403786
8.823429	7.998871	9.699494	9.391014	7.329967	7.5403786
5.087546	5.1645646	5.56461	5.108251	3.0732775	2.4279919
8.819718	7.9536505	7.1527667	7.5777636	7.439103	5.7869215
8.819718	7.9536505	7.1527667	7.5777636	7.439103	5.7869215
8.819718	7.9536505	7.1527667	7.5777636	7.439103	5.7869215
4.9588814	6.47958	7.3939986	7.1928806	3.8583853	4.054921
4.9588814	6.47958	7.3939986	7.1928806	3.8583853	4.054921
5.355707	5.8287396	5.739109	5.743083	3.2783608	3.2062829
5.355707	5.8287396	5.739109	5.743083	3.2783608	3.2062829
5.355707	5.8287396	5.739109	5.743083	3.2783608	3.2062829
11.014019	10.261244	2.3344212	8.841461	2.3467326	2.4279919
5.4854183	5.476534	5.2141614	5.444116	3.7141337	3.9765046
5.4854183	5.476534	5.2141614	5.444116	3.7141337	3.9765046
5.4854183	5.476534	5.2141614	5.444116	3.7141337	3.9765046
5.4854183	5.476534	5.2141614	5.444116	3.7141337	3.9765046
5.4854183	5.476534	5.2141614	5.444116	3.7141337	3.9765046
5.4854183	5.476534	5.2141614	5.444116	3.7141337	3.9765046
5.4854183	5.476534	5.2141614	5.444116	3.7141337	3.9765046
5.4854183	5.476534	5.2141614	5.444116	3.7141337	3.9765046
5.4854183	5.476534	5.2141614	5.444116	3.7141337	3.9765046
5.4854183	5.476534	5.2141614	5.444116	3.7141337	3.9765046
8.52075	7.048739	7.752356	7.5164394	6.4356647	6.6168866
8.52075	7.048739	7.752356	7.5164394	6.4356647	6.6168866
6.14995	5.811692	5.908574	6.0518217	2.573803	2.4279919
6.14995	5.811692	5.908574	6.0518217	2.573803	2.4279919
6.14995	5.811692	5.908574	6.0518217	2.573803	2.4279919
6.14995	5.811692	5.908574	6.0518217	2.573803	2.4279919
3.3984115	4.075333	5.513158	5.1657352	3.5430317	3.7806046
3.3984115	4.075333	5.513158	5.1657352	3.5430317	3.7806046
11.636798	12.52699	10.713436	11.852957	10.535038	10.949598
4.3837647	3.998289	4.503815	3.8016653	2.3467326	2.4279919
6.6416154	6.787432	6.9676757	6.6126523	5.7876873	5.2535625
11.425573	13.600112	10.966507	12.679465	10.157421	9.616259
5.0499916	4.446186	5.528633	5.0305443	4.5710063	2.4279919
5.0499916	4.446186	5.528633	5.0305443	4.5710063	2.4279919
5.0499916	4.446186	5.528633	5.0305443	4.5710063	2.4279919
5.0499916	4.446186	5.528633	5.0305443	4.5710063	2.4279919
5.831658	6.9461403	5.5942783	6.1757693	3.9277487	4.9453607
9.5384445	10.025298	9.441012	9.708057	8.464608	8.725792
12.305031	12.897419	11.775125	12.400587	9.7928095	10.158717
12.305031	12.897419	11.775125	12.400587	9.7928095	10.158717
5.1460795	8.473957	5.65751	7.610174	4.365209	4.904441
4.428035	4.977532	4.4629774	4.371319	4.0437183	2.4279919
8.561582	6.1615076	5.988676	6.283251	5.507947	3.6302216
8.561582	6.1615076	5.988676	6.283251	5.507947	3.6302216
9.721865	10.309741	10.542946	10.433864	3.884795	4.84819
5.0793104	4.303543	6.8127427	6.125393	5.004211	4.663981
4.7654424	4.876692	5.892806	5.2986283	3.8238106	3.9314744
8.383522	8.189564	8.413831	8.345742	7.656013	7.087643
3.1722012	3.8669095	3.8098526	4.2453465	2.8869739	2.4279919
4.2293096	3.6891792	3.6263895	3.9578702	2.787435	3.166536
5.9727454	3.3886535	5.836652	5.4352903	2.3467326	2.4279919
9.171591	7.986807	8.338986	8.16763	6.6199603	7.424053

2.350709	2.3408759	2.3618152	2.352752
3.7765641	2.5578697	2.3618152	3.523165
3.7765641	2.5578697	2.3618152	3.523165
3.6940415	2.714133	3.1838622	2.713648
3.6940415	2.714133	3.1838622	2.713648
10.973721	11.151194	11.631184	11.321924
3.6597424	4.0969563	3.8236775	3.9003673
7.590277	7.5092316	8.683072	8.006677
7.590277	7.5092316	8.683072	8.006677
7.590277	7.5092316	8.683072	8.006677
2.350709	3.785805	3.531699	2.852138
7.957231	5.0870504	6.954068	6.1650643
7.957231	5.0870504	6.954068	6.1650643
7.957231	5.0870504	6.954068	6.1650643
4.022765	4.8016486	5.226414	5.180871
4.022765	4.8016486	5.226414	5.180871
3.780518	2.7360554	3.8886185	2.8476713
3.780518	2.7360554	3.8886185	2.8476713
3.780518	2.7360554	3.8886185	2.8476713
2.350709	5.9891486	2.3618152	4.860441
3.2085156	4.268133	5.2574396	4.605299
3.2085156	4.268133	5.2574396	4.605299
3.2085156	4.268133	5.2574396	4.605299
3.2085156	4.268133	5.2574396	4.605299
3.2085156	4.268133	5.2574396	4.605299
3.2085156	4.268133	5.2574396	4.605299
3.2085156	4.268133	5.2574396	4.605299
3.2085156	4.268133	5.2574396	4.605299
3.2085156	4.268133	5.2574396	4.605299
3.2085156	4.268133	5.2574396	4.605299
6.5597563	6.7891617	6.964942	6.7559404
6.5597563	6.7891617	6.964942	6.7559404
3.2304492	3.5774732	2.3618152	2.67516
3.2304492	3.5774732	2.3618152	2.67516
3.2304492	3.5774732	2.3618152	2.67516
3.2304492	3.5774732	2.3618152	2.67516
2.350709	2.3408759	4.4519744	3.3630126
2.350709	2.3408759	4.4519744	3.3630126
10.418685	10.12784	10.65826	10.322233
2.787155	2.3408759	2.3618152	2.352752
6.5142684	5.76488	5.7986546	5.3757563
10.440105	10.562181	10.0166855	10.185456
4.6830173	2.9587145	3.7914703	3.4482164
4.6830173	2.9587145	3.7914703	3.4482164
4.6830173	2.9587145	3.7914703	3.4482164
4.6830173	2.9587145	3.7914703	3.4482164
3.0024228	5.235897	4.7277565	4.7771173
8.29063	8.268391	9.366451	8.756494
9.550444	10.419105	10.65195	10.399229
9.550444	10.419105	10.65195	10.399229
3.8070812	5.957973	4.2156496	5.136894
4.3596163	2.6987305	3.241848	3.6072707
6.4353776	3.3385913	6.37782	5.001696
6.4353776	3.3385913	6.37782	5.001696
3.4589298	6.3956637	5.6483765	6.0214953
4.886185	2.7306228	4.351959	3.0431654
3.4571464	4.011917	3.5394244	4.010012
7.9928718	7.002425	6.952891	6.8345838
2.350709	2.3408759	2.3618152	2.352752
3.1722012	2.3408759	2.3618152	2.352752
2.350709	3.714735	2.3618152	2.352752
6.0368934	7.650967	6.823078	6.97556

ASHGA5P046416	0.016292506	0.077996855	2.4871719	up	noncoding
ASHGA5P043872	0.000229821	0.011769168	11.0577383	up	noncoding
ASHGA5P043872	0.000229821	0.011769168	11.0577383	up	noncoding
ASHGA5P046049	0.000349439	0.013409132	2.4119265	up	noncoding
ASHGA5P025812	0.031123017	0.110685904	2.0602831	up	noncoding
ASHGA5P025812	0.031123017	0.110685904	2.0602831	up	noncoding
ASHGA5P026799	0.000656695	0.017168766	2.0351667	up	noncoding
ASHGA5P026799	0.000656695	0.017168766	2.0351667	up	noncoding
ASHGA5P032478	0.01262914	0.06880509	2.0892227	up	noncoding
ASHGA5P032478	0.01262914	0.06880509	2.0892227	up	noncoding
ASHGA5P032478	0.01262914	0.06880509	2.0892227	up	noncoding
ASHGA5P020317	0.009898103	0.061737522	2.328077	up	noncoding
ASHGA5P020317	0.009898103	0.061737522	2.328077	up	noncoding
ASHGA5P020317	0.009898103	0.061737522	2.328077	up	noncoding
ASHGA5P016541	0.01723248	0.079988469	2.4348507	up	noncoding
ASHGA5P046098	3.86444E-07	0.001647562	4.077901	up	noncoding
ASHGA5P038417	0.000271836	0.012302262	6.3859265	up	noncoding
ASHGA5P038417	0.000271836	0.012302262	6.3859265	up	noncoding
ASHGA5P031094	0.00605005	0.049583484	4.2383322	up	noncoding
ASHGA5P031094	0.00605005	0.049583484	4.2383322	up	noncoding
ASHGA5P057142	0.048554941	0.143101902	2.8529945	up	noncoding
ASHGA5P016549	0.037266017	0.122466478	2.6263744	up	noncoding
ASHGA5P026218	0.012098555	0.067400671	2.093307	up	noncoding
ASHGA5P053762	0.018578054	0.08287643	2.1374719	up	noncoding
ASHGA5P045934	0.003003908	0.035467016	2.0368639	up	noncoding
ASHGA5P047627	0.038173864	0.124228413	2.1818442	up	noncoding
ASHGA5P053394	0.001541695	0.026420993	2.0588543	up	noncoding
ASHGA5P048793	0.002314099	0.031638821	2.0525688	up	noncoding
ASHGA5P048793	0.002314099	0.031638821	2.0525688	up	noncoding
ASHGA5P053589	0.008350373	0.05708397	2.1762685	up	noncoding
ASHGA5P039589	0.002293898	0.031437116	2.4295175	up	noncoding
ASHGA5P015357	0.049601541	0.144887709	3.777448	up	noncoding
ASHGA5P039450	0.031019839	0.110553198	4.4514473	up	noncoding
ASHGA5P039450	0.031019839	0.110553198	4.4514473	up	noncoding
ASHGA5P041837	0.002365581	0.031814951	2.0304729	up	noncoding
ASHGA5P053012	0.044695244	0.13592263	3.4380831	up	noncoding
ASHGA5P038743	0.001848413	0.028874356	7.2016412	up	noncoding
ASHGA5P031128	0.006343598	0.050739988	2.5975002	up	noncoding
ASHGA5P049222	0.002543436	0.0327562	13.2848742	up	noncoding
ASHGA5P054618	0.000712037	0.017913741	4.9716648	up	noncoding
ASHGA5P030825	7.5138E-05	0.008283845	2.1553408	up	noncoding
ASHGA5P045201	0.004337371	0.041731826	4.9603582	up	noncoding
ASHGA5P045201	0.004337371	0.041731826	4.9603582	up	noncoding
ASHGA5P045201	0.004337371	0.041731826	4.9603582	up	noncoding
ASHGA5P046691	0.00046823	0.015177128	2.4840981	up	noncoding
ASHGA5P046691	0.00046823	0.015177128	2.4840981	up	noncoding
ASHGA5P046691	0.00046823	0.015177128	2.4840981	up	noncoding
ASHGA5P046691	0.00046823	0.015177128	2.4840981	up	noncoding
ASHGA5P046691	0.00046823	0.015177128	2.4840981	up	noncoding
ASHGA5P028238	0.011513999	0.066123362	2.0183217	up	noncoding
ASHGA5P031841	0.003999912	0.040535409	2.7906078	up	noncoding
ASHGA5P038659	0.005562947	0.047409675	2.6890893	up	noncoding
ASHGA5P027034	0.005533549	0.047322231	2.1036244	up	noncoding
ASHGA5P020758	0.004557848	0.042992804	2.0260814	up	noncoding
ASHGA5P036251	0.027287187	0.103048424	2.3689239	up	noncoding
ASHGA5P038013	0.00111681	0.022608782	2.2012925	up	noncoding
ASHGA5P053792	0.002076314	0.030074411	3.6802567	up	noncoding
ASHGA5P016384	0.003163908	0.036430985	2.2708292	up	noncoding
ASHGA5P050387	0.017777672	0.080955991	2.3332116	up	noncoding
ASHGA5P036997	0.003604926	0.038636328	7.0800417	up	noncoding
ASHGA5P049060	0.004546736	0.042958215	2.0816791	up	noncoding
ASHGA5P049060	0.004546736	0.042958215	2.0816791	up	noncoding

ENST00000391707	PPP2R3B-AS1	GENCODE	428	chrX	+
NR_036484	FEZF1-AS1	RefSeq	2653	chr7	+
NR_036484	FEZF1-AS1	RefSeq	2653	chr7	+
TCONS_00001617	XLOC_000947	LincRNAs identified	533	chr1	-
NR_038210	SAMD12-AS1	RefSeq	701	chr8	+
NR_038210	SAMD12-AS1	RefSeq	701	chr8	+
ENST00000539685	AP000462.1	GENCODE	384	chr11	+
ENST00000539685	AP000462.1	GENCODE	384	chr11	+
ENST00000572349	RP11-63A1.1	GENCODE	395	chr17	-
ENST00000572349	RP11-63A1.1	GENCODE	395	chr17	-
ENST00000572349	RP11-63A1.1	GENCODE	395	chr17	-
ENST00000511484	CTC-458I2.2	GENCODE	664	chr5	+
ENST00000511484	CTC-458I2.2	GENCODE	664	chr5	+
ENST00000511484	CTC-458I2.2	GENCODE	664	chr5	+
ENST00000431464	RP11-308D16.4	GENCODE	646	chrX	+
ENST00000457936	RP11-706O15.5	GENCODE	468	chrX	-
ENST00000428732	RP4-694A7.4	GENCODE	1247	chr1	+
ENST00000428732	RP4-694A7.4	GENCODE	1247	chr1	+
TCONS_00017504	XLOC_008152	LincRNAs identified	2622	chrX	-
TCONS_00017504	XLOC_008152	LincRNAs identified	2622	chrX	-
TCONS_00013698	XLOC_006356	LincRNAs identified	484	chr7	-
ENST00000431558	PRRT3-AS1	GENCODE	614	chr3	+
AI283175		UCR	417	chr2	+
uc004axb.2	BC070371	UCSC_knowngene	1370	chr9	-
ENST00000450803	RP11-27I1.2	GENCODE	912	chr9	+
ENST00000416191	RP11-441O15.3	GENCODE	666	chr10	+
ENST00000422183	GAS5	GENCODE	745	chr1	-
ENST00000562009	CTD-2311M21.2	GENCODE	3337	chr15	+
ENST00000562009	CTD-2311M21.2	GENCODE	3337	chr15	+
ENST00000519852	RP13-582O9.5	GENCODE	328	chr8	-
ENST00000514265	RP11-184M15.1	GENCODE	1623	chr4	-
ENST00000419604	RP11-64P14.7	GENCODE	497	chr9	+
uc003hhv.1	BC016361	UCSC_knowngene	823	chr4	-
uc003hhv.1	BC016361	UCSC_knowngene	823	chr4	-
uc003orh.1	DQ592954	UCSC_knowngene	2513	chr6	-
NR_002795	HOXA11-AS	RefSeq	1628	chr7	+
ENST00000455974	AC123023.1	GENCODE	555	chr3	+
ENST00000568752	RP11-989E6.10	GENCODE	3971	chr16	-
ENST00000560029	CRNDE	GENCODE	659	chr16	-
ENST00000522718	RP11-150O12.1	GENCODE	1093	chr8	-
uc010utu.2	WASH3P	UCSC_knowngene	1923	chr15	+
uc021pku.1	AX747026	UCSC_knowngene	2133	chr1	+
uc021pku.1	AX747026	UCSC_knowngene	2133	chr1	+
uc021pku.1	AX747026	UCSC_knowngene	2133	chr1	+
ENST00000441414	FMR1-IT1	GENCODE	343	chrX	+
ENST00000441414	FMR1-IT1	GENCODE	343	chrX	+
ENST00000441414	FMR1-IT1	GENCODE	343	chrX	+
ENST00000441414	FMR1-IT1	GENCODE	343	chrX	+
ENST00000441414	FMR1-IT1	GENCODE	343	chrX	+
ENST00000552707	RP11-554E23.4	GENCODE	374	chr12	+
NR_046242	LOC100505865	RefSeq	913	chr16	+
ENST00000424493	AC024560.2	GENCODE	1471	chr3	-
TCONS_00017518	XLOC_008186	LincRNAs identified	1269	chrX	-
NR_024281	LINC00599	RefSeq	2942	chr8	-
ENST00000436820	AC018470.4	GENCODE	1635	chr2	+
NR_024355	LINC00634	RefSeq	1506	chr22	+
ENST00000563434	RP11-4O1.2	GENCODE	4488	chr9	-
ENST00000429932	LL0XNC01-237H1.2	GENCODE	439	chrX	+
ENST00000547349	RP11-320M2.1	GENCODE	732	chr2	+
ENST00000416638	RP3-410C9.2	GENCODE	626	chr20	+
ENST00000572913	RP11-485G7.5	GENCODE	714	chr16	-
ENST00000572913	RP11-485G7.5	GENCODE	714	chr16	-

281724	282586	NR_027231	TCTACGTTACTTTATGTGGGAGTAG'	intergenic
121943711	121950131		CACGGTTCGACT(154860	natural antisense
121943711	121950131		CACGGTTCGACT(154860	natural antisense
106015902	106029242		AATGCCATGGGTGCAGCATTCAGAA	intergenic
119633239	119738306		GCAAAGTAATAC' 552860	intronic antisense
119633239	119738306		GCAAAGTAATAC' 552860	intronic antisense
115267537	115268295	ENST00000540545,	ATGTATCAAGATGTAGAATAGTACA	intronic antisense
115267537	115268295	ENST00000540545,	ATGTATCAAGATGTAGAATAGTACA	intronic antisense
44994135	45000400		AGAGCAAAGTTGAAGGCGAGCCGC1	bidirectional
44994135	45000400		AGAGCAAAGTTGAAGGCGAGCCGC1	bidirectional
44994135	45000400		AGAGCAAAGTTGAAGGCGAGCCGC1	bidirectional
79424183	79439706		AACATCCCAGATTGGCTTTCTTTCT/	intronic antisense
79424183	79439706		AACATCCCAGATTGGCTTTCTTTCT/	intronic antisense
79424183	79439706		AACATCCCAGATTGGCTTTCTTTCT/	natural antisense
135991633	136103777		GTTTGTAGAGAGACAGTTGGATTTT'	intergenic
3811138	3823602	ENST00000424415,	TCAACCAACAACTGTCGAAGAGGAC	intergenic
68962358	69004310		ACTGTGGGGATAAATTTAAAACATT	intronic antisense
68962358	69004310		ACTGTGGGGATAAATTTAAAACATT	intronic antisense
46458742	46461839	NM_001257291, NM	AAGTACAGCTGAAAAGATCACGAA/	exon sense-overlappi
46458742	46461839	NM_001257291, NM	AAGTACAGCTGAAAAGATCACGAA/	exon sense-overlappi
12713476	12714328		TTTTGGTGTAAAGCAGGTAAAAGCT	intergenic
9989087	9996471		GTAAGGGTCGTG' 100874032	natural antisense
171571428	171571847		TTCTTTGATGATTGGGTAGCGGCAA	intergenic
99957619	99958989	NM_001242797, NM	GATCATGATAATATTAGGGCCAGTG	intergenic
123608959	123614245		ATCTCTGAGAGTTAACAGCAGAAGC	intergenic
101190947	101195542		TATCTAGAGCCCTCCAAGAAGTCTT'	bidirectional
173833039	173836181	ENST00000434796,	GTTGGGACTATT/ 60674	intergenic
74646796	74656961	ENST00000564109	TACTATCAGCCATGGTCAACCCCAC(	intronic antisense
74646796	74656961	ENST00000564109	TACTATCAGCCATGGTCAACCCCAC(	intronic antisense
144362997	144363420	ENST00000518073	TGGCAAAGTAAGCGCGTGGGACAG(	intergenic
129489126	129491686		GTTTCCTCAGTGCTTTGAAGATTTTT	intergenic
125381636	125401964	ENST00000431442	AAACGGAAAGATTCCTCAACCATAA	intronic antisense
75144013	75196255		CGGATTCTAAGTATCAAGAAGAATC	intronic antisense
75144013	75196255		CGGATTCTAAGTATCAAGAAGAATC	intronic antisense
41757633	41760146		CCTCCATTAAAATCCATGTTTTGGG(	natural antisense
27225026	27228912	uc022aat.1	CTGGGGACTACC/ 221883	bidirectional
34244735	34310303		TCCCAATTACAGGGAAGCCTGCTGA	intergenic
33344308	33348279		GTGCTTGTTATTAGTTGTATTACATC	intergenic
54952781	54954528		AAATCAATAGGT/ 643911	intergenic
37278858	37351431		TTGAAGAATAAGAAGCCACAGCTTC	intergenic
102501015	102514305	NR_003659, uc010b	ACCCGGAGGCGC 374666	intergenic
235712454	235714587		AACAGGTCCTTATATTACTGCAGCC(	natural antisense
235712454	235714587		AACAGGTCCTTATATTACTGCAGCC(	natural antisense
235712454	235714587		AACAGGTCCTTATATTACTGCAGCC(	natural antisense
147028460	147029103		CTGATATGTTTGATGAACTTGGCAG'	intron sense-overlapp
147028460	147029103		CTGATATGTTTGATGAACTTGGCAG'	intron sense-overlapp
147028460	147029103		CTGATATGTTTGATGAACTTGGCAG'	intron sense-overlapp
147028460	147029103		CTGATATGTTTGATGAACTTGGCAG'	intron sense-overlapp
147028460	147029103		CTGATATGTTTGATGAACTTGGCAG'	intron sense-overlapp
102457132	102468598		CAGTCCAGACTCCATTTTTGCAGTC1	natural antisense
66442426	66444803		GCTGGAGGAGTA 100505865	intergenic
197387435	197392628		TCACCGTGAAATATCTTCTGAGACA(	intergenic
73167079	73225243		ATCAAGTGTTTTGCCTTCTCCCAAG(	intergenic
9757573	9760839		AGCTTATATGAT1 157627	intergenic
175193499	175195270		TGTTTAAGGTACATTGATCAATCTA/	intergenic
42348190	42354946		CCCCCTTCGCTGC 339674	intergenic
114794834	114800010		ATTCCTGTCATATGTTTTGTAAACA/	intergenic
102152508	102160619		TTGAACATCGTGTGACATGATGAAT	intergenic
10589853	10590920		CCAAAACCAGGTCTGAGGCACAGTC	intergenic
26171624	26174582		CAGGGTAAAATGAATCAAGTTCAGA	intergenic
11435665	11439068		AAAGAACTTCCTCTTTCAACGCATC'	intronic antisense
11435665	11439068		AAAGAACTTCCTCTTTCAACGCATC'	intronic antisense

NM_001024613	FEZF1	fez family zinc finge	-	121941447	121944565
NM_001160264	FEZF1	fez family zinc finge	-	121941447	121944565
NM_001101676	SAMD12	sterile alpha motif dc	-	119201694	119634184
NM_207506	SAMD12	sterile alpha motif dc	-	119390226	119634184
NM_001098517	CADM1	cell adhesion molecu	-	115044344	115375241
NM_014333	CADM1	cell adhesion molecu	-	115044344	115375241
NM_001012511	GOSR2	Golgi SNAP recepto	+	45000485	45014205
NM_004287	GOSR2	Golgi SNAP recepto	+	45000485	45018733
NM_054022	GOSR2	Golgi SNAP recepto	+	45000485	45018733
NM_001174071	SERINC5	serine incorporator 5	-	79407049	79551898
NM_178276	SERINC5	serine incorporator 5	-	79407049	79551898
NM_001174072	SERINC5	serine incorporator 5	-	79434549	79551898
NM_001114120	DEPDC1	DEP domain-contain	-	68939834	68962799
NM_017779	DEPDC1	DEP domain-contain	-	68939834	68962799
NM_001257291	SLC9A7	sodium/hydrogen exc	-	46458685	46618607
NM_032591	SLC9A7	sodium/hydrogen exc	-	46458685	46618607
NM_207351	PRRT3	proline-rich transmer	-	9987225	9994078
NM_002079	GOT1	aspartate aminotrans	-	101156626	101190530
NM_000781	CYP11A1	cholesterol side-chai	-	74630102	74660081
NM_001099773	CYP11A1	cholesterol side-chai	-	74630102	74658553
NM_001004450	OR1B1	olfactory receptor 1E	-	125390857	125391814
NM_001013442	EPGN	epigen precursor	+	75174203	75179307
NM_001144978	MTHFD2L	probable bifunctiona	+	75023828	75168814
NM_001134493	TOMM6	mitochondrial import	+	41755180	41757634
NM_005523	HOXA11	homeobox protein H	-	27220775	27224835
NM_001098721	GNG4	guanine nucleotide-b	-	235710984	235814054
NM_001098722	GNG4	guanine nucleotide-b	-	235710984	235813293
NM_004485	GNG4	guanine nucleotide-b	-	235710984	235814054
NM_001185075	FMR1	fragile X mental reta	+	146993468	147032647
NM_001185076	FMR1	fragile X mental reta	+	146993468	147032647
NM_001185081	FMR1	fragile X mental reta	+	146993468	147032647
NM_001185082	FMR1	fragile X mental reta	+	146993468	147032647
NM_002024	FMR1	fragile X mental reta	+	146993468	147032647
NM_024057	NUP37	nucleoporin Nup37	-	102467972	102512361
ENST00000381820	RMI2	RMI2, RecQ mediate	+	11410635	11445585
ENST00000572173	RMI2	RMI2, RecQ mediate	+	11343505	11445612

671.20362	129.280747	8.574494	7.259988	253.73955	243.23259
412.805026	25.867451	8.02908	4.562095	533.0699	947.89233
412.805026	25.867451	8.02908	4.562095	533.0699	947.89233
20.566902	5.609559	4.040058	2.769872	26.114342	15.859098
31.739433	12.351739	4.644624	3.601781	33.794193	27.391552
31.739433	12.351739	4.644624	3.601781	33.794193	27.391552
449.823663	138.671366	8.418452	7.393306	428.46072	418.45123
449.823663	138.671366	8.418452	7.393306	428.46072	418.45123
132.659919	37.885936	6.523572	5.460606	118.05167	43.920975
132.659919	37.885936	6.523572	5.460606	118.05167	43.920975
132.659919	37.885936	6.523572	5.460606	118.05167	43.920975
22.743672	6.434565	4.109725	2.890587	29.63126	36.770275
22.743672	6.434565	4.109725	2.890587	29.63126	36.770275
22.743672	6.434565	4.109725	2.890587	29.63126	36.770275
359.59739	75.331805	7.793698	6.509865	200.33554	159.46729
8944.815833	1231.241583	12.639341	10.611514	8595.918	9368.947
71.911374	6.037911	5.497023	2.822127	100.22652	173.47102
71.911374	6.037911	5.497023	2.822127	100.22652	173.47102
221.195779	31.768577	7.299253	5.215756	255.54494	163.8613
221.195779	31.768577	7.299253	5.215756	255.54494	163.8613
43.315978	8.989279	4.776907	3.264431	67.38665	95.45235
703.641905	164.357054	8.903529	7.510456	1022.31116	1127.4922
67.49698	20.258525	5.583039	4.517255	66.16638	37.27291
1030.742667	308.925797	9.570382	8.474476	1006.8697	697.5163
43.138424	14.813597	5.08925	4.0629	41.291748	33.647884
130.59945	42.824518	6.559588	5.43404	115.34968	62.319252
6606.02085	1741.364283	12.190637	11.148795	5135.8267	5439.718
2778.055667	764.232133	10.993856	9.956426	3092.0317	2993.7837
2778.055667	764.232133	10.993856	9.956426	3092.0317	2993.7837
3024.579917	772.504498	11.10722	9.985364	3539.2769	2626.9949
27.242682	8.593664	4.425282	3.144612	25.384558	18.145573
65.080668	8.370677	4.99228	3.074868	99.01095	4.9999995
546.065132	79.242659	8.168043	6.013768	725.00555	1385.9906
546.065132	79.242659	8.168043	6.013768	725.00555	1385.9906
1471.789923	428.32903	10.05482	9.033004	1288.5668	889.52844
7206.22425	1823.257095	12.157038	10.375434	5838.377	9070.881
356.271108	32.481188	7.814915	4.966589	442.84528	798.38965
366.931295	80.674987	8.047866	6.670743	334.519	149.86488
329.594535	14.497235	7.580697	3.848984	232.41103	34.40794
96.344149	13.104474	6.210244	3.896515	75.91766	73.24381
588.097642	167.631005	8.809325	7.70141	611.9099	578.4583
188.858192	28.840442	7.06676	4.756316	199.85606	211.78928
188.858192	28.840442	7.06676	4.756316	199.85606	211.78928
188.858192	28.840442	7.06676	4.756316	199.85606	211.78928
471.40069	123.228682	8.381908	7.069186	503.31506	182.40248
471.40069	123.228682	8.381908	7.069186	503.31506	182.40248
471.40069	123.228682	8.381908	7.069186	503.31506	182.40248
471.40069	123.228682	8.381908	7.069186	503.31506	182.40248
471.40069	123.228682	8.381908	7.069186	503.31506	182.40248
72.640222	22.29534	5.688251	4.675095	103.00626	67.224365
1066.92896	183.331068	9.304492	7.823913	964.86523	1588.3994
7296.37665	1693.200853	12.071685	10.644568	5915.099	3184.3025
475.522757	144.311274	8.460324	7.387447	599.65234	616.627
148.457023	53.57498	6.845718	5.827025	194.96333	138.16191
35.845211	10.985012	4.781252	3.53702	40.565536	45.376064
191.540465	57.279936	7.20382	6.065469	234.36052	215.14835
868.838228	182.299244	9.306713	7.426906	674.1523	478.80157
127.336625	32.907507	6.534521	5.351302	186.28818	174.99513
41.164003	10.116118	4.814613	3.592296	51.618027	92.024666
141.066022	10.697367	6.454201	3.630443	160.89691	184.76048
31.255109	9.43861	4.541928	3.484181	39.137627	47.573627
31.255109	9.43861	4.541928	3.484181	39.137627	47.573627

286.2082	407.20108	1479.0515	1357.7888	103.42919	52.098633
126.292206	299.64053	243.69633	326.23886	24.608393	52.58406
126.292206	299.64053	243.69633	326.23886	24.608393	52.58406
18.397598	26.094755	15.58177	21.35385	5.8255844	5.692079
39.784683	38.320972	18.836853	32.308346	17.086332	4.9999995
39.784683	38.320972	18.836853	32.308346	17.086332	4.9999995
426.77908	607.75775	463.51096	353.98224	110.7414	72.974335
426.77908	607.75775	463.51096	353.98224	110.7414	72.974335
158.24849	74.75836	201.48866	199.49136	25.856478	16.941402
158.24849	74.75836	201.48866	199.49136	25.856478	16.941402
158.24849	74.75836	201.48866	199.49136	25.856478	16.941402
14.986009	18.544651	19.214079	17.315758	4.9999995	5.2764606
14.986009	18.544651	19.214079	17.315758	4.9999995	5.2764606
14.986009	18.544651	19.214079	17.315758	4.9999995	5.2764606
216.52159	852.79456	154.82321	573.64215	50.20129	46.275692
7691.036	5072.604	11278.917	11661.473	1174.6461	861.8712
33.030704	26.936182	47.24785	50.55597	9.187589	5.365917
33.030704	26.936182	47.24785	50.55597	9.187589	5.365917
351.79648	84.525894	240.68584	230.76022	24.30252	14.01255
351.79648	84.525894	240.68584	230.76022	24.30252	14.01255
21.332623	14.926057	31.17306	29.62513	9.849689	4.9999995
893.99677	408.02588	317.27634	452.74908	199.98055	330.89413
60.293495	150.66992	34.72685	55.852325	16.68924	7.655116
1280.2148	1348.1548	689.9794	1161.721	348.5954	179.88452
46.12807	54.37024	32.43362	50.958984	13.08245	6.3591924
162.23169	226.8341	73.4513	143.41068	47.419125	12.146311
5093.9814	10018.558	4845.096	9102.945	1431.231	1452.2375
3243.6501	2915.7593	1784.7921	2638.3171	689.35205	566.5641
3243.6501	2915.7593	1784.7921	2638.3171	689.35205	566.5641
4451.2363	2510.1887	2168.2385	2851.5442	836.3771	890.17786
29.249052	42.98654	23.83895	23.851418	4.9999995	4.9999995
183.28923	30.182858	37.487514	35.513454	4.9999995	4.9999995
71.11921	192.34004	441.11566	460.81973	43.750675	70.05242
71.11921	192.34004	441.11566	460.81973	43.750675	70.05242
1542.3085	2227.5315	966.5786	1916.2257	490.2582	216.8051
1890.446	12629.073	4595.2935	9213.275	513.98956	787.1209
98.33504	193.13806	304.0135	300.90512	42.405724	70.89327
482.01184	479.72824	281.71155	473.75226	71.08186	72.7857
282.47983	325.15378	376.53638	726.57825	9.795468	7.806606
82.83483	97.42343	107.507774	141.13739	11.71605	12.41221
665.8767	634.0742	488.26755	549.9992	207.24457	125.57869
167.84314	62.420963	267.5137	223.72601	44.17083	13.305239
167.84314	62.420963	267.5137	223.72601	44.17083	13.305239
167.84314	62.420963	267.5137	223.72601	44.17083	13.305239
798.1058	454.80774	367.82016	521.9529	152.2401	48.15187
798.1058	454.80774	367.82016	521.9529	152.2401	48.15187
798.1058	454.80774	367.82016	521.9529	152.2401	48.15187
798.1058	454.80774	367.82016	521.9529	152.2401	48.15187
798.1058	454.80774	367.82016	521.9529	152.2401	48.15187
135.53238	48.452423	34.908245	46.71766	24.792227	10.256871
294.4443	1947.0696	279.03253	1327.7627	145.47002	150.4515
7922.1294	15645.356	1640.123	9471.25	2123.5808	401.21442
351.32935	284.3022	522.1803	479.04535	151.02914	79.07642
171.94913	126.90697	124.37538	134.38542	63.330936	18.990917
41.18108	44.83142	19.57098	23.546188	8.160525	4.9999995
210.1307	173.26314	125.26318	191.0769	57.9891	28.582373
788.0398	909.6095	1014.8951	1347.5311	109.37683	38.627575
160.71205	84.26235	73.81636	83.94568	38.77283	35.349983
29.021847	30.817965	18.02045	25.48106	11.914889	6.75845
106.64323	20.279812	202.63623	171.17947	10.11433	13.367831
13.775978	28.636839	29.307579	29.099005	13.93261	6.305507
13.775978	28.636839	29.307579	29.099005	13.93261	6.305507

186.7882	125.94431	170.97055	136.4536	7.5304184	7.66034
4.9999995	44.793858	6.998294	21.2201	8.572103	9.550927
4.9999995	44.793858	6.998294	21.2201	8.572103	9.550927
4.9999995	5.2556195	5.846801	6.037268	4.249222	4.006304
23.316027	9.069829	12.647221	6.991023	4.6161747	4.721788
23.316027	9.069829	12.647221	6.991023	4.6161747	4.721788
141.95694	188.64235	200.76328	116.94989	8.283264	8.401617
141.95694	188.64235	200.76328	116.94989	8.283264	8.401617
40.592106	48.840233	59.821777	35.26362	6.4327116	5.3407936
40.592106	48.840233	59.821777	35.26362	6.4327116	5.3407936
40.592106	48.840233	59.821777	35.26362	6.4327116	5.3407936
10.624071	5.6570206	6.6374264	5.4124136	4.433352	5.106573
10.624071	5.6570206	6.6374264	5.4124136	4.433352	5.106573
10.624071	5.6570206	6.6374264	5.4124136	4.433352	5.106573
65.69278	90.40048	115.18681	84.23378	7.1943493	7.0752144
1531.2681	848.3157	1884.7251	1086.6233	12.57487	12.738068
4.9999995	4.9999995	6.0128636	5.6610947	6.1965528	7.2003274
4.9999995	4.9999995	6.0128636	5.6610947	6.1965528	7.2003274
29.558603	51.98918	30.938353	39.810257	7.540243	7.11353
29.558603	51.98918	30.938353	39.810257	7.540243	7.11353
14.987499	8.451028	7.006478	8.640983	5.6135015	6.361598
52.374027	165.94241	121.35799	115.593216	9.5236	9.78898
32.16271	23.79824	19.258718	21.987127	5.5886607	5.125633
564.3572	141.6647	399.7734	219.27956	9.506457	9.122319
22.840633	15.566407	19.484777	11.54812	4.9049745	4.9935856
96.18481	22.041058	47.330997	31.824804	6.3975606	5.796814
1390.6674	2144.3096	2230.1643	1799.5759	11.833317	11.955706
802.4681	930.3179	836.9694	759.72125	11.116395	11.127507
802.4681	930.3179	836.9694	759.72125	11.116395	11.127507
761.2903	719.28723	787.252	640.6425	11.311047	10.937656
8.333652	11.620361	14.221619	7.386353	4.2073703	4.177958
6.525768	17.860292	9.065646	6.772355	6.1789713	2.3499577
8.501146	226.56932	23.343193	103.2392	9.033738	10.067459
8.501146	226.56932	23.343193	103.2392	9.033738	10.067459
717.0488	386.5696	420.45953	338.83295	9.865118	9.4597025
115.46491	1405.8851	5373.6353	2743.4468	12.017592	12.688867
14.711951	39.46258	6.0835543	21.33005	8.319926	9.309109
84.81719	104.5819	77.8727	72.91057	7.9318666	6.9931664
19.312536	32.781372	4.9999995	12.28743	7.4073486	5.023758
4.9999995	20.662361	14.367782	14.468439	5.793828	6.0104623
241.05762	138.1941	159.23903	134.47202	8.786216	8.86651
72.88825	15.317427	16.209726	11.151178	7.190648	7.4719305
72.88825	15.317427	16.209726	11.151178	7.190648	7.4719305
72.88825	15.317427	16.209726	11.151178	7.190648	7.4719305
267.3392	75.55464	107.07337	89.01291	8.492818	7.2694163
267.3392	75.55464	107.07337	89.01291	8.492818	7.2694163
267.3392	75.55464	107.07337	89.01291	8.492818	7.2694163
267.3392	75.55464	107.07337	89.01291	8.492818	7.2694163
267.3392	75.55464	107.07337	89.01291	8.492818	7.2694163
35.842136	27.482635	16.534939	18.863232	6.239457	5.8973746
136.79701	316.6172	155.5712	195.07948	9.441455	10.244089
4488.196	1711.3002	457.1824	977.7313	12.033515	11.220499
274.12427	91.559204	161.61008	108.46853	8.753903	8.950588
121.18968	43.63532	38.717896	35.58513	7.158551	6.879056
12.041229	18.41108	10.624661	11.67258	4.8789563	5.381849
103.79778	54.787468	55.23336	43.289536	7.4206467	7.4934654
104.1069	112.54352	487.63858	241.50206	8.927469	8.59573
33.761803	24.822588	30.386644	34.351192	7.087643	7.210077
10.659899	11.817322	6.852397	12.693749	5.224572	6.315923
4.9999995	11.231161	14.706201	9.764679	6.87187	7.289172
6.8251214	11.381239	10.0069	8.180283	4.8281054	5.4447474
6.8251214	11.381239	10.0069	8.180283	4.8281054	5.4447474

7.8272285	8.357157	10.250535	9.821284	6.930485	6.5517464
6.6574407	7.9084496	7.685308	7.80025	4.862937	6.5657587
6.6574407	7.9084496	7.685308	7.80025	4.862937	6.5657587
3.8823683	4.239607	3.8863428	3.9765046	2.742965	3.246647
4.9975	4.8327775	4.1396093	4.559895	4.331605	2.4279919
4.9975	4.8327775	4.1396093	4.559895	4.331605	2.4279919
8.391178	8.929193	8.598193	7.90727	7.027333	7.0325565
8.391178	8.929193	8.598193	7.90727	7.027333	7.0325565
6.9713655	5.864566	7.417538	7.1144567	4.934734	4.900222
6.9713655	5.864566	7.417538	7.1144567	4.934734	4.900222
6.9713655	5.864566	7.417538	7.1144567	4.934734	4.900222
3.5806754	3.678304	4.1675897	3.6918578	2.3467326	3.1313941
3.5806754	3.678304	4.1675897	3.6918578	2.3467326	3.1313941
3.5806754	3.678304	4.1675897	3.6918578	2.3467326	3.1313941
7.4377666	9.417315	7.057797	8.579748	5.885928	6.3799043
12.535432	11.972755	13.13357	12.8813505	10.497507	10.625169
4.7332783	4.289108	5.3939104	5.1689596	3.4127748	3.1568046
4.7332783	4.289108	5.3939104	5.1689596	3.4127748	3.1568046
8.12047	6.047019	7.6684155	7.30584	4.8446326	4.6193247
8.12047	6.047019	7.6684155	7.30584	4.8446326	4.6193247
4.096808	3.3328784	4.821272	4.435387	3.5225472	2.4279919
9.441012	8.359835	8.057017	8.250728	7.889124	9.230179
5.60286	6.9065914	4.964533	5.3099575	4.293543	3.6972954
9.958928	10.072502	9.167778	9.594307	8.708695	8.338426
5.205475	5.377553	4.8757076	5.1782045	3.9351397	3.4204826
7.008871	7.519272	5.993535	6.6414747	5.799244	4.3964095
11.951582	12.9224415	11.9368925	12.543884	10.781179	11.382436
11.282167	11.167049	10.5040245	10.765995	9.722932	10.000871
11.282167	11.167049	10.5040245	10.765995	9.722932	10.000871
11.776076	10.956213	10.79228	10.8700485	10.017597	10.67332
4.5625024	5.0135794	4.464936	4.125343	2.3467326	2.4279919
7.196364	4.464068	5.076396	4.687924	2.3467326	2.4279919
5.8396645	7.2676635	8.527192	8.272539	5.684752	6.9781785
5.8396645	7.2676635	8.527192	8.272539	5.684752	6.9781785
10.222787	10.797219	9.663778	10.3203125	9.204457	8.617914
10.528252	13.281222	11.865097	12.5612	9.278971	10.480659
6.2985687	7.275075	7.9996448	7.6871657	5.6404276	6.995111
8.561983	8.590928	7.894612	8.314643	6.3829923	7.0290794
7.8046746	8.024868	8.298724	8.924807	3.5169606	3.7271729
6.0557256	6.255379	6.534135	6.611933	3.7795494	4.4300017
9.013358	8.995454	8.670761	8.523654	7.9403963	7.8198905
7.06087	5.5886607	7.8213663	7.267085	5.6980553	4.537126
7.06087	5.5886607	7.8213663	7.267085	5.6980553	4.537126
7.06087	5.5886607	7.8213663	7.267085	5.6980553	4.537126
9.289892	8.517357	8.269833	8.452133	7.489456	6.4353776
9.289892	8.517357	8.269833	8.452133	7.489456	6.4353776
9.289892	8.517357	8.269833	8.452133	7.489456	6.4353776
9.289892	8.517357	8.269833	8.452133	7.489456	6.4353776
9.289892	8.517357	8.269833	8.452133	7.489456	6.4353776
6.7579703	5.203842	4.972126	5.058739	4.8728247	4.1421285
7.868254	10.598185	7.880502	9.794467	7.4165874	8.085681
12.580116	13.593259	10.397721	12.605001	11.36705	9.514151
8.118296	7.833856	8.773338	8.331963	7.4778247	7.156011
7.097624	6.6576986	6.737322	6.544054	6.218216	5.0705757
5.046386	5.0784626	4.193379	4.108478	3.2318087	2.4279919
7.3998876	7.111563	6.746698	7.050657	6.0947323	5.674794
9.270608	9.508995	9.728731	9.808744	7.0081854	6.1127987
6.9941425	6.042271	6.000761	5.872233	5.509366	5.984674
4.551494	4.498391	4.0814533	4.215843	3.803711	3.5167973
6.4189997	3.82294	7.425077	6.897148	3.562583	4.543818
3.4580486	4.3790574	4.7336884	4.4079237	4.0265036	3.4093049
3.4580486	4.3790574	4.7336884	4.4079237	4.0265036	3.4093049

7.49635	7.3420224	7.657093	7.5822287
2.350709	5.7374434	3.1077106	4.748013
2.350709	5.7374434	3.1077106	4.748013
2.350709	2.5467792	2.8766468	2.8554862
4.5739746	3.2745988	3.9360118	3.0665069
4.5739746	3.2745988	3.9360118	3.0665069
7.1065903	7.9547024	7.898885	7.339766
7.1065903	7.9547024	7.898885	7.339766
5.3420205	5.876349	6.169448	5.54086
5.3420205	5.876349	6.169448	5.54086
5.3420205	5.876349	6.169448	5.54086
3.5101712	2.616294	3.0371811	2.7017462
3.5101712	2.616294	3.0371811	2.7017462
3.5101712	2.616294	3.0371811	2.7017462
6.016551	6.8289957	7.10093	6.8468814
10.518528	10.201367	11.137936	10.688578
2.350709	2.3408759	2.9085991	2.7629974
2.350709	2.3408759	2.9085991	2.7629974
4.904108	5.976059	5.225091	5.7253222
4.904108	5.976059	5.225091	5.7253222
3.97025	3.1699255	3.1095936	3.3862748
5.702269	7.7481146	7.1706767	7.322373
5.020739	4.7562895	4.5342627	4.8014026
9.08696	7.5194373	8.885209	8.308131
4.5450134	4.101447	4.5491076	3.8262122
6.549296	4.6318274	5.8395257	5.387936
10.385819	11.5615225	11.374494	11.407322
9.5945215	10.3476305	9.942313	10.130286
9.5945215	10.3476305	9.942313	10.130286
9.518845	9.960872	9.854203	9.887344
3.189057	3.6515532	4.101447	3.1508884
2.8877764	4.3029103	3.4660807	3.0177178
3.2139766	8.233379	4.8191915	7.1531315
3.2139766	8.233379	4.8191915	7.1531315
9.427729	9.046899	8.959087	8.9419365
6.8123584	10.969803	12.678185	12.032627
3.9452424	5.539167	2.9239688	4.755618
6.374604	7.0564795	6.5431466	6.638154
4.3183746	5.2479434	2.3618152	3.9216375
2.350709	4.5314713	4.1178923	4.169465
7.8594213	7.4805417	7.5460916	7.5621157
6.1615076	4.0761456	4.2916765	3.7733836
6.1615076	4.0761456	4.2916765	3.7733836
6.1615076	4.0761456	4.2916765	3.7733836
8.00898	6.559858	6.9987216	6.9227233
8.00898	6.559858	6.9987216	6.9227233
8.00898	6.559858	6.9987216	6.9227233
8.00898	6.559858	6.9987216	6.9227233
8.00898	6.559858	6.9987216	6.9227233
5.1676702	4.9801993	4.3231797	4.5645695
7.0504174	8.755194	7.511914	8.123682
12.1123	11.259593	9.077545	10.536766
8.046344	6.848503	7.5668983	7.2291
6.881053	5.6934676	5.5432744	5.555566
3.6769385	4.3509207	3.690627	3.843833
6.660993	6.0544424	6.052504	5.855347
6.6660466	7.161613	9.160876	8.451919
5.089528	4.820091	5.201601	5.5025525
3.5140042	3.673463	3.0783288	3.9674692
2.350709	3.6001558	4.150476	3.5749178
2.9399204	3.6191301	3.6034086	3.306817
2.9399204	3.6191301	3.6034086	3.306817

ASHGA5P058233	6.09476E-05	0.007814388	3.5029966	up	noncoding
ASHGA5P019758	0.007539673	0.054581432	2.1017047	up	noncoding
ASHGA5P048229	0.018774576	0.083325418	2.1731903	up	noncoding
ASHGA5P040805	0.002345623	0.031719059	2.6178864	up	noncoding
ASHGA5P028982	0.011584167	0.066244722	4.9049256	up	noncoding
ASHGA5P029695	0.020841213	0.088852355	2.0492451	up	noncoding
ASHGA5P021779	0.011919384	0.066970246	2.2416072	up	noncoding
ASHGA5P021779	0.011919384	0.066970246	2.2416072	up	noncoding
ASHGA5P053420	0.000295665	0.012593001	2.5127246	up	noncoding
ASHGA5P053420	0.000295665	0.012593001	2.5127246	up	noncoding
ASHGA5P053590	0.000419662	0.014598519	2.925979	up	noncoding
ASHGA5P023343	0.014097745	0.072819416	2.0700087	up	noncoding
ASHGA5P031491	0.001141461	0.022709835	2.7244736	up	noncoding
ASHGA5P053426	0.015265681	0.075646627	3.4905115	up	noncoding
ASHGA5P047454	0.002327634	0.031698104	2.3649914	up	noncoding
ASHGA5P047454	0.002327634	0.031698104	2.3649914	up	noncoding
ASHGA5P032882	0.000186559	0.01146874	3.537921	up	noncoding
ASHGA5P048847	0.027985353	0.104553957	2.072057	up	noncoding
ASHGA5P019500	0.000205287	0.01146874	2.2665064	up	noncoding
ASHGA5P019500	0.000205287	0.01146874	2.2665064	up	noncoding
ASHGA5P019500	0.000205287	0.01146874	2.2665064	up	noncoding
ASHGA5P019500	0.000205287	0.01146874	2.2665064	up	noncoding
ASHGA5P052176	0.045264019	0.136923261	5.3556423	up	noncoding
ASHGA5P038050	0.038189346	0.124228413	2.3653695	up	noncoding
ASHGA5P030222	0.003565561	0.038481895	2.1671536	up	noncoding
ASHGA5P031609	0.02127184	0.089913135	2.7108231	up	noncoding
ASHGA5P031609	0.02127184	0.089913135	2.7108231	up	noncoding
ASHGA5P031609	0.02127184	0.089913135	2.7108231	up	noncoding
ASHGA5P036678	0.009643042	0.060938813	2.0202326	up	noncoding
ASHGA5P028716	0.003063715	0.035804635	2.79383	up	noncoding
ASHGA5P043150	0.004226782	0.04130748	2.0059552	up	noncoding
ASHGA5P027965	0.012353901	0.068094238	2.2832466	up	noncoding
ASHGA5P027965	0.012353901	0.068094238	2.2832466	up	noncoding
ASHGA5P027965	0.012353901	0.068094238	2.2832466	up	noncoding
ASHGA5P027965	0.012353901	0.068094238	2.2832466	up	noncoding
ASHGA5P027965	0.012353901	0.068094238	2.2832466	up	noncoding
ASHGA5P025988	0.000656655	0.017168766	3.3232276	up	noncoding
ASHGA5P025988	0.000656655	0.017168766	3.3232276	up	noncoding
ASHGA5P025988	0.000656655	0.017168766	3.3232276	up	noncoding
ASHGA5P025988	0.000656655	0.017168766	3.3232276	up	noncoding
ASHGA5P058526	0.001735063	0.027807594	3.6667672	up	noncoding
ASHGA5P030337	0.016927018	0.079250679	2.5103755	up	noncoding
ASHGA5P036844	0.049417546	0.144516069	2.2154665	up	noncoding
ASHGA5P034471	0.004172267	0.041167338	4.534002	up	noncoding
ASHGA5P037513	0.001409909	0.025119259	3.1456113	up	noncoding
ASHGA5P037513	0.001409909	0.025119259	3.1456113	up	noncoding
ASHGA5P037513	0.001409909	0.025119259	3.1456113	up	noncoding
ASHGA5P038151	0.001234336	0.023574374	2.1646267	up	noncoding
ASHGA5P022834	0.019605583	0.085393317	3.8997606	up	noncoding
ASHGA5P038746	0.000768329	0.018599313	2.0219844	up	noncoding
ASHGA5P017188	0.002669178	0.033470143	6.1438984	up	noncoding
ASHGA5P044925	0.000437132	0.014669266	3.1735727	up	noncoding
ASHGA5P044925	0.000437132	0.014669266	3.1735727	up	noncoding
ASHGA5P042482	0.008658899	0.058001887	2.3345408	up	noncoding
ASHGA5P042482	0.008658899	0.058001887	2.3345408	up	noncoding
ASHGA5P042482	0.008658899	0.058001887	2.3345408	up	noncoding
ASHGA5P015253	0.043102032	0.13312867	3.7282937	up	noncoding
ASHGA5P058657	3.0333E-05	0.005526678	3.8498244	up	noncoding
ASHGA5P020516	0.003425232	0.037690616	2.0185337	up	noncoding
ASHGA5P022824	0.000354925	0.013460607	2.2690839	up	noncoding
ASHGA5P022824	0.000354925	0.013460607	2.2690839	up	noncoding
ASHGA5P028559	0.000504064	0.015567285	3.7949139	up	noncoding

TCONS_00028402	XLOC_013754	LincRNAs identified	514	chr20	-
ENST00000502693	RP11-323F5.2	GENCODE	724	chr4	+
TCONS_00013381	XLOC_006016	LincRNAs identified	2334	chr7	+
ENST00000445225	RP11-277L2.2	GENCODE	5730	chr1	+
ENST00000548107	CTD-2314B22.3	GENCODE	617	chr14	-
ENST00000554181	RP11-736P16.1	GENCODE	412	chr14	+
NR_037793	LRR1	RefSeq	1853	chr14	+
NR_037793	LRR1	RefSeq	1853	chr14	+
ENST00000443799	GAS5	GENCODE	897	chr1	-
ENST00000443799	GAS5	GENCODE	897	chr1	-
ENST00000521207	RP13-582O9.5	GENCODE	429	chr8	-
ENST00000584327	SNHG15	GENCODE	505	chr7	-
NR_047572	LOC100128770	RefSeq	3061	chr16	+
ENST00000534117	RP11-163N6.2	GENCODE	502	chr8	-
AY034104		LincRNAs identified	306	chr2	+
AY034104		LincRNAs identified	306	chr2	+
NR_027179	C17orf76-AS1	RefSeq	376	chr17	+
ENST00000561107	RP11-752G15.6	GENCODE	790	chr15	+
ENST00000488310	RP4-584D14.5	GENCODE	577	chr7	-
ENST00000488310	RP4-584D14.5	GENCODE	577	chr7	-
ENST00000488310	RP4-584D14.5	GENCODE	577	chr7	-
ENST00000488310	RP4-584D14.5	GENCODE	577	chr7	-
ENST00000504989	RP11-325I22.2	GENCODE	1616	chr5	-
ENST00000426112	RP3-439F8.1	GENCODE	2949	chr22	+
uc002blm.3	DQ596274	UCSC_knowngene	260	chr15	-
ENST00000568827	RP11-21M24.2	GENCODE	1415	chr16	+
ENST00000568827	RP11-21M24.2	GENCODE	1415	chr16	+
ENST00000568827	RP11-21M24.2	GENCODE	1415	chr16	+
uc002wrh.3	HSPC072	UCSC_knowngene	1951	chr20	-
uc001unz.1	AK093279	UCSC_knowngene	2100	chr13	+
uc003uch.1	PMS2L2	UCSC_knowngene	957	chr7	-
ENST00000547866	RP11-386G11.5	GENCODE	2114	chr12	+
ENST00000547866	RP11-386G11.5	GENCODE	2114	chr12	+
ENST00000547866	RP11-386G11.5	GENCODE	2114	chr12	+
ENST00000547866	RP11-386G11.5	GENCODE	2114	chr12	+
ENST00000547866	RP11-386G11.5	GENCODE	2114	chr12	+
NR_047694	PIGT	RefSeq	2068	chr20	+
NR_047694	PIGT	RefSeq	2068	chr20	+
NR_047694	PIGT	RefSeq	2068	chr20	+
NR_047694	PIGT	RefSeq	2068	chr20	+
uc009xzb.3	CASC2	UCSC_knowngene	2333	chr10	+
TCONS_00008667	XLOC_004182	LincRNAs identified	1012	chr4	-
ENST00000438154	RP5-1112F19.2	GENCODE	2326	chr20	-
ENST00000431363	ZNF833P	pseudogene	1407	chr19	+
ENST00000417820	AL773604.8	GENCODE	1031	chr21	+
ENST00000417820	AL773604.8	GENCODE	1031	chr21	+
ENST00000417820	AL773604.8	GENCODE	1031	chr21	+
ENST00000565519	RP11-384L8.1	GENCODE	1891	chr3	-
ENST00000563823	AC004158.2	GENCODE	563	chr16	-
TCONS_00023262	XLOC_011248	LincRNAs identified	3624	chr15	+
ENST00000438702	RP5-881L22.5	GENCODE	754	chr20	-
NR_024522	TMEM67	RefSeq	3381	chr8	+
NR_024522	TMEM67	RefSeq	3381	chr8	+
uc003pdp.3	KIAA1702	UCSC_knowngene	4385	chr6	+
uc003pdp.3	KIAA1702	UCSC_knowngene	4385	chr6	+
uc003pdp.3	KIAA1702	UCSC_knowngene	4385	chr6	+
ENST00000418442	AC005062.2	GENCODE	587	chr7	-
Y13808		RNAdb	205	chr3	+
ENST00000515065	RP11-423H2.1	GENCODE	628	chr5	+
NR_028477	RBMX	RefSeq	2132	chrX	-
NR_028477	RBMX	RefSeq	2132	chrX	-
ENST00000438752	DLEU2	GENCODE	2090	chr13	-

43766346	43767617		TCAACATCAGTGTACAGAGCTTTGG		intergenic
4543966	4639631		CAGGATAGAGCAAACATTGAGTGG		natural antisense
25878710	25888418		ACTGGAAACCAGAAGTAATGTCAG		intergenic
149576489	149651107	ENST00000325963,	AGATATGCACTTGGCCTGAGAAATG		intergenic
19912754	19925311	ENST00000547779,	TAAAGGGGGCTCTCTGGCCTTGAAA		intergenic
87402983	87455469		CACAGTTTAGAGATTTGAAAGCCCA		intergenic
50065414	50081390	ENST00000298288,	AGTTATGAAGCT	122769	exon sense-overlappi
50065414	50081390	ENST00000298288,	AGTTATGAAGCT	122769	exon sense-overlappi
173834490	173837127	ENST00000421068,	TGTAAATGCAGC	60674	bidirectional
173834490	173837127	ENST00000421068,	TGTAAATGCAGC	60674	bidirectional
144363195	144363780		GTGAAAAAATGTGAATTGTTCTACT		intergenic
45022735	45026159	ENST00000582727,	ACTGTCACAGCA	285958	intergenic
3082481	3089133		GGCCATGGGAGG	100128770	natural antisense
61324898	61325813		GGGAGCCATCTCATCTTTATAACCT		intergenic
75186481	75252012		TGGAAAAAGTAAGCCTAAATCGGG		exon sense-overlappi
75186481	75252012		TGGAAAAAGTAAGCCTAAATCGGG		exon sense-overlappi
16342300	16344525	ENST00000460249,	GTAAGCCTTGAG	125144	intergenic
83419323	83425956	ENST00000558174	TCTGTTCTAGATGTTTTCTTCTAAAT		natural antisense
150060865	150069679		ATGGAGCTAGACTACTCTCCTGTTT		natural antisense
150060865	150069679		ATGGAGCTAGACTACTCTCCTGTTT		natural antisense
150060865	150069679		ATGGAGCTAGACTACTCTCCTGTTT		natural antisense
150060865	150069679		ATGGAGCTAGACTACTCTCCTGTTT		natural antisense
1363696	1380182		ATGTGCGCTATTGTTAGATCTCCGG		intergenic
46937391	46944093		GTTTTCCTGCTTGGAAGTTGCCAGA		intergenic
85747305	85747565	ENST00000335068,	AGTACATATTGGCCAGCGGCTCCGA		intergenic
22386179	22387594		GCAGCTGATGGAAGACAACAGGGC		intronic antisense
22386179	22387594		GCAGCTGATGGAAGACAACAGGGC		intronic antisense
22386179	22387594		GCAGCTGATGGAAGACAACAGGGC		natural antisense
18768614	18775228		TGTCTTCTTGGAT	29075	intergenic
21872311	21906571		CTGCTCACATCTGGTCTGTTCCCCA		intergenic
74880904	74987787		ACATCCTAATTT	5380	intergenic
49392154	49412980		ATCGGCCTCTAACACTGCAAGATTT		natural antisense
49392154	49412980		ATCGGCCTCTAACACTGCAAGATTT		natural antisense
49392154	49412980		ATCGGCCTCTAACACTGCAAGATTT		natural antisense
49392154	49412980		ATCGGCCTCTAACACTGCAAGATTT		natural antisense
49392154	49412980		ATCGGCCTCTAACACTGCAAGATTT		natural antisense
44044706	44054885		CGGGAAGGAGTG	51604	exon sense-overlappi
44044706	44054885		CGGGAAGGAGTG	51604	exon sense-overlappi
44044706	44054885		CGGGAAGGAGTG	51604	exon sense-overlappi
44044706	44054885		CGGGAAGGAGTG	51604	exon sense-overlappi
119806331	119859647		CTTCCCCAAGGA	255082	bidirectional
178141536	178148458	TCONS_00009177	CGTGGTACATAGAAGCTGAGCAAC		intergenic
50448335	50479451		GAATTGTGCATGCAACTTCCAATCT		intergenic
11759178	11763692		GTTGCTTTTCGTA	401898	
46222491	46224632		CAGGAGCATCAATTTAACCCAAAGA		bidirectional
46222491	46224632		CAGGAGCATCAATTTAACCCAAAGA		bidirectional
46222491	46224632		CAGGAGCATCAATTTAACCCAAAGA		bidirectional
32278179	32280070		CAAGCATTATGCATGACATTTAGTG		bidirectional
72557230	72661585	ENST00000563770	GCAAAGAAACAAGAAGAGCTTAAA		intergenic
50655997	50660476		ACTATCTGGAAAACTGTGCTAATAG		intergenic
42976191	42983825		GGAGACAGAGGAGCATTAGGCTAC		natural antisense
94767071	94831460		ACTATGAATGCT	91147	exon sense-overlappi
94767071	94831460		ACTATGAATGCT	91147	exon sense-overlappi
56965018	56969403		TTGTGGATTTGACTTCTCTGATTCC		exon sense-overlappi
56965018	56969403		TTGTGGATTTGACTTCTCTGATTCC		intron sense-overlapp
56965018	56969403		TTGTGGATTTGACTTCTCTGATTCC		intron sense-overlapp
20046727	20171342		CTTTGACAAGATAAGAATAACAATC		intergenic
15907723	15907924		CTATCCTGGGTAGGTAGGACCTATA		intergenic
177236407	177310917		GACAGGAATGCTACCCAGAACTGA		intergenic
135955605	135962939		GCAGTATTTGGC	27316	exon sense-overlappi
135955605	135962939		GCAGTATTTGGC	27316	exon sense-overlappi
50617571	50656114		CAGAGATACAGCTTCTTATAGTAAC		bidirectional

ENST00000507908	STX18	syntaxin 18 [Source:]	-	4426899	4544073
NM_152329	LRR1	leucine-rich repeat pı	+	50065414	50081390
NM_203467	LRR1	leucine-rich repeat pı	+	50065414	50081390
NM_001122770	ZBTB37	zinc finger and BTB	+	173837492	173855774
NM_032522	ZBTB37	zinc finger and BTB	+	173837492	173842778
NM_001103175	CCDC64B	bicaudal D-related pı	-	3077867	3085542
ENST00000483063	POLE4	polymerase (DNA-di	+	75185618	75197255
NM_019896	POLE4	DNA polymerase eps	+	75185774	75196859
ENST00000334574	FSD2	fibronectin type III aı	-	83424113	83474821
NM_001099695	REPIN1	replication initiator 1	+	150065878	150071133
NM_001099696	REPIN1	replication initiator 1	+	150065878	150071133
NM_013400	REPIN1	replication initiator 1	+	150065878	150071133
NM_014374	REPIN1	replication initiator 1	+	150068257	150071133
ENST00000563573	CDR2	cerebellar degenerati	-	22360692	22444986
ENST00000564542	CDR2	cerebellar degenerati	-	22358795	22442917
ENST00000567406	CDR2	cerebellar degenerati	-	22359075	22386411
NM_001206709	PRKAG1	5'-AMP-activated prc	-	49396054	49412629
NM_001206710	PRKAG1	5'-AMP-activated prc	-	49396054	49412531
NM_002733	PRKAG1	5'-AMP-activated prc	-	49396054	49412629
NM_003482	MLL2	histone-lysine N-met	-	49412757	49449107
NM_015086	DDN	dendrin	-	49388932	49393088
NM_001184728	PIGT	GPI transamidase coı	+	44044706	44054885
NM_001184729	PIGT	GPI transamidase coı	+	44044706	44054885
NM_001184730	PIGT	GPI transamidase coı	+	44044706	44054885
NM_015937	PIGT	GPI transamidase coı	+	44044706	44054885
NM_014904	RAB11FIP2	rab11 family-interact	-	119764426	119806114
NM_001202489	UBE2G2	ubiquitin-conjugating	-	46188494	46221751
NM_003343	UBE2G2	ubiquitin-conjugating	-	46188494	46221751
NM_182688	UBE2G2	ubiquitin-conjugating	-	46188494	46221751
NM_178868	CMTM8	CKLF-like MARVEI	+	32280170	32411813
NM_178491	R3HDML	peptidase inhibitor R	+	42965625	42979875
NM_001142301	TMEM67	Meckelin isoform 2	+	94767071	94830347
NM_153704	TMEM67	Meckelin isoform 1 p	+	94767071	94830347
NM_001257273	ZNF451	zinc finger protein 45	+	56954807	56974512
NM_001031623	ZNF451	zinc finger protein 45	+	56954827	57035098
NM_015555	ZNF451	zinc finger protein 45	+	56954827	57035098
NM_001164803	RBMX	RNA-binding motif p	-	135951352	135962939
NM_002139	RBMX	RNA-binding motif p	-	135955605	135962939
ENST00000378180	DLEU1	deleted in lymphocyt	+	50656306	50679433

30.180638	6.031173	4.527087	2.718497	37.913578	13.027849
100.682292	27.610373	6.167043	5.095483	141.71266	181.19125
19.96633	6.926253	3.990961	2.871147	24.199226	17.449657
44.16954	10.988113	5.086963	3.698561	38.865776	24.79957
949.278533	89.21627	8.773142	6.478911	1472.6993	128.44197
166.403098	61.847462	6.990346	5.955254	213.64847	217.20482
742.489177	202.480672	9.075136	7.910602	587.1631	627.8376
742.489177	202.480672	9.075136	7.910602	587.1631	627.8376
747.294217	179.550778	9.139845	7.810593	761.6602	803.93665
747.294217	179.550778	9.139845	7.810593	761.6602	803.93665
285.157073	61.628205	7.753319	6.2044	264.18216	188.66405
351.553933	100.70103	7.996338	6.946701	466.14822	557.10046
60.191781	15.132957	5.566352	4.120374	66.42745	66.84981
69.001243	12.990811	5.681787	3.878348	86.61041	64.11088
132.8721	34.905853	6.638831	5.396996	147.82242	200.66762
132.8721	34.905853	6.638831	5.396996	147.82242	200.66762
2093.033323	314.79383	10.474488	8.651587	2254.7102	3644.3713
4649.178433	1264.18391	11.660671	10.609607	3608.7717	4938.589
240.170155	67.004207	7.526584	6.346114	274.31116	218.32439
240.170155	67.004207	7.526584	6.346114	274.31116	218.32439
240.170155	67.004207	7.526584	6.346114	274.31116	218.32439
240.170155	67.004207	7.526584	6.346114	274.31116	218.32439
672.419884	33.794902	7.313934	4.892874	136.8477	209.07147
26.024581	6.882567	4.149061	2.906996	38.331066	10.555879
820.214605	227.888507	9.249365	8.133563	822.94073	1038.4177
24.389535	6.715916	4.162711	2.72398	50.53801	23.614916
24.389535	6.715916	4.162711	2.72398	50.53801	23.614916
24.389535	6.715916	4.162711	2.72398	50.53801	23.614916
92.664134	30.672569	6.163441	5.14892	104.04867	120.41079
866.930508	185.07115	9.357194	7.87495	847.7125	657.6028
597.986425	183.799252	8.796334	7.792044	605.0208	495.4487
24.374645	7.65744	4.243622	3.052536	28.644432	31.052261
24.374645	7.65744	4.243622	3.052536	28.644432	31.052261
24.374645	7.65744	4.243622	3.052536	28.644432	31.052261
24.374645	7.65744	4.243622	3.052536	28.644432	31.052261
24.374645	7.65744	4.243622	3.052536	28.644432	31.052261
231.222397	46.843199	7.463499	5.730914	284.7869	291.10046
231.222397	46.843199	7.463499	5.730914	284.7869	291.10046
231.222397	46.843199	7.463499	5.730914	284.7869	291.10046
231.222397	46.843199	7.463499	5.730914	284.7869	291.10046
29.339801	5.720599	4.47398	2.599471	38.615795	24.096125
71.791815	11.828926	4.868718	3.540815	117.07822	236.3781
28.098253	12.429889	4.371566	3.223955	18.904844	9.479187
608.74877	83.883464	8.690391	6.509606	460.9541	578.5543
38.432153	7.90762	4.83453	3.181189	42.633415	58.540306
38.432153	7.90762	4.83453	3.181189	42.633415	58.540306
38.432153	7.90762	4.83453	3.181189	42.633415	58.540306
722.464765	208.744442	9.066992	7.952874	753.9753	491.60336
96.77571	18.596141	6.111471	4.148085	145.0085	92.08457
472.101097	126.943156	8.344559	7.328788	564.9059	898.8594
3574.642433	369.231105	11.324755	8.7056	3981.1594	4407.7344
308.532148	54.605703	7.757131	6.091023	208.84735	181.71819
308.532148	54.605703	7.757131	6.091023	208.84735	181.71819
194.07315	47.2598	7.101414	5.878275	243.06213	124.43907
194.07315	47.2598	7.101414	5.878275	243.06213	124.43907
194.07315	47.2598	7.101414	5.878275	243.06213	124.43907
83.261372	22.815149	5.972907	4.074391	80.5054	97.807594
592.28537	89.260489	8.754232	6.809439	719.2972	886.72394
906.187972	257.615658	9.370362	8.357055	784.7938	1003.23553
387.283195	108.07246	8.209409	7.027299	409.1646	413.0881
387.283195	108.07246	8.209409	7.027299	409.1646	413.0881
1029.733025	147.899659	9.364959	7.440891	1580.778	1200.1613

37.44557	41.817738	22.03727	28.841824	5.2477345	4.9999995
69.224266	94.315	49.26294	68.387634	25.211477	28.288355
25.258509	22.1652	12.820798	17.90459	4.9999995	4.9999995
46.1811	42.506756	47.138145	65.525894	15.380679	7.227844
2554.5718	862.025	140.84023	537.0929	129.0742	70.58612
168.28502	141.79005	119.359726	138.1305	74.30176	23.592213
504.3705	1227.8982	563.2071	944.45856	154.6595	112.5504
504.3705	1227.8982	563.2071	944.45856	154.6595	112.5504
711.5024	896.8989	504.95898	804.80817	151.70259	107.789566
711.5024	896.8989	504.95898	804.80817	151.70259	107.789566
270.94043	375.43756	237.13072	374.58752	57.69145	30.661633
335.9288	294.79837	185.1417	270.20605	105.543785	124.94121
54.378593	57.644768	50.632927	65.21714	14.588209	7.2879224
106.426216	35.293224	62.127598	59.43913	17.107908	19.446291
79.516975	108.584496	108.02828	152.61281	27.857742	30.898499
79.516975	108.584496	108.02828	152.61281	27.857742	30.898499
961.66364	1080.527	2367.5308	2249.397	303.33258	269.85583
2107.4067	6079.5825	4321.8022	6838.9185	1356.566	748.1012
320.6174	235.86894	169.1151	222.78394	64.56095	37.01028
320.6174	235.86894	169.1151	222.78394	64.56095	37.01028
320.6174	235.86894	169.1151	222.78394	64.56095	37.01028
320.6174	235.86894	169.1151	222.78394	64.56095	37.01028
52.683865	2488.583	15.5413685	1131.7919	40.44125	12.654268
56.80878	14.839899	18.565649	17.046211	8.948438	4.9999995
484.9108	658.6643	945.5024	970.8517	205.1776	117.02958
15.656108	24.694073	13.749121	18.08498	4.9999995	4.9999995
15.656108	24.694073	13.749121	18.08498	4.9999995	4.9999995
15.656108	24.694073	13.749121	18.08498	4.9999995	4.9999995
79.4304	68.64398	89.386826	94.06414	26.899837	15.026337
995.5761	730.44867	961.99994	1008.24304	199.6254	238.53978
671.1199	798.12585	360.1644	658.0389	174.0722	81.6239
20.074385	33.536835	13.89647	19.04349	6.6936774	4.9999995
20.074385	33.536835	13.89647	19.04349	6.6936774	4.9999995
20.074385	33.536835	13.89647	19.04349	6.6936774	4.9999995
20.074385	33.536835	13.89647	19.04349	6.6936774	4.9999995
20.074385	33.536835	13.89647	19.04349	6.6936774	4.9999995
248.02568	174.81407	172.17354	216.43373	50.516502	22.602594
248.02568	174.81407	172.17354	216.43373	50.516502	22.602594
248.02568	174.81407	172.17354	216.43373	50.516502	22.602594
248.02568	174.81407	172.17354	216.43373	50.516502	22.602594
48.28	22.45579	16.723171	25.867924	5.851781	4.9999995
10.11711	14.53135	22.909548	29.736563	21.439781	22.131569
31.106272	42.024685	27.220642	39.853886	4.9999995	4.9999995
220.06137	414.32968	1019.16077	959.4324	94.14952	35.53095
19.029182	27.2638	37.63786	45.488354	5.9994035	5.334133
19.029182	27.2638	37.63786	45.488354	5.9994035	5.334133
19.029182	27.2638	37.63786	45.488354	5.9994035	5.334133
1037.5477	558.63495	699.50824	793.51904	189.49252	112.24147
163.01875	73.20155	44.215088	63.1258	17.69775	4.9999995
204.14285	472.10413	276.82697	415.76733	132.02429	161.3283
3083.2634	3892.4253	1909.5201	4173.752	282.041	278.79047
233.65584	562.64484	194.57657	469.7501	51.547615	45.59426
233.65584	562.64484	194.57657	469.7501	51.547615	45.59426
359.0329	209.31873	87.69421	140.89186	46.75512	40.08135
359.0329	209.31873	87.69421	140.89186	46.75512	40.08135
359.0329	209.31873	87.69421	140.89186	46.75512	40.08135
64.101364	126.53542	43.81032	86.808136	37.272	6.8270392
564.30945	542.9899	314.49518	525.89655	99.663956	99.21933
517.0586	1357.4551	738.6805	1035.9043	245.42752	214.14044
377.30353	433.23544	277.06586	413.84164	130.19997	95.513824
377.30353	433.23544	277.06586	413.84164	130.19997	95.513824
1876.4532	331.76517	582.93494	606.30554	180.9771	96.18286

6.2105436	8.180477	4.9999995	6.5482845	4.78003	3.7461262
25.048714	31.156021	33.641014	22.316658	6.6963983	7.2598467
4.9999995	8.984909	7.950448	9.622162	4.14022	4.1271
11.81045	14.14843	8.797504	8.56377	4.816172	4.5872583
212.98692	48.024227	38.862076	35.76408	10.057912	6.77469
156.05122	37.216873	49.87213	30.050577	7.289172	7.5055323
197.64062	148.13876	383.79935	218.0954	8.723602	8.976759
197.64062	148.13876	383.79935	218.0954	8.723602	8.976759
231.04457	195.1392	208.8011	182.82764	9.109694	9.322119
231.04457	195.1392	208.8011	182.82764	9.109694	9.322119
69.37803	50.934856	92.63337	68.469894	7.5907454	7.3188386
57.70037	146.5902	72.651276	96.77934	8.38964	8.811726
20.391397	12.01023	21.903612	14.616371	5.594424	5.888313
4.9999995	12.993561	10.192538	13.204571	5.981959	5.832124
18.49834	52.9675	37.747665	41.46537	6.751892	7.398277
18.49834	52.9675	37.747665	41.46537	6.751892	7.398277
327.3382	240.42647	448.50137	299.30853	10.667007	11.409957
2172.0818	872.09186	1499.5282	936.7344	11.335681	11.808327
97.41837	74.85284	74.746086	53.436714	7.6442246	7.512378
97.41837	74.85284	74.746086	53.436714	7.6442246	7.512378
97.41837	74.85284	74.746086	53.436714	7.6442246	7.512378
97.41837	74.85284	74.746086	53.436714	7.6442246	7.512378
72.57385	47.40894	4.9999995	24.691103	6.6422896	7.4532213
4.9999995	9.385622	5.350071	7.6112733	4.794468	3.4709065
314.41556	206.72716	300.82556	223.15558	9.223209	9.675767
13.368468	4.9999995	4.9999995	6.927028	5.194806	4.5190167
13.368468	4.9999995	4.9999995	6.927028	5.194806	4.5190167
13.368468	4.9999995	4.9999995	6.927028	5.194806	4.5190167
52.80799	34.06612	31.982195	23.252935	6.2545114	6.684485
165.89948	180.6895	182.5149	143.15784	9.264433	9.038364
276.47345	174.9783	233.00618	162.64148	8.766468	8.641002
12.192518	8.743812	7.0638566	6.2507763	4.3874264	4.88963
12.192518	8.743812	7.0638566	6.2507763	4.3874264	4.88963
12.192518	8.743812	7.0638566	6.2507763	4.3874264	4.88963
12.192518	8.743812	7.0638566	6.2507763	4.3874264	4.88963
12.192518	8.743812	7.0638566	6.2507763	4.3874264	4.88963
80.9807	28.778435	61.705578	36.475388	7.6971235	7.915071
80.9807	28.778435	61.705578	36.475388	7.6971235	7.915071
80.9807	28.778435	61.705578	36.475388	7.6971235	7.915071
80.9807	28.778435	61.705578	36.475388	7.6971235	7.915071
4.9999995	4.9999995	8.471814	4.9999995	4.8059683	4.548462
4.9999995	10.03124	4.9999995	7.370968	6.419948	7.623695
4.9999995	37.989017	4.9999995	16.59032	3.7792497	3.3330002
153.74536	37.399002	116.37451	66.10144	8.371012	8.86678
6.7106147	5.712141	14.61625	9.073176	4.95292	5.711576
6.7106147	5.712141	14.61625	9.073176	4.95292	5.711576
6.7106147	5.712141	14.61625	9.073176	4.95292	5.711576
266.62585	131.11887	358.83453	194.15341	9.091652	8.628077
24.954641	8.642542	29.946371	25.33554	6.726115	6.3167596
117.8438	127.164734	117.46209	105.83572	8.662384	9.477921
118.86003	339.65753	745.8348	450.2028	11.468857	11.669477
47.126476	82.49367	47.446323	53.425873	7.252865	7.263584
47.126476	82.49367	47.446323	53.425873	7.252865	7.263584
62.6506	46.19835	48.257282	39.616096	7.4756866	6.7326884
62.6506	46.19835	48.257282	39.616096	7.4756866	6.7326884
62.6506	46.19835	48.257282	39.616096	7.4756866	6.7326884
68.39247	4.9999995	11.58622	7.8131623	5.877446	6.3958316
102.33332	75.64192	89.92814	68.77627	9.021271	9.454793
236.56224	218.51988	373.87607	257.1678	9.157509	9.624605
178.49126	93.17479	81.69885	69.356064	8.218085	8.384856
178.49126	93.17479	81.69885	69.356064	8.218085	8.384856
268.19077	96.540085	146.6946	98.81254	10.1589775	9.870806

4.9122868	4.9716067	4.3585105	4.393959	2.601026	2.4279919
5.8032274	6.2079754	5.44986	5.584952	4.8992343	5.6595592
4.3463387	3.9709456	3.6206162	3.7405472	2.3467326	2.4279919
5.206724	4.9966507	5.39038	5.524594	4.1742296	3.615275
10.967577	9.429362	6.918059	8.491252	7.243159	6.988554
7.0648217	6.821927	6.6765327	6.5840926	6.4416	5.3905077
8.625051	9.947439	8.8779745	9.29999	7.5152416	7.6636105
8.625051	9.947439	8.8779745	9.29999	7.5152416	7.6636105
9.12156	9.490204	8.722115	9.073381	7.483323	7.5978823
9.12156	9.490204	8.722115	9.073381	7.483323	7.5978823
7.747354	8.234578	7.6454926	7.9829063	6.0879364	5.776781
8.058647	7.8885503	7.3008018	7.5286636	6.9598274	7.811996
5.448364	5.4658356	5.4842143	5.516959	4.0965295	3.626508
6.416896	4.7061467	5.7644024	5.389193	4.3331227	5.10717
5.997186	6.4153295	6.54012	6.73018	5.0348816	5.787242
5.997186	6.4153295	6.54012	6.73018	5.0348816	5.787242
9.542218	9.755116	10.934139	10.538493	8.513052	8.94312
10.688578	12.228256	11.777889	12.125295	10.696188	10.398167
7.9880214	7.575602	7.1766357	7.262645	6.2485695	6.056382
7.9880214	7.575602	7.1766357	7.262645	6.2485695	6.056382
7.9880214	7.575602	7.1766357	7.262645	6.2485695	6.056382
7.9880214	7.575602	7.1766357	7.262645	6.2485695	6.056382
5.4006066	10.947975	3.8833416	9.556169	5.573363	4.4593306
5.514719	3.324197	4.1212645	3.6688137	3.3773136	2.4279919
8.571576	9.0523205	9.634483	9.338832	7.926648	7.720291
3.6470206	4.1457596	3.713131	3.7565346	2.3467326	2.4279919
3.6470206	4.1457596	3.713131	3.7565346	2.3467326	2.4279919
3.6470206	4.1457596	3.713131	3.7565346	2.3467326	2.4279919
5.9951396	5.735118	6.274384	6.037009	4.987628	4.7229214
9.588116	9.20575	9.6579075	9.388596	7.886544	8.747334
9.023521	9.325902	8.236465	8.784643	7.6957006	7.2025995
4.0059657	4.6258793	3.7291548	3.8236775	2.9379373	2.4279919
4.0059657	4.6258793	3.7291548	3.8236775	2.9379373	2.4279919
4.0059657	4.6258793	3.7291548	3.8236775	2.9379373	2.4279919
4.0059657	4.6258793	3.7291548	3.8236775	2.9379373	2.4279919
4.0059657	4.6258793	3.7291548	3.8236775	2.9379373	2.4279919
7.62342	7.1251006	7.1987567	7.2215233	5.895338	5.326412
7.62342	7.1251006	7.1987567	7.2215233	5.895338	5.326412
7.62342	7.1251006	7.1987567	7.2215233	5.895338	5.326412
7.62342	7.1251006	7.1987567	7.2215233	5.895338	5.326412
5.2766347	3.9930918	3.984094	4.235627	2.750073	2.4279919
3.025926	3.2920148	4.408493	4.442233	4.659763	5.296691
4.651634	4.9796686	4.642429	4.843414	2.3467326	2.4279919
7.460045	8.384178	9.736995	9.3233385	6.787743	5.993319
3.93022	4.307352	5.082461	5.02265	2.787523	3.1485484
3.93022	4.307352	5.082461	5.02265	2.787523	3.1485484
3.93022	4.307352	5.082461	5.02265	2.787523	3.1485484
9.643356	8.802414	9.18377	9.052686	7.8110924	7.6596923
7.0147543	5.83261	5.3056035	5.4729824	4.383585	2.4279919
7.3606114	8.568567	7.8684397	8.129433	7.272711	8.181859
11.206703	11.577353	10.601215	11.424923	8.399102	8.989393
7.544601	8.814109	7.367026	8.300599	5.923726	6.357631
7.544601	8.814109	7.367026	8.300599	5.923726	6.357631
8.14843	7.394985	6.248054	6.6086392	5.7760777	6.1684523
8.14843	7.394985	6.248054	6.6086392	5.7760777	6.1684523
8.14843	7.394985	6.248054	6.6086392	5.7760777	6.1684523
5.697238	6.6525836	5.294198	5.920142	5.4566307	3.531699
8.779269	8.761826	8.046786	8.461447	6.8743606	7.4888945
8.655772	10.0853	9.265468	9.433519	8.198083	8.601015
8.210738	8.449653	7.8697543	8.123365	7.255137	7.4352374
8.210738	8.449653	7.8697543	8.123365	7.255137	7.4352374
10.515139	8.05764	8.925515	8.661674	7.750097	7.443655

2.8275633	3.120954	2.3618152	2.9716313
4.671764	5.173439	5.345134	4.8237696
2.350709	3.2634592	3.2852955	3.5526924
3.6515532	3.9508004	3.4252677	3.374238
7.6812725	5.8466845	5.549306	5.5644884
7.2348433	5.450528	5.914273	5.299771
7.5775166	7.583874	8.823786	8.299586
7.5775166	7.583874	8.823786	8.299586
7.7941194	8.005218	7.958147	8.024868
7.7941194	8.005218	7.958147	8.024868
6.0902853	5.9438987	6.785332	6.542165
5.8372808	7.5706005	6.447755	7.052748
4.391829	3.698442	4.72583	4.1831064
2.350709	3.819745	3.627902	4.0314417
4.2618074	6.0023475	5.5084543	5.787242
4.2618074	6.0023475	5.5084543	5.787242
8.305659	8.331963	9.050843	8.764882
11.049557	10.247843	10.799775	10.466114
6.5665956	6.5473337	6.4858212	6.1719832
6.5665956	6.5473337	6.4858212	6.1719832
6.5665956	6.5473337	6.4858212	6.1719832
6.5665956	6.5473337	6.4858212	6.1719832
6.155364	5.8267007	2.3618152	4.980672
2.350709	3.3292668	2.7627459	3.193948
8.24472	8.095094	8.479794	8.334832
3.8146052	2.3408759	2.3618152	3.0518618
3.8146052	2.3408759	2.3618152	3.0518618
3.8146052	2.3408759	2.3618152	3.0518618
5.712997	5.311313	5.27341	4.885249
7.323999	7.879078	7.7596226	7.6531234
8.057017	7.8287354	8.118135	7.850077
3.6954606	3.227034	3.1213126	2.9054773
3.6954606	3.227034	3.1213126	2.9054773
3.6954606	3.227034	3.1213126	2.9054773
3.6954606	3.227034	3.1213126	2.9054773
3.6954606	3.227034	3.1213126	2.9054773
6.3042245	5.054576	6.212938	5.591996
6.3042245	5.054576	6.212938	5.591996
6.3042245	5.054576	6.212938	5.591996
6.3042245	5.054576	6.212938	5.591996
2.350709	2.3408759	3.374424	2.352752
2.350709	3.4275374	2.3618152	3.1483753
2.350709	5.4808626	2.3618152	4.3756213
7.212807	5.457521	7.1151757	6.4910727
2.919931	2.6274114	4.1421285	3.4615943
2.919931	2.6274114	4.1421285	3.4615943
2.919931	2.6274114	4.1421285	3.4615943
8.004561	7.402501	8.724084	8.115315
4.6663866	3.207969	5.1810617	5.0215173
6.8427434	7.3579674	7.126451	7.1909933
6.8531103	8.854871	9.775311	9.3618145
5.5570536	6.6931663	5.843215	6.1713448
5.5570536	6.6931663	5.843215	6.1713448
5.9543104	5.787163	5.86562	5.718027
5.9543104	5.787163	5.86562	5.718027
5.9543104	5.787163	5.86562	5.718027
6.073433	2.3408759	3.8078172	3.2358902
6.6409817	6.5613384	6.7432756	6.5477853
7.832697	8.180323	8.783784	8.546425
7.4307237	6.8726234	6.607797	6.562273
7.4307237	6.8726234	6.607797	6.562273
8.013227	6.926316	7.4249196	7.087134

ASHGA5P046122	0.035157768	0.118717479	3.9074078	up	noncoding
ASHGA5P043504	0.003698442	0.038992846	2.3718888	up	noncoding
ASHGA5P029970	0.021328725	0.090021677	2.7013902	up	noncoding
ASHGA5P029970	0.021328725	0.090021677	2.7013902	up	noncoding
ASHGA5P031283	0.001967009	0.029367232	2.6990893	up	noncoding
ASHGA5P030926	0.000108423	0.009526401	3.4937056	up	noncoding
ASHGA5P034780	0.004072664	0.040758638	3.9066153	up	noncoding
ASHGA5P017257	8.26233E-06	0.004010044	4.4279784	up	noncoding
ASHGA5P029990	0.048387093	0.142801738	2.3833245	up	noncoding
ASHGA5P025808	0.01193286	0.066970246	2.0440184	up	noncoding
ASHGA5P017497	0.000616712	0.016837013	2.3318803	up	noncoding
ASHGA5P053591	0.015561993	0.076285362	2.8930365	up	noncoding
ASHGA5P044042	0.000884634	0.020090144	4.444959	up	noncoding
ASHGA5P027841	0.0111663	0.065174842	3.0120646	up	noncoding
ASHGA5P057475	0.011354167	0.065642623	2.4252162	up	noncoding
ASHGA5P026144	0.002563643	0.032817311	2.7087193	up	noncoding
ASHGA5P035114	0.003059513	0.035781836	2.9647347	up	noncoding
ASHGA5P035114	0.003059513	0.035781836	2.9647347	up	noncoding
ASHGA5P019112	0.001118408	0.022608782	2.0382746	up	noncoding
ASHGA5P043298	0.011569438	0.066242493	3.7997264	up	noncoding
ASHGA5P040755	0.001119796	0.022608782	2.0992611	up	noncoding
ASHGA5P039623	0.007122474	0.053286323	2.4395819	up	noncoding
ASHGA5P044100	0.009073021	0.059385427	2.5310073	up	noncoding
ASHGA5P039818	0.010023256	0.062088421	2.9846781	up	noncoding
ASHGA5P020826	0.002449955	0.032178734	2.3862461	up	noncoding
ASHGA5P029188	0.003666905	0.038875464	4.8658907	up	noncoding
ASHGA5P029188	0.003666905	0.038875464	4.8658907	up	noncoding
ASHGA5P029188	0.003666905	0.038875464	4.8658907	up	noncoding
ASHGA5P018181	0.0221686	0.092018296	2.0262877	up	noncoding
ASHGA5P041671	0.002305849	0.031550941	2.4462539	up	noncoding
ASHGA5P041671	0.002305849	0.031550941	2.4462539	up	noncoding
ASHGA5P041671	0.002305849	0.031550941	2.4462539	up	noncoding
ASHGA5P017358	0.004780397	0.043918141	2.1125441	up	noncoding
ASHGA5P039455	0.00518833	0.045795413	4.0895781	up	noncoding
ASHGA5P042993	0.006256903	0.05052414	2.2747275	up	noncoding
ASHGA5P042993	0.006256903	0.05052414	2.2747275	up	noncoding
ASHGA5P044369	0.000562753	0.016101946	6.7100431	up	noncoding
ASHGA5P044369	0.000562753	0.016101946	6.7100431	up	noncoding
ASHGA5P055433	0.00089529	0.020125432	3.5277274	up	noncoding
ASHGA5P055433	0.00089529	0.020125432	3.5277274	up	noncoding
ASHGA5P047408	0.005288265	0.046247034	3.0870764	up	noncoding
ASHGA5P057660	0.00568276	0.047888459	4.2115915	up	noncoding
ASHGA5P018706	0.009150426	0.059552152	2.327906	up	noncoding
ASHGA5P022237	0.01183526	0.066924702	2.1456003	up	noncoding
ASHGA5P051277	3.77096E-05	0.006301164	2.6329187	up	noncoding
ASHGA5P047938	0.013825567	0.07198363	4.984185	up	noncoding
ASHGA5P018221	0.002909529	0.034948668	2.0056401	up	noncoding
ASHGA5P018221	0.002909529	0.034948668	2.0056401	up	noncoding
ASHGA5P018221	0.002909529	0.034948668	2.0056401	up	noncoding
ASHGA5P028433	0.00019312	0.01146874	7.1270194	up	noncoding
ASHGA5P017505	0.005345941	0.046545718	2.1724501	up	noncoding
ASHGA5P033101	0.000549101	0.016081868	4.1512591	up	noncoding
ASHGA5P032629	0.001015393	0.021591454	4.042618	up	noncoding
ASHGA5P054755	0.004366692	0.041920723	4.2854242	up	noncoding
ASHGA5P026326	0.000199299	0.01146874	3.2965763	up	noncoding
ASHGA5P046584	0.005394932	0.046690443	3.0307657	up	noncoding
ASHGA5P056182	0.034734991	0.117910957	2.0583952	up	noncoding
ASHGA5P037181	5.2942E-06	0.003272762	3.369726	up	noncoding
ASHGA5P037181	5.2942E-06	0.003272762	3.369726	up	noncoding
ASHGA5P037181	5.2942E-06	0.003272762	3.369726	up	noncoding
ASHGA5P037181	5.2942E-06	0.003272762	3.369726	up	noncoding
ASHGA5P037181	5.2942E-06	0.003272762	3.369726	up	noncoding

ENST00000435789	RP11-431J24.2	GENCODE	1037	chrX	-
ENST00000431453	PMS2CL	GENCODE	792	chr7	+
ENST00000455788	RP11-263K19.6	GENCODE	568	chr1	-
ENST00000455788	RP11-263K19.6	GENCODE	568	chr1	-
ENST00000561611	AC004158.2	GENCODE	2194	chr16	-
ENST00000574705	RP11-295D4.1	GENCODE	1949	chr16	-
TCONS_00003548	XLOC_001320	LincRNAs identified	740	chr2	+
ENST00000439407	AC087859.1	GENCODE	576	chr3	-
TCONS_00016231	XLOC_007614	LincRNAs identified	2264	chr9	+
NR_038205	LOC100616530	RefSeq	219	chr8	+
ENST00000442370	RP11-107C16.2	GENCODE	536	chr10	+
ENST00000524335	RP13-582O9.5	GENCODE	499	chr8	-
NR_038835	LOC645249	RefSeq	992	chr7	+
NR_038920	LOC100506314	RefSeq	1743	chr12	+
TCONS_00018162	XLOC_008454	LincRNAs identified	532	chr10	+
NR_047550	LOC100506082	RefSeq	463	chr11	+
ENST00000413452	DGUOK-AS1	GENCODE	419	chr2	-
ENST00000413452	DGUOK-AS1	GENCODE	419	chr2	-
NR_023353	EXOSC7	RefSeq	1246	chr3	+
ENST00000456270	AC000111.6	GENCODE	376	chr7	-
ENST00000456826	RP11-403I13.6	pseudogene	2544	chr1	+
ENST00000561977	RP11-308D13.3	GENCODE	1098	chr4	-
ENST00000521218	CTA-398F10.2	GENCODE	510	chr8	-
ENST00000413231	RP11-389O22.1	GENCODE	377	chr1	+
ENST00000520422	RP11-150O12.1	GENCODE	734	chr8	-
NR_046320	GPX2	RefSeq	1311	chr14	-
NR_046320	GPX2	RefSeq	1311	chr14	-
NR_046320	GPX2	RefSeq	1311	chr14	-
ENST00000450299	LINC00238	GENCODE	722	chr14	+
ENST00000566170	RP1-223E5.4	GENCODE	1045	chr6	-
ENST00000566170	RP1-223E5.4	GENCODE	1045	chr6	-
ENST00000566170	RP1-223E5.4	GENCODE	1045	chr6	-
ENST00000440568	AC034193.5	GENCODE	558	chr3	+
ENST00000567197	RP11-44F21.5	GENCODE	571	chr4	-
ENST00000358772	NPSR1-AS1	GENCODE	550	chr7	-
ENST00000358772	NPSR1-AS1	GENCODE	550	chr7	-
ENST00000562577	RP11-219B4.7	GENCODE	487	chr8	-
ENST00000562577	RP11-219B4.7	GENCODE	487	chr8	-
ENST00000435388	RP4-635E18.6	GENCODE	440	chr1	+
ENST00000435388	RP4-635E18.6	GENCODE	440	chr1	+
ENST00000423283	RP11-123B3.2	GENCODE	435	chr10	+
TCONS_00020502	XLOC_009823	LincRNAs identified	285	chr12	+
ENST00000456633	RP11-29H23.1	GENCODE	911	chr1	+
ENST00000553181	RP11-320M2.1	GENCODE	1737	chr2	+
ENST00000428858	TTC28-AS1	GENCODE	565	chr22	+
ENST00000533002	CTC-497E21.3	GENCODE	670	chr11	-
NR_047580	TKT	RefSeq	2069	chr3	-
NR_047580	TKT	RefSeq	2069	chr3	-
NR_047580	TKT	RefSeq	2069	chr3	-
ENST00000449656	C1QTNF9-AS1	GENCODE	470	chr13	-
ENST00000442440	RP3-322G13.5	GENCODE	514	chr20	-
ENST00000436334	RP5-1061H20.4	GENCODE	315	chr1	-
ENST00000532249	AC005152.2	GENCODE	577	chr17	-
TCONS_00007987	XLOC_003455	LincRNAs identified	375	chr4	+
TCONS_00014250	XLOC_006350	LincRNAs identified	867	chr7	-
ENST00000444482	RP13-216E22.4	GENCODE	451	chrX	+
TCONS_00000823	XLOC_000032	LincRNAs identified	624	chr1	+
NR_045370	LOC100505815	RefSeq	4760	chr20	+
NR_045370	LOC100505815	RefSeq	4760	chr20	+
NR_045370	LOC100505815	RefSeq	4760	chr20	+
NR_045370	LOC100505815	RefSeq	4760	chr20	+
NR_045370	LOC100505815	RefSeq	4760	chr20	+

16171342	16188992		ATATACTTGTTGAGGAGGAACTGCA		natural antisense
6777265	6786736		ATTTTCACCGGA	441194	intergenic
155180941	155183610		TTGGGAAGTTAGAAATGAGGAAGA		natural antisense
155180941	155183610		TTGGGAAGTTAGAAATGAGGAAGA		natural antisense
72459383	72698903		CGATAGAAGTCGACGTGGCATTGCC		intergenic
4385870	4387819		GAAAAAACATACACATCAGCTCCTT		natural antisense
2875155	2876517		ACCTACATAATCTGCCTACTATCAG		intergenic
8540294	8653610		ATGGTGTGTTGGTGCCAACCCACTT		intronic antisense
139505191	139508134		TTTGAGGTCAAGAGTTTGAGACCTT		intergenic
96281063	96822371		ATTATGCGGTAA	100616530	intronic antisense
124578331	124583027		GTGTGCGCAGGTCTGGCTGACTCTC		intergenic
144363239	144363835		CTCTTTTCAGCTCCTTCGGTAGTCA		intergenic
156803550	156809118		ATGTGTTTTGGA	645249	bidirectional
13132770	13137576		GAATGTAAAGTT	100506314	intronic antisense
37668109	37669239		AATTGACTTTGGAACTGTCTTTGTC		intergenic
4208381	4223883		GTAGCTTCTGCT	100506082	intergenic
74185142	74208488	ENST00000439192	CCAAGTCTTACTCAGAGAACAGATC		natural antisense
74185142	74208488	ENST00000439192	CCAAGTCTTACTCAGAGAACAGATC		natural antisense
45017740	45054158		TGGCGTCCAGTT	23016	exon sense-overlappi
117244844	117287469		CATACTGGAATGGAATTAAAGACCC		intronic antisense
149261185	149263729	NM_144963	GTGGAGTTCCACATCCGGCACAACTACCCCTGGAACAA		
140539289	140544386		TGAGGAATACGGTCGTGTCTGTTAA		intergenic
8314418	8318847		AGGTTGAATTTCCTCTTGGTGGGGA		intergenic
113550875	113589677		ATGCTTCAGACAACTCTCGCAAACT		intergenic
37278921	37351352		ATTTTGCCTGACAGGAGACAGAGGC		intergenic
65405869	65409623		AACTGCTCAGGG	2877	exon sense-overlappi
65405869	65409623		AACTGCTCAGGG	2877	intronic antisense
65405869	65409623		AACTGCTCAGGG	2877	intronic antisense
66953124	66965118		TTCAGGATCTTA	440184	intergenic
13614342	13615387		CAAATGTCTGTGTAGTTTCTGCACT		natural antisense
13614342	13615387		CAAATGTCTGTGTAGTTTCTGCACT		natural antisense
13614342	13615387		CAAATGTCTGTGTAGTTTCTGCACT		natural antisense
10028898	10044781		AACACCATAGCTAATATTAACTTTC		bidirectional
76007134	76007705	ENST00000561705	TATGTGTCTATCTTTCCTGCTGTTCA		intergenic
34607863	34797884		CTACGTAGACCA	404744	intronic antisense
34607863	34797884		CTACGTAGACCA	404744	intronic antisense
86084311	86089276		GCCTAAGAAAACGGAATCTAAAAG		bidirectional
86084311	86089276		GCCTAAGAAAACGGAATCTAAAAG		bidirectional
11159731	11162157		GCCCTTAATACAATTCATTCCACCC		natural antisense
11159731	11162157		GCCCTTAATACAATTCATTCCACCC		natural antisense
50627322	50680949		CCAGAACATCAAAGGACAGTCAAA		intronic antisense
88811914	88813084		ATTCTACAGGTTACCAGTACACACC		intergenic
155531832	155533437		CGGCCAGCGGGGGCAGGAGAGGAA		natural antisense
10588819	10591453		CAGTAACTAGGTCTGAGGCACAGTC		bidirectional
28315459	28318075		ACGCTTTCCGGT	284900	natural antisense
13022636	13030706		TGGCATGTCATTTGACCCAATGGCT		bidirectional
53259652	53290130		AATACTTCGACA	7086	exon sense-overlappi
53259652	53290130		AATACTTCGACA	7086	exon sense-overlappi
53259652	53290130		AATACTTCGACA	7086	exon sense-overlappi
24889862	24895736		TTGTGATTGGAACCACTCTTCAAGG		natural antisense
23337230	23338642		TTTCTTGTTGTTCTGGGAATATCTGC		intergenic
229405112	229406775		CAGAGATCCCTGAAACCTGGACAAC		bidirectional
70137526	70216859		AGCGAATGCCTTTTGCCTCTTTGAT		intergenic
11315541	11339069	TCONS_00008754	ACAAGGTGGACCTAAAACTGGGTG		intergenic
7591940	7606158	TCONS_00013689,	CAGGAGTGAAGTAAAAAGACATGG		intergenic
73168807	73169393		GCCAACTGTACCGTTAGTGATTAGT		intergenic
4192616	4194185		TTTTATGTGGGGAAAGCAGCGAGGA		intergenic
62507483	62512243		CGAACCACAGAA	100505815	intron sense-overlapp
62507483	62512243		CGAACCACAGAA	100505815	intron sense-overlapp
62507483	62512243		CGAACCACAGAA	100505815	intron sense-overlapp
62507483	62512243		CGAACCACAGAA	100505815	intron sense-overlapp
62507483	62512243		CGAACCACAGAA	100505815	intron sense-overlapp

NM_005314	GRPR	gastrin-releasing pep	+	16141423	16171641
NM_002455	MTX1	metaxin-1 isoform 1	+	155178489	155183614
NM_198883	MTX1	metaxin-1 isoform 2	+	155178489	155183624
NM_032575	GLIS2	zinc finger protein G	+	4382224	4389598
NM_014583	LMCD1	LIM and cysteine-ric	+	8543510	8609806
NM_177965	C8orf37	protein C8orf37	-	96257140	96281462
NM_005515	MNX1	motor neuron and pa	-	156797546	156803347
NM_015987	HEBP1	heme-binding proteir	-	13127798	13153243
NM_080916	DGUOK	deoxyguanosine kina	+	74153952	74186088
NM_080918	DGUOK	deoxyguanosine kina	+	74153952	74186088
NM_015004	EXOSC7	exosome complex co	+	45017740	45052962
NM_000492	CFTR	cystic fibrosis transm	+	117120016	117308718
GTTGCCGGCCAACGTGAGGCAG					
NM_002083	GPX2	glutathione peroxida:	-	65405869	65409623
NM_001202558	CHURC1-FNTB	CHURC1-FNTB pro	+	65381078	65529373
NM_001202559	CHURC1-FNTB	CHURC1-FNTB pro	+	65381078	65529373
NM_001193267	SIRT5	NAD-dependent prot	+	13574760	13615390
NM_001242827	SIRT5	NAD-dependent prot	+	13574760	13615390
NM_012241	SIRT5	NAD-dependent prot	+	13574858	13615390
NM_018447	EMC3	ER membrane protei	-	10005635	10028522
NM_207172	NPSR1	neuropeptide S recep	+	34697896	34889590
NM_207173	NPSR1	neuropeptide S recep	+	34697896	34917944
NM_001083588	E2F5	transcription factor E	+	86089618	86126753
NM_001951	E2F5	transcription factor E	+	86089618	86126753
NM_001001998	EXOSC10	exosome component	-	11126675	11159938
NM_002685	EXOSC10	exosome component	-	11126675	11159938
NM_000124	ERCC6	DNA excision repair	-	50664490	50747147
NM_018489	ASH1L	histone-lysine N-met	-	155305051	155532324
NM_002539	ODC1	ornithine decarboxyl:	-	10580507	10588453
ENST00000415296	PITPNB	phosphatidylinositol	-	28256183	28316122
NM_001080521	RASSF10	ras association doma	+	13030969	13033653
NM_001064	TKT	transketolase isoforn	-	53259652	53290130
NM_001135055	TKT	transketolase isoforn	-	53258722	53290130
NM_001258028	TKT	transketolase isoforn	-	53259652	53290130
NM_178540	C1QTNF9	complement C1q and	+	24883715	24896669
NM_004578	RAB4A	ras-related protein R:	+	229406878	229440518
NM_001243891	TPD52L2	tumor protein D54 is	+	62496580	62522898
NM_001243892	TPD52L2	tumor protein D54 is	+	62496580	62522898
NM_001243894	TPD52L2	tumor protein D54 is	+	62496580	62522898
NM_001243895	TPD52L2	tumor protein D54 is	+	62496580	62522898
NM_003288	TPD52L2	tumor protein D54 is	+	62496580	62522898

224.410548	18.714125	6.469089	4.502877	405.29977	72.511154
28.193335	8.706689	4.441243	3.195206	19.5845	14.414917
35.77559	9.695261	4.764953	3.331251	48.44392	52.278923
35.77559	9.695261	4.764953	3.331251	48.44392	52.278923
47.819005	10.802534	5.118787	3.686315	63.147816	84.39003
494.030928	81.195784	8.490145	6.685387	575.66376	753.43176
219.971637	36.368158	7.245707	5.279788	282.81372	350.2592
29.820345	5.031636	4.555215	2.408567	35.804176	33.242344
656.47146	95.767181	8.06721	6.814235	193.93669	128.88011
53.868157	17.410876	5.365921	4.334513	78.486084	58.000217
107.297177	29.070173	6.353357	5.131863	153.93669	110.15648
55.196152	15.626888	5.447281	3.914696	57.562653	49.175644
213.477742	32.718718	7.354991	5.202821	254.90956	279.6683
827.466327	197.558677	9.272053	7.6813	790.5379	1000.6896
149.383597	35.468122	6.593824	5.31571	254.77454	300.53537
141.790966	29.827369	6.603026	5.165415	207.00325	205.1405
2194.6436	531.094662	10.471618	8.903715	1486.261	701.1679
2194.6436	531.094662	10.471618	8.903715	1486.261	701.1679
670.095865	194.296562	8.985973	7.958625	723.8433	850.3245
67.280394	12.1923	5.614003	3.688107	81.956055	62.795864
166.588952	53.385502	7.017075	5.947193	142.99081	131.54846
27.407439	8.155098	4.415255	3.128621	22.158855	20.553856
32.167218	9.167269	4.571472	3.23176	40.347366	42.770107
57.217832	10.29408	5.222687	3.645112	62.234818	78.471375
31.059394	9.137509	4.622089	3.367346	28.343227	25.32988
13997.0395	1630.301243	13.255764	10.97306	14724.919	13145.666
13997.0395	1630.301243	13.255764	10.97306	14724.919	13145.666
13997.0395	1630.301243	13.255764	10.97306	14724.919	13145.666
27.081245	9.831299	4.431519	3.41268	32.501476	32.300827
272.89448	71.977139	7.699559	6.408985	315.32077	227.83276
272.89448	71.977139	7.699559	6.408985	315.32077	227.83276
272.89448	71.977139	7.699559	6.408985	315.32077	227.83276
94.518496	27.114515	6.132276	5.053295	111.812584	151.20142
3248.8624	522.104922	11.169626	9.137674	3492.149	5194.646
23.50598	7.878123	4.225729	3.040035	29.324146	18.94343
23.50598	7.878123	4.225729	3.040035	29.324146	18.94343
378.036297	27.330168	7.804079	5.057757	174.90515	206.4599
378.036297	27.330168	7.804079	5.057757	174.90515	206.4599
1726.307258	250.1405	10.154198	8.335459	2363.0215	3303.1235
1726.307258	250.1405	10.154198	8.335459	2363.0215	3303.1235
72.879554	14.888315	5.71966	4.093419	87.57748	121.00561
323.559471	44.262251	7.61567	5.541304	521.5769	719.18976
560.722028	146.58485	8.632746	7.413713	729.57764	864.6731
41.326102	11.178664	4.858498	3.757116	58.41564	80.71276
17.017795	5	3.760142	2.363479	24.679884	15.523139
77.35575	7.502269	5.437907	3.120549	28.01744	17.873697
206.581	63.563934	7.25806	6.253997	258.79672	305.0099
206.581	63.563934	7.25806	6.253997	258.79672	305.0099
206.581	63.563934	7.25806	6.253997	258.79672	305.0099
94.230279	7.205509	5.680456	2.847157	40.712482	62.62909
577.15631	175.096453	8.767541	7.648218	662.8118	724.0333
30.829333	5.652032	4.597132	2.543583	31.747114	25.824442
47.686432	8.699577	5.208087	3.192797	61.710922	57.20234
65.826436	7.536389	5.140655	3.041217	87.728386	156.53189
52.570843	10.369033	5.291147	3.570179	56.058	74.9288
153.887118	36.723259	6.799021	5.199339	161.48044	191.54488
22.201429	7.543642	4.09412	3.0526	34.916405	28.111052
400.742417	72.696413	8.184351	6.43172	430.2499	444.7889
400.742417	72.696413	8.184351	6.43172	430.2499	444.7889
400.742417	72.696413	8.184351	6.43172	430.2499	444.7889
400.742417	72.696413	8.184351	6.43172	430.2499	444.7889
400.742417	72.696413	8.184351	6.43172	430.2499	444.7889

724.54034	30.744865	58.459137	54.90802	23.631382	13.76804
30.519697	36.143982	31.052137	37.44478	8.732702	4.9999995
31.967863	23.894554	32.00571	26.06257	7.019572	4.9999995
31.967863	23.894554	32.00571	26.06257	7.019572	4.9999995
46.496433	28.14678	28.68025	36.05272	11.328181	8.632324
380.38605	553.4574	265.0424	436.2042	86.880066	80.516235
141.84186	72.17678	254.67097	218.06729	38.605446	21.09379
36.793083	25.706215	21.05784	26.318409	4.9999995	4.9999995
188.49463	2056.941	137.48363	1233.0927	67.77535	43.676197
45.33292	60.362186	29.018604	52.00893	15.171461	9.01969
129.08691	79.78407	93.87633	76.94258	35.1331	17.97315
62.473988	66.416145	40.21097	55.337513	13.191848	4.9999995
169.21004	200.98709	166.92786	209.1636	36.84542	13.486149
545.6623	721.4615	837.83466	1068.612	200.24472	68.70168
76.8786	58.308987	107.84958	97.954506	43.423595	27.747625
173.66774	41.925102	112.13123	110.877975	36.615395	40.633877
2291.343	1308.3093	3844.5344	3536.246	244.20398	56.83945
2291.343	1308.3093	3844.5344	3536.246	244.20398	56.83945
566.0336	622.6642	567.41223	690.29736	169.24208	181.805
102.93639	80.46949	22.72914	52.795425	13.17473	4.9999995
188.55557	163.3477	172.64417	200.447	61.975872	26.063673
22.402348	25.037779	38.087116	36.20468	10.77962	4.9999995
29.254765	13.440599	36.100594	31.089878	11.60772	4.9999995
16.87813	107.25412	19.571672	58.89688	10.11414	9.340063
24.55762	43.958443	27.963303	36.20389	9.6755085	6.6506395
15942.132	21402.07	5288.157	13479.293	1107.6469	819.63226
15942.132	21402.07	5288.157	13479.293	1107.6469	819.63226
15942.132	21402.07	5288.157	13479.293	1107.6469	819.63226
25.96548	25.132032	22.045582	24.542072	10.33629	4.9999995
385.59036	244.4679	199.04575	265.10934	60.626812	37.055874
385.59036	244.4679	199.04575	265.10934	60.626812	37.055874
385.59036	244.4679	199.04575	265.10934	60.626812	37.055874
60.865456	58.35736	86.7783	98.095856	29.42151	30.87654
1752.7296	3040.782	2637.6248	3375.243	357.88083	211.05673
29.257927	20.140158	22.860964	20.509258	4.9999995	4.9999995
29.257927	20.140158	22.860964	20.509258	4.9999995	4.9999995
150.49329	944.1477	176.21664	615.9951	18.234312	19.75026
150.49329	944.1477	176.21664	615.9951	18.234312	19.75026
1403.303	1494.6562	622.51025	1171.2291	253.09778	306.2097
1403.303	1494.6562	622.51025	1171.2291	253.09778	306.2097
30.596952	82.89364	48.51399	66.68965	18.967318	14.366621
171.20787	90.397705	255.54604	183.43855	50.047432	23.991608
437.5653	238.63551	628.896	464.98462	168.68893	80.6891
33.46148	29.234293	18.081305	28.051132	10.24387	10.23667
14.177432	18.446602	13.663301	15.616409	4.9999995	4.9999995
28.228422	184.41072	61.52484	144.07938	5.2988596	6.156163
165.22072	112.73953	212.0424	185.67673	85.64107	101.18311
165.22072	112.73953	212.0424	185.67673	85.64107	101.18311
165.22072	112.73953	212.0424	185.67673	85.64107	101.18311
21.166885	37.18969	221.32379	182.35974	4.9999995	4.9999995
540.03107	498.45187	612.1968	425.41302	210.39645	83.56151
25.38872	40.374405	20.589287	41.05203	4.9999995	4.9999995
54.71765	37.107216	34.76211	40.618355	12.39386	4.9999995
27.893738	74.78463	5.2497497	42.77022	7.787353	14.805231
32.62762	31.37764	53.97356	66.45944	13.96829	11.0948
116.75497	61.639576	205.43626	186.46658	50.68316	14.206709
18.23071	16.145931	17.741674	18.0628	6.4023476	4.9999995
470.94647	512.59265	160.49998	385.3766	82.78152	48.545845
470.94647	512.59265	160.49998	385.3766	82.78152	48.545845
470.94647	512.59265	160.49998	385.3766	82.78152	48.545845
470.94647	512.59265	160.49998	385.3766	82.78152	48.545845
470.94647	512.59265	160.49998	385.3766	82.78152	48.545845

23.186682	19.014866	16.5023	16.181479	8.20354	5.999069
13.8371105	7.4763765	11.68075	5.5131946	3.8323848	3.8736084
8.733802	7.636619	18.293512	11.48806	5.1391444	5.5713167
8.733802	7.636619	18.293512	11.48806	5.1391444	5.5713167
13.337009	9.405043	14.165818	7.9468293	5.5237694	6.2035284
65.983116	92.23682	84.28048	77.277985	8.692874	9.231252
82.78959	22.979628	34.23989	18.500603	7.6881914	8.168598
5.189821	4.9999995	4.9999995	4.9999995	4.699124	4.979194
121.50958	161.94904	77.18509	102.50783	7.1485634	6.779146
29.0317	16.280909	17.153448	17.808048	5.8398933	5.7017303
40.129772	25.05659	26.481054	29.647375	6.8089333	6.563367
27.599844	8.667647	24.526543	14.775448	5.3850904	5.4887266
53.417416	21.944452	42.633896	27.984974	7.5375977	7.8594213
355.86032	67.23519	332.20166	161.10849	9.166004	9.620287
60.97862	18.341656	42.314606	20.00263	7.5367417	7.95938
33.281425	21.406622	20.487957	26.538937	7.2395864	7.4290247
401.4107	483.35544	1333.1372	667.6212	10.069458	9.130616
401.4107	483.35544	1333.1372	667.6212	10.069458	9.130616
156.5175	250.11972	212.49332	195.60175	9.031186	9.393203
13.054968	9.3730955	15.53252	17.018486	5.904527	5.8058276
93.096016	36.61392	57.881523	44.68201	6.708371	6.8058624
11.94208	7.0806704	8.864149	5.26407	4.006923	4.3332253
8.380477	4.9999995	16.788248	8.22717	4.871534	5.3066463
7.4501367	9.847801	11.311388	13.700951	5.5040965	6.1057725
7.1660905	14.509058	4.9999995	11.82376	4.3731055	4.614922
1367.6798	1821.126	2703.9597	1961.7628	13.384729	13.243889
1367.6798	1821.126	2703.9597	1961.7628	13.384729	13.243889
1367.6798	1821.126	2703.9597	1961.7628	13.384729	13.243889
11.76082	9.244757	13.53467	9.111257	4.562217	4.9392123
98.18723	52.431618	112.2595	71.3018	7.8457923	7.574256
98.18723	52.431618	112.2595	71.3018	7.8457923	7.574256
98.18723	52.431618	112.2595	71.3018	7.8457923	7.574256
18.72677	35.30036	19.11723	29.244682	6.356434	7.0055604
496.8186	240.38507	1252.6914	573.7969	11.295942	11.877131
9.92397	6.3605723	12.52037	8.463828	4.4180727	4.2303934
9.92397	6.3605723	12.52037	8.463828	4.4180727	4.2303934
31.554806	33.993057	27.134193	33.314377	6.997226	7.4355407
31.554806	33.993057	27.134193	33.314377	6.997226	7.4355407
207.62425	287.76285	219.87837	226.27005	10.729164	11.268146
207.62425	287.76285	219.87837	226.27005	10.729164	11.268146
24.702229	12.72026	8.018811	10.554651	5.999274	6.6925745
101.83389	19.07109	40.83613	29.793356	8.542692	9.167778
281.0226	111.66202	141.18755	96.2589	9.0463	9.421204
12.82093	10.663641	8.137023	14.969851	5.4061484	6.1405816
4.9999995	4.9999995	4.9999995	4.9999995	4.167419	3.9763172
9.862616	4.9999995	10.35478	8.341195	4.3544574	4.1581364
47.13562	51.546883	52.2708	43.60612	7.559444	7.9811974
47.13562	51.546883	52.2708	43.60612	7.559444	7.9811974
47.13562	51.546883	52.2708	43.60612	7.559444	7.9811974
4.9999995	4.9999995	13.149168	10.08389	4.884555	5.801633
332.79996	102.77923	190.86716	130.17441	8.899571	9.178617
4.9999995	4.9999995	8.912193	4.9999995	4.529036	4.6421456
11.18578	6.565073	12.0226	5.0301485	5.491579	5.68291
4.9999995	6.476231	4.9999995	6.1495204	6.0032883	7.0526505
9.915331	4.9999995	14.312997	7.9227786	5.345576	6.040837
72.56951	13.611561	47.962173	21.306438	6.878362	7.33761
9.170167	10.94785	6.5098867	7.2315984	4.6652417	4.75725
113.574196	95.654915	43.03704	52.584965	8.28749	8.484675
113.574196	95.654915	43.03704	52.584965	8.28749	8.484675
113.574196	95.654915	43.03704	52.584965	8.28749	8.484675
113.574196	95.654915	43.03704	52.584965	8.28749	8.484675
113.574196	95.654915	43.03704	52.584965	8.28749	8.484675

9.149382	4.494861	5.6811395	5.2865415	4.8035316	4.5876474
4.6234026	4.7426004	4.815528	4.759932	3.3368583	2.4279919
4.686373	4.0907717	4.855031	4.247084	3.0058832	2.4279919
4.686373	4.0907717	4.855031	4.247084	3.0058832	2.4279919
5.219002	4.3507433	4.708958	4.7067227	3.7321603	3.8833416
8.22118	8.78719	7.807702	8.200672	6.669672	7.1818714
6.825223	5.8098702	7.7514086	7.2309523	5.5034294	5.230659
4.8874345	4.2133555	4.29086	4.261322	2.3467326	2.4279919
7.2356753	10.6726	6.880534	9.686741	6.3180237	6.294335
5.1819935	5.5380483	4.720988	5.2128735	4.1586657	3.948143
6.6906977	5.9592047	6.34217	5.75577	5.369686	4.9888506
5.657337	5.683706	5.170342	5.298483	3.9486513	2.4279919
7.0719175	7.334071	7.1552453	7.1716957	5.4394574	4.555777
8.736874	9.186425	9.448238	9.47449	7.8915606	6.952528
5.9501505	5.4848337	6.5383945	6.0934415	5.6738076	5.629012
7.1107492	4.975722	6.5932446	6.2698274	5.429651	6.1886063
10.820469	10.03167	11.601778	11.1757145	8.190673	6.6834564
10.820469	10.03167	11.601778	11.1757145	8.190673	6.6834564
8.781971	8.96433	8.888732	8.856418	7.647561	8.3561
6.370512	5.971423	4.398897	5.23283	3.946541	2.4279919
7.236043	7.0294094	7.2034097	7.1193533	6.189556	5.5376835
4.1722894	4.168667	5.097692	4.7127304	3.655815	2.4279919
4.5630417	3.1595688	5.0227356	4.505304	3.7659097	2.4279919
3.7596726	6.3968415	4.1935434	5.376197	3.5624294	3.999057
4.308036	5.047921	4.67599	4.712561	3.4965892	3.4913578
13.67262	14.0679035	12.07122	13.094224	10.406976	10.538916
13.67262	14.0679035	12.07122	13.094224	10.406976	10.538916
13.67262	14.0679035	12.07122	13.094224	10.406976	10.538916
4.3889117	4.1755342	4.358796	4.164445	3.597268	2.4279919
8.240637	7.629021	7.4012246	7.506423	6.1569686	6.0584326
8.240637	7.629021	7.4012246	7.506423	6.1569686	6.0584326
8.240637	7.629021	7.4012246	7.506423	6.1569686	6.0584326
5.6189127	5.48584	6.231531	6.0953784	5.1105514	5.786395
10.415147	11.236628	11.088171	11.104737	8.745834	8.581947
4.563217	3.8130364	4.406117	3.9235346	2.3467326	2.4279919
4.563217	3.8130364	4.406117	3.9235346	2.3467326	2.4279919
6.9028945	9.563779	7.236231	8.688803	4.423615	5.132658
6.9028945	9.563779	7.236231	8.688803	4.423615	5.132658
10.083912	10.225321	9.014239	9.604408	8.242432	9.11972
10.083912	10.225321	9.014239	9.604408	8.242432	9.11972
4.6277194	6.018274	5.4287705	5.551348	4.4813766	4.656107
7.089847	6.145237	7.75637	6.992095	5.881146	5.417628
8.424522	7.5904217	9.029845	8.2841835	7.6422405	7.1871147
4.751691	4.412583	4.086128	4.3538537	3.5824375	4.1390724
3.5001023	3.6697216	3.702781	3.5445132	2.3467326	2.4279919
4.5099397	7.2052975	5.751811	6.6478004	2.6156101	3.3722532
7.0358076	6.4759145	7.486373	7.0096207	6.6484303	7.5114684
7.0358076	6.4759145	7.486373	7.0096207	6.6484303	7.5114684
7.0358076	6.4759145	7.486373	7.0096207	6.6484303	7.5114684
4.083895	4.7846975	7.543465	6.984489	2.3467326	2.4279919
8.721822	8.648746	8.995454	8.1610365	7.961089	7.238552
4.353895	4.9162817	4.26018	4.8812566	2.3467326	2.4279919
5.459435	4.781448	4.9656973	4.8674526	3.8606162	2.4279919
4.493106	5.86562	2.4909728	4.9382896	3.1578577	4.698577
4.7175193	4.526578	5.5708313	5.5455427	4.0298715	4.2634883
6.5487666	5.569736	7.4434056	7.0162473	5.900284	4.639372
3.8692296	3.458236	4.060231	3.7545311	2.876869	2.4279919
8.530447	8.683948	7.101765	8.01778	6.5971103	6.4474926
8.530447	8.683948	7.101765	8.01778	6.5971103	6.4474926
8.530447	8.683948	7.101765	8.01778	6.5971103	6.4474926
8.530447	8.683948	7.101765	8.01778	6.5971103	6.4474926
8.530447	8.683948	7.101765	8.01778	6.5971103	6.4474926

4.5665607	4.401739	4.320482	4.3373013
3.8642788	2.9922	3.821414	2.728495
3.249674	3.022371	4.4637537	3.8178346
3.249674	3.022371	4.4637537	3.8178346
3.8114226	3.3315463	4.0954337	3.2639832
6.0228376	6.858911	6.652111	6.7269187
6.3390436	4.699809	5.3725133	4.533274
2.6212332	2.3408759	2.3618152	2.352752
6.88399	7.71244	6.5330515	7.143567
4.880762	4.16768	4.372303	4.479524
5.324855	4.8353558	4.995413	5.27702
4.807457	3.214057	4.887112	4.2029085
5.7305045	4.623012	5.686862	5.181315
8.420762	6.37772	8.61003	7.835201
5.9159765	4.3450875	5.6756496	4.6547284
5.069063	4.5872583	4.6223826	5.0955276
8.604717	9.362572	10.638556	9.942313
8.604717	9.362572	10.638556	9.942313
7.240632	8.393701	7.9854956	8.12826
3.7850182	3.3268895	4.2288575	4.4133453
6.5059156	5.425444	6.119405	5.905155
3.6659305	2.9180255	3.4354823	2.6684783
3.1975226	2.3408759	4.3444376	3.3138227
3.0457366	3.4000354	3.7736626	4.0897503
2.9974213	3.994142	2.3618152	3.862753
10.360314	11.337718	11.654014	11.540424
10.360314	11.337718	11.654014	11.540424
10.360314	11.337718	11.654014	11.540424
3.6472712	3.3047054	4.0308647	3.4679809
6.57789	5.989274	7.065678	6.6056657
6.57789	5.989274	7.065678	6.6056657
6.57789	5.989274	7.065678	6.6056657
4.2763743	5.3719535	4.5226064	5.2518873
8.911175	8.331337	10.546904	9.708847
3.416931	2.7713077	3.9213226	3.355923
3.416931	2.7713077	3.9213226	3.355923
4.9949074	5.308905	5.0341306	5.452326
4.9949074	5.308905	5.0341306	5.452326
7.6490374	8.612217	8.034049	8.3553
7.6490374	8.612217	8.034049	8.3553
4.6500263	3.7856636	3.2971907	3.690149
6.633363	4.406572	5.624324	5.284793
8.079927	7.149965	7.378119	7.044913
3.7612078	3.5220854	3.3164263	4.2214684
2.350709	2.3408759	2.3618152	2.352752
3.4086986	2.3408759	3.6539404	3.3319185
5.557356	5.9611406	5.9781337	5.8674517
5.557356	5.9611406	5.9781337	5.8674517
5.557356	5.9611406	5.9781337	5.8674517
2.350709	2.3408759	3.992458	3.624174
8.32407	7.0281167	7.824687	7.5127935
2.350709	2.3408759	3.4424396	2.352752
3.5794473	2.8148434	3.8645585	2.6093252
2.350709	2.7978566	2.3618152	2.8804836
3.4159043	2.3408759	4.1116395	3.259294
6.1551523	3.8901327	5.856945	4.7541475
3.3167026	3.5627506	3.012346	3.1189392
6.7897825	6.9109945	5.699424	6.1455135
6.7897825	6.9109945	5.699424	6.1455135
6.7897825	6.9109945	5.699424	6.1455135
6.7897825	6.9109945	5.699424	6.1455135
6.7897825	6.9109945	5.699424	6.1455135

ASHGA5P037181	5.2942E-06	0.003272762	3.369726	up	noncoding
ASHGA5P037181	5.2942E-06	0.003272762	3.369726	up	noncoding
ASHGA5P037181	5.2942E-06	0.003272762	3.369726	up	noncoding
ASHGA5P037181	5.2942E-06	0.003272762	3.369726	up	noncoding
ASHGA5P037181	5.2942E-06	0.003272762	3.369726	up	noncoding
ASHGA5P047821	0.002357298	0.031728208	2.0297971	up	noncoding
ASHGA5P050700	0.00188882	0.029138666	3.2824478	up	noncoding
ASHGA5P050700	0.00188882	0.029138666	3.2824478	up	noncoding
ASHGA5P053101	0.006699177	0.051962277	2.2161077	up	noncoding
ASHGA5P041099	0.003774432	0.039427161	5.4719032	up	noncoding
ASHGA5P020987	0.02992857	0.108408349	3.5753139	up	noncoding
ASHGA5P032911	0.000289884	0.012512734	2.4743176	up	noncoding
ASHGA5P056005	0.022418518	0.092633596	12.5250546	up	noncoding
ASHGA5P000309	0.002596463	0.032877969	2.4705243	up	noncoding
ASHGA5P015365	0.04319768	0.133282412	3.3486109	up	noncoding
ASHGA5P015365	0.04319768	0.133282412	3.3486109	up	noncoding
ASHGA5P042645	0.005419142	0.04682972	3.3427323	up	noncoding
ASHGA5P042645	0.005419142	0.04682972	3.3427323	up	noncoding
ASHGA5P042645	0.005419142	0.04682972	3.3427323	up	noncoding
ASHGA5P026575	0.023501024	0.094945968	3.8754837	up	noncoding
ASHGA5P026575	0.023501024	0.094945968	3.8754837	up	noncoding
ASHGA5P050525	0.001161602	0.022899969	3.0924537	up	noncoding
ASHGA5P050525	0.001161602	0.022899969	3.0924537	up	noncoding
ASHGA5P047598	8.45255E-05	0.008738697	5.2341979	up	noncoding
ASHGA5P045003	0.013882292	0.072158735	2.2376496	up	noncoding
ASHGA5P050965	0.015357585	0.075812413	4.8143969	up	noncoding
ASHGA5P026953	0.018275781	0.082058492	2.316815	up	noncoding
ASHGA5P029372	0.033607852	0.115626776	2.0650459	up	noncoding
ASHGA5P044727	0.040453961	0.128716472	2.4585731	up	noncoding
ASHGA5P044727	0.040453961	0.128716472	2.4585731	up	noncoding
ASHGA5P016696	5.69828E-06	0.003401089	3.3186426	up	noncoding
ASHGA5P045019	0.029694843	0.107980682	2.4555828	up	noncoding
ASHGA5P043241	0.035039111	0.118455462	2.3699407	up	noncoding
ASHGA5P039338	0.023221869	0.09436573	2.1530731	up	noncoding
ASHGA5P032878	0.012119127	0.067450153	2.4207405	up	noncoding
ASHGA5P021682	9.26925E-05	0.009080764	2.5380518	up	noncoding
ASHGA5P021682	9.26925E-05	0.009080764	2.5380518	up	noncoding
ASHGA5P021682	9.26925E-05	0.009080764	2.5380518	up	noncoding
ASHGA5P048251	0.000667552	0.017209782	2.3723946	up	noncoding
ASHGA5P049223	0.001133569	0.022643498	20.1587172	up	noncoding
ASHGA5P031501	0.000222045	0.011576416	2.5645979	up	noncoding
ASHGA5P019869	5.76421E-05	0.007736798	2.4272047	up	noncoding
ASHGA5P019869	5.76421E-05	0.007736798	2.4272047	up	noncoding
ASHGA5P045023	0.000314439	0.012923818	7.3489004	up	noncoding
ASHGA5P040040	0.00567076	0.047810609	8.5614812	up	noncoding
ASHGA5P042658	0.004172482	0.041167338	2.7703821	up	noncoding
ASHGA5P029114	0.008461057	0.057522562	2.1496447	up	noncoding
ASHGA5P018460	0.000211894	0.01146874	3.3763589	up	noncoding
ASHGA5P041921	0.003792129	0.039487588	3.2492451	up	noncoding
ASHGA5P030958	0.003440593	0.037787575	2.8026426	up	noncoding
ASHGA5P023186	0.001550485	0.026466815	5.1086219	up	noncoding
ASHGA5P023186	0.001550485	0.026466815	5.1086219	up	noncoding
ASHGA5P023186	0.001550485	0.026466815	5.1086219	up	noncoding
ASHGA5P023186	0.001550485	0.026466815	5.1086219	up	noncoding
ASHGA5P023186	0.001550485	0.026466815	5.1086219	up	noncoding
ASHGA5P023186	0.001550485	0.026466815	5.1086219	up	noncoding
ASHGA5P023186	0.001550485	0.026466815	5.1086219	up	noncoding
ASHGA5P023186	0.001550485	0.026466815	5.1086219	up	noncoding
ASHGA5P014616	0.03759377	0.123077466	2.4817332	up	noncoding
ASHGA5P007912	0.028259276	0.104954123	2.4503068	up	noncoding
ASHGA5P039566	2.20734E-05	0.005043652	3.5896993	up	noncoding
ASHGA5P029655	0.027167078	0.102915644	2.132228	up	noncoding

NR_045370	LOC100505815	RefSeq	4760	chr20	+
NR_045370	LOC100505815	RefSeq	4760	chr20	+
NR_045370	LOC100505815	RefSeq	4760	chr20	+
NR_045370	LOC100505815	RefSeq	4760	chr20	+
NR_045370	LOC100505815	RefSeq	4760	chr20	+
ENST00000450713	RP11-656D10.3	GENCODE	605	chr1	-
uc002ugc.1	AK127400	UCSC_knowngene	2184	chr2	-
uc002ugc.1	AK127400	UCSC_knowngene	2184	chr2	-
ENST00000429320	GS1-124K5.2	GENCODE	498	chr7	-
ENST00000506435	RP11-506H20.1	GENCODE	770	chr5	+
ENST00000523087	RP3-399L15.3	GENCODE	493	chr6	+
ENST00000434303	AC090286.2	pseudogene	587	chr17	+
NR_026797	LINC00520	RefSeq	2030	chr14	-
chr18:18297450-183	chr18:18297450-183	LincRNAs identified	13175	chr18	-
ENST00000419644	RP11-69E9.1	GENCODE	480	chr1	-
ENST00000419644	RP11-69E9.1	GENCODE	480	chr1	-
ENST00000444302	RP3-388E23.2	GENCODE	577	chr6	+
ENST00000444302	RP3-388E23.2	GENCODE	577	chr6	+
ENST00000444302	RP3-388E23.2	GENCODE	577	chr6	+
ENST00000569428	RP11-554A11.6	GENCODE	532	chr11	+
ENST00000569428	RP11-554A11.6	GENCODE	532	chr11	+
ENST00000439192	DGUOK-AS1	GENCODE	563	chr2	-
ENST00000439192	DGUOK-AS1	GENCODE	563	chr2	-
ENST00000432120	RP11-119K6.6	GENCODE	645	chr10	+
ENST00000517482	RP11-383J24.1	GENCODE	553	chr8	+
ENST00000400465	FER1L4	GENCODE	3350	chr20	-
DB378707		LincRNAs identified	534	chr7	+
ENST00000554954	RP11-982M15.7	GENCODE	486	chr14	-
ENST00000523935	RP11-181B11.2	GENCODE	443	chr8	+
ENST00000523935	RP11-181B11.2	GENCODE	443	chr8	+
uc001hkf.2	PROX1-AS1	UCSC_knowngene	3558	chr1	-
ENST00000517915	RP11-89K10.1	GENCODE	317	chr8	+
ENST00000441882	RP11-395B7.4	GENCODE	407	chr7	-
ENST00000506899	RP11-22A3.2	GENCODE	2694	chr4	-
ENST00000480811	C17orf76-AS1	GENCODE	968	chr17	+
ENST00000540904	SNHG1	GENCODE	594	chr11	-
ENST00000540904	SNHG1	GENCODE	594	chr11	-
ENST00000540904	SNHG1	GENCODE	594	chr11	-
ENST00000533311	RP11-655M14.13	GENCODE	831	chr11	-
ENST00000558952	CRNDE	GENCODE	600	chr16	-
ENST00000573820	LA16c-390H2.4	GENCODE	480	chr16	+
NR_037947	NSUN2	RefSeq	3304	chr5	-
NR_037947	NSUN2	RefSeq	3304	chr5	-
ENST00000521586	RP11-382A18.2	GENCODE	290	chr8	+
ENST00000569927	RP11-1299A16.3	GENCODE	5647	chr4	+
ENST00000428044	RP11-390P2.4	GENCODE	421	chr6	+
ENST00000555043	RP11-649E7.5	GENCODE	2469	chr14	-
ENST00000453795	AC025627.9	pseudogene	1220	chr17	-
ENST00000418567	RP11-379B8.1	GENCODE	822	chr6	-
TCONS_00006262	XLOC_002865	LincRNAs identified	819	chr3	+
uc002kmi.3	BC094703	UCSC_knowngene	2250	chr18	+
uc002kmi.3	BC094703	UCSC_knowngene	2250	chr18	+
uc002kmi.3	BC094703	UCSC_knowngene	2250	chr18	+
uc002kmi.3	BC094703	UCSC_knowngene	2250	chr18	+
uc002kmi.3	BC094703	UCSC_knowngene	2250	chr18	+
uc002kmi.3	BC094703	UCSC_knowngene	2250	chr18	+
uc002kmi.3	BC094703	UCSC_knowngene	2250	chr18	+
uc002kmi.3	BC094703	UCSC_knowngene	2250	chr18	+
ENST00000411839	RP11-128M1.1	GENCODE	536	chr20	+
ENST00000492083	ANKRD19P	GENCODE	426	chr9	+
ENST00000514608	RP11-21I10.2	GENCODE	974	chr4	-
uc001xqo.1	AK096592	UCSC_knowngene	3327	chr14	+

62507483	62512243		CGAACCACAGAA	100505815	intron sense-overlapp
62507483	62512243		CGAACCACAGAA	100505815	intron sense-overlapp
62507483	62512243		CGAACCACAGAA	100505815	intron sense-overlapp
62507483	62512243		CGAACCACAGAA	100505815	intron sense-overlapp
62507483	62512243		CGAACCACAGAA	100505815	intron sense-overlapp
40958828	40974333		CTCTTTGAGATTTCATCCAGAGGCT		intronic antisense
171496888	171551884		TGGCTAACAGGTGTGGGTATGCCTG		intronic antisense
171496888	171551884		TGGCTAACAGGTGTGGGTATGCCTG		intronic antisense
65941289	65955046		CTGAAAGAAAATACAGTGTGTCTAA		intergenic
54529761	54591029		AACCAATAGCAGTGAAAACTCACAT		natural antisense
114317118	114556880		TTAAATTTTACAAGCATACTCTCTTT		intronic antisense
18811591	18812178	NM_002882	CCATCCACTTCCTGAATATTGAGAATGCACAGAAATTC		
56247852	56263392	NR_026796	GTTAAATACCTC	645687	intergenic
20043452	20056627		CAAATGCTAGCTCTCCTCTTACTAA		intergenic
23162205	23163342		ACTCATTTTCTTGAAACACACAGAA		intronic antisense
23162205	23163342		ACTCATTTTCTTGAAACACACAGAA		intronic antisense
135622705	135628296		GCTTTTCTGGACCATAATTATATGA		intronic antisense
135622705	135628296		GCTTTTCTGGACCATAATTATATGA		intronic antisense
135622705	135628296		GCTTTTCTGGACCATAATTATATGA		intronic antisense
68779821	68780795		TAGAGCCCTCTCCTGCTGGAAGAGA		natural antisense
68779821	68780795		TAGAGCCCTCTCCTGCTGGAAGAGA		natural antisense
74185096	74208560		AACAGATCTAAATTCAACATGACTT		natural antisense
74185096	74208560		AACAGATCTAAATTCAACATGACTT		natural antisense
96337195	96370995		GAATATGATTTGCAGAAATCTCAGT		intron sense-overlapp
125283923	125289825		TGGAGAGACCAAGTTTCAGCAGCCA		intergenic
34146513	34171889		TCTTTTTTAGGGC	80307	intergenic
17093707	17094196		TTAACTGCGTTCCAGAGTTTCCGTC		intergenic
105119935	105122124		TCATTTTGCTGATCTGATGCTTCTCC		intergenic
28304780	28313204		GCATAGCTATTTGTCTGTTTGAGTT		natural antisense
28304780	28313204		GCATAGCTATTTGTCTGTTTGAGTT		natural antisense
213991094	214139608	ENST00000433082	GACAACATGACT	100505832	intergenic
127601458	127647029		GAAAAGTCTTGGGGATCCTGAATTC		intergenic
100657600	100660889	ENST00000448513	ATCTGCTCTCCCTCAGATTCTGGTC		natural antisense
12860724	12866585		TGTTACTGAAGACAAAGCAGATACC		intergenic
16342640	16345146	ENST00000497774	ACCCCGAGGGCG	125144	intergenic
62622151	62622960	uc001nvs.3	TGTTGTTGCACCC	23642	bidirectional
62622151	62622960	uc001nvs.3	TGTTGTTGCACCC	23642	bidirectional
62622151	62622960	uc001nvs.3	TGTTGTTGCACCC	23642	bidirectional
67385749	67394775		TTGGATTTACAGTACATTGATGGAT		intergenic
54957192	54962798		CTGGCTATTGGA	643911	intergenic
3594430	3595785		CAAAAGGGGATGCTTTTCACAGCCA		intronic antisense
6599351	6633473		TCATCATAAGAG	54888	exon sense-overlappi
6599351	6633473		TCATCATAAGAG	54888	exon sense-overlappi
128351518	128404876		TGTGGTTCCTGTTGGCCTTTTAACTC		intergenic
99580054	99585701		TGGGGCATATCTGTCTCGTAAATAT		bidirectional
139015455	139018164		CGCCTCATTTTTTACAATGTGGGCC		intergenic
50087532	50090198		TCGTTAGGGTACAACTGAATGCTGA		natural antisense
20415872	20420792		TAAGCATCTTGGTGGCTGAGCGGTGACCACCTGTCAGC		
82554901	82643197		CTTGAGAAAAGCACAGTTCTCTGTC		intergenic
148942374	148944122		CGTCTTAAGGATTTCTGGACTGGGT		intergenic
3603735	3608323		CTGCAGTGAAGCATCAGAAAAGTAT		intronic antisense
3603735	3608323		CTGCAGTGAAGCATCAGAAAAGTAT		intronic antisense
3603735	3608323		CTGCAGTGAAGCATCAGAAAAGTAT		intronic antisense
3603735	3608323		CTGCAGTGAAGCATCAGAAAAGTAT		intronic antisense
3603735	3608323		CTGCAGTGAAGCATCAGAAAAGTAT		intronic antisense
3603735	3608323		CTGCAGTGAAGCATCAGAAAAGTAT		intronic antisense
3603735	3608323		CTGCAGTGAAGCATCAGAAAAGTAT		intronic antisense
3603735	3608323		CTGCAGTGAAGCATCAGAAAAGTAT		intronic antisense
2187872	2193797		CCGGGCTCTATCCGCCAGCCCACGA		intergenic
95584503	95592045	ENST00000460854,	AGTCATCTTTGA	138649	intergenic
119990478	119991452	NM_018407	TAGGCAGTGAAACATCCCCCAAATC		intergenic
75292223	75295550		CAACCTGCTTTTCATATGACACAGT		intron sense-overlapp

NM_199359	TPD52L2	tumor protein D54 is	+	62496580	62522898
NM_199360	TPD52L2	tumor protein D54 is	+	62496580	62522898
NM_199361	TPD52L2	tumor protein D54 is	+	62496580	62522898
NM_199362	TPD52L2	tumor protein D54 is	+	62496580	62522898
NM_199363	TPD52L2	tumor protein D54 is	+	62496580	62522898
NM_198494	ZNF642	zinc finger protein 64	+	40943301	40962015
NM_001083615	MYO3B	myosin-IIIb isoform	+	171034654	171511674
NM_138995	MYO3B	myosin-IIIb isoform	+	171034654	171511674
NM_019030	DHX29	ATP-dependent RNA	-	54552072	54603521
NM_153612	HS3ST5	heparan sulfate gluco	-	114376749	114384041
AAAACAAAGTTTGAAGAATGCA					
NM_004442	EPHB2	ephrin type-B recepto	+	23037330	23241823
NM_017449	EPHB2	ephrin type-B recepto	+	23037330	23241823
NM_001134830	AHI1	jouberin isoform a	-	135605109	135818903
NM_001134831	AHI1	jouberin isoform a	-	135605109	135818903
NM_017651	AHI1	jouberin isoform a	-	135605109	135818903
NM_001098515	MRGPRF	mas-related G-protei	-	68771861	68780850
NM_145015	MRGPRF	mas-related G-protei	-	68771861	68780850
NM_080916	DGUOK	deoxyguanosine kina	+	74153952	74186088
NM_080918	DGUOK	deoxyguanosine kina	+	74153952	74186088
NM_018063	HELLS	lymphoid-specific he	+	96305573	96361856
NM_001258211	FBXO16	F-box only protein 1(	-	28285925	28347835
NM_172366	FBXO16	F-box only protein 1(	-	28285925	28347835
NM_001164462	MUC12	mucin-12 precursor	+	100612903	100662230
NM_001012662	SLC3A2	4F2 cell-surface anti	+	62623483	62656355
NM_001012664	SLC3A2	4F2 cell-surface anti	+	62623483	62656355
NM_002394	SLC3A2	4F2 cell-surface anti	+	62623483	62656355
NM_178844	NLRC3	protein NLRC3	-	3589035	3627392
NM_001193455	NSUN2	tRNA (cytosine(34)-(	-	6599351	6633473
NM_017755	NSUN2	tRNA (cytosine(34)-(	-	6599351	6633473
NM_005723	TSPAN5	tetraspanin-5	-	99391517	99579812
NM_002408	MGAT2	alpha-1,6-mannosyl-(	+	50087488	50090199
;TGCCTACCATTGTGGAAGAGGT					
NM_001003809	DLGAP1	disks large-associate	-	3496029	3845358
NM_001242761	DLGAP1	disks large-associate	-	3502142	4455266
NM_001242762	DLGAP1	disks large-associate	-	3496029	3874767
NM_001242763	DLGAP1	disks large-associate	-	3496029	3874767
NM_001242764	DLGAP1	disks large-associate	-	3496029	3874253
NM_001242765	DLGAP1	disks large-associate	-	3502142	3845358
NM_001242766	DLGAP1	disks large-associate	-	3496029	3845358
NM_004746	DLGAP1	disks large-associate	-	3496029	4455266
NM_019589	YLPM1	YLP motif-containin	+	75230068	75304013

400.742417	72.696413	8.184351	6.43172	430.2499	444.7889
400.742417	72.696413	8.184351	6.43172	430.2499	444.7889
400.742417	72.696413	8.184351	6.43172	430.2499	444.7889
400.742417	72.696413	8.184351	6.43172	430.2499	444.7889
400.742417	72.696413	8.184351	6.43172	430.2499	444.7889
62.900613	20.723452	5.605172	4.583836	60.827667	36.478218
54.54677	8.533384	4.899135	3.184363	21.749039	14.744379
54.54677	8.533384	4.899135	3.184363	21.749039	14.744379
61.413842	19.503972	5.526121	4.378094	68.73289	58.854458
145.05073	22.440628	6.823642	4.371599	154.68082	157.39021
851.596687	209.394096	9.144478	7.306408	881.5901	964.2894
5816.095817	1286.79455	12.035452	10.728421	5597.627	5472.839
387.247206	18.930667	7.48319	3.836445	515.30615	43.11935
85.482952	24.454407	6.068845	4.764028	95.53612	86.710464
37.750929	7.253517	4.569731	2.826168	63.98454	32.927906
37.750929	7.253517	4.569731	2.826168	63.98454	32.927906
27.935909	5.522752	4.308606	2.567578	40.536224	39.428658
27.935909	5.522752	4.308606	2.567578	40.536224	39.428658
27.935909	5.522752	4.308606	2.567578	40.536224	39.428658
1561.327045	269.743392	9.939345	7.984968	1816.5511	2981.1252
1561.327045	269.743392	9.939345	7.984968	1816.5511	2981.1252
2315.61645	517.755328	10.549264	8.920512	1611.0001	732.5492
2315.61645	517.755328	10.549264	8.920512	1611.0001	732.5492
362.456067	46.338651	8.092144	5.704176	347.45618	380.49393
43.068998	10.182692	4.482868	3.320884	14.94487	4.9999995
361.010275	35.062596	7.667166	5.399811	330.57538	37.87852
35.160467	11.579423	4.789378	3.577235	45.00619	34.729465
78.462039	27.716027	5.918976	4.872803	98.66611	96.89956
40.108941	11.385621	4.895455	3.597634	63.43673	33.615604
40.108941	11.385621	4.895455	3.597634	63.43673	33.615604
27.433595	5.858006	4.36218	2.631586	21.446217	14.700041
111.122463	25.892006	6.264322	4.968256	152.99684	78.73606
121.996748	38.412909	6.521266	5.276415	123.85641	121.78383
100.493315	25.151237	6.054609	4.948212	131.82559	124.76656
447.260768	113.295859	8.36343	7.087982	436.78445	562.5906
132.321046	33.232907	6.673631	5.32991	134.44794	137.22513
132.321046	33.232907	6.673631	5.32991	134.44794	137.22513
132.321046	33.232907	6.673631	5.32991	134.44794	137.22513
170.982025	44.829834	7.000242	5.753898	177.85097	207.0029
2214.665237	70.263428	10.474811	6.141479	1809.9934	468.56042
132.079049	32.469265	6.678165	5.319433	147.8622	107.86309
156.465082	41.732691	6.933038	5.653742	165.53354	142.97847
156.465082	41.732691	6.933038	5.653742	165.53354	142.97847
2526.19411	173.813612	10.541442	7.663914	3953.3062	5371.6035
235.072628	24.18902	7.327796	4.229936	208.73431	226.13681
38.152785	10.613111	4.899736	3.429651	40.4932	41.963474
27.81431	9.480168	4.463305	3.359206	26.456934	22.90284
45.24147	7.807521	4.956636	3.201168	35.083885	41.6765
108.556846	19.490936	5.8804	4.180296	177.34029	163.45955
20.374541	5.319966	3.971912	2.485124	28.58447	19.054602
205.267666	22.066708	7.047309	4.694375	212.65639	161.93651
205.267666	22.066708	7.047309	4.694375	212.65639	161.93651
205.267666	22.066708	7.047309	4.694375	212.65639	161.93651
205.267666	22.066708	7.047309	4.694375	212.65639	161.93651
205.267666	22.066708	7.047309	4.694375	212.65639	161.93651
205.267666	22.066708	7.047309	4.694375	212.65639	161.93651
205.267666	22.066708	7.047309	4.694375	212.65639	161.93651
205.267666	22.066708	7.047309	4.694375	212.65639	161.93651
63.93389	14.26614	5.398566	4.087218	94.184044	139.80139
228.947117	73.426932	7.424546	6.131584	303.64093	168.1736
1436.524292	245.432518	10.037526	8.193663	1669.4965	748.42615
365.283832	110.579904	8.067936	6.975574	299.98596	306.89484

470.94647	512.59265	160.49998	385.3766	82.78152	48.545845
470.94647	512.59265	160.49998	385.3766	82.78152	48.545845
470.94647	512.59265	160.49998	385.3766	82.78152	48.545845
470.94647	512.59265	160.49998	385.3766	82.78152	48.545845
470.94647	512.59265	160.49998	385.3766	82.78152	48.545845
86.209946	61.677605	68.47607	63.734173	19.405777	14.29368
21.852617	157.06029	23.627157	88.24714	6.781066	4.9999995
21.852617	157.06029	23.627157	88.24714	6.781066	4.9999995
98.63027	61.397125	28.800558	52.067753	27.622778	11.89753
145.94997	131.66891	121.8468	158.76767	25.149265	6.740981
498.32635	246.11717	1348.3446	1170.9125	334.68225	103.2892
4795.0884	3890.8508	7654.533	7485.6367	1181.1555	1089.0394
992.30145	51.905445	397.1836	323.66724	4.9999995	4.9999995
81.61853	71.47241	91.46414	86.09605	24.44099	11.220092
80.17308	23.3321	9.746559	16.34139	4.9999995	4.9999995
80.17308	23.3321	9.746559	16.34139	4.9999995	4.9999995
40.548443	14.036548	12.637059	20.42852	6.4704423	4.9999995
40.548443	14.036548	12.637059	20.42852	6.4704423	4.9999995
40.548443	14.036548	12.637059	20.42852	6.4704423	4.9999995
677.9401	457.61417	1810.0018	1624.7299	320.51886	88.98837
677.9401	457.61417	1810.0018	1624.7299	320.51886	88.98837
2355.707	1376.1528	4035.575	3782.7146	235.29517	71.16266
2355.707	1376.1528	4035.575	3782.7146	235.29517	71.16266
308.1717	227.4295	437.69894	473.48615	45.930614	19.24423
18.179203	105.76172	40.461678	74.06652	6.0329356	4.9999995
562.7624	144.72275	614.1825	475.9401	23.541683	22.387278
27.620659	27.20592	37.41918	38.98139	8.592903	4.9999995
76.4604	82.836174	65.4012	50.50879	30.26115	8.805974
39.82322	58.73749	27.632174	17.408428	8.09458	8.713233
39.82322	58.73749	27.632174	17.408428	8.09458	8.713233
20.049652	25.374065	40.790966	42.24063	4.9999995	4.9999995
211.98172	112.15047	39.725136	71.144554	22.65035	19.460018
85.956696	75.23282	168.77599	156.37474	37.343063	11.323491
52.497528	173.24991	22.727749	97.892555	20.300669	22.57629
267.87857	633.3051	301.52396	481.48193	106.576035	91.05321
106.31301	96.970146	157.35245	161.6176	35.577953	31.857523
106.31301	96.970146	157.35245	161.6176	35.577953	31.857523
106.31301	96.970146	157.35245	161.6176	35.577953	31.857523
132.61313	93.46194	213.34871	201.6145	37.870285	32.57299
2765.8367	3524.412	1830.8657	2888.3232	48.439484	56.412003
121.0324	106.439415	147.42929	161.8479	27.994642	30.020905
159.25587	136.24634	150.9698	183.80647	45.105595	30.954994
159.25587	136.24634	150.9698	183.80647	45.105595	30.954994
2132.8208	1812.2202	550.87396	1336.34	226.25665	315.07864
207.01282	447.94434	64.89527	255.71222	39.89247	6.946046
40.77576	25.131998	40.435783	40.116497	16.868486	6.9931965
24.764153	40.513588	20.80523	31.443117	10.345749	4.9999995
31.654156	16.371872	85.89864	60.763767	7.550785	7.2538986
179.48364	12.012521	73.59105	45.454025	28.163158	11.197451
28.905907	17.768698	10.675709	17.257862	4.9999995	4.9999995
253.88686	36.746304	308.48373	257.8962	19.503036	22.273462
253.88686	36.746304	308.48373	257.8962	19.503036	22.273462
253.88686	36.746304	308.48373	257.8962	19.503036	22.273462
253.88686	36.746304	308.48373	257.8962	19.503036	22.273462
253.88686	36.746304	308.48373	257.8962	19.503036	22.273462
253.88686	36.746304	308.48373	257.8962	19.503036	22.273462
253.88686	36.746304	308.48373	257.8962	19.503036	22.273462
253.88686	36.746304	308.48373	257.8962	19.503036	22.273462
50.89563	40.882626	23.713003	34.126648	10.246801	10.32512
324.02002	154.47339	181.10097	242.27379	92.744514	9.94977
2004.8351	1391.0554	1285.7776	1519.555	338.68628	120.9292
272.63986	598.0412	240.00005	474.14108	125.7918	48.612125

113.574196	95.654915	43.03704	52.584965	8.28749	8.484675
113.574196	95.654915	43.03704	52.584965	8.28749	8.484675
113.574196	95.654915	43.03704	52.584965	8.28749	8.484675
113.574196	95.654915	43.03704	52.584965	8.28749	8.484675
113.574196	95.654915	43.03704	52.584965	8.28749	8.484675
35.462196	12.557801	23.40824	19.213018	5.4680576	5.096133
5.5509233	15.98697	7.751524	10.12982	3.9830396	3.9070604
5.5509233	15.98697	7.751524	10.12982	3.9830396	3.9070604
39.295685	9.539519	16.052668	12.61565	5.642805	5.718027
45.286613	5.469217	36.441875	15.5558195	6.815986	7.059497
590.3896	26.40857	149.53032	52.064636	9.316734	9.574456
1255.398	1280.3105	1674.8962	1239.9677	11.95285	11.963039
4.9999995	44.04406	25.451574	29.08837	8.525904	5.3180714
47.140793	28.098911	20.561304	15.264351	6.131279	6.239282
4.9999995	14.036647	4.9999995	9.484457	5.5416555	4.965174
4.9999995	14.036647	4.9999995	9.484457	5.5416555	4.965174
4.9999995	4.9999995	6.666071	4.9999995	4.8775835	5.198589
4.9999995	4.9999995	6.666071	4.9999995	4.8775835	5.198589
4.9999995	4.9999995	6.666071	4.9999995	4.8775835	5.198589
772.3608	119.09471	208.09142	109.40619	10.348287	11.118158
772.3608	119.09471	208.09142	109.40619	10.348287	11.118158
407.02402	437.77448	1277.904	677.37164	10.18626	9.19311
407.02402	437.77448	1277.904	677.37164	10.18626	9.19311
80.5605	30.290562	61.760548	40.24545	7.988207	8.280949
4.9999995	19.78607	13.413679	11.863471	3.4295454	2.3499577
27.37763	45.989754	47.578785	43.500446	7.91233	5.1460795
11.190721	14.184779	21.117222	9.390912	5.0325212	5.0352874
57.56096	18.96397	27.29727	23.406837	6.173862	6.3803196
21.390526	9.842773	4.9999995	15.272612	5.5296226	4.992101
21.390526	9.842773	4.9999995	15.272612	5.5296226	4.992101
4.9999995	4.9999995	9.18361	5.964427	3.9617064	3.9025369
21.219084	40.961533	29.958815	21.102236	6.8007755	6.1107016
91.79012	35.628662	34.374077	20.01804	6.5010376	6.7027397
19.092918	35.926678	22.215483	30.795382	6.5946174	6.735208
101.83106	66.982376	198.81937	114.5131	8.305873	8.826876
34.78301	25.102047	48.171326	23.905582	6.6201415	6.8688946
34.78301	25.102047	48.171326	23.905582	6.6201415	6.8688946
34.78301	25.102047	48.171326	23.905582	6.6201415	6.8688946
56.170876	39.223873	69.5932	33.54778	7.0197916	7.439549
29.159756	171.99512	20.56823	95.005974	10.341629	8.564204
43.80249	24.894522	33.48304	34.61999	6.7525077	6.5350475
59.338627	30.55569	51.854176	32.587063	6.9132805	6.9284525
59.338627	30.55569	51.854176	32.587063	6.9132805	6.9284525
93.46478	185.0136	102.13541	120.932594	11.44923	11.938954
71.041794	8.385096	10.967418	7.9012985	7.252239	7.565162
19.074255	4.9999995	10.742729	4.9999995	4.876491	5.2811337
13.281881	8.904524	11.00595	8.342902	4.2690463	4.4754643
7.3542194	4.9999995	11.508641	8.177583	4.672278	5.2708964
49.60299	5.136861	11.837968	11.007189	7.0162473	7.110643
4.9999995	4.9999995	6.919796	4.9999995	4.383658	4.238575
18.16654	14.15988	39.22512	19.072212	7.282482	7.096914
18.16654	14.15988	39.22512	19.072212	7.282482	7.096914
18.16654	14.15988	39.22512	19.072212	7.282482	7.096914
18.16654	14.15988	39.22512	19.072212	7.282482	7.096914
18.16654	14.15988	39.22512	19.072212	7.282482	7.096914
18.16654	14.15988	39.22512	19.072212	7.282482	7.096914
18.16654	14.15988	39.22512	19.072212	7.282482	7.096914
18.16654	14.15988	39.22512	19.072212	7.282482	7.096914
16.488829	13.169891	20.10348	15.262721	6.110185	6.898524
91.752	38.556618	136.13249	71.4262	7.794395	7.156603
404.9294	176.62932	240.06479	191.35612	10.235072	9.223209
206.79488	73.68741	128.37483	80.21838	7.7760553	7.9894986

8.530447	8.683948	7.101765	8.01778	6.5971103	6.4474926
8.530447	8.683948	7.101765	8.01778	6.5971103	6.4474926
8.530447	8.683948	7.101765	8.01778	6.5971103	6.4474926
8.530447	8.683948	7.101765	8.01778	6.5971103	6.4474926
8.530447	8.683948	7.101765	8.01778	6.5971103	6.4474926
6.1145105	5.5712175	5.8973746	5.4837375	4.5156016	4.6495347
4.135307	6.9723787	4.4530163	5.9440107	2.9597833	2.4279919
4.135307	6.9723787	4.4530163	5.9440107	2.9597833	2.4279919
6.3031664	5.5642824	4.7137165	5.2147317	5.0232244	4.362938
6.8646913	6.7106695	6.706986	6.784023	4.895192	3.5127513
8.61003	7.6380415	10.12401	9.603598	8.653327	7.5387526
11.87361	11.575719	12.583225	12.264268	10.508742	10.964863
9.582331	5.303317	8.379883	7.789635	2.3467326	2.4279919
6.03405	5.7947083	6.3046255	5.909124	4.852783	4.2797875
6.0102873	4.053699	3.2385747	3.608997	2.3467326	2.4279919
6.0102873	4.053699	3.2385747	3.608997	2.3467326	2.4279919
5.0254507	3.2326677	3.5995119	3.9178333	2.8905628	2.4279919
5.0254507	3.2326677	3.5995119	3.9178333	2.8905628	2.4279919
5.0254507	3.2326677	3.5995119	3.9178333	2.8905628	2.4279919
9.042802	8.524173	10.52566	10.076989	8.594909	7.3246202
9.042802	8.524173	10.52566	10.076989	8.594909	7.3246202
10.861384	10.103235	11.679065	11.2725315	8.131969	6.9998484
10.861384	10.103235	11.679065	11.2725315	8.131969	6.9998484
7.9296865	7.5251203	8.514866	8.314035	5.7509656	5.0912514
3.8650498	6.375064	5.1793556	5.698235	2.7952871	2.4279919
8.774788	6.8501253	8.999585	8.320087	4.797934	5.3134613
4.4786997	4.303238	5.0738363	4.812683	3.3134234	2.4279919
5.9405036	6.0174527	5.8334036	5.168317	5.1523995	3.9140952
4.9985676	5.495871	4.65747	3.699097	3.2193837	3.8980575
4.9985676	5.495871	4.65747	3.699097	3.2193837	3.8980575
4.0030117	4.1908183	5.193052	4.921953	2.3467326	2.4279919
7.4123926	6.4676766	5.151762	5.6426225	4.7410307	5.1078234
6.110303	5.873739	7.1736655	6.766109	5.4592195	4.292384
5.395195	7.1109986	4.3987904	6.0928473	4.5789	5.3249574
7.730387	8.991877	7.9880214	8.337546	6.972909	7.358615
6.415063	6.2493677	7.0776844	6.810637	5.3881826	5.83121
6.415063	6.2493677	7.0776844	6.810637	5.3881826	5.83121
6.415063	6.2493677	7.0776844	6.810637	5.3881826	5.83121
6.7259846	6.1934466	7.4956903	7.126987	5.4796605	5.864566
11.0663395	11.444418	10.544133	10.8881445	5.832375	6.673009
6.599952	6.384901	6.9839907	6.8125935	5.0430794	5.746234
6.9805536	6.7612343	7.019458	6.995248	5.7253222	5.7898073
6.9805536	6.7612343	7.019458	6.995248	5.7253222	5.7898073
10.704519	10.504831	8.848296	9.802824	8.073602	9.156576
7.378119	8.498161	5.823613	7.4494824	5.5541863	3.5599482
5.0343432	4.1754317	5.1783333	4.852681	4.309779	3.5686388
4.319272	4.921042	4.2755427	4.51946	3.5985448	2.4279919
4.674862	3.4819586	6.2199244	5.4198976	3.1116595	3.6211133
7.1583014	2.9806724	5.996013	5.020525	5.0510387	4.2757807
4.544694	3.6090968	3.367957	3.6874905	2.3467326	2.4279919
7.653679	4.7664685	8.022054	7.4622574	4.5225024	5.3062377
7.653679	4.7664685	8.022054	7.4622574	4.5225024	5.3062377
7.653679	4.7664685	8.022054	7.4622574	4.5225024	5.3062377
7.653679	4.7664685	8.022054	7.4622574	4.5225024	5.3062377
7.653679	4.7664685	8.022054	7.4622574	4.5225024	5.3062377
7.653679	4.7664685	8.022054	7.4622574	4.5225024	5.3062377
7.653679	4.7664685	8.022054	7.4622574	4.5225024	5.3062377
7.653679	4.7664685	8.022054	7.4622574	4.5225024	5.3062377
5.354027	4.936578	4.457778	4.6343026	3.583119	4.1535244
8.004561	6.946399	7.2689795	7.3763385	6.766363	4.0966506
10.612279	10.119452	10.057912	9.977232	8.673	7.765064
7.755804	8.905782	7.663843	8.316631	7.207845	6.450272

6.7897825	6.9109945	5.699424	6.1455135
6.7897825	6.9109945	5.699424	6.1455135
6.7897825	6.9109945	5.699424	6.1455135
6.7897825	6.9109945	5.699424	6.1455135
6.7897825	6.9109945	5.699424	6.1455135
5.1523995	3.7681487	4.822117	4.595216
2.6961973	4.1405125	3.2495263	3.632169
2.6961973	4.1405125	3.2495263	3.632169
5.2956986	3.350052	4.2770696	3.9595785
5.4992824	2.5799823	5.4618535	4.280535
9.149382	4.9151745	7.4518023	6.1300116
10.235072	10.825296	10.961977	10.874577
2.350709	5.709058	4.940152	5.244028
5.5574336	5.015324	4.6274443	4.251393
2.350709	3.9391282	2.3618152	3.530634
2.350709	3.9391282	2.3618152	3.530634
2.350709	2.3408759	3.0425775	2.352752
2.350709	2.3408759	3.0425775	2.352752
2.350709	2.3408759	3.0425775	2.352752
9.541262	7.252679	7.9527364	7.243604
9.541262	7.252679	7.9527364	7.243604
8.629734	9.222115	10.576612	9.962795
8.629734	9.222115	10.576612	9.962795
6.296566	5.131464	6.2142	5.740606
2.350709	4.464144	4.017787	3.8693838
4.796448	5.780081	5.847417	5.863523
3.5801945	3.9557266	4.66864	3.5174322
5.83351	4.3980055	5.043133	4.8956723
4.454823	3.3991673	2.3618152	4.252555
4.454823	3.3991673	2.3618152	4.252555
2.350709	2.3408759	3.483499	2.8397102
4.44475	5.594043	5.1819935	4.7398973
6.4854016	5.386405	5.3787117	4.6563663
4.303238	5.39892	4.745561	5.3376966
6.6332884	6.3718486	7.8821135	7.3091154
5.1287136	4.8391314	5.8633924	4.928829
5.1287136	4.8391314	5.8633924	4.928829
5.1287136	4.8391314	5.8633924	4.928829
5.801211	5.529325	6.3851333	5.4634895
4.886387	7.802719	4.6277194	7.026667
5.449744	4.8249235	5.3382306	5.514385
5.8779507	5.1432085	5.966532	5.4196315
5.8779507	5.1432085	5.966532	5.4196315
6.512019	7.922687	6.9291496	7.38945
6.1223397	3.1584446	3.7304301	3.254265
4.3025103	2.3408759	3.7033482	2.352752
3.8066633	3.2540982	3.7355707	3.332369
3.0291898	2.3408759	3.797826	3.3063428
5.6308594	2.5295956	3.8407645	3.7537358
2.350709	2.3408759	3.0916831	2.352752
4.2383866	3.9529219	5.563604	4.5825973
4.2383866	3.9529219	5.563604	4.5825973
4.2383866	3.9529219	5.563604	4.5825973
4.2383866	3.9529219	5.563604	4.5825973
4.2383866	3.9529219	5.563604	4.5825973
4.2383866	3.9529219	5.563604	4.5825973
4.2383866	3.9529219	5.563604	4.5825973
4.2383866	3.9529219	5.563604	4.5825973
4.1013002	3.8410258	4.593457	4.2508802
6.4848495	5.5040965	7.3294683	6.6080737
8.620922	7.845251	8.16127	8.096471
7.6424513	6.524137	7.2481155	6.780623

ASHGA5P044228	0.000245181	0.012133712	4.9727329	up	noncoding
ASHGA5P042595	0.022694376	0.093195005	2.3123553	up	noncoding
ASHGA5P051677	0.002412598	0.031997277	2.2367614	up	noncoding
ASHGA5P031617	4.67316E-06	0.003111069	2.0764589	up	noncoding
ASHGA5P041337	0.002809936	0.034179323	3.3080749	up	noncoding
ASHGA5P029537	0.005014513	0.044969228	2.4185236	up	noncoding
ASHGA5P029537	0.005014513	0.044969228	2.4185236	up	noncoding
ASHGA5P035732	0.00279312	0.034179323	2.2816323	up	noncoding
ASHGA5P016346	0.006791964	0.052273054	3.145239	up	noncoding
ASHGA5P028459	0.000189801	0.01146874	5.2941512	up	noncoding
ASHGA5P044744	0.002427035	0.032019471	2.518527	up	noncoding
ASHGA5P021755	9.85012E-05	0.009080764	2.8687017	up	noncoding
ASHGA5P021755	9.85012E-05	0.009080764	2.8687017	up	noncoding
ASHGA5P021755	9.85012E-05	0.009080764	2.8687017	up	noncoding
ASHGA5P058489	0.000271832	0.012302262	3.8635173	up	noncoding
ASHGA5P021539	0.000893021	0.020125432	3.3510039	up	noncoding
ASHGA5P029013	0.000551831	0.016097331	2.9361061	up	noncoding
ASHGA5P043694	0.018203891	0.081962493	2.1373239	up	noncoding
ASHGA5P043694	0.018203891	0.081962493	2.1373239	up	noncoding
ASHGA5P025658	0.021397878	0.090137716	2.1448767	up	noncoding
ASHGA5P047541	0.00235576	0.031728208	5.332836	up	noncoding
ASHGA5P038000	0.041926277	0.13107999	2.580326	up	noncoding
ASHGA5P036261	0.035551975	0.119284932	2.3618837	up	noncoding
ASHGA5P037473	0.036390773	0.120992679	3.2619341	up	noncoding
ASHGA5P037473	0.036390773	0.120992679	3.2619341	up	noncoding
ASHGA5P037473	0.036390773	0.120992679	3.2619341	up	noncoding
ASHGA5P037473	0.036390773	0.120992679	3.2619341	up	noncoding
ASHGA5P037473	0.036390773	0.120992679	3.2619341	up	noncoding
ASHGA5P054918	9.93559E-05	0.009080764	2.6097212	up	noncoding
ASHGA5P043658	0.005453514	0.046969887	2.5688962	up	noncoding
ASHGA5P015886	0.01093771	0.064718011	3.4706248	up	noncoding
ASHGA5P015886	0.01093771	0.064718011	3.4706248	up	noncoding
ASHGA5P015886	0.01093771	0.064718011	3.4706248	up	noncoding
ASHGA5P036783	0.025363417	0.099118545	2.1476594	up	noncoding
ASHGA5P038470	0.012018245	0.06714145	2.5620256	up	noncoding
ASHGA5P049487	0.007268553	0.053644962	2.1886608	up	noncoding
ASHGA5P040059	0.016132575	0.077717436	2.2538267	up	noncoding
ASHGA5P034023	0.006636074	0.051810468	2.061943	up	noncoding
ASHGA5P029339	0.002609503	0.032993346	2.023697	up	noncoding
ASHGA5P030482	0.008153012	0.056720455	2.8674021	up	noncoding
ASHGA5P030482	0.008153012	0.056720455	2.8674021	up	noncoding
ASHGA5P030482	0.008153012	0.056720455	2.8674021	up	noncoding
ASHGA5P030482	0.008153012	0.056720455	2.8674021	up	noncoding
ASHGA5P030482	0.008153012	0.056720455	2.8674021	up	noncoding
ASHGA5P023414	0.00173415	0.027807594	2.6437683	up	noncoding
ASHGA5P028264	0.002236962	0.031175176	2.2213742	up	noncoding
ASHGA5P028264	0.002236962	0.031175176	2.2213742	up	noncoding
ASHGA5P037118	0.000400193	0.014223693	3.2594046	up	noncoding
ASHGA5P034195	0.007486306	0.054377035	2.3550697	up	noncoding
ASHGA5P027837	0.001966782	0.029367232	2.350743	up	noncoding
ASHGA5P044929	0.005590085	0.04747657	2.4997847	up	noncoding
ASHGA5P029638	0.000577699	0.016232767	2.9733644	up	noncoding
ASHGA5P029638	0.000577699	0.016232767	2.9733644	up	noncoding
ASHGA5P036620	0.014981413	0.074989017	2.1393041	up	noncoding
ASHGA5P000322	0.028584879	0.105405981	2.1022091	up	noncoding
ASHGA5P052420	0.007692703	0.055067411	2.3951771	up	noncoding
ASHGA5P036777	0.001322516	0.024233782	2.5964448	up	noncoding
ASHGA5P055679	0.036656376	0.121455823	2.4217533	up	noncoding
ASHGA5P053210	0.000495467	0.015564508	4.4700719	up	noncoding
ASHGA5P038919	0.001615729	0.026987492	2.347238	up	noncoding
ASHGA5P044525	0.006267053	0.05052414	2.7395188	up	noncoding
ASHGA5P048836	0.012765774	0.069158933	2.4401793	up	noncoding

ENST00000578500	RP11-360L9.7	GENCODE	884	chr8	-
TCONS_00020785	XLOC_010078	LincRNAs identified	277	chr12	-
ENST00000273411	RPL39P5	GENCODE	449	chr3	-
ENST00000568262	CTD-2196E14.6	GENCODE	919	chr16	+
ENST00000510853	CTC-369A16.3	GENCODE	591	chr5	+
NR_037792	LRR1	RefSeq	1965	chr14	+
NR_037792	LRR1	RefSeq	1965	chr14	+
ENST00000426539	AC130710.1	GENCODE	1096	chr2	+
uc003ftr.2	LOC100131551	UCSC_knowngene	1718	chr3	-
ENST00000413591	LINC00365	GENCODE	2618	chr13	-
ENST00000518096	TUBBP1	GENCODE	1646	chr8	+
ENST00000539975	SNHG1	GENCODE	955	chr11	-
ENST00000539975	SNHG1	GENCODE	955	chr11	-
ENST00000539975	SNHG1	GENCODE	955	chr11	-
uc004aut.1	BC080653	UCSC_knowngene	1212	chr9	+
ENST00000534398	RP11-429J17.6	GENCODE	582	chr8	+
ENST00000557232	AE000662.93	GENCODE	386	chr14	-
uc003ttf.3	BC053669	UCSC_knowngene	2761	chr7	+
uc003ttf.3	BC053669	UCSC_knowngene	2761	chr7	+
NR_024405	LOC730101	RefSeq	3554	chr6	+
AA731927		LincRNAs identified	270	chr2	+
NR_039988	LOC100130899	RefSeq	4246	chr22	+
ENST00000453206	AC016751.3	GENCODE	280	chr2	+
ENST00000429588	AP000695.6	GENCODE	893	chr21	+
ENST00000429588	AP000695.6	GENCODE	893	chr21	+
ENST00000429588	AP000695.6	GENCODE	893	chr21	+
ENST00000429588	AP000695.6	GENCODE	893	chr21	+
ENST00000429588	AP000695.6	GENCODE	893	chr21	+
ENST00000545920	SNHG1	GENCODE	625	chr11	-
ENST00000541385	LINC00525	GENCODE	1173	chr7	+
ENST00000424434	RP1-309F20.3	GENCODE	367	chr20	-
ENST00000424434	RP1-309F20.3	GENCODE	367	chr20	-
ENST00000424434	RP1-309F20.3	GENCODE	367	chr20	-
TCONS_00026565	XLOC_012884	LincRNAs identified	563	chr18	-
ENST00000460977	RP11-383G6.3	GENCODE	765	chr3	-
NR_033827	KDM4A-AS1	RefSeq	1574	chr1	-
ENST00000508266	LEF1-AS1	GENCODE	559	chr4	+
TCONS_00004043	XLOC_001882	LincRNAs identified	796	chr2	+
ENST00000557546	RP11-566J3.2	GENCODE	722	chr14	-
ENST00000559959	RP11-16O9.2	GENCODE	476	chr15	+
ENST00000559959	RP11-16O9.2	GENCODE	476	chr15	+
ENST00000559959	RP11-16O9.2	GENCODE	476	chr15	+
ENST00000559959	RP11-16O9.2	GENCODE	476	chr15	+
ENST00000559959	RP11-16O9.2	GENCODE	476	chr15	+
ENST00000580458	SNHG15	GENCODE	710	chr7	-
ENST00000542331	RP11-443D10.2	GENCODE	560	chr12	+
ENST00000542331	RP11-443D10.2	GENCODE	560	chr12	+
ENST00000414762	RP11-96L14.7	GENCODE	447	chr1	-
ENST00000566551	RP5-1057J7.6	GENCODE	5444	chr1	-
ENST00000538231	RP11-392P7.6	GENCODE	457	chr12	+
ENST00000517655	KB-1047C11.2	GENCODE	562	chr8	+
ENST00000557691	RP6-65G23.3	GENCODE	629	chr14	+
ENST00000557691	RP6-65G23.3	GENCODE	629	chr14	+
TCONS_00016396	XLOC_007765	LincRNAs identified	936	chr9	-
chr2:102247075-102	chr2:102247075-102	LincRNAs identified	13675	chr2	+
ENST00000521133	CTB-131B5.4	GENCODE	454	chr5	-
uc002xig.3	LOC388796	UCSC_knowngene	2149	chr20	-
uc003wmd.1	AX747269	UCSC_knowngene	2946	chr7	-
ENST00000411909	AZGP1P1	pseudogene	902	chr7	+
ENST00000569034	RP11-15N24.4	GENCODE	2164	chr3	+
uc003ysb.3	BC106081	UCSC_knowngene	574	chr8	-
uc010bme.2	LOC727849	UCSC_knowngene	2447	chr15	+

41399565	41402288		ATGGGTATGCACATTAAGGCTTAGG		natural antisense
52233846	52234850		AGGTGTGAGGCTGCGTTGTGTGCTA		intergenic
134070693	134075403		TGTTTGGCTCCGCCTCTCTCCATGTC		exon sense-overlappi
23579993	23580912		CTAGCTAAATTAACATGTCATCATT(		intron sense-overlapp
123731639	123736348		TGTTTATACCCCAGAATTCTCTCCTC		intergenic
50065414	50081390		GAGACACTCCCT(	122769	exon sense-overlappi
50065414	50081390		GAGACACTCCCT(	122769	exon sense-overlappi
16190548	16225923		GCAATTTGCCCTTGAATCTTCCATA(		intergenic
194017423	194030593		AACAGAACAGAT.	100131551	intergenic
30677314	30683005		CTTCATCATGTAACTCTTGAATAAA.		intergenic
30209388	30211034		CGCTGCTCCAGCATCTAGGGCATAT		intergenic
62619491	62622708		AGTAATGTTCCA(	23642	bidirectional
62619491	62622708		AGTAATGTTCCA(	23642	bidirectional
62619491	62622708		AGTAATGTTCCA(	23642	bidirectional
97317422	97330409		AGTTTTGGGATAGATAGAGGGTCCT		natural antisense
144821565	144824305		GCCCCCACAGGGACACCTGGTCGCC		intergenic
23025204	23025956		GAAGTGGGAGACTTTTTCCACTTCC		natural antisense
64141510	64147264		ACGGGTTTCTGTTCCAACTGGTTGA.		intron sense-overlapp
64141510	64147264		ACGGGTTTCTGTTCCAACTGGTTGA.		intron sense-overlapp
52529198	52533951		TGAGGAGAAGAT	730101	intergenic
174255520	174255791		GGAAATTCTATGTTGATTAGGAAAA		intergenic
40428335	40432581		CCCACAACCTTT(	100130899	intergenic
176762063	176768960		GGGGGAAAATAGCCTCTCTGTTTGC		intergenic
37802657	37853368		AAATGTTATTGATGGAGGATTGACA		intronic antisense
37802657	37853368		AAATGTTATTGATGGAGGATTGACA		intronic antisense
37802657	37853368		AAATGTTATTGATGGAGGATTGACA		intronic antisense
37802657	37853368		AAATGTTATTGATGGAGGATTGACA		intronic antisense
37802657	37853368		AAATGTTATTGATGGAGGATTGACA		intronic antisense
62620543	62621814		CTAATTGAATGA.	23642	intergenic
47802946	47806369	ENST00000412730	TATAAACAACAC.	84847	intergenic
57456804	57463864		GACTGAGGAAGCTCCATTTTCTTTG.		intronic antisense
57456804	57463864		GACTGAGGAAGCTCCATTTTCTTTG.		intronic antisense
57456804	57463864		GACTGAGGAAGCTCCATTTTCTTTG.		intronic antisense
72824304	72837517		ATGCTATTCTCGGTTTCCCCAATAA(		intergenic
142184156	142191179		GAAGGGGGGTGTCTAAATTTGAATA		intron sense-overlapp
44165408	44173012		ACCTAAACAGTG.	100132774	natural antisense
109092926	109094924		TTTGGGAACAAA(	641518	intergenic
224904214	224907185		TCTTGTTTCCTCTCTTGGAAGAAGT(		intergenic
101142266	101146221		CAAAATATTCTCCTTTCTCTCCCTGT		intergenic
41635277	41636423		AAGATTTTTGATAAGGCATGACTCC		intron sense-overlapp
41635277	41636423		AAGATTTTTGATAAGGCATGACTCC		intron sense-overlapp
41635277	41636423		AAGATTTTTGATAAGGCATGACTCC		intron sense-overlapp
41635277	41636423		AAGATTTTTGATAAGGCATGACTCC		intron sense-overlapp
41635277	41636423		AAGATTTTTGATAAGGCATGACTCC		intron sense-overlapp
45022632	45026249	ENST00000584686,	TTCCTGAGCCCA(	285958	intergenic
109541771	109545809		GCTTCCACTCTTTCTCTTAAAGGAA(		intron sense-overlapp
109541771	109545809		GCTTCCACTCTTTCTCTTAAAGGAA(		intron sense-overlapp
26496670	26497304		TCTTCAGGCCATGTCAGGTAGAGGT		natural antisense
23607801	23613245		GGTAGGTAATTTACTTTCTATTGTA(		intergenic
13100098	13101511		GTATTTATCTGAACAGGATTTAATT.		intronic antisense
96216683	96444894		ACTTTTGTTAGGTCCATTTTCACTTC		intronic antisense
71277046	71282120		CGAGATAGGAGGCCCATAATGTTTC		bidirectional
71277046	71282120		CGAGATAGGAGGCCCATAATGTTTC		bidirectional
90892188	90893536		ATTGGAACACGAGCATTTCCTCTCC		intergenic
102880643	102894318		TCACAATTATTATAGATACTACCTG(		intergenic
139485009	139488173	ENST00000520928	TAATTTTATGTATTTATAGTAGTTCT		intergenic
37049238	37063962		CTTTCTGTTAGGA	388796	intergenic
155434167	155437113		AAATGGTCTCTATTAAAAAGCACGT		exon sense-overlappi
99578384	99585158		ATGATGGAAAGG	646282	
99898410	99900574		GTGGTAGAAAGAGCATCAAGTGAA1		intergenic
128240803	128241377	TCONS_00015171	GTTAATACTATGTAACCCCAGTGTT		intergenic
82944794	82975798	ENST00000335068,	AGCGGGTAGAGA	727849	intergenic

NM_032336	GINS4	DNA replication con	+	41386724	41402565
NM_016201	AMOTL2	angiomotin-like prote	-	134074189	134093406
NM_019116	UBFD1	ubiquitin domain-cor	+	23568861	23585710
NM_152329	LRR1	leucine-rich repeat pı	+	50065414	50081390
NM_203467	LRR1	leucine-rich repeat pı	+	50065414	50081390
NM_001012662	SLC3A2	4F2 cell-surface anti	+	62623483	62656355
NM_001012664	SLC3A2	4F2 cell-surface anti	+	62623483	62656355
NM_002394	SLC3A2	4F2 cell-surface anti	+	62623483	62656355
NM_003837	FBP2	fructose-1,6-bisphos	-	97321002	97356075
ENST00000557595	AE000662.92	Uncharacterized prot	+	23025533	23027939
NM_001013746	ZNF107	zinc finger protein 1(	+	64126510	64171401
NM_016220	ZNF107	zinc finger protein 1(	+	64126510	64171401
NM_001146077	CLDN14	claudin-14	-	37832919	37948867
NM_001146078	CLDN14	claudin-14	-	37832919	37915117
NM_001146079	CLDN14	claudin-14	-	37832919	37852388
NM_012130	CLDN14	claudin-14	-	37832919	37838739
NM_144492	CLDN14	claudin-14	-	37832919	37852388
NM_001077490	GNAS	protein ALEX alex	+	57428035	57486250
NM_016592	GNAS	protein ALEX NESP	+	57414794	57486250
NM_080425	GNAS	protein ALEX XLas	+	57428035	57486250
NM_001184	ATR	serine/threonine-prot	-	142168076	142297668
NM_014663	KDM4A	lysine-specific demet	+	44115796	44171189
NM_001243142	NUSAP1	nucleolar and spindle	+	41624891	41673248
NM_001243143	NUSAP1	nucleolar and spindle	+	41624891	41673248
NM_001243144	NUSAP1	nucleolar and spindle	+	41624891	41673248
NM_016359	NUSAP1	nucleolar and spindle	+	41624891	41673248
NM_018454	NUSAP1	nucleolar and spindle	+	41624891	41673248
NM_003362	UNG	uracil-DNA glycosyl	+	109535922	109548798
NM_080911	UNG	uracil-DNA glycosyl	+	109535398	109548798
NM_015871	ZNF593	zinc finger protein 5(	+	26496387	26497364
NM_018654	GPRC5D	G-protein coupled re	-	13093708	13103318
NM_177965	C8orf37	protein C8orf37	-	96257140	96281462
ENST00000381250	MAP3K9	mitogen-activated pr	-	71196652	71276223
ENST00000555993	MAP3K9	mitogen-activated pr	-	71196367	71276251
ENST00000401694	AC009403.2	Uncharacterized prot	-	155403924	155437095

38.346584	5.421924	4.874714	2.560675	42.459198	39.224148
101.276245	26.176928	6.163773	4.95441	116.352974	157.01756
948.597348	271.151282	9.482673	8.321261	950.9462	790.03326
225.249573	68.273962	7.446281	6.392155	209.50456	187.21826
45.778558	11.386348	5.173988	3.447996	53.87906	41.580105
82.129146	22.172709	5.955969	4.681843	78.64638	60.25638
82.129146	22.172709	5.955969	4.681843	78.64638	60.25638
69.636726	21.954134	5.719344	4.529278	107.980804	81.9307
30.633928	6.496018	4.483418	2.830249	33.593613	13.687741
390.41185	46.369483	8.193381	5.788982	473.26617	429.57297
161.32528	38.447592	6.915627	5.583046	193.2512	216.84372
694.513735	144.442114	9.02138	7.500983	616.2554	535.3189
694.513735	144.442114	9.02138	7.500983	616.2554	535.3189
694.513735	144.442114	9.02138	7.500983	616.2554	535.3189
28.188751	5.106032	4.42453	2.474615	39.6495	24.88094
70.817035	14.038543	5.784984	4.040391	70.150826	74.297775
83.882273	17.8035	5.859638	4.305734	67.799034	21.56316
134.152983	49.222924	6.664172	5.568366	105.58398	78.981895
134.152983	49.222924	6.664172	5.568366	105.58398	78.981895
41.592472	12.434637	4.81124	3.710345	62.898933	81.01735
326.523374	40.942751	7.549849	5.134946	141.9045	51.098263
8357.976417	2414.464542	12.420485	11.052931	11182.865	4587.784
30.261497	7.478578	4.395568	3.15563	36.548855	31.95807
43.349562	7.609044	4.789141	3.083413	28.387394	6.421906
43.349562	7.609044	4.789141	3.083413	28.387394	6.421906
43.349562	7.609044	4.789141	3.083413	28.387394	6.421906
43.349562	7.609044	4.789141	3.083413	28.387394	6.421906
43.349562	7.609044	4.789141	3.083413	28.387394	6.421906
273.900922	66.240235	7.687612	6.303717	249.65894	203.15112
342.749572	74.790451	7.85138	6.490231	453.0212	208.04018
62.917038	9.800069	5.331793	3.536598	102.2072	21.943314
62.917038	9.800069	5.331793	3.536598	102.2072	21.943314
62.917038	9.800069	5.331793	3.536598	102.2072	21.943314
25.476924	8.268826	4.290323	3.187557	31.348707	26.133095
31.232662	8.543466	4.642914	3.285629	31.923254	24.571737
112.239556	35.344869	6.429165	5.299117	130.92502	108.21962
39.137176	11.688611	4.817162	3.644786	36.62326	21.82868
23.02673	8.23886	4.195821	3.151817	29.876583	22.012758
30.963471	10.03631	4.605071	3.588078	24.369322	26.916548
26.778072	6.984352	4.392765	2.873021	25.935007	35.378353
26.778072	6.984352	4.392765	2.873021	25.935007	35.378353
26.778072	6.984352	4.392765	2.873021	25.935007	35.378353
26.778072	6.984352	4.392765	2.873021	25.935007	35.378353
26.778072	6.984352	4.392765	2.873021	25.935007	35.378353
260.67223	58.786771	7.608777	6.206182	311.0595	377.47165
74.539217	21.757548	5.849956	4.698504	77.90099	92.87605
74.539217	21.757548	5.849956	4.698504	77.90099	92.87605
89.388226	16.608442	6.027199	4.32259	107.283585	164.75966
3179.451483	831.578155	11.152118	9.916348	3043.7583	2180.9128
44.520409	14.029717	5.025135	3.792019	45.789955	15.547912
455.803557	122.27024	8.420571	7.098767	586.08276	596.9191
259.009762	57.567185	7.637078	6.064982	258.89716	303.33902
259.009762	57.567185	7.637078	6.064982	258.89716	303.33902
23.283252	8.138231	4.192989	3.095847	24.955187	26.20568
20.826361	7.521871	4.017279	2.945373	29.357594	14.740099
1223.757463	267.476228	9.623347	8.363214	964.2818	429.96133
214.94165	50.373761	7.348822	5.972284	276.95325	307.85
669.159053	183.540222	8.855723	7.579672	453.6928	464.10175
1699.473867	216.327574	10.144627	7.984329	2197.2268	3286.032
24.435327	7.604752	4.244947	3.013983	19.94159	11.958449
28.480569	7.483895	4.421781	2.967859	30.952795	13.26432
196.90687	46.251555	7.087461	5.800474	203.5338	305.4457

22.39445	29.058714	46.488552	50.454445	4.9999995	4.9999995
42.362045	55.407097	123.162796	113.354996	37.56748	30.20021
939.5225	1132.9319	761.09753	1117.0527	218.34827	140.71465
215.40692	267.31552	192.20558	279.8466	71.3929	38.599205
52.731182	36.137543	43.91659	46.42687	16.591978	6.437701
61.006702	131.76216	55.060783	106.04247	15.64878	11.521231
61.006702	131.76216	55.060783	106.04247	15.64878	11.521231
67.0017	43.769245	54.28577	62.852135	29.17035	13.307381
46.66903	43.006596	12.689699	34.15689	4.9999995	6.9926248
475.91742	355.5979	239.13388	368.98276	48.71495	25.362728
125.89578	87.21835	179.48685	165.25578	32.732574	29.770746
650.9867	560.1162	854.59985	949.80536	153.5967	123.8743
650.9867	560.1162	854.59985	949.80536	153.5967	123.8743
650.9867	560.1162	854.59985	949.80536	153.5967	123.8743
33.995865	14.653258	25.068352	30.884592	5.327371	4.9999995
54.26663	60.394333	79.522415	86.27023	17.153742	9.348891
96.71833	147.0938	61.722385	108.39693	16.036451	7.6816006
111.28779	184.37915	140.39117	184.29391	17.319656	14.878519
111.28779	184.37915	140.39117	184.29391	17.319656	14.878519
34.424923	15.585321	24.35741	31.270897	13.51183	8.230274
242.79268	263.3071	674.1781	585.8596	39.807228	5.368586
16788.502	7489.656	3368.6875	6730.364	2800.4106	220.56935
12.077791	58.497173	11.397339	31.089756	7.061759	7.217812
46.956615	34.00279	75.258804	69.06986	10.31078	5.5131097
46.956615	34.00279	75.258804	69.06986	10.31078	5.5131097
46.956615	34.00279	75.258804	69.06986	10.31078	5.5131097
46.956615	34.00279	75.258804	69.06986	10.31078	5.5131097
46.956615	34.00279	75.258804	69.06986	10.31078	5.5131097
254.99585	199.90984	360.14798	375.5418	58.700558	49.772457
660.2279	367.86975	122.92342	244.41498	80.814545	41.42485
140.79819	37.404602	26.981482	48.16744	12.280589	7.203097
140.79819	37.404602	26.981482	48.16744	12.280589	7.203097
140.79819	37.404602	26.981482	48.16744	12.280589	7.203097
30.546158	35.528893	16.895689	12.409001	8.747339	5.928433
29.728642	42.371017	26.821178	31.980143	11.64052	9.451308
153.6301	94.133316	76.50125	110.02803	40.000526	11.87989
35.80666	85.3475	18.641802	36.575153	11.586388	4.9999995
17.932817	21.26649	23.006386	24.065348	9.883603	4.9999995
25.48452	47.508026	24.72563	36.77678	11.451948	7.8827586
16.991789	27.205101	24.363644	30.794537	7.754792	4.9999995
16.991789	27.205101	24.363644	30.794537	7.754792	4.9999995
16.991789	27.205101	24.363644	30.794537	7.754792	4.9999995
16.991789	27.205101	24.363644	30.794537	7.754792	4.9999995
16.991789	27.205101	24.363644	30.794537	7.754792	4.9999995
259.8496	252.8894	147.25548	215.50775	55.057762	69.13863
54.991272	71.91038	58.215515	91.341095	23.94478	14.00783
54.991272	71.91038	58.215515	91.341095	23.94478	14.00783
60.659023	61.773716	60.14555	81.70782	19.35994	15.036251
3565.5798	4561.8433	1914.1027	3810.512	985.9344	291.33533
65.346855	31.642105	53.493618	55.302006	15.479142	4.9999995
415.14035	365.02063	418.8744	352.7841	121.71369	54.331238
187.15033	218.76486	291.4744	294.4328	44.181477	28.136223
187.15033	218.76486	291.4744	294.4328	44.181477	28.136223
23.997967	30.977787	21.205189	12.3577	10.18545	4.9999995
19.538168	31.82493	13.169019	16.328358	8.349351	4.9999995
1123.7784	593.54395	2262.4993	1968.48	212.35837	137.04628
190.13249	175.28676	156.46242	182.96498	41.684006	40.99561
401.1977	1168.884	463.40067	1063.6774	193.05197	38.385315
1050.5646	674.5365	1517.9738	1470.5095	341.87225	328.78833
23.213627	35.133667	25.014297	31.350334	8.804812	4.9999995
39.239876	39.543625	14.743429	33.139366	13.249079	4.9999995
84.63601	87.40299	262.17834	238.24438	48.47775	28.890148

5.636218	6.0024424	4.9999995	5.892883	4.9473968	5.190097
27.936485	27.833132	12.982691	20.54157	6.4086394	7.0560155
280.1822	533.06635	167.58322	287.013	9.423069	9.293261
91.7607	77.67977	64.84669	65.36451	7.2576933	7.307643
28.1722	4.9999995	5.828055	6.288156	5.2838044	5.2682505
25.62019	23.64221	36.493366	20.110477	5.8434753	5.751268
25.62019	23.64221	36.493366	20.110477	5.8434753	5.751268
46.67867	9.450924	18.5047	14.61278	6.3068833	6.162426
4.9999995	11.527129	4.9999995	5.456355	4.6079326	3.8112924
56.77139	38.261692	70.36492	38.74122	8.40955	8.436878
42.993107	46.132587	40.48604	38.5705	7.1425095	7.502681
153.92876	109.223694	195.91153	130.1177	8.794905	8.754577
153.92876	109.223694	195.91153	130.1177	8.794905	8.754577
153.92876	109.223694	195.91153	130.1177	8.794905	8.754577
4.9999995	4.9999995	5.3088217	4.9999995	4.8487864	4.5926166
13.315861	23.212875	10.058029	11.14186	5.673632	6.030472
18.971788	35.13352	11.163542	17.8341	5.6234236	4.394413
31.620098	126.38692	36.201153	68.9312	6.2756343	6.113735
31.620098	126.38692	36.201153	68.9312	6.2756343	6.113735
24.320423	4.9999995	15.610723	7.9345737	5.5185785	6.146406
76.763374	12.78853	74.30777	36.62102	6.6983423	5.541128
5741.011	874.9513	3217.5818	1632.2632	12.999295	11.726
11.377839	5.787665	5.6066055	7.8197904	4.726695	4.925186
4.9999995	10.784961	4.9999995	9.045415	4.3749185	2.852024
4.9999995	10.784961	4.9999995	9.045415	4.3749185	2.852024
4.9999995	10.784961	4.9999995	9.045415	4.3749185	2.852024
4.9999995	10.784961	4.9999995	9.045415	4.3749185	2.852024
4.9999995	10.784961	4.9999995	9.045415	4.3749185	2.852024
68.193634	43.53294	117.759	59.48282	7.505351	7.4143014
121.30317	65.941864	77.62852	61.629757	8.347784	7.448058
8.500926	6.378087	13.411603	11.02611	6.228389	4.4207354
8.500926	6.378087	13.411603	11.02611	6.228389	4.4207354
8.500926	6.378087	13.411603	11.02611	6.228389	4.4207354
14.49966	5.5656476	4.9999995	9.871879	4.513104	4.6576047
4.9999995	4.9999995	10.38666	9.782307	4.5376577	4.575687
55.800827	25.587566	46.44363	32.356777	6.584625	6.539441
16.252779	12.080541	11.372201	13.839759	4.729383	4.412583
10.70723	6.3083735	11.284829	6.249128	4.447179	4.4252086
11.023401	12.62894	10.216929	7.013884	4.150207	4.6980534
12.005489	6.318772	5.827062	4.9999995	4.2387695	5.060999
12.005489	6.318772	5.827062	4.9999995	4.2387695	5.060999
12.005489	6.318772	5.827062	4.9999995	4.2387695	5.060999
12.005489	6.318772	5.827062	4.9999995	4.2387695	5.060999
12.005489	6.318772	5.827062	4.9999995	4.2387695	5.060999
41.37767	70.99175	54.433243	61.721573	7.8253846	8.267057
26.533016	16.976227	29.973701	19.109732	5.8314757	6.3266964
26.533016	16.976227	29.973701	19.109732	5.8314757	6.3266964
18.518906	17.373688	19.06758	10.294288	6.300123	7.1228595
1457.1542	537.0929	1080.4556	637.4965	11.090976	10.6769705
28.289206	14.00904	12.40685	8.994067	5.056939	3.978356
269.49377	94.09023	91.67079	102.32172	8.7197895	8.908517
49.66167	48.81603	105.51278	69.09493	7.5604796	7.9744473
49.66167	48.81603	105.51278	69.09493	7.5604796	7.9744473
14.394659	8.159953	5.433492	5.655833	4.1814556	4.661509
9.137138	4.9999995	4.9999995	12.644738	4.420042	3.9064555
262.4511	297.4592	417.55652	277.9859	9.439632	8.438746
49.286	55.195347	68.921745	46.159855	7.6626673	7.9930224
402.35672	146.72122	184.4818	136.24431	8.349809	8.549073
214.52908	180.15494	119.01688	113.603966	10.6263275	11.261673
4.9999995	13.129539	6.5129147	7.1812487	3.8582156	3.636282
7.46981	6.1250443	8.059437	4.9999995	4.4944496	3.7672367
66.10664	39.7824	58.33989	35.912502	7.215641	7.984416

4.1713567	4.402366	5.3701468	5.1669183	2.3467326	2.4279919
5.0835905	5.408334	6.724544	6.3015146	5.467793	5.755429
9.506867	9.822046	9.311349	9.539445	8.018646	7.9854956
7.4336886	7.7448225	7.3527207	7.581115	6.3903785	6.111639
5.4017715	4.7421403	5.2964544	5.051507	4.284698	3.438599
5.621011	6.711897	5.5984983	6.209666	4.197416	4.315952
5.621011	6.711897	5.5984983	6.209666	4.197416	4.315952
5.758358	5.042064	5.579201	5.467133	5.096626	4.5372353
5.225091	5.014564	3.6059313	4.6356993	2.3467326	3.568541
8.545436	8.1492	7.6592255	7.9599986	5.840043	5.498396
6.65241	6.0924406	7.2588835	6.844835	5.264597	5.736408
8.981791	8.806364	9.480629	9.310017	7.5035105	7.801304
8.981791	8.806364	9.480629	9.310017	7.5035105	7.801304
8.981791	8.806364	9.480629	9.310017	7.5035105	7.801304
4.772266	3.30342	4.5342627	4.4958277	2.622869	2.4279919
5.44487	5.538499	6.110455	5.911976	4.337932	3.9997532
6.272367	6.8736258	5.7547174	6.239282	4.2347283	3.7014015
6.479357	7.2044687	6.9128895	6.998948	4.3526316	4.7061467
6.479357	7.2044687	6.9128895	6.998948	4.3526316	4.7061467
4.79185	3.4041388	4.493492	4.512973	3.984622	3.8092117
7.595459	7.725825	9.130125	8.608215	5.5509915	3.1578577
13.763353	12.518451	11.422207	12.093601	11.768271	8.6404
3.26634	5.4898167	3.460215	4.505154	3.0137167	3.6135645
5.2346973	4.646934	6.0281043	5.598165	3.592714	3.2001655
5.2346973	4.646934	6.0281043	5.598165	3.592714	3.2001655
5.2346973	4.646934	6.0281043	5.598165	3.592714	3.2001655
5.2346973	4.646934	6.0281043	5.598165	3.592714	3.2001655
5.2346973	4.646934	6.0281043	5.598165	3.592714	3.2001655
7.6596923	7.324397	8.236217	7.985715	6.1112947	6.4845138
9.001366	8.202094	6.7210536	7.387924	6.560937	6.2177825
6.813902	4.794995	4.6309633	5.1017733	3.8468306	3.6107388
6.813902	4.794995	4.6309633	5.1017733	3.8468306	3.6107388
6.813902	4.794995	4.6309633	5.1017733	3.8468306	3.6107388
4.625408	4.715929	3.9972217	3.2326677	3.339285	3.3143082
4.58448	4.991452	4.624325	4.543882	3.7698262	4.0166245
6.9314837	6.204856	6.052959	6.2616277	5.55792	4.360355
4.8454013	6.0602655	4.1275835	4.7277565	3.7632835	2.4279919
3.8473296	3.9040053	4.414177	4.1370273	3.5271208	2.4279919
4.360154	5.170717	4.5153484	4.7359467	3.747278	3.7405472
3.7680078	4.3030634	4.4942245	4.491528	3.1513758	2.4279919
3.7680078	4.3030634	4.4942245	4.491528	3.1513758	2.4279919
3.7680078	4.3030634	4.4942245	4.491528	3.1513758	2.4279919
3.7680078	4.3030634	4.4942245	4.491528	3.1513758	2.4279919
3.7680078	4.3030634	4.4942245	4.491528	3.1513758	2.4279919
7.688475	7.673708	6.9811416	7.216898	6.0179925	6.9610286
5.46651	5.8058276	5.675357	5.9938703	4.8245826	4.6186566
5.46651	5.8058276	5.675357	5.9938703	4.8245826	4.6186566
5.6122036	5.5730724	5.720251	5.834682	4.5112634	4.7243066
11.436981	11.8123665	10.60498	11.290435	10.260273	9.0494795
5.723614	4.5380025	5.55721	5.296691	4.1844497	2.4279919
8.354368	8.190673	8.448782	7.901298	7.1600614	6.6135163
7.226626	7.461958	7.9444704	7.654488	5.698433	5.6508336
7.226626	7.461958	7.9444704	7.654488	5.698433	5.6508336
4.278139	4.5076456	4.302038	3.227144	3.5741935	2.4279919
3.9668114	4.5466657	3.6559534	3.6077442	3.267405	2.4279919
9.75942	8.893606	10.850326	10.35835	7.9744473	7.9464073
7.249302	7.1291246	7.0700097	6.9888043	5.6152544	6.202302
8.299586	9.872683	8.596709	9.466481	7.83819	6.104165
9.662657	9.091095	10.295151	9.930856	8.683072	9.220763
4.2283254	4.7000003	4.5312595	4.5156016	3.350052	2.4279919
4.9789333	4.8841896	3.8108997	4.59498	3.9552395	2.4279919
6.0864935	6.094824	7.791039	7.3523517	5.833062	5.6912537

2.715229	2.6932654	2.3618152	2.8190138
4.8254213	5.001877	3.9755201	4.700419
8.075413	9.518299	7.627012	8.702703
6.4850082	6.604062	6.2869625	6.474881
4.8372645	2.3408759	2.8717132	2.9148262
4.702451	4.7459803	5.4637485	4.665508
4.702451	4.7459803	5.4637485	4.665508
5.5438776	3.3375847	4.477513	4.182831
2.350709	3.639562	2.3618152	2.714133
5.816333	5.4927983	6.4008884	5.685433
5.421285	5.7849503	5.611554	5.679484
7.214459	7.113854	7.8606377	7.51213
7.214459	7.113854	7.8606377	7.51213
7.214459	7.113854	7.8606377	7.51213
2.350709	2.3408759	2.7524924	2.352752
3.8096309	4.71585	3.6080103	3.7711673
4.295093	5.3637314	3.756932	4.482518
4.99756	7.349468	5.4526463	6.5517464
4.99756	7.349468	5.4526463	6.5517464
4.6291103	2.3408759	4.2364063	3.2618437
6.2306647	3.793397	6.4773726	5.599393
12.462249	10.25152	11.933474	11.261673
3.603805	2.6453981	2.8206	3.236695
2.350709	3.5373566	2.3618152	3.457717
2.350709	3.5373566	2.3618152	3.457717
2.350709	3.5373566	2.3618152	3.457717
2.350709	3.5373566	2.3618152	3.457717
2.350709	3.5373566	2.3618152	3.457717
6.069859	5.6892433	7.1297617	6.337627
6.881975	6.3487215	6.540354	6.391618
3.2137773	2.7745209	4.017525	3.756193
3.2137773	2.7745209	4.017525	3.756193
3.2137773	2.7745209	4.017525	3.756193
3.9239259	2.5957327	2.3618152	3.590277
2.350709	2.3408759	3.6582654	3.5774732
5.7922044	4.8677244	5.8065066	5.4099917
4.083551	3.7086086	3.7811992	4.104079
3.5202184	2.7601893	3.770302	2.9050775
3.5618496	3.774617	3.6313791	3.072796
3.6729438	2.7617857	2.8712776	2.352752
3.6729438	2.7617857	2.8712776	2.352752
3.6729438	2.7617857	2.8712776	2.352752
3.6729438	2.7617857	2.8712776	2.352752
3.6729438	2.7617857	2.8712776	2.352752
5.3677835	6.465655	6.031085	6.393545
4.7520566	4.2271276	5.182884	4.585715
4.7520566	4.2271276	5.182884	4.585715
4.2632356	4.262516	4.5175815	3.6566377
10.448406	9.529076	10.335355	9.875501
4.8438864	3.9367251	3.9091399	3.4499185
8.022054	6.8874693	6.7691593	7.140344
5.631933	5.87541	6.977507	6.555775
5.631933	5.87541	6.977507	6.555775
3.9140952	3.118308	2.7796426	2.7608507
3.3109074	2.3408759	2.3618152	3.9632397
7.983128	8.6677265	8.947453	8.660124
5.622583	6.0682335	6.373878	5.951454
8.608826	7.5716796	7.777078	7.578091
7.688899	7.8740253	7.145105	7.294107
2.350709	3.8357491	3.0129642	3.1064334
3.0482035	2.719315	3.303652	2.352752
6.024882	5.5517516	6.13182	5.570073

ASHGA5P028446	0.004421434	0.042188865	3.2467649	up	noncoding
ASHGA5P017214	0.029202051	0.106794485	2.5431592	up	noncoding
ASHGA5P051050	0.028383917	0.105047356	6.7641415	up	noncoding
ASHGA5P032626	0.002338349	0.031698104	3.2280776	up	noncoding
ASHGA5P037745	8.48172E-05	0.008738697	9.3699558	up	noncoding
ASHGA5P042602	0.000173619	0.011182026	4.2729367	up	noncoding
ASHGA5P028691	0.048801115	0.143530748	4.4745479	up	noncoding
ASHGA5P047753	0.049604516	0.144887709	2.094463	up	noncoding
ASHGA5P025795	0.02633863	0.101175177	2.3222656	up	noncoding
ASHGA5P025795	0.02633863	0.101175177	2.3222656	up	noncoding
ASHGA5P025795	0.02633863	0.101175177	2.3222656	up	noncoding
ASHGA5P020105	0.000287853	0.012492602	2.7145097	up	noncoding
ASHGA5P057588	0.002105691	0.03028922	2.4917643	up	noncoding
ASHGA5P017522	0.016281926	0.077996855	3.0939713	up	noncoding
ASHGA5P029487	0.003343273	0.037382889	2.2295682	up	noncoding
ASHGA5P018810	0.034280945	0.11696607	2.1755517	up	noncoding
ASHGA5P017279	0.019795538	0.085917997	2.2896627	up	noncoding
ASHGA5P017279	0.019795538	0.085917997	2.2896627	up	noncoding
ASHGA5P017279	0.019795538	0.085917997	2.2896627	up	noncoding
ASHGA5P026489	0.009040037	0.059313506	2.0415428	up	noncoding
ASHGA5P043753	0.010861536	0.064472676	2.1864096	up	noncoding
ASHGA5P043753	0.010861536	0.064472676	2.1864096	up	noncoding
ASHGA5P053561	0.000256899	0.012149764	4.08303	up	noncoding
ASHGA5P043890	0.001344929	0.024539537	3.2951689	up	noncoding
ASHGA5P022417	0.003707134	0.039054649	2.0690161	up	noncoding
ASHGA5P017022	0.000527702	0.015826428	3.784878	up	noncoding
ASHGA5P034858	0.034607499	0.117709021	2.3411737	up	noncoding
ASHGA5P053501	0.002815464	0.034198499	2.2536841	up	noncoding
ASHGA5P050711	0.013832748	0.07198363	4.0626928	up	noncoding
ASHGA5P020480	0.000593558	0.016411961	2.6207034	up	noncoding
ASHGA5P029745	0.000680506	0.017403699	2.9842402	up	noncoding
ASHGA5P033094	0.038926815	0.125823386	2.0480652	up	noncoding
ASHGA5P035741	0.005457199	0.046970992	3.0999763	up	noncoding
ASHGA5P019075	0.028958758	0.106241445	2.0828856	up	noncoding
ASHGA5P019075	0.028958758	0.106241445	2.0828856	up	noncoding
ASHGA5P022303	0.026519373	0.101666408	4.1805088	up	noncoding
ASHGA5P028530	0.048395801	0.142802321	3.0272709	up	noncoding
ASHGA5P046103	0.003150924	0.036407477	2.5872999	up	noncoding
ASHGA5P046935	0.001926203	0.029259988	4.4255155	up	noncoding
ASHGA5P027431	0.004445865	0.042328642	2.9086867	up	noncoding
ASHGA5P045031	2.33197E-06	0.00231943	6.830869	up	noncoding
ASHGA5P028942	0.04581485	0.13789067	2.8051289	up	noncoding
ASHGA5P025990	0.000109855	0.009555212	2.7017327	up	noncoding
ASHGA5P025990	0.000109855	0.009555212	2.7017327	up	noncoding
ASHGA5P051881	0.006124007	0.049942924	2.8350782	up	noncoding
ASHGA5P023526	0.001093341	0.022343136	2.5811847	up	noncoding
ASHGA5P032428	0.00818838	0.056791621	2.0399259	up	noncoding
ASHGA5P022831	0.004450435	0.04234886	4.0517582	up	noncoding
ASHGA5P045066	0.033383282	0.115144552	3.3549586	up	noncoding
ASHGA5P057743	0.004677609	0.043513602	2.2120224	up	noncoding
ASHGA5P045032	0.000101021	0.009142535	2.1340762	up	noncoding
ASHGA5P019079	0.016746069	0.078875667	2.0789575	up	noncoding
ASHGA5P019079	0.016746069	0.078875667	2.0789575	up	noncoding
ASHGA5P018367	0.012112781	0.067436516	2.3783779	up	noncoding
ASHGA5P018367	0.012112781	0.067436516	2.3783779	up	noncoding
ASHGA5P037802	0.040856691	0.129143254	2.0679495	up	noncoding
ASHGA5P037802	0.040856691	0.129143254	2.0679495	up	noncoding
ASHGA5P037802	0.040856691	0.129143254	2.0679495	up	noncoding
ASHGA5P037802	0.040856691	0.129143254	2.0679495	up	noncoding
ASHGA5P042471	0.009147914	0.059552152	3.5379627	up	noncoding
ASHGA5P055865	0.001132704	0.022643498	2.2042473	up	noncoding
ASHGA5P055865	0.001132704	0.022643498	2.2042473	up	noncoding

ENST00000444744	RP11-428O18.4	GENCODE	420	chr13	-
ENST00000438934	DGCR5	GENCODE	5492	chr22	+
uc002ybl.2	AX747739	UCSC_knowngene	2054	chr20	+
ENST00000533232	AC005152.2	GENCODE	1739	chr17	-
ENST00000422971	CTA-941F9.9	GENCODE	548	chr22	-
TCONS_00014546	XLOC_006923	LincRNAs identified	4060	chr8	+
NR_047495	LINC00565	RefSeq	2494	chr13	-
ENST00000448347	RP11-282I1.1	GENCODE	831	chr10	+
NR_037930	SLMO2-ATP5E	RefSeq	768	chr20	-
NR_037930	SLMO2-ATP5E	RefSeq	768	chr20	-
NR_037930	SLMO2-ATP5E	RefSeq	768	chr20	-
ENST00000508286	LEF1-AS1	GENCODE	444	chr4	+
TCONS_00019456	XLOC_009265	LincRNAs identified	639	chr11	+
ENST00000442687	AC034228.7	pseudogene	1578	chr5	-
ENST00000555350	RP11-572M18.1	GENCODE	470	chr14	+
ENST00000457870	AC016700.3	GENCODE	501	chr2	-
ENST00000439622	RP3-323P24.3	GENCODE	665	chrX	+
ENST00000439622	RP3-323P24.3	GENCODE	665	chrX	+
ENST00000439622	RP3-323P24.3	GENCODE	665	chrX	+
CN412465		LincRNAs identified	563	chr6	-
uc003ufr.3	BC090058	UCSC_knowngene	1983	chr7	+
uc003ufr.3	BC090058	UCSC_knowngene	1983	chr7	+
ENST00000517790	PVT1	GENCODE	599	chr8	+
ENST00000478818	RP11-155G14.5	pseudogene	450	chr7	+
ENST00000556361	RP11-72M17.1	GENCODE	442	chr14	-
ENST00000436764	RP4-564F22.2	GENCODE	973	chr20	-
ENST00000450467	AC097517.2	GENCODE	358	chr2	-
ENST00000518613	KB-1608C10.2	GENCODE	1203	chr8	-
ENST00000447538	HOXD-AS1	GENCODE	558	chr2	-
ENST00000514368	RP11-434D9.1	GENCODE	756	chr5	+
ENST00000435624	C14orf63	GENCODE	448	chr14	+
ENST00000433734	RP5-1065P14.2	GENCODE	599	chr1	-
uc002rdd.2	FLJ12334	UCSC_knowngene	2509	chr2	+
NR_033201	HOXB-AS3	RefSeq	570	chr17	+
NR_033201	HOXB-AS3	RefSeq	570	chr17	+
ENST00000554149	RP11-1085N6.5	GENCODE	621	chr14	-
TCONS_00009837	XLOC_004253	LincRNAs identified	324	chr5	+
ENST00000438499	RP11-1M18.1	GENCODE	517	chrX	-
uc001jtm.3	BC069792	UCSC_knowngene	632	chr10	-
ENST00000546601	RP11-186F10.2	GENCODE	527	chr12	-
ENST00000522875	PVT1	GENCODE	922	chr8	+
ENST00000421423	LINC00403	GENCODE	545	chr13	+
NR_048557	TSN	RefSeq	3217	chr2	+
NR_048557	TSN	RefSeq	3217	chr2	+
ENST00000512092	FAM86EP	GENCODE	462	chr4	-
ENST00000583052	RP11-583F2.1	GENCODE	599	chr17	+
ENST00000560400	RP11-156E6.1	GENCODE	2930	chr17	-
ENST00000563602	RP11-44F21.5	GENCODE	345	chr4	-
ENST00000439341	RP5-858B6.3	GENCODE	794	chr1	+
TCONS_00021722	XLOC_010305	LincRNAs identified	206	chr13	+
ENST00000512617	PVT1	GENCODE	383	chr8	+
NR_024250	FAM86JP	RefSeq	1970	chr3	+
NR_024250	FAM86JP	RefSeq	1970	chr3	+
NR_033963	FLJ44511	RefSeq	3506	chr7	+
NR_033963	FLJ44511	RefSeq	3506	chr7	+
ENST00000431090	AC000068.5	GENCODE	2097	chr22	+
ENST00000431090	AC000068.5	GENCODE	2097	chr22	+
ENST00000431090	AC000068.5	GENCODE	2097	chr22	+
ENST00000431090	AC000068.5	GENCODE	2097	chr22	+
ENST00000448327	RP11-345L23.1	GENCODE	566	chr6	+
uc001vtl.1	AK126278	UCSC_knowngene	1939	chr13	+
uc001vtl.1	AK126278	UCSC_knowngene	1939	chr13	+

27810418	27810948		GGTTGTCACTGGAAGTTCCTGAAGT	intergenic
18958026	18982141		GAAGAAAAAGTGCTTAGGTAGAGG	intergenic
58713536	58897314	uc010gjw.2	TTTGCGTTTCAGATGCTCCCAGGCT(	intergenic
70076078	70116934		TAACTCACAACTTGGTGTCTCATAA(	bidirectional
46000311	46001501		TGTCCACACAAGACTTTAATAAAT(	intergenic
128938354	128943402		TGGGTTGTCATCACAGTTGACTTAG.	intergenic
114629486	114631964	TCONS_00022133	ACTTAGCTTCCCA 100861555	intergenic
125115965	125277224		GTTGCCAGAGTGAAATTCCCCAGAC	intergenic
57603732	57617901		TTCGGAGCACGT(100533975	exon sense-overlappi
57603732	57617901		TTCGGAGCACGT(100533975	exon sense-overlappi
57603732	57617901		TTCGGAGCACGT(100533975	exon sense-overlappi
109094574	109097586		GGTGGATATGTG(641518	intergenic
104071252	104112755		AGAGTGGAAGCATCTCTCCAATTTG	intergenic
131142923	131281198		GTGCTTCAAATTCAGAATCAGTTATAGATAATGCAGCT	
28103330	28105875		ATATTAATGTCTCAGTCCCGAGAAA	intergenic
70321028	70322126		GTGGTGGTCATGTGTTTGTCAACCT(	intergenic
57148094	57153676		GGAGTTGGAACAGAGAAATTCACTA	bidirectional
57148094	57153676		GGAGTTGGAACAGAGAAATTCACTA	bidirectional
57148094	57153676		GGAGTTGGAACAGAGAAATTCACTA	bidirectional
37103028	37103592		TAAAATTAGAACTGACTTTCAGTAC	intergenic
76178657	76182447		CTTTCTTATGTGACTCTAAAGGTCA	intron sense-overlapp
76178657	76182447		CTTTCTTATGTGACTCTAAAGGTCA	intron sense-overlapp
128807999	128903178		TCCTGTGGTCATA 5820	intergenic
128106616	128109225	NM_001144933, NM	CAAATGCTCAAGAAAAAGCAGACTTGTATAGTGGCCGA	
66679527	66963043		GGAGGAGGAATTCTTGGCTAAGAGT	intergenic
37049234	37063976	NR_015366, NR_02	ATCTGCGTGGGTCTCAGCCTTAGAT(	intergenic
6789237	6790375	ENST00000415657,	CTACATTTGCAGCATCTCCAAGTTT(	intergenic
95804510	95806064		CTGCCTATATACACAGAAAGTAAAT	natural antisense
177041107	177042812	ENST00000456876,	AGGGGCCCGGGG 401022	intergenic
66915618	67093432		GGAGCTTTCCCTTGTGAAGTGAAAG	intergenic
97409915	97411731		GAAACTCCATGTTACCAGCATGTTA	intergenic
229228561	229230652		CTTAGCGTGAGCCTGGGATATGCAT	intergenic
20068614	20077296	uc002rde.2	TGAAGAGTGAAA 400946	intergenic
46667822	46683774		TCCAGTAAGAAG 404266	intronic antisense
46667822	46683774		TCCAGTAAGAAG 404266	intronic antisense
57284287	57360066		AGAAGTTTGGCAGACACTTCCGACG	intergenic
2268284	2278578		GGAATTTGTTGAGGGAATTTGTATT	intergenic
6585905	6639585		ATTCTTTTCTGGGCAAATCTAAACCT	intergenic
74884658	74885290		GAACAACTCACAGAAAAATGCCAAT	natural antisense
71976072	71977632		CAAATTCCTTGTGCTCTAGCTTTCTT	natural antisense
129001506	129108902		GCCAACAGAGAT 5820	intergenic
112761432	112762329		TAGTGCAACCAA(100505996	intergenic
122513120	122525428	NR_048559	CATCCGAGAGAT 7247	exon sense-overlappi
122513120	122525428	NR_048559	CATCCGAGAGAT 7247	exon sense-overlappi
3949631	3956770		CTTTTTAAAGGCAGTAATCGTTCTG(	intergenic
62926266	62930321		GAACCTATGGATTGACCTTTGTAAA	intergenic
40004769	40007699		GTTTTATGTTCCTGAAGCTATCTTGT	intergenic
76007246	76008878		AGATTCTAAGGTGGAAAATGAAGCA	intergenic
231010591	231014761		CATTAGCCAAAGCTCCGGGACTTGG	intergenic
23507414	23508772		AGAAAAGCGGCCCCCACCTCTGCTG	intergenic
129001429	129082518	ENST00000521600	TTGTCAGTTAAGT 5820	intergenic
125635443	125648867	ENST00000427587,	ACCGAGCTAGGC(100125556	natural antisense
125635443	125648867	ENST00000427587,	ACCGAGCTAGGC(100125556	natural antisense
560027	564869		TGCAACCTCGAA 441307	bidirectional
560027	564869		TGCAACCTCGAA 441307	bidirectional
19435415	19437628		TTCACTAAGGAGCCAGTTATGTCCA	natural antisense
19435415	19437628		TTCACTAAGGAGCCAGTTATGTCCA	natural antisense
19435415	19437628		TTCACTAAGGAGCCAGTTATGTCCA	natural antisense
19435415	19437628		TTCACTAAGGAGCCAGTTATGTCCA	natural antisense
53493177	53496192		CGAAGCTTTTGAAGGTTTAAGGATC	intergenic
113888345	113890284		TGTTTGTAAGGTTCCAGAATATCTTA	exon sense-overlappi
113888345	113890284		TGTTTGTAAGGTTCCAGAATATCTTA	exon sense-overlappi

NM_000346	SOX9	transcription factor S	+	70117160	70122560
NM_001256403	SLMO2	protein slowmo homo	-	57608199	57617901
NM_006886	ATP5E	ATP synthase subuni	-	57603732	57607422
NM_016045	SLMO2	protein slowmo homo	-	57608199	57617901
TGTGAGATTTTACTTGCTGAGA					
NM_001006681	SPIN2B	spindlin-2B	-	57146114	57147979
NM_001006682	SPIN2B	spindlin-2B	-	57146114	57147979
NM_001006683	SPIN2B	spindlin-2B	-	57146114	57147979
ENST00000419923	UPK3B	uroplakin 3B [Source	+	76139744	76648340
ENST00000443097	UPK3B	uroplakin 3B [Source	+	76139744	76648340
ATGCAACTACTATTAAACAACAT					
NM_181787	DPY19L4	protein dpy-19 homo	+	95732102	95806076
NM_002147	HOXB5	homeobox protein Ho	-	46668618	46671103
NM_018952	HOXB6	homeobox protein Ho	-	46673098	46682334
NM_015901	NUDT13	nucleoside diphospha	+	74870209	74891581
NM_003667	LGR5	leucine-rich repeat-co	+	71833812	71978621
NM_001261401	TSN	translin isoform 2	+	122513120	122525428
NM_004622	TSN	translin isoform 1	+	122513120	122525428
NM_001015050	ALG1L	putative glycosyltran	-	125648117	125655887
NM_001195223	ALG1L	putative glycosyltran	-	125648117	125655887
NM_002607	PDGFA	platelet-derived grow	-	536896	559481
NM_033023	PDGFA	platelet-derived grow	-	536896	559481
NM_001035247	UFD1L	ubiquitin fusion degr	-	19437463	19466738
NM_001166242	C22orf39	UPF0545 protein C2	-	19428409	19435755
NM_005659	UFD1L	ubiquitin fusion degr	-	19437463	19466738
NM_173793	C22orf39	UPF0545 protein C2	-	19430672	19435755
NM_001008895	CUL4A	cullin-4A isoform 1	+	113863930	113919392
NM_003589	CUL4A	cullin-4A isoform 2	+	113863085	113919392

92.052497	16.179842	5.937141	4.238138	43.07636	39.61487
88.7968	18.514298	5.828093	4.481472	63.019043	18.740238
20272.5755	4333.402903	13.566453	10.808547	21746.863	23993.293
71.423709	12.953819	5.652237	3.961561	73.159996	26.882257
79.150215	5.698624	5.794701	2.566659	109.114685	50.699493
381.061872	55.539379	8.183943	6.088715	358.8113	408.39392
629.482038	189.41634	8.74226	6.580518	682.2636	757.8183
37.526759	10.035966	4.640247	3.573667	31.359049	49.687874
851.0604	230.629612	9.272061	8.056528	936.81305	902.5186
851.0604	230.629612	9.272061	8.056528	936.81305	902.5186
851.0604	230.629612	9.272061	8.056528	936.81305	902.5186
78.516204	19.992049	5.75598	4.315289	65.97149	16.099607
28.094792	8.402815	4.472924	3.155756	38.6706	23.453745
44.245145	9.573393	5.045115	3.415655	35.165302	17.85141
117.483724	34.829854	6.494049	5.337285	151.73264	152.0191
730.221017	222.749642	9.049092	7.927711	823.33954	944.7433
43.281816	15.676834	5.065365	3.87023	53.955082	38.68104
43.281816	15.676834	5.065365	3.87023	53.955082	38.68104
43.281816	15.676834	5.065365	3.87023	53.955082	38.68104
391.253815	107.52869	7.998314	6.968654	323.8665	640.8166
581.362942	168.53195	8.73475	7.606187	585.88794	576.1907
581.362942	168.53195	8.73475	7.606187	585.88794	576.1907
57.142486	9.386562	5.495375	3.465735	63.995094	60.12333
199.565593	40.718721	7.269684	5.549331	203.73795	258.30997
324.957845	90.766199	7.85143	6.802485	392.51212	544.60254
1452.62605	226.92445	10.06653	8.146283	1639.101	2070.9814
34.354617	10.941639	4.73669	3.509458	24.151676	43.88023
54.508398	16.457833	5.376218	4.203932	45.96606	25.795753
1032.106777	168.222714	9.201204	7.178768	1229.6658	2406.4534
35.522207	8.868293	4.751049	3.361095	31.792948	20.17467
205.241427	42.713142	7.262727	5.685363	230.62083	261.35626
36.063745	14.530002	4.802902	3.76864	31.554264	30.917192
79.695651	19.388436	5.927877	4.29562	69.39966	48.691658
211.710322	54.320071	7.129558	6.070974	332.72296	435.64285
211.710322	54.320071	7.129558	6.070974	332.72296	435.64285
1097.109872	276.221219	9.591495	7.527817	1164.9069	1228.8011
405.508108	47.816389	7.478057	5.880039	699.72144	81.78328
33.522611	8.491917	4.703125	3.331677	39.370308	36.06559
3300.916433	442.749393	11.258552	9.112706	3710.3435	3756.004
47.498355	10.217536	5.082805	3.542437	41.017826	34.143314
213.040258	20.219782	7.348211	4.576142	202.66965	138.64699
61.795269	11.687447	4.836374	3.348307	28.780241	10.808602
639.429413	149.070853	8.929453	7.495569	679.4323	577.71204
639.429413	149.070853	8.929453	7.495569	679.4323	577.71204
521.633262	101.915206	8.434606	6.931218	710.08014	1078.02
147.542285	33.871544	6.729295	5.361261	93.37459	74.8396
6291.67625	1737.46165	12.090801	11.062284	6593.3857	3724.111
278.162303	49.528168	7.696144	5.677596	300.5157	402.17194
37.766059	6.692404	4.704015	2.95772	32.733116	9.8561125
1786.078183	467.66869	10.358265	9.212899	1712.5779	1152.4476
13.682226	5	3.457091	2.363479	13.708798	15.768819
551.13411	171.550489	8.701115	7.645255	514.744	609.8299
551.13411	171.550489	8.701115	7.645255	514.744	609.8299
54.682733	13.404414	5.264583	4.014605	35.527145	32.059284
54.682733	13.404414	5.264583	4.014605	35.527145	32.059284
430.960457	148.904062	8.312563	7.264362	349.16574	294.6756
430.960457	148.904062	8.312563	7.264362	349.16574	294.6756
430.960457	148.904062	8.312563	7.264362	349.16574	294.6756
430.960457	148.904062	8.312563	7.264362	349.16574	294.6756
79.787322	15.742435	5.58562	3.762702	131.18266	71.38567
796.493175	216.516387	9.212718	8.072432	749.3657	552.5786
796.493175	216.516387	9.212718	8.072432	749.3657	552.5786

45.352417	106.784386	151.88025	165.6067	13.730569	8.614886
74.1839	56.544968	164.49005	155.8026	20.612534	11.59505
13938.793	2772.564	31346.59	27837.35	4372.5444	388.9493
104.38361	114.8205	30.667887	78.628006	14.046481	8.582743
163.13722	47.349182	46.893322	57.70739	4.9999995	4.9999995
290.92944	407.01703	348.7745	472.44504	56.960434	72.91756
399.0864	199.51213	983.1567	755.0551	228.23486	30.191608
4.9999995	50.731712	35.306522	53.075397	9.575189	7.413819
732.9071	1123.5482	394.90045	1015.675	252.13142	122.8264
732.9071	1123.5482	394.90045	1015.675	252.13142	122.8264
732.9071	1123.5482	394.90045	1015.675	252.13142	122.8264
99.3611	116.9211	67.309204	105.43472	13.170048	4.9999995
22.545568	26.898912	31.669016	25.33091	12.66467	4.9999995
45.406982	61.559624	40.08747	65.40008	11.690788	11.055631
114.53994	98.793274	84.66547	103.15192	36.60338	16.804369
528.8762	358.6671	869.7223	855.97766	238.47955	83.01592
64.28638	38.627003	27.786043	36.35535	19.457542	4.9999995
64.28638	38.627003	27.786043	36.35535	19.457542	4.9999995
64.28638	38.627003	27.786043	36.35535	19.457542	4.9999995
178.51988	129.86525	628.5099	445.94476	125.176186	113.60877
618.2065	810.6344	282.98425	614.27386	189.53969	71.9997
618.2065	810.6344	282.98425	614.27386	189.53969	71.9997
47.653793	64.78518	48.395622	57.9019	10.40215	10.59874
176.35025	205.1127	150.3425	203.54019	56.712315	70.87671
217.94803	363.18274	139.19698	292.30466	80.508385	107.94887
1165.8013	1166.7965	1223.32	1449.7561	170.35323	198.94583
23.70291	35.10646	42.80999	36.476437	13.758271	4.9999995
68.66804	65.76906	48.21449	72.636986	19.439428	8.372651
415.68408	190.87668	1069.8921	880.0686	225.10512	109.91728
26.057188	32.489662	52.662506	49.95627	6.0459085	6.1833644
167.03952	111.36609	217.91887	243.14699	39.9427	23.80883
33.76649	62.84619	22.787653	34.51068	6.1365733	11.01486
98.54444	115.70632	58.180508	87.65132	19.821888	4.9999995
127.075195	163.96927	113.6895	97.162155	54.26417	43.054455
127.075195	163.96927	113.6895	97.162155	54.26417	43.054455
831.16705	430.94528	1625.122	1301.7169	434.47842	74.85501
1277.6271	140.44678	100.20237	133.26768	49.901993	32.794106
39.49548	33.71654	16.658487	35.82926	9.927426	5.2551546
3764.3606	2778.7786	2533.0195	3262.9924	325.47934	538.25055
31.309504	104.95078	22.326021	51.242683	9.570139	11.46614
260.41827	257.92725	172.18378	246.39561	15.586133	10.400391
50.47174	4.9999995	148.37192	127.33911	5.099242	4.9999995
722.18634	548.6205	592.03375	716.59155	167.59927	123.44571
722.18634	548.6205	592.03375	716.59155	167.59927	123.44571
292.8429	216.09512	430.73224	402.02917	115.64939	67.28563
106.43167	230.07533	140.57784	239.95468	29.216375	21.629171
9087.656	8429.861	2888.1228	7026.921	2047.1567	1052.1721
160.02559	280.10904	219.22122	306.93033	31.867308	22.641998
37.955746	75.58407	21.713621	48.75369	8.087764	10.48197
2200.5918	2441.4797	1329.0746	1880.2975	393.16833	351.15033
12.880248	15.375411	10.058711	14.301369	4.9999995	4.9999995
379.9342	656.0796	520.62726	625.5897	111.005585	166.8266
379.9342	656.0796	520.62726	625.5897	111.005585	166.8266
61.34824	108.64671	21.55265	68.96237	11.26223	10.425401
61.34824	108.64671	21.55265	68.96237	11.26223	10.425401
327.63043	654.956	362.65137	596.6836	118.017296	26.210264
327.63043	654.956	362.65137	596.6836	118.017296	26.210264
327.63043	654.956	362.65137	596.6836	118.017296	26.210264
327.63043	654.956	362.65137	596.6836	118.017296	26.210264
182.519	25.513378	30.578533	37.54469	22.742876	4.9999995
848.87274	1035.0062	572.22797	1020.90784	227.86432	135.42017
848.87274	1035.0062	572.22797	1020.90784	227.86432	135.42017

23.463793	13.6361	17.952156	19.68155	4.969976	5.2045507
17.543983	25.840134	16.39909	19.094995	5.521252	4.2159348
19401.277	441.73282	907.4168	488.4971	13.950177	14.133187
16.74106	13.033379	12.03458	13.284672	5.7370043	4.696725
7.004584	7.1871624	4.9999995	4.9999995	6.321594	5.5317774
33.17481	77.06034	47.20024	45.92289	8.027199	8.368383
773.9482	30.85896	45.705097	27.559317	8.9428835	9.235538
8.215114	16.151321	10.92313	7.9372244	4.5137343	5.500935
399.17004	118.31619	322.43442	168.8992	9.403176	9.484208
399.17004	118.31619	322.43442	168.8992	9.403176	9.484208
399.17004	118.31619	322.43442	168.8992	9.403176	9.484208
20.190453	27.750929	24.970903	28.869959	5.583965	4.0259857
11.850092	5.383131	9.357645	6.1613526	4.8081784	4.51008
4.9999995	17.190624	5.455216	7.0481005	4.6758213	4.155762
61.999905	33.406467	28.992088	31.172915	6.7892675	7.0110326
516.32666	173.68382	184.82138	140.17052	9.223883	9.545251
33.280888	14.48613	14.978561	6.857881	5.2867675	5.1746325
33.280888	14.48613	14.978561	6.857881	5.2867675	5.1746325
33.280888	14.48613	14.978561	6.857881	5.2867675	5.1746325
131.94185	51.244358	159.82129	63.379684	7.8810744	9.004382
318.15717	126.512215	177.35852	127.624405	8.718557	8.859375
318.15717	126.512215	177.35852	127.624405	8.718557	8.859375
4.9999995	9.376392	8.47743	12.464661	5.5419273	5.7483354
24.726595	37.93934	30.654945	23.402422	7.2172213	7.748699
57.457577	139.80638	75.66895	83.20703	8.159349	8.778149
148.66724	271.02176	341.94504	230.6136	10.208026	10.600171
12.08071	16.98533	11.594051	6.2314734	4.136646	5.339797
30.12361	16.268332	13.808148	10.73483	5.0625634	4.640668
469.7864	30.76308	116.357475	57.40693	9.7872715	10.805613
11.88777	7.0873256	13.232698	8.772693	4.5321236	4.3089967
44.984703	41.127647	66.14614	40.26883	7.3976846	7.765858
7.2287655	36.638226	4.9999995	21.16159	4.5203495	4.8843236
22.169947	21.425253	28.908573	19.004957	5.6594	5.477187
76.77857	47.792187	56.384434	47.64661	7.9216166	8.45911
76.77857	47.792187	56.384434	47.64661	7.9216166	8.45911
940.6367	55.88614	85.33142	66.139626	9.709318	9.902028
67.55164	51.762188	42.39695	42.49146	8.979126	6.159248
5.92984	12.389869	8.9255085	8.523706	4.836656	5.082327
240.30437	589.5355	508.06143	454.86517	11.377388	11.461204
13.82932	11.31792	4.9999995	10.1217	4.895299	5.0142083
22.442753	28.296125	19.20107	25.39222	7.209155	6.8859763
4.9999995	9.422351	31.058594	14.544499	4.3946624	3.5007918
226.21628	93.132545	179.13931	104.892006	8.938146	8.863318
226.21628	93.132545	179.13931	104.892006	8.938146	8.863318
174.75006	73.102715	106.58079	74.12265	9.002161	9.727964
39.86905	50.248035	33.21302	29.053616	6.099095	6.0394397
3270.5225	1277.7454	1668.0779	1109.0953	12.21414	11.443338
26.285696	30.595356	121.3229	64.45575	7.777924	8.348778
4.9999995	4.9999995	5.429803	6.1548905	4.571232	3.3823872
413.76532	728.31274	389.84485	529.77057	10.269868	9.817555
4.9999995	4.9999995	4.9999995	4.9999995	3.2980678	3.9986062
59.10558	229.88477	247.52383	214.95657	8.524447	8.937201
59.10558	229.88477	247.52383	214.95657	8.524447	8.937201
18.240105	13.949211	14.472899	12.07664	4.6896424	4.929501
18.240105	13.949211	14.472899	12.07664	4.6896424	4.929501
211.8212	110.2518	272.51373	154.61008	7.9952574	7.9328475
211.8212	110.2518	272.51373	154.61008	7.9952574	7.9328475
211.8212	110.2518	272.51373	154.61008	7.9952574	7.9328475
211.8212	110.2518	272.51373	154.61008	7.9952574	7.9328475
33.586597	5.856607	20.38763	6.8809013	6.588511	5.9786
310.49396	183.68738	262.55298	179.07951	9.082062	8.801169
310.49396	183.68738	262.55298	179.07951	9.082062	8.801169

5.1825056	6.3904905	7.027473	6.84785	4.006775	3.8792942
5.8971314	5.4382467	7.1350074	6.7609873	4.603236	4.3248324
13.480722	11.104032	14.563588	14.167015	12.407313	9.472637
6.388678	6.509368	4.7978883	5.783756	4.0395145	3.8736084
7.0156975	5.165352	5.3833957	5.350389	2.3467326	2.4279919
7.8522677	8.356371	8.188391	8.311046	6.0704503	7.031358
8.291078	7.3206496	9.684285	8.979126	8.086018	5.7551394
2.3258038	5.272189	4.9883494	5.2404714	3.4782882	3.6510592
9.165597	9.807618	8.371483	9.400282	8.235976	7.7894106
9.165597	9.807618	8.371483	9.400282	8.235976	7.7894106
9.165597	9.807618	8.371483	9.400282	8.235976	7.7894106
6.315116	6.5367284	5.8743763	6.199711	3.9453838	2.4279919
4.182075	4.286735	4.842116	4.2083573	3.8899095	2.4279919
5.1851177	5.567591	5.1657352	5.520661	3.7762213	4.257183
6.5207367	6.2760463	6.1985574	6.168655	5.429368	4.886526
8.692288	8.163056	9.509842	9.160231	8.150287	7.228176
5.701051	4.845128	4.665508	4.719104	4.519997	2.4279919
5.701051	4.845128	4.665508	4.719104	4.519997	2.4279919
5.701051	4.845128	4.665508	4.719104	4.519997	2.4279919
7.151937	6.6922803	9.028956	8.231252	7.1994696	7.679269
8.8983135	9.347438	7.9002376	8.684581	7.811996	7.0142674
8.8983135	9.347438	7.9002376	8.684581	7.811996	7.0142674
5.2572536	5.6450987	5.4249654	5.3546724	3.6071098	4.192396
7.1332765	7.365166	7.0142674	7.1394725	6.0631127	6.994612
7.447058	8.1801195	6.899891	7.6440134	6.55507	7.600642
9.817851	9.871557	9.990443	9.91113	7.657093	8.495431
4.258119	4.6986756	5.2630916	4.723813	4.008932	2.4279919
5.7919316	5.668151	5.420184	5.6738076	4.518054	3.8351514
8.3553	7.255569	9.808744	9.194729	8.065393	7.626748
4.3946624	4.579701	5.536999	5.1538105	2.7983694	3.379466
7.0526505	6.456041	7.5244865	7.3796406	5.555566	5.409044
4.7632113	5.5982914	4.4015985	4.6496377	2.8203588	4.251554
6.3017235	6.520857	5.6742473	5.933847	4.5441346	2.4279919
6.665085	7.034821	6.6141176	6.082597	5.9977736	6.274859
6.665085	7.034821	6.6141176	6.082597	5.9977736	6.274859
9.347438	8.443109	10.380815	9.766264	9.028195	7.070514
9.95616	6.808729	6.4335265	6.5315537	5.876034	5.8732653
4.9886355	4.633879	3.978674	4.698577	3.533727	3.123984
11.519689	11.107282	11.027109	11.058637	8.614396	9.935325
4.661604	6.362386	4.3753033	5.1880274	3.4772117	4.310066
7.693179	7.701006	7.198965	7.4009843	4.1928	4.164661
5.342207	2.3221264	6.992454	6.466003	2.5704718	2.4279919
9.141812	8.778149	8.949514	8.905782	7.633998	7.7978673
9.141812	8.778149	8.949514	8.905782	7.633998	7.7978673
7.8606377	7.446378	8.492233	8.078265	7.0913277	6.926316
6.4173727	7.542966	6.91467	7.3622255	5.098074	5.265789
12.823107	12.690246	11.211527	12.162447	11.3147135	10.919481
6.988374	7.812336	7.5324755	7.716975	5.2259626	5.328396
4.9331083	5.880164	4.33707	5.120128	3.2178926	4.176596
10.754457	10.91888	10.101313	10.287517	8.877764	9.325216
3.3580575	3.3810966	3.2827628	3.423956	2.3467326	2.4279919
8.219886	9.044526	8.767781	8.712848	7.0300875	8.229831
8.219886	9.044526	8.767781	8.712848	7.0300875	8.229831
5.630742	6.416201	4.326337	5.5950766	3.7238145	4.169031
5.630742	6.416201	4.326337	5.5950766	3.7238145	4.169031
8.021657	9.041888	8.247615	8.636113	7.119849	5.5478826
8.021657	9.041888	8.247615	8.636113	7.119849	5.5478826
8.021657	9.041888	8.247615	8.636113	7.119849	5.5478826
8.021657	9.041888	8.247615	8.636113	7.119849	5.5478826
7.188959	4.2008896	4.79337	4.763393	4.748013	2.4279919
9.379216	9.700932	8.902398	9.410531	8.083208	7.9316487
9.379216	9.700932	8.902398	9.410531	8.083208	7.9316487

4.581539	3.8931668	4.437399	4.6306543
4.1842256	4.8829436	4.3089967	4.5845947
14.1761265	9.235369	10.0708685	9.488966
4.123904	3.8246913	3.8661284	4.0415215
2.9718444	2.938816	2.3618152	2.352752
5.06311	6.590323	5.8348327	5.942216
9.544808	5.1570044	5.7830095	5.1571293
3.1701298	4.1560416	3.7241259	3.2623558
8.596906	7.242311	8.573426	7.9011364
8.596906	7.242311	8.573426	7.9011364
8.596906	7.242311	8.573426	7.9011364
4.3766584	4.9970264	4.91364	5.231032
3.6561031	2.5656483	3.5120616	2.8828216
2.350709	4.2456536	2.7844732	3.0796893
5.9393587	5.277883	5.136894	5.3536797
8.965703	7.8187933	7.780123	7.623181
5.0689945	3.991607	4.1775355	3.0352545
5.0689945	3.991607	4.1775355	3.0352545
5.0689945	3.991607	4.1775355	3.0352545
7.000282	5.9525433	7.550085	6.430274
8.264001	7.3515215	7.7126794	7.4826546
8.264001	7.3515215	7.7126794	7.4826546
2.350709	3.3274324	3.3754	3.9413645
4.6513805	5.4783583	5.213279	4.8952456
5.831658	7.496756	6.503477	6.8273077
7.1691866	8.520073	8.6537485	8.382165
3.6818385	4.227992	3.8092785	2.9007175
4.929294	4.1658506	4.0589104	3.7163334
8.829414	5.152663	7.1148143	6.2835765
3.6602206	2.9191723	4.0002627	3.4090776
5.489077	5.6008096	6.316082	5.7416005
3.007496	5.4263725	2.3618152	4.7442455
4.503815	4.588143	5.1318736	4.5777607
6.2309866	5.840043	6.0837755	5.998408
6.2309866	5.840043	6.0837755	5.998408
9.819699	6.0880957	6.6686206	6.4917765
6.05861	5.9685664	5.6786127	5.825148
2.7745209	3.747278	3.4444818	3.3660731
7.853992	9.66805	9.223558	9.380915
3.8631744	3.6108353	2.3618152	3.631518
4.519867	5.0259094	4.5289574	5.024656
2.350709	3.33342	5.2313824	4.175867
7.7635474	6.871183	7.7307467	7.176069
7.7635474	6.871183	7.7307467	7.176069
7.403175	6.5131993	6.991296	6.6619935
5.314548	5.9222245	5.325081	5.241852
11.64623	10.82421	10.955095	10.713975
4.739735	5.145493	7.1701584	6.455829
2.350709	2.3408759	2.7789788	2.8812673
8.648947	9.978375	8.846115	9.600977
2.350709	2.3408759	2.3618152	2.352752
5.8718977	8.254787	8.208026	8.276898
5.8718977	8.254787	8.208026	8.276898
4.243815	3.9282544	4.1269555	3.8957617
4.243815	3.9282544	4.1269555	3.8957617
7.674352	7.1293497	8.341963	7.7727757
7.674352	7.1293497	8.341963	7.7727757
7.674352	7.1293497	8.341963	7.7727757
7.674352	7.1293497	8.341963	7.7727757
5.082327	2.660783	4.616348	3.0407467
8.2238035	7.9109406	8.2918215	7.993169
8.2238035	7.9109406	8.2918215	7.993169

ASHGA5P041074	0.000450046	0.014980461	2.1677044	up	noncoding
ASHGA5P042199	0.009947312	0.061915152	2.0652369	up	noncoding
ASHGA5P027391	0.011298323	0.065455073	2.0822545	up	noncoding
ASHGA5P052531	0.001656978	0.027211232	2.0575955	up	noncoding
ASHGA5P057641	0.016384255	0.07814689	2.4070855	up	noncoding
ASHGA5P017601	0.038401725	0.124684947	4.0701541	up	noncoding
ASHGA5P029203	0.00028306	0.012492602	4.7146348	up	noncoding
ASHGA5P029203	0.00028306	0.012492602	4.7146348	up	noncoding
ASHGA5P029203	0.00028306	0.012492602	4.7146348	up	noncoding
ASHGA5P029203	0.00028306	0.012492602	4.7146348	up	noncoding
ASHGA5P029203	0.00028306	0.012492602	4.7146348	up	noncoding
ASHGA5P036775	0.000236404	0.011933736	3.0242906	up	noncoding
ASHGA5P020919	0.001652917	0.027211232	2.6006781	up	noncoding
ASHGA5P020919	0.001652917	0.027211232	2.6006781	up	noncoding
ASHGA5P020919	0.001652917	0.027211232	2.6006781	up	noncoding
ASHGA5P014668	0.000812644	0.019071379	4.6661324	up	noncoding
ASHGA5P026756	0.00055781	0.016097331	2.0335124	up	noncoding
ASHGA5P016643	0.000543107	0.016009043	6.9760798	up	noncoding
ASHGA5P021756	0.038123836	0.124178673	2.4211824	up	noncoding
ASHGA5P046457	0.022687612	0.093189341	2.5241125	up	noncoding
ASHGA5P023551	0.016778807	0.078957015	2.4459357	up	noncoding
ASHGA5P019332	0.001823655	0.028643965	2.7157544	up	noncoding
ASHGA5P019332	0.001823655	0.028643965	2.7157544	up	noncoding
ASHGA5P051749	0.003356491	0.037423637	2.3339914	up	noncoding
ASHGA5P025918	0.003185604	0.036540506	3.1892772	up	noncoding
ASHGA5P025918	0.003185604	0.036540506	3.1892772	up	noncoding
ASHGA5P025918	0.003185604	0.036540506	3.1892772	up	noncoding
ASHGA5P025918	0.003185604	0.036540506	3.1892772	up	noncoding
ASHGA5P022979	0.011289904	0.065455073	2.2176838	up	noncoding
ASHGA5P022979	0.011289904	0.065455073	2.2176838	up	noncoding
ASHGA5P022979	0.011289904	0.065455073	2.2176838	up	noncoding
ASHGA5P027541	0.007028974	0.052947146	2.1461837	up	noncoding
ASHGA5P056316	0.000870793	0.019883224	3.5098283	up	noncoding
ASHGA5P036779	0.002417958	0.031997277	4.245446	up	noncoding
ASHGA5P051170	0.000112088	0.009604611	3.3498184	up	noncoding
ASHGA5P028818	0.010354642	0.062843093	2.3710475	up	noncoding
ASHGA5P028818	0.010354642	0.062843093	2.3710475	up	noncoding
ASHGA5P020639	0.000546723	0.01603938	3.5793683	up	noncoding
ASHGA5P053882	0.016128457	0.077717436	2.042821	up	noncoding
ASHGA5P016660	1.93261E-05	0.004838692	2.2632897	up	noncoding
ASHGA5P029596	0.039052982	0.125985736	2.2724824	up	noncoding
ASHGA5P030691	0.000129936	0.010460795	4.6171897	up	noncoding
ASHGA5P030532	0.040997537	0.129441514	12.2338209	up	noncoding
ASHGA5P030532	0.040997537	0.129441514	12.2338209	up	noncoding
ASHGA5P030532	0.040997537	0.129441514	12.2338209	up	noncoding
ASHGA5P030532	0.040997537	0.129441514	12.2338209	up	noncoding
ASHGA5P030532	0.040997537	0.129441514	12.2338209	up	noncoding
ASHGA5P039165	0.019271613	0.084598619	2.029227	up	noncoding
ASHGA5P049514	0.007307525	0.053778037	2.3085138	up	noncoding
ASHGA5P049514	0.007307525	0.053778037	2.3085138	up	noncoding
ASHGA5P038533	0.031394587	0.111265481	2.366561	up	noncoding
ASHGA5P026504	0.002104181	0.03028922	2.196086	up	noncoding
ASHGA5P032551	0.012945484	0.069717918	2.6249989	up	noncoding
ASHGA5P039311	0.030539932	0.109648554	3.8394862	up	noncoding
ASHGA5P028120	0.019099728	0.084217792	2.348014	up	noncoding
ASHGA5P030911	8.33913E-05	0.008738697	6.4559835	up	noncoding
ASHGA5P014184	1.40415E-05	0.004434727	4.1280499	up	noncoding
ASHGA5P014184	1.40415E-05	0.004434727	4.1280499	up	noncoding
ASHGA5P048363	0.01338832	0.070917754	2.0529517	up	noncoding
ASHGA5P028899	0.000209711	0.01146874	7.6262526	up	noncoding
ASHGA5P028519	0.02476597	0.09773105	2.1818576	up	noncoding
ASHGA5P043242	0.020856138	0.088872205	2.2752255	up	noncoding

TCONS_00021656	XLOC_010622	LincRNAs identified	6166	chr13	-
uc003qxf.3	AK023627	UCSC_knowngene	2072	chr6	-
ENST00000546214	RP11-415I12.5	GENCODE	563	chr12	-
ENST00000515525	RP11-423H2.1	GENCODE	573	chr5	+
TCONS_00020287	XLOC_009632	LincRNAs identified	882	chr12	+
ENST00000443621	LINC00393	GENCODE	282	chr13	-
ENST00000557409	RP11-363J20.1	GENCODE	538	chr14	-
ENST00000557409	RP11-363J20.1	GENCODE	538	chr14	-
ENST00000557409	RP11-363J20.1	GENCODE	538	chr14	-
ENST00000557409	RP11-363J20.1	GENCODE	538	chr14	-
ENST00000557409	RP11-363J20.1	GENCODE	538	chr14	-
uc002xid.1	AX746690	UCSC_knowngene	2002	chr20	-
NR_027427	TATDN1	RefSeq	1158	chr8	-
NR_027427	TATDN1	RefSeq	1158	chr8	-
NR_027427	TATDN1	RefSeq	1158	chr8	-
ENST00000412301	AC129778.2	GENCODE	476	chr9	-
TCONS_00012453	XLOC_005238	LincRNAs identified	956	chr6	+
ENST00000432481	AC009410.1	GENCODE	676	chr2	-
ENST00000540093	RP11-885B4.1	GENCODE	220	chr12	-
ENST00000433228	RP1-60N8.1	GENCODE	1554	chrX	+
ENST00000583854	CTD-2532D12.4	GENCODE	1140	chr17	+
NR_045147	ITGB3BP	RefSeq	1069	chr1	-
NR_045147	ITGB3BP	RefSeq	1069	chr1	-
uc001eso.1	AB007962	UCSC_knowngene	5053	chr1	+
NR_046318	ENC1	RefSeq	3705	chr5	-
NR_046318	ENC1	RefSeq	3705	chr5	-
NR_046318	ENC1	RefSeq	3705	chr5	-
NR_046318	ENC1	RefSeq	3705	chr5	-
ENST00000567047	RP11-626G11.5	GENCODE	380	chr16	-
ENST00000567047	RP11-626G11.5	GENCODE	380	chr16	-
ENST00000567047	RP11-626G11.5	GENCODE	380	chr16	-
ENST00000549807	RP11-818F20.5	GENCODE	701	chr12	-
TCONS_00002364	XLOC_000808	LincRNAs identified	322	chr1	-
ENST00000439912	SNORA71A	GENCODE	138	chr20	-
ENST00000447037	AP001469.9	GENCODE	686	chr21	-
NR_046511	MED4-AS1	RefSeq	544	chr13	+
NR_046511	MED4-AS1	RefSeq	544	chr13	+
ENST00000517838	PVT1	GENCODE	518	chr8	+
uc004cin.3	SNHG7	UCSC_knowngene	743	chr9	-
ENST00000432673	RP11-390P2.4	GENCODE	917	chr6	+
ENST00000419578	RP1-13P20.6	GENCODE	1997	chr1	-
ENST00000561560	RP11-358L4.1	GENCODE	529	chr15	+
NR_024490	GABPB1-AS1	RefSeq	4139	chr15	+
NR_024490	GABPB1-AS1	RefSeq	4139	chr15	+
NR_024490	GABPB1-AS1	RefSeq	4139	chr15	+
NR_024490	GABPB1-AS1	RefSeq	4139	chr15	+
NR_024490	GABPB1-AS1	RefSeq	4139	chr15	+
ENST00000569932	RP11-255C15.3	GENCODE	1566	chr3	+
NR_045027	C17orf76-AS1	RefSeq	1158	chr17	+
NR_045027	C17orf76-AS1	RefSeq	1158	chr17	+
ENST00000566372	RP11-16N11.2	GENCODE	1715	chr3	-
ENST00000527789	AP003068.12	GENCODE	175	chr11	+
TCONS_00029045	XLOC_013960	LincRNAs identified	461	chr21	+
ENST00000511928	AC141928.1	GENCODE	4525	chr4	-
ENST00000546170	GS1-410F4.4	GENCODE	444	chr12	+
ENST00000576762	AJ003147.8	GENCODE	454	chr16	-
NR_033255	DTYMK	RefSeq	1136	chr2	-
NR_033255	DTYMK	RefSeq	1136	chr2	-
uc009yvh.3	AK311356	UCSC_knowngene	853	chr11	-
ENST00000581816	MIR17HG	GENCODE	2032	chr13	+
ENST00000413246	RP11-187A9.3	GENCODE	363	chr13	-
uc010lhv.1	AK091729	UCSC_knowngene	1986	chr7	-

53263721	53274883		CACACTTGTATATGCTCCACACTGA1	intergenic
170149607	170151679		GTGTTCCCTGTGTCTTTCTGAATCTC	exon sense-overlappi
64202624	64215936		TTCCAGTTCTTGATAAAGATAAAGG	natural antisense
177303255	177309506	ENST00000502601,	AATCTCCTGGAACAATGACAATAAT	intergenic
6387276	6391856		TTTTTCCTGATTTGCTCTGAGAAGA(	intergenic
73987609	74120978		GACTTTTTTGGTCTACTGTGTCTGCC	intergenic
69657594	69658809		CTATGCCATCTTTTATAGCGATAAC(	intronic antisense
69657594	69658809		CTATGCCATCTTTTATAGCGATAAC(	intronic antisense
69657594	69658809		CTATGCCATCTTTTATAGCGATAAC(	intronic antisense
69657594	69658809		CTATGCCATCTTTTATAGCGATAAC(	natural antisense
69657594	69658809		CTATGCCATCTTTTATAGCGATAAC(	natural antisense
37034659	37045444		AAGGCTCAACTGGGTATGATTGTCA	intergenic
125500734	125551329		GATTTAATGGTT(83940	exon sense-overlappi
125500734	125551329		GATTTAATGGTT(83940	exon sense-overlappi
125500734	125551329		GATTTAATGGTT(83940	natural antisense
42853719	42857852	ENST00000432011	TGGATGGTGGCTTCCAATTGTGGAC	intergenic
30259313	30271912		GCTTCTCTAGGTGAAGGTTTAAATT/	intergenic
229347976	229476111		CCCTGGAGCCTTCATATAGAACTAC'	intergenic
2851791	2881074		GTACATACAGAGTGTTCTCACCCAA	intergenic
17546557	17605281		GGACAATGCTGATTTAGTAAGTTGG	intron sense-overlapp
71733992	71752696		GATTATAAAGCTTCTCCCTAATGGG(	intergenic
63906440	63988944		GCTCTCCTAGCA1 23421	exon sense-overlappi
63906440	63988944		GCTCTCCTAGCA1 23421	exon sense-overlappi
149627308	149651107	ENST00000325963,	GCACCCCTTGAAGGTTTTGTAATGA	intergenic
73923230	73937249		AGTCGCCACTGC1 8507	exon sense-overlappi
73923230	73937249		AGTCGCCACTGC1 8507	exon sense-overlappi
73923230	73937249		AGTCGCCACTGC1 8507	exon sense-overlappi
73923230	73937249		AGTCGCCACTGC1 8507	exon sense-overlappi
19074183	19079013		CACAGCAGACGAAAAAGCAAAGAG(	natural antisense
19074183	19079013		CACAGCAGACGAAAAAGCAAAGAG(	natural antisense
19074183	19079013		CACAGCAGACGAAAAAGCAAAGAG(	natural antisense
104656091	104674500		ACTGCACATGTAGAACGGTGTTCCT(	intronic antisense
43823627	43824390		TTAAATCCTACACGAGAGTCGTATT'	intergenic
37055948	37056086		AAGAGGTTGTCC(26777	intergenic
47671462	47672748		CATAGTAAGATGTGGTATTTGTGGA	exon sense-overlappi
48651272	48654129		AGGATTGTTCAT(100873965	natural antisense
48651272	48654129		AGGATTGTTCAT(100873965	natural antisense
128903115	129008896		ATTTGGAGAGAA' 5820	intronic antisense
139619045	139620988	NR_003672	AGGATCACGCAG(84973	natural antisense
139013684	139018425		AGCGCGACAGGGTTGTTATGAGGCT	natural antisense
152902515	152921686		GTAAGGCAGCTAATATTTCTGTCAC	intergenic
79705699	79706228		AGTGGCAGGTCTGATTTGGTTATAG	intergenic
50646370	50650503		ATCTGGGTTCCG(100129387	natural antisense
50646370	50650503		ATCTGGGTTCCG(100129387	natural antisense
50646370	50650503		ATCTGGGTTCCG(100129387	natural antisense
50646370	50650503		ATCTGGGTTCCG(100129387	natural antisense
50646370	50650503		ATCTGGGTTCCG(100129387	natural antisense
179058109	179059675		CTTGTATACCGTGTAAATATTTTATA	intergenic
16342300	16345340	ENST00000472293,	GGGCGCTGCGGC(125144	natural antisense
16342300	16345340	ENST00000472293,	GGGCGCTGCGGC(125144	natural antisense
160471215	160472930		CTGAAGATTTGGTGGAAATATGCTT	bidirectional
64884628	64884930		CGAGCTCCAGCTCCGCGGCGTCACT	natural antisense
44776023	44777849		TTCAACGTCTCTTGGTTGAAACCTT(	intergenic
3760474	3765117		GAGACTGATGCTACATTTCTCTTTA1	intergenic
68323042	68363797		GGGTGAAGTTTTAAGGGTGCTTCAC	intergenic
3231232	3234018		GTATCCGACTTATAATACAACTGTC(	intergenic
242615156	242626383		TTTTTCTGCAAAT 1841	exon sense-overlappi
242615156	242626383		TTTTTCTGCAAAT 1841	exon sense-overlappi
82903178	82904681		GAGTTTGACTCAATTCTTATTATAA(	bidirectional
92000195	92004350	NR_027350	CCCCATTAGGGA' 407975	intergenic
42617862	42618383		CCCATAAGGGATAATGCAACAGGAT	intronic antisense
100893615	100895601		TCCACAGGCATAGAATCCATGTGAC	exon sense-overlappi

NM_174910	TCTE3	tctex1 domain-contai	-	170140214	170151638
NM_014254	TMEM5	transmembrane prote	+	64173636	64202887
NM_001193360	EXD2	exonuclease 3'-5' dor	+	69658193	69710737
NM_001193361	EXD2	exonuclease 3'-5' dor	+	69658193	69710737
NM_018199	EXD2	exonuclease 3'-5' dor	+	69658193	69710737
NM_001193362	EXD2	exonuclease 3'-5' dor	+	69658482	69710737
NM_001193363	EXD2	exonuclease 3'-5' dor	+	69658482	69710737
NM_001146160	TATDN1	putative deoxyribonu	-	125500734	125551329
NM_032026	TATDN1	putative deoxyribonu	-	125500734	125551329
NM_007218	RNF139	E3 ubiquitin-protein	+	125487007	125500859
NM_198270	NHS	Nance-Horan syndro	+	17393542	17754113
NM_001206739	ITGB3BP	centromere protein R	-	63906440	63988944
NM_014288	ITGB3BP	centromere protein R	-	63906440	63988944
NM_001256574	ENC1	ectoderm-neural cort	-	73923230	73937249
NM_001256575	ENC1	ectoderm-neural cort	-	73923230	73937249
NM_001256576	ENC1	ectoderm-neural cort	-	73923230	73937249
NM_003633	ENC1	ectoderm-neural cort	-	73923230	73937249
NM_001160364	TMC7	transmembrane chan	+	18995608	19075262
NM_016138	COQ7	ubiquinone biosynthe	+	19078916	19091417
NM_024847	TMC7	transmembrane chan	+	18995255	19075262
NM_001093771	TXNRD1	thioredoxin reductase	+	104609556	104744085
NM_003906	MCM3AP	80 kDa MCM3-assoc	-	47655047	47705236
NM_001270629	MED4	mediator of RNA pol	-	48649863	48669277
NM_014166	MED4	mediator of RNA pol	-	48649863	48669277
ENST00000539634	TMEM75	transmembrane prote	-	128959125	128960591
NM_152421	FAM69B	protein FAM69B	+	139607023	139619170
ENST00000491526	NHSL1	NHS-like 1 [Source:]	-	138768201	139013708
NM_002041	GABPB1	GA-binding protein s	-	50578076	50647605
NM_005254	GABPB1	GA-binding protein s	-	50569388	50647605
NM_016654	GABPB1	GA-binding protein s	-	50569388	50647605
NM_016655	GABPB1	GA-binding protein s	-	50578076	50647605
NM_181427	GABPB1	GA-binding protein s	-	50578076	50647605
NM_001113567	FAM211A	leucine-rich repeat-c	-	16345318	16395505
NM_207387	FAM211A	leucine-rich repeat-c	-	16345318	16395505
ENST00000497343	PPM1L	protein phosphatase,	+	160473389	160740250
NM_014205	ZNHIT2	zinc finger HIT doma	-	64883874	64885170
NM_001165031	DTYMK	thymidylate kinase is	-	242615156	242626383
NM_012145	DTYMK	thymidylate kinase is	-	242615156	242626383
NM_182603	ANKRD42	ankyrin repeat domai	+	82905290	82960013
NM_001204504	DGKH	diacylglycerol kinase	+	42614171	42803891
ENST00000435848	FIS1	fission 1 (mitochond	-	100882941	100895414

17.341341	6.314945	3.761827	2.645659	21.002962	13.17585
2937.58385	900.963438	11.074709	10.028402	2803.287	2804.8367
31.275145	9.928612	4.57992	3.521773	30.79649	36.809273
3439.117633	930.63824	11.270346	10.229386	2920.192	3649.7307
52.628528	16.001841	5.337701	4.070414	60.052982	74.80703
194.74838	18.546389	6.525658	4.500574	330.99893	35.242622
69.290104	9.799341	5.751368	3.514222	75.29869	92.783485
69.290104	9.799341	5.751368	3.514222	75.29869	92.783485
69.290104	9.799341	5.751368	3.514222	75.29869	92.783485
69.290104	9.799341	5.751368	3.514222	75.29869	92.783485
69.290104	9.799341	5.751368	3.514222	75.29869	92.783485
989.563728	167.831162	9.184477	7.58788	552.58374	868.1611
311.790197	67.690258	7.791208	6.41232	260.77313	315.28763
311.790197	67.690258	7.791208	6.41232	260.77313	315.28763
311.790197	67.690258	7.791208	6.41232	260.77313	315.28763
1341.267772	166.643827	9.883431	7.661204	877.0788	916.6797
261.819833	80.17887	7.649765	6.625791	260.37454	318.20154
138.039595	11.086417	6.460839	3.658422	163.368	21.88066
27.166411	8.416845	4.368231	3.092519	43.091183	34.383213
30.420119	7.870265	4.382755	3.046978	32.37982	14.138102
120.877387	37.146033	6.543432	5.253046	140.75078	121.81378
1960.320117	425.448225	10.507396	9.066043	2304.4915	2630.6892
1960.320117	425.448225	10.507396	9.066043	2304.4915	2630.6892
114.032624	33.982923	6.466938	5.244139	108.23882	75.253555
4991.5496	881.190125	11.740212	10.066983	3471.6458	2870.5928
4991.5496	881.190125	11.740212	10.066983	3471.6458	2870.5928
4991.5496	881.190125	11.740212	10.066983	3471.6458	2870.5928
4991.5496	881.190125	11.740212	10.066983	3471.6458	2870.5928
26.685079	8.898132	4.377106	3.228052	22.602362	21.54552
26.685079	8.898132	4.377106	3.228052	22.602362	21.54552
26.685079	8.898132	4.377106	3.228052	22.602362	21.54552
36.558511	11.118216	4.807245	3.705471	59.150547	19.261902
38.357729	7.220526	4.822283	3.010883	36.26363	16.37106
2090.693233	266.06498	10.501369	8.415453	3119.334	2959.4937
31.313444	6.264698	4.501661	2.757579	36.624954	53.22727
551.801183	143.450455	8.591384	7.345859	530.0662	263.91074
551.801183	143.450455	8.591384	7.345859	530.0662	263.91074
936.570662	144.462248	9.165306	7.325601	547.49884	402.05524
430.365267	131.415423	8.31005	7.279487	378.13397	426.58786
91.782202	26.400514	6.140853	4.962432	108.38679	105.13261
58.33407	13.880863	5.18815	4.003881	39.9807	15.839849
119.807889	17.185398	6.546201	4.339186	123.59412	90.594635
379.967563	23.461199	7.122745	3.509942	625.4194	788.8231
379.967563	23.461199	7.122745	3.509942	625.4194	788.8231
379.967563	23.461199	7.122745	3.509942	625.4194	788.8231
379.967563	23.461199	7.122745	3.509942	625.4194	788.8231
379.967563	23.461199	7.122745	3.509942	625.4194	788.8231
50.333266	14.892615	5.171331	4.150401	34.913563	24.23906
22.427806	6.222889	4.102582	2.895618	21.226168	25.829376
22.427806	6.222889	4.102582	2.895618	21.226168	25.829376
635.114442	172.271792	8.77621	7.533418	479.8107	288.56363
116.036537	33.927001	6.469632	5.334698	135.38298	144.07681
34.906434	9.568376	4.746984	3.354667	40.624386	44.70674
908.17266	93.937987	8.675972	6.735059	400.87244	336.01675
52.588298	12.295293	5.148129	3.916688	60.937458	89.770096
212.643362	19.684587	7.236823	4.546186	209.27873	96.63413
594.613518	85.100718	8.765575	6.720114	667.0389	865.0813
594.613518	85.100718	8.765575	6.720114	667.0389	865.0813
74.96026	27.65377	5.849185	4.811485	85.67026	85.37416
549.52166	38.40619	8.453357	5.522383	857.99725	967.81195
23.559959	7.745967	4.18201	3.056453	22.37207	10.69984
1258.290517	350.881634	9.808209	8.622199	902.78595	970.4417

27.140553	14.0023	13.03001	15.696369	4.9999995	4.9999995
2642.0815	3959.3318	2092.1372	3323.8289	1009.0178	398.30917
19.369162	22.145544	39.893055	38.637344	12.367258	10.000041
1945.9205	4500.1265	3031.9705	4586.7656	876.86664	713.9443
49.2646	36.206955	51.083824	44.35578	18.069654	6.2871137
580.63245	50.014706	93.772354	77.829216	14.58235	16.59602
67.65658	64.64183	50.21684	65.1432	13.260829	7.1260695
67.65658	64.64183	50.21684	65.1432	13.260829	7.1260695
67.65658	64.64183	50.21684	65.1432	13.260829	7.1260695
67.65658	64.64183	50.21684	65.1432	13.260829	7.1260695
67.65658	64.64183	50.21684	65.1432	13.260829	7.1260695
230.26758	495.11115	2035.969	1755.2898	127.72277	169.91849
185.69312	560.59186	160.6693	387.72614	75.18758	75.05263
185.69312	560.59186	160.6693	387.72614	75.18758	75.05263
185.69312	560.59186	160.6693	387.72614	75.18758	75.05263
833.42773	1247.018	2031.7488	2141.6536	140.8621	181.90013
190.96881	220.04312	269.43945	311.89154	87.68435	57.733562
269.46936	97.69409	126.67162	149.15384	9.580966	5.1379037
23.12965	24.806192	23.621082	13.967148	7.3995724	4.9999995
60.533165	11.36771	35.90037	28.201546	9.277977	5.084067
96.80452	92.69031	117.44233	155.7626	40.97945	14.593278
1773.2217	1638.4423	1498.2333	1916.8427	331.98083	255.1202
1773.2217	1638.4423	1498.2333	1916.8427	331.98083	255.1202
120.031204	117.03628	115.487854	148.14803	38.598873	19.01997
3396.7795	4458.339	7488.9775	8262.963	1041.954	405.0116
3396.7795	4458.339	7488.9775	8262.963	1041.954	405.0116
3396.7795	4458.339	7488.9775	8262.963	1041.954	405.0116
3396.7795	4458.339	7488.9775	8262.963	1041.954	405.0116
26.185568	21.597954	26.111372	42.067696	10.609251	4.9999995
26.185568	21.597954	26.111372	42.067696	10.609251	4.9999995
26.185568	21.597954	26.111372	42.067696	10.609251	4.9999995
39.59421	41.57998	27.267504	32.49692	10.377052	8.388899
58.32171	24.865032	45.399	48.925945	9.014354	5.151454
2563.7424	1587.6163	944.0628	1369.9102	239.36502	220.6381
14.200449	42.066807	18.237633	23.523554	4.9999995	8.269627
741.2823	910.4559	285.85876	579.2332	180.12357	73.4247
741.2823	910.4559	285.85876	579.2332	180.12357	73.4247
665.55194	2296.8904	411.09735	1296.3302	83.648796	54.945526
257.28152	649.2621	337.75128	533.17487	149.76431	65.19156
101.548904	57.722267	93.73334	84.1693	33.018936	26.824274
63.469196	127.04979	16.9756	86.689285	11.754152	7.1887364
135.16493	121.08438	104.86449	143.54478	12.577131	11.471699
69.338646	9.545171	551.1985	235.48056	23.684843	4.9999995
69.338646	9.545171	551.1985	235.48056	23.684843	4.9999995
69.338646	9.545171	551.1985	235.48056	23.684843	4.9999995
69.338646	9.545171	551.1985	235.48056	23.684843	4.9999995
69.338646	9.545171	551.1985	235.48056	23.684843	4.9999995
35.308952	103.33629	38.34072	65.86101	13.298459	11.74649
17.41297	13.56739	24.18855	32.342384	6.9791346	6.670179
17.41297	13.56739	24.18855	32.342384	6.9791346	6.670179
688.24927	1113.4176	372.40005	868.2454	205.96603	68.54839
94.04755	74.61373	132.43079	115.66736	32.37604	21.41135
30.41502	21.756443	42.51107	29.424944	8.185029	4.9999995
387.15244	2835.2593	107.06803	1382.667	85.416695	142.6715
28.209291	81.97862	14.304423	40.3299	13.343161	10.941849
267.37103	368.02957	112.80491	221.7418	16.18585	13.748661
373.10452	386.92395	608.75854	666.7739	78.74722	74.75184
373.10452	386.92395	608.75854	666.7739	78.74722	74.75184
74.0477	45.800503	76.99405	81.874886	34.0356	11.636329
707.00574	178.86864	276.59558	308.8508	51.01129	60.529144
33.312363	28.00186	18.521631	28.45199	7.156559	5.350254
812.04175	2084.6921	1005.8459	1773.9357	412.88608	101.920494

12.712511	4.9999995	4.9999995	5.17716	3.9345727	3.7589092
1883.7212	615.7081	911.51996	587.5044	10.970824	11.036021
4.9999995	14.58656	9.71089	7.9069257	4.4884305	5.108135
1002.87024	771.0187	1357.718	861.41156	11.0246725	11.413313
34.014175	16.832253	11.72325	9.084601	5.4492054	6.038421
19.89164	25.906084	16.768589	17.53365	7.9141436	5.0557775
9.597082	5.579149	14.10222	9.130694	5.780515	6.325285
9.597082	5.579149	14.10222	9.130694	5.780515	6.325285
9.597082	5.579149	14.10222	9.130694	5.780515	6.325285
9.597082	5.579149	14.10222	9.130694	5.780515	6.325285
9.597082	5.579149	14.10222	9.130694	5.780515	6.325285
78.55771	109.37683	339.1812	182.22997	8.625574	9.427729
67.75954	75.635475	59.86197	52.644352	7.5689297	8.024633
67.75954	75.635475	59.86197	52.644352	7.5689297	8.024633
67.75954	75.635475	59.86197	52.644352	7.5689297	8.024633
71.76428	228.61455	203.75114	172.97076	9.309738	9.505845
112.7563	68.397575	92.59609	61.905342	7.566424	8.03762
14.095072	8.559048	15.84898	13.296532	6.8944926	4.415666
14.95578	7.84061	4.9999995	10.30511	4.9704556	5.022216
14.669509	8.190039	4.9999995	4.9999995	4.5576544	3.852475
89.19609	35.979034	20.36686	21.761484	6.6840997	6.70318
424.78638	531.2593	579.2205	430.32214	10.695556	10.942325
424.78638	531.2593	579.2205	430.32214	10.695556	10.942325
66.35267	22.12261	36.41706	21.386354	6.3117275	6.0465374
1523.9691	870.37335	740.45807	705.37463	11.286921	11.067009
1523.9691	870.37335	740.45807	705.37463	11.286921	11.067009
1523.9691	870.37335	740.45807	705.37463	11.286921	11.067009
1523.9691	870.37335	740.45807	705.37463	11.286921	11.067009
11.072381	6.349895	14.291089	6.066175	4.0369625	4.3927336
11.072381	6.349895	14.291089	6.066175	4.0369625	4.3927336
11.072381	6.349895	14.291089	6.066175	4.0369625	4.3927336
8.870218	18.620491	12.817282	7.635352	5.4262314	4.251554
10.734108	4.9999995	8.423238	4.9999995	4.7162933	4.045841
227.65602	373.01624	257.27914	278.43536	11.130484	11.10604
4.9999995	9.318565	4.9999995	4.9999995	4.7294884	5.592175
287.96167	92.9807	131.81357	94.39852	8.565757	7.7784724
287.96167	92.9807	131.81357	94.39852	8.565757	7.7784724
89.705734	287.8901	159.21297	191.37036	8.613816	8.348345
235.99745	117.49762	116.948	103.0936	8.1036625	8.426911
36.274323	14.836959	27.39138	20.057215	6.3133197	6.4993467
9.279189	22.449163	17.637032	14.976903	4.860441	4.004207
13.55842	29.58731	19.440622	16.477207	6.4979596	6.2950754
96.79406	5.2882934	4.9999995	4.9999995	8.813105	9.289527
96.79406	5.2882934	4.9999995	4.9999995	8.813105	9.289527
96.79406	5.2882934	4.9999995	4.9999995	8.813105	9.289527
96.79406	5.2882934	4.9999995	4.9999995	8.813105	9.289527
96.79406	5.2882934	4.9999995	4.9999995	8.813105	9.289527
22.10957	17.000092	10.3925905	14.80849	4.665109	4.5563664
5.5717106	7.431926	5.684382	4.9999995	3.9485388	4.6426964
5.5717106	7.431926	5.684382	4.9999995	3.9485388	4.6426964
377.12997	87.43064	184.79266	109.76306	8.428621	7.9020243
56.837345	26.279224	32.8964	33.761646	6.6298532	6.9382973
12.387219	12.20949	12.43072	7.1977983	4.8810954	5.3637314
28.063381	146.14001	71.3274	90.008934	8.188149	8.10952
12.49364	12.356279	12.27389	12.36294	5.4708495	6.2842546
29.5532	25.886732	17.197847	15.535232	7.255569	6.37757
67.97239	76.40599	130.0395	82.68737	8.911175	9.422739
67.97239	76.40599	130.0395	82.68737	8.911175	9.422739
62.033768	12.000501	28.48401	17.732412	5.9674797	6.219793
30.749327	30.974737	28.177143	28.995499	9.281862	9.579651
8.861109	4.9999995	15.107879	4.9999995	4.0217915	3.487864
697.1309	301.35797	321.01038	270.98398	9.348302	9.581283

4.4530163	3.229476	3.6422966	3.5526924	2.3467326	2.4279919
11.00899	11.6064625	10.746457	11.079502	10.289848	9.503888
3.953847	3.969763	5.1584783	4.800864	3.857448	4.104079
10.569022	11.7914	11.268146	11.555522	10.08823	10.331934
5.306449	4.7449207	5.497911	4.9893017	4.4136496	3.4039397
8.820725	5.251429	6.340445	5.771427	4.096079	4.8677244
5.7719064	5.6419253	5.4734282	5.515151	3.9568033	3.5945632
5.7719064	5.6419253	5.4734282	5.515151	3.9568033	3.5945632
5.7719064	5.6419253	5.4734282	5.515151	3.9568033	3.5945632
5.7719064	5.6419253	5.4734282	5.515151	3.9568033	3.5945632
5.7719064	5.6419253	5.4734282	5.515151	3.9568033	3.5945632
7.5247645	8.639454	10.703518	10.185821	7.230088	8.255551
7.2154703	8.808239	7.103531	8.0264435	6.457227	7.0756
7.2154703	8.808239	7.103531	8.0264435	6.457227	7.0756
7.2154703	8.808239	7.103531	8.0264435	6.457227	7.0756
9.351081	9.967498	10.699966	10.466457	7.367026	8.35696
7.2560787	7.470888	7.831473	7.7361064	6.684647	6.70329
7.7381287	6.258562	6.763799	6.6943846	3.4792402	3.0907724
4.2227345	4.152633	4.452562	3.388782	3.0812142	2.4279919
5.6084337	2.9032662	5.012658	4.3620405	3.427131	3.076306
6.2738523	6.18273	6.656803	6.7599277	5.592544	4.6777444
10.432134	10.374018	10.278591	10.32175	8.643317	8.85311
10.432134	10.374018	10.278591	10.32175	8.643317	8.85311
6.586121	6.5378366	6.633688	6.68572	5.5032907	5.07296
11.362432	11.771961	12.555501	12.397449	10.333155	9.529542
11.362432	11.771961	12.555501	12.397449	10.333155	9.529542
11.362432	11.771961	12.555501	12.397449	10.333155	9.529542
11.362432	11.771961	12.555501	12.397449	10.333155	9.529542
4.4002595	3.9299695	4.587537	4.9151745	3.6341178	2.4279919
4.4002595	3.9299695	4.587537	4.9151745	3.6341178	2.4279919
4.4002595	3.9299695	4.587537	4.9151745	3.6341178	2.4279919
4.9926424	4.962196	4.64349	4.567354	3.6028914	3.8382626
5.550839	4.156689	5.339569	5.124469	3.386055	3.093895
10.973277	10.330931	9.633047	9.8344345	8.153923	8.641392
3.5030398	4.981494	4.0964947	4.107277	2.3467326	3.8159783
9.185588	9.51058	7.914412	8.593494	7.745798	7.043944
9.185588	9.51058	7.914412	8.593494	7.745798	7.043944
9.011743	10.83495	8.422269	9.760712	6.6106086	6.631062
7.673708	9.030386	8.145838	8.479794	7.4645443	6.8803277
6.3483076	5.4676833	6.3394814	5.8769794	5.274919	5.580175
5.682138	6.6593595	4.0043583	5.918398	3.7846107	3.6080103
6.7538023	6.588646	6.4987645	6.6429596	3.8804686	4.3107786
5.805438	2.6431284	8.84956	7.335711	4.8063755	2.4279919
5.805438	2.6431284	8.84956	7.335711	4.8063755	2.4279919
5.805438	2.6431284	8.84956	7.335711	4.8063755	2.4279919
5.805438	2.6431284	8.84956	7.335711	4.8063755	2.4279919
5.805438	2.6431284	8.84956	7.335711	4.8063755	2.4279919
4.825527	6.340822	5.107203	5.5329585	3.961549	4.345438
3.8016653	3.1775806	4.4836073	4.5614047	2.9984167	3.4975178
3.8016653	3.1775806	4.4836073	4.5614047	2.9984167	3.4975178
9.065356	9.798503	8.28551	9.177244	7.9314446	6.9494414
6.232277	5.861451	6.8245177	6.331398	5.2476974	5.251569
4.6186566	3.940665	5.252078	4.4256787	3.236695	2.4279919
8.247144	11.136646	6.527737	9.846637	6.6441875	8.007623
4.5086327	5.9986544	3.767781	4.858601	3.965542	4.242084
7.7271056	8.202548	6.6030097	7.255137	4.249222	4.58448
8.19394	8.276066	8.986283	8.803245	6.523743	7.0678725
8.19394	8.276066	8.986283	8.803245	6.523743	7.0678725
5.895338	5.111562	6.0629215	5.8380156	5.3201237	4.3307014
9.111173	7.1583014	7.8667803	7.7223754	5.9094243	6.7742696
4.7454233	4.342375	4.1188745	4.375734	3.0326693	3.1529326
9.310479	10.694319	9.713501	10.201367	8.946791	7.5214567

3.7490194	2.3408759	2.3618152	2.64752
10.82003	9.73262	10.078104	9.745923
2.350709	4.0013022	3.5612116	3.2558897
9.904636	10.059139	10.666222	10.326158
5.100188	4.214951	3.8262122	3.463544
4.356949	4.886185	4.3416862	4.454823
3.3752956	2.5986176	4.0893188	3.4707363
3.3752956	2.5986176	4.0893188	3.4707363
3.3752956	2.5986176	4.0893188	3.4707363
3.3752956	2.5986176	4.0893188	3.4707363
3.3752956	2.5986176	4.0893188	3.4707363
6.260869	7.117462	8.643317	8.019993
6.061576	6.561231	6.170115	6.1481705
6.061576	6.561231	6.170115	6.1481705
6.061576	6.561231	6.170115	6.1481705
6.136952	8.247144	7.9212956	7.937844
6.7802334	6.404502	6.784346	6.397729
3.887465	3.192993	4.25739	4.042673
3.9674692	3.0586014	2.3618152	3.6580207
3.9411843	3.1226819	2.3618152	2.352752
6.4472156	5.400522	4.613391	4.7868567
8.683948	9.511481	9.410817	9.293583
8.683948	9.511481	9.410817	9.293583
6.030771	4.637021	5.4599223	4.76087
10.512869	10.244089	9.760712	10.021529
10.512869	10.244089	9.760712	10.021529
10.512869	10.244089	9.760712	10.021529
10.512869	10.244089	9.760712	10.021529
3.5671906	2.7695398	4.108478	2.8609965
3.5671906	2.7695398	4.108478	2.8609965
3.5671906	2.7695398	4.108478	2.8609965
3.268405	4.368171	3.955556	3.1995404
3.5244231	2.3408759	3.3672965	2.352752
7.7731857	9.000512	8.26203	8.661674
2.350709	3.3174844	2.3618152	2.352752
8.11505	6.8686066	7.28551	7.0162473
8.11505	6.8686066	7.28551	7.0162473
6.456397	8.6130705	7.5456038	8.096863
7.8293486	7.2309523	7.121126	7.150624
5.1851177	4.027316	5.048232	4.658831
3.332369	4.6611667	4.414396	4.2227345
3.8357491	5.095924	4.5463896	4.3658075
6.5592103	2.5515056	2.3618152	2.352752
6.5592103	2.5515056	2.3618152	2.352752
6.5592103	2.5515056	2.3618152	2.352752
6.5592103	2.5515056	2.3618152	2.352752
6.5592103	2.5515056	2.3618152	2.352752
4.5005097	4.2291527	3.659387	4.206368
2.7008371	2.983921	2.8402622	2.352752
2.7008371	2.983921	2.8402622	2.352752
8.511303	6.7814245	7.779081	7.2478113
5.817466	4.907438	5.30984	5.4741764
3.714735	3.7257423	3.9118285	3.1110115
4.831638	7.5653086	6.4202304	6.941366
3.7249858	3.7428057	3.8944898	3.93022
4.903884	4.885249	4.376144	4.278139
6.0653067	6.576402	7.2674937	6.8198676
6.0653067	6.576402	7.2674937	6.8198676
5.9400697	3.6965852	5.1088867	4.472545
4.9573803	5.163886	5.091035	5.238302
3.2673018	2.3408759	4.192189	2.352752
9.390692	8.687897	8.566363	8.619994

ASHGA5P043242	0.020856138	0.088872205	2.2752255	up	noncoding
ASHGA5P032536	0.049117452	0.1440311	3.9706496	up	noncoding
ASHGA5P038081	0.00111953	0.022608782	4.8684154	up	noncoding
ASHGA5P038081	0.00111953	0.022608782	4.8684154	up	noncoding
ASHGA5P038081	0.00111953	0.022608782	4.8684154	up	noncoding
ASHGA5P018764	0.000639658	0.017037535	5.0773539	up	noncoding
ASHGA5P035475	1.11203E-05	0.004010044	7.1071469	up	noncoding
ASHGA5P016921	0.000953749	0.021003113	3.9420906	up	noncoding
ASHGA5P048053	0.000166843	0.011182026	2.62284	up	noncoding
ASHGA5P046681	0.003153485	0.036407477	3.7319341	up	noncoding
ASHGA5P030019	0.006557425	0.051498397	2.458728	up	noncoding
ASHGA5P055800	0.000409363	0.014460542	2.3999577	up	noncoding
ASHGA5P055866	0.002777068	0.034115169	3.4025846	up	noncoding
ASHGA5P025629	0.009629409	0.060887465	2.4412165	up	noncoding
ASHGA5P014877	0.001173357	0.0230268	2.4589675	up	noncoding
ASHGA5P038690	0.000327976	0.013141059	3.3692734	up	noncoding
ASHGA5P031686	0.0095397	0.060604099	4.1193547	up	noncoding
ASHGA5P034007	0.004134968	0.041062628	2.523383	up	noncoding
ASHGA5P026664	0.013786063	0.071982793	2.0067368	up	noncoding
ASHGA5P014247	0.014135261	0.072947894	2.1577486	up	noncoding
ASHGA5P014247	0.014135261	0.072947894	2.1577486	up	noncoding
ASHGA5P014247	0.014135261	0.072947894	2.1577486	up	noncoding
ASHGA5P014247	0.014135261	0.072947894	2.1577486	up	noncoding
ASHGA5P014247	0.014135261	0.072947894	2.1577486	up	noncoding
ASHGA5P023120	0.003416172	0.037690616	2.3557256	up	noncoding
ASHGA5P056347	8.41892E-05	0.008738697	6.1091287	up	noncoding
ASHGA5P033820	0.025788014	0.100086328	2.0009534	up	noncoding
ASHGA5P035735	0.002125544	0.030380707	4.8160307	up	noncoding
ASHGA5P035735	0.002125544	0.030380707	4.8160307	up	noncoding
ASHGA5P007789	0.029906939	0.108404427	2.7574114	up	noncoding
ASHGA5P053608	0.020493826	0.087825608	2.1395082	up	noncoding
ASHGA5P048394	0.000612936	0.016760351	2.2054124	up	noncoding
ASHGA5P048394	0.000612936	0.016760351	2.2054124	up	noncoding
ASHGA5P048394	0.000612936	0.016760351	2.2054124	up	noncoding
ASHGA5P048394	0.000612936	0.016760351	2.2054124	up	noncoding
ASHGA5P033959	0.015975452	0.077348	3.1941152	up	noncoding
ASHGA5P050948	0.003874809	0.039898314	2.4393848	up	noncoding
ASHGA5P018194	0.00033896	0.013255256	8.4584532	up	noncoding
ASHGA5P018194	0.00033896	0.013255256	8.4584532	up	noncoding
ASHGA5P018194	0.00033896	0.013255256	8.4584532	up	noncoding
ASHGA5P018194	0.00033896	0.013255256	8.4584532	up	noncoding
ASHGA5P047340	0.001187572	0.023096268	2.8997013	up	noncoding
ASHGA5P037355	0.01616481	0.077740057	6.4395396	up	noncoding
ASHGA5P026794	0.003255551	0.036951034	2.1287235	up	noncoding
ASHGA5P026794	0.003255551	0.036951034	2.1287235	up	noncoding
ASHGA5P032812	0.024534476	0.097200103	17.4824588	up	noncoding
ASHGA5P022885	0.046036201	0.138218388	2.3123502	up	noncoding
ASHGA5P046834	0.005774767	0.048306083	6.0477946	up	noncoding
ASHGA5P021725	0.000653946	0.017168766	2.0792399	up	noncoding
ASHGA5P021725	0.000653946	0.017168766	2.0792399	up	noncoding
ASHGA5P021725	0.000653946	0.017168766	2.0792399	up	noncoding
ASHGA5P031178	0.000484002	0.015384974	23.4623194	up	noncoding
ASHGA5P044309	9.83814E-05	0.009080764	2.2332406	up	noncoding
ASHGA5P042272	6.00609E-05	0.007803818	2.4247994	up	noncoding
ASHGA5P042272	6.00609E-05	0.007803818	2.4247994	up	noncoding
ASHGA5P031284	0.000624882	0.016926568	4.0532154	up	noncoding
ASHGA5P037709	0.008073421	0.056530276	3.4161706	up	noncoding
ASHGA5P021185	0.031021831	0.110553198	2.1002003	up	noncoding
ASHGA5P021185	0.031021831	0.110553198	2.1002003	up	noncoding
ASHGA5P021185	0.031021831	0.110553198	2.1002003	up	noncoding
ASHGA5P037031	0.005664275	0.047783478	3.4713843	up	noncoding
ASHGA5P042987	0.000181327	0.011439647	2.7169309	up	noncoding

uc010lhv.1	AK091729	UCSC_knowngene	1986	chr7	-
ENST00000508851	RP11-893F2.9	GENCODE	750	chr17	-
NR_004428	EGOT	RefSeq	1529	chr3	-
NR_004428	EGOT	RefSeq	1529	chr3	-
NR_004428	EGOT	RefSeq	1529	chr3	-
ENST00000457407	AC093388.3	GENCODE	404	chr2	+
ENST00000436557	AC008174.3	GENCODE	689	chr2	-
ENST00000435718	RP4-564F22.2	GENCODE	261	chr20	-
ENST00000446167	RP11-7O11.3	GENCODE	486	chr1	-
ENST00000440604	RP11-308D16.4	GENCODE	729	chrX	+
ENST00000558372	CTD-3110H11.1	GENCODE	612	chr15	-
ENST00000416599	RP11-64P12.8	GENCODE	3494	chr13	-
TCONS_00003943	XLOC_001757	LincRNAs identified	314	chr2	+
NR_015410	LINC00340	RefSeq	1904	chr6	+
NR_038200	M1	RefSeq	554	chr10	-
ENST00000326237	AC034193.5	GENCODE	2882	chr3	+
ENST00000576745	RP11-388M20.9	GENCODE	1201	chr16	+
ENST00000453935	RP4-669L17.4	GENCODE	498	chr1	+
ENST00000447748	RP11-182I10.3	GENCODE	2804	chr1	-
NR_073005	DUS4L	RefSeq	2012	chr7	+
NR_073005	DUS4L	RefSeq	2012	chr7	+
NR_073005	DUS4L	RefSeq	2012	chr7	+
NR_073005	DUS4L	RefSeq	2012	chr7	+
NR_073005	DUS4L	RefSeq	2012	chr7	+
uc003tez.3	BC043264	UCSC_knowngene	1729	chr7	+
TCONS_00003615	XLOC_001406	LincRNAs identified	2194	chr2	+
ENST00000579160	RP11-746B8.1	GENCODE	387	chr18	+
uc002rcs.1	AX748233	UCSC_knowngene	2612	chr2	+
uc002rcs.1	AX748233	UCSC_knowngene	2612	chr2	+
uc002tfj.3	BC113052	UCSC_knowngene	1412	chr2	+
NR_036490	LOC284648	RefSeq	1504	chr1	+
ENST00000526310	RP11-864G5.3	GENCODE	628	chr11	-
ENST00000526310	RP11-864G5.3	GENCODE	628	chr11	-
ENST00000526310	RP11-864G5.3	GENCODE	628	chr11	-
ENST00000526310	RP11-864G5.3	GENCODE	628	chr11	-
TCONS_00003103	XLOC_001850	LincRNAs identified	228	chr2	+
NR_049777	SRG7	RefSeq	476	chr1	+
ENST00000450469	RP4-784A16.3	GENCODE	237	chr1	-
ENST00000450469	RP4-784A16.3	GENCODE	237	chr1	-
ENST00000450469	RP4-784A16.3	GENCODE	237	chr1	-
ENST00000450469	RP4-784A16.3	GENCODE	237	chr1	-
TCONS_00017432	XLOC_008014	LincRNAs identified	1773	chrX	+
ENST00000456880	AP001065.2	GENCODE	2056	chr21	-
ENST00000546273	RP11-713B9.1	GENCODE	301	chr11	+
ENST00000546273	RP11-713B9.1	GENCODE	301	chr11	+
TCONS_00029017	XLOC_013940	LincRNAs identified	447	chr21	+
ENST00000564598	RP11-20I23.12	GENCODE	551	chr16	-
ENST00000422807	RP11-445N18.5	GENCODE	535	chr10	-
ENST00000539303	SNHG1	GENCODE	240	chr11	-
ENST00000539303	SNHG1	GENCODE	240	chr11	-
ENST00000539303	SNHG1	GENCODE	240	chr11	-
ENST00000560208	CRNDE	GENCODE	735	chr16	-
ENST00000521127	SNHG6	GENCODE	560	chr8	-
ENST00000433182	E2F3-IT1	GENCODE	400	chr6	+
ENST00000433182	E2F3-IT1	GENCODE	400	chr6	+
ENST00000444470	GAS5	GENCODE	424	chr1	-
ENST00000415054	RP1-85F18.6	GENCODE	357	chr22	-
NR_040008	CAPN1	RefSeq	3040	chr11	+
NR_040008	CAPN1	RefSeq	3040	chr11	+
NR_040008	CAPN1	RefSeq	3040	chr11	+
ENST00000429167	FAM83C-AS1	GENCODE	352	chr20	+
uc003tdf.1	RP9P	UCSC_knowngene	3348	chr7	-

100893615	100895601		TCCACAGGCATAGAATCCATGTGAC		exon sense-overlappi
48358937	48365216		TGGAAGTGTGGTGAGTGATTGACTT		intergenic
4790877	4793274		TAGCTATTTGCCA	100126791	intronic antisense
4790877	4793274		TAGCTATTTGCCA	100126791	intronic antisense
4790877	4793274		TAGCTATTTGCCA	100126791	intronic antisense
191399580	191432828		GATGGCACTATGGAAAGTAGCTAAA		bidirectional
186648417	186664877		GCTTGCTATGAAAGATTCAAGTGAT		natural antisense
37049531	37059692		TTCAGTGGCTGTTGCAGAGGAAGAC		intergenic
44410041	44411974	ENST00000412378	TAAGTCTTTGGGAGCCAGAAGGGCT		bidirectional
136075712	136103777		CTTGTGATTACTTGCTCAGAAGTTA		intergenic
50651646	50652391		GGTTCTTCTGACTCCCTTCATCTGTC		intergenic
52741843	52768600	uc001vgl.1	CATCTTTCCAGGACAAAAAATAATA		intergenic
173188049	173232618		TAAACCACAGGTTTGATCTCAGTTA		intergenic
21666674	22194616	uc011djk.2	ATCGCTGGGAAT	401237	intergenic
61496747	61513203	NR_038199	GATTAATTCAGG	100507027	intergenic
10028594	10046928		ACCAATGTGCCACCATGGATATTCA		natural antisense
31237280	31238481		TAGCTAAGGGATTATTTGGTTGTAA		intergenic
450886	453942	ENST00000438217	GCGTCGTTGGATGCTGTATGACAAT		intergenic
65437907	65458695		GCATACACCCTATCAGGGTCTAAAA		intergenic
107204401	107218968	NR_073003, NR_07	TACCACAAATCA	11062	exon sense-overlappi
107204401	107218968	NR_073003, NR_07	TACCACAAATCA	11062	exon sense-overlappi
107204401	107218968	NR_073003, NR_07	TACCACAAATCA	11062	natural antisense
107204401	107218968	NR_073003, NR_07	TACCACAAATCA	11062	natural antisense
107204401	107218968	NR_073003, NR_07	TACCACAAATCA	11062	natural antisense
36134922	36140262		AATGGAGAAAAGCGTGGAATGCTCT		intergenic
27938797	27978571		TCTGATGAAGCTCTCCTTTGGAGTA		intergenic
30415062	30416276		CATCTGGAAAAAACAAGCAAAGCTC		intergenic
17935161	17937773		GACATCAATATACTTATTCATTATA		exon sense-overlappi
17935161	17937773		GACATCAATATACTTATTCATTATA		exon sense-overlappi
110745137	110752147	NR_046111, uc002tf	TCTATCAAAGTTTGCAAGGTTCATT		intergenic
182584274	182585764		TGCTCCGAAGCT	284648	intergenic
102100581	102101653		TACAATAACCATCCCAAGCTACAAC		natural antisense
102100581	102101653		TACAATAACCATCCCAAGCTACAAC		natural antisense
102100581	102101653		TACAATAACCATCCCAAGCTACAAC		natural antisense
102100581	102101653		TACAATAACCATCCCAAGCTACAAC		natural antisense
216444129	216447358		CTTGCCGTCCTTGATTTCAGCTAGCA		intergenic
109399838	109400962		CAATCAGCTTTGA	642864	intronic antisense
53757462	53757810		CCCCAGAACTGGACAGGGAAGAAA		intron sense-overlapp
53757462	53757810		CCCCAGAACTGGACAGGGAAGAAA		intron sense-overlapp
53757462	53757810		CCCCAGAACTGGACAGGGAAGAAA		intron sense-overlapp
53757462	53757810		CCCCAGAACTGGACAGGGAAGAAA		intron sense-overlapp
73045949	73047819	NR_003255	TACTTGAGTTCTGGGTTTTACTGCA		intergenic
45834477	45836623		CAGCCACCTATTTTGTACTCCTTGTT		intronic antisense
115045696	115046044		AGGAATGAAAGTTTCACACAGATTC		natural antisense
115045696	115046044		AGGAATGAAAGTTTCACACAGATTC		natural antisense
40383132	40386878		ACGAATCAGCAAACAAAGGAGAAAT		intergenic
2688691	2723398		CACCTAGCTGATTCTTGATGACATT		intergenic
45659675	45676875		GAGAGTGCCCTGAAATATTTGAAAT		intergenic
62621044	62622646		TCAGAGCTGAGA	23642	bidirectional
62621044	62622646		TCAGAGCTGAGA	23642	bidirectional
62621044	62622646		TCAGAGCTGAGA	23642	bidirectional
54958770	54963061		ATTATTGATAATA	643911	intergenic
67834505	67837776		AATTACTGACAT	641638	intergenic
20438051	20440409		CTCCACTTACTACATATCAAGGATT		intron sense-overlapp
20438051	20440409		CTCCACTTACTACATATCAAGGATT		intron sense-overlapp
173833091	173834043	ENST00000422207,	GGAGATTTTGGA	60674	intergenic
41565193	41579148		CTGGCACATACACATCAATCAGTAT		natural antisense
64949303	64979477		TTCCTCCAAGAC	823	exon sense-overlappi
64949303	64979477		TTCCTCCAAGAC	823	exon sense-overlappi
64949303	64979477		TTCCTCCAAGAC	823	exon sense-overlappi
33873053	33873559		CTCCTCAAAGTTCAGGATACACCAG		natural antisense
32961114	32982159		TCTGGGACCTTA	441212	intronic antisense

ENST00000474120	FIS1	fission 1 (mitochondı	-	100882874	100895597
NM_001099952	ITPR1	inositol 1,4,5-trispho	+	4535031	4889524
NM_001168272	ITPR1	inositol 1,4,5-trispho	+	4535031	4889524
NM_002222	ITPR1	inositol 1,4,5-trispho	+	4535031	4889524
NM_001142645	TMEM194B	transmembrane prote	-	191371618	191399468
NM_173651	FSIP2	fibrous sheath-interac	+	186603354	186698016
NM_014652	IPO13	importin-13	+	44412477	44433694
ENST00000245046	EMC3	ER membrane protei	-	10004220	10028804
NM_001270419	DUS4L	tRNA-dihydrouridine	+	107204401	107218968
NM_181581	DUS4L	tRNA-dihydrouridine	+	107204401	107218968
NM_001161520	COG5	conserved oligomeric	-	106848297	107204959
NM_006348	COG5	conserved oligomeric	-	106842188	107204959
NM_181733	COG5	conserved oligomeric	-	106842188	107204959
NM_001130009	GEN1	flap endonuclease GI	+	17935413	17966632
NM_182625	GEN1	flap endonuclease GI	+	17935176	17966632
NM_001130145	YAP1	yorkie homolog isofc	+	101981191	102104154
NM_001195044	YAP1	yorkie homolog isofc	+	101981191	102104154
NM_001195045	YAP1	yorkie homolog isofc	+	101983170	102104154
NM_006106	YAP1	yorkie homolog isofc	+	101981191	102104154
ENST00000357393	AKNAD1	AKNA domain conta	-	109365460	109506106
NM_001018054	LRP8	low-density lipoprote	-	53708040	53793821
NM_004631	LRP8	low-density lipoprote	-	53708040	53793821
NM_017522	LRP8	low-density lipoprote	-	53708040	53793821
NM_033300	LRP8	low-density lipoprote	-	53708040	53793821
NM_003307	TRPM2	transient receptor pot	+	45773483	45862964
NM_001098517	CADM1	cell adhesion molecu	-	115044344	115375241
NM_014333	CADM1	cell adhesion molecu	-	115044344	115375241
NM_001012662	SLC3A2	4F2 cell-surface antiş	+	62623483	62656355
NM_001012664	SLC3A2	4F2 cell-surface antiş	+	62623483	62656355
NM_002394	SLC3A2	4F2 cell-surface antiş	+	62623483	62656355
NM_001243076	E2F3	transcription factor E	+	20404033	20493945
NM_001949	E2F3	transcription factor E	+	20402136	20493945
NM_001429	EP300	histone acetyltransfeı	+	41488613	41576081
NM_001198868	CAPN1	calpain-1 catalytic su	+	64948685	64979477
NM_001198869	CAPN1	calpain-1 catalytic su	+	64949149	64979477
NM_005186	CAPN1	calpain-1 catalytic su	+	64949303	64979477
NM_178468	FAM83C	protein FAM83C	-	33873533	33880225
ENST00000404479	AVL9	AVL9 homolog (S. c	+	32535321	33078516

1258.290517	350.881634	9.808209	8.622199	902.78595	970.4417
330.417979	43.789126	7.655555	5.66618	357.74792	43.912132
49.481026	7.263521	5.247294	2.963842	60.63621	35.492496
49.481026	7.263521	5.247294	2.963842	60.63621	35.492496
49.481026	7.263521	5.247294	2.963842	60.63621	35.492496
47.743621	6.095834	5.038082	2.694006	34.2206	29.050005
708.775418	56.897263	8.751588	5.922318	1084.4419	522.55945
374.35764	56.056251	8.102359	6.123398	445.3553	437.1698
406.166352	94.79348	8.247662	6.856532	375.30408	367.826
354.602248	57.272866	7.874058	5.974134	396.28064	145.18181
76.091603	19.677933	5.844881	4.546969	100.5249	109.69317
205.288672	54.14631	7.299135	6.036126	232.1995	162.86865
35.844443	8.08762	4.772818	3.006187	46.15725	41.42834
41.71884	10.046014	4.719858	3.432258	38.925785	4.9999995
64.975771	19.093219	5.51908	4.221028	44.84784	28.770996
43.358494	8.702283	5.065357	3.31292	43.535946	52.158936
162.575323	21.574095	6.745264	4.702845	186.63904	33.666588
20.073657	5.719877	4.004658	2.669299	18.962297	16.58257
192.482714	52.098683	6.94904	5.944189	191.72299	115.066605
247.169627	70.700674	7.528056	6.418529	216.25089	249.40005
247.169627	70.700674	7.528056	6.418529	216.25089	249.40005
247.169627	70.700674	7.528056	6.418529	216.25089	249.40005
247.169627	70.700674	7.528056	6.418529	216.25089	249.40005
247.169627	70.700674	7.528056	6.418529	216.25089	249.40005
17.338622	5.453567	3.784844	2.548673	16.11249	21.921864
159.679227	16.828704	6.909517	4.298551	195.48799	247.1486
19.993082	7.916174	3.989056	2.988369	22.830044	22.426352
122.925856	20.920454	6.577731	4.309887	119.531105	103.47118
122.925856	20.920454	6.577731	4.309887	119.531105	103.47118
454.34275	120.383147	8.398056	6.934741	428.55023	467.79846
24.40123	9.190209	4.288721	3.191441	23.01765	19.631588
301.76687	90.770458	7.83861	6.697561	310.96146	221.44276
301.76687	90.770458	7.83861	6.697561	310.96146	221.44276
301.76687	90.770458	7.83861	6.697561	310.96146	221.44276
301.76687	90.770458	7.83861	6.697561	310.96146	221.44276
156.603903	38.099136	6.893121	5.217705	214.36281	193.70563
27.35224	7.334252	4.388043	3.101525	28.667418	33.20759
76.603512	6.420291	5.879152	2.798759	62.458794	53.13058
76.603512	6.420291	5.879152	2.798759	62.458794	53.13058
76.603512	6.420291	5.879152	2.798759	62.458794	53.13058
76.603512	6.420291	5.879152	2.798759	62.458794	53.13058
85.260935	16.928947	5.74439	4.208485	86.79832	115.11001
129.865114	8.827308	6.019572	3.332614	222.20952	237.72578
89.227174	25.82633	5.950724	4.860736	96.40833	23.43304
89.227174	25.82633	5.950724	4.860736	96.40833	23.43304
6687.080891	194.281128	10.655569	6.527732	5506.806	97.909966
34.628996	12.953721	4.773327	3.563967	38.147804	37.227848
187.724819	14.987244	6.661256	4.064847	318.8471	266.6033
614.438438	183.826024	8.829979	7.773922	553.43036	470.39764
614.438438	183.826024	8.829979	7.773922	553.43036	470.39764
614.438438	183.826024	8.829979	7.773922	553.43036	470.39764
259.816657	5.947452	7.16022	2.607946	223.81558	19.97728
4167.3279	1084.853693	11.571164	10.412025	4148.364	2941.968
186.07058	48.761351	7.173837	5.895972	193.09958	186.06898
186.07058	48.761351	7.173837	5.895972	193.09958	186.06898
6437.76685	894.757618	12.167091	10.148025	5794.997	4649.5415
109.617227	30.186941	6.38005	4.60767	169.87868	108.60919
122.805441	47.742651	6.530014	5.459487	145.89363	138.88104
122.805441	47.742651	6.530014	5.459487	145.89363	138.88104
122.805441	47.742651	6.530014	5.459487	145.89363	138.88104
156.217421	32.855164	6.893751	5.09824	138.41248	145.68909
140.588082	32.764154	6.735409	5.293431	152.99806	101.996284

812.04175	2084.6921	1005.8459	1773.9357	412.88608	101.920494
638.45636	211.93507	359.96564	370.49075	48.162457	57.690266
78.15306	43.1107	34.928696	44.564995	4.9999995	4.9999995
78.15306	43.1107	34.928696	44.564995	4.9999995	4.9999995
78.15306	43.1107	34.928696	44.564995	4.9999995	4.9999995
36.69402	21.16178	81.142654	84.19267	4.9999995	4.9999995
1655.7528	374.21704	233.3557	382.32562	80.83682	32.83636
447.37054	423.88147	166.12016	326.24857	46.083622	47.459045
311.14447	292.99353	531.39343	558.3366	92.39612	49.613647
658.9373	475.4046	124.4675	327.34164	55.53942	15.878791
51.398567	79.42163	50.017498	65.49385	15.760899	11.615182
277.03638	181.44984	156.17046	222.0072	60.7083	30.084446
44.678143	21.519999	36.30858	24.974346	12.02261	4.9999995
75.63082	38.384315	40.67774	51.69438	12.173339	4.9999995
54.869976	43.813538	111.50247	106.049805	9.673052	8.489222
26.844877	36.092743	52.40568	49.112785	8.094989	5.839463
298.39624	180.18561	93.96648	182.59798	16.258732	17.613834
16.58507	28.687967	17.371794	22.252241	4.9999995	8.502986
266.8133	337.79172	34.640858	208.86081	55.962532	35.04966
183.01465	381.64008	153.07909	299.633	82.59278	54.413635
183.01465	381.64008	153.07909	299.633	82.59278	54.413635
183.01465	381.64008	153.07909	299.633	82.59278	54.413635
183.01465	381.64008	153.07909	299.633	82.59278	54.413635
183.01465	381.64008	153.07909	299.633	82.59278	54.413635
13.031251	17.57249	16.7905	18.603136	5.0814486	4.9999995
130.96411	136.9563	105.29006	142.2283	18.0105	10.291361
19.690964	16.013968	16.257744	22.739418	5.5787673	4.9999995
124.90714	106.77701	118.678	164.1907	18.892002	6.2510633
124.90714	106.77701	118.678	164.1907	18.892002	6.2510633
298.86902	609.46075	319.65454	601.7235	160.59221	58.94444
25.225655	30.106	19.838037	28.588448	9.636254	4.9999995
399.8741	312.11554	219.6119	346.59546	107.446396	47.195328
399.8741	312.11554	219.6119	346.59546	107.446396	47.195328
399.8741	312.11554	219.6119	346.59546	107.446396	47.195328
399.8741	312.11554	219.6119	346.59546	107.446396	47.195328
154.51347	158.2308	121.552	97.258705	39.046047	17.040037
12.9498205	38.53525	23.400024	27.353336	6.2016997	8.897997
95.26995	60.36033	95.58217	92.81925	4.9999995	5.840568
95.26995	60.36033	95.58217	92.81925	4.9999995	5.840568
95.26995	60.36033	95.58217	92.81925	4.9999995	5.840568
95.26995	60.36033	95.58217	92.81925	4.9999995	5.840568
19.399286	34.967403	150.14551	105.14508	16.5102	19.282343
158.29227	6.428844	86.735886	67.798386	14.279897	8.449969
152.3604	111.407326	55.42426	96.32969	31.6529	12.00447
152.3604	111.407326	55.42426	96.32969	31.6529	12.00447
10878.724	15729.945	112.57008	7796.5303	25.551489	40.21799
30.924765	28.533026	29.97818	42.962353	14.6022	4.9999995
343.96158	24.476706	101.2995	71.16073	22.084408	26.69239
578.2668	423.74167	811.67786	849.1163	141.61026	121.302765
578.2668	423.74167	811.67786	849.1163	141.61026	121.302765
578.2668	423.74167	811.67786	849.1163	141.61026	121.302765
420.27893	464.10666	115.94137	314.78012	4.9999995	4.9999995
5474.3477	3523.571	3911.2407	5004.476	1011.8619	571.16
172.36029	146.04967	195.31142	223.53354	49.57457	41.57514
172.36029	146.04967	195.31142	223.53354	49.57457	41.57514
6405.129	9281.47	4174.5356	8320.928	652.7849	603.97626
115.42466	104.71178	72.44422	86.634834	45.778	6.2907233
142.71762	61.290276	129.06989	118.98019	47.91928	13.100169
142.71762	61.290276	129.06989	118.98019	47.91928	13.100169
142.71762	61.290276	129.06989	118.98019	47.91928	13.100169
122.06858	230.61938	106.196526	194.31847	36.17399	12.815191
193.78938	167.34338	82.31619	145.0852	37.471684	19.596743

697.1309	301.35797	321.01038	270.98398	9.348302	9.581283
47.413372	64.02873	19.141453	26.29848	8.023324	5.3405786
5.5015626	9.196589	10.051681	8.831296	5.462109	5.063734
5.5015626	9.196589	10.051681	8.831296	5.462109	5.063734
5.5015626	9.196589	10.051681	8.831296	5.462109	5.063734
6.7648	5.820306	8.989898	4.9999995	4.634572	4.798945
125.27391	36.52283	35.285805	30.627853	9.608767	8.717515
44.319454	83.58919	51.89145	62.994743	8.326399	8.464822
121.325386	97.84458	117.92859	89.65256	8.088139	8.2289915
97.287735	51.12119	77.70592	46.10414	8.174321	6.9488573
24.488657	20.404188	26.652067	19.146608	6.2015896	6.557655
65.44315	48.79788	73.25863	46.585453	7.40632	7.10531
13.659499	4.9999995	4.9999995	7.843615	5.0685134	5.2625294
13.40538	7.4061832	10.514869	11.776311	4.818704	2.3499577
20.848618	7.6221433	47.562733	20.363548	5.0282154	4.7870464
8.687705	4.9999995	13.769931	10.8216095	4.986884	5.5674644
19.470383	33.61823	24.380238	18.10315	7.0913277	4.994234
5.816277	4.9999995	4.9999995	4.9999995	3.7844949	4.0625954
78.002144	75.20706	28.824451	39.54625	7.1316414	6.623887
102.23582	49.915752	83.9548	51.091255	7.3053923	7.696294
102.23582	49.915752	83.9548	51.091255	7.3053923	7.696294
102.23582	49.915752	83.9548	51.091255	7.3053923	7.696294
102.23582	49.915752	83.9548	51.091255	7.3053923	7.696294
102.23582	49.915752	83.9548	51.091255	7.3053923	7.696294
7.440783	5.199169	4.9999995	4.9999995	3.5386198	4.419765
27.879553	15.786359	15.63296	13.3714905	7.161766	7.6849566
15.399421	7.748104	4.9999995	8.77075	4.050323	4.4485517
44.22104	6.2074623	31.351059	18.6001	6.4508348	6.476785
44.22104	6.2074623	31.351059	18.6001	6.4508348	6.476785
288.28375	46.960403	106.0444	61.47368	8.283695	8.5612955
16.20903	6.974661	12.321311	4.9999995	4.061588	4.276203
179.19989	60.83973	84.394714	65.54669	7.824967	7.535128
179.19989	60.83973	84.394714	65.54669	7.824967	7.535128
179.19989	60.83973	84.394714	65.54669	7.824967	7.535128
179.19989	60.83973	84.394714	65.54669	7.824967	7.535128
89.14887	10.26421	44.122128	28.973524	7.2939515	7.353887
7.573715	8.379484	4.9999995	7.952615	4.3884783	4.976894
4.9999995	12.198889	4.9999995	5.4822893	5.5092583	5.5904655
4.9999995	12.198889	4.9999995	5.4822893	5.5092583	5.5904655
4.9999995	12.198889	4.9999995	5.4822893	5.5092583	5.5904655
4.9999995	12.198889	4.9999995	5.4822893	5.5092583	5.5904655
9.444296	5.3062754	26.201897	24.828669	5.9853973	6.6245146
7.2309375	9.612844	8.390201	4.9999995	7.345891	7.6308746
44.374344	30.058376	15.146712	21.721178	6.1434126	4.5086327
44.374344	30.058376	15.146712	21.721178	6.1434126	4.5086327
20.445036	730.4443	23.152252	325.8757	11.922487	6.3975606
29.41766	8.074859	15.520007	5.107598	4.788298	5.124046
7.7906184	8.324977	12.9987	12.0323715	7.863238	7.793955
151.3331	140.16599	358.46494	190.07909	8.629734	8.568922
151.3331	140.16599	358.46494	190.07909	8.629734	8.568922
151.3331	140.16599	358.46494	190.07909	8.629734	8.568922
8.37407	7.3106437	4.9999995	4.9999995	7.3555274	4.2987247
1509.4186	878.8135	1543.6344	994.23376	11.533211	11.095217
73.47551	39.52516	51.11404	37.303684	7.1417656	7.2986503
73.47551	39.52516	51.11404	37.303684	7.1417656	7.2986503
737.58655	976.9606	1438.8883	958.3491	12.007929	11.745006
86.61897	18.674883	10.77736	12.981709	6.9530897	6.544403
98.205086	11.676641	77.459465	38.095264	6.733493	6.8879776
98.205086	11.676641	77.459465	38.095264	6.733493	6.8879776
98.205086	11.676641	77.459465	38.095264	6.733493	6.8879776
76.901474	20.922037	27.521494	22.796799	6.658544	6.952891
49.35076	30.99529	32.855816	26.314634	6.800972	6.456201

9.310479	10.694319	9.713501	10.201367	8.946791	7.5214567
8.952066	7.415809	8.235066	7.9664865	5.824733	6.7025757
5.97195	5.018354	4.973589	4.9940286	2.3467326	2.4279919
5.97195	5.018354	4.973589	4.9940286	2.3467326	2.4279919
5.97195	5.018354	4.973589	4.9940286	2.3467326	2.4279919
4.883236	3.895083	6.139213	5.877446	2.3467326	2.4279919
10.326608	8.228167	7.620659	8.0078125	6.5615597	5.8750777
8.453865	8.419691	7.1489687	7.8004084	5.755705	6.415596
7.9453964	7.880502	8.79986	8.543084	6.760474	6.4802055
8.998642	8.578119	6.738639	7.8057694	6.0326552	4.807745
5.3671384	5.9501505	5.469121	5.5236344	4.209	4.327213
7.777924	7.179931	7.0678725	7.257452	6.159248	5.7483354
5.161788	3.9250717	5.0294785	4.1895256	3.8186889	2.4279919
5.923726	4.8355274	5.1878457	5.2033877	3.8345864	2.4279919
5.4627233	5.0429378	6.583504	6.2100544	3.4965007	3.8561304
4.4360514	4.7402105	5.531058	5.1304765	3.2195497	3.2878168
7.8861995	7.1702876	6.3435903	6.985943	4.2549696	4.96185
3.7347383	4.3821063	4.0334444	4.03057	2.3467326	3.85964
7.724357	8.082412	4.9619446	7.170001	6.0444593	5.973104
7.193693	8.253093	7.040218	7.6796465	6.5937204	6.616384
7.193693	8.253093	7.040218	7.6796465	6.5937204	6.616384
7.193693	8.253093	7.040218	7.6796465	6.5937204	6.616384
7.193693	8.253093	7.040218	7.6796465	6.5937204	6.616384
7.193693	8.253093	7.040218	7.6796465	6.5937204	6.616384
3.3767688	3.5917988	3.9879944	3.7941175	2.5668135	2.4279919
6.7101984	6.770028	6.504014	6.626141	4.4081416	4.14771
3.9788797	3.4476595	3.9466958	4.062228	2.6802049	2.4279919
6.6428337	6.39021	6.669389	6.836334	4.4747753	3.3952608
6.6428337	6.39021	6.669389	6.836334	4.4747753	3.3952608
7.8872366	8.935019	8.069753	8.651334	7.5744343	6.732023
4.344529	4.4576254	4.210272	4.3821063	3.4907894	2.4279919
8.296036	7.964705	7.533695	7.8771286	6.9842486	6.410164
8.296036	7.964705	7.533695	7.8771286	6.9842486	6.410164
8.296036	7.964705	7.533695	7.8771286	6.9842486	6.410164
8.296036	7.964705	7.533695	7.8771286	6.9842486	6.410164
6.937498	6.984489	6.704623	6.08428	5.5193677	4.908648
3.3664968	4.8418093	4.437298	4.3172793	2.8341522	3.927845
6.251731	5.5379395	6.367337	6.018183	2.3467326	3.288384
6.251731	5.5379395	6.367337	6.018183	2.3467326	3.288384
6.251731	5.5379395	6.367337	6.018183	2.3467326	3.288384
6.251731	5.5379395	6.367337	6.018183	2.3467326	3.288384
3.9564953	4.6923003	7.011234	6.1963964	4.2766814	5.0950284
6.971765	2.3644512	6.2304897	5.5739594	4.06421	3.849219
6.918961	6.4565935	5.6068864	6.069859	5.2160473	4.3790574
6.918961	6.4565935	5.6068864	6.069859	5.2160473	4.3790574
13.088534	13.602689	6.5985875	12.323554	4.920095	6.1737413
4.643329	4.3735476	4.7663503	4.9443884	4.0984774	2.4279919
8.088139	4.1300054	6.448945	5.6432543	4.7018857	5.5715437
8.813948	8.418445	9.400775	9.148048	7.3750467	7.7695537
8.813948	8.418445	9.400775	9.148048	7.3750467	7.7695537
8.813948	8.418445	9.400775	9.148048	7.3750467	7.7695537
8.37176	8.544586	6.6398687	7.7508507	2.3467326	2.4279919
12.063105	11.443338	11.627442	11.664671	10.293921	10.011373
7.100487	6.8625493	7.3732386	7.266332	5.864369	6.222662
7.100487	6.8625493	7.3732386	7.266332	5.864369	6.222662
12.286313	12.816938	11.730327	12.416035	9.637304	10.098842
6.531791	6.3592005	5.974218	5.917599	5.745844	3.4048026
6.833834	5.560849	6.7888656	6.375064	5.8151846	4.5102587
6.833834	5.560849	6.7888656	6.375064	5.8151846	4.5102587
6.833834	5.560849	6.7888656	6.375064	5.8151846	4.5102587
6.6102934	7.5460916	6.5149226	7.0797615	5.412638	4.4770713
7.28121	7.058901	6.1588383	6.656333	5.4633427	5.118687

9.390692	8.687897	8.566363	8.619994
5.56461	6.301192	4.5241785	5.0797906
2.6848872	3.2959056	3.6074893	3.4200444
2.6848872	3.2959056	3.6074893	3.4200444
2.6848872	3.2959056	3.6074893	3.4200444
2.9299414	2.652359	3.4542572	2.352752
6.930485	5.421158	5.416299	5.329326
5.467793	6.711897	5.967298	6.4220996
6.882299	6.949231	7.1316414	6.9353433
6.564968	5.9489	6.5411854	5.949353
4.6382585	4.5120177	5.0065136	4.5888133
6.0119452	5.8748646	6.4577093	5.964653
3.8449483	2.3408759	2.3618152	3.2428002
3.8189805	2.9792404	3.6762753	3.8564725
4.4221272	3.0190907	5.8468485	4.6854677
3.2438054	2.3408759	4.0554543	3.7300177
4.329341	5.289346	4.8786163	4.502949
2.7539785	2.3408759	2.3618152	2.352752
6.2505713	6.554103	5.1265464	5.7163506
6.638735	5.911976	6.645652	6.1047087
6.638735	5.911976	6.645652	6.1047087
6.638735	5.911976	6.645652	6.1047087
6.638735	5.911976	6.645652	6.1047087
6.638735	5.911976	6.645652	6.1047087
3.0434377	2.5392249	2.3618152	2.352752
4.8232274	4.122161	4.23848	4.0515842
4.0083365	3.0431654	2.3618152	3.4086986
5.4643283	2.7369564	5.2451262	4.542872
5.4643283	2.7369564	5.2451262	4.542872
8.117848	5.8115625	6.9846425	6.3879356
4.0796003	2.8988798	3.898635	2.352752
7.4381466	6.219662	6.654179	6.4789686
7.4381466	6.219662	6.654179	6.4789686
7.4381466	6.219662	6.654179	6.4789686
7.4381466	6.219662	6.654179	6.4789686
6.446036	3.4629354	5.732198	5.2370453
3.0642402	3.156166	2.3618152	3.2649329
2.350709	3.7244644	2.3618152	2.7204459
2.350709	3.7244644	2.3618152	2.7204459
2.350709	3.7244644	2.3618152	2.7204459
2.350709	3.7244644	2.3618152	2.7204459
3.3542109	2.5540571	4.9811063	4.989828
3.0081766	3.3601177	3.3612103	2.352752
5.469121	5.1198263	4.1960816	4.78428
5.469121	5.1198263	4.1960816	4.78428
4.395179	9.980859	4.8072286	8.889292
4.8980455	3.1027772	4.227571	2.6289368
3.1011732	3.147706	3.9763172	3.890456
7.1901817	7.50123	8.7229805	8.084542
7.1901817	7.50123	8.7229805	8.084542
7.1901817	7.50123	8.7229805	8.084542
3.1954978	2.9628856	2.3618152	2.352752
10.499889	10.258546	10.843684	10.564739
6.173225	5.5413747	5.9462333	5.6279664
6.173225	5.5413747	5.9462333	5.6279664
9.4709425	10.428811	10.7457485	10.506499
6.406916	4.3750353	3.7074575	4.0059657
6.5785966	3.6567564	6.537415	5.6587105
6.5785966	3.6567564	6.537415	5.6587105
6.5785966	3.6567564	6.537415	5.6587105
6.2327456	4.553449	5.0561547	4.857379
5.624324	5.1650705	5.307986	5.0811777

ASHGA5P036142	0.000786412	0.018693364	2.5048049	up	noncoding
ASHGA5P036142	0.000786412	0.018693364	2.5048049	up	noncoding
ASHGA5P032620	0.016584614	0.078580714	2.1473727	up	noncoding
ASHGA5P056404	0.002804693	0.034179323	2.0866832	up	noncoding
ASHGA5P030604	0.012811735	0.069277826	2.2713409	up	noncoding
ASHGA5P044146	0.014196993	0.07311388	2.335728	up	noncoding
ASHGA5P032923	0.001963999	0.029367232	2.1390394	up	noncoding
ASHGA5P029346	0.026262692	0.101017984	7.6021628	up	noncoding
ASHGA5P037313	0.036963475	0.122052801	2.9333153	up	noncoding
ASHGA5P031757	0.001774506	0.028194527	6.7935347	up	noncoding
ASHGA5P036109	0.001030376	0.02180291	2.8007194	up	noncoding
ASHGA5P043646	0.005388813	0.046686888	3.012069	up	noncoding
ASHGA5P043646	0.005388813	0.046686888	3.012069	up	noncoding
ASHGA5P040298	0.003969963	0.040458123	2.6552881	up	noncoding
ASHGA5P035267	0.007597837	0.05476971	2.6365736	up	noncoding
ASHGA5P035267	0.007597837	0.05476971	2.6365736	up	noncoding
ASHGA5P035267	0.007597837	0.05476971	2.6365736	up	noncoding
ASHGA5P026978	0.003467272	0.037964495	3.1932996	up	noncoding
ASHGA5P033505	0.001516222	0.026061852	4.4064652	up	noncoding
ASHGA5P018793	0.046099546	0.138314619	2.1886253	up	noncoding
ASHGA5P053143	0.006304386	0.050660451	2.5009764	up	noncoding
ASHGA5P053143	0.006304386	0.050660451	2.5009764	up	noncoding
ASHGA5P021149	0.005874032	0.048858059	4.4627149	up	noncoding
ASHGA5P019670	0.000157143	0.011056858	2.7525392	up	noncoding
ASHGA5P025704	0.000557999	0.016097331	3.8259934	up	noncoding
ASHGA5P023578	0.000458137	0.015075849	2.3702058	up	noncoding
ASHGA5P028042	0.019863847	0.086084959	3.3599994	up	noncoding
ASHGA5P056540	0.035522737	0.119277847	2.0404766	up	noncoding
ASHGA5P040794	0.000507233	0.015567285	2.0495668	up	noncoding
ASHGA5P043693	0.000165905	0.011182026	3.4625892	up	noncoding
ASHGA5P043362	0.006163889	0.050136632	2.4841032	up	noncoding
ASHGA5P023330	0.000111756	0.009604611	2.4379832	up	noncoding
ASHGA5P029329	0.006616549	0.051709907	2.1881848	up	noncoding
ASHGA5P029329	0.006616549	0.051709907	2.1881848	up	noncoding
ASHGA5P029329	0.006616549	0.051709907	2.1881848	up	noncoding
ASHGA5P044814	0.001122395	0.022616453	6.1266661	up	noncoding
ASHGA5P053398	0.001926607	0.029259988	3.1829509	up	noncoding
ASHGA5P047196	0.00890742	0.058871793	2.0982918	up	noncoding
ASHGA5P047196	0.00890742	0.058871793	2.0982918	up	noncoding
ASHGA5P018545	0.006904616	0.052741391	2.5618194	up	noncoding
ASHGA5P031462	0.001562147	0.026561102	3.4046659	up	noncoding
ASHGA5P031462	0.001562147	0.026561102	3.4046659	up	noncoding
ASHGA5P040967	0.000189334	0.01146874	2.693979	up	noncoding
ASHGA5P019108	0.005808126	0.048450721	2.7847072	up	noncoding
ASHGA5P020260	0.002332754	0.031698104	2.1157001	up	noncoding
ASHGA5P046527	0.023234983	0.09438496	2.2865541	up	noncoding
ASHGA5P037005	0.003423315	0.037690616	3.0008076	up	noncoding
ASHGA5P037005	0.003423315	0.037690616	3.0008076	up	noncoding
ASHGA5P037005	0.003423315	0.037690616	3.0008076	up	noncoding
ASHGA5P027695	0.011506333	0.066101265	5.3609735	up	noncoding
ASHGA5P020787	0.035734463	0.119707935	2.1740432	up	noncoding
ASHGA5P027536	0.000534127	0.015930561	3.3814083	up	noncoding
ASHGA5P021701	0.00059207	0.016411961	3.2487973	up	noncoding
ASHGA5P040749	0.000510138	0.015567285	2.221559	up	noncoding
ASHGA5P029721	0.027172251	0.102915644	2.0544306	up	noncoding
ASHGA5P031092	0.002033718	0.029655965	2.2325178	up	noncoding
ASHGA5P031092	0.002033718	0.029655965	2.2325178	up	noncoding
ASHGA5P050981	0.002923617	0.034973658	2.4816182	up	noncoding
ASHGA5P028925	0.007332916	0.053840332	72.9688452	up	noncoding
ASHGA5P044953	0.01784005	0.081069421	2.6314169	up	noncoding
ASHGA5P027994	0.025876485	0.100335141	2.2658034	up	noncoding
ASHGA5P027994	0.025876485	0.100335141	2.2658034	up	noncoding

NR_033903	CCDC74B-AS1	RefSeq	1835	chr2	+
NR_033903	CCDC74B-AS1	RefSeq	1835	chr2	+
TCONS_00016645	XLOC_007451	LincRNAs identified	469	chr9	+
TCONS_00003955	XLOC_001773	LincRNAs identified	444	chr2	+
uc002aqh.1	BX538221	UCSC_knowngene	4347	chr15	+
ENST00000520920	RP11-1080G15.1	GENCODE	561	chr8	-
ENST00000420951	AC025627.7	GENCODE	500	chr17	+
ENST00000555860	LINC00524	GENCODE	281	chr14	-
ENST00000415824	AF064858.6	GENCODE	407	chr21	-
ENST00000563424	RP11-401P9.4	GENCODE	3441	chr16	+
ENST00000445332	AC009404.2	GENCODE	424	chr2	+
uc010kyk.2	BC008049	UCSC_knowngene	1942	chr7	+
uc010kyk.2	BC008049	UCSC_knowngene	1942	chr7	+
uc003jcv.3	LOC728613	UCSC_knowngene	1205	chr5	-
ENST00000454260	STEAP3-AS1	GENCODE	3218	chr2	-
ENST00000454260	STEAP3-AS1	GENCODE	3218	chr2	-
ENST00000454260	STEAP3-AS1	GENCODE	3218	chr2	-
uc001qls.1	BC047090	UCSC_knowngene	699	chr12	-
ENST00000580197	RP11-146G7.3	GENCODE	2343	chr18	-
ENST00000457694	AC098820.2	GENCODE	666	chr2	-
NR_029411	LOC100133091	RefSeq	2712	chr7	+
NR_029411	LOC100133091	RefSeq	2712	chr7	+
ENST00000531402	CTC-497E21.4	GENCODE	590	chr11	-
uc001vmo.4	BC035106	UCSC_knowngene	2905	chr13	-
NR_027241	LOC388796	RefSeq	1186	chr20	-
ENST00000585030	SNHG15	GENCODE	953	chr7	-
ENST00000547177	RP11-968A15.2	GENCODE	735	chr12	+
TCONS_00005994	XLOC_002619	LincRNAs identified	414	chr3	+
ENST00000419105	RP11-277L2.2	GENCODE	777	chr1	+
uc003tsz.3	AK123141	UCSC_knowngene	3644	chr7	+
NR_024454	NDUFB2-AS1	RefSeq	1648	chr7	-
ENST00000578497	RP11-146G7.3	GENCODE	545	chr18	-
ENST00000569214	RP11-872J21.3	GENCODE	1512	chr14	-
ENST00000569214	RP11-872J21.3	GENCODE	1512	chr14	-
ENST00000569214	RP11-872J21.3	GENCODE	1512	chr14	-
ENST00000521188	RP11-546K22.1	GENCODE	672	chr8	+
ENST00000436285	GAS5	GENCODE	772	chr1	-
NR_027709	IDI2-AS1	RefSeq	1324	chr10	+
NR_027709	IDI2-AS1	RefSeq	1324	chr10	+
ENST00000454833	TMEM191A	GENCODE	419	chr22	+
ENST00000568795	RP11-304L19.4	GENCODE	456	chr16	+
ENST00000568795	RP11-304L19.4	GENCODE	456	chr16	+
TCONS_00022268	XLOC_010588	LincRNAs identified	442	chr13	-
ENST00000468527	KRT8P12	GENCODE	998	chr3	+
ENST00000510565	FAM86EP	GENCODE	741	chr4	-
ENST00000421132	RP11-351M16.3	GENCODE	273	chr10	-
ENST00000421894	HM13-IT1	GENCODE	356	chr20	+
ENST00000421894	HM13-IT1	GENCODE	356	chr20	+
ENST00000421894	HM13-IT1	GENCODE	356	chr20	+
ENST00000561864	RP11-474D1.4	GENCODE	577	chr12	-
ENST00000519819	CTD-3046C4.1	GENCODE	432	chr8	-
ENST00000550029	RP11-341G23.4	GENCODE	738	chr12	-
uc010zaz.1	LOC150776	UCSC_knowngene	1304	chr2	+
uc010pbe.1	BC062745	UCSC_knowngene	1072	chr1	+
ENST00000478994	SERPINA13	pseudogene	924	chr14	+
ENST00000563777	FBXL19-AS1	GENCODE	3951	chr16	-
ENST00000563777	FBXL19-AS1	GENCODE	3951	chr16	-
ENST00000423536	RP4-564F22.2	GENCODE	517	chr20	-
NR_034119	LINC00460	RefSeq	935	chr13	+
ENST00000499653	KB-431C1.4	GENCODE	2048	chr8	+
NR_047672	SLC4A8	RefSeq	520	chr12	+
NR_047672	SLC4A8	RefSeq	520	chr12	+

130887195	130896986		GATACCTGCTTCA	285103	natural antisense
130887195	130896986		GATACCTGCTTCA	285103	natural antisense
92763073	92767272		AAAGATCAAACATCGTCCCAAATCT		intergenic
181556788	181589386		AGTCCTCAGGTGCCATCAATATTCT		intergenic
67152640	67156987		AGGCAGACATGAGTCAACATATAAC		intergenic
18942501	19116979		GAATTACCAGGAAGATTTTGTTTGT		intergenic
19483255	19499371		TCTTGCGCAGTTGATGATCTTTATA		intergenic
101872329	101903211	ENST00000556987	GGACAATGTCTGAATTAAATGCCGT		intergenic
40252230	40328392		AAGAGAGCTTCCTAATGAGGCATCC		intergenic
50679719	50683160		TTTAAAGATGGTGGCAGTGAAACTG		intergenic
118595319	118596965	ENST00000420330	CTAACACAGAAGCCATAGTGGACTT		intergenic
44808453	44810735		TTTCTTGTGAATGTGCCAAGTATTAC		exon sense-overlappi
44808453	44810735		TTTCTTGTGAATGTGCCAAGTATTAC		exon sense-overlappi
1628800	1634120	uc003jct.3, uc003jcu	GAAAAAGACTGT	728613	intergenic
120001997	120006647		ATTTTGCATCTTGGAGCAGTTCTTCT		natural antisense
120001997	120006647		ATTTTGCATCTTGGAGCAGTTCTTCT		natural antisense
120001997	120006647		ATTTTGCATCTTGGAGCAGTTCTTCT		natural antisense
3445188	3445980		GGCAAGAGTTTTTGAAGAGTCTTGG		intergenic
6925476	6928571		TCACAGGAGACATTTTTGATGTCCC		intergenic
217250010	217277419		AAGAACCCAGCTCTATGAGGTTTGA		natural antisense
76178657	76257299		TTTTAGACGGTTA	100133091	natural antisense
76178657	76257299		TTTTAGACGGTTA	100133091	natural antisense
13002175	13011093	ENST00000526388	ATGGGTGTAGGGAGAAAGTTTAAGC		intergenic
96325091	96329179		GTAATGTGAAGAATAAAGGAGTCAC		bidirectional
37049238	37064018		CGTGGGTAAAGT	388796	intergenic
45022624	45026268	ENST00000580458,	TCAGAAGTGATT	285958	intergenic
54656398	54672847		ACTGGGCACATGGATTACATAAAAC		intronic antisense
39418963	39422626		AAGATTGCTAAAGCTGTCCCTGCCA		intergenic
149591178	149592306		CTTATGAATACTGAATAGTGAACAT		intergenic
63813368	63817012		AGAATTAATATTACCCAACTTGTCCA		intergenic
140395135	140396877		GTCAGCCACTTTC	100134713	natural antisense
6927304	6929961		TGGAGCAGTGGAGGTGTTAACCACC		intergenic
96967146	96968658		CTTTTTTCACAGAATGTCTAGTAGGA		bidirectional
96967146	96968658		CTTTTTTCACAGAATGTCTAGTAGGA		bidirectional
96967146	96968658		CTTTTTTCACAGAATGTCTAGTAGGA		bidirectional
52874017	52935534		GGAGGAAACTGACACACTTTTCTTA		intergenic
173833038	173836181	ENST00000412059,	GTAGGGCAAATT	60674	intergenic
1068576	1090141	ENST00000434470	TTGGAGTCTGTGA	55853	natural antisense
1068576	1090141	ENST00000434470	TTGGAGTCTGTGA	55853	natural antisense
21057262	21058407	ENST00000414022,	GACGCTGCAGGA	84222	intergenic
2162335	2163343		AGACCACGTTCTGGAAGGTCAGGGA		natural antisense
2162335	2163343		AGACCACGTTCTGGAAGGTCAGGGA		natural antisense
44977061	44978565		AGCCTATTTGTGCCTTGTCACCAGA		intergenic
160283234	160285987		GACAGGAAAGTAAGCTCTCTGAAGT		natural antisense
3944637	3957135		TCACTCACAGGCCAGGCCAGGATCA		intergenic
28721936	28784742		TGGTTGCTGCAAGCGTGTGATACCC		intergenic
30150968	30151879		TCCAACACACAGGTAGTCCCAGTGC		intron sense-overlapp
30150968	30151879		TCCAACACACAGGTAGTCCCAGTGC		intron sense-overlapp
30150968	30151879		TCCAACACACAGGTAGTCCCAGTGC		intron sense-overlapp
130509037	130529501		CTGGCTCCGAGTGTCCGTATCGCGT		intergenic
64298166	64374108		CTTCCTTTTGCTCTATCCCCATTTCC		intergenic
104157089	104162636		GCCTCTGGGAGCTTTTCCTTTTTTAA		natural antisense
132250385	132273977		CACTGGGTAGTG	150776	bidirectional
149230715	149232553		TTGTAAGAATGGCATAAAGCCGGTT		intergenic
95107654	95111363	ENST00000469935	GAACTCGATTTCC	388007	
30930639	30934590		TCCAGCTTCTCTT	283932	natural antisense
30930639	30934590		TCCAGCTTCTCTT	283932	natural antisense
37049234	37059709	ENST00000417578,	TTTGAATTTGTAGCTGTCGTTCACTT		intergenic
107028910	107030142		CGCCTCTGAAATC	728192	intergenic
103255836	103265561		CTTCCAGTTCTTTAAGTCAAAATTTT		natural antisense
51818593	51834963		GTACTCAGTTCAC	9498	exon sense-overlappi
51818593	51834963		GTACTCAGTTCAC	9498	exon sense-overlappi

NM_001258307	CCDC74B	coiled-coil domain-c	-	130896855	130902707
NM_207310	CCDC74B	coiled-coil domain-c	-	130896855	130902707
NM_031449	ZMIZ2	zinc finger MIZ dom	+	44788529	44809479
NM_174929	ZMIZ2	zinc finger MIZ dom	+	44795786	44809479
NM_001008410	STEAP3	metalloreductase STI	+	119981383	120023227
NM_018234	STEAP3	metalloreductase STI	+	119981383	120023227
NM_182915	STEAP3	metalloreductase STI	+	119981383	120023227
NM_014140	SMARCAL1	SWI/SNF-related ma	+	217277136	217347774
NM_012230	POMZP3	POM121 and ZP3 fu	-	76239302	76256620
NM_152992	POMZP3	POM121 and ZP3 fu	-	76239302	76256620
NM_006260	DNAJC3	dnaJ homolog subfar	+	96329392	96447243
NM_012117	CBX5	chromobox protein h	-	54624730	54673915
NM_004546	NDUFB2	NADH dehydrogena	+	140396480	140406446
NM_001252006	PAPOLA	poly(A) polymerase a	+	96968712	97001935
NM_001252007	PAPOLA	poly(A) polymerase a	+	96968712	97001935
NM_032632	PAPOLA	poly(A) polymerase a	+	96968712	97033453
NM_004508	IDI1	isopentenyl-diphosph	-	1085963	1095061
NM_033261	IDI2	isopentenyl-diphosph	-	1064846	1071799
NM_000296	PKD1	polycystin-1 isoform	-	2138710	2185899
NM_001009944	PKD1	polycystin-1 isoform	-	2138710	2185899
NM_002268	KPNA4	importin subunit alph	-	160212782	160283376
NM_030789	HM13	minor histocompatibi	+	30102240	30157370
NM_178580	HM13	minor histocompatibi	+	30102240	30157370
NM_178581	HM13	minor histocompatibi	+	30102240	30157370
NM_017564	STAB2	stabilin-2 precursor	+	103981068	104160502
NM_001085365	MZT2A	mitotic-spindle orgar	-	132241532	132250064
ENST00000471231	FBXL19	F-box and leucine-ric	+	30934391	30960097
ENST00000562798	FBXL19	F-box and leucine-ric	+	30934375	30937252
NM_015902	UBR5	E3 ubiquitin-protein	-	103264501	103424917
NM_001039960	SLC4A8	electroneutral sodiun	+	51818593	51909547
NM_001258401	SLC4A8	electroneutral sodiun	+	51785100	51909547

57.989404	14.863491	5.508481	4.183782	65.820946	54.05838
57.989404	14.863491	5.508481	4.183782	65.820946	54.05838
314.285922	95.589945	7.904729	6.802156	351.23972	422.22678
50.590083	16.32663	5.306559	4.245348	51.500088	40.24259
70.8302	22.376488	5.760941	4.577397	94.020134	95.678635
31.368197	9.247581	4.598277	3.374405	46.193035	28.39325
52.691784	16.664524	5.358005	4.261042	65.72899	61.366188
599.282789	27.092385	7.373833	4.447423	57.71704	17.728333
50.5066	7.659808	4.50353	2.950998	90.191605	15.236608
3285.366483	245.742572	11.048088	8.283926	3254.004	997.7515
169.794437	40.74752	6.990928	5.50513	133.02089	146.54112
2795.121717	555.386225	10.990049	9.399294	3038.9153	2319.8462
2795.121717	555.386225	10.990049	9.399294	3038.9153	2319.8462
10384.67718	1899.417717	12.660806	11.251937	6271.88	6743.0244
243.093103	61.26616	7.527753	6.129089	207.6955	327.29187
243.093103	61.26616	7.527753	6.129089	207.6955	327.29187
243.093103	61.26616	7.527753	6.129089	207.6955	327.29187
62.928832	9.6118	4.877254	3.202206	13.491349	7.790182
226.776595	46.023772	7.109833	4.970211	199.8087	60.81705
29.657929	11.863385	4.563226	3.433201	35.820972	29.354277
103.757485	26.842168	6.306319	4.983827	105.18971	134.63791
103.757485	26.842168	6.306319	4.983827	105.18971	134.63791
51.230203	8.865263	5.234014	3.076093	29.49046	37.970978
523.18953	116.237167	8.551911	7.091148	607.759	260.12372
1386.573383	214.211422	10.000242	8.064407	1620.9452	1926.9719
118.857297	30.824582	6.509982	5.264969	142.45149	159.43773
81.304772	18.554808	5.868549	4.120088	59.089115	71.07308
447.043967	119.278872	8.184731	7.155825	426.1879	891.98444
194.932087	63.213047	7.167366	6.132047	255.75516	160.75471
3092.8916	519.248198	11.134102	9.342251	2997.899	2237.0967
765.133808	162.52787	8.981063	7.668338	514.1136	613.4526
62.970882	16.672326	5.552534	4.266846	82.35568	69.83546
150.012257	45.008084	6.809537	5.679802	192.18347	136.9185
150.012257	45.008084	6.809537	5.679802	192.18347	136.9185
150.012257	45.008084	6.809537	5.679802	192.18347	136.9185
265.285568	26.456335	7.552392	4.93729	180.08743	203.736
12422.196	2244.188517	13.142706	11.472341	12344.187	9786.553
42.012726	13.64383	5.041304	3.972089	57.50682	46.121777
42.012726	13.64383	5.041304	3.972089	57.50682	46.121777
107.751445	29.293506	6.385327	5.028158	100.11551	93.175865
57.001915	11.525421	5.417134	3.649621	81.37161	82.50495
57.001915	11.525421	5.417134	3.649621	81.37161	82.50495
135.401585	35.01134	6.707662	5.277923	131.37228	106.54268
407.018333	86.622194	8.223281	6.745756	336.0516	310.8482
309.561708	90.567832	7.828542	6.747407	315.38162	413.323
67.135822	16.407833	5.507917	4.314742	93.35407	34.773445
233.522828	45.989497	7.41185	5.826499	273.19788	185.29216
233.522828	45.989497	7.41185	5.826499	273.19788	185.29216
233.522828	45.989497	7.41185	5.826499	273.19788	185.29216
243.389337	33.499198	7.14771	4.725215	334.23364	588.7352
32.477501	10.205469	4.62564	3.505259	40.865456	26.72454
908.646035	149.187834	9.280838	7.523214	769.27094	842.2036
88.229619	18.160414	6.043353	4.343447	100.0344	138.29729
192.486782	52.285504	7.123407	5.971834	200.41812	103.91041
20.183392	7.314897	3.994987	2.956249	19.401121	16.933039
1513.800367	386.947105	10.096119	8.937448	1391.9089	1880.6642
1513.800367	386.947105	10.096119	8.937448	1391.9089	1880.6642
45523.52283	10551.7165	15.016679	13.705397	49813.395	63730.812
2356.882562	6.245971	8.788776	2.599568	482.55923	40.08796
3436.054	659.671308	11.12452	9.72868	3177.5508	1244.902
107.86383	35.50309	6.14855	4.968527	128.64229	24.02607
107.86383	35.50309	6.14855	4.968527	128.64229	24.02607

76.619225	49.953346	47.51627	53.96826	15.894078	18.36288
76.619225	49.953346	47.51627	53.96826	15.894078	18.36288
272.69772	252.62373	322.1076	264.81998	90.895134	50.999237
53.31915	39.77209	55.488117	63.218464	15.998241	7.7077003
59.84246	51.724068	62.326626	61.389275	26.459085	8.788421
37.79662	35.8843	18.201399	21.74058	4.9999995	10.020928
49.01145	37.75328	51.88299	50.407806	14.973848	10.02388
80.51826	436.8578	1577.9852	1424.8901	8.965571	13.071131
157.9795	16.868298	13.409859	9.35373	8.343867	4.9999995
5445.366	5072.2954	1607.6958	3335.0862	224.76967	183.21564
100.37258	231.29358	155.10527	252.43318	24.043777	26.358307
3751.3638	1634.736	2860.281	3165.588	381.81293	257.2237
3751.3638	1634.736	2860.281	3165.588	381.81293	257.2237
5472.7627	22687.594	5025.836	16106.966	1729.7086	1010.8731
157.66658	232.99364	261.2051	271.70593	66.09404	26.344954
157.66658	232.99364	261.2051	271.70593	66.09404	26.344954
157.66658	232.99364	261.2051	271.70593	66.09404	26.344954
19.083008	165.11258	53.10786	118.98801	4.9999995	4.9999995
382.73856	61.4494	350.9039	304.94196	29.613138	4.9999995
23.738605	26.904932	31.113564	31.015223	9.729845	4.9999995
81.37949	134.1394	61.97413	105.22427	38.30813	20.475481
81.37949	134.1394	61.97413	105.22427	38.30813	20.475481
37.07725	89.51384	36.935383	76.39331	9.274922	4.9999995
910.2368	441.1075	405.48926	514.4209	127.574936	55.978924
1135.5974	1109.404	1136.1403	1390.3815	173.89629	182.87279
122.866394	102.303696	80.28244	105.80203	29.038994	30.276709
47.77191	50.476845	126.63198	132.7857	25.50075	4.9999995
255.39691	129.29218	550.48627	428.9161	127.5641	76.43286
299.8335	103.628685	165.05005	184.57042	98.57337	42.420216
3745.5928	3801.4397	2066.339	3708.9824	483.48486	286.46237
399.23758	1578.845	366.43637	1118.7177	140.0548	98.14755
78.13733	28.989214	61.305878	57.201733	22.582611	14.287958
232.64981	95.527214	110.23384	132.56071	38.557636	23.55926
232.64981	95.527214	110.23384	132.56071	38.557636	23.55926
232.64981	95.527214	110.23384	132.56071	38.557636	23.55926
153.20067	467.18906	183.12961	404.37064	30.565104	27.9801
11784.882	16959.373	8316.677	15341.504	1625.1047	1404.5197
35.11544	44.270584	39.027573	30.034164	14.308558	8.463162
35.11544	44.270584	39.027573	30.034164	14.308558	8.463162
115.740326	144.42572	77.67251	115.37874	23.560202	10.679379
54.891636	34.733414	44.567142	43.94274	12.35747	6.9413066
54.891636	34.733414	44.567142	43.94274	12.35747	6.9413066
160.01761	102.37767	145.17715	166.92212	32.57608	12.921641
349.24677	681.7682	256.162	508.03323	86.79244	74.771904
145.61818	363.71515	254.99078	364.34152	62.496582	78.90302
135.96185	70.173035	21.239298	47.313232	13.918319	12.703568
331.78998	324.6774	105.66322	180.51633	53.697933	30.42245
331.78998	324.6774	105.66322	180.51633	53.697933	30.42245
331.78998	324.6774	105.66322	180.51633	53.697933	30.42245
79.807526	62.148937	213.23346	182.17726	47.86345	93.54285
38.0659	50.298897	15.504508	23.405708	10.05062	7.2660246
651.98663	1667.174	346.57944	1174.6616	128.3363	80.669716
75.75401	49.419807	78.89788	86.97433	21.349552	14.13898
281.2068	101.49039	225.67546	242.21951	53.98885	34.316433
18.166979	30.447226	12.837561	23.314425	7.071251	4.9999995
771.2491	1361.9851	1722.7456	1954.2493	370.93552	265.6616
771.2491	1361.9851	1722.7456	1954.2493	370.93552	265.6616
32739.246	32173.625	45794.426	48889.633	9585.252	11876.891
965.54407	50.01241	6943.351	5659.7407	4.9999995	4.9999995
4634.9897	5872.0034	1435.8317	4251.0464	546.636	483.2564
223.77031	67.2321	100.61742	102.89479	51.346947	10.46524
223.77031	67.2321	100.61742	102.89479	51.346947	10.46524

15.101159	15.525481	12.463801	11.8335495	5.580175	5.6142583
15.101159	15.525481	12.463801	11.8335495	5.580175	5.6142583
163.89282	127.06508	52.95635	87.73105	8.001393	8.412938
21.684946	22.808752	15.540362	14.219781	5.220228	5.223381
46.52266	14.597543	21.92869	15.962531	6.1078196	6.3650327
12.720709	11.63459	10.802989	5.306273	5.0707	4.7696514
29.305582	14.62325	18.673283	12.3873	5.5773425	5.7771635
4.9999995	83.736046	14.095279	37.686283	5.3893533	4.147052
13.329058	4.9999995	4.9999995	9.285926	6.0416627	3.949644
268.3809	217.14818	344.56873	236.37231	11.193956	9.617431
31.534204	88.61749	21.782988	52.148354	6.607952	6.962137
504.33652	789.1381	773.4592	626.3469	11.089832	10.757927
504.33652	789.1381	773.4592	626.3469	11.089832	10.757927
2496.69	2349.769	2027.7277	1781.7379	12.127857	12.252377
115.991196	56.36133	59.679546	43.125893	7.245403	8.07393
115.991196	56.36133	59.679546	43.125893	7.245403	8.07393
115.991196	56.36133	59.679546	43.125893	7.245403	8.07393
4.9999995	19.45441	11.10472	12.1116705	3.2738295	3.0900133
49.367126	8.825499	131.39607	51.9408	7.1903667	5.765414
32.288235	9.530474	7.7434525	6.8883057	4.7000003	4.8119617
35.628155	16.94805	29.319098	20.374092	6.2712216	6.840281
35.628155	16.94805	29.319098	20.374092	6.2712216	6.840281
20.502142	5.7826123	4.9999995	7.631904	4.4259925	5.149482
195.22267	82.9058	140.74403	94.99664	8.774443	7.7588778
134.66158	236.49274	317.59927	239.74586	10.196391	10.496512
29.134766	39.642517	28.805555	28.048952	6.7039247	7.075101
34.1961	10.47798	23.80802	12.346	5.424624	5.9727454
186.89676	85.49971	154.49779	84.78201	8.275676	9.463863
117.20668	38.458324	49.2947	33.324993	7.5417705	7.0844865
682.9901	477.5672	724.71136	460.2733	11.06766	10.708962
186.33165	188.74057	202.3786	159.51405	8.522138	8.945379
19.002295	9.196097	23.759195	11.205799	5.911231	5.9490223
63.040897	27.74283	78.85307	38.29481	7.1355205	6.865786
63.040897	27.74283	78.85307	38.29481	7.1355205	6.865786
63.040897	27.74283	78.85307	38.29481	7.1355205	6.865786
39.85992	30.759157	12.833931	16.7398	7.036104	7.4182854
1671.536	2602.5115	3615.2495	2546.2097	13.134815	12.797764
23.408772	13.668818	8.313623	13.700049	5.3830338	5.403407
23.408772	13.668818	8.313623	13.700049	5.3830338	5.403407
40.86925	21.75346	47.861855	31.03689	6.1948295	6.332045
24.48424	9.947154	6.0626626	9.359693	5.894414	6.171751
24.48424	9.947154	6.0626626	9.359693	5.894414	6.171751
59.01153	24.012169	50.243202	31.30342	6.5900054	6.5178013
88.10422	62.391922	123.09699	84.57569	7.938771	8.005108
40.191082	121.6335	128.4872	111.69561	7.8464494	8.385143
18.44019	21.996626	13.673229	17.715067	6.0988364	5.036575
54.95688	38.043686	48.290215	50.525818	7.635591	7.2920423
54.95688	38.043686	48.290215	50.525818	7.635591	7.2920423
54.95688	38.043686	48.290215	50.525818	7.635591	7.2920423
8.927493	27.070665	4.9999995	18.590733	7.9308023	8.891282
18.65725	4.9999995	9.003551	11.25537	4.8902235	4.687924
170.60808	212.85687	146.96355	155.69249	9.128752	9.383112
34.354576	11.2319	17.728638	10.15884	6.1939445	6.8808627
78.36412	35.159435	63.65165	48.232533	7.194901	6.485219
10.713859	4.9999995	10.4301195	5.674152	3.8186889	4.0894413
542.10803	333.09378	480.18607	329.69763	9.972914	10.463493
542.10803	333.09378	480.18607	329.69763	9.972914	10.463493
5437.151	10723.617	14990.009	10697.379	15.194954	15.56402
4.9999995	12.47583	4.9999995	4.9999995	8.435698	5.2184324
585.80194	850.67084	795.4123	696.25037	11.157704	9.921005
93.16481	31.1859	10.79869	16.05695	6.557001	4.543225
93.16481	31.1859	10.79869	16.05695	6.557001	4.543225

5.9440107	5.2490444	5.4014916	5.2619033	4.221769	5.0212007
5.9440107	5.2490444	5.4014916	5.2619033	4.221769	5.0212007
7.756576	7.6724052	8.0817795	7.5032816	6.733922	6.520251
5.4203696	4.891742	5.6089435	5.4746904	4.230795	3.7075639
5.591584	5.297826	5.768214	5.435169	4.9664836	3.9111662
4.926651	4.730274	4.093437	3.9989493	2.3467326	4.107153
5.299771	4.810434	5.517968	5.165352	4.1370273	4.107901
6.016551	8.461049	10.344941	9.884054	3.3796728	4.5074472
6.9679394	3.5258389	3.678391	2.8577027	3.26634	2.4279919
12.051443	11.971611	10.368048	11.086041	8.063625	8.3694105
6.3292575	7.5502944	7.0601554	7.43577	4.830284	5.555982
11.509357	10.369906	11.194374	11.018895	8.837509	8.8645
11.509357	10.369906	11.194374	11.018895	8.837509	8.8645
12.059278	14.167015	11.993926	13.36438	11.0612545	10.864643
6.965077	7.5596147	7.7857533	7.5367417	6.28343	5.554864
6.965077	7.5596147	7.7857533	7.5367417	6.28343	5.554864
6.965077	7.5596147	7.7857533	7.5367417	6.28343	5.554864
3.9343784	7.04215	5.5478826	6.375268	2.3467326	2.4279919
8.229831	5.565685	8.199354	7.708346	5.1190248	2.4279919
4.260743	4.286852	4.8187933	4.501006	3.505611	2.4279919
6.0297656	6.7384973	5.7611837	6.1969643	5.493387	5.1877437
6.0297656	6.7384973	5.7611837	6.1969643	5.493387	5.1877437
4.8971243	6.1319814	5.0536613	5.745844	3.426033	2.4279919
9.463334	8.474819	8.406461	8.433533	7.228176	6.659801
9.774508	9.791647	9.888836	9.853557	7.6924233	8.366545
6.619802	6.328231	6.1261964	6.2066355	5.089304	5.759997
5.260334	5.2640877	6.7629538	6.526549	4.9178734	2.4279919
7.6624255	6.6858573	8.847273	8.173292	7.2277017	7.105103
7.8918023	6.3452587	7.13914	7.0017366	6.856805	6.253792
11.508128	11.550407	10.727373	11.242085	9.185387	9.027562
8.29204	10.320081	8.2645235	9.542218	7.357365	7.4721193
5.9716144	4.3980055	5.747101	5.3382306	4.73555	4.6488047
7.538528	6.2262073	6.567598	6.5235806	5.502432	5.3880587
7.538528	6.2262073	6.567598	6.5235806	5.502432	5.3880587
7.538528	6.2262073	6.567598	6.5235806	5.502432	5.3880587
6.9284525	8.553777	7.28867	8.089065	5.16627	5.6443367
13.200209	13.739205	12.706604	13.2776375	10.966507	11.331601
4.817517	5.0583897	5.1287136	4.4567623	4.0669613	3.8510356
4.817517	5.0583897	5.1287136	4.4567623	4.0669613	3.8510356
6.5357842	6.8468814	6.0746307	6.327792	4.7990713	4.2057467
5.4633427	4.6809225	5.317424	4.9749517	3.856246	3.5589366
5.4633427	4.6809225	5.317424	4.9749517	3.856246	3.5589366
6.9878607	6.3289576	6.961946	6.8594003	5.256826	4.4887533
8.10952	9.109448	7.761488	8.415353	6.668409	7.068444
6.8613634	8.182675	7.7539873	7.9416356	6.200468	7.151573
6.7627482	5.7663975	4.3055325	5.077414	4.024627	4.464144
8.042403	8.023324	6.5080023	6.9697366	5.984077	5.765588
8.042403	8.023324	6.5080023	6.9697366	5.984077	5.765588
8.042403	8.023324	6.5080023	6.9697366	5.984077	5.765588
6.003419	5.5830975	7.4952927	6.982366	5.8129916	7.399451
4.9364414	5.259838	3.880103	4.099309	3.5536509	3.6230757
8.984114	10.397721	8.178058	9.613273	7.2356753	7.185997
5.9262476	5.236319	6.0984163	5.924327	4.654578	4.6320844
7.7982454	6.3167596	7.56967	7.3756456	5.992336	5.940664
3.8642788	4.478991	3.6219935	4.0965295	3.0157905	2.4279919
9.242205	10.090847	10.4563	10.350957	8.800274	8.916411
9.242205	10.090847	10.4563	10.350957	8.800274	8.916411
14.7137985	14.66977	15.043529	14.9140005	13.498958	14.423648
9.547023	5.2512894	12.4406595	11.839555	2.3467326	2.4279919
11.824774	12.181334	10.209119	11.453185	9.370539	9.783263
7.4838247	5.702115	6.439118	6.1660147	5.918398	4.1750994
7.4838247	5.702115	6.439118	6.1660147	5.918398	4.1750994

3.9811304	4.0984774	3.9156823	3.864434
3.9811304	4.0984774	3.9156823	3.864434
7.3039308	7.3569174	5.9956584	6.902258
4.4734273	4.6874986	4.229629	4.1431713
5.538986	4.002629	4.7282124	4.3169026
3.7500198	3.6531522	3.7109392	2.6784322
4.8932486	4.0054483	4.4908953	3.9317322
2.350709	6.7147865	4.088671	5.6432543
3.8107257	2.3408759	2.3618152	3.4982371
8.015125	8.17238	8.663923	8.419092
4.9934807	6.8007755	4.718278	6.1319814
8.931026	10.092948	9.827123	9.842656
8.931026	10.092948	9.827123	9.842656
11.243219	11.697415	11.247831	11.39726
6.8189287	6.1015563	6.1653085	5.8504467
6.8189287	6.1015563	6.1653085	5.8504467
6.8189287	6.1015563	6.1653085	5.8504467
2.350709	4.4372454	3.7498775	3.900678
5.625095	3.2401462	7.281335	6.127673
5.025608	3.3489451	3.2481415	3.0429091
5.1579037	4.2245765	5.152258	4.687096
5.1579037	4.2245765	5.152258	4.687096
4.3987904	2.643308	2.3618152	3.1986172
7.560084	6.69963	7.372795	7.026403
7.028619	8.305137	8.554007	8.4397135
4.8851585	5.5455427	5.1255145	5.1843
5.1085196	3.4940498	4.8440294	3.9280639
7.497245	6.7483788	7.49937	6.8571515
6.8335066	5.5001483	5.8952074	5.4528213
9.3618145	9.343954	9.730056	9.404734
7.4929805	7.9559665	7.912108	7.8194895
4.2978034	3.295794	4.8423557	3.780768
5.962536	4.996209	6.5615597	5.6680174
5.962536	4.996209	6.5615597	5.6680174
5.962536	4.996209	6.5615597	5.6680174
5.3143153	5.1523995	3.957766	4.388653
10.646077	11.852957	12.108326	11.928577
4.5791698	3.896771	3.3489451	4.0896487
4.5791698	3.896771	3.3489451	4.0896487
5.3506637	4.610817	5.8547955	5.347856
4.638044	3.4145653	2.9190226	3.510912
4.638044	3.4145653	2.9190226	3.510912
5.8695827	4.7677107	5.9228053	5.3618617
6.428979	6.2618966	7.1929398	6.8538656
5.326988	7.2850866	7.2487216	7.2716055
4.257909	4.6271977	4.043961	4.4706144
5.771663	5.4840956	5.8666234	6.0869474
5.771663	5.4840956	5.8666234	6.0869474
5.771663	5.4840956	5.8666234	6.0869474
3.2784464	4.956358	2.3618152	4.5422273
4.2712398	2.3408759	3.4557133	3.7869997
7.366003	8.1410055	7.4270854	7.7835183
5.1147532	3.6003551	4.421072	3.63784
6.2574377	5.3650203	6.258095	6.0174527
3.5216076	2.3408759	3.6647577	2.766468
9.032396	8.825093	9.14473	8.905782
9.032396	8.825093	9.14473	8.905782
12.370878	13.82473	14.148254	13.965917
2.350709	3.7574043	2.3618152	2.352752
9.138176	10.208026	9.869025	10.003053
6.5068955	5.175134	3.7096405	4.325995
6.5068955	5.175134	3.7096405	4.325995

ASHGA5P027994	0.025876485	0.100335141	2.2658034	up	noncoding
ASHGA5P027994	0.025876485	0.100335141	2.2658034	up	noncoding
ASHGA5P027994	0.025876485	0.100335141	2.2658034	up	noncoding
ASHGA5P018324	0.013148317	0.070198708	2.3321071	up	noncoding
ASHGA5P030238	0.015643389	0.076553979	2.7238014	up	noncoding
ASHGA5P020922	1.23407E-06	0.001921139	4.4081106	up	noncoding
ASHGA5P037725	0.009551006	0.060604099	3.0360418	up	noncoding
ASHGA5P047338	0.045090111	0.136612065	2.1302571	up	noncoding
ASHGA5P047338	0.045090111	0.136612065	2.1302571	up	noncoding
ASHGA5P019665	0.022156245	0.092018282	2.4369091	up	noncoding
ASHGA5P057882	0.010107994	0.062335956	2.0352791	up	noncoding
ASHGA5P035209	0.017505367	0.080423645	3.5020004	up	noncoding
ASHGA5P048599	0.010220804	0.0625836	2.6513131	up	noncoding
ASHGA5P042959	0.044735094	0.135964836	2.3351065	up	noncoding
ASHGA5P045022	0.000251551	0.012149764	3.0997388	up	noncoding
ASHGA5P022714	0.013827929	0.07198363	24.1542675	up	noncoding
ASHGA5P041436	0.005915163	0.048964879	2.7822395	up	noncoding
ASHGA5P041436	0.005915163	0.048964879	2.7822395	up	noncoding
ASHGA5P041436	0.005915163	0.048964879	2.7822395	up	noncoding
ASHGA5P041436	0.005915163	0.048964879	2.7822395	up	noncoding
ASHGA5P041436	0.005915163	0.048964879	2.7822395	up	noncoding
ASHGA5P041436	0.005915163	0.048964879	2.7822395	up	noncoding
ASHGA5P041436	0.005915163	0.048964879	2.7822395	up	noncoding
ASHGA5P041436	0.005915163	0.048964879	2.7822395	up	noncoding
ASHGA5P041436	0.005915163	0.048964879	2.7822395	up	noncoding
ASHGA5P041436	0.005915163	0.048964879	2.7822395	up	noncoding
ASHGA5P041436	0.005915163	0.048964879	2.7822395	up	noncoding
ASHGA5P041436	0.005915163	0.048964879	2.7822395	up	noncoding
ASHGA5P041436	0.005915163	0.048964879	2.7822395	up	noncoding
ASHGA5P041436	0.005915163	0.048964879	2.7822395	up	noncoding
ASHGA5P041436	0.005915163	0.048964879	2.7822395	up	noncoding
ASHGA5P041436	0.005915163	0.048964879	2.7822395	up	noncoding
ASHGA5P041436	0.005915163	0.048964879	2.7822395	up	noncoding
ASHGA5P041436	0.005915163	0.048964879	2.7822395	up	noncoding
ASHGA5P041436	0.005915163	0.048964879	2.7822395	up	noncoding
ASHGA5P026685	0.001042478	0.021871823	2.0269007	up	noncoding
ASHGA5P035703	0.001388567	0.02495816	17.8678608	up	noncoding
ASHGA5P030299	0.005941999	0.049073045	2.0881025	up	noncoding
ASHGA5P031729	0.004570422	0.043045046	2.1705896	up	noncoding
ASHGA5P039481	0.048097598	0.142262701	2.6485015	up	noncoding
ASHGA5P020951	0.007002321	0.052947146	3.4758544	up	noncoding
ASHGA5P056356	0.030176856	0.108909758	2.1358321	up	noncoding
ASHGA5P033338	0.001976642	0.029469164	6.5191428	up	noncoding
ASHGA5P057644	0.000941294	0.020834855	2.7597328	up	noncoding
ASHGA5P048380	0.000642021	0.017037535	3.377434	up	noncoding
ASHGA5P048380	0.000642021	0.017037535	3.377434	up	noncoding
ASHGA5P041620	0.014326714	0.073312647	4.3623281	up	noncoding
ASHGA5P055874	0.015019106	0.075030908	8.282576	up	noncoding
ASHGA5P044581	0.000677191	0.017403699	2.5500919	up	noncoding
ASHGA5P045750	0.020870579	0.088889974	2.6816445	up	noncoding
ASHGA5P036780	0.000440186	0.014708848	4.8110018	up	noncoding
ASHGA5P035371	0.004054082	0.040655911	3.4103689	up	noncoding
ASHGA5P019295	8.10342E-05	0.008717574	2.218445	up	noncoding
ASHGA5P049582	0.001440021	0.025382209	2.196291	up	noncoding
ASHGA5P034257	0.001946188	0.029329994	6.6810835	up	noncoding
ASHGA5P010071	0.017849794	0.08107113	2.1946696	up	noncoding
ASHGA5P042548	0.025308987	0.098977236	2.4037215	up	noncoding
ASHGA5P055860	0.001944845	0.029329994	4.3778239	up	noncoding
ASHGA5P055860	0.001944845	0.029329994	4.3778239	up	noncoding
ASHGA5P055860	0.001944845	0.029329994	4.3778239	up	noncoding
ASHGA5P055860	0.001944845	0.029329994	4.3778239	up	noncoding
ASHGA5P019028	0.003158208	0.036419331	2.7231048	up	noncoding

NR_047672	SLC4A8	RefSeq	520	chr12	+
NR_047672	SLC4A8	RefSeq	520	chr12	+
NR_047672	SLC4A8	RefSeq	520	chr12	+
ENST00000452026	CCT6P3	pseudogene	1383	chr7	+
ENST00000563472	RP11-31E22.1	GENCODE	478	chr15	-
ENST00000521989	RP11-150O12.6	GENCODE	470	chr8	-
ENST00000452785	RP4-533D7.5	GENCODE	359	chr1	+
ENST00000414157	RP11-462L8.1	GENCODE	1041	chr10	+
ENST00000414157	RP11-462L8.1	GENCODE	1041	chr10	+
NR_033890	LOC338817	RefSeq	3684	chr12	+
TCONS_00023170	XLOC_011119	LincRNAs identified	913	chr14	-
TCONS_00004374	XLOC_002258	LincRNAs identified	2671	chr2	-
ENST00000566793	ULK4P2	GENCODE	582	chr15	+
ENST00000523331	RP1-170O19.14	GENCODE	556	chr7	-
ENST00000561978	PCAT1	GENCODE	1992	chr8	+
ENST00000561362	RP11-20G13.3	GENCODE	323	chr15	-
ENST00000502926	PCDHGB8P	GENCODE	1973	chr5	+
ENST00000502926	PCDHGB8P	GENCODE	1973	chr5	+
ENST00000502926	PCDHGB8P	GENCODE	1973	chr5	+
ENST00000502926	PCDHGB8P	GENCODE	1973	chr5	+
ENST00000502926	PCDHGB8P	GENCODE	1973	chr5	+
ENST00000502926	PCDHGB8P	GENCODE	1973	chr5	+
ENST00000502926	PCDHGB8P	GENCODE	1973	chr5	+
ENST00000502926	PCDHGB8P	GENCODE	1973	chr5	+
ENST00000502926	PCDHGB8P	GENCODE	1973	chr5	+
ENST00000502926	PCDHGB8P	GENCODE	1973	chr5	+
ENST00000502926	PCDHGB8P	GENCODE	1973	chr5	+
ENST00000502926	PCDHGB8P	GENCODE	1973	chr5	+
ENST00000502926	PCDHGB8P	GENCODE	1973	chr5	+
ENST00000502926	PCDHGB8P	GENCODE	1973	chr5	+
ENST00000502926	PCDHGB8P	GENCODE	1973	chr5	+
ENST00000502926	PCDHGB8P	GENCODE	1973	chr5	+
ENST00000502926	PCDHGB8P	GENCODE	1973	chr5	+
ENST00000502926	PCDHGB8P	GENCODE	1973	chr5	+
ENST00000502926	PCDHGB8P	GENCODE	1973	chr5	+
ENST00000533708	RP11-113K21.5	GENCODE	825	chr11	+
ENST00000565044	RP11-734K21.5	GENCODE	3273	chr2	+
ENST00000452465	RP11-98G7.1	GENCODE	675	chr1	-
ENST00000565625	RP11-352B15.1	GENCODE	726	chr16	+
uc003hoz.1	AK095285	UCSC_knowngene	2838	chr4	-
ENST00000522471	RP11-150O12.3	GENCODE	397	chr8	-
TCONS_00003675	XLOC_001469	LincRNAs identified	636	chr2	+
ENST00000577281	RP11-57A1.1	GENCODE	636	chr17	+
TCONS_00020322	XLOC_009653	LincRNAs identified	832	chr12	+
ENST00000541092	RP11-685N10.1	GENCODE	581	chr11	-
ENST00000541092	RP11-685N10.1	GENCODE	581	chr11	-
ENST00000412295	CTC-338M12.9	GENCODE	549	chr5	+
ENST00000551881	AL589743.1	GENCODE	2239	chr14	+
ENST00000527908	RP11-429J17.5	GENCODE	299	chr8	-
ENST00000425587	RP11-561O23.8	GENCODE	340	chr9	+
ENST00000449469	RP4-564F22.2	GENCODE	2138	chr20	-
uc002tzb.1	AK128708	UCSC_knowngene	5089	chr2	-
ENST00000477247	FAM86DP	pseudogene	986	chr3	-
ENST00000577198	KRT18P55	GENCODE	1950	chr17	-
uc002pdz.1	AK096622	UCSC_knowngene	3230	chr19	-
ENST00000421599	SNHG11	GENCODE	1046	chr20	+
ENST00000424521	RP3-453D15.1	GENCODE	138	chr6	+
NR_034002	MCF2L-AS1	RefSeq	1173	chr13	-
NR_034002	MCF2L-AS1	RefSeq	1173	chr13	-
NR_034002	MCF2L-AS1	RefSeq	1173	chr13	-
NR_034002	MCF2L-AS1	RefSeq	1173	chr13	-
NR_037894	ZNF670-ZNF695	RefSeq	1119	chr1	-

51818593	51834963		GTACTCAGTTCA(	9498	exon sense-overlappi
51818593	51834963		GTACTCAGTTCA(	9498	exon sense-overlappi
51818593	51834963		GTACTCAGTTCA(	9498	exon sense-overlappi
64525376	64534226	ENST00000434500	CCTCATTTGTAA(	643180	
86848281	86860404		TATAAATGCTGTGAAGGAATATGGC		intronic antisense
37373967	37374539		TGATGGAATTGAGCAGATGGGAAT(		intergenic
46599042	46604753		CTGTTGATGTCAGTGTGTGAAATTC		intronic antisense
33271468	33371030	ENST00000450890	GCATAAACTCACCAGTTTTATCCTT/		intronic antisense
33271468	33371030	ENST00000450890	GCATAAACTCACCAGTTTTATCCTT/		intronic antisense
11700963	11717335		CTACTCTTTCACA	338817	intergenic
99750983	99752177	TCONS_00022869	ACATTCACAGGGTCATTGTGCATTT		intergenic
108845323	108848661		CATCGCCAACAGCTGGCACCAGCAT		intergenic
30865087	30889187		CTGTTGTTGAGGTAATGTCTTTTAT(		intergenic
27226191	27233067		TCTCAAGAAAGCTAGACTCAGAGTA		intergenic
128025398	128033259		GCAGAGAAAGCA	100750225	intergenic
99935383	99936798		GATATGTAAAGTCTGTCATCCATTT(		intergenic
140805852	140807825		AGAATCGAATCT	56120	intron sense-overlapp
140805852	140807825		AGAATCGAATCT	56120	intron sense-overlapp
140805852	140807825		AGAATCGAATCT	56120	intron sense-overlapp
140805852	140807825		AGAATCGAATCT	56120	intron sense-overlapp
140805852	140807825		AGAATCGAATCT	56120	intron sense-overlapp
140805852	140807825		AGAATCGAATCT	56120	intron sense-overlapp
140805852	140807825		AGAATCGAATCT	56120	intron sense-overlapp
140805852	140807825		AGAATCGAATCT	56120	intron sense-overlapp
140805852	140807825		AGAATCGAATCT	56120	intron sense-overlapp
140805852	140807825		AGAATCGAATCT	56120	intron sense-overlapp
140805852	140807825		AGAATCGAATCT	56120	intron sense-overlapp
140805852	140807825		AGAATCGAATCT	56120	intron sense-overlapp
140805852	140807825		AGAATCGAATCT	56120	intron sense-overlapp
140805852	140807825		AGAATCGAATCT	56120	intron sense-overlapp
140805852	140807825		AGAATCGAATCT	56120	intron sense-overlapp
140805852	140807825		AGAATCGAATCT	56120	intron sense-overlapp
140805852	140807825		AGAATCGAATCT	56120	intron sense-overlapp
140805852	140807825		AGAATCGAATCT	56120	intron sense-overlapp
140805852	140807825		AGAATCGAATCT	56120	intron sense-overlapp
82783159	82784425		CAAAGTTAATTGTCTTTTGAGTGAT		bidirectional
9246721	9249994		TAAAATGTGATCTATTAGCACAGCT		intergenic
156422688	156426369		GGTGAGGTAGAAAGTATTGCTTAAT		intergenic
34982727	34990167		GGAGGCTTTTGGATTGCCTCTCCAT		intergenic
84889234	85220322	ENST00000508406	CTTGATTCTATACAGAGTGGACCTT(		intergenic
37455152	37456296		AGGGGTGCAGGGCTTCCCAGGAAT(		intergenic
52925299	52926173		TAGCACCAAGAATGAAGTAGAAGA(		intergenic
70422079	70424796		CAGAAAAAGGGGAGTAATTCATTTC		intergenic
9600968	9614245	NR_038927	TGGAATGACAGGTAATGGAGAGTG/		intergenic
94206073	94206736		GGCACAATGGCATCATGTTGTATTT(		intron sense-overlapp
94206073	94206736		GGCACAATGGCATCATGTTGTATTT(		intron sense-overlapp
180708375	180710653		TAGTAGGAGCCTTGGAGGGTGGGT/		intergenic
19650077	19663469	uc002tte.3	CAAAACGACTAAGCTACAATGTTTT		intergenic
144800415	144801061		CACCCATAAACTCAAACACCAGGTA		natural antisense
70843566	70844228	ENST00000422178,	GGCAAATCAACTGTTAACTTTGCAG		intergenic
37049238	37057973		TTCTTGTCCATCTCTGGAATTTTGGC		intergenic
157193083	157198172		GGCCTCTTTCCTATGCATTAAAATA(		intron sense-overlapp
75472111	75484261	ENST00000338711,	CCTTCCCCTGAG(	692099	
26603011	26634408		TGATACCTTAAG(	284085	intergenic
46560931	46580887		GATGCCTGTAACTACCCAGATGAAA		intergenic
37075333	37079564	ENST00000420006,	AGACTGGCGGGC	128439	intergenic
99006309	99006447		GGAGTTGGTTTTTACTCCCAAGCCC		intergenic
113621797	113622952		ATTACAGAATAA(	100289410	intronic antisense
113621797	113622952		ATTACAGAATAA(	100289410	intronic antisense
113621797	113622952		ATTACAGAATAA(	100289410	natural antisense
113621797	113622952		ATTACAGAATAA(	100289410	natural antisense
247108848	247242115		TACAGAAACCTG.	100533111	exon sense-overlappi

NM_001258402	SLC4A8	electroneutral sodiun	+	51818593	51868361
NM_001258403	SLC4A8	electroneutral sodiun	+	51818900	51874391
NM_001267615	SLC4A8	electroneutral sodiun	+	51785100	51868361
NM_152336	AGBL1	cytosolic carboxypep	+	86685241	87572283
ENST00000540385	PIK3R3	phosphoinositide-3-k	-	46509332	46642160
ENST00000475184	ITGB1	integrin, beta 1 (fibrc	-	33218751	33294720
ENST00000534049	ITGB1	integrin, beta 1 (fibrc	-	33217139	33281391
NM_003736	PCDHGB4	protocadherin gamma	+	140767451	140892546
NM_018912	PCDHGA1	protocadherin gamma	+	140710251	140892546
NM_018913	PCDHGA10	protocadherin gamma	+	140792742	140892546
NM_018914	PCDHGA11	protocadherin gamma	+	140800536	140892546
NM_018915	PCDHGA2	protocadherin gamma	+	140718353	140892546
NM_018916	PCDHGA3	protocadherin gamma	+	140723600	140892546
NM_018917	PCDHGA4	protocadherin gamma	+	140734767	140892546
NM_018918	PCDHGA5	protocadherin gamma	+	140743897	140892546
NM_018919	PCDHGA6	protocadherin gamma	+	140753650	140892546
NM_018920	PCDHGA7	protocadherin gamma	+	140762466	140892546
NM_018921	PCDHGA9	protocadherin gamma	+	140782519	140892546
NM_018922	PCDHGB1	protocadherin gamma	+	140729827	140892546
NM_018923	PCDHGB2	protocadherin gamma	+	140739702	140892546
NM_018924	PCDHGB3	protocadherin gamma	+	140749961	140892546
NM_018925	PCDHGB5	protocadherin gamma	+	140777694	140892546
NM_018926	PCDHGB6	protocadherin gamma	+	140787769	140892546
NM_018927	PCDHGB7	protocadherin gamma	+	140797259	140892548
NM_032088	PCDHGA8	protocadherin gamma	+	140771482	140892546
NM_032092	PCDHGA11	protocadherin gamma	+	140800536	140892546
NM_014488	RAB30	ras-related protein Ra	-	82692477	82782884
NM_005590	MRE11A	double-strand break r	-	94150468	94227040
NM_005591	MRE11A	double-strand break r	-	94150468	94227040
NM_139021	MAPK15	mitogen-activated pro	+	144798506	144804633
ENST00000409572	NR4A2	nuclear receptor subf	-	157181609	157198860
ENST00000375608	MCF2L	MCF.2 cell line deriv	+	113548691	113751175
ENST00000442652	MCF2L	MCF.2 cell line deriv	+	113556506	113750999
ENST00000375604	MCF2L	MCF.2 cell line deriv	+	113622756	113752862
ENST00000397030	MCF2L	MCF.2 cell line deriv	+	113622813	113754053
NM_001204220	ZNF670	zinc finger protein 67	-	247197939	247242115

107.86383	35.50309	6.14855	4.968527	128.64229	24.02607
107.86383	35.50309	6.14855	4.968527	128.64229	24.02607
107.86383	35.50309	6.14855	4.968527	128.64229	24.02607
844.906145	207.1976	9.204699	7.983065	748.4905	557.9835
47.647194	13.921562	5.193063	3.747442	42.135574	52.60073
510.03496	67.57841	8.44772	6.30756	281.68027	259.43854
185.792895	37.534664	7.072712	5.470521	217.36655	106.3631
70.731201	24.486553	5.770876	4.679848	72.62244	90.90896
70.731201	24.486553	5.770876	4.679848	72.62244	90.90896
78.725725	19.413765	5.460347	4.175294	70.03464	115.6934
177.342987	55.821793	7.057135	6.031908	166.98718	149.23347
72.434885	16.448555	5.691139	3.88296	46.638927	38.82302
70.53886	17.638601	5.729429	4.322722	61.446823	70.41041
66.077203	21.198748	5.66652	4.443032	83.73341	78.51872
21.931971	5.290486	4.111353	2.479206	21.73728	18.074247
890.573047	9.258389	8.083002	3.488796	1244.7902	35.03562
42.456156	9.87091	4.968655	3.492409	33.32528	49.838715
42.456156	9.87091	4.968655	3.492409	33.32528	49.838715
42.456156	9.87091	4.968655	3.492409	33.32528	49.838715
42.456156	9.87091	4.968655	3.492409	33.32528	49.838715
42.456156	9.87091	4.968655	3.492409	33.32528	49.838715
42.456156	9.87091	4.968655	3.492409	33.32528	49.838715
42.456156	9.87091	4.968655	3.492409	33.32528	49.838715
42.456156	9.87091	4.968655	3.492409	33.32528	49.838715
42.456156	9.87091	4.968655	3.492409	33.32528	49.838715
42.456156	9.87091	4.968655	3.492409	33.32528	49.838715
42.456156	9.87091	4.968655	3.492409	33.32528	49.838715
42.456156	9.87091	4.968655	3.492409	33.32528	49.838715
42.456156	9.87091	4.968655	3.492409	33.32528	49.838715
42.456156	9.87091	4.968655	3.492409	33.32528	49.838715
42.456156	9.87091	4.968655	3.492409	33.32528	49.838715
42.456156	9.87091	4.968655	3.492409	33.32528	49.838715
42.456156	9.87091	4.968655	3.492409	33.32528	49.838715
42.456156	9.87091	4.968655	3.492409	33.32528	49.838715
42.456156	9.87091	4.968655	3.492409	33.32528	49.838715
632.895632	193.883248	8.881323	7.862047	554.27496	588.31274
279.935728	8.824051	7.24649	3.087195	148.30371	85.40608
49.62385	17.407375	5.216719	4.154526	60.65814	33.23349
378.132655	108.017428	8.084222	6.966135	584.9584	578.8141
38.552872	7.836991	4.589587	3.184411	51.42419	65.68764
947.865043	158.20803	9.365394	7.568026	1347.8768	1315.2273
84.46881	28.817354	6.007251	4.912452	74.98742	85.13991
71.280791	6.07078	5.398033	2.693351	23.871544	20.940212
207.439748	48.077869	7.29536	5.830832	212.70377	239.36139
37.4047	8.765616	4.763782	3.007855	43.48422	30.543139
37.4047	8.765616	4.763782	3.007855	43.48422	30.543139
1026.980232	108.55019	9.19693	7.071832	1068.973	1805.054
315.683857	15.94658	6.895014	3.844934	519.42957	18.79172
320.448195	77.791191	7.918539	6.56799	385.16113	410.5553
132.923671	24.306707	6.282089	4.858971	112.05051	30.606562
148.656766	18.569507	6.736609	4.470271	217.05188	252.64128
857.832272	179.061361	9.294186	7.524259	854.9298	675.21375
413.868723	108.334698	8.247311	7.097762	438.12247	532.21075
102067.425	28867.64617	16.239599	15.10453	110990.05	126368.98
88.088158	6.712063	5.772926	3.032844	46.59965	42.44618
555.415997	155.103868	8.655498	7.521494	607.1952	731.8599
52.318586	11.851209	5.025637	3.760367	90.07768	110.96472
3665.6193	589.851688	11.296252	9.166038	2617.8833	1956.2397
3665.6193	589.851688	11.296252	9.166038	2617.8833	1956.2397
3665.6193	589.851688	11.296252	9.166038	2617.8833	1956.2397
3665.6193	589.851688	11.296252	9.166038	2617.8833	1956.2397
816.707037	173.537282	9.182768	7.737516	647.07135	559.1773

223.77031	67.2321	100.61742	102.89479	51.346947	10.46524
223.77031	67.2321	100.61742	102.89479	51.346947	10.46524
223.77031	67.2321	100.61742	102.89479	51.346947	10.46524
582.1936	475.08417	1206.2609	1499.4242	232.16895	168.55089
32.595165	36.85378	61.526917	60.171	4.9999995	7.261096
280.03812	669.70483	724.6438	844.7042	40.662186	33.79669
279.6894	91.68918	208.15828	211.49086	24.2306	19.378305
49.81523	70.89594	56.023735	84.1209	25.040485	34.17375
49.81523	70.89594	56.023735	84.1209	25.040485	34.17375
17.726639	158.39757	12.14804	98.35406	29.059378	40.37972
194.30202	282.80698	99.1972	171.53107	50.377045	23.206799
68.452896	148.98357	42.111725	89.599174	9.342443	4.9999995
43.167347	53.203648	92.80465	102.20028	13.958071	8.118657
41.318195	73.49135	69.8561	49.545444	19.147888	5.838301
30.218454	16.860043	19.134052	25.56775	4.9999995	4.9999995
2366.1023	25.679619	924.978	746.85254	11.98919	10.151031
21.42412	31.047674	64.28941	54.811737	17.70879	12.387441
21.42412	31.047674	64.28941	54.811737	17.70879	12.387441
21.42412	31.047674	64.28941	54.811737	17.70879	12.387441
21.42412	31.047674	64.28941	54.811737	17.70879	12.387441
21.42412	31.047674	64.28941	54.811737	17.70879	12.387441
21.42412	31.047674	64.28941	54.811737	17.70879	12.387441
21.42412	31.047674	64.28941	54.811737	17.70879	12.387441
21.42412	31.047674	64.28941	54.811737	17.70879	12.387441
21.42412	31.047674	64.28941	54.811737	17.70879	12.387441
21.42412	31.047674	64.28941	54.811737	17.70879	12.387441
21.42412	31.047674	64.28941	54.811737	17.70879	12.387441
21.42412	31.047674	64.28941	54.811737	17.70879	12.387441
21.42412	31.047674	64.28941	54.811737	17.70879	12.387441
21.42412	31.047674	64.28941	54.811737	17.70879	12.387441
21.42412	31.047674	64.28941	54.811737	17.70879	12.387441
21.42412	31.047674	64.28941	54.811737	17.70879	12.387441
21.42412	31.047674	64.28941	54.811737	17.70879	12.387441
21.42412	31.047674	64.28941	54.811737	17.70879	12.387441
21.42412	31.047674	64.28941	54.811737	17.70879	12.387441
464.21344	513.98035	782.8798	893.7125	175.83888	93.5794
206.77411	62.22811	653.22736	523.675	7.8180265	4.9999995
65.134544	64.45637	20.277205	53.983353	22.352121	8.668623
283.45456	207.55489	312.57492	301.43906	149.87509	84.61679
23.29382	55.2054	4.9999995	30.706184	7.9755526	7.6964626
1342.4589	533.13226	530.3768	618.1182	126.76771	99.82949
62.309242	59.494537	111.80867	113.07308	29.628098	15.419679
25.828829	135.6963	86.27747	135.07039	4.9999995	4.9999995
168.00381	126.30031	256.41696	241.85225	35.908348	25.041553
70.229744	34.685757	13.475449	32.00989	14.262449	4.9999995
70.229744	34.685757	13.475449	32.00989	14.262449	4.9999995
443.36667	139.95752	1501.6431	1202.8871	162.7203	74.90882
906.3671	264.17987	27.411974	157.92291	22.218536	17.20053
233.04207	278.4453	245.56334	369.92203	92.88741	42.372505
159.65172	295.74304	28.168875	171.32132	27.059153	15.210139
133.4405	117.616516	85.61811	85.57231	21.38899	17.228142
964.29266	1255.0262	473.59695	923.93427	204.79602	48.26521
216.88408	291.0762	514.6454	490.27344	123.714966	111.168495
76091.54	58039.1	123531.24	117383.64	27171.002	16959.238
34.51237	52.006798	191.8832	161.08075	8.151568	5.8161244
358.2444	300.03278	721.3404	613.8233	145.27019	83.53066
25.925142	19.418911	44.226227	23.298834	14.4674	9.48504
2744.8218	5287.1333	3126.6523	6260.9854	222.89943	162.6775
2744.8218	5287.1333	3126.6523	6260.9854	222.89943	162.6775
2744.8218	5287.1333	3126.6523	6260.9854	222.89943	162.6775
2744.8218	5287.1333	3126.6523	6260.9854	222.89943	162.6775
506.49847	637.0344	1281.4468	1269.0139	171.5191	98.71382

93.16481	31.1859	10.79869	16.05695	6.557001	4.543225
93.16481	31.1859	10.79869	16.05695	6.557001	4.543225
93.16481	31.1859	10.79869	16.05695	6.557001	4.543225
338.32623	155.4793	218.56041	130.09982	9.080026	8.814412
4.9999995	20.984755	24.194492	21.08903	4.9351153	5.579085
53.989864	94.37805	104.01772	78.62595	7.683443	7.7550197
36.548252	61.483837	43.367443	40.199547	7.3133464	6.515517
4.9999995	42.32939	17.417109	22.958584	5.7242975	6.2998967
4.9999995	42.32939	17.417109	22.958584	5.7242975	6.2998967
8.757072	24.879929	4.9999995	8.406489	5.671432	6.632685
82.76833	57.607685	73.15811	47.81279	6.9255586	6.9873548
6.4265995	15.918031	38.87111	23.133144	5.085977	5.1790113
24.75542	27.56194	12.1389885	19.29853	5.4826636	5.9596243
36.4744	11.761491	33.9129	20.057507	5.9354773	6.10652
4.9999995	4.9999995	6.7429185	4.9999995	3.9817667	4.170391
7.4328275	10.5543995	6.3375077	9.08538	9.80426	5.046617
5.168099	7.603021	10.178859	6.1792483	4.5960293	5.505192
5.168099	7.603021	10.178859	6.1792483	4.5960293	5.505192
5.168099	7.603021	10.178859	6.1792483	4.5960293	5.505192
5.168099	7.603021	10.178859	6.1792483	4.5960293	5.505192
5.168099	7.603021	10.178859	6.1792483	4.5960293	5.505192
5.168099	7.603021	10.178859	6.1792483	4.5960293	5.505192
5.168099	7.603021	10.178859	6.1792483	4.5960293	5.505192
5.168099	7.603021	10.178859	6.1792483	4.5960293	5.505192
5.168099	7.603021	10.178859	6.1792483	4.5960293	5.505192
5.168099	7.603021	10.178859	6.1792483	4.5960293	5.505192
5.168099	7.603021	10.178859	6.1792483	4.5960293	5.505192
5.168099	7.603021	10.178859	6.1792483	4.5960293	5.505192
5.168099	7.603021	10.178859	6.1792483	4.5960293	5.505192
5.168099	7.603021	10.178859	6.1792483	4.5960293	5.505192
5.168099	7.603021	10.178859	6.1792483	4.5960293	5.505192
5.168099	7.603021	10.178859	6.1792483	4.5960293	5.505192
5.168099	7.603021	10.178859	6.1792483	4.5960293	5.505192
5.168099	7.603021	10.178859	6.1792483	4.5960293	5.505192
5.168099	7.603021	10.178859	6.1792483	4.5960293	5.505192
282.1495	153.1785	286.92517	171.62804	8.632329	8.889557
19.792871	8.259505	4.9999995	7.0739064	6.7571926	6.2203083
41.014557	11.866702	8.998556	11.54369	5.462497	4.978508
199.23682	82.11003	67.88835	64.37749	8.716214	8.867599
12.673918	4.9999995	6.354533	7.3214793	5.217492	5.864086
168.12657	109.25709	283.88565	161.38167	9.931617	10.001391
69.80784	18.781387	23.96985	15.297271	5.7745996	6.21594
6.6836896	6.2672763	4.9999995	8.473717	4.1171703	4.3557043
44.21215	49.243763	81.452065	52.609337	7.283653	7.6408815
17.462732	5.868516	4.9999995	4.9999995	4.984706	4.866904
17.462732	5.868516	4.9999995	4.9999995	4.984706	4.866904
110.38511	114.870605	97.192604	91.2237	9.589903	10.415147
37.905796	5.677243	4.9999995	7.677376	8.536068	4.2195354
109.944786	84.149635	67.66612	69.72669	8.130996	8.375731
34.784157	28.703142	17.170118	22.913532	6.3593407	4.8700476
23.951176	12.3753395	13.2250395	23.248356	7.3100233	7.7153516
422.36887	108.45183	178.13011	112.356125	9.277597	9.072867
84.662025	105.51871	125.73909	99.2049	8.309637	8.747149
35241.184	14536.003	50759.645	28538.805	16.445969	16.660711
5.5115175	8.002172	5.0082316	7.7827625	5.0843563	5.2965736
291.64554	149.9965	142.54568	117.634636	8.77201	9.191759
20.33079	6.557738	9.658116	10.6081705	6.039762	6.5735674
295.0955	573.1778	1501.262	783.9979	10.868176	10.515139
295.0955	573.1778	1501.262	783.9979	10.868176	10.515139
295.0955	573.1778	1501.262	783.9979	10.868176	10.515139
295.0955	573.1778	1501.262	783.9979	10.868176	10.515139
271.55383	184.43912	169.67583	145.32199	8.865744	8.816502

7.4838247	5.702115	6.439118	6.1660147	5.918398	4.1750994
7.4838247	5.702115	6.439118	6.1660147	5.918398	4.1750994
7.4838247	5.702115	6.439118	6.1660147	5.918398	4.1750994
8.825093	8.577859	9.970528	9.960274	8.111857	8.246369
4.715737	4.770769	5.751959	5.405715	2.3467326	3.6227787
7.792295	9.07921	9.238177	9.138176	5.5796084	5.9179115
7.790839	6.1650643	7.460996	7.190512	4.8404107	5.1021514
5.321934	5.7815433	5.621803	5.8757806	4.888808	5.9356117
5.321934	5.7815433	5.621803	5.8757806	4.888808	5.9356117
3.8286333	6.98551	3.5443516	6.0994687	5.0903254	6.1791477
7.28551	7.8251305	6.4187746	6.9004803	5.8917165	5.3650203
5.787242	6.8913126	5.235543	5.967749	3.4370816	2.4279919
5.108251	5.345372	6.32572	6.1549444	4.029031	3.788291
5.0510387	5.839262	5.92506	5.141764	4.4955463	3.2875323
4.6095304	3.5250933	4.1607585	4.2205772	2.3467326	2.4279919
10.865225	4.2108264	9.6070795	8.964006	3.8143277	4.1269555
4.1030116	4.511938	5.811692	5.2840695	4.3845143	4.425802
4.1030116	4.511938	5.811692	5.2840695	4.3845143	4.425802
4.1030116	4.511938	5.811692	5.2840695	4.3845143	4.425802
4.1030116	4.511938	5.811692	5.2840695	4.3845143	4.425802
4.1030116	4.511938	5.811692	5.2840695	4.3845143	4.425802
4.1030116	4.511938	5.811692	5.2840695	4.3845143	4.425802
4.1030116	4.511938	5.811692	5.2840695	4.3845143	4.425802
4.1030116	4.511938	5.811692	5.2840695	4.3845143	4.425802
4.1030116	4.511938	5.811692	5.2840695	4.3845143	4.425802
4.1030116	4.511938	5.811692	5.2840695	4.3845143	4.425802
4.1030116	4.511938	5.811692	5.2840695	4.3845143	4.425802
4.1030116	4.511938	5.811692	5.2840695	4.3845143	4.425802
4.1030116	4.511938	5.811692	5.2840695	4.3845143	4.425802
4.1030116	4.511938	5.811692	5.2840695	4.3845143	4.425802
4.1030116	4.511938	5.811692	5.2840695	4.3845143	4.425802
4.1030116	4.511938	5.811692	5.2840695	4.3845143	4.425802
4.1030116	4.511938	5.811692	5.2840695	4.3845143	4.425802
4.1030116	4.511938	5.811692	5.2840695	4.3845143	4.425802
4.1030116	4.511938	5.811692	5.2840695	4.3845143	4.425802
8.507132	8.686811	9.350787	9.221321	7.711773	7.4002895
7.376218	5.584634	9.084705	8.45588	3.163977	2.4279919
5.718527	5.638229	4.2401876	5.2623625	4.718999	3.8908231
7.810547	7.3829193	8.038058	7.689992	7.4670644	7.2536974
4.2330384	5.400207	2.3344212	4.48828	3.1957858	3.7047482
10.030225	8.737794	8.798042	8.693295	7.2180247	7.4977984
5.6540127	5.514168	6.5870624	6.2977214	5.1202636	4.7626176
4.3812876	6.7564874	6.2253547	6.5521927	2.3467326	2.4279919
7.061858	6.649456	7.7637186	7.372594	5.402174	5.477331
5.821973	4.67796	3.6858318	4.545319	4.062689	2.4279919
5.821973	4.67796	3.6858318	4.545319	4.062689	2.4279919
8.442973	6.802757	10.281227	9.649572	7.5946083	7.0719175
9.4567795	7.730387	4.6493225	6.7779894	4.709844	4.924436
7.540243	7.804861	7.6957006	7.9637036	6.7688084	6.2520556
6.9832497	7.89346	4.6874986	6.8989396	4.996209	4.7419906
6.733618	6.545861	6.215357	5.899441	4.6568856	4.9273515
9.545251	9.976512	8.628458	9.264433	7.9243445	6.4385448
7.4403353	7.871956	8.752054	8.362733	7.1843734	7.6452236
15.911007	15.667396	16.499355	16.253157	15.006781	14.9627075
4.7958937	5.3065453	7.349266	6.804921	3.2307165	3.2795393
8.145644	7.9107165	9.229785	8.683072	7.41358	7.237836
4.387062	3.7524185	5.305802	4.0952086	4.0842423	4.0223045
11.053577	12.027215	11.31948	11.993926	8.0526285	8.195155
11.053577	12.027215	11.31948	11.993926	8.0526285	8.195155
11.053577	12.027215	11.31948	11.993926	8.0526285	8.195155
11.053577	12.027215	11.31948	11.993926	8.0526285	8.195155
8.6294	9.00169	10.051112	9.732162	7.669079	7.4807715

6.5068955	5.175134	3.7096405	4.325995
6.5068955	5.175134	3.7096405	4.325995
6.5068955	5.175134	3.7096405	4.325995
8.348345	7.6554623	8.024633	7.5117216
2.350709	4.558445	4.867207	4.738779
5.746815	6.8911066	6.956998	6.7529187
5.1937704	6.2365017	5.711576	5.7387147
2.350709	5.643138	4.3941407	4.8666825
2.350709	5.643138	4.3941407	4.8666825
3.252148	4.823413	2.3618152	3.3449166
6.338797	6.135362	6.456041	6.0045123
2.8702238	4.135307	5.5495715	4.8775835
4.653181	4.9856176	3.8790774	4.601135
5.1916485	3.6665664	5.357901	4.6589975
2.350709	2.3408759	3.0561757	2.352752
3.0429091	3.5060802	2.9788454	3.4636588
2.6158013	3.015297	3.62583	2.8872082
2.6158013	3.015297	3.62583	2.8872082
2.6158013	3.015297	3.62583	2.8872082
2.6158013	3.015297	3.62583	2.8872082
2.6158013	3.015297	3.62583	2.8872082
2.6158013	3.015297	3.62583	2.8872082
2.6158013	3.015297	3.62583	2.8872082
2.6158013	3.015297	3.62583	2.8872082
2.6158013	3.015297	3.62583	2.8872082
2.6158013	3.015297	3.62583	2.8872082
2.6158013	3.015297	3.62583	2.8872082
2.6158013	3.015297	3.62583	2.8872082
2.6158013	3.015297	3.62583	2.8872082
2.6158013	3.015297	3.62583	2.8872082
2.6158013	3.015297	3.62583	2.8872082
2.6158013	3.015297	3.62583	2.8872082
2.6158013	3.015297	3.62583	2.8872082
2.6158013	3.015297	3.62583	2.8872082
2.6158013	3.015297	3.62583	2.8872082
8.0867605	7.6348925	8.411921	7.926648
4.351688	3.1336715	2.3618152	3.0840242
5.3565598	3.6799047	3.4553642	3.8255053
7.5875382	6.684485	6.3515396	6.452483
3.745258	2.3408759	2.9824033	3.1373956
7.342331	7.1149187	8.397126	7.8379593
6.0994687	4.383066	4.853403	4.2558956
2.9149988	2.7509098	2.3618152	3.357655
5.4641356	5.890881	6.604062	6.146406
4.1782403	2.66364	2.3618152	2.352752
4.1782403	2.66364	2.3618152	2.352752
6.7486825	7.194019	6.859071	6.962691
5.2455544	2.6192493	2.3618152	3.2087057
6.7440724	6.7238874	6.3478794	6.5712366
5.128867	5.0491295	4.373925	4.863707
4.6080914	3.7456305	3.9994793	4.8841896
8.676496	7.1046176	7.7212687	7.2802796
6.3707705	7.071259	7.220653	7.094292
15.033827	14.272763	15.941489	15.409612
2.687208	3.090545	2.6781585	3.230896
8.135238	7.6024404	7.390699	7.3491707
4.387575	2.8136933	3.5542767	3.7001102
8.150287	9.623875	10.800699	10.173585
8.150287	9.623875	10.800699	10.173585
8.150287	9.623875	10.800699	10.173585
8.150287	9.623875	10.800699	10.173585
8.033613	7.9186974	7.6462	7.676734

ASHGA5P019028	0.003158208	0.036419331	2.7231048	up	noncoding
ASHGA5P019028	0.003158208	0.036419331	2.7231048	up	noncoding
ASHGA5P019028	0.003158208	0.036419331	2.7231048	up	noncoding
ASHGA5P029048	0.000101631	0.009142535	2.3363494	up	noncoding
ASHGA5P051945	0.009762279	0.061334043	2.1795039	up	noncoding
ASHGA5P021677	4.93199E-05	0.007378879	2.5459392	up	noncoding
ASHGA5P021677	4.93199E-05	0.007378879	2.5459392	up	noncoding
ASHGA5P021677	4.93199E-05	0.007378879	2.5459392	up	noncoding
ASHGA5P046369	0.01174859	0.066707739	2.1168998	up	noncoding
ASHGA5P046369	0.01174859	0.066707739	2.1168998	up	noncoding
ASHGA5P046369	0.01174859	0.066707739	2.1168998	up	noncoding
ASHGA5P046369	0.01174859	0.066707739	2.1168998	up	noncoding
ASHGA5P046369	0.01174859	0.066707739	2.1168998	up	noncoding
ASHGA5P023105	0.002476491	0.032328309	4.8525609	up	noncoding
ASHGA5P028420	0.001894347	0.029145997	2.98288	up	noncoding
ASHGA5P043647	0.02753922	0.10367037	2.6601935	up	noncoding
ASHGA5P033327	0.04644992	0.139076571	2.0091339	up	noncoding
ASHGA5P023932	0.016158815	0.077740057	2.8561373	up	noncoding
ASHGA5P019290	0.004749027	0.043793775	3.0430231	up	noncoding
ASHGA5P019290	0.004749027	0.043793775	3.0430231	up	noncoding
ASHGA5P019290	0.004749027	0.043793775	3.0430231	up	noncoding
ASHGA5P019290	0.004749027	0.043793775	3.0430231	up	noncoding
ASHGA5P035999	0.000258044	0.012170274	3.0677421	up	noncoding
ASHGA5P019608	0.006272828	0.05052414	2.4036721	up	noncoding
ASHGA5P052417	0.000365036	0.013617271	7.0007248	up	noncoding
ASHGA5P052417	0.000365036	0.013617271	7.0007248	up	noncoding
ASHGA5P035220	0.017243265	0.079995624	2.3881179	up	noncoding
ASHGA5P028708	0.000129557	0.010460795	2.2159346	up	noncoding
ASHGA5P028708	0.000129557	0.010460795	2.2159346	up	noncoding
ASHGA5P028708	0.000129557	0.010460795	2.2159346	up	noncoding
ASHGA5P028708	0.000129557	0.010460795	2.2159346	up	noncoding
ASHGA5P028503	0.009691162	0.061042328	2.1589977	up	noncoding
ASHGA5P038049	0.001482688	0.025652132	2.7409518	up	noncoding
ASHGA5P038049	0.001482688	0.025652132	2.7409518	up	noncoding
ASHGA5P038049	0.001482688	0.025652132	2.7409518	up	noncoding
ASHGA5P038049	0.001482688	0.025652132	2.7409518	up	noncoding
ASHGA5P017189	0.006421986	0.050969566	2.6158373	up	noncoding
ASHGA5P030683	0.04902838	0.143860354	3.9924104	up	noncoding
ASHGA5P026001	0.01164981	0.066443866	2.5792308	up	noncoding
ASHGA5P030794	0.000910504	0.02038276	2.6436897	up	noncoding
ASHGA5P044944	0.01750118	0.080423645	2.0000403	up	noncoding
ASHGA5P041281	7.1872E-06	0.003887603	22.0846006	up	noncoding
ASHGA5P036817	0.015595958	0.07641666	2.1829021	up	noncoding
ASHGA5P035523	0.000318375	0.012923818	5.8333715	up	noncoding
ASHGA5P036776	0.001118745	0.022608782	2.6662357	up	noncoding
ASHGA5P019958	0.013611377	0.071676095	2.1780808	up	noncoding
ASHGA5P052066	8.76159E-05	0.008766146	2.1719444	up	noncoding
ASHGA5P035623	0.001481762	0.025652132	4.1753088	up	noncoding
ASHGA5P016692	0.000894402	0.020125432	2.654483	up	noncoding
ASHGA5P048256	0.000350935	0.013409132	2.021685	up	noncoding
ASHGA5P042821	0.02202383	0.091769495	3.9831349	up	noncoding
ASHGA5P034586	0.014298077	0.073284489	2.0811012	up	noncoding
ASHGA5P037245	2.16946E-05	0.005043652	2.9705319	up	noncoding
ASHGA5P017065	0.003319571	0.037316455	3.1483472	up	noncoding
ASHGA5P017065	0.003319571	0.037316455	3.1483472	up	noncoding
ASHGA5P037315	0.008397347	0.057281529	2.6799961	up	noncoding
ASHGA5P040423	0.003620117	0.038659181	2.0853846	up	noncoding
ASHGA5P009811	0.014615791	0.073959919	2.6076621	up	noncoding
ASHGA5P009811	0.014615791	0.073959919	2.6076621	up	noncoding
ASHGA5P009811	0.014615791	0.073959919	2.6076621	up	noncoding
ASHGA5P042691	0.001040646	0.021859875	3.2745133	up	noncoding
ASHGA5P017159	0.01520177	0.075446109	2.6347925	up	noncoding

NR_037894	ZNF670-ZNF695	RefSeq	1119	chr1	-
NR_037894	ZNF670-ZNF695	RefSeq	1119	chr1	-
NR_037894	ZNF670-ZNF695	RefSeq	1119	chr1	-
TCONS_00010341	XLOC_004829	LincRNAs identified	378	chr5	-
ENST00000425653	DANCR	GENCODE	823	chr4	+
ENST00000545440	SNHG1	GENCODE	754	chr11	-
ENST00000545440	SNHG1	GENCODE	754	chr11	-
ENST00000545440	SNHG1	GENCODE	754	chr11	-
NR_024501	FMR1-AS1	RefSeq	2644	chrX	-
NR_024501	FMR1-AS1	RefSeq	2644	chrX	-
NR_024501	FMR1-AS1	RefSeq	2644	chrX	-
NR_024501	FMR1-AS1	RefSeq	2644	chrX	-
NR_024501	FMR1-AS1	RefSeq	2644	chrX	-
ENST00000570035	AC004158.2	GENCODE	831	chr16	-
ENST00000423023	PSPC1-OT1	GENCODE	376	chr13	-
ENST00000553157	KDM5B-AS1	GENCODE	551	chr1	+
uc001hwk.4	BC016972	UCSC_knowngene	888	chr1	-
HMlincRNA99+	HMlincRNA99	LincRNAs identified	11479	chr1	+
NR_037583	LEMD1	RefSeq	779	chr1	-
NR_037583	LEMD1	RefSeq	779	chr1	-
NR_037583	LEMD1	RefSeq	779	chr1	-
NR_037583	LEMD1	RefSeq	779	chr1	-
uc002suw.1	BC016831	UCSC_knowngene	1119	chr2	+
NR_024526	ARL6IP6	RefSeq	1804	chr2	+
NR_036536	SNHG4	RefSeq	1047	chr5	+
NR_036536	SNHG4	RefSeq	1047	chr5	+
ENST00000567491	AC133109.1	GENCODE	1604	chr2	-
ENST00000421863	ZMYM2-IT1	GENCODE	550	chr13	+
ENST00000421863	ZMYM2-IT1	GENCODE	550	chr13	+
ENST00000421863	ZMYM2-IT1	GENCODE	550	chr13	+
ENST00000421863	ZMYM2-IT1	GENCODE	550	chr13	+
ENST00000434565	LINC00571	GENCODE	521	chr13	-
ENST00000421637	RP4-784A16.2	GENCODE	675	chr1	+
ENST00000421637	RP4-784A16.2	GENCODE	675	chr1	+
ENST00000421637	RP4-784A16.2	GENCODE	675	chr1	+
ENST00000421637	RP4-784A16.2	GENCODE	675	chr1	+
ENST00000438705	SNHG15	GENCODE	509	chr7	-
ENST00000569846	RP11-160C18.2	GENCODE	568	chr15	+
uc010ady.1	THSD1P1	UCSC_knowngene	2336	chr13	-
uc021sxj.1	AL109706	UCSC_knowngene	2503	chr15	+
ENST00000521625	KB-1083B1.1	GENCODE	449	chr8	+
ENST00000514769	RP11-138J23.1	GENCODE	574	chr5	+
ENST00000419897	RP11-465L10.10	GENCODE	491	chr20	-
ENST00000447111	AC079354.5	GENCODE	311	chr2	-
ENST00000417578	RP4-564F22.2	GENCODE	880	chr20	-
ENST00000505873	AC108142.1	GENCODE	939	chr4	-
ENST00000509671	RP11-125O18.1	GENCODE	241	chr4	-
uc002vvo.1	AF279775	UCSC_knowngene	723	chr2	-
ENST00000433036	AC133528.2	GENCODE	415	chr2	+
ENST00000529253	FAM86C2P	GENCODE	1857	chr11	-
TCONS_00019368	XLOC_009191	LincRNAs identified	1838	chr11	+
ENST00000445300	RP11-431K24.1	GENCODE	2044	chr1	+
ENST00000457162	AF129075.5	GENCODE	355	chr21	-
ENST00000437243	AC092573.3	GENCODE	336	chr2	-
ENST00000437243	AC092573.3	GENCODE	336	chr2	-
ENST00000419664	AF064858.11	GENCODE	509	chr21	-
TCONS_00022202	XLOC_010428	LincRNAs identified	868	chr13	+
NR_026910	ABHD11	RefSeq	1462	chr7	-
NR_026910	ABHD11	RefSeq	1462	chr7	-
NR_026910	ABHD11	RefSeq	1462	chr7	-
TCONS_00014547	XLOC_006924	LincRNAs identified	1836	chr8	+
ENST00000438380	RP11-175D17.3	GENCODE	617	chr9	+

247108848	247242115		TACAGAAACCTG.	100533111	exon sense-overlappi
247108848	247242115		TACAGAAACCTG.	100533111	exon sense-overlappi
247108848	247242115		TACAGAAACCTG.	100533111	exon sense-overlappi
55748450	55752589		ATTTTTTAAGGACCATCTGGGCACA(		intergenic
53578947	53580304		ACCGTAACAATT	57291	intergenic
62619723	62622960	ENST00000537869,	CTGATGACAGTC	23642	bidirectional
62619723	62622960	ENST00000537869,	CTGATGACAGTC	23642	bidirectional
62619723	62622960	ENST00000537869,	CTGATGACAGTC	23642	bidirectional
146990948	147003676	NR_024499, NR_02	AAGACTTTGAAC	100126270	natural antisense
146990948	147003676	NR_024499, NR_02	AAGACTTTGAAC	100126270	natural antisense
146990948	147003676	NR_024499, NR_02	AAGACTTTGAAC	100126270	natural antisense
146990948	147003676	NR_024499, NR_02	AAGACTTTGAAC	100126270	natural antisense
146990948	147003676	NR_024499, NR_02	AAGACTTTGAAC	100126270	natural antisense
72556036	72698908		GATCTCAGGGAGGGGAAGGTATAA		intergenic
20248763	20250024		GAAAAATATACTATGGAGATTAGAT		intergenic
202780081	202781284		GCTGAGGATGGA	100506696	intergenic
235093089	235099746		CCTGGGGGAATGAATCTTTCAGAGC		intergenic
200231279	200242758		TAGGGACTCTTCATATTCATAAGGC.		intergenic
205350505	205391214		CCAATACTACTG(	93273	exon sense-overlappi
205350505	205391214		CCAATACTACTG(	93273	exon sense-overlappi
205350505	205391214		CCAATACTACTG(	93273	exon sense-overlappi
205350505	205391214		CCAATACTACTG(	93273	exon sense-overlappi
96331831	96334463		TCTCACAATTTTGGTTGACAGTCAA(		intergenic
153574406	153617767		TACGCACCAGAG	151188	exon sense-overlappi
138609440	138618873		AGCCCGTAAGCT	724102	exon sense-overlappi
138609440	138618873		AGCCCGTAAGCT	724102	exon sense-overlappi
109743782	109745386	NR_029193	AGCCATTGGGGCTGAATTTTCATTT		bidirectional
20651664	20653696		ATTTCTTCTGTAGATTCTCATTGGTT		intron sense-overlapp
20651664	20653696		ATTTCTTCTGTAGATTCTCATTGGTT		intron sense-overlapp
20651664	20653696		ATTTCTTCTGTAGATTCTCATTGGTT		intron sense-overlapp
20651664	20653696		ATTTCTTCTGTAGATTCTCATTGGTT		intron sense-overlapp
38624953	38717369		TAACAACAAAAT	100874188	intergenic
53753695	53755378		CCGTTGTCACAGGTGAAGTCACTGT		natural antisense
53753695	53755378		CCGTTGTCACAGGTGAAGTCACTGT		natural antisense
53753695	53755378		CCGTTGTCACAGGTGAAGTCACTGT		natural antisense
53753695	53755378		CCGTTGTCACAGGTGAAGTCACTGT		natural antisense
45022683	45026169		CTGAGCCCAGTG	285958	intergenic
78952985	78996226		AACCTGGTTGAATATGGAGTTGATT		intronic antisense
52741846	52768575		ACCTAGTTAGGA.	374500	intergenic
99571772	99574275	TCONS_00023546	CTTGTTAAGTGGAGCTGGGGGATGA		intergenic
101630808	101632221	ENST00000521535	AAGTTACCCACATTTCTGCCCAGCT(		intronic antisense
103415611	103441104		GAAGGCAGTGTACTGAATAGTACCA		intergenic
44649257	44650366	ENST00000535913	TCGCTGGGAGGCCCTGGGCTCCCCA		natural antisense
203043382	203044114		CTTGGATAATATGTAACATTAACAG		intronic antisense
37049239	37063920	ENST00000423536,	CCATGCCTGGAATGACTTTAATAAC		intergenic
183063535	183064929		AAGGATTTCCACCCCCGATTAGCGG		bidirectional
128322880	128391724		GGCTCGCGGGGTGCGGGGGAGCCT(		intergenic
235791851	235797446		GAAAGTGTTGAAAATTGACATCTAT		intergenic
242629828	242633704		GGCCTGATAGACAAAATACATGAAC		intergenic
67559237	67572805	ENST00000525180,	TCTATGTGGCCCT	645332	intergenic
69184376	69187267	ENST00000561588	AAAGAACCAAGAGCTGCAAATGTCC		intergenic
8086797	8182762		ACTGTTTTCCTGAAAACATTGCTTCT		bidirectional
30430393	30431977		TGCACTTAGGTTCTAAAATTATGCT		intron sense-overlapp
174203067	174220936		GAGATGAACTTCTTTGGCAGTTAAC		intronic antisense
174203067	174220936		GAGATGAACTTCTTTGGCAGTTAAC		intronic antisense
40378573	40383255		AGAGTGAGCTTGAAGAACGTGTGAC		intergenic
75017245	75018926		TGAGTGAGAGGCCTCAAGTGACCCT		intergenic
73150426	73153184	NM_001145364, NM	AAACTAACTTCA	83451	exon sense-overlappi
73150426	73153184	NM_001145364, NM	AAACTAACTTCA	83451	exon sense-overlappi
73150426	73153184	NM_001145364, NM	AAACTAACTTCA	83451	exon sense-overlappi
129000922	129004320		AAGAAAGGCCCAGGCATTTGCATCT		intergenic
127532401	127535206		TTAACTCGAGCACTGATGAAGTCCA		intronic antisense

NM_001204221	ZNF695	zinc finger protein 69	-	247108848	247171395
NM_020394	ZNF695	zinc finger protein 69	-	247148624	247171395
NM_033213	ZNF670	zinc finger protein 67	-	247197939	247242115
NM_001012662	SLC3A2	4F2 cell-surface antig	+	62623483	62656355
NM_001012664	SLC3A2	4F2 cell-surface antig	+	62623483	62656355
NM_002394	SLC3A2	4F2 cell-surface antig	+	62623483	62656355
NM_001185075	FMR1	fragile X mental retar	+	146993468	147032647
NM_001185076	FMR1	fragile X mental retar	+	146993468	147032647
NM_001185081	FMR1	fragile X mental retar	+	146993468	147032647
NM_001185082	FMR1	fragile X mental retar	+	146993468	147032647
NM_002024	FMR1	fragile X mental retar	+	146993468	147032647
NM_001001552	LEMD1	LEM domain-contair	-	205350505	205391214
NM_001199050	LEMD1	LEM domain-contair	-	205350505	205391214
NM_001199051	LEMD1	LEM domain-contair	-	205350505	205391214
NM_001199052	LEMD1	LEM domain-contair	-	205350505	205391214
NM_152522	ARL6IP6	ADP-ribosylation fac	+	153574406	153617767
NM_001194954	MATR3	matrin-3 isoform a	+	138609440	138667366
NM_199189	MATR3	matrin-3 isoform a	+	138609440	138667366
NM_001099289	SH3RF3	SH3 domain-containi	+	109745996	110262207
NM_001190964	ZMYM2	zinc finger MYM-typ	+	20532847	20665968
NM_001190965	ZMYM2	zinc finger MYM-typ	+	20532973	20665968
NM_003453	ZMYM2	zinc finger MYM-typ	+	20532809	20665968
NM_197968	ZMYM2	zinc finger MYM-typ	+	20532809	20665968
NM_001018054	LRP8	low-density lipoprote	-	53708040	53793821
NM_004631	LRP8	low-density lipoprote	-	53708040	53793821
NM_017522	LRP8	low-density lipoprote	-	53708040	53793821
NM_033300	LRP8	low-density lipoprote	-	53708040	53793821
ENST00000560511	CHRNB4	cholinergic receptor,	-	78923403	79012628
NM_152628	SNX31	sorting nexin-31	-	101585111	101661893
NM_001134771	SLC12A5	solute carrier family	+	44650328	44688789
ENST00000498697	AC079354.1	Uncharacterized prot	+	202965157	203059074
ENST00000512480	ODZ3	odz, odd Oz/ten-m hc	+	183065842	183268082
NM_018948	ERRFI1	ERBB receptor feedb	-	8071778	8086393
NM_006585	CCT8	T-complex protein 1	-	30428647	30446010
NM_031942	CDCA7	cell division cycle-as	+	174219560	174233718
NM_145810	CDCA7	cell division cycle-as	+	174219560	174233718
NM_001145364	ABHD11	abhydrolase domain-	-	73150424	73153184
NM_148912	ABHD11	abhydrolase domain-	-	73150426	73153184
NM_148913	ABHD11	abhydrolase domain-	-	73150426	73153184
NM_001489	NR6A1	nuclear receptor subf	-	127284702	127533576

816.707037	173.537282	9.182768	7.737516	647.07135	559.1773
816.707037	173.537282	9.182768	7.737516	647.07135	559.1773
816.707037	173.537282	9.182768	7.737516	647.07135	559.1773
50.726197	14.58997	5.287984	4.063728	48.225105	33.475605
129.158616	36.204447	6.587783	5.463784	172.625	199.64175
696.195195	161.362683	9.010984	7.662786	612.79803	536.88135
696.195195	161.362683	9.010984	7.662786	612.79803	536.88135
696.195195	161.362683	9.010984	7.662786	612.79803	536.88135
19.392102	7.921922	3.958802	2.876849	21.935337	17.605732
19.392102	7.921922	3.958802	2.876849	21.935337	17.605732
19.392102	7.921922	3.958802	2.876849	21.935337	17.605732
19.392102	7.921922	3.958802	2.876849	21.935337	17.605732
19.392102	7.921922	3.958802	2.876849	21.935337	17.605732
143.2682	19.623985	6.73303	4.454283	162.76797	161.75323
682.52728	124.497272	8.86292	7.286214	497.8565	299.14673
39.176129	10.054405	4.817742	3.406211	58.603012	70.70001
45.918514	14.60086	5.098123	4.091549	58.336323	43.036213
55.124343	13.712261	5.37494	3.860875	65.06197	84.8882
74.172567	14.60446	5.626328	4.020823	44.15091	14.308191
74.172567	14.60446	5.626328	4.020823	44.15091	14.308191
74.172567	14.60446	5.626328	4.020823	44.15091	14.308191
74.172567	14.60446	5.626328	4.020823	44.15091	14.308191
152.086757	33.768749	6.822618	5.205441	179.00868	152.73381
798.41805	197.61759	9.222907	7.957666	935.887	628.7339
69.548866	6.509752	5.547249	2.739745	35.943745	49.055275
69.548866	6.509752	5.547249	2.739745	35.943745	49.055275
268.63044	69.082358	7.470445	6.21457	189.38176	138.7771
42.839822	13.091207	5.060587	3.912671	41.7199	23.938972
42.839822	13.091207	5.060587	3.912671	41.7199	23.938972
42.839822	13.091207	5.060587	3.912671	41.7199	23.938972
42.839822	13.091207	5.060587	3.912671	41.7199	23.938972
39.552334	11.67367	4.899251	3.788889	36.246025	28.197426
24.302894	6.459921	4.269546	2.814869	27.93751	17.878288
24.302894	6.459921	4.269546	2.814869	27.93751	17.878288
24.302894	6.459921	4.269546	2.814869	27.93751	17.878288
24.302894	6.459921	4.269546	2.814869	27.93751	17.878288
508.8478	114.067294	8.528586	7.141313	629.6647	739.061
69.761771	9.11744	5.048356	3.051096	141.33012	162.4149
185.077872	47.57882	7.133852	5.766911	171.7685	110.5485
131.039897	30.897196	6.66049	5.257937	151.68886	115.17516
293.793497	95.548768	7.761786	6.761757	336.24338	343.48126
172.968924	5.364717	7.025074	2.560105	225.45955	187.4608
52.900191	16.790312	5.344596	4.218348	44.35446	35.48451
291.599293	33.577556	7.713934	5.169604	393.02618	452.3835
26091.43367	5426.811783	14.197728	12.782923	29074.523	36506.35
671.60353	165.728181	8.735759	7.612702	943.1596	1317.7736
813.92628	219.007263	9.261068	8.142081	763.45374	798.0694
909.368173	149.358944	9.348424	7.286541	900.1693	492.0172
949.863848	228.711015	9.492099	8.083668	997.18646	901.64386
562.378703	165.000695	8.710196	7.694637	543.755	692.368
630.354292	82.197743	8.642082	6.648178	805.7482	775.53375
78.874964	27.317252	5.879744	4.822397	103.84826	95.9337
40.256432	9.720112	4.950761	3.38004	37.487537	22.345304
84.66214	16.51056	5.932849	4.278254	101.36038	150.40433
84.66214	16.51056	5.932849	4.278254	101.36038	150.40433
118.633046	27.192449	6.427512	5.005281	83.894554	77.72819
28.75761	8.72641	4.43405	3.373737	43.48235	29.342306
555.681942	152.70552	8.722314	7.339557	588.32166	581.96466
555.681942	152.70552	8.722314	7.339557	588.32166	581.96466
555.681942	152.70552	8.722314	7.339557	588.32166	581.96466
2047.9777	384.798335	10.565602	8.854321	1864.4635	1650.585
36.517909	11.027871	4.829067	3.431378	50.213932	44.42035

506.49847	637.0344	1281.4468	1269.0139	171.5191	98.71382
506.49847	637.0344	1281.4468	1269.0139	171.5191	98.71382
506.49847	637.0344	1281.4468	1269.0139	171.5191	98.71382
33.43718	65.21983	57.66716	66.3323	15.059291	5.748883
89.61505	119.61078	78.89911	114.560005	34.07279	23.44298
594.9506	516.05096	961.513	954.97723	142.14587	133.31451
594.9506	516.05096	961.513	954.97723	142.14587	133.31451
594.9506	516.05096	961.513	954.97723	142.14587	133.31451
23.124285	18.583189	14.586199	20.51787	10.74159	4.9999995
23.124285	18.583189	14.586199	20.51787	10.74159	4.9999995
23.124285	18.583189	14.586199	20.51787	10.74159	4.9999995
23.124285	18.583189	14.586199	20.51787	10.74159	4.9999995
23.124285	18.583189	14.586199	20.51787	10.74159	4.9999995
218.7134	127.31769	69.56811	119.4888	19.13899	7.0374846
671.7106	400.84805	1180.4546	1045.1472	107.3909	93.48283
29.389215	26.004267	21.980158	28.380114	15.090082	4.9999995
75.60868	25.105099	35.376762	38.04801	10.621881	7.8643823
39.06527	37.39281	43.660637	60.67717	13.171989	5.0009046
78.57452	107.85094	78.74001	121.41083	9.360028	7.3912773
78.57452	107.85094	78.74001	121.41083	9.360028	7.3912773
78.57452	107.85094	78.74001	121.41083	9.360028	7.3912773
78.57452	107.85094	78.74001	121.41083	9.360028	7.3912773
196.08191	181.40097	64.8918	138.40337	43.943233	23.625986
1091.8666	640.3271	676.5818	817.1119	202.01132	169.98808
21.78812	107.69773	83.997826	118.8105	4.9999995	4.9999995
21.78812	107.69773	83.997826	118.8105	4.9999995	4.9999995
212.79012	567.77167	106.35649	396.7055	54.58813	82.91065
54.753723	46.356934	37.75202	52.517384	9.5597	6.3394456
54.753723	46.356934	37.75202	52.517384	9.5597	6.3394456
54.753723	46.356934	37.75202	52.517384	9.5597	6.3394456
54.753723	46.356934	37.75202	52.517384	9.5597	6.3394456
35.841743	69.84728	20.5003	46.681232	10.937611	7.3365235
25.582705	35.592434	20.967028	17.859402	4.9999995	4.9999995
25.582705	35.592434	20.967028	17.859402	4.9999995	4.9999995
25.582705	35.592434	20.967028	17.859402	4.9999995	4.9999995
25.582705	35.592434	20.967028	17.859402	4.9999995	4.9999995
499.48523	527.57697	242.04985	415.24905	101.10709	122.39327
21.649734	4.9999995	60.659325	27.516546	9.807784	5.0806937
223.52727	235.83035	140.30331	228.4893	53.990883	31.823553
119.14023	168.40543	89.868355	141.96135	27.298876	32.397057
202.73846	157.4028	409.01343	313.88165	112.508224	57.769928
243.6625	115.676575	122.1997	143.35442	4.9999995	7.1883025
65.00854	79.57936	34.210976	58.763298	6.870341	17.754656
343.53415	183.57144	161.54674	215.53375	45.403683	70.62696
16208.401	14726.477	28813.15	31219.701	5124.52	6240.928
347.96136	189.19832	722.1658	509.3625	209.00859	135.36969
678.4072	1070.4955	670.8852	902.24664	237.69272	210.33847
1305.008	1179.531	528.66754	1050.816	157.43298	35.482105
1039.3778	910.1075	771.70447	1079.163	227.1396	138.64488
376.28082	424.1402	627.0052	710.723	143.1407	150.60808
722.692	95.4579	750.0779	632.616	81.821724	117.38688
97.32485	76.37003	34.945717	64.827225	39.11082	37.09494
52.368748	53.541695	26.801151	48.994156	8.486335	4.9999995
48.24543	86.86746	41.8368	79.25844	21.191414	15.332101
48.24543	86.86746	41.8368	79.25844	21.191414	15.332101
159.35754	211.09872	64.77388	114.94539	24.19035	13.022039
39.518837	29.245485	15.77412	15.182559	11.111439	7.08374
424.76953	583.76166	491.53934	663.7348	125.938774	56.2379
424.76953	583.76166	491.53934	663.7348	125.938774	56.2379
424.76953	583.76166	491.53934	663.7348	125.938774	56.2379
1957.849	2595.979	1714.6235	2504.3662	250.8782	249.74246
31.884237	27.603092	28.783342	36.202503	12.65942	4.9999995

271.55383	184.43912	169.67583	145.32199	8.865744	8.816502
271.55383	184.43912	169.67583	145.32199	8.865744	8.816502
271.55383	184.43912	169.67583	145.32199	8.865744	8.816502
11.619431	20.954058	16.62913	17.52903	5.131353	4.9861116
37.805515	33.26478	52.134308	36.50631	6.9790344	7.392539
181.18706	125.86021	227.15147	158.51698	8.788559	8.760189
181.18706	125.86021	227.15147	158.51698	8.788559	8.760189
181.18706	125.86021	227.15147	158.51698	8.788559	8.760189
16.789942	4.9999995	4.9999995	4.9999995	3.9932153	4.137368
16.789942	4.9999995	4.9999995	4.9999995	3.9932153	4.137368
16.789942	4.9999995	4.9999995	4.9999995	3.9932153	4.137368
16.789942	4.9999995	4.9999995	4.9999995	3.9932153	4.137368
16.789942	4.9999995	4.9999995	4.9999995	3.9932153	4.137368
22.75569	17.342936	32.827625	18.641186	6.890214	7.095615
141.05917	113.57994	165.99118	125.479614	8.479397	7.9522905
11.38789	5.6041284	11.598061	11.646269	5.411232	5.965311
13.521501	19.66866	20.547295	15.381441	5.404421	5.3148355
27.56899	14.062891	14.128131	8.340659	5.56461	6.210959
23.475801	23.52472	12.68577	11.189161	5.007328	3.8650498
23.475801	23.52472	12.68577	11.189161	5.007328	3.8650498
23.475801	23.52472	12.68577	11.189161	5.007328	3.8650498
23.475801	23.52472	12.68577	11.189161	5.007328	3.8650498
69.66627	27.588425	19.738668	18.04991	7.0292754	7.0172696
151.54819	185.38618	296.3513	180.42047	9.401504	8.979126
4.9999995	4.9999995	11.623892	7.4346223	4.704103	5.4854183
4.9999995	4.9999995	11.623892	7.4346223	4.704103	5.4854183
37.895287	145.7041	20.29875	73.09723	7.1141315	6.887596
16.783693	17.120913	11.28691	17.45658	4.919989	4.538577
16.783693	17.120913	11.28691	17.45658	4.919989	4.538577
16.783693	17.120913	11.28691	17.45658	4.919989	4.538577
16.783693	17.120913	11.28691	17.45658	4.919989	4.538577
8.273516	14.510448	17.024668	11.959251	4.7156153	4.76087
7.0154014	6.0013404	9.358593	6.384194	4.3509693	4.158482
7.0154014	6.0013404	9.358593	6.384194	4.3509693	4.158482
7.0154014	6.0013404	9.358593	6.384194	4.3509693	4.158482
7.0154014	6.0013404	9.358593	6.384194	4.3509693	4.158482
66.94008	165.30501	121.539986	107.11833	8.824776	9.204719
24.816164	4.9999995	4.9999995	4.9999995	6.691802	7.100487
69.68085	22.888197	71.08402	36.00542	6.9719443	6.5682406
39.49484	26.710447	30.437807	29.044147	6.7888656	6.6255684
197.61926	56.334175	79.6353	69.42572	7.9391212	8.142352
4.9999995	4.9999995	4.9999995	4.9999995	7.3681536	7.3098707
12.464051	33.670815	11.755082	18.22693	5.015324	5.063548
22.512053	30.585062	15.50941	16.828169	8.161489	8.5139675
3098.444	5478.6484	7605.865	5012.4653	14.384752	14.806293
275.72644	84.080376	172.86879	117.3152	9.413595	10.004086
236.00291	241.71207	195.28372	193.01369	9.115931	9.307951
293.65356	79.99764	201.1154	128.47198	9.341197	8.629734
348.74222	122.95122	340.82437	193.9638	9.491922	9.482655
113.08011	177.00948	234.39702	171.76878	8.603131	9.110519
43.639442	95.66652	85.45069	69.2212	9.19311	9.269911
49.087208	11.386268	13.828319	13.39596	6.2512703	6.36874
12.28965	15.13874	7.170188	10.23576	4.7632113	4.44475
21.486553	10.827091	19.475693	10.75051	6.2149935	6.997098
21.486553	10.827091	19.475693	10.75051	6.2149935	6.997098
20.054941	35.26596	36.876625	33.74478	5.9390764	6.091457
8.941907	12.37382	5.1232853	7.7242684	4.98413	4.811493
213.22006	61.691223	309.5748	149.57036	8.72662	8.875066
213.22006	61.691223	309.5748	149.57036	8.72662	8.875066
213.22006	61.691223	309.5748	149.57036	8.72662	8.875066
236.62776	692.928	430.97977	447.63382	10.383313	10.293598
24.059645	9.384424	8.854726	6.2090087	5.1868286	5.355707

8.6294	9.00169	10.051112	9.732162	7.669079	7.4807715
8.6294	9.00169	10.051112	9.732162	7.669079	7.4807715
8.6294	9.00169	10.051112	9.732162	7.669079	7.4807715
4.750881	5.655074	5.6615515	5.5429316	4.14586	3.2630506
6.16616	6.5722437	6.098575	6.3181477	5.322156	5.3804464
8.851934	8.692	9.657539	9.315685	7.38026	7.9070816
8.851934	8.692	9.657539	9.315685	7.38026	7.9070816
8.851934	8.692	9.657539	9.315685	7.38026	7.9070816
4.2223215	3.6814904	3.7943544	3.9240603	3.6494975	2.4279919
4.2223215	3.6814904	3.7943544	3.9240603	3.6494975	2.4279919
4.2223215	3.6814904	3.7943544	3.9240603	3.6494975	2.4279919
4.2223215	3.6814904	3.7943544	3.9240603	3.6494975	2.4279919
4.2223215	3.6814904	3.7943544	3.9240603	3.6494975	2.4279919
7.4507546	6.662826	5.9182405	6.3805285	4.4951386	3.5783641
9.026353	8.332553	9.941084	9.445841	6.9828115	7.3985543
4.5695276	4.232088	4.355845	4.3724475	4.1492453	2.4279919
5.9232073	4.172698	4.992227	4.7813497	3.6352596	3.738757
4.972652	4.794572	5.289053	5.4177947	3.9460435	3.046911
5.98077	6.406177	6.0947323	6.403911	3.4395602	3.6462574
5.98077	6.406177	6.0947323	6.403911	3.4395602	3.6462574
5.98077	6.406177	6.0947323	6.403911	3.4395602	3.6462574
5.98077	6.406177	6.0947323	6.403911	3.4395602	3.6462574
7.2993307	7.1796627	5.8233943	6.586777	5.690164	5.393093
9.717943	9.010224	9.134651	9.093991	7.9051075	8.255904
4.1291914	6.403911	6.1881447	6.3727245	2.3467326	2.4279919
4.1291914	6.403911	6.1881447	6.3727245	2.3467326	2.4279919
7.417124	8.826271	6.5174766	8.060068	6.005711	7.226442
5.4603024	5.132815	5.086352	5.225484	3.4756367	3.4159937
5.4603024	5.132815	5.086352	5.225484	3.4756367	3.4159937
5.4603024	5.132815	5.086352	5.225484	3.4756367	3.4159937
5.4603024	5.132815	5.086352	5.225484	3.4756367	3.4159937
4.846171	5.7595925	4.254867	5.0583897	3.678304	3.6350408
4.3659554	4.718999	4.2855873	3.7372835	2.3467326	2.4279919
4.3659554	4.718999	4.2855873	3.7372835	2.3467326	2.4279919
4.3659554	4.718999	4.2855873	3.7372835	2.3467326	2.4279919
4.3659554	4.718999	4.2855873	3.7372835	2.3467326	2.4279919
8.6130705	8.72496	7.676522	8.127468	6.8944926	7.783832
4.1176867	2.3221264	5.732545	4.3254886	3.5187483	3.0749142
7.4829626	7.5753284	6.911847	7.2927866	5.9924417	5.829446
6.576539	7.06807	6.2822256	6.6216736	5.0079174	5.8572493
7.351408	6.9764214	8.4162855	7.7451296	7.050524	6.704183
7.6002307	6.520251	6.7122445	6.6396914	2.3467326	3.6077442
5.716176	5.953912	4.945983	5.3726315	2.9779243	4.9725666
8.086385	7.1965575	7.108122	7.2170854	5.7347116	6.9892874
13.705409	13.49674	14.450358	14.342814	12.638865	13.501165
8.103829	7.2424264	9.231528	8.419092	7.9522905	7.930527
9.045032	9.744242	9.121104	9.232148	8.144797	8.576431
9.9908	9.884054	8.79155	9.453207	7.5420322	5.9904175
9.645759	9.510329	9.331725	9.490204	8.079763	7.9627295
8.20824	8.420494	9.024453	8.894337	7.391492	8.087527
9.145623	6.225221	9.291616	8.72701	6.578766	7.724579
6.2827773	5.8923397	4.97458	5.5087557	5.5217867	6.0602655
5.3929477	5.3539352	4.6233177	5.126404	3.2950459	2.4279919
5.275046	6.0863614	5.2280498	5.795545	4.6443276	4.7539115
5.275046	6.0863614	5.2280498	5.795545	4.6443276	4.7539115
6.9811416	7.4099135	5.8207946	6.3226867	4.83883	4.5006766
4.9894166	4.4136496	3.9031775	3.5024354	3.7041554	3.5856655
8.3844185	8.868647	8.681088	8.798042	7.209708	6.666758
8.3844185	8.868647	8.681088	8.798042	7.209708	6.666758
8.3844185	8.868647	8.681088	8.798042	7.209708	6.666758
10.576313	11.000381	10.452557	10.687448	8.229517	8.821267
4.6830854	4.3235664	4.713121	4.7120953	3.889101	2.4279919

8.033613	7.9186974	7.6462	7.676734
8.033613	7.9186974	7.6462	7.676734
8.033613	7.9186974	7.6462	7.676734
3.6318192	4.5560927	4.3310704	4.4544735
5.242817	5.2697606	5.974097	5.593424
7.4534135	7.340494	8.085354	7.810115
7.4534135	7.340494	8.085354	7.810115
7.4534135	7.340494	8.085354	7.810115
4.1281595	2.3408759	2.3618152	2.352752
4.1281595	2.3408759	2.3618152	2.352752
4.1281595	2.3408759	2.3618152	2.352752
4.1281595	2.3408759	2.3618152	2.352752
4.1281595	2.3408759	2.3618152	2.352752
4.5395336	4.26018	5.306449	4.5460353
7.096438	7.175758	7.609906	7.453815
3.60552	2.6047366	3.8099597	3.8398104
3.8319757	4.453799	4.6262693	4.2632356
4.806872	3.941811	4.092065	3.3315463
4.5822473	4.737487	3.9410737	3.7783108
4.5822473	4.737487	3.9410737	3.7783108
4.5822473	4.737487	3.9410737	3.7783108
4.5822473	4.737487	3.9410737	3.7783108
6.0968275	4.987355	4.566816	4.498391
7.1924505	7.9274664	8.460256	8.004814
2.350709	2.3408759	3.8129427	3.159215
2.350709	2.3408759	3.8129427	3.159215
5.2451262	7.5612764	4.6084595	6.6404076
4.1275835	4.2383866	3.7705467	4.4478803
4.1275835	4.2383866	3.7705467	4.4478803
4.1275835	4.2383866	3.7705467	4.4478803
4.1275835	4.2383866	3.7705467	4.4478803
3.179149	3.994277	4.3654075	3.881157
2.973409	2.6928782	3.512324	2.9358792
2.973409	2.6928782	3.512324	2.9358792
2.973409	2.6928782	3.512324	2.9358792
2.973409	2.6928782	3.512324	2.9358792
6.0446863	7.741766	7.172818	7.2102838
4.65747	2.3408759	2.3618152	2.352752
6.0970135	4.692999	6.416201	5.573363
5.3025317	4.9352064	5.2037377	5.2409825
7.577301	6.1009207	6.5737085	6.563906
2.350709	2.3408759	2.3618152	2.352752
3.7229505	5.29266	3.8302543	4.5137343
4.524444	5.144755	4.2267237	4.397705
11.567577	12.897419	13.197399	12.895115
8.053862	6.721661	7.673363	7.344508
7.829624	8.338986	7.855874	8.106773
8.1440735	6.647506	7.90175	7.4934654
8.391739	7.304121	8.649779	8.113877
6.784023	7.848084	8.127468	7.9292307
5.4444838	6.911137	6.6707797	6.5593195
5.616732	3.619529	4.061465	4.054602
3.7056875	4.060231	3.1436143	3.6476676
4.460566	3.5432389	4.548326	3.7191558
4.460566	3.5432389	4.548326	3.7191558
4.3694506	5.3709407	5.4783583	5.4734282
3.2799807	3.7454367	2.7081296	3.2190535
7.682559	6.243641	8.518262	7.716412
7.682559	6.243641	8.518262	7.716412
7.682559	6.243641	8.518262	7.716412
7.833492	9.902287	8.987645	9.351719
4.614075	3.3287625	3.4342847	2.8940525

ASHGA5P017159	0.01520177	0.075446109	2.6347925	up	noncoding
ASHGA5P038657	0.026565035	0.101808156	5.1135499	up	noncoding
ASHGA5P032483	0.005900029	0.048956663	2.1346575	up	noncoding
ASHGA5P038838	0.000828618	0.019333235	2.7711026	up	noncoding
ASHGA5P026915	0.001699714	0.027620998	2.3016162	up	noncoding
ASHGA5P026915	0.001699714	0.027620998	2.3016162	up	noncoding
ASHGA5P026915	0.001699714	0.027620998	2.3016162	up	noncoding
ASHGA5P026915	0.001699714	0.027620998	2.3016162	up	noncoding
ASHGA5P026915	0.001699714	0.027620998	2.3016162	up	noncoding
ASHGA5P031668	0.001151068	0.022812819	2.462606	up	noncoding
ASHGA5P032134	0.015293734	0.075720608	2.0894942	up	noncoding
ASHGA5P047634	0.006701	0.051962277	2.0412269	up	noncoding
ASHGA5P046199	0.0007151	0.01793864	3.7714179	up	noncoding
ASHGA5P017370	0.021683151	0.090884093	2.2897541	up	noncoding
ASHGA5P017370	0.021683151	0.090884093	2.2897541	up	noncoding
ASHGA5P017370	0.021683151	0.090884093	2.2897541	up	noncoding
ASHGA5P017370	0.021683151	0.090884093	2.2897541	up	noncoding
ASHGA5P014992	0.044795277	0.135994806	3.3783872	up	noncoding
ASHGA5P000271	0.011766668	0.066715537	2.4473843	up	noncoding
ASHGA5P054769	0.028117182	0.104710173	2.3004383	up	noncoding
ASHGA5P030988	2.91541E-05	0.005447654	2.2791799	up	noncoding
ASHGA5P027352	0.012207316	0.067658161	2.4657878	up	noncoding
ASHGA5P039087	0.02437846	0.09688833	2.4167738	up	noncoding
ASHGA5P038017	0.000393051	0.014144117	2.4924897	up	noncoding
ASHGA5P029242	0.024431281	0.096991067	2.361175	up	noncoding
ASHGA5P057023	0.000483715	0.015384974	5.9412375	up	noncoding
ASHGA5P029551	6.02919E-05	0.007803818	4.4538387	up	noncoding
ASHGA5P019000	2.48781E-05	0.005188131	3.3895	up	noncoding
ASHGA5P019000	2.48781E-05	0.005188131	3.3895	up	noncoding
ASHGA5P019000	2.48781E-05	0.005188131	3.3895	up	noncoding
ASHGA5P019000	2.48781E-05	0.005188131	3.3895	up	noncoding
ASHGA5P019000	2.48781E-05	0.005188131	3.3895	up	noncoding
ASHGA5P019000	2.48781E-05	0.005188131	3.3895	up	noncoding
ASHGA5P014166	0.028722461	0.105733109	2.0300714	up	noncoding
ASHGA5P033226	0.032718752	0.113900491	2.3884481	up	noncoding
ASHGA5P034452	0.035466338	0.119224479	2.0893114	up	noncoding
ASHGA5P044348	0.008311933	0.056954491	4.7109111	up	noncoding
ASHGA5P058434	1.80946E-05	0.004674623	2.5516587	up	noncoding
ASHGA5P058434	1.80946E-05	0.004674623	2.5516587	up	noncoding
ASHGA5P034071	0.011874396	0.066970246	3.3624137	up	noncoding
ASHGA5P030902	0.002426679	0.032019471	2.5359237	up	noncoding
ASHGA5P034904	0.026381124	0.101270986	2.1545402	up	noncoding
ASHGA5P038329	0.007447634	0.054185848	2.0428356	up	noncoding
ASHGA5P047581	0.000192978	0.01146874	5.4689966	up	noncoding
ASHGA5P056349	0.000274116	0.012323835	4.1561125	up	noncoding
ASHGA5P046546	0.000338719	0.013255256	2.4974954	up	noncoding
ASHGA5P046546	0.000338719	0.013255256	2.4974954	up	noncoding
ASHGA5P027636	1.61412E-05	0.004434727	4.3391317	up	noncoding
ASHGA5P044539	0.011456325	0.065908731	2.0109594	up	noncoding
ASHGA5P025486	0.02784078	0.104306505	2.0075714	up	noncoding
ASHGA5P025486	0.02784078	0.104306505	2.0075714	up	noncoding
ASHGA5P043861	0.00018162	0.011439647	4.4567715	up	noncoding
ASHGA5P053399	0.001679888	0.027439569	3.3847312	up	noncoding
ASHGA5P022247	0.046346584	0.138863255	5.029163	up	noncoding
ASHGA5P044310	2.31772E-06	0.00231943	2.976169	up	noncoding
ASHGA5P021016	0.000215596	0.01146874	3.0032892	up	noncoding
ASHGA5P028713	0.000750371	0.018465789	2.5690516	up	noncoding
ASHGA5P000115	0.000957441	0.021030899	2.1192907	up	noncoding
ASHGA5P048450	0.041195228	0.129607517	2.0274968	up	noncoding
ASHGA5P054995	0.000584616	0.016315159	2.7037052	up	noncoding
ASHGA5P018401	0.006014803	0.049415317	2.8220528	up	noncoding
ASHGA5P018401	0.006014803	0.049415317	2.8220528	up	noncoding

ENST00000438380	RP11-175D17.3	GENCODE	617	chr9	+
ENST00000453454	AC024560.3	GENCODE	956	chr3	-
ENST00000574741	RP11-156P1.3	GENCODE	836	chr17	-
NR_046572	ARHGEF3-AS1	RefSeq	500	chr3	+
BP305238		LincRNAs identified	583	chr7	-
BP305238		LincRNAs identified	583	chr7	-
BP305238		LincRNAs identified	583	chr7	-
BP305238		LincRNAs identified	583	chr7	-
BP305238		LincRNAs identified	583	chr7	-
ENST00000563540	RP11-146F11.5	GENCODE	482	chr16	+
BX478947		LincRNAs identified	685	chr1	-
ENST00000444359	RP11-316M21.6	GENCODE	846	chr10	+
TCONS_00002373	XLOC_000811	LincRNAs identified	364	chr1	-
NR_052024	EIF6	RefSeq	803	chr20	-
NR_052024	EIF6	RefSeq	803	chr20	-
NR_052024	EIF6	RefSeq	803	chr20	-
NR_052024	EIF6	RefSeq	803	chr20	-
ENST00000437191	AC005062.2	GENCODE	571	chr7	-
chr13:90287700-902	chr13:90287700-902	LincRNAs identified	11100	chr13	-
ENST00000525302	RP11-1L12.3	GENCODE	583	chr11	-
ENST00000568026	RP11-916L7.1	GENCODE	551	chr16	-
ENST00000445845	RP11-302M6.2	GENCODE	598	chr1	-
ENST00000498005	RP11-167H9.6	GENCODE	525	chr3	+
ENST00000420096	Z83851.4	GENCODE	544	chr22	+
ENST00000553507	RP11-488C13.6	GENCODE	262	chr14	-
TCONS_00012039	XLOC_005574	LincRNAs identified	498	chr6	-
ENST00000555689	RP11-589M4.1	GENCODE	528	chr14	+
NR_048535	WDR4	RefSeq	2060	chr21	-
NR_048535	WDR4	RefSeq	2060	chr21	-
NR_048535	WDR4	RefSeq	2060	chr21	-
NR_048535	WDR4	RefSeq	2060	chr21	-
NR_048535	WDR4	RefSeq	2060	chr21	-
NR_048535	WDR4	RefSeq	2060	chr21	-
ENST00000434729	SNHG11	GENCODE	1058	chr20	+
ENST00000512717	RP11-700H6.2	GENCODE	789	chr17	+
FN100697		LincRNAs identified	104	chr12	-
ENST00000523313	RP11-697M17.1	GENCODE	557	chr8	-
uc003myy.1	LOC100130275	UCSC_knowngene	1488	chr6	+
uc003myy.1	LOC100130275	UCSC_knowngene	1488	chr6	+
NR_038336	DDX39A	RefSeq	1651	chr19	-
ENST00000573130	RP11-473M20.7	GENCODE	2163	chr16	-
TCONS_00003580	XLOC_001356	LincRNAs identified	381	chr2	+
ENST00000494840	RP11-219E24.1	GENCODE	2595	chr3	-
uc001kgu.3	KRMP1	UCSC_knowngene	206	chr10	+
TCONS_00003617	XLOC_001406	LincRNAs identified	331	chr2	+
uc004dsb.1	AY927613	UCSC_knowngene	329	chrX	+
uc004dsb.1	AY927613	UCSC_knowngene	329	chrX	+
NR_038854	LOC100506668	RefSeq	1897	chr12	-
ENST00000523502	KB-1568E2.1	GENCODE	2072	chr8	-
NR_003103	POT1	RefSeq	4006	chr7	-
NR_003103	POT1	RefSeq	4006	chr7	-
TCONS_00018589	XLOC_008925	LincRNAs identified	447	chr10	-
ENST00000422008	GAS5	GENCODE	497	chr1	-
ENST00000553410	RP11-692C24.1	GENCODE	431	chr15	+
ENST00000502076	SNHG6	GENCODE	2578	chr8	-
ENST00000523427	PVT1	GENCODE	938	chr8	+
ENST00000431928	AL592284.1	GENCODE	1063	chr1	-
BF989096R		LincRNAs identified	353	chr18	-
AW076083		LincRNAs identified	433	chr4	+
ENST00000528684	FAM86C2P	pseudogene	1004	chr11	-
ENST00000453103	DGUOK-AS1	GENCODE	601	chr2	-
ENST00000453103	DGUOK-AS1	GENCODE	601	chr2	-

127532401	127535206		TTAACTCGAGCACTGATGAAGTCCA		intronic antisense
197305083	197342531		TCAATCTGGTGGTCTTTTGTTTCCAC		intergenic
45159285	45177605	ENST00000573341	CTGGGGGAGAGGAGACGCGTGTGT(		intergenic
56974067	56994880		CCCTTCTCCATTA	100874200	intronic antisense
135585497	135662101		TGAGCTCCAGTGCTGTGAATCTCTG(		exon sense-overlappi
135585497	135662101		TGAGCTCCAGTGCTGTGAATCTCTG(		exon sense-overlappi
135585497	135662101		TGAGCTCCAGTGCTGTGAATCTCTG(		exon sense-overlappi
135585497	135662101		TGAGCTCCAGTGCTGTGAATCTCTG(		exon sense-overlappi
135585497	135662101		TGAGCTCCAGTGCTGTGAATCTCTG(		intronic antisense
30597227	30619914		CTTCCTTCTGCTACTGAGGTGAGAA(		intronic antisense
111460165	111460747		AAGTTATAGATCTCAAACTAATCCC.		intergenic
101989423	101993757		CACAAAGAAGTCAAAATGGAAACT/		natural antisense
44404329	44406129		TTTGAAGGGAGCAAAGGCATCGAA/		intergenic
33866708	33872520	NM_001267810, NM	ACTTCTACAGGA(	3692	exon sense-overlappi
33866708	33872520	NM_001267810, NM	ACTTCTACAGGA(	3692	exon sense-overlappi
33866708	33872520	NM_001267810, NM	ACTTCTACAGGA(	3692	exon sense-overlappi
33866708	33872520	NM_001267810, NM	ACTTCTACAGGA(	3692	exon sense-overlappi
19970875	20180049	ENST00000415499	GAAAATGATAATGAGGAACATCGAT		exon sense-overlappi
91489699	91500799		TTTTTTCCTATCTTAAAGTTCATTTC		intergenic
27084529	27241660		TTTCTAGGTCATGCTACGGTTACCCT		intronic antisense
17634187	17634842		TTCTAAGGTCTTTCTAAGAAGGATT(		intergenic
90421224	90422281		TTCAAGGTTTAGTCAAGGCAAAGGA		intergenic
149956486	149957996		AACATTTCTGAAATCCTGTTCTTAGT		intergenic
42672360	42673057		CTGAGCGGCTTCACCATTCAAGTTT/		intronic antisense
77247050	77247799		CTACTTCAGGGATTGCAAACTGGCA		natural antisense
1308820	1311276		AGGTGGAGCTTGACCAAACACTTGT		intergenic
53243069	53243858		CTCAAATTGTTCAGTGGTAAGAAAG		natural antisense
44269335	44299699		TGGCACATGTTG/	10785	exon sense-overlappi
44269335	44299699		TGGCACATGTTG/	10785	exon sense-overlappi
44269335	44299699		TGGCACATGTTG/	10785	exon sense-overlappi
44269335	44299699		TGGCACATGTTG/	10785	exon sense-overlappi
44269335	44299699		TGGCACATGTTG/	10785	exon sense-overlappi
44269335	44299699		TGGCACATGTTG/	10785	exon sense-overlappi
37075399	37079564	ENST00000400436,	TATCCTTGAGCTT	128439	intergenic
49021474	49026111		GCACAAAAGGACCTTTCCTTTGACA'		intergenic
48010161	48010265		CCAGTCTGCAAACTCAGAAGAAATC		intergenic
76136132	76236551	ENST00000521147,	ACACATTTGCTGCTTCCATTCTAAA(		intergenic
10414299	10416190		TTGCTCGCCAGT/	100130275	intronic antisense
10414299	10416190		TTGCTCGCCAGT/	100130275	natural antisense
14519609	14529906		TGTCATGGCCTA(	10212	exon sense-overlappi
3103367	3106233		AAGGTCACTTTGTCCCTGGATGACC.		intronic antisense
13141014	13353671		AGATATTCTCTGAGACCGGGAACGT		intergenic
95373266	95402037		GAAAAGTATTTATTCTAATCTATTCT		intergenic
91492812	91493018		TATACATTTTCTAGGGATGGGCATA(		exon sense-overlappi
27975724	27980489		AGTATCTGATGAAGGAGGAAATGG/		intergenic
53221897	53223882		CAGGAGCTGAGGTCTGAACAATACC		natural antisense
53221897	53223882		CAGGAGCTGAGGTCTGAACAATACC		natural antisense
120928140	120933749		GTCACTCTTTAAT	100506668	intronic antisense
131607544	131667958		GATTTCTACCAATTATTGACAATTC/		intergenic
124462439	124570037		ATCACTATGGCA(	25913	exon sense-overlappi
124462439	124570037		ATCACTATGGCA(	25913	exon sense-overlappi
103461753	103464357		TTTCTCTGACCAGTGCTTGTTCTGCT		intergenic
173833621	173836168	ENST00000412059,	GCAAAAGGTTTT(	60674	intergenic
92076120	92078325		AAGACATTAAAAGCAATCTGTGAAC		intergenic
67834229	67837776		TGTGTACTCACT/	641638	natural antisense
128806820	128903198		TCCTTGTCATGAC	5820	intergenic
144479977	144481765	ENST00000417976	TGTTTGTGAAAATGGCATAAAGCCG		intronic antisense
46489320	46490311		GGACAGCTGGCTGAGATCAAGGATT		intergenic
37774187	37774619		GGAATCTACATTCTGGTGCAATGTT(		intergenic
67560666	67572784		AAGAGACTCATC.	645332	
74174768	74208568		GAACTCTCAGGAGGCTTTCCGGAAG		intronic antisense
74174768	74208568		GAACTCTCAGGAGGCTTTCCGGAAG		intronic antisense

NM_033334	NR6A1	nuclear receptor subf	-	127284702	127533576
NM_001128615	ARHGEF3	rho guanine nucleotic	-	56761445	57113336
ENST00000393085	MTPN	myotrophin [Source:]	-	135611508	135662064
ENST00000435723	MTPN	myotrophin [Source:]	-	135614206	135662101
NM_001128619	LUZP6	leucine zipper protei	-	135611502	135662204
NM_145808	MTPN	myotrophin	-	135611502	135662204
ENST00000416501	AC015987.2		+	135611541	135613214
NM_138447	ZNF689	zinc finger protein 68	-	30614699	30621682
NM_018294	CWF19L1	CWF19-like protein	-	101992054	102027437
NM_001267810	EIF6	eukaryotic translatior	-	33866708	33872520
NM_002212	EIF6	eukaryotic translatior	-	33866708	33872619
NM_181466	EIF6	eukaryotic translatior	-	33866708	33872619
NM_181468	EIF6	eukaryotic translatior	-	33866708	33872520
NM_182762	MACC1	metastasis-associated	-	20174278	20257013
NM_003986	BBOX1	gamma-butyrobetain	+	27062508	27149354
ENST00000515426	TCF20	transcription factor 2	-	42610852	42739622
NM_014909	VASH1	vasohibin-1	+	77228234	77249363
NM_198066	GNPNAT1	glucosamine 6-phosp	-	53241910	53258386
NM_001260474	WDR4	tRNA (guanine-N(7)	-	44269335	44299693
NM_001260475	WDR4	tRNA (guanine-N(7)	-	44269335	44299693
NM_001260476	WDR4	tRNA (guanine-N(7)	-	44269335	44299699
NM_001260477	WDR4	tRNA (guanine-N(7)	-	44269335	44299385
NM_018669	WDR4	tRNA (guanine-N(7)	-	44269335	44299693
NM_033661	WDR4	tRNA (guanine-N(7)	-	44263189	44299693
NM_001042425	TFAP2A	transcription factor A	-	10396915	10419797
NM_003220	TFAP2A	transcription factor A	-	10396915	10415470
NM_005804	DDX39A	ATP-dependent RNA	-	14519609	14530195
NM_022468	MMP25	matrix metalloprotei	+	3096681	3110724
NM_016195	KIF20B	kinesin-like protein K	+	91461366	91534700
NM_001146702	KDM5C	lysine-specific demet	-	53220502	53254604
NM_004187	KDM5C	lysine-specific demet	-	53220502	53254604
NM_001037494	DYNLL1	dynein light chain 1,	+	120907659	120936298
NM_001042594	POT1	protection of telomer	-	124462439	124570037
NM_015450	POT1	protection of telomer	-	124462439	124570037
NM_173518	MCMDC2	MCM domain-contai	+	67782983	67834283
ENST00000538264	AL592284.2	Uncharacterized prot	+	144339737	144521058
NM_080916	DGUOK	deoxyguanosine kina	+	74153952	74186088
NM_080918	DGUOK	deoxyguanosine kina	+	74153952	74186088

36.517909	11.027871	4.829067	3.431378	50.213932	44.42035
38028.6205	8847.991383	14.553849	12.199524	38255.336	43764.42
165.828363	53.200285	6.996141	5.902136	182.91498	200.21996
109.877235	26.365919	6.365793	4.895333	137.20523	151.5278
75.158943	18.878581	5.695313	4.492666	52.28314	70.67467
75.158943	18.878581	5.695313	4.492666	52.28314	70.67467
75.158943	18.878581	5.695313	4.492666	52.28314	70.67467
75.158943	18.878581	5.695313	4.492666	52.28314	70.67467
75.158943	18.878581	5.695313	4.492666	52.28314	70.67467
22.239648	6.478617	4.129598	2.829412	24.159523	17.455408
190.58094	62.357304	7.203342	6.140189	194.30234	192.76855
108.690326	37.066932	6.400391	5.370954	138.85121	118.57732
55.288977	11.087623	5.424041	3.508934	45.50894	49.06439
3516.91265	960.492633	11.334443	10.13925	4005.0212	4279.475
3516.91265	960.492633	11.334443	10.13925	4005.0212	4279.475
3516.91265	960.492633	11.334443	10.13925	4005.0212	4279.475
3516.91265	960.492633	11.334443	10.13925	4005.0212	4279.475
38.123526	7.865309	4.691104	2.934769	29.309525	46.044235
86.461473	25.621681	6.055446	4.764205	80.65855	51.40939
410.512652	94.971952	8.081954	6.880045	551.927	188.41696
164.808128	44.493543	6.950131	5.761616	208.20761	212.98756
27.449007	8.01252	4.42486	3.122812	27.53253	28.44098
56.86262	16.587065	5.471962	4.198879	53.93308	58.96889
385.858282	101.635782	8.207799	6.890211	407.8754	358.24915
27.712391	9.730197	4.448957	3.209452	25.904833	26.33019
46.131967	5.256773	5.119475	2.548712	41.941734	24.666086
58.826703	8.670647	5.523288	3.368238	53.431347	57.45349
149.720198	26.226694	6.777698	5.016625	192.12999	238.41035
149.720198	26.226694	6.777698	5.016625	192.12999	238.41035
149.720198	26.226694	6.777698	5.016625	192.12999	238.41035
149.720198	26.226694	6.777698	5.016625	192.12999	238.41035
149.720198	26.226694	6.777698	5.016625	192.12999	238.41035
149.720198	26.226694	6.777698	5.016625	192.12999	238.41035
115.581021	36.498973	6.441281	5.419751	144.95808	174.89087
73.57265	23.110608	5.843674	4.5876	79.6173	81.19433
55.665959	15.243555	5.261541	4.198513	31.487284	27.704473
729.380272	159.891451	8.859231	6.623225	784.83276	133.6462
892.564017	220.75601	9.365237	8.013802	1048.5907	1056.1902
892.564017	220.75601	9.365237	8.013802	1048.5907	1056.1902
41.340898	8.64206	4.921775	3.172277	54.35279	72.93795
25.498198	7.663424	4.35912	3.016609	25.089148	23.232601
869.048535	288.443903	9.359294	8.251914	932.87225	1010.513
299.721498	97.720762	7.837011	6.806438	335.17752	375.20038
59.894256	7.081966	5.503812	3.052536	72.3442	69.4562
294.120795	45.113725	7.760419	5.705185	297.53314	396.27017
39.906585	10.912959	4.925421	3.604939	41.78216	43.314613
39.906585	10.912959	4.925421	3.604939	41.78216	43.314613
49.540046	7.391295	5.277865	3.160459	52.078835	61.75961
27.914229	9.220725	4.401413	3.39353	40.047527	39.157787
478.358562	141.998762	8.425364	7.419913	425.7385	372.95856
478.358562	141.998762	8.425364	7.419913	425.7385	372.95856
50.776144	8.666706	5.179285	3.023286	72.99826	27.509535
10158.19638	1667.06735	12.812007	11.052966	8714.625	7460.4478
111.816179	8.473086	5.482886	3.152568	168.50676	8.719618
213.481817	45.405621	7.367553	5.794096	191.2054	158.48163
79.926809	17.062318	5.925593	4.33905	67.782166	58.758404
244.415837	61.225368	7.489521	6.128285	289.53333	144.32426
144.63131	41.838499	6.762155	5.678574	163.5572	176.6897
1670.363983	479.097927	10.161339	9.141639	2161.004	2851.1426
758.148362	161.8472	9.107697	7.672759	796.947	1049.9551
62.501353	18.678645	5.457295	3.96055	58.841583	20.832794
62.501353	18.678645	5.457295	3.96055	58.841583	20.832794

31.884237	27.603092	28.783342	36.202503	12.65942	4.9999995
25277.338	7790.621	59308.5	53775.508	11848.081	1408.6954
149.12431	114.08172	165.72076	182.90845	68.33108	27.696022
82.181	64.82123	123.87859	99.64956	32.835926	19.614122
36.729267	162.16516	36.86717	92.23425	18.822016	14.213259
36.729267	162.16516	36.86717	92.23425	18.822016	14.213259
36.729267	162.16516	36.86717	92.23425	18.822016	14.213259
36.729267	162.16516	36.86717	92.23425	18.822016	14.213259
36.729267	162.16516	36.86717	92.23425	18.822016	14.213259
20.313232	28.51042	13.43198	29.567327	5.6349072	4.9999995
156.0195	155.69409	250.66527	194.03589	76.44213	43.23573
106.1045	91.63792	87.317635	109.65337	44.08147	17.606152
46.428574	48.567047	72.8429	69.32201	9.219546	4.9999995
3902.9243	3185.7046	2329.532	3398.8188	701.3255	356.5995
3902.9243	3185.7046	2329.532	3398.8188	701.3255	356.5995
3902.9243	3185.7046	2329.532	3398.8188	701.3255	356.5995
3902.9243	3185.7046	2329.532	3398.8188	701.3255	356.5995
12.217701	79.62133	19.190996	42.35737	11.982269	4.9999995
95.426765	77.3894	99.21093	114.673805	31.993284	14.739149
779.9464	144.18208	432.30997	366.2935	97.987495	59.433784
126.7063	100.138176	199.80144	141.00768	44.647644	42.1095
29.782959	41.67176	20.89999	16.365824	6.5368714	4.9999995
39.931362	77.30818	55.45215	55.58206	21.067057	9.786456
452.0469	376.53586	303.87808	416.5643	113.3904	44.972183
29.853022	19.66019	31.671392	32.85472	13.249699	4.9999995
77.228546	35.030296	51.96587	45.95927	4.9999995	5.2650576
47.003788	69.66468	48.92978	76.477135	11.427549	5.2589593
90.89817	88.679756	138.09329	150.10963	27.92788	28.188229
90.89817	88.679756	138.09329	150.10963	27.92788	28.188229
90.89817	88.679756	138.09329	150.10963	27.92788	28.188229
90.89817	88.679756	138.09329	150.10963	27.92788	28.188229
90.89817	88.679756	138.09329	150.10963	27.92788	28.188229
90.89817	88.679756	138.09329	150.10963	27.92788	28.188229
108.527596	103.62409	69.93092	91.55457	36.49153	21.16506
59.64551	90.65832	53.938393	76.38205	20.6603	7.9891624
27.806787	54.806084	99.124275	93.06685	13.514861	14.970489
1402.4442	725.71857	571.5165	758.1234	245.52382	13.51573
1191.7118	626.5689	637.2808	795.0417	287.3929	140.1228
1191.7118	626.5689	637.2808	795.0417	287.3929	140.1228
24.597511	28.175179	35.462162	32.519794	8.861241	4.9999995
25.79767	28.226274	21.555386	29.08811	10.560051	4.9999995
677.64484	693.15015	969.57477	930.5362	315.02576	108.85429
283.32867	313.93915	200.6446	290.03867	101.69148	42.22363
68.99655	30.710285	59.831505	58.026794	4.9999995	5.845311
196.22389	391.56207	155.91284	327.22266	60.528625	36.143612
32.95827	22.263731	50.095108	49.025627	8.049293	6.690165
32.95827	22.263731	50.095108	49.025627	8.049293	6.690165
44.776524	40.62386	42.715252	55.286198	9.637973	5.3204327
21.546732	17.447016	21.81184	27.47447	10.294519	5.828338
451.2593	809.8626	235.10191	575.2305	107.01641	106.036415
451.2593	809.8626	235.10191	575.2305	107.01641	106.036415
98.29166	47.659527	27.565775	30.632105	6.8198495	4.9999995
9647.771	15892.931	6521.8315	12711.572	1222.9513	1179.694
334.307	98.92223	9.653068	50.7884	4.9999995	4.9999995
218.19273	228.77661	212.44563	271.7889	41.00309	26.24553
69.359825	126.55479	55.514862	101.590805	12.376399	10.939619
395.51437	159.53087	220.50192	257.09027	86.85609	44.7918
91.37902	214.4745	96.29282	125.39462	36.616653	34.169502
994.8761	748.2565	1678.7437	1588.161	588.53796	266.15057
439.04993	479.79324	872.3092	910.8357	197.82864	189.30185
103.44256	36.379597	76.662766	78.84882	9.614739	4.9999995
103.44256	36.379597	76.662766	78.84882	9.614739	4.9999995

24.059645	9.384424	8.854726	6.2090087	5.1868286	5.355707
34606.75	897.6724	2957.1626	1369.5869	14.813899	15.086061
103.483086	41.56991	46.73006	31.39155	7.058047	7.395783
44.397144	11.789611	28.74348	20.815231	6.646385	7.0079236
18.240353	32.136124	13.87174	15.987992	5.2416797	5.9647217
18.240353	32.136124	13.87174	15.987992	5.2416797	5.9647217
18.240353	32.136124	13.87174	15.987992	5.2416797	5.9647217
18.240353	32.136124	13.87174	15.987992	5.2416797	5.9647217
18.240353	32.136124	13.87174	15.987992	5.2416797	5.9647217
4.9999995	6.6614313	7.4073906	9.167972	4.1375227	4.1276827
127.37906	38.66057	39.865902	48.560432	7.1538815	7.3475327
70.58043	37.00782	19.307259	33.818462	6.66297	6.665085
8.735777	11.84449	22.189453	9.536472	5.0491295	5.4860024
1136.6143	697.5421	1795.1074	1075.767	11.4803095	11.631184
1136.6143	697.5421	1795.1074	1075.767	11.4803095	11.631184
1136.6143	697.5421	1795.1074	1075.767	11.4803095	11.631184
1136.6143	697.5421	1795.1074	1075.767	11.4803095	11.631184
15.17375	4.9999995	5.0358386	4.9999995	4.417604	5.4014916
50.49116	9.612575	16.861708	30.03221	5.8804207	5.548712
126.26612	111.07319	86.173706	88.897415	8.6234255	7.3159294
53.083908	30.86961	60.83764	35.412956	7.2483916	7.4795403
10.405619	9.258637	5.655503	11.218488	4.3302937	4.771877
25.945261	9.158736	9.525462	24.039415	5.2860045	5.7211576
170.43253	74.16534	119.30967	87.54457	8.211768	8.196291
20.512966	7.808945	6.809575	4.9999995	4.2370005	4.6662374
4.9999995	6.2755823	4.9999995	4.9999995	4.928829	4.580166
9.089143	8.8801565	9.457175	7.910899	5.2723403	5.688736
21.86391	24.809185	32.406746	22.164217	7.1350074	7.6345825
21.86391	24.809185	32.406746	22.164217	7.1350074	7.6345825
21.86391	24.809185	32.406746	22.164217	7.1350074	7.6345825
21.86391	24.809185	32.406746	22.164217	7.1350074	7.6345825
21.86391	24.809185	32.406746	22.164217	7.1350074	7.6345825
21.86391	24.809185	32.406746	22.164217	7.1350074	7.6345825
40.455112	23.42127	60.983818	36.47705	6.725789	7.2087994
38.047436	11.154431	40.00361	20.80871	5.862277	6.1488204
18.662289	12.350169	19.827824	12.135699	4.518054	4.7364674
507.73712	22.61464	111.32112	58.636276	9.157744	6.8307967
423.81927	149.09386	177.4755	146.63173	9.561833	9.6990385
423.81927	149.09386	177.4755	146.63173	9.561833	9.6990385
16.86522	7.3520575	6.3760386	7.3978043	5.2983294	6.0050597
9.083032	9.447727	4.9999995	6.8897367	4.189369	4.4962845
732.9415	164.4944	200.15982	209.18765	9.397005	9.637304
194.65723	86.640274	86.05499	75.05697	7.935408	8.258741
8.579306	7.6300945	8.544928	6.8921556	5.7182283	5.943034
72.588936	50.48689	22.370333	28.563957	7.7627907	8.333413
11.62681	6.049648	20.381628	12.680209	4.922166	5.323417
11.62681	6.049648	20.381628	12.680209	4.922166	5.323417
6.2192707	6.866813	8.12209	8.181189	5.2367263	5.7849503
16.419401	6.33314	6.903862	9.545087	4.8627152	5.1884604
81.423294	169.56767	145.26337	242.68541	8.273193	8.250728
81.423294	169.56767	145.26337	242.68541	8.273193	8.250728
15.412652	14.767738	4.9999995	4.9999995	5.7331724	4.7281084
1235.7389	1866.7906	2600.5046	1896.7247	12.594571	12.404257
7.195631	12.332378	10.71527	10.595239	6.9406652	3.2312198
53.66518	45.421677	59.90761	46.19064	7.1282496	7.068897
13.677021	24.38367	22.738592	18.25861	5.622878	5.7158804
112.20957	40.379276	44.475307	38.640167	7.72486	6.940894
44.124737	63.18168	26.877522	46.0609	6.8956866	7.2234597
960.9075	388.87683	344.3622	325.7525	10.600697	11.061976
177.09843	144.00763	140.67236	122.17429	9.1765995	9.690059
18.759052	6.0033064	46.835297	25.859478	5.4185853	4.3497925
18.759052	6.0033064	46.835297	25.859478	5.4185853	4.3497925

4.6830854	4.3235664	4.713121	4.7120953	3.889101	2.4279919
14.364794	12.583225	15.402314	15.072802	13.790269	11.337718
6.8923244	6.497556	7.1448784	6.9882574	6.3292575	5.62692
6.0431924	5.646363	6.732023	6.118871	5.2683916	5.1202636
4.884555	7.0202956	5.050795	6.00983	4.4688506	4.6402507
4.884555	7.0202956	5.050795	6.00983	4.4688506	4.6402507
4.884555	7.0202956	5.050795	6.00983	4.4688506	4.6402507
4.884555	7.0202956	5.050795	6.00983	4.4688506	4.6402507
4.884555	7.0202956	5.050795	6.00983	4.4688506	4.6402507
4.025434	4.3724475	3.6813183	4.4331803	2.6973226	2.4279919
6.950888	6.9601846	7.7302284	7.077339	6.4801035	6.280188
6.411529	6.164458	6.2418895	6.2564144	5.695669	4.961122
5.2159524	5.2072406	5.982767	5.6031547	3.4181259	2.4279919
11.564221	11.305792	10.908756	11.116395	9.747459	9.353385
11.564221	11.305792	10.908756	11.116395	9.747459	9.353385
11.564221	11.305792	10.908756	11.116395	9.747459	9.353385
11.564221	11.305792	10.908756	11.116395	9.747459	9.353385
3.2825963	5.9547	4.1654124	4.924819	3.8130364	2.4279919
6.253614	5.91164	6.419143	6.319147	5.2302775	4.6919217
9.259303	6.845393	8.498161	7.949512	6.847015	6.7450323
6.6614027	6.294335	7.4070687	6.6100464	5.711854	6.240376
4.5876474	4.9668937	4.2813272	3.6111238	2.9043539	2.4279919
5.0021043	5.9102693	5.607611	5.304624	4.635839	4.0720043
8.470838	8.236581	7.998141	8.133172	7.060309	6.336935
4.591385	3.7738929	4.8423557	4.5828695	3.9553974	2.4279919
5.955542	4.6950574	5.520516	5.0367393	2.3467326	3.1275885
5.236319	5.755562	5.4393625	5.747406	3.745138	3.1256516
6.188476	6.116856	6.8886895	6.7025757	5.0398574	5.6542716
6.188476	6.116856	6.8886895	6.7025757	5.0398574	5.6542716
6.188476	6.116856	6.8886895	6.7025757	5.0398574	5.6542716
6.188476	6.116856	6.8886895	6.7025757	5.0398574	5.6542716
6.188476	6.116856	6.8886895	6.7025757	5.0398574	5.6542716
6.188476	6.116856	6.8886895	6.7025757	5.0398574	5.6542716
6.4430714	6.3451195	5.926499	5.998408	5.42693	5.235102
5.5864143	6.1493278	5.5699444	5.7452593	4.6066203	3.7616313
4.487645	5.38837	6.417212	6.0214953	3.984831	4.7162933
10.083012	9.197691	8.900707	8.985438	8.199003	4.558445
9.851664	8.975401	9.046899	9.056586	8.427569	7.977901
9.851664	8.975401	9.046899	9.056586	8.427569	7.977901
4.3102117	4.3526316	4.9957995	4.568616	3.3616679	2.4279919
4.3783617	4.3568316	4.326484	4.40739	3.6274884	2.4279919
9.042079	9.131728	9.66805	9.2796	8.572605	7.6110425
7.8095045	7.9720025	7.412202	7.634206	6.906183	6.2459517
5.7977057	4.4931965	5.7136416	5.357065	2.3467326	3.2896845
7.3008018	8.29456	7.065579	7.805371	6.1544623	6.0230484
4.7304535	3.978674	5.4707227	5.127093	3.2106555	3.5022998
4.7304535	3.978674	5.4707227	5.127093	3.2106555	3.5022998
5.164958	4.9255667	5.258837	5.2961526	3.4912353	3.1448622
4.1108203	3.5801945	4.342724	4.3235664	3.589954	3.2834003
8.467389	9.346241	7.63198	8.582654	6.9784765	7.574872
8.467389	9.346241	7.63198	8.582654	6.9784765	7.574872
6.29789	5.176109	4.655272	4.485157	2.967167	2.4279919
12.909136	13.621698	12.339968	13.002414	10.5570965	11.081052
8.050286	6.2779746	3.2233045	5.1738677	2.3467326	2.4279919
7.4483156	7.5330043	7.489253	7.5375977	5.593311	5.549306
5.8056364	6.652924	5.609582	6.146659	3.8587291	4.24169
8.277728	6.9980917	7.5387526	7.456801	6.669389	6.32963
6.1961746	7.4336886	6.377026	6.446896	5.429809	5.9349227
9.586453	9.240418	10.42822	10.05027	9.475051	8.921411
8.429304	8.591605	9.513567	9.245046	7.8730164	8.420225
6.3765316	4.7532887	6.056242	5.789332	3.48692	2.4279919
6.3765316	4.7532887	6.056242	5.789332	3.48692	2.4279919

4.614075	3.3287625	3.4342847	2.8940525
14.999971	10.283728	11.781255	11.004203
6.6566997	5.616732	5.817911	5.3652983
5.4698343	3.6708906	5.1222267	4.7203903
4.244282	5.217492	4.0659833	4.3191347
4.244282	5.217492	4.0659833	4.3191347
4.244282	5.217492	4.0659833	4.3191347
4.244282	5.217492	4.0659833	4.3191347
4.244282	5.217492	4.0659833	4.3191347
2.350709	2.836006	3.1866665	3.4777744
6.952528	5.5088496	5.591009	6.0284534
6.1118073	5.4427023	4.536811	5.477615
3.250031	3.6758711	4.743839	3.5377455
10.091462	9.91113	11.0612545	10.670811
10.091462	9.91113	11.0612545	10.670811
10.091462	9.91113	11.0612545	10.670811
10.091462	9.91113	11.0612545	10.670811
3.9885035	2.3408759	2.6854556	2.352752
5.652783	3.3599436	4.351824	5.298483
6.940233	7.142674	6.6834564	6.9218607
5.720479	5.15769	6.191215	5.548082
3.4836776	3.3069425	2.8312874	3.7826176
4.721429	3.2886584	3.5371802	4.938165
7.3643236	6.5330515	7.1467557	6.899891
4.3993173	3.0522783	3.0689743	2.352752
2.350709	2.7526717	2.3618152	2.352752
3.302375	3.2498379	3.5294843	3.2569432
4.4832006	4.819709	5.2895956	4.813118
4.4832006	4.819709	5.2895956	4.813118
4.4832006	4.819709	5.2895956	4.813118
4.4832006	4.819709	5.2895956	4.813118
4.4832006	4.819709	5.2895956	4.813118
4.4832006	4.819709	5.2895956	4.813118
5.336752	4.7317834	6.1956625	5.5922737
5.2512145	3.59052	5.596192	4.719424
4.271352	3.7420893	4.5733356	3.9031775
8.9419365	4.6727796	7.05524	6.311948
8.679562	7.592475	7.7155695	7.6897326
8.679562	7.592475	7.7155695	7.6897326
4.1332693	2.9704218	2.9874654	3.1528482
3.3015637	3.33721	2.3618152	3.0435836
9.46278	7.7353005	7.89518	8.234578
7.5555167	6.768513	6.681616	6.680845
3.2253118	3.0211706	3.3883796	3.043935
6.155937	5.9305	4.7562895	5.21087
3.6326103	2.7024727	4.615554	3.9660418
3.6326103	2.7024727	4.615554	3.9660418
2.8294182	2.8759942	3.314162	3.3070807
4.095331	2.7642784	3.088338	3.5398755
6.314666	7.7786613	7.4152036	8.457598
6.314666	7.7786613	7.4152036	8.457598
4.0096884	4.0203004	2.3618152	2.352752
10.215148	11.372682	11.593684	11.498134
3.0028353	3.7401023	3.7005663	3.6971796
5.738274	5.759463	6.171242	5.9529824
3.8473296	4.7915974	4.7794514	4.515502
6.772745	5.5724835	5.7432103	5.6822543
5.461342	6.280188	5.0173407	5.94784
9.846637	9.055908	8.662384	8.888445
7.4206467	7.543465	7.3708143	7.408386
4.2789593	2.6937456	5.822558	5.0531273
4.2789593	2.6937456	5.822558	5.0531273

ASHGA5P044962	0.00341991	0.037690616	2.2904215	up	noncoding
ASHGA5P032707	0.028761723	0.10581013	2.8365354	up	noncoding
ASHGA5P037126	0.000657628	0.017168766	2.7387288	up	noncoding
ASHGA5P041019	0.00164009	0.02720749	2.0507916	up	noncoding
ASHGA5P028248	0.000196484	0.01146874	3.5595812	up	noncoding
ASHGA5P037045	0.012452022	0.068356958	2.2543206	up	noncoding
ASHGA5P039452	0.008893146	0.058842304	6.029481	up	noncoding
ASHGA5P035635	0.000562929	0.016101946	2.4726924	up	noncoding
ASHGA5P022539	0.001255143	0.02370642	2.4671059	up	noncoding
ASHGA5P039047	0.001215898	0.023487681	2.4848536	up	noncoding
ASHGA5P039047	0.001215898	0.023487681	2.4848536	up	noncoding
ASHGA5P021511	0.000571707	0.016151364	2.1750195	up	noncoding
ASHGA5P036334	0.015464833	0.076002496	2.8048465	up	noncoding
ASHGA5P036334	0.015464833	0.076002496	2.8048465	up	noncoding
ASHGA5P036334	0.015464833	0.076002496	2.8048465	up	noncoding
ASHGA5P039027	0.033221327	0.114846653	2.6578025	up	noncoding
ASHGA5P029351	0.001797217	0.028435129	3.800567	up	noncoding
ASHGA5P028676	0.00087188	0.019883224	2.3952402	up	noncoding
ASHGA5P030816	0.002744502	0.033931843	3.4415946	up	noncoding
ASHGA5P030816	0.002744502	0.033931843	3.4415946	up	noncoding
ASHGA5P030816	0.002744502	0.033931843	3.4415946	up	noncoding
ASHGA5P030816	0.002744502	0.033931843	3.4415946	up	noncoding
ASHGA5P035554	0.006957855	0.052947146	2.543462	up	noncoding
ASHGA5P032676	8.7194E-05	0.008766146	4.9276375	up	noncoding
ASHGA5P032676	8.7194E-05	0.008766146	4.9276375	up	noncoding
ASHGA5P032676	8.7194E-05	0.008766146	4.9276375	up	noncoding
ASHGA5P043827	0.00034545	0.013385271	7.1785115	up	noncoding
ASHGA5P014852	0.015174398	0.075388534	2.1968029	up	noncoding
ASHGA5P033504	0.001010924	0.021549372	4.0143183	up	noncoding
ASHGA5P019467	0.006287672	0.050596613	2.2571034	up	noncoding
ASHGA5P019467	0.006287672	0.050596613	2.2571034	up	noncoding
ASHGA5P052897	0.01674407	0.078875667	2.5813928	up	noncoding
ASHGA5P021967	0.002158734	0.030562731	2.232472	up	noncoding
ASHGA5P056801	0.001669105	0.027358461	3.4418765	up	noncoding
ASHGA5P045302	0.016588707	0.078580714	2.3499889	up	noncoding
ASHGA5P054278	0.012594417	0.068740842	4.4975476	up	noncoding
ASHGA5P050404	0.000451876	0.014983741	2.0435707	up	noncoding
ASHGA5P015648	0.000647062	0.017096671	2.8957105	up	noncoding
ASHGA5P036499	0.004321334	0.041670179	2.0599079	up	noncoding
ASHGA5P030854	0.000731888	0.018183732	2.5023377	up	noncoding
ASHGA5P030854	0.000731888	0.018183732	2.5023377	up	noncoding
ASHGA5P035261	0.015631432	0.076538741	2.9142194	up	noncoding
ASHGA5P049943	0.000262998	0.01219365	4.7195022	up	noncoding
ASHGA5P049943	0.000262998	0.01219365	4.7195022	up	noncoding
ASHGA5P049943	0.000262998	0.01219365	4.7195022	up	noncoding
ASHGA5P049943	0.000262998	0.01219365	4.7195022	up	noncoding
ASHGA5P049943	0.000262998	0.01219365	4.7195022	up	noncoding
ASHGA5P049943	0.000262998	0.01219365	4.7195022	up	noncoding
ASHGA5P049943	0.000262998	0.01219365	4.7195022	up	noncoding
ASHGA5P049943	0.000262998	0.01219365	4.7195022	up	noncoding
ASHGA5P014188	0.00015681	0.011056858	6.8356822	up	noncoding
ASHGA5P032251	0.011116094	0.065090819	2.4579221	up	noncoding
ASHGA5P025709	0.018181499	0.081962493	2.6805255	up	noncoding
ASHGA5P027770	0.033221861	0.114846653	3.3975072	up	noncoding
ASHGA5P028788	0.00498557	0.044828687	4.897791	up	noncoding
ASHGA5P025487	0.009019255	0.059313506	2.0761239	up	noncoding
ASHGA5P025487	0.009019255	0.059313506	2.0761239	up	noncoding
ASHGA5P040476	0.0459807	0.138173601	2.1285615	up	noncoding
ASHGA5P028430	0.005908911	0.048960114	2.1752966	up	noncoding
ASHGA5P054247	0.000146659	0.01076444	2.0335684	up	noncoding
ASHGA5P047820	0.028337824	0.105047356	2.2076015	up	noncoding
ASHGA5P049096	0.00488783	0.044366328	2.0328507	up	noncoding

uc010mce.2	BX641143	UCSC_knowngene	4286	chr8	+
ENST00000433576	RP11-815M8.1	GENCODE	720	chr1	-
uc010gir.2	BC037891	UCSC_knowngene	1165	chr20	+
ENST00000509649	RP11-823P9.1	GENCODE	362	chr5	+
ENST00000547750	RP11-61E11.1	GENCODE	289	chr12	+
TCONS_00025500	XLOC_012315	LincRNAs identified	1194	chr17	+
ENST00000510419	AC142293.3	GENCODE	732	chr4	-
uc002vyh.3	AF055024	UCSC_knowngene	1834	chr2	-
ENST00000558556	CTD-2008A1.2	pseudogene	1069	chr15	-
ENST00000513802	RP11-231L11.3	GENCODE	393	chr3	+
ENST00000513802	RP11-231L11.3	GENCODE	393	chr3	+
ENST00000533775	TSSC2	GENCODE	1210	chr11	+
ENST00000489557	RP11-486F17.1	GENCODE	831	chr2	+
ENST00000489557	RP11-486F17.1	GENCODE	831	chr2	+
ENST00000489557	RP11-486F17.1	GENCODE	831	chr2	+
uc003erl.1	AX746877	UCSC_knowngene	1139	chr3	+
ENST00000557778	RP11-1029J19.5	GENCODE	454	chr14	-
ENST00000417976	BX248398.1	GENCODE	1054	chr1	-
ENST00000558696	RP11-299G20.3	GENCODE	639	chr15	+
ENST00000558696	RP11-299G20.3	GENCODE	639	chr15	+
ENST00000558696	RP11-299G20.3	GENCODE	639	chr15	+
ENST00000558696	RP11-299G20.3	GENCODE	639	chr15	+
ENST00000423530	LINC00607	GENCODE	2104	chr2	-
uc021uec.1	EPR-1	UCSC_knowngene	624	chr17	-
uc021uec.1	EPR-1	UCSC_knowngene	624	chr17	-
uc021uec.1	EPR-1	UCSC_knowngene	624	chr17	-
ENST00000429254	RP11-132A1.3	GENCODE	756	chr7	+
ENST00000414065	AC018685.1	GENCODE	547	chr2	-
ENST00000578278	RP11-146G7.3	GENCODE	695	chr18	-
ENST00000486431	RP11-666A20.1	GENCODE	1671	chr3	+
ENST00000486431	RP11-666A20.1	GENCODE	1671	chr3	+
NR_073096	SERAC1	RefSeq	1963	chr6	-
ENST00000545800	RP11-783K16.13	GENCODE	853	chr11	-
TCONS_00008753	XLOC_003455	LincRNAs identified	217	chr4	+
ENST00000418478	RP11-213G2.2	GENCODE	360	chr9	-
uc001hpx.3	BC032899	UCSC_knowngene	506	chr1	+
ENST00000438414	AC008073.7	GENCODE	674	chr2	-
ENST00000422201	AC093732.1	GENCODE	504	chr2	-
ENST00000445199	AC093171.1	GENCODE	517	chr2	+
ENST00000563223	LA16c-390E6.3	GENCODE	307	chr16	-
ENST00000563223	LA16c-390E6.3	GENCODE	307	chr16	-
ENST00000449075	AC093901.1	GENCODE	580	chr2	-
uc002kmj.1	AK091102	UCSC_knowngene	1917	chr18	+
uc002kmj.1	AK091102	UCSC_knowngene	1917	chr18	+
uc002kmj.1	AK091102	UCSC_knowngene	1917	chr18	+
uc002kmj.1	AK091102	UCSC_knowngene	1917	chr18	+
uc002kmj.1	AK091102	UCSC_knowngene	1917	chr18	+
uc002kmj.1	AK091102	UCSC_knowngene	1917	chr18	+
uc002kmj.1	AK091102	UCSC_knowngene	1917	chr18	+
uc002kmj.1	AK091102	UCSC_knowngene	1917	chr18	+
ENST00000400831	RP11-351J23.1	GENCODE	2557	chr6	-
ENST00000573168	CTC-457L16.2	GENCODE	2673	chr17	-
NR_027405	MTHFD2	RefSeq	2306	chr2	+
TCONS_00010144	XLOC_004611	LincRNAs identified	1464	chr5	+
ENST00000432967	AKAP11-IT1	GENCODE	539	chr13	+
NR_003104	POT1	RefSeq	4192	chr7	-
NR_003104	POT1	RefSeq	4192	chr7	-
ENST00000515199	CTC-537E7.2	GENCODE	340	chr5	-
ENST00000577004	RP11-363G2.5	GENCODE	1177	chr13	-
NR_026761	LINC00467	RefSeq	797	chr1	+
ENST00000449749	AC068580.7	GENCODE	338	chr11	-
ENST00000564490	CTD-2380F24.1	GENCODE	686	chr16	-

104476927	104481213		ATAGCCAAGTGTTTGCTTATTTACT		intergenic
222054322	222092054		GCATTTTCCTGTCCCTTCTAAAATTT		intergenic
55841852	55858053		AGAAGAGAGCTCCAGTTACCAGGGC		bidirectional
21459605	21462099		AAGAAGTTCAGATATTTGTCGTTTTA		intergenic
105466263	105467799		CTCTGGCCAATTAGTGCCAACTTCAC		natural antisense
75249855	75266597		GTTGATAAATATTCCTGACCCAGCCT		intergenic
75418300	75514664		ATTTAGAAAGATATCTTCACTCCAAC		intronic antisense
239359012	239360880		CAACCAACATTTTGTTTTGCAACCAA		natural antisense
45119257	45154048	NM_003104, NR_03	TGCATGGACCAGGTGACTTGCGCCTGGAGAACTATCCT		
140691585	140692097		CTGGTGGAATCTGTAGGTAATTAAG		intron sense-overlapp
140691585	140692097		CTGGTGGAATCTGTAGGTAATTAAG		intron sense-overlapp
3404691	3430191		TAGAAAGTAGAGTTTGAACAACTGA		intergenic
200190032	200194085		GTCCTGCTTAGCTTTACAAATCTGCC		natural antisense
200190032	200194085		GTCCTGCTTAGCTTTACAAATCTGCC		natural antisense
200190032	200194085		GTCCTGCTTAGCTTTACAAATCTGCC		natural antisense
136734712	136735851		TTCCACTTGAACTTTCTGAGAACGGT		intergenic
102100790	102197445		AAGGTGTTGAAGCATAGACGCTGGA		intronic antisense
144299757	144301536	ENST00000431928	TCATGTGCTTGTGAAAATGGCATAA		intergenic
101874641	101877633		CCTTTGGAAGACGTAAAAATGAGAA		intronic antisense
101874641	101877633		CCTTTGGAAGACGTAAAAATGAGAA		intronic antisense
101874641	101877633		CCTTTGGAAGACGTAAAAATGAGAA		intronic antisense
101874641	101877633		CCTTTGGAAGACGTAAAAATGAGAA		intronic antisense
216484357	216692126		CAGACTCATAAAT	646324	intergenic
76220156	76220780		TTGTTAAACAGTAGAGGAGCCAGGC		natural antisense
76220156	76220780		TTGTTAAACAGTAGAGGAGCCAGGC		natural antisense
76220156	76220780		TTGTTAAACAGTAGAGGAGCCAGGC		natural antisense
100942893	100944551		ATCTGAAGCATTTTTCTCACTTTAAA		intergenic
2645760	2660402		GGCACACAAGATTTTCTAGATGGCT		intergenic
6925475	6926989	ENST00000580197,	CCACCGAATTTCAAGAGCCCAAGCA		intergenic
125635467	125648832		CATCAAAAAGGCCAGGCCAGGATCA		natural antisense
125635467	125648832		CATCAAAAAGGCCAGGCCAGGATCA		natural antisense
158535502	158589312		TCTCATTTTATAA	84947	exon sense-overlappi
64014410	64016966		GTGGCACAAGCCTTCTGAGGAAATG		exon sense-overlappi
11315512	11330339	TCONS_00007986, '	GTCAAAGAAATCTTATGAAGCAAGC		intergenic
88400636	88406454	ENST00000439378	CTGCTGTTCTTGCTACTAGTGAATTC		intergenic
226271655	226277994		CTTTCTATGAGGTCCTAGGAATGAA		intergenic
24392817	24397874		CGCCGAGGCCCCCGCCGCCCTTCTGC		bidirectional
47262417	47267663		TGATATGAAAACAAACAAAACAACC		intronic antisense
230990025	231032210		TTTTTCCTGGGAAAGAAAACTTGTT		intergenic
1517673	1522685		GGTTTCCTGTCTGTCTTTGCAAATAC		intron sense-overlapp
1517673	1522685		GGTTTCCTGTCTGTCTTTGCAAATAC		intron sense-overlapp
118940514	118943949		GATGGATGTCTGGAATCGTTGTGAT		intergenic
3604967	3606884		CTTCTACACTGACCAGTAGTTTTGCT		intronic antisense
3604967	3606884		CTTCTACACTGACCAGTAGTTTTGCT		intronic antisense
3604967	3606884		CTTCTACACTGACCAGTAGTTTTGCT		intronic antisense
3604967	3606884		CTTCTACACTGACCAGTAGTTTTGCT		intronic antisense
3604967	3606884		CTTCTACACTGACCAGTAGTTTTGCT		intronic antisense
3604967	3606884		CTTCTACACTGACCAGTAGTTTTGCT		intronic antisense
3604967	3606884		CTTCTACACTGACCAGTAGTTTTGCT		intronic antisense
3604967	3606884		CTTCTACACTGACCAGTAGTTTTGCT		intronic antisense
168080305	168084467		TTCCCAAGGCAGAGATGAAGAATGC		intergenic
19044329	19047002		TTCAGGTCTGATCATGTGCGTGTGCC		intronic antisense
74425689	74442424		CGGCAGTTCGGG	10797	exon sense-overlappi
154258473	154263999		GGCCTAGGAATGGATGTCACTGGAA		intergenic
42848463	42851124		CCTGGGTTAAAATTCTGAACATATTT		intron sense-overlapp
124462439	124570037		GTACTCAAGGTTA	25913	exon sense-overlappi
124462439	124570037		GTACTCAAGGTTA	25913	exon sense-overlappi
67827516	67829357		CTGAAGAACCCCTTCCTGATATTGTC		intergenic
23468794	23490508		TTATTTCTTTTGAAAATACCTTGGTC		intergenic
211556096	211605877		TGCGCGCGCTGTC	84791	intergenic
1798159	1799618		AAATGGCAGCGTTGTGGGTAAAATA		intergenic
19772493	19777421		AAATGCCCGTTAAAGCAAGAGCTCT		intronic antisense

NM_001719	BMP7	bone morphogenetic	-	55743808	55841707
NM_001034173	ALDH1L2	mitochondrial 10-for	-	105413561	105478341
NM_001657	AREG	amphiregulin preprop	+	75480628	75490485
NM_001040445	ASB1	ankyrin repeat and S(	+	239335625	239360891
'ATCCCTGAACCAGGCCCAAATG					
NM_001104647	SLC25A36	solute carrier family	+	140660661	140698785
NM_018155	SLC25A36	solute carrier family	+	140660661	140698785
NM_001172509	SATB2	DNA-binding protein	-	200134222	200322819
NM_001172517	SATB2	DNA-binding protein	-	200134222	200329831
NM_015265	SATB2	DNA-binding protein	-	200134222	200335989
ENST00000556973	RP11-796G6.2	Uncharacterized prot	+	102196773	102198859
NM_002570	PCSK6	proprotein convertase	-	101844132	102030187
NM_138319	PCSK6	proprotein convertase	-	101844132	102030187
NM_138320	PCSK6	proprotein convertase	-	101853222	102030187
NM_138321	PCSK6	proprotein convertase	-	101853222	102030187
NM_001012270	BIRC5	baculoviral IAP repe	+	76210276	76221716
NM_001012271	BIRC5	baculoviral IAP repe	+	76210276	76221716
NM_001168	BIRC5	baculoviral IAP repe	+	76210276	76221716
NM_001015050	ALG1L	putative glycosyltran	-	125648117	125655887
NM_001195223	ALG1L	putative glycosyltran	-	125648117	125655887
NM_032861	SERAC1	protein SERAC1	-	158530535	158589312
NM_138689	PPP1R14B	protein phosphatase	-	64011950	64014413
NM_001040710	FAM228A	UPF0638 protein C2	+	24397971	24414567
NM_020458	TTC7A	tetratricopeptide repe	+	47168312	47303275
NM_001114331	CLCN7	H(+)/Cl(-) exchange	-	1494933	1525085
NM_001287	CLCN7	H(+)/Cl(-) exchange	-	1494933	1525085
NM_001003809	DLGAP1	disks large-associated	-	3496029	3845358
NM_001242761	DLGAP1	disks large-associated	-	3502142	4455266
NM_001242762	DLGAP1	disks large-associated	-	3496029	3874767
NM_001242763	DLGAP1	disks large-associated	-	3496029	3874767
NM_001242764	DLGAP1	disks large-associated	-	3496029	3874253
NM_001242765	DLGAP1	disks large-associated	-	3502142	3845358
NM_001242766	DLGAP1	disks large-associated	-	3496029	3845358
NM_004746	DLGAP1	disks large-associated	-	3496029	4455266
NM_001129778	GRAPL	GRB2-related adapte	+	19030781	19062148
NM_006636	MTHFD2	bifunctional methyler	+	74425689	74442424
NM_016248	AKAP11	A-kinase anchor prot	+	42846288	42897403
NM_001042594	POT1	protection of telomer	-	124462439	124570037
NM_015450	POT1	protection of telomer	-	124462439	124570037
NM_153208	IQCK	IQ domain-containin	+	19727777	19868859

31.943144	8.801272	4.589093	3.39348	25.121817	16.29239
122.090716	24.064187	6.352535	4.848405	95.141785	35.72408
155.301881	29.560438	6.492027	5.038521	256.28546	381.28278
2927.495167	927.784678	11.081976	10.045795	3192.9148	2738.542
28.079908	5.595856	4.460608	2.6289	21.358988	26.822145
422.65963	130.336066	8.304824	7.132131	331.12476	543.15576
63.165963	6.014121	5.181178	2.589144	37.435215	50.580456
18.849348	6.102893	3.883007	2.576924	19.802969	15.092369
63.220671	16.51944	5.59423	4.29141	76.58821	89.96494
40.96359	11.728291	5.01049	3.697329	44.53359	34.500988
40.96359	11.728291	5.01049	3.697329	44.53359	34.500988
635.23287	176.443806	8.907419	7.786391	722.501	737.3355
100.234502	22.29236	6.102678	4.614756	141.08636	106.11459
100.234502	22.29236	6.102678	4.614756	141.08636	106.11459
100.234502	22.29236	6.102678	4.614756	141.08636	106.11459
46.352078	10.466007	4.980345	3.570112	25.074202	13.62734
40.428752	5.980746	4.61923	2.693016	48.89009	102.62617
264.461605	70.505527	7.603491	6.343321	286.81595	152.87856
25.415237	5	4.146557	2.363479	37.937622	49.628048
25.415237	5	4.146557	2.363479	37.937622	49.628048
25.415237	5	4.146557	2.363479	37.937622	49.628048
25.415237	5	4.146557	2.363479	37.937622	49.628048
21.350672	6.176836	4.04418	2.697387	18.868134	13.46593
1875.5043	230.592033	10.450246	8.14935	2035.9879	2207.5007
1875.5043	230.592033	10.450246	8.14935	2035.9879	2207.5007
1875.5043	230.592033	10.450246	8.14935	2035.9879	2207.5007
79.337553	6.14564	5.659044	2.81536	88.23492	160.31157
97.509396	29.758567	6.176678	5.041272	155.01308	130.4139
10743.3985	1881.975993	12.767062	10.761907	12033.65	4629.5547
490.529527	140.040103	8.529723	7.35525	451.44937	559.66693
490.529527	140.040103	8.529723	7.35525	451.44937	559.66693
82.229612	20.041479	5.921204	4.553054	75.58969	63.182243
92.775414	25.986782	6.036819	4.878177	106.7912	130.55272
40.564441	6.537911	4.621265	2.83807	61.13047	89.75235
23.753667	7.559064	4.186589	2.953935	34.50448	33.29757
122.025444	17.6574	6.492372	4.323233	159.93501	84.019905
3167.905133	972.13235	11.200222	10.16913	3251.1765	3184.8645
140.149573	27.559284	6.496347	4.96243	164.87236	273.76172
16.349766	5.908041	3.715785	2.673206	14.201421	14.6313715
141.608872	38.907483	6.770527	5.44725	165.89449	133.05998
141.608872	38.907483	6.770527	5.44725	165.89449	133.05998
42.641376	8.908871	4.748271	3.205162	26.977674	46.013046
57.20224	7.611911	5.289484	3.05085	56.11313	34.4077
57.20224	7.611911	5.289484	3.05085	56.11313	34.4077
57.20224	7.611911	5.289484	3.05085	56.11313	34.4077
57.20224	7.611911	5.289484	3.05085	56.11313	34.4077
57.20224	7.611911	5.289484	3.05085	56.11313	34.4077
57.20224	7.611911	5.289484	3.05085	56.11313	34.4077
57.20224	7.611911	5.289484	3.05085	56.11313	34.4077
57.20224	7.611911	5.289484	3.05085	56.11313	34.4077
64.817301	7.032902	5.558501	2.785415	45.317234	23.33662
27.963069	7.529922	4.437331	3.139892	33.879936	33.759583
45.862884	12.574139	5.144809	3.722293	55.37169	65.95664
128.079212	42.251235	6.611771	4.847294	160.04532	137.71985
82.044851	15.626574	5.809489	3.517358	96.1079	31.698442
91.171504	27.218785	6.105904	5.052011	84.6303	61.011158
91.171504	27.218785	6.105904	5.052011	84.6303	61.011158
64.863195	17.109346	5.44791	4.358031	88.23384	34.64473
17.860698	6.307253	3.819734	2.698522	20.67651	14.485071
15.236412	5.761828	3.612376	2.588362	17.093569	13.873999
37.24586	9.12996	4.595325	3.452845	15.60716	17.181433
37.500444	12.223138	4.88742	3.863916	46.543194	34.90132

22.358536	48.27478	31.55634	48.055	7.0696597	6.48841
133.19868	64.82521	211.49254	192.162	18.163101	19.02207
60.274204	111.46438	42.682114	79.82235	33.195713	55.94402
3621.6743	2612.0098	2237.7014	3162.1287	1144.092	486.05713
19.511013	33.64427	30.389256	36.753777	4.9999995	6.2782946
417.72495	267.76367	499.03574	477.1529	137.47168	56.37312
10.021261	158.79628	30.666588	91.49598	11.084731	4.9999995
28.01645	13.26744	20.085136	16.831724	4.9999995	4.9999995
41.405235	56.884106	50.98825	63.493282	19.204441	15.869531
46.305958	54.17527	25.654991	40.610744	17.32765	6.705357
46.305958	54.17527	25.654991	40.610744	17.32765	6.705357
672.2703	447.28568	584.8795	647.12524	158.35193	118.789925
167.97179	31.084967	76.91985	78.229454	16.27931	10.100369
167.97179	31.084967	76.91985	78.229454	16.27931	10.100369
167.97179	31.084967	76.91985	78.229454	16.27931	10.100369
30.072884	72.53946	59.28897	77.50961	8.2703705	13.394489
9.595249	22.285568	24.839626	34.33581	4.9999995	10.696641
421.94775	169.84859	260.0192	295.25958	98.45247	47.092068
14.530311	20.450848	10.969951	18.974642	4.9999995	4.9999995
14.530311	20.450848	10.969951	18.974642	4.9999995	4.9999995
14.530311	20.450848	10.969951	18.974642	4.9999995	4.9999995
14.530311	20.450848	10.969951	18.974642	4.9999995	4.9999995
16.22613	19.889318	26.582129	33.07239	6.865143	4.9999995
1748.7289	1459.9291	1784.9928	2015.8864	214.81609	102.40021
1748.7289	1459.9291	1784.9928	2015.8864	214.81609	102.40021
1748.7289	1459.9291	1784.9928	2015.8864	214.81609	102.40021
16.368587	49.460888	66.949394	94.69996	6.580181	7.83813
69.579124	61.64359	86.3883	82.01838	27.264772	16.63969
18310.545	3676.6843	13611.876	12198.081	1400.3469	307.27686
330.644	595.65466	437.5925	568.1697	83.88746	119.90019
330.644	595.65466	437.5925	568.1697	83.88746	119.90019
68.972404	44.21208	109.457695	131.96356	25.811104	33.99807
84.95149	29.545143	112.22649	92.58544	25.75367	17.192327
17.133575	38.658	7.8810525	28.831198	5.948466	11.334719
18.544588	24.857416	15.0888405	16.22911	8.88539	4.9999995
205.48775	77.62398	91.88322	113.2028	19.045572	28.69784
3997.475	2436.8667	2894.3132	3242.7349	1192.0962	593.6134
55.981865	181.18628	38.43719	126.65802	21.099705	48.635895
13.962341	21.772234	15.08707	18.444157	5.7765727	4.9999995
184.5309	109.54738	112.71457	143.90591	48.63681	14.01082
184.5309	109.54738	112.71457	143.90591	48.63681	14.01082
4.9999995	43.572437	66.47658	67.80852	4.9999995	5.826825
59.05526	17.840769	94.31272	81.483864	8.090139	4.9999995
59.05526	17.840769	94.31272	81.483864	8.090139	4.9999995
59.05526	17.840769	94.31272	81.483864	8.090139	4.9999995
59.05526	17.840769	94.31272	81.483864	8.090139	4.9999995
59.05526	17.840769	94.31272	81.483864	8.090139	4.9999995
59.05526	17.840769	94.31272	81.483864	8.090139	4.9999995
59.05526	17.840769	94.31272	81.483864	8.090139	4.9999995
59.05526	17.840769	94.31272	81.483864	8.090139	4.9999995
59.645058	80.17873	81.80372	98.622444	4.9999995	4.9999995
33.198215	17.429518	26.055948	23.455217	6.600071	5.2534842
32.166664	41.22252	42.44392	38.01587	14.469659	5.479945
123.53269	75.485504	139.33691	132.355	72.357666	8.009714
160.65979	93.014725	31.194208	79.59404	19.132988	4.9999995
77.13067	126.971664	60.852703	136.43253	24.565891	25.846668
77.13067	126.971664	60.852703	136.43253	24.565891	25.846668
146.81271	55.52984	26.113314	37.844734	13.667359	13.75052
20.209827	13.2429695	16.29336	22.256449	5.5300894	4.9999995
19.555101	15.43681	11.93129	13.5277	6.5748906	4.9999995
13.765881	49.885666	67.78523	59.24979	6.3745236	7.7298527
26.523844	46.35514	40.05256	30.626608	8.185054	10.38592

11.866691	8.79231	8.22697	10.363592	4.1914296	4.040421
27.676805	39.350376	17.935728	22.237043	6.125025	5.0705757
20.883617	33.38439	10.7752495	23.17964	7.5444484	8.285212
1987.9485	488.2014	887.9575	572.45154	11.167049	11.005357
4.9999995	7.296844	4.9999995	4.9999995	3.9568033	4.692999
315.6636	84.81764	98.941956	88.7484	7.915357	8.774788
4.9999995	4.9999995	4.9999995	4.9999995	4.7607245	5.526905
11.61736	4.9999995	4.9999995	4.9999995	3.8478281	3.9375293
18.79021	20.54204	16.39612	8.314298	5.804283	6.2878036
18.682926	13.24275	8.501823	5.909238	5.0200667	5.02645
18.682926	13.24275	8.501823	5.909238	5.0200667	5.02645
209.37553	162.93074	248.91994	160.29477	9.028195	9.202325
24.947393	12.9894495	41.426907	28.010729	6.689171	6.512019
24.947393	12.9894495	41.426907	28.010729	6.689171	6.512019
24.947393	12.9894495	41.426907	28.010729	6.689171	6.512019
4.9999995	17.22887	7.6898236	11.21249	4.1884904	3.8059404
4.9999995	5.1878376	4.9999995	4.9999995	5.151762	6.467302
125.3224	44.31626	63.282246	44.56772	7.7080994	7.0183516
4.9999995	4.9999995	4.9999995	4.9999995	4.7813497	5.4998183
4.9999995	4.9999995	4.9999995	4.9999995	4.7813497	5.4998183
4.9999995	4.9999995	4.9999995	4.9999995	4.7813497	5.4998183
4.9999995	4.9999995	4.9999995	4.9999995	4.7813497	5.4998183
8.382483	6.813392	4.9999995	4.9999995	3.7756088	3.7913766
298.1072	253.25133	277.61752	237.35985	10.508357	10.692127
298.1072	253.25133	277.61752	237.35985	10.508357	10.692127
298.1072	253.25133	277.61752	237.35985	10.508357	10.692127
4.9999995	4.9999995	7.4555326	4.9999995	6.010209	7.081414
65.50203	22.042871	25.08729	22.014751	6.818631	6.7945495
2452.8777	491.1223	4502.6675	2137.5647	13.11075	11.737667
58.65279	210.38742	197.65701	169.75575	8.343381	8.817958
58.65279	210.38742	197.65701	169.75575	8.343381	8.817958
12.902031	15.19393	16.597412	15.74633	5.7862334	5.812722
43.08248	13.484072	36.445595	19.962547	6.29234	6.7961006
4.9999995	6.9442806	4.9999995	4.9999995	5.4756737	6.2837324
13.66345	6.171361	4.9999995	6.634186	4.646934	4.981494
5.8831573	23.103878	12.0722	17.14175	6.8625493	6.198168
1963.9685	741.347	740.3465	601.4225	11.191627	11.221441
12.18321	40.51312	16.69909	26.224684	6.907239	7.830685
7.7320857	4.9999995	6.9395885	4.9999995	3.3561351	3.8961308
67.516	31.983452	39.70916	31.588657	6.9163547	6.823198
67.516	31.983452	39.70916	31.588657	6.9163547	6.823198
4.9999995	20.889389	7.865444	8.871566	4.2985954	5.4006066
8.189777	5.100463	12.27793	7.013155	5.3471713	5.0236588
8.189777	5.100463	12.27793	7.013155	5.3471713	5.0236588
8.189777	5.100463	12.27793	7.013155	5.3471713	5.0236588
8.189777	5.100463	12.27793	7.013155	5.3471713	5.0236588
8.189777	5.100463	12.27793	7.013155	5.3471713	5.0236588
8.189777	5.100463	12.27793	7.013155	5.3471713	5.0236588
8.189777	5.100463	12.27793	7.013155	5.3471713	5.0236588
8.189777	5.100463	12.27793	7.013155	5.3471713	5.0236588
4.9999995	4.9999995	15.35282	6.844592	5.043401	4.5011563
7.7514534	12.997469	5.5804863	6.996571	4.6196284	4.998079
24.377996	16.681667	5.2899356	9.14563	5.3253365	5.8697395
132.5672	16.828447	9.765343	13.97904	6.863506	6.8762584
46.98487	4.9999995	12.641589	4.9999995	6.138674	4.913529
16.346249	37.779846	33.023	25.751057	5.949608	5.769944
16.346249	37.779846	33.023	25.751057	5.949608	5.769944
22.852798	12.894908	19.3293	20.161192	6.010092	5.0318465
10.266488	7.046941	4.9999995	4.9999995	3.9096634	3.8795578
7.996077	4.9999995	4.9999995	4.9999995	3.622942	3.8270633
9.891688	12.009472	9.739136	9.035088	3.4913578	4.108164
8.560635	16.686636	14.24089	15.279691	5.082461	5.041581

4.1697717	5.1966147	4.837557	5.0987654	3.0156307	3.4530287
6.731858	5.6466036	7.482416	7.058729	4.4185843	5.073177
5.6022563	6.457069	5.2575397	5.8056364	5.2834845	6.659038
11.459146	11.005357	10.837766	11.0171795	10.460816	9.789701
3.9645047	4.6300178	4.78409	4.7352324	2.3467326	3.401673
8.363289	7.7466054	8.705725	8.323177	7.3300815	6.6716013
3.0137167	6.9909477	4.797777	5.996995	3.7007189	2.4279919
4.499286	3.1354063	4.227489	3.6505048	2.3467326	2.4279919
5.053482	5.4454794	5.4945555	5.479774	4.500871	4.8064885
5.2123337	5.3721776	4.564861	4.867053	4.353104	3.5049899
5.2123337	5.3721776	4.564861	4.867053	4.353104	3.5049899
9.028195	8.495712	8.93032	8.759766	7.5512576	7.739751
7.061634	4.513941	6.060962	5.7783403	4.2569375	4.118314
7.061634	4.513941	6.060962	5.7783403	4.2569375	4.118314
7.061634	4.513941	6.060962	5.7783403	4.2569375	4.118314
4.602726	5.819184	5.6998444	5.7658873	3.2536128	4.5470037
2.953177	3.9799414	4.5212617	4.641938	2.3467326	4.2087736
8.37692	7.078644	7.7807927	7.6581407	6.8546376	6.4059496
3.5361571	3.8350055	3.4089556	3.8180535	2.3467326	2.4279919
3.5361571	3.8350055	3.4089556	3.8180535	2.3467326	2.4279919
3.5361571	3.8350055	3.4089556	3.8180535	2.3467326	2.4279919
3.5361571	3.8350055	3.4089556	3.8180535	2.3467326	2.4279919
3.7031064	3.7917395	4.6114135	4.5918374	2.9768457	2.4279919
10.409206	10.194751	10.504383	10.39265	7.9944077	7.5288925
10.409206	10.194751	10.504383	10.39265	7.9944077	7.5288925
10.409206	10.194751	10.504383	10.39265	7.9944077	7.5288925
3.7149806	5.2370453	5.864888	6.04573	2.9151864	3.735219
5.810545	5.5699444	6.2267313	5.8396645	5.0065136	4.8723693
13.904315	11.498745	13.417522	12.933375	10.738141	9.123176
8.0353	8.899571	8.514295	8.567831	6.615508	7.7518415
8.0353	8.899571	8.514295	8.567831	6.615508	7.7518415
5.7969284	5.055658	6.558033	6.517649	4.93216	5.9266467
6.0921493	4.430452	6.595354	6.0145164	4.930354	4.924103
3.7794375	4.8463974	2.949308	4.393042	2.7757387	4.2939744
3.8936048	4.15622	3.8419373	3.599344	3.3659308	2.4279919
7.3672194	5.9159765	6.310869	6.29945	4.4873343	5.680401
11.610452	10.915692	11.214177	11.047943	10.518034	10.069458
5.4916573	7.1781096	5.1105514	6.4598413	4.6378922	6.4514074
3.4768379	3.9419389	3.8417947	3.7818747	2.7301795	2.4279919
7.206245	6.4299316	6.601903	6.645529	5.8374357	4.6189504
7.206245	6.4299316	6.601903	6.645529	5.8374357	4.6189504
2.3258038	5.0347667	5.8557734	5.5740805	2.3467326	3.2829602
5.5712175	3.6156893	6.347694	5.8314757	3.2183049	2.4279919
5.5712175	3.6156893	6.347694	5.8314757	3.2183049	2.4279919
5.5712175	3.6156893	6.347694	5.8314757	3.2183049	2.4279919
5.5712175	3.6156893	6.347694	5.8314757	3.2183049	2.4279919
5.5712175	3.6156893	6.347694	5.8314757	3.2183049	2.4279919
5.5712175	3.6156893	6.347694	5.8314757	3.2183049	2.4279919
5.5712175	3.6156893	6.347694	5.8314757	3.2183049	2.4279919
5.5712175	3.6156893	6.347694	5.8314757	3.2183049	2.4279919
5.5862265	5.966366	6.1504903	6.103363	2.3467326	2.4279919
4.7396126	3.578799	4.585051	4.1028185	2.9190226	3.1231673
4.6950574	4.9497857	5.2495847	4.7793493	4.084797	3.1915407
6.6284337	5.8786163	6.9021025	6.521708	6.4078817	3.766464
6.993594	6.186941	4.8225617	5.801633	4.494563	2.4279919
5.9537373	6.6586704	5.7370043	6.566458	4.860613	5.5260644
5.9537373	6.6586704	5.7370043	6.566458	4.860613	5.5260644
6.872517	5.412138	4.5876474	4.773218	3.9999266	4.585132
4.0167656	3.131978	3.9497054	4.0307317	2.6682644	2.4279919
3.9683623	3.3856626	3.5220854	3.3481388	2.9135373	2.4279919
3.4564526	5.2476974	5.883565	5.384713	2.8711395	3.7127137
4.4181757	5.132658	5.164806	4.4848375	3.2367938	4.161701

3.657897	3.2348557	3.3350792	3.6643894
4.81032	5.5346456	4.4356217	4.8180795
4.42505	5.27702	3.7070186	4.8795123
10.89951	9.379478	10.039968	9.705296
2.350709	2.9597204	2.3618152	2.352752
8.251027	6.737011	6.884366	6.9186983
2.350709	2.3408759	2.3618152	2.352752
3.6313791	2.3408759	2.3618152	2.352752
4.2813272	4.5227823	4.308839	3.3281515
4.2730446	3.8483925	3.380406	2.8240397
4.2730446	3.8483925	3.380406	2.8240397
7.6604695	7.72336	8.217877	7.825628
4.6661053	3.8189805	5.6458116	5.1823874
4.6661053	3.8189805	5.6458116	5.1823874
4.6661053	3.8189805	5.6458116	5.1823874
2.350709	4.2497716	3.2380939	3.7814782
2.350709	2.5373118	2.3618152	2.352752
6.9309874	5.7197704	6.2487144	5.899867
2.350709	2.3408759	2.3618152	2.352752
2.350709	2.3408759	2.3618152	2.352752
2.350709	2.3408759	2.3618152	2.352752
2.350709	2.3408759	2.3618152	2.352752
3.1978693	2.8670468	2.3618152	2.352752
8.166046	8.414114	8.367121	8.425516
8.166046	8.414114	8.367121	8.425516
8.166046	8.414114	8.367121	8.425516
2.350709	2.3408759	3.1974163	2.352752
6.0136633	4.631938	4.920095	4.803054
11.218254	9.389385	12.426967	11.675521
5.860408	8.122254	7.873806	7.9076834
5.860408	8.122254	7.873806	7.9076834
3.7684033	4.0650077	4.328889	4.297219
5.424624	3.8768017	5.462109	4.651068
2.350709	2.8934298	2.3618152	2.352752
3.8456671	2.7300053	2.3618152	2.9922
2.7674325	4.709099	3.8722668	4.4228673
10.880179	10.003172	9.759966	9.78397
3.6937294	5.5782995	4.3367057	5.076545
3.0916831	2.3408759	3.0957508	2.352752
6.0573835	5.2101164	5.582863	5.3767533
6.0573835	5.2101164	5.582863	5.3767533
2.350709	4.551494	3.2700584	3.4290152
3.1663814	2.5246897	3.895083	3.0726476
3.1663814	2.5246897	3.895083	3.0726476
3.1663814	2.5246897	3.895083	3.0726476
3.1663814	2.5246897	3.895083	3.0726476
3.1663814	2.5246897	3.895083	3.0726476
3.1663814	2.5246897	3.895083	3.0726476
3.1663814	2.5246897	3.895083	3.0726476
3.1663814	2.5246897	3.895083	3.0726476
2.350709	2.3408759	4.2142487	3.031933
3.0947804	3.8198743	2.8144748	3.068034
4.6320844	4.2033253	2.7486336	3.473377
7.007196	4.2144294	3.5688038	4.1189904
5.552823	2.3408759	3.9351397	2.352752
4.091229	5.4721494	5.315744	5.046267
4.091229	5.4721494	5.315744	5.046267
4.5458055	3.8079543	4.539034	4.670334
3.4672792	2.9130266	2.3618152	2.352752
3.133201	2.3408759	2.3618152	2.352752
3.412644	3.6982055	3.5659158	3.4564526
3.2231522	4.203589	4.1042857	4.253972

ASHGA5P019880	0.019508343	0.085195149	2.6045077	up	noncoding
ASHGA5P037006	0.000836243	0.019402866	3.691863	up	noncoding
ASHGA5P055788	0.001437556	0.025364577	3.4138215	up	noncoding
ASHGA5P047936	0.018308108	0.082097163	2.1654	up	noncoding
ASHGA5P017104	0.007596301	0.05476971	3.2196926	up	noncoding
ASHGA5P032141	0.040638174	0.12894705	3.018057	up	noncoding
ASHGA5P032141	0.040638174	0.12894705	3.018057	up	noncoding
ASHGA5P032141	0.040638174	0.12894705	3.018057	up	noncoding
ASHGA5P032141	0.040638174	0.12894705	3.018057	up	noncoding
ASHGA5P032141	0.040638174	0.12894705	3.018057	up	noncoding
ASHGA5P032141	0.040638174	0.12894705	3.018057	up	noncoding
ASHGA5P035476	1.28197E-05	0.004261616	4.149326	up	noncoding
ASHGA5P021643	8.3724E-05	0.008738697	3.0085869	up	noncoding
ASHGA5P021643	8.3724E-05	0.008738697	3.0085869	up	noncoding
ASHGA5P021643	8.3724E-05	0.008738697	3.0085869	up	noncoding
ASHGA5P049357	0.002921466	0.034973658	2.2062401	up	noncoding
ASHGA5P030199	0.007730954	0.055191161	3.0049499	up	noncoding
ASHGA5P025755	0.022622456	0.093083856	2.0327277	up	noncoding
ASHGA5P025755	0.022622456	0.093083856	2.0327277	up	noncoding
ASHGA5P025755	0.022622456	0.093083856	2.0327277	up	noncoding
ASHGA5P025755	0.022622456	0.093083856	2.0327277	up	noncoding
ASHGA5P025755	0.022622456	0.093083856	2.0327277	up	noncoding
ASHGA5P029341	0.008012821	0.056265282	2.0716439	up	noncoding
ASHGA5P015864	4.94512E-05	0.007378879	2.2000951	up	noncoding
ASHGA5P027229	0.002191478	0.030784944	3.7135157	up	noncoding
ASHGA5P017082	0.00064467	0.017062064	4.6883867	up	noncoding
ASHGA5P023152	0.013118115	0.070080694	3.4166505	up	noncoding
ASHGA5P031100	0.008095374	0.056581181	2.048235	up	noncoding
ASHGA5P020049	0.003789109	0.039485661	2.2115283	up	noncoding
ASHGA5P017704	0.000185099	0.01146874	2.3835254	up	noncoding
ASHGA5P017704	0.000185099	0.01146874	2.3835254	up	noncoding
ASHGA5P017704	0.000185099	0.01146874	2.3835254	up	noncoding
ASHGA5P033150	0.000832534	0.019383269	2.4564602	up	noncoding
ASHGA5P037185	0.005743068	0.048164708	2.9573699	up	noncoding
ASHGA5P033152	1.21955E-05	0.004221854	2.224938	up	noncoding
ASHGA5P000456	0.043996109	0.134664659	2.0681541	up	noncoding
ASHGA5P029713	0.000147524	0.01076444	4.546374	up	noncoding
ASHGA5P053031	0.000374351	0.013880988	2.0500429	up	noncoding
ASHGA5P017425	0.008192387	0.056791621	3.4336599	up	noncoding
ASHGA5P017425	0.008192387	0.056791621	3.4336599	up	noncoding
ASHGA5P017425	0.008192387	0.056791621	3.4336599	up	noncoding
ASHGA5P017425	0.008192387	0.056791621	3.4336599	up	noncoding
ASHGA5P017425	0.008192387	0.056791621	3.4336599	up	noncoding
ASHGA5P017425	0.008192387	0.056791621	3.4336599	up	noncoding
ASHGA5P017425	0.008192387	0.056791621	3.4336599	up	noncoding
ASHGA5P017425	0.008192387	0.056791621	3.4336599	up	noncoding
ASHGA5P017425	0.008192387	0.056791621	3.4336599	up	noncoding
ASHGA5P017425	0.008192387	0.056791621	3.4336599	up	noncoding
ASHGA5P017425	0.008192387	0.056791621	3.4336599	up	noncoding
ASHGA5P017425	0.008192387	0.056791621	3.4336599	up	noncoding
ASHGA5P017425	0.008192387	0.056791621	3.4336599	up	noncoding
ASHGA5P022693	0.000201761	0.01146874	3.4171314	up	noncoding
ASHGA5P056739	0.017709072	0.08079239	2.0413415	up	noncoding
ASHGA5P057525	0.038726874	0.125317529	2.1486131	up	noncoding
ASHGA5P057377	0.022866477	0.093657324	2.0095245	up	noncoding
ASHGA5P054732	0.031373871	0.111265481	3.9812204	up	noncoding
ASHGA5P021001	9.6664E-05	0.009080764	2.9457354	up	noncoding
ASHGA5P000046	0.002892044	0.034821141	2.1423387	up	noncoding
ASHGA5P020462	0.009235789	0.059806719	3.0259671	up	noncoding
ASHGA5P054543	9.41694E-05	0.009080764	3.6029786	up	noncoding
ASHGA5P022529	0.000108194	0.009526401	4.7963127	up	noncoding
ASHGA5P053146	0.012181829	0.067588007	2.0365217	up	noncoding

ENST00000504620	CTC-806A22.1	GENCODE	530	chr5	-
ENST00000565390	RP1-228H13.5	GENCODE	2446	chr1	+
uc001vdy.3	DLEU2	UCSC_knowngene	1279	chr13	-
ENST00000532541	CTC-497E21.4	GENCODE	1124	chr11	-
ENST00000437707	RP11-27I1.4	GENCODE	430	chr9	+
ENST00000571370	RP11-199F11.2	GENCODE	1112	chr17	-
ENST00000571370	RP11-199F11.2	GENCODE	1112	chr17	-
ENST00000571370	RP11-199F11.2	GENCODE	1112	chr17	-
ENST00000571370	RP11-199F11.2	GENCODE	1112	chr17	-
ENST00000571370	RP11-199F11.2	GENCODE	1112	chr17	-
ENST00000571370	RP11-199F11.2	GENCODE	1112	chr17	-
ENST00000429929	AC008174.3	GENCODE	440	chr2	-
NR_003098	SNHG1	RefSeq	1134	chr11	-
NR_003098	SNHG1	RefSeq	1134	chr11	-
NR_003098	SNHG1	RefSeq	1134	chr11	-
TCONS_00021927	XLOC_010525	LincRNAs identified	932	chr13	-
uc010bmn.3	LOC727849	UCSC_knowngene	3113	chr15	-
NR_033699	FMR1	RefSeq	4215	chrX	+
NR_033699	FMR1	RefSeq	4215	chrX	+
NR_033699	FMR1	RefSeq	4215	chrX	+
NR_033699	FMR1	RefSeq	4215	chrX	+
NR_033699	FMR1	RefSeq	4215	chrX	+
ENST00000555250	RP11-909M7.3	GENCODE	707	chr14	-
ENST00000424274	RP11-349P19.1	GENCODE	525	chr6	+
ENST00000547748	RP11-493L12.3	GENCODE	663	chr12	-
ENST00000437494	AC005077.5	GENCODE	300	chr7	+
ENST00000571449	RP11-473M20.14	GENCODE	919	chr16	-
ENST00000569291	RP11-452L6.1	GENCODE	449	chr16	-
ENST00000507301	FAM86EP	GENCODE	624	chr4	-
ENST00000444872	RP11-32B11.2	GENCODE	433	chr9	+
ENST00000444872	RP11-32B11.2	GENCODE	433	chr9	+
ENST00000444872	RP11-32B11.2	GENCODE	433	chr9	+
ENST00000444561	AC126544.2	pseudogene	288	chr17	+
TCONS_00016430	XLOC_007796	LincRNAs identified	2713	chr9	-
NR_026905	C17orf69	RefSeq	2477	chr17	+
chr5:3881075-38945	chr5:3881075-38945	LincRNAs identified	13450	chr5	+
ENST00000557524	RP11-895M11.3	GENCODE	303	chr14	+
uc010kvv.3	DJ031144	UCSC_knowngene	1145	chr7	-
ENST00000441442	SGOL1-AS1	GENCODE	499	chr3	+
ENST00000441442	SGOL1-AS1	GENCODE	499	chr3	+
ENST00000441442	SGOL1-AS1	GENCODE	499	chr3	+
ENST00000441442	SGOL1-AS1	GENCODE	499	chr3	+
ENST00000441442	SGOL1-AS1	GENCODE	499	chr3	+
ENST00000441442	SGOL1-AS1	GENCODE	499	chr3	+
ENST00000441442	SGOL1-AS1	GENCODE	499	chr3	+
ENST00000441442	SGOL1-AS1	GENCODE	499	chr3	+
ENST00000441442	SGOL1-AS1	GENCODE	499	chr3	+
ENST00000441442	SGOL1-AS1	GENCODE	499	chr3	+
ENST00000441442	SGOL1-AS1	GENCODE	499	chr3	+
ENST00000441442	SGOL1-AS1	GENCODE	499	chr3	+
ENST00000441442	SGOL1-AS1	GENCODE	499	chr3	+
ENST00000560967	RP11-109D20.2	GENCODE	426	chr15	-
TCONS_00008256	XLOC_003694	LincRNAs identified	435	chr4	+
TCONS_00018521	XLOC_008828	LincRNAs identified	354	chr10	-
TCONS_00016481	XLOC_007860	LincRNAs identified	653	chr9	-
NR_038904	LOC100506305	RefSeq	1576	chr11	-
ENST00000523190	PVT1	GENCODE	443	chr8	+
AK025564		NRED	2233	chr19	-
ENST00000514010	RP11-93K22.13	GENCODE	713	chr3	-
ENST00000427300	RP11-429G19.2	GENCODE	389	chr10	-
ENST00000558419	CTD-2008A1.1	GENCODE	424	chr15	+
NR_038361	RSBN1L-AS1	RefSeq	1978	chr7	-

144717494	144761233		CTCCTCTACGGTCACTAAAAATATA		intergenic
40929990	40932436		CTATGGTCTTAGATTTCCCAAGAAT		intergenic
50618047	50656139	ENST00000235290,	CCATTTTATTACT	8847	bidirectional
13001584	13011084	NR_038904	ATTTCTACAAATATTTCGACATCTCC		intergenic
123555828	123560910		ATAAAGCAAGGTGACTTTCTGCAGA		bidirectional
7588577	7589689		TTGTCGGAAAAACAATCTACCTGTT		intron sense-overlapp
7588577	7589689		TTGTCGGAAAAACAATCTACCTGTT		intron sense-overlapp
7588577	7589689		TTGTCGGAAAAACAATCTACCTGTT		intron sense-overlapp
7588577	7589689		TTGTCGGAAAAACAATCTACCTGTT		intron sense-overlapp
7588577	7589689		TTGTCGGAAAAACAATCTACCTGTT		intron sense-overlapp
7588577	7589689		TTGTCGGAAAAACAATCTACCTGTT		natural antisense
186652746	186664878		GCTGAAAGGGTGAGCCTGTGGTCTA		natural antisense
62619459	62623360	ENST00000537068	TTTTTCTACTGCT	23642	bidirectional
62619459	62623360	ENST00000537068	TTTTTCTACTGCT	23642	bidirectional
62619459	62623360	ENST00000537068	TTTTTCTACTGCT	23642	bidirectional
21138601	21141197		AACCAAGATCCTTTAAATCCTGATG		natural antisense
83140198	83182930	ENST00000335068,	TAGAACCATTTTC	727849	intergenic
146993468	147032647	NR_033700	CTATTTAAAGGA	2332	exon sense-overlappi
146993468	147032647	NR_033700	CTATTTAAAGGA	2332	exon sense-overlappi
146993468	147032647	NR_033700	CTATTTAAAGGA	2332	exon sense-overlappi
146993468	147032647	NR_033700	CTATTTAAAGGA	2332	exon sense-overlappi
146993468	147032647	NR_033700	CTATTTAAAGGA	2332	exon sense-overlappi
101536823	101537573		GCACTTGGTGGAGATTTATGCCCAT		intergenic
65087687	65122672		GTGTCCTCAGGGATATCACATGAAA		intronic antisense
47631516	47672804		TGGGACATTCTGGCAAATTCTGACT		intergenic
75803626	75806523		TTATGGGATGGAGACGCTGACAGGC		intergenic
3181757	3184857		CAACTAGTAGATTTGGACATTTACA		intronic antisense
31460855	31464814		ACAGTCACAGTTATGTCTCTGAGCT		intergenic
3944578	3957090		TTTGCAGAAGGCCAGGCCAGGATCA		intergenic
134907272	134952977		CCCACCTTGGCTTGTTTGACCACTTA		natural antisense
134907272	134952977		CCCACCTTGGCTTGTTTGACCACTTA		natural antisense
134907272	134952977		CCCACCTTGGCTTGTTTGACCACTTA		natural antisense
43710414	43713807		TACTTGTTAGAGGACGATGAGCTCAGAAATGCAGTTTT		
100164457	100174237		CTGCTATTGTAAGCTTTCTTTAGGGA		natural antisense
43716340	43719671	ENST00000580655	CGAGGCATGTGT	147081	intron sense-overlapp
3828075	3841525		GTTTTCTACATTGACCATGCATCTTC		intergenic
91884609	91887520		GCTCAAGGTGATGCATGTAAAAAGA		bidirectional
30617110	30634419	ENST00000578994,	TCGCCGCCGCAGATTCGACACAAAA		natural antisense
20215777	20227607		CTTGCTCCATTGA	100874028	natural antisense
20215777	20227607		CTTGCTCCATTGA	100874028	natural antisense
20215777	20227607		CTTGCTCCATTGA	100874028	natural antisense
20215777	20227607		CTTGCTCCATTGA	100874028	natural antisense
20215777	20227607		CTTGCTCCATTGA	100874028	natural antisense
20215777	20227607		CTTGCTCCATTGA	100874028	natural antisense
20215777	20227607		CTTGCTCCATTGA	100874028	natural antisense
20215777	20227607		CTTGCTCCATTGA	100874028	natural antisense
20215777	20227607		CTTGCTCCATTGA	100874028	natural antisense
20215777	20227607		CTTGCTCCATTGA	100874028	natural antisense
20215777	20227607		CTTGCTCCATTGA	100874028	natural antisense
20215777	20227607		CTTGCTCCATTGA	100874028	natural antisense
20215777	20227607		CTTGCTCCATTGA	100874028	natural antisense
45365689	45366246	ENST00000558419	CTCAATGAAGGCACACCAATTCCTC		natural antisense
135548787	135561139		TGAACTGCAGATACAGACAACTACA		intergenic
57911541	57914731		ATCAACACAGAAGCAGAAAGGGTCC		intergenic
131097665	131100554		TATGCACACCAGGATTCATTCAAGG		intergenic
13001080	13011095		TACCAAGAGAGG	100506305	intergenic
129021903	129113343		TTTAACCTGTCAT	5820	intergenic
12537720	12539934		CACTTTTTGTCCCCTGCTAGCCTTTT		intergenic
129808275	129813147		ACACCTCAGGAAATTTCCCCTAAAG		intronic antisense
97664412	97667640		CCAACTTAACATCAGCAGATATCCT		bidirectional
45118737	45119292	ENST00000560967	CTCAATGAAGGCACACCAATTCCTC		intergenic
77313167	77326662		TCTTAACTTGAAA	100505854	natural antisense

ENST00000378180	DLEU1	deleted in lymphocyt	+	50656306	50679433
NM_012164	FBXW2	F-box/WD repeat-co	-	123519253	123555740
NM_000546	TP53	cellular tumor antige	-	7571719	7590868
NM_001126112	TP53	cellular tumor antige	-	7571719	7590868
NM_001126113	TP53	cellular tumor antige	-	7571719	7590868
NM_001126114	TP53	cellular tumor antige	-	7571719	7590868
NM_001126118	TP53	cellular tumor antige	-	7571719	7590868
NM_001143990	WRAP53	telomerase Cajal bod	+	7589388	7606820
NM_173651	FSIP2	fibrous sheath-intera	+	186603354	186698016
NM_001012662	SLC3A2	4F2 cell-surface anti	+	62623483	62656355
NM_001012664	SLC3A2	4F2 cell-surface anti	+	62623483	62656355
NM_002394	SLC3A2	4F2 cell-surface anti	+	62623483	62656355
ENST00000382778	IFT88	intraflagellar transpo	+	21140584	21265501
NM_001185075	FMR1	fragile X mental reta	+	146993468	147032647
NM_001185076	FMR1	fragile X mental reta	+	146993468	147032647
NM_001185081	FMR1	fragile X mental reta	+	146993468	147032647
NM_001185082	FMR1	fragile X mental reta	+	146993468	147032647
NM_002024	FMR1	fragile X mental reta	+	146993468	147032647
NM_001142800	EYS	protein eyes shut hon	-	64429875	66417118
ENST00000573771	ZNF213	zinc finger protein 21	+	3179777	3187435
NM_001253881	MED27	mediator of RNA pol	-	134735496	134955274
NM_001253882	MED27	mediator of RNA pol	-	134948737	134955274
NM_004269	MED27	mediator of RNA pol	-	134735496	134955274
'ATTGGTATTTGCCAATAAACAA					
ENST00000355295	TDRD7	tudor domain contair	+	100174231	100258407
NM_001256299	CRHR1	corticotropin-releasir	+	43697709	43913194
NM_001080414	CCDC88C	protein Daple	-	91737666	91884188
NM_002047	GARS	glycine--tRNA ligase	+	30634180	30673648
NM_001012409	SGOL1	shugoshin-like 1 isof	-	20209935	20227698
NM_001012410	SGOL1	shugoshin-like 1 isof	-	20202084	20227698
NM_001012411	SGOL1	shugoshin-like 1 isof	-	20209935	20227698
NM_001012412	SGOL1	shugoshin-like 1 isof	-	20202084	20227698
NM_001012413	SGOL1	shugoshin-like 1 isof	-	20209935	20227698
NM_001199251	SGOL1	shugoshin-like 1 isof	-	20209935	20227724
NM_001199252	SGOL1	shugoshin-like 1 isof	-	20202084	20227724
NM_001199253	SGOL1	shugoshin-like 1 isof	-	20209935	20227724
NM_001199254	SGOL1	shugoshin-like 1 isof	-	20202084	20227724
NM_001199255	SGOL1	shugoshin-like 1 isof	-	20209935	20227724
NM_001199256	SGOL1	shugoshin-like 1 isof	-	20202084	20227724
NM_001199257	SGOL1	shugoshin-like 1 isof	-	20202084	20227724
NM_138484	SGOL1	shugoshin-like 1 isof	-	20202084	20227698
NM_003104	SORD	sorbitol dehydrogena	+	45315301	45367287
NM_001136152	ALG1L2	putative glycosyltran	+	129800673	129817233
NM_001130446	C10orf131	uncharacterized prot	+	97667721	97698415
NM_198467	RSBN1L	round spermatid basi	+	77325742	77409120

35.214154	11.525106	4.811619	3.430609	32.677975	31.839376
159.966273	26.919288	6.91123	5.026881	181.21497	205.3259
777.862183	120.96903	8.979646	7.208258	1117.6858	673.8493
725.222087	155.398835	8.714629	7.599995	322.4779	258.48215
32.660173	7.919386	4.689986	3.003063	34.082867	36.12547
85.039293	18.585567	5.895839	4.302219	59.945915	90.850716
85.039293	18.585567	5.895839	4.302219	59.945915	90.850716
85.039293	18.585567	5.895839	4.302219	59.945915	90.850716
85.039293	18.585567	5.895839	4.302219	59.945915	90.850716
85.039293	18.585567	5.895839	4.302219	59.945915	90.850716
85.039293	18.585567	5.895839	4.302219	59.945915	90.850716
1733.54805	267.87987	10.339007	8.28613	2038.8013	1606.9202
91.98084	19.975788	6.126249	4.537163	102.8817	120.08152
91.98084	19.975788	6.126249	4.537163	102.8817	120.08152
91.98084	19.975788	6.126249	4.537163	102.8817	120.08152
126.053971	33.927447	6.533275	5.391685	95.46774	105.130104
67.540644	14.243212	5.623607	4.036266	66.067566	100.18979
26.561313	8.489266	4.246964	3.223547	28.702147	11.84721
26.561313	8.489266	4.246964	3.223547	28.702147	11.84721
26.561313	8.489266	4.246964	3.223547	28.702147	11.84721
26.561313	8.489266	4.246964	3.223547	28.702147	11.84721
26.561313	8.489266	4.246964	3.223547	28.702147	11.84721
552.058505	171.759516	8.665014	7.614237	732.4412	822.4888
49.564804	14.882496	5.283039	4.145473	43.556225	36.124966
71.996643	12.315012	5.765598	3.872813	58.58219	39.66317
52.047823	8.163056	5.350857	3.121765	50.53604	40.976715
58.344822	15.69181	5.529417	3.756834	55.191296	49.935024
908.41932	281.556713	9.410593	8.376212	805.37866	936.11163
335.60206	96.230324	7.951415	6.806371	318.4233	462.98273
48.789492	13.847882	5.26998	4.016883	51.93067	40.763443
48.789492	13.847882	5.26998	4.016883	51.93067	40.763443
48.789492	13.847882	5.26998	4.016883	51.93067	40.763443
19.361174	5.768047	3.95436	2.65778	17.67862	16.75165
26.206528	6.792963	4.308914	2.744599	25.76184	25.489061
197.931067	56.561523	7.256539	6.102774	183.71991	162.23677
376.747014	109.715092	7.925993	6.877649	398.16464	403.00702
61.527219	8.236671	5.494593	3.309876	87.07647	91.07996
3434.8336	1053.23925	11.303399	10.267745	3694.9553	3351.5203
29.052032	5.48008	4.333255	2.553508	32.767464	44.127274
29.052032	5.48008	4.333255	2.553508	32.767464	44.127274
29.052032	5.48008	4.333255	2.553508	32.767464	44.127274
29.052032	5.48008	4.333255	2.553508	32.767464	44.127274
29.052032	5.48008	4.333255	2.553508	32.767464	44.127274
29.052032	5.48008	4.333255	2.553508	32.767464	44.127274
29.052032	5.48008	4.333255	2.553508	32.767464	44.127274
29.052032	5.48008	4.333255	2.553508	32.767464	44.127274
29.052032	5.48008	4.333255	2.553508	32.767464	44.127274
29.052032	5.48008	4.333255	2.553508	32.767464	44.127274
29.052032	5.48008	4.333255	2.553508	32.767464	44.127274
29.052032	5.48008	4.333255	2.553508	32.767464	44.127274
29.052032	5.48008	4.333255	2.553508	32.767464	44.127274
89.461293	17.29389	6.11761	4.344825	99.038864	114.3809
17.770083	6.675336	3.7524	2.722883	13.102758	9.707878
1942.424708	569.826215	10.417826	9.314421	2030.4451	2338.8562
220.491889	71.30377	7.310573	6.303718	266.2046	310.09274
446.988548	33.413465	7.318303	5.325092	70.88052	56.93192
144.816597	30.183999	6.750347	5.191719	128.61432	113.77998
280.35939	83.59366	7.72697	6.627783	310.83466	345.7152
31.597298	7.700948	4.622252	3.024856	39.890507	40.728577
53.930757	10.425199	5.419357	3.570167	49.674557	53.85292
69.43471	9.512859	5.752351	3.490425	75.50519	87.86358
11662.64767	3374.618167	13.021415	11.995308	14408.794	16862.91

38.62145	34.907692	31.015806	42.222622	15.1396	4.9999995
125.09517	90.55117	162.63428	194.97615	17.835352	19.554758
1624.8373	296.0423	451.34583	503.41257	130.77158	90.68517
320.61594	1815.4852	441.96063	1192.3107	134.7192	105.25419
27.6032	43.47433	21.48139	33.193783	6.0293465	4.9999995
29.631329	137.56677	62.938736	129.30229	20.20381	6.85913
29.631329	137.56677	62.938736	129.30229	20.20381	6.85913
29.631329	137.56677	62.938736	129.30229	20.20381	6.85913
29.631329	137.56677	62.938736	129.30229	20.20381	6.85913
29.631329	137.56677	62.938736	129.30229	20.20381	6.85913
29.631329	137.56677	62.938736	129.30229	20.20381	6.85913
2143.0698	1538.4971	1326.296	1747.7039	339.45755	174.49542
84.13848	52.26645	95.532486	96.984406	27.60205	24.18302
84.13848	52.26645	95.532486	96.984406	27.60205	24.18302
84.13848	52.26645	95.532486	96.984406	27.60205	24.18302
88.609764	82.30874	195.36636	189.44112	30.612692	27.755743
27.773582	53.011303	81.6944	76.50722	13.21594	8.337841
44.172	12.003471	31.07679	31.56626	10.53886	7.758108
44.172	12.003471	31.07679	31.56626	10.53886	7.758108
44.172	12.003471	31.07679	31.56626	10.53886	7.758108
44.172	12.003471	31.07679	31.56626	10.53886	7.758108
44.172	12.003471	31.07679	31.56626	10.53886	7.758108
478.3223	383.48615	429.3118	466.30078	207.88712	94.410934
55.74475	45.83562	56.780003	59.34726	13.287508	10.01587
63.761772	66.14074	100.82228	103.009705	11.908272	10.88523
57.101295	64.0626	36.605762	63.004528	9.052318	4.9999995
58.91923	67.962685	54.08971	63.970985	19.369308	4.9999995
603.48737	942.05176	963.7465	1199.74	242.00233	111.88697
174.67233	384.4921	270.79022	402.25168	62.907978	86.65958
43.638348	50.046272	46.37859	59.97963	13.348359	6.373055
43.638348	50.046272	46.37859	59.97963	13.348359	6.373055
43.638348	50.046272	46.37859	59.97963	13.348359	6.373055
27.254744	19.798153	16.5556	18.128279	7.5690455	4.9999995
10.67823	37.054207	24.669281	33.58655	4.9999995	4.9999995
180.82404	192.03362	208.84396	259.9281	46.25832	29.479496
424.47565	601.1502	61.695137	371.98944	128.83508	88.677086
75.65405	39.0675	37.96976	38.315575	9.73746	12.370708
4530.763	2348.7207	3067.1736	3615.8687	1404.6125	620.5423
12.49218	12.354619	35.01399	37.556667	4.9999995	4.9999995
12.49218	12.354619	35.01399	37.556667	4.9999995	4.9999995
12.49218	12.354619	35.01399	37.556667	4.9999995	4.9999995
12.49218	12.354619	35.01399	37.556667	4.9999995	4.9999995
12.49218	12.354619	35.01399	37.556667	4.9999995	4.9999995
12.49218	12.354619	35.01399	37.556667	4.9999995	4.9999995
12.49218	12.354619	35.01399	37.556667	4.9999995	4.9999995
12.49218	12.354619	35.01399	37.556667	4.9999995	4.9999995
12.49218	12.354619	35.01399	37.556667	4.9999995	4.9999995
12.49218	12.354619	35.01399	37.556667	4.9999995	4.9999995
12.49218	12.354619	35.01399	37.556667	4.9999995	4.9999995
12.49218	12.354619	35.01399	37.556667	4.9999995	4.9999995
12.49218	12.354619	35.01399	37.556667	4.9999995	4.9999995
72.86008	79.648674	73.920586	96.918655	18.877626	11.71744
14.673321	17.20808	27.481491	24.44697	4.9999995	4.9999995
1286.5386	922.80505	2586.7031	2489.2002	601.15894	241.49759
119.470276	111.88331	279.93835	235.36206	89.511086	45.253128
83.48965	1546.6577	174.7608	749.2107	30.510752	27.067291
140.02238	242.45789	76.58469	167.44032	27.010221	19.00276
188.6121	198.68286	324.39935	313.91217	93.43223	67.52546
29.127235	30.168936	18.223587	31.444946	5.6409926	4.9999995
51.65554	65.66558	47.827522	54.90842	6.150355	5.990272
54.301167	77.685295	49.74141	71.51162	9.183686	5.8305683
11277.358	10727.666	5889.516	10809.642	4224.381	1779.3439

21.982897	4.9999995	15.702829	6.325308	4.568616	4.919657
26.298046	35.70093	35.64865	26.477991	7.0423045	7.430937
190.69514	96.87471	125.76648	91.0211	9.650539	9.071569
153.91899	231.52855	154.26456	152.70752	7.875224	7.7494173
14.6394	4.9999995	11.2187395	5.628828	4.6286354	5.08421
36.84016	11.13368	21.818712	14.657911	5.4473157	6.29945
36.84016	11.13368	21.818712	14.657911	5.4473157	6.29945
36.84016	11.13368	21.818712	14.657911	5.4473157	6.29945
36.84016	11.13368	21.818712	14.657911	5.4473157	6.29945
36.84016	11.13368	21.818712	14.657911	5.4473157	6.29945
36.84016	11.13368	21.818712	14.657911	5.4473157	6.29945
525.3861	177.24565	235.7582	154.9363	10.51125	10.260273
20.32221	10.775821	24.154358	12.817269	6.237662	6.6811523
20.32221	10.775821	24.154358	12.817269	6.237662	6.6811523
20.32221	10.775821	24.154358	12.817269	6.237662	6.6811523
44.94838	32.23706	36.091278	31.919529	6.1300116	6.4992404
23.81825	11.54364	16.88597	11.65763	5.586079	6.4325547
13.508468	4.9999995	9.130159	4.9999995	4.3908386	3.6239464
13.508468	4.9999995	9.130159	4.9999995	4.3908386	3.6239464
13.508468	4.9999995	9.130159	4.9999995	4.3908386	3.6239464
13.508468	4.9999995	9.130159	4.9999995	4.3908386	3.6239464
13.508468	4.9999995	9.130159	4.9999995	4.3908386	3.6239464
357.68927	103.8066	130.1859	136.57727	9.0504	9.352016
22.25389	15.526158	15.491269	12.720281	4.987628	5.0840874
13.89715	13.29468	6.6816907	17.223051	5.410706	5.206507
9.247421	6.8371196	13.529529	5.311949	5.1946907	5.2489533
40.339565	6.2938876	14.595338	8.552764	5.3203783	5.507521
444.36572	219.5291	415.26746	256.2887	9.192798	9.53218
38.2157	132.97672	148.72896	107.893005	7.860411	8.54518
16.08807	17.8038	13.437681	16.03633	5.23283	5.242158
16.08807	17.8038	13.437681	16.03633	5.23283	5.242158
16.08807	17.8038	13.437681	16.03633	5.23283	5.242158
5.6811676	4.9999995	6.358071	4.9999995	3.6750293	4.075333
4.9999995	4.9999995	14.785301	5.972479	4.229718	4.6237307
65.97162	63.947475	74.24178	59.47045	7.064236	7.098665
227.23242	52.73336	38.243107	122.569496	8.181606	8.351657
6.142438	6.7910376	7.50134	6.877045	5.990756	6.302118
2148.386	676.81665	800.25275	668.8253	11.371436	11.295942
7.042278	4.9999995	5.838204	4.9999995	4.5733356	5.3475575
7.042278	4.9999995	5.838204	4.9999995	4.5733356	5.3475575
7.042278	4.9999995	5.838204	4.9999995	4.5733356	5.3475575
7.042278	4.9999995	5.838204	4.9999995	4.5733356	5.3475575
7.042278	4.9999995	5.838204	4.9999995	4.5733356	5.3475575
7.042278	4.9999995	5.838204	4.9999995	4.5733356	5.3475575
7.042278	4.9999995	5.838204	4.9999995	4.5733356	5.3475575
7.042278	4.9999995	5.838204	4.9999995	4.5733356	5.3475575
7.042278	4.9999995	5.838204	4.9999995	4.5733356	5.3475575
7.042278	4.9999995	5.838204	4.9999995	4.5733356	5.3475575
7.042278	4.9999995	5.838204	4.9999995	4.5733356	5.3475575
7.042278	4.9999995	5.838204	4.9999995	4.5733356	5.3475575
7.042278	4.9999995	5.838204	4.9999995	4.5733356	5.3475575
26.132206	11.76667	19.274958	15.99444	6.17936	6.615792
13.2753315	4.9999995	6.7766843	4.9999995	3.229991	3.3648887
1339.2848	431.76392	438.17517	367.07687	10.5040245	10.7691555
133.764	34.077045	83.64028	41.57708	7.599098	8.002637
29.60438	56.70821	19.6527	36.937458	5.689101	5.6761985
33.733986	38.77797	21.920176	40.65888	6.5566463	6.6095557
140.39465	45.15994	88.78058	66.2691	7.8239183	8.151571
8.481638	4.9999995	12.38543	9.69763	4.857488	5.2411833
13.989302	13.988781	13.600181	8.832303	5.1729603	5.608648
12.31349	9.529158	11.70712	8.51313	5.7838755	6.253922
6371.0557	2005.5336	3643.562	2223.8328	13.352356	13.588936

4.957791	4.6899266	4.812683	4.921042	4.1552176	2.4279919
6.6453767	6.1481705	7.1162405	7.084349	4.3935523	5.114909
10.299192	7.8950005	8.559589	8.401984	7.260927	7.352209
7.987714	10.507644	8.528748	9.639024	7.3036885	7.562488
4.477397	5.0313506	4.3215647	4.5967603	2.794245	2.4279919
4.5791698	6.7763214	5.784082	6.488696	4.570642	3.5400226
4.5791698	6.7763214	5.784082	6.488696	4.570642	3.5400226
4.5791698	6.7763214	5.784082	6.488696	4.570642	3.5400226
4.5791698	6.7763214	5.784082	6.488696	4.570642	3.5400226
4.5791698	6.7763214	5.784082	6.488696	4.570642	3.5400226
4.5791698	6.7763214	5.784082	6.488696	4.570642	3.5400226
10.709379	10.275186	10.097441	10.180511	8.676286	8.289914
6.0775433	5.315853	6.366231	6.079051	5.0224605	5.4292765
6.0775433	5.315853	6.366231	6.079051	5.0224605	5.4292765
6.0775433	5.315853	6.366231	6.079051	5.0224605	5.4292765
6.1507096	6.004851	7.373605	7.041234	5.1691947	5.6294403
4.4860234	5.3401175	6.1483808	5.748485	3.9516587	3.8286333
5.145861	2.9790778	4.817106	4.5249524	3.624174	3.7179737
5.145861	2.9790778	4.817106	4.5249524	3.624174	3.7179737
5.145861	2.9790778	4.817106	4.5249524	3.624174	3.7179737
5.145861	2.9790778	4.817106	4.5249524	3.624174	3.7179737
5.145861	2.9790778	4.817106	4.5249524	3.624174	3.7179737
8.550504	8.261265	8.486896	8.289	7.9453964	7.4149513
5.48584	5.1134753	5.6403193	5.386883	3.9607365	4.106607
5.6899724	5.676843	6.4422555	6.1673064	3.8031375	4.233918
5.521658	5.6283197	5.0410547	5.470466	3.3934383	2.4279919
5.565819	5.720097	5.5739594	5.4887266	4.5121126	2.4279919
8.868992	9.561344	9.661961	9.646286	8.175813	7.6548643
7.1191206	8.266245	7.83777	8.079763	6.2093406	7.286792
5.1239724	5.25235	5.3669186	5.401653	3.965902	3.4244344
5.1239724	5.25235	5.3669186	5.401653	3.965902	3.4244344
5.1239724	5.25235	5.3669186	5.401653	3.965902	3.4244344
4.4604807	3.7839713	3.9721143	3.759234	3.1156545	2.4279919
3.0958815	4.779579	4.51231	4.612265	2.3467326	2.4279919
7.171873	7.264973	7.465314	7.474171	5.7603602	5.721699
8.383337	8.913827	5.7541656	7.971365	7.240632	7.3184557
5.9241323	4.8660374	5.0942035	4.79031	3.5066657	4.424124
11.794888	10.862106	11.289319	11.206703	10.743273	10.139626
3.3138227	3.0228915	4.9783998	4.763521	2.3467326	2.4279919
3.3138227	3.0228915	4.9783998	4.763521	2.3467326	2.4279919
3.3138227	3.0228915	4.9783998	4.763521	2.3467326	2.4279919
3.3138227	3.0228915	4.9783998	4.763521	2.3467326	2.4279919
3.3138227	3.0228915	4.9783998	4.763521	2.3467326	2.4279919
3.3138227	3.0228915	4.9783998	4.763521	2.3467326	2.4279919
3.3138227	3.0228915	4.9783998	4.763521	2.3467326	2.4279919
3.3138227	3.0228915	4.9783998	4.763521	2.3467326	2.4279919
3.3138227	3.0228915	4.9783998	4.763521	2.3467326	2.4279919
3.3138227	3.0228915	4.9783998	4.763521	2.3467326	2.4279919
3.3138227	3.0228915	4.9783998	4.763521	2.3467326	2.4279919
3.3138227	3.0228915	4.9783998	4.763521	2.3467326	2.4279919
3.3138227	3.0228915	4.9783998	4.763521	2.3467326	2.4279919
5.873641	5.955542	6.002578	6.078749	4.4733043	4.342244
3.5508687	3.55852	4.6517677	4.1583643	2.3467326	2.4279919
9.965419	9.528695	11.060142	10.6795225	9.512833	8.766824
6.579909	6.463395	7.8839846	7.334412	6.712404	6.347512
6.066306	10.288283	7.2221823	8.967747	5.1645646	5.593311
6.8056498	7.6127796	6.0544424	6.8630075	4.9938626	5.071493
7.2364316	7.3138404	8.090627	7.745432	6.777698	6.9317765
4.5560927	4.463447	4.0954337	4.519867	2.6997633	2.4279919
5.373929	5.6655035	5.408334	5.2867675	2.823394	3.3299453
5.44571	5.917599	5.4633427	5.649657	3.4122398	3.2852955
13.140427	13.043523	12.220333	12.782915	12.351572	11.689759

4.4922643	2.3408759	4.2439847	2.923317
4.7402105	5.389193	5.431282	5.0921373
7.524301	6.9309874	7.22117	6.9599533
7.21432	8.267684	7.497245	7.7545447
3.9380352	2.3408759	3.7627664	2.754466
5.205836	3.5887544	4.7203903	4.1876693
5.205836	3.5887544	4.7203903	4.1876693
5.205836	3.5887544	4.7203903	4.1876693
5.205836	3.5887544	4.7203903	4.1876693
5.205836	3.5887544	4.7203903	4.1876693
5.205836	3.5887544	4.7203903	4.1876693
8.991232	7.8490705	8.133397	7.7768784
4.386918	3.5361571	4.863917	3.9842474
4.386918	3.5361571	4.863917	3.9842474
4.386918	3.5361571	4.863917	3.9842474
5.487895	5.222901	5.4486933	5.3919883
4.601135	3.6425295	4.3529983	3.84064
3.830193	2.3408759	3.4753118	2.352752
3.830193	2.3408759	3.4753118	2.352752
3.830193	2.3408759	3.4753118	2.352752
3.830193	2.3408759	3.4753118	2.352752
3.830193	2.3408759	3.4753118	2.352752
8.428124	7.043944	7.269566	7.583443
4.509212	4.098588	4.2257657	3.9719284
3.8703914	3.8547552	3.0454931	4.429181
3.3273416	2.8709912	4.030385	2.680445
5.332212	2.7567475	4.139688	3.3722532
8.755931	8.18761	8.940363	8.542692
5.2580667	7.4200006	7.4430404	7.220987
4.0679245	4.2987247	4.020419	4.3238955
4.0679245	4.2987247	4.020419	4.3238955
4.0679245	4.2987247	4.020419	4.3238955
2.7256498	2.3408759	2.9837534	2.352752
2.350709	2.3408759	4.1579375	2.8433492
6.022262	6.300123	6.475262	6.336935
7.7707605	5.9977736	5.525881	7.4123926
2.8164632	2.8642077	3.207969	3.0398293
11.0278225	9.872683	9.877325	9.94574
2.978301	2.3408759	2.8743918	2.352752
2.978301	2.3408759	2.8743918	2.352752
2.978301	2.3408759	2.8743918	2.352752
2.978301	2.3408759	2.8743918	2.352752
2.978301	2.3408759	2.8743918	2.352752
2.978301	2.3408759	2.8743918	2.352752
2.978301	2.3408759	2.8743918	2.352752
2.978301	2.3408759	2.8743918	2.352752
2.978301	2.3408759	2.8743918	2.352752
2.978301	2.3408759	2.8743918	2.352752
2.978301	2.3408759	2.8743918	2.352752
2.978301	2.3408759	2.8743918	2.352752
2.978301	2.3408759	2.8743918	2.352752
4.7304535	3.6677918	4.5354285	4.3197255
3.8056703	2.3408759	3.0632725	2.352752
10.32376	9.204118	9.014928	9.064061
7.019251	5.3120155	6.6404076	5.7907205
4.9066095	6.111949	4.560942	5.6131773
5.088114	5.513756	4.7270536	5.756035
7.0885835	5.7486734	6.7256374	6.494332
3.2108774	2.3408759	3.9059365	3.563689
3.8783271	3.9335487	4.0355835	3.4202032
3.707674	3.3486922	3.8246913	3.363959
12.602959	11.47612	12.117373	11.734064

ASHGA5P053396	0.005768145	0.048278925	2.9249335	up	noncoding
ASHGA5P048257	0.005109564	0.04540115	3.5042899	up	noncoding
ASHGA5P045554	0.010055594	0.062161527	2.2073697	up	noncoding
ASHGA5P019706	0.001317953	0.024205782	16.817697	up	noncoding
ASHGA5P015915	0.000313576	0.012923062	4.3265009	up	noncoding
ASHGA5P019601	3.63296E-05	0.006164999	2.5049282	up	noncoding
ASHGA5P019601	3.63296E-05	0.006164999	2.5049282	up	noncoding
ASHGA5P051021	0.000171295	0.011182026	3.2402254	up	noncoding
ASHGA5P022628	0.005151509	0.04558664	2.0127852	up	noncoding
ASHGA5P033046	0.021369189	0.090089027	4.363316	up	noncoding
ASHGA5P033447	0.000420016	0.014598519	2.3552318	up	noncoding
ASHGA5P030819	0.009021594	0.059313506	2.1568351	up	noncoding
ASHGA5P041845	0.000813142	0.019071379	2.0857918	up	noncoding
ASHGA5P041845	0.000813142	0.019071379	2.0857918	up	noncoding
ASHGA5P017056	0.038825351	0.12556577	2.6700308	up	noncoding
ASHGA5P041149	8.81954E-06	0.004010044	3.1256532	up	noncoding
ASHGA5P047835	0.022410242	0.092621507	2.4296065	up	noncoding
ASHGA5P022933	0.016453163	0.078324477	3.2871146	up	noncoding
ASHGA5P022933	0.016453163	0.078324477	3.2871146	up	noncoding
ASHGA5P058457	0.000144907	0.010718755	2.5287809	up	noncoding
ASHGA5P037280	0.000133243	0.010628132	3.6460353	up	noncoding
ASHGA5P037280	0.000133243	0.010628132	3.6460353	up	noncoding
ASHGA5P029480	0.008263135	0.056914687	2.3179226	up	noncoding
ASHGA5P051222	0.005008527	0.044965036	2.0885861	up	noncoding
ASHGA5P033345	0.048692548	0.143360998	3.1099446	up	noncoding
ASHGA5P021293	0.014020963	0.072574415	2.5739962	up	noncoding
ASHGA5P036274	0.021112237	0.089478872	2.2220884	up	noncoding
ASHGA5P036274	0.021112237	0.089478872	2.2220884	up	noncoding
ASHGA5P036274	0.021112237	0.089478872	2.2220884	up	noncoding
ASHGA5P027738	0.014634741	0.073959919	3.4482127	up	noncoding
ASHGA5P016120	0.027652884	0.103853965	2.0215157	up	noncoding
ASHGA5P018491	0.025163821	0.098610046	2.0813683	up	noncoding
ASHGA5P031329	0.006185487	0.050189379	2.621886	up	noncoding
ASHGA5P019192	0.012303488	0.067865226	2.3459187	up	noncoding
ASHGA5P037758	0.019511292	0.085195149	4.4025163	up	noncoding
ASHGA5P032010	0.002222796	0.031049607	5.3993182	up	noncoding
ASHGA5P045188	0.032907179	0.114192132	2.9110999	up	noncoding
ASHGA5P046991	0.013623808	0.071680418	2.0512483	up	noncoding
ASHGA5P027686	0.005000699	0.044918059	3.3862048	up	noncoding
ASHGA5P045033	0.00012043	0.010119041	9.0292075	up	noncoding
ASHGA5P032772	0.030805545	0.110259134	7.8136614	up	noncoding
ASHGA5P038918	0.00078566	0.018693364	3.1318515	up	noncoding
ASHGA5P038918	0.00078566	0.018693364	3.1318515	up	noncoding
ASHGA5P038918	0.00078566	0.018693364	3.1318515	up	noncoding
ASHGA5P000216	0.015677994	0.076574828	2.2219408	up	noncoding
ASHGA5P055810	0.003769271	0.039397533	2.2652929	up	noncoding
ASHGA5P055810	0.003769271	0.039397533	2.2652929	up	noncoding
ASHGA5P015787	0.000260474	0.012185279	3.7150277	up	noncoding
ASHGA5P019801	0.007345661	0.053840332	2.1619951	up	noncoding
ASHGA5P037833	0.003637817	0.038710442	2.3163047	up	noncoding
ASHGA5P031493	0.001936605	0.029326945	3.7421899	up	noncoding
ASHGA5P019644	0.001491879	0.025745699	2.2582985	up	noncoding
ASHGA5P019644	0.001491879	0.025745699	2.2582985	up	noncoding
ASHGA5P038691	0.000668148	0.017209782	3.3484459	up	noncoding
ASHGA5P022820	0.00831254	0.056954491	4.7218036	up	noncoding
ASHGA5P022820	0.00831254	0.056954491	4.7218036	up	noncoding
ASHGA5P039927	0.010392084	0.062967308	2.733505	up	noncoding
ASHGA5P006918	0.00033011	0.013196023	2.4848971	up	noncoding
ASHGA5P023069	0.022934567	0.093794227	2.0286676	up	noncoding
ASHGA5P023069	0.022934567	0.093794227	2.0286676	up	noncoding
ASHGA5P023069	0.022934567	0.093794227	2.0286676	up	noncoding
ASHGA5P018708	0.000303249	0.012633464	3.9398001	up	noncoding

ENST00000454813	GAS5	GENCODE	621	chr1	-
ENST00000528089	FAM86C2P	GENCODE	2496	chr11	-
NR_003672	SNHG7	RefSeq	2176	chr9	-
uc010vhb.2	CRNDE	UCSC_knowngene	838	chr16	-
ENST00000424756	LINC00539	GENCODE	347	chr13	+
NR_048556	TSN	RefSeq	3314	chr2	+
NR_048556	TSN	RefSeq	3314	chr2	+
ENST00000428008	ZNFX1-AS1	GENCODE	566	chr20	+
ENST00000559979	RP11-17L5.4	GENCODE	558	chr15	-
ENST00000577546	RP11-118G23.2	GENCODE	307	chr17	+
ENST00000571113	RP11-497H17.1	GENCODE	504	chr17	+
ENST00000420498	RP11-297K8.2	GENCODE	379	chr1	-
uc003owm.1	AK024736	UCSC_knowngene	1515	chr6	-
uc003owm.1	AK024736	UCSC_knowngene	1515	chr6	-
ENST00000437157	RP3-510D11.1	GENCODE	548	chr1	-
ENST00000457791	PMCHL2	pseudogene	237	chr5	+
AK056943		LincRNAs identified	1856	chr20	+
ENST00000565764	CTA-445C9.14	GENCODE	981	chr22	+
ENST00000565764	CTA-445C9.14	GENCODE	981	chr22	+
uc003tcy.3	AK057321	UCSC_knowngene	1766	chr7	+
uc002ypm.3	BC034802	UCSC_knowngene	579	chr21	-
uc002ypm.3	BC034802	UCSC_knowngene	579	chr21	-
ENST00000552826	NOVA1-AS1	GENCODE	449	chr14	+
uc021wmd.1	BCR	UCSC_knowngene	1500	chr22	-
TCONS_00028072	XLOC_013814	LincRNAs identified	2067	chr20	-
ENST00000529667	AC005152.2	GENCODE	340	chr17	-
uc002umb.1	BX538254	UCSC_knowngene	1247	chr2	+
uc002umb.1	BX538254	UCSC_knowngene	1247	chr2	+
uc002umb.1	BX538254	UCSC_knowngene	1247	chr2	+
DB336485		LincRNAs identified	589	chrX	+
ENST00000427019	AC113607.3	GENCODE	589	chr2	-
ENST00000454174	RP11-80H5.2	GENCODE	563	chr10	+
TCONS_00006757	XLOC_003387	LincRNAs identified	2081	chr3	-
NR_028444	PDIA5	RefSeq	1717	chr3	+
uc010ofi.1	SFN	UCSC_knowngene	663	chr1	-
NR_036480	LOC100128881	RefSeq	1753	chr16	+
ENST00000424177	RP11-462B18.2	GENCODE	551	chr9	-
TCONS_00015070	XLOC_007146	LincRNAs identified	244	chr8	-
ENST00000545739	RP11-955H22.3	GENCODE	852	chr12	-
ENST00000522414	PVT1	GENCODE	654	chr8	+
ENST00000573270	RP11-141J13.5	GENCODE	1273	chr17	+
uc003dtq.3	AF090939	UCSC_knowngene	2039	chr3	+
uc003dtq.3	AF090939	UCSC_knowngene	2039	chr3	+
uc003dtq.3	AF090939	UCSC_knowngene	2039	chr3	+
chr11:8171725-8184	chr11:8171725-8184	LincRNAs identified	12300	chr11	+
ENST00000568302	RP11-173B14.5	GENCODE	549	chr13	-
ENST00000568302	RP11-173B14.5	GENCODE	549	chr13	-
uc002xij.3	LOC388796	UCSC_knowngene	805	chr20	-
ENST00000503428	RP11-1379J22.5	GENCODE	325	chr5	+
ENST00000429328	PDZK1IP1-AS1	GENCODE	661	chr1	+
ENST00000570700	RP11-473M20.11	GENCODE	302	chr16	+
NR_048558	TSN	RefSeq	3101	chr2	+
NR_048558	TSN	RefSeq	3101	chr2	+
ENST00000430621	AC034193.5	GENCODE	683	chr3	+
ENST00000563284	RP11-304L19.1	GENCODE	501	chr16	+
ENST00000563284	RP11-304L19.1	GENCODE	501	chr16	+
ENST00000441504	DANCR	GENCODE	336	chr4	+
NR_026591	RAD51	RefSeq	4525	chr5	-
ENST00000568971	RP11-626G11.5	GENCODE	360	chr16	-
ENST00000568971	RP11-626G11.5	GENCODE	360	chr16	-
ENST00000568971	RP11-626G11.5	GENCODE	360	chr16	-
ENST00000456651	RP3-522D1.1	GENCODE	587	chr1	+

173833039	173834009	ENST00000412059,	GATTCTGAGGTT/ 60674	intergenic
67559254	67565140	NR_024253	TTTCCATCTAATC 645332	intergenic
139619683	139622636		TGTCAGACTCCT(84973	intergenic
54952777	54962690	ENST00000559432	CTATTGGAAGGA(643911	intergenic
21872277	21878694		GACCGACTTGAAATCAGAAAGGA/	intergenic
122513120	122525428		CATCCGAGAGTT(7247	exon sense-overlappi
122513120	122525428		CATCCGAGAGTT(7247	exon sense-overlappi
47895178	47905797	NR_036659	CTAGTTGCAGTC/ 441951	bidirectional
79529047	79576183		AGTCATTCAGGTTTTCCAGGAGAGA	intronic antisense
32007595	32007902		CCATCTGTGGTACTTGTTAGTTTATI	intronic antisense
80876157	80876661		CGCTGAGCCGCGCGTGCCGTTTTTA	intron sense-overlapp
161123549	161129139		TGTAAACAGATTACTCCGTCCTATA	natural antisense
43964176	44042389		CCTGCTTTGAGGACAGCTGAAGTCT	natural antisense
43964176	44042389		CCTGCTTTGAGGACAGCTGAAGTCT	natural antisense
9228326	9262678		TTTGCGCAGGTGGAGATCTCGCGGG	intergenic
70671656	70672163	ENST00000415808,	GAGAAACTGGGG 5370	
23128722	23130576		AAGGTTCAGATGAGGATGGTAGTTC	intergenic
26908520	26909878		TTTCCAGCAGATTCAAGAGGCAATT	bidirectional
26908520	26909878		TTTCCAGCAGATTCAAGAGGCAATT	bidirectional
32768503	32794385		GCGCATGTTTGGAGACTATATCATC	intron sense-overlapp
33678774	33681045		GAAGGGAACATTGGATGTGTCAGCC	natural antisense
33678774	33681045		GAAGGGAACATTGGATGTGTCAGCC	natural antisense
27106446	27275587		TGAAAATTACTGAGGAAGCCTCTTA	intergenic
21642517	21644017	NM_004327, NM_0:	TCCCCAACAGATCCAGATACCTAAT	intergenic
56130114	56134053		CTCTCTCCCCAGAAGTTTTGTGATTI	intergenic
70067182	70115634		TCAGACACAGGTGAAAACCATCCAT	intergenic
179278389	179298394		TCGTGCTTGGTTTTCCGGACCTTGCI	intronic antisense
179278389	179298394		TCGTGCTTGGTTTTCCGGACCTTGCI	intronic antisense
179278389	179298394		TCGTGCTTGGTTTTCCGGACCTTGCI	intronic antisense
42804381	42804970		AGGCTCTCTCAAATTTTTCTCTAAG/	intergenic
906611	916810		CAAAATACTATGCTGCTGGTGTTAC	intergenic
91405038	91410424		AGAGGTGTCAGGGGATTTGATGCAC	natural antisense
194565037	194567506		TTCATGACCCCAGTATACCTCTCCC/	intergenic
122785855	122880953		TGGCTGAAGAAG 10954	exon sense-overlappi
27189786	27190449		TCTTATAGGCTACTGAGAGCAGGTT	natural antisense
89778263	89784573		TGGTCACAGTCG(100128881	natural antisense
32840595	32841760		CACACAGTAGAATAACATAACTGTA	intergenic
89578174	89600696		ATGTCAGTGGTGATCATTTTACTGA(	intergenic
127808699	127824617		ACATTGAAAAGTTTATCCTCACCCT/	intergenic
129108764	129112133		TAAACATCTTGC(5820	intergenic
4513743	4520945		AGGCCACTTACAGCACTCGAATAAG	intergenic
99652068	99654107		TGCATACTAGGGTAAAGAAAAATTT	intron sense-overlapp
99652068	99654107		TGCATACTAGGGTAAAGAAAAATTT	intronic antisense
99652068	99654107		TGCATACTAGGGTAAAGAAAAATTT	intronic antisense
8215149	8227449		AATTTGTATATTTAATTCTCCCTCTC	intergenic
76178835	76210130		ACTGTTACCTCTAATAAAGTTTCAT(	intronic antisense
76178835	76210130		ACTGTTACCTCTAATAAAGTTTCAT(	natural antisense
37049238	37063962	ENST00000423536,	TGCTTGTAAGGT(388796	intergenic
179085265	179091431		ATGGAACAAAAGGTCGGCTTGAGGI	intergenic
47644921	47646011		TAGTCCTGATCTA 100874253	intergenic
3156764	3159577		CTCATTCTCAAGAAGAAGTTAATGT(	intergenic
122513120	122525428		CATCCGAGAGTT(7247	exon sense-overlappi
122513120	122525428		CATCCGAGAGTT(7247	exon sense-overlappi
10045951	10048674		GAGCCTGCAGCACTTTCCTGCAAAG	natural antisense
2141539	2145426		TGCGCAGAGGGGTCTGGCTGGACTA	natural antisense
2141539	2145426		TGCGCAGAGGGGTCTGGCTGGACTA	natural antisense
53578664	53579296		CGGCTCCCCTTA/ 57291	intergenic
34905365	34915731		TTTTATTCAGGG/ 5810	exon sense-overlappi
19074183	19078963		CCGCGCACCGGAAAAAGCAAAGAG(	natural antisense
19074183	19078963		CCGCGCACCGGAAAAAGCAAAGAG(	natural antisense
19074183	19078963		CCGCGCACCGGAAAAAGCAAAGAG(	natural antisense
113392649	113420493		CCTTCCGCAGGCTCAATCAAACCTT(	intergenic

NM_001261401	TSN	translin isoform 2	+	122513120	122525428
NM_004622	TSN	translin isoform 1	+	122513120	122525428
NM_021035	ZNFX1	NFX1-type zinc fing	-	47862438	47894756
NM_001146341	ANKRD34C	ankyrin repeat domai	+	79575145	79590581
NM_001094	ASIC2	acid-sensing ion char	-	31340105	32483825
NM_005993	TBCD	tubulin-specific chap	+	80709939	80901062
NM_016406	UFC1	ubiquitin-fold modifi	+	161123533	161128646
NM_001171992	C6orf223	uncharacterized prot	+	43968336	43973694
NM_153246	C6orf223	uncharacterized prot	+	43968336	43973694
NM_001008697	TFIP11	tuftelin-interacting pı	-	26887893	26908437
NM_012143	TFIP11	tuftelin-interacting pı	-	26887893	26908437
ENST00000404479	AVL9	AVL9 homolog (S. c	+	32535321	33078516
NM_178817	MRAP	melanocortin-2 recep	+	33664123	33684599
NM_206898	MRAP	melanocortin-2 recep	+	33664123	33687094
NM_001139517	PRKRA	interferon-inducible	-	179296140	179315484
NM_001139518	PRKRA	interferon-inducible	-	179296140	179315355
NM_003690	PRKRA	interferon-inducible	-	179296140	179315958
NM_148977	PANK1	pantothenate kinase 1	-	91339253	91405329
NM_006810	PDIA5	protein disulfide-isor	+	122785855	122880953
NM_006142	SFN	14-3-3 protein sigma	+	27189632	27190947
NM_004913	C16orf7	uncharacterized prot	-	89773540	89787394
NM_032359	C3orf26	uncharacterized prot	+	99536677	99897476
NM_001042459	FILIP1L	filamin A-interacting	-	99566771	99833349
NM_182909	FILIP1L	filamin A-interacting	-	99551987	99833349
NM_005358	LMO7	LIM domain only pro	+	76194569	76434006
NM_006002	UCHL3	ubiquitin carboxyl-te	+	76123926	76180068
NM_001261401	TSN	translin isoform 2	+	122513120	122525428
NM_004622	TSN	translin isoform 1	+	122513120	122525428
NM_001008737	LOC401052	uncharacterized prot	-	10048101	10052779
NM_000296	PKD1	polycystin-1 isoform	-	2138710	2185899
NM_001009944	PKD1	polycystin-1 isoform	-	2138710	2185899
NM_002853	RAD1	cell cycle checkpoint	-	34905365	34915780
NM_001160364	TMC7	transmembrane chanı	+	18995608	19075262
NM_016138	COQ7	ubiquinone biosynthe	+	19078916	19091417
NM_024847	TMC7	transmembrane chanı	+	18995255	19075262

10750.6451	2034.910117	12.885199	11.336795	9477.081	7520.9316
87.047469	17.791313	6.051807	4.242685	94.28642	116.381516
1577.29391	437.799977	10.180522	9.038193	1534.9366	1882.5709
1939.807417	71.429164	10.308621	6.236713	1677.7031	419.5658
255.162943	33.927209	7.489885	5.376685	185.98445	127.68867
1457.510383	358.957973	10.084494	8.759725	1741.8114	1639.5204
1457.510383	358.957973	10.084494	8.759725	1741.8114	1639.5204
1920.072717	310.934813	10.354292	8.658197	2395.331	3572.6296
266.69533	83.649062	7.667126	6.657932	309.90176	335.3147
57.717888	7.870297	5.048721	2.923296	111.14891	108.22752
23.441889	7.641521	4.147555	2.911686	30.066444	12.422369
71.158937	23.193359	5.757188	4.648272	85.17901	103.14032
26.039988	8.41812	4.369322	3.308727	32.747986	26.105202
26.039988	8.41812	4.369322	3.308727	32.747986	26.105202
157.60176	47.533578	6.624234	5.207378	180.3705	207.60675
40.753135	8.682593	4.902129	3.257971	24.475904	31.692387
21.510867	7.124972	4.068024	2.787302	26.756659	20.170282
49.680378	9.579941	5.012235	3.295413	33.82583	12.257938
49.680378	9.579941	5.012235	3.295413	33.82583	12.257938
568.053025	141.898035	8.744401	7.405959	577.88696	421.6126
25.754533	5.186516	4.350742	2.484413	29.332417	29.801147
25.754533	5.186516	4.350742	2.484413	29.332417	29.801147
116.316455	44.961433	6.365415	5.152582	149.23245	60.79848
20.628618	7.761166	4.000625	2.938098	20.8772	10.79214
782.89104	213.624437	9.084569	7.44768	689.9766	731.3918
427.859113	102.867011	8.28253	6.91852	452.7533	555.4388
28.503099	9.896651	4.498355	3.346439	36.62815	30.316042
28.503099	9.896651	4.498355	3.346439	36.62815	30.316042
28.503099	9.896651	4.498355	3.346439	36.62815	30.316042
36.965432	8.550593	4.784997	2.999148	29.491035	59.75153
1037.368517	322.723299	9.53828	8.522843	931.0764	1003.9726
31.146869	12.555868	4.545984	3.488452	35.78565	28.212313
112.494284	30.900586	6.415515	5.024911	141.77417	157.60875
147.707082	39.294843	6.807881	5.577728	145.02911	90.95939
117.01996	22.567778	6.427223	4.288895	167.3139	187.97725
151.444525	16.608473	6.661809	4.229032	206.4612	315.21988
79.876579	14.671084	5.605952	4.064387	55.50912	88.75417
37.09734	12.929272	4.834134	3.797632	52.42129	48.704067
154.929213	31.383718	6.786255	5.026586	222.2414	237.48242
147.755772	10.97213	6.815831	3.641231	139.80717	128.9881
354.917593	7.801135	5.985613	3.019614	33.34432	49.98985
1376.360667	269.948275	9.954222	8.307206	1060.4282	987.708
1376.360667	269.948275	9.954222	8.307206	1060.4282	987.708
1376.360667	269.948275	9.954222	8.307206	1060.4282	987.708
250.99047	81.493835	7.579522	6.427702	305.88437	315.1074
159.116192	41.528583	6.865588	5.685891	142.41237	61.297024
159.116192	41.528583	6.865588	5.685891	142.41237	61.297024
1120.976067	174.800988	9.650401	7.757028	1268.7169	1752.0636
1237.044517	327.276028	9.759644	8.647281	1527.1295	2018.1864
387.106852	97.446883	8.161594	6.949769	482.01343	375.67862
344.183117	58.648181	8.031199	6.127316	313.15692	245.66928
492.681673	135.115991	8.551975	7.376738	513.16693	429.15292
492.681673	135.115991	8.551975	7.376738	513.16693	429.15292
1807.960467	288.442212	10.274753	8.531261	1857.4738	2733.4202
182.387528	23.276871	6.890342	4.651004	227.84512	388.6142
182.387528	23.276871	6.890342	4.651004	227.84512	388.6142
3627.1691	715.33611	11.25052	9.799768	4083.691	6016.137
1227.51774	288.607962	9.853087	8.539901	1253.3474	1079.5638
23.057858	8.306912	4.184729	3.164197	19.401173	17.766785
23.057858	8.306912	4.184729	3.164197	19.401173	17.766785
23.057858	8.306912	4.184729	3.164197	19.401173	17.766785
1865.186167	230.532465	10.168462	8.190339	2731.8196	4057.815

11265.564	17442.828	6095.361	12702.105	1457.5242	1350.3302
52.209198	91.12787	67.91101	100.3688	6.4325	9.542981
935.19196	1721.614	1482.1716	1907.2784	537.34863	237.80438
2544.808	2721.0386	1663.474	2612.255	52.416042	63.805264
196.90344	183.07654	414.9571	422.36746	25.471645	26.304087
1586.521	1068.2518	1216.136	1492.8217	416.07474	235.77245
1586.521	1068.2518	1216.136	1492.8217	416.07474	235.77245
896.5119	1800.5338	1156.7584	1698.6716	317.0396	327.73093
198.1051	193.83295	266.66287	296.3546	99.48429	46.243263
22.011026	11.34776	58.169605	35.402508	8.343639	4.9999995
37.23398	15.35244	21.699602	23.8765	6.501653	4.9999995
58.7234	46.47485	62.67367	70.76237	33.966194	12.973089
25.468615	31.282568	16.46153	24.174028	7.030767	6.093693
25.468615	31.282568	16.46153	24.174028	7.030767	6.093693
122.85239	25.6617	268.42044	140.69878	68.81674	15.053138
21.631058	43.69169	61.38471	61.64306	5.5015745	5.7097197
16.57612	16.38664	18.275267	30.900234	8.114907	4.9999995
57.09554	108.82429	21.99864	64.08003	6.0481043	4.9999995
57.09554	108.82429	21.99864	64.08003	6.0481043	4.9999995
626.641	539.85864	518.93915	723.3798	137.9469	77.54272
24.188448	19.55415	27.03338	24.617659	4.9999995	4.9999995
24.188448	19.55415	27.03338	24.617659	4.9999995	4.9999995
223.51389	116.8175	59.20584	88.33057	66.04621	8.052607
31.585838	23.664288	14.716338	22.135906	4.9999995	4.9999995
470.34634	370.9002	1382.8269	1051.9044	199.3131	100.74629
269.9925	599.66144	235.71269	453.59595	77.25147	51.397175
21.153128	26.708319	25.383945	30.829008	14.52259	4.9999995
21.153128	26.708319	25.383945	30.829008	14.52259	4.9999995
21.153128	26.708319	25.383945	30.829008	14.52259	4.9999995
23.637655	24.619562	45.023197	39.269615	4.9999995	4.9999995
1152.1102	1517.3818	452.6213	1167.0488	400.03882	262.881
44.309814	14.26193	34.340954	29.97055	12.92976	4.9999995
92.541046	74.80904	111.44949	96.78321	36.845615	16.476908
219.19124	113.84359	152.25146	164.9677	26.977015	29.609888
98.15576	78.355	79.16055	91.1573	23.85625	4.9999995
61.174553	78.614	119.841995	127.35552	17.278507	12.712532
14.24332	170.59337	40.863293	109.2962	23.76211	20.550522
27.11855	37.03348	22.805813	34.50084	19.585592	10.363169
110.59901	66.15954	172.2711	120.82181	37.335335	20.705294
122.48898	203.2481	97.53829	194.46399	16.89942	12.168402
6.608051	1392.4952	23.761496	623.30664	6.1242003	4.9999995
1160.8174	2190.867	968.8668	1889.4766	191.67204	211.23085
1160.8174	2190.867	968.8668	1889.4766	191.67204	211.23085
1160.8174	2190.867	968.8668	1889.4766	191.67204	211.23085
208.26523	176.13048	224.16612	276.38922	91.671425	29.9913
171.91287	218.11234	140.7339	220.22865	42.118855	28.575373
171.91287	218.11234	140.7339	220.22865	42.118855	28.575373
711.2311	658.3378	1169.1962	1166.3108	140.94658	163.20955
761.9639	619.7929	1356.4459	1138.7485	414.59708	241.29413
578.5983	237.64848	312.33038	336.3719	95.60457	80.29828
322.97092	434.70248	300.9268	447.6723	57.933968	70.146164
591.6105	466.69922	440.70587	514.7546	140.99448	119.89298
591.6105	466.69922	440.70587	514.7546	140.99448	119.89298
741.1779	1048.3759	2171.5464	2295.7686	275.38562	226.99312
47.941235	159.92793	119.58848	150.4082	17.03396	17.988161
47.941235	159.92793	119.58848	150.4082	17.03396	17.988161
1658.2612	4482.7993	1885.4965	3636.6296	547.69434	433.18304
1231.8217	1424.358	927.41174	1448.6038	332.763	283.11935
18.215904	23.888443	28.951077	30.123768	8.618533	5.881529
18.215904	23.888443	28.951077	30.123768	8.618533	5.881529
18.215904	23.888443	28.951077	30.123768	8.618533	5.881529
681.1596	770.904	1447.0073	1502.4115	256.00644	266.25687

1522.8372	2342.7432	3220.3074	2315.7185	12.716614	12.418426
12.25663	34.56606	23.966578	19.98313	6.111949	6.638878
805.96985	323.46915	398.98193	323.22592	10.118137	10.465122
45.127888	169.75772	20.540112	76.92796	10.241213	8.405157
42.798546	33.69071	44.45701	30.841259	7.0846567	6.7675476
600.33966	218.44637	427.76312	255.3515	10.285152	10.2847595
600.33966	218.44637	427.76312	255.3515	10.285152	10.2847595
342.25192	337.15747	273.73856	267.6904	10.74749	11.380152
132.20389	82.56025	69.729805	71.672874	7.8194895	8.106307
17.613869	4.9999995	6.2642775	4.9999995	6.348556	6.5395555
15.47081	4.9999995	8.876665	4.9999995	4.4552975	3.6861494
42.969044	12.240588	22.73294	14.278301	5.959047	6.4735727
8.783686	13.697829	5.7921677	9.11058	4.5726643	4.6559877
8.783686	13.697829	5.7921677	9.11058	4.5726643	4.6559877
130.8164	10.064541	48.42013	12.03052	7.0376287	7.444062
4.9999995	11.342192	15.684301	8.857769	4.1560416	4.913018
4.9999995	14.634929	4.9999995	4.9999995	4.285285	4.308419
12.721691	6.1584544	16.746729	10.804669	4.617239	3.6673532
12.721691	6.1584544	16.746729	10.804669	4.617239	3.6673532
207.69302	100.54517	207.4302	120.2302	8.697764	8.410749
5.8445764	4.9999995	4.9999995	5.274524	4.4188957	4.8323226
5.8445764	4.9999995	4.9999995	5.274524	4.4188957	4.8323226
133.46768	21.312073	23.81356	17.07647	6.766109	5.764691
9.772247	14.777357	4.9999995	7.017396	3.9254444	3.4983444
751.549	71.84063	84.25216	74.04544	8.959921	9.190696
97.70233	91.91839	189.12737	109.80533	8.346241	8.808494
16.652136	9.856381	6.497839	6.850963	4.730001	4.8565645
16.652136	9.856381	6.497839	6.850963	4.730001	4.8565645
16.652136	9.856381	6.497839	6.850963	4.730001	4.8565645
21.982664	6.9751105	6.3531804	5.992606	4.426147	5.737925
522.4824	172.32628	118.697815	459.91348	9.394972	9.625287
30.682272	6.3048453	15.114631	5.3037004	4.6981654	4.761913
69.858315	23.707752	24.609165	13.905761	6.696677	7.0612116
28.747158	49.95041	56.442757	44.041832	6.72624	6.300601
46.928314	9.409764	35.72559	14.48675	6.9289193	7.3125806
32.53402	14.14483	13.248291	9.732659	7.236231	8.024297
11.52274	15.600319	8.863767	7.727048	5.3299847	6.266438
24.460474	9.100443	7.5111957	6.554757	5.2444377	5.4777617
73.93515	14.337931	16.349808	25.638792	7.3460164	7.629021
9.629401	15.612959	5.5820165	5.940584	6.674798	6.780623
7.3587017	12.923561	4.9999995	10.40035	4.5973473	5.5092583
156.90063	577.1025	191.5571	291.22653	9.580333	9.603598
156.90063	577.1025	191.5571	291.22653	9.580333	9.603598
156.90063	577.1025	191.5571	291.22653	9.580333	9.603598
194.01933	55.02098	64.601105	53.658867	7.8046746	8.023499
42.405163	56.771877	39.15552	40.14471	6.703782	5.775085
42.405163	56.771877	39.15552	40.14471	6.703782	5.775085
110.04651	169.53333	270.52972	194.54024	9.836138	10.369123
553.28516	234.98592	284.84735	234.64653	10.1121025	10.564302
104.41108	104.09899	108.34932	91.91906	8.433533	8.260652
46.606197	93.84922	26.48987	56.86367	7.835102	7.674845
154.48799	88.04018	195.80492	111.475395	8.518262	8.435698
154.48799	88.04018	195.80492	111.475395	8.518262	8.435698
281.6259	318.605	383.04	245.00363	10.379971	11.004203
19.17898	8.704041	48.806282	27.949802	7.3804493	8.308653
19.17898	8.704041	48.806282	27.949802	7.3804493	8.308653
615.97394	669.6207	1214.29	811.25464	11.507314	12.093601
340.74915	255.93909	294.04303	225.03415	9.814762	9.728731
13.35673	4.9999995	11.984681	4.9999995	3.8188398	4.1497364
13.35673	4.9999995	11.984681	4.9999995	3.8188398	4.1497364
13.35673	4.9999995	11.984681	4.9999995	3.8188398	4.1497364
199.4907	170.1862	294.6131	196.64148	10.926817	11.56925

13.13705	13.785112	12.256933	12.997057	10.806877	11.278831
5.3876977	6.1569686	5.885818	6.129531	2.8828216	4.032107
9.500546	10.435458	10.255947	10.3079195	9.343954	8.742029
10.961197	11.078003	10.414447	10.751712	5.947156	6.8535905
7.3070207	7.193693	8.435932	8.150463	4.9158983	5.5526185
10.266412	9.74134	9.978375	9.950925	8.959466	8.73072
10.266412	9.74134	9.978375	9.950925	8.959466	8.73072
9.444992	10.500728	9.914339	10.138048	8.579748	9.215206
7.316505	7.280607	7.8162346	7.6636105	6.87187	6.3787045
4.14684	2.8993402	5.6731024	4.6849294	3.2661953	2.4279919
4.9037623	3.377954	4.33538	4.1267853	2.8961146	2.4279919
5.560849	5.1369796	5.7778983	5.6347795	5.3167114	4.4951386
4.3590403	4.5226064	3.962995	4.1426377	3.008656	3.356644
4.3590403	4.5226064	3.962995	4.1426377	3.008656	3.356644
6.619415	4.209964	7.8271074	6.6072288	6.337456	4.726418
4.1168876	5.0393906	5.7483354	5.4391	2.6623921	3.252258
3.7340884	3.4836776	4.0999346	4.4967422	3.2238717	2.4279919
5.521252	6.4182673	4.356733	5.492564	2.7994356	2.4279919
5.521252	6.4182673	4.356733	5.492564	2.7994356	2.4279919
8.922047	8.753903	8.762941	8.919001	7.336216	7.1282496
4.2874265	3.76308	4.6332603	4.169465	2.3467326	2.4279919
4.2874265	3.76308	4.6332603	4.169465	2.3467326	2.4279919
7.4826546	6.5351486	5.698315	5.94557	6.2824507	3.7756088
4.6721487	4.0761456	3.8092117	4.0224533	2.3467326	2.4279919
8.5281725	8.215106	10.1589775	9.454541	7.883546	7.506278
7.7427273	8.909478	7.6363735	8.251866	6.4941735	6.5322685
4.0829077	4.275234	4.5520873	4.493335	4.0906777	2.4279919
4.0829077	4.275234	4.5520873	4.493335	4.0906777	2.4279919
4.0829077	4.275234	4.5520873	4.493335	4.0906777	2.4279919
4.2544146	4.1396093	5.329326	4.8225617	2.3467326	2.4279919
9.797711	10.248859	8.563796	9.599056	8.903383	8.899571
5.149675	3.2614124	4.9509387	4.453799	3.918315	2.4279919
6.2093406	5.8658195	6.5832157	6.0768285	5.4395046	4.857379
7.4539933	6.4931407	7.030743	6.842569	4.991452	5.729191
6.296392	5.9305	6.103254	5.991692	4.817898	2.4279919
5.625095	5.9359426	6.6829767	6.4663134	4.3484817	4.4655905
3.506844	7.0858855	5.196118	6.2504406	4.812683	5.1927495
4.4519744	4.7787347	4.4027066	4.6491885	4.527752	4.158482
6.4703364	5.677317	7.199653	6.395189	5.4589586	5.204162
6.6151643	7.350687	6.393365	7.080346	4.3138666	4.398897
2.5193186	10.1200905	4.460094	8.707568	2.8166542	2.4279919
9.809677	10.766919	9.66763	10.297173	7.826436	8.583392
9.809677	10.766919	9.66763	10.297173	7.826436	8.583392
9.809677	10.766919	9.66763	10.297173	7.826436	8.583392
7.387667	7.137956	7.560299	7.563038	6.746698	5.7451324
7.097097	7.459026	6.9165754	7.2419643	5.6280966	5.6740084
7.097097	7.459026	6.9165754	7.2419643	5.6280966	5.6740084
9.120057	9.051258	9.927347	9.598484	7.367963	8.199669
9.223883	8.958134	10.133249	9.566196	8.953343	8.765599
8.814412	7.5857844	8.03762	7.8375635	6.813386	7.1781096
8.000007	8.455187	7.984756	8.237296	6.093305	6.9797316
8.845293	8.552278	8.525616	8.4347	7.3688364	7.7516103
8.845293	8.552278	8.525616	8.4347	7.3688364	7.7516103
9.184741	9.717477	10.79365	10.568475	8.368076	8.681705
5.2653704	7.002425	6.679782	6.70537	4.327213	4.9900074
5.2653704	7.002425	6.679782	6.70537	4.327213	4.9900074
10.327265	11.78335	10.575918	11.215674	9.372992	9.628566
9.899648	10.156183	9.609886	9.909314	8.645444	9.009822
3.8681867	4.0905347	4.718844	4.4622335	3.3173547	3.301185
3.8681867	4.0905347	4.718844	4.4622335	3.3173547	3.301185
3.8681867	4.0905347	4.718844	4.4622335	3.3173547	3.301185
9.049915	9.27991	10.221312	9.963567	8.257734	8.921684

10.511782	11.691804	11.934742	11.796733
3.7015316	5.3344903	4.852885	4.652274
9.601361	8.78512	8.882076	8.87462
5.493387	7.7804074	4.6258116	6.719927
5.4151297	5.294198	5.7426953	5.339569
9.176752	8.179365	8.978088	8.533957
9.176752	8.179365	8.978088	8.533957
8.363706	8.841461	8.348345	8.600718
7.0026126	6.694112	6.3870625	6.6132326
4.1911573	2.3408759	2.9608014	2.352752
4.0151806	2.3408759	3.437199	2.352752
5.420487	3.7291548	4.7790666	4.1490726
3.2557085	3.8993936	2.8642077	3.4677505
3.2557085	3.8993936	2.8642077	3.4677505
6.987682	3.432223	5.870579	3.8899095
2.350709	3.6137557	4.242465	3.426247
2.350709	4.006671	2.3618152	2.352752
3.750249	2.727484	4.3402977	3.7270198
3.750249	2.727484	4.3402977	3.7270198
7.6501603	6.9918776	7.948268	7.3809814
2.7586527	2.3408759	2.3618152	2.6704106
2.7586527	2.3408759	2.3618152	2.6704106
7.015192	4.58052	4.844448	4.4172745
3.3972692	4.0210915	2.3618152	3.073688
9.498456	6.4859223	6.651796	6.6600823
6.5707603	6.8550367	7.8107657	7.2481155
4.1159286	3.4010706	3.0094736	3.0334897
4.1159286	3.4010706	3.0094736	3.0334897
4.1159286	3.4010706	3.0094736	3.0334897
4.4921613	2.899209	2.9821725	2.8466237
8.984114	7.8060694	7.141159	9.40276
4.9546123	2.7595077	4.1928	2.677483
6.100109	4.7498713	4.8921423	4.110457
4.868076	5.9137545	6.0849648	5.8789306
5.551254	3.3318276	5.433426	4.1709704
5.0386252	3.9503138	4.0018587	3.5693223
3.6207082	4.105268	3.4353662	3.2195497
4.636594	3.2791445	3.2108774	2.9729407
6.180808	3.9726171	4.3036833	5.0392885
3.3799818	4.1070237	2.8148434	2.8327746
3.029783	3.8113515	2.3618152	3.6700885
7.2449427	9.635363	7.8309865	8.722115
7.2449427	9.635363	7.8309865	8.722115
7.2449427	9.635363	7.8309865	8.722115
7.5514555	6.0635896	6.280188	6.1791477
5.400729	6.1143775	5.560849	5.7372837
5.400729	6.1143775	5.560849	5.7372837
6.7457557	7.7781305	8.330459	8.120192
9.061621	8.292739	8.402716	8.407669
6.6695514	7.0485106	7.0156317	6.9734254
5.5419273	6.883382	4.995922	6.2696285
7.220987	6.7906613	7.859729	7.2686057
7.220987	6.7906613	7.859729	7.2686057
8.084107	8.763752	8.819457	8.47047
4.309255	3.2195497	5.880985	5.1790113
4.309255	3.2195497	5.880985	5.1790113
9.208775	9.853944	10.507352	10.226981
8.357478	8.4298525	8.449027	8.347784
3.813519	2.3408759	3.8594937	2.352752
3.813519	2.3408759	3.8594937	2.352752
3.813519	2.3408759	3.8594937	2.352752
7.5896845	7.783832	8.452133	8.136969

ASHGA5P051168	0.006104865	0.049856523	4.0762319	up	noncoding
ASHGA5P027784	0.002794163	0.034179323	2.1111857	up	noncoding
ASHGA5P026242	0.001915398	0.02921028	2.6577364	up	noncoding
ASHGA5P019130	0.002766248	0.034110227	2.5123226	up	noncoding
ASHGA5P019130	0.002766248	0.034110227	2.5123226	up	noncoding
ASHGA5P019130	0.002766248	0.034110227	2.5123226	up	noncoding
ASHGA5P019130	0.002766248	0.034110227	2.5123226	up	noncoding
ASHGA5P019130	0.002766248	0.034110227	2.5123226	up	noncoding
ASHGA5P023676	0.014320729	0.073312647	2.2296534	up	noncoding
ASHGA5P021984	0.014721089	0.0741837	2.8666456	up	noncoding
ASHGA5P021984	0.014721089	0.0741837	2.8666456	up	noncoding
ASHGA5P021984	0.014721089	0.0741837	2.8666456	up	noncoding
ASHGA5P016221	0.000381481	0.013977512	2.6180707	up	noncoding
ASHGA5P016221	0.000381481	0.013977512	2.6180707	up	noncoding
ASHGA5P028688	0.000783512	0.018680184	3.219742	up	noncoding
ASHGA5P046043	0.001647175	0.027211232	2.6800831	up	noncoding
ASHGA5P046246	0.043972835	0.134639736	2.0977676	up	noncoding
ASHGA5P035933	0.028885242	0.106084163	2.0732178	up	noncoding
ASHGA5P033299	0.015925146	0.077147596	3.2959495	up	noncoding
ASHGA5P033015	0.007945462	0.056185826	2.0135183	up	noncoding
ASHGA5P047378	0.001219438	0.023487681	2.2256098	up	noncoding
ASHGA5P047378	0.001219438	0.023487681	2.2256098	up	noncoding
ASHGA5P049732	0.001040226	0.021859875	4.4783382	up	noncoding
ASHGA5P017559	7.06167E-05	0.008148697	2.6906579	up	noncoding
ASHGA5P054035	6.04143E-05	0.007803818	2.0235081	up	noncoding
ASHGA5P013446	0.001644141	0.027211232	2.5845256	up	noncoding
ASHGA5P013446	0.001644141	0.027211232	2.5845256	up	noncoding
ASHGA5P013446	0.001644141	0.027211232	2.5845256	up	noncoding
ASHGA5P013446	0.001644141	0.027211232	2.5845256	up	noncoding
ASHGA5P013446	0.001644141	0.027211232	2.5845256	up	noncoding
ASHGA5P013446	0.001644141	0.027211232	2.5845256	up	noncoding
ASHGA5P051627	0.004243582	0.041329133	2.2736954	up	noncoding
ASHGA5P051627	0.004243582	0.041329133	2.2736954	up	noncoding
ASHGA5P057368	0.006373145	0.050835376	2.3153823	up	noncoding
ASHGA5P017167	0.001366125	0.024734332	2.2685308	up	noncoding
ASHGA5P028561	0.000265166	0.012239337	3.4052835	up	noncoding
ASHGA5P034923	0.002328801	0.031698104	2.8003409	up	noncoding
ASHGA5P050607	0.003092283	0.035994844	2.326086	up	noncoding
ASHGA5P044545	0.018093394	0.081727179	2.1407286	up	noncoding
ASHGA5P029033	0.001845774	0.028874356	2.3747957	up	noncoding
ASHGA5P035008	0.015165646	0.075388329	18.6183457	up	noncoding
ASHGA5P040074	0.004201296	0.041263975	4.3477601	up	noncoding
ASHGA5P049521	0.000195915	0.01146874	3.6506345	up	noncoding
ASHGA5P022339	0.022582563	0.092978491	2.6094926	up	noncoding
ASHGA5P028010	0.004691292	0.04352049	3.8355519	up	noncoding
ASHGA5P053209	0.03602431	0.120468465	2.9347867	up	noncoding
ASHGA5P007948	0.026853323	0.102230927	2.614317	up	noncoding
ASHGA5P007948	0.026853323	0.102230927	2.614317	up	noncoding
ASHGA5P026990	0.026164773	0.100745216	2.0821929	up	noncoding
ASHGA5P036781	0.000626005	0.0169305	2.3618568	up	noncoding
ASHGA5P043456	0.008496024	0.057580891	2.0482363	up	noncoding
ASHGA5P033432	5.0087E-05	0.007392592	3.0655174	up	noncoding
ASHGA5P033432	5.0087E-05	0.007392592	3.0655174	up	noncoding
ASHGA5P023928	0.043208986	0.133288439	2.0824095	up	noncoding
ASHGA5P026006	0.004126711	0.041046245	2.1510514	up	noncoding
ASHGA5P026006	0.004126711	0.041046245	2.1510514	up	noncoding
ASHGA5P026006	0.004126711	0.041046245	2.1510514	up	noncoding
ASHGA5P026006	0.004126711	0.041046245	2.1510514	up	noncoding
ASHGA5P031786	0.001087985	0.022286303	9.8893522	up	noncoding
ASHGA5P053490	0.001778983	0.028224023	2.5048662	up	noncoding
ASHGA5P037208	0.00088743	0.020090144	3.3076784	up	noncoding
ASHGA5P055834	0.000656504	0.017168766	8.8708804	up	noncoding

ENST00000444998	MCM3AP-AS1	GENCODE	2193	chr21	+
N54862		LincRNAs identified	415	chrX	+
ENST00000528646	RP11-583F24.8	GENCODE	707	chr11	+
NR_037667	INTS7	RefSeq	4589	chr1	-
NR_037667	INTS7	RefSeq	4589	chr1	-
NR_037667	INTS7	RefSeq	4589	chr1	-
NR_037667	INTS7	RefSeq	4589	chr1	-
NR_037667	INTS7	RefSeq	4589	chr1	-
HMlincRNA1441+	HMlincRNA1441	LincRNAs identified	7848	chr8	+
ENST00000546421	RP11-181C3.2	GENCODE	738	chr12	-
ENST00000546421	RP11-181C3.2	GENCODE	738	chr12	-
ENST00000546421	RP11-181C3.2	GENCODE	738	chr12	-
NR_045512	WBSCR22	RefSeq	1159	chr7	+
NR_045512	WBSCR22	RefSeq	1159	chr7	+
ENST00000412194	BX248398.1	GENCODE	373	chr1	-
ENST00000423793	RP11-432J22.2	GENCODE	311	chr9	+
TCONS_00001509	XLOC_000852	LincRNAs identified	503	chr1	-
ENST00000441587	ALMS1-IT1	GENCODE	1766	chr2	+
uc002jcf.3	TCAM1P	UCSC_knowngene	3291	chr17	+
ENST00000582557	SUZ12P1	GENCODE	1560	chr17	+
ENST00000418966	ZNF32-AS2	GENCODE	905	chr10	+
ENST00000418966	ZNF32-AS2	GENCODE	905	chr10	+
ENST00000492522	CTD-2377D24.6	GENCODE	695	chr17	+
uc003tui.3	CCT6P1	UCSC_knowngene	1528	chr7	+
ENST00000453317	JPX	GENCODE	483	chrX	+
NR_029416	SHMT2	RefSeq	2176	chr12	+
NR_029416	SHMT2	RefSeq	2176	chr12	+
NR_029416	SHMT2	RefSeq	2176	chr12	+
NR_029416	SHMT2	RefSeq	2176	chr12	+
NR_029416	SHMT2	RefSeq	2176	chr12	+
NR_029416	SHMT2	RefSeq	2176	chr12	+
ENST00000469070	RP11-18H7.1	GENCODE	982	chr3	+
ENST00000469070	RP11-18H7.1	GENCODE	982	chr3	+
TCONS_00016364	XLOC_007744	LincRNAs identified	414	chr9	-
NR_038429	LOC100506054	RefSeq	1329	chr2	+
ENST00000449579	DLEU2	GENCODE	791	chr13	-
ENST00000423706	AC012506.1	GENCODE	525	chr2	-
ENST00000440975	AC012487.2	GENCODE	574	chr2	-
ENST00000429151	AF230666.2	GENCODE	773	chr8	-
NR_023922	DHRS4-AS1	RefSeq	2770	chr14	-
ENST00000444871	RP11-89K21.1	GENCODE	581	chr2	-
ENST00000420701	AC113617.1	GENCODE	597	chr4	+
NR_045026	C17orf76-AS1	RefSeq	552	chr17	+
ENST00000554841	RP1-261D10.2	GENCODE	525	chr14	-
NR_027358	LINC00592	RefSeq	1075	chr12	+
ENST00000425474	AZGP1P1	GENCODE	1181	chr7	+
NR_026675	CRYM-AS1	RefSeq	2304	chr16	+
NR_026675	CRYM-AS1	RefSeq	2304	chr16	+
ENST00000543308	RP11-1038A11.2	GENCODE	1727	chr12	-
ENST00000424235	RP4-564F22.2	GENCODE	764	chr20	-
uc003sio.1	AK024243	UCSC_knowngene	4411	chr7	+
ENST00000583521	RP11-498C9.13	GENCODE	450	chr17	+
ENST00000583521	RP11-498C9.13	GENCODE	450	chr17	+
HMlincRNA971+	HMlincRNA971	LincRNAs identified	12189	chr3	+
NR_052023	EIF6	RefSeq	1128	chr20	-
NR_052023	EIF6	RefSeq	1128	chr20	-
NR_052023	EIF6	RefSeq	1128	chr20	-
NR_052023	EIF6	RefSeq	1128	chr20	-
ENST00000560487	CTD-3032H12.2	GENCODE	359	chr16	+
ENST00000523859	RP11-37B2.1	GENCODE	696	chr8	-
TCONS_00025773	XLOC_012582	LincRNAs identified	857	chr17	-
uc001vnl.3	AF339817	UCSC_knowngene	6082	chr13	+

47649153	47662913	ENST00000421927,	GCAGAAGGATGA 114044	natural antisense
64070590	64070929		TTTCAGTAGAACTGGTAAACAGTTC	intergenic
18956531	18961268		TGAGATGATTCTTGGCCTCTGTCCC	natural antisense
212113740	212209002		GTCTGTGAAGTA 25896	exon sense-overlappi
212113740	212209002		GTCTGTGAAGTA 25896	exon sense-overlappi
212113740	212209002		GTCTGTGAAGTA 25896	exon sense-overlappi
212113740	212209002		GTCTGTGAAGTA 25896	exon sense-overlappi
212113740	212209002		GTCTGTGAAGTA 25896	natural antisense
30192504	30200352		TCATCCATACATCCAAACTTCAGAG	intergenic
98909071	98909988		GCGAGAGGAGACGCCGATAAGGGA	natural antisense
98909071	98909988		GCGAGAGGAGACGCCGATAAGGGA	natural antisense
98909071	98909988		GCGAGAGGAGACGCCGATAAGGGA	natural antisense
73097897	73112551		GTTGCATCAGGT 114049	exon sense-overlappi
73097897	73112551		GTTGCATCAGGT 114049	exon sense-overlappi
144325838	144336254	ENST00000444883	CCCGTTCTACTTTTTCAGCTTGTTGC	intergenic
138799657	138805359		AGATTGTCTATTTTTTCTTGAGTCAC	bidirectional
59761065	59761673		GTTTAGCAGTCGCTGAGTCCAAACA	intergenic
73684229	73686609		CAAACCAAACAAAAACCATGAACTC	intron sense-overlapp
61934375	61941739	ENST00000478379	CTCCCAAATCAG 146771	intergenic
29061080	29097068		GCGTGATTTCCATAGCAGATGACAA	intergenic
44141389	44143467		CACAGGTCTGAG 414208	natural antisense
44141389	44143467		CACAGGTCTGAG 414208	natural antisense
46782622	46783692		GGCAGATCGGGTATATCTTTAAAGA	intergenic
65216091	65228662		CCATAAAGATGG 643253	intergenic
73164180	73164781		GTTGCAGAAGGTAGCTCCTAGGAGC	intergenic
57623827	57628718	NR_029415, NR_02	TGCCTCAGAGAT 6472	exon sense-overlappi
57623827	57628718	NR_029415, NR_02	TGCCTCAGAGAT 6472	exon sense-overlappi
57623827	57628718	NR_029415, NR_02	TGCCTCAGAGAT 6472	exon sense-overlappi
57623827	57628718	NR_029415, NR_02	TGCCTCAGAGAT 6472	exon sense-overlappi
57623827	57628718	NR_029415, NR_02	TGCCTCAGAGAT 6472	exon sense-overlappi
57623827	57628718	NR_029415, NR_02	TGCCTCAGAGAT 6472	natural antisense
119814116	119818033		AAGAGGGGAGGTAAATTACTTTCCA	bidirectional
119814116	119818033		AAGAGGGGAGGTAAATTACTTTCCA	bidirectional
81484524	81496832		CAGACTCAAGGTCTCTCCTGAGCAC	intergenic
3605975	3609340		AGTTGAGAAGGC 100506054	natural antisense
50623325	50656114	uc001veb.2	CTGTGCTGGGCACAGAATGGACTTC	bidirectional
23553534	23554915		ATCCTGGATCACTCTGAGAGGTCAC	intergenic
109128144	109150638		GGAAATTGTGGTGTACTTGTGTAAG	bidirectional
133850374	133856543		CCAGGAAAAAGTCACACCTCTTCCC	natural antisense
24407939	24424298		TTTATTATGACCT 55449	natural antisense
45158260	45166027		GGCAAACACTATGTCCTTTTCTTTTT	intergenic
118281780	118293754	ENST00000448804	GGCTGTTCCATATATATATATATGG	intergenic
16342300	16344525	ENST00000460249,	ATGATCCCTTCA 125144	intergenic
71759434	71762079		AGCCTGGATTCTTTAACAGTGATGT	intergenic
52604713	52617597		CCCCTTATACAA 283404	bidirectional
99578384	99581860		ATGGAGACCCTG 646282	intergenic
21312169	21329912		GGGTACCTAGAG 400508	intronic antisense
21312169	21329912		GGGTACCTAGAG 400508	intronic antisense
5475213	5476940		ATTTAAGATGTAGCAACTGGTATAA	intergenic
37059422	37063977		CTGAGCTTGATTGGGGCTCTGAGAG	intergenic
149596	154868		GGGCTCCTAGTCGTTTTATTTTTAGC	intergenic
79890273	79890934		TGAGGTTGGGGAATCCACCCCTGTT	natural antisense
79890273	79890934		TGAGGTTGGGGAATCCACCCCTGTT	natural antisense
182007053	182019242		AACTTAGATCTTTTCACATCCTTTCA	intergenic
33866708	33872619		ATGTGTGTGGGC 3692	exon sense-overlappi
33866708	33872619		ATGTGTGTGGGC 3692	exon sense-overlappi
33866708	33872619		ATGTGTGTGGGC 3692	exon sense-overlappi
33866708	33872619		ATGTGTGTGGGC 3692	exon sense-overlappi
54968824	54988577		CCAACTCCAATGCGTAGTGCTCATT	intergenic
90768462	90769592	ENST00000504145,	GAGAGGTGGATGATAAACCCTGTCA	bidirectional
76613497	76614757	TCONS_00025772	GGATTGCAAGACAGTAGTTCCCTGT	intergenic
99053669	99059751		GTAAGAACAAGGTAGCACATTCATT	intron sense-overlapp

NM_003906	MCM3AP	80 kDa MCM3-assoc -	47655047	47705236
NM_147199	MRGPRX1	mas-related G-protei -	18955359	18956549
NM_001199809	INTS7	integrator complex su -	212113740	212209002
NM_001199811	INTS7	integrator complex su -	212113740	212209002
NM_001199812	INTS7	integrator complex su -	212113740	212209002
NM_015434	INTS7	integrator complex su -	212113740	212209002
NM_016448	DTL	denticleless protein h +	212208918	212278187
NM_001032283	TMPO	thymopoietin isoform +	98909350	98944157
NM_001032284	TMPO	thymopoietin isoform +	98909350	98944157
NM_003276	TMPO	thymopoietin isoform +	98909350	98929412
NM_001202560	WBSCR22	uncharacterized meth +	73097897	73112551
NM_017528	WBSCR22	uncharacterized meth +	73097897	73112551
NM_015447	CAMSAP1	calmodulin-regulated -	138700332	138799005
NM_015120	ALMS1	Alstrom syndrome pr +	73612885	73837046
NM_001005368	ZNF32	zinc finger protein 32 -	44139306	44144152
NM_006973	ZNF32	zinc finger protein 32 -	44139306	44144326
NM_001166356	SHMT2	serine hydroxymethy +	57623355	57628718
NM_001166357	SHMT2	serine hydroxymethy +	57624142	57628718
NM_001166358	SHMT2	serine hydroxymethy +	57623827	57628718
NM_001166359	SHMT2	serine hydroxymethy +	57624110	57628718
NM_005412	SHMT2	serine hydroxymethy +	57623355	57628718
NM_020142	NDUFA4L2	NADH dehydrogenas -	57628685	57634475
NM_001146156	GSK3B	glycogen synthase ki -	119540801	119813264
NM_002093	GSK3B	glycogen synthase ki -	119540801	119813264
ENST00000315212	RNASEH1	ribonuclease H1 [Sou -	3592704	3606206
ENST00000378180	DLEU1	deleted in lymphocyt +	50656306	50679433
NM_001193483	LIMS1	LIM and senescent ce +	109150810	109303702
NM_016018	PHF20L1	PHD finger protein 2 +	133787603	133861052
NM_021004	DHRS4	dehydrogenase/reduc +	24422964	24438488
NM_001242696	LOC283403	uncharacterized prote -	52599364	52604639
NM_001014444	CRYM	thiomorpholine-carbo -	21269838	21314404
NM_001888	CRYM	thiomorpholine-carbo -	21269838	21314404
NM_006907	PYCR1	pyrroline-5-carboxyla -	79890268	79894968
NM_153824	PYCR1	pyrroline-5-carboxyla -	79890266	79894968
NM_001267810	EIF6	eukaryotic translation -	33866708	33872520
NM_002212	EIF6	eukaryotic translation -	33866708	33872619
NM_181466	EIF6	eukaryotic translation -	33866708	33872619
NM_181468	EIF6	eukaryotic translation -	33866708	33872520
NM_003821	RIPK2	receptor-interacting s +	90769974	90803292
NM_005766	FARP1	FERM, RhoGEF and +	98795433	99102023

40.037812	6.831844	4.875043	2.847806	50.272453	60.374695
17.135352	5.826154	3.787476	2.709423	19.421278	17.735922
1772.217933	413.472578	10.349817	8.939619	2132.4219	2327.3372
68.158755	18.642617	5.730823	4.401801	65.4348	75.46517
68.158755	18.642617	5.730823	4.401801	65.4348	75.46517
68.158755	18.642617	5.730823	4.401801	65.4348	75.46517
68.158755	18.642617	5.730823	4.401801	65.4348	75.46517
68.158755	18.642617	5.730823	4.401801	65.4348	75.46517
236.555358	72.922087	7.465223	6.308404	318.53238	225.38623
34.718673	8.693894	4.729795	3.210432	41.13226	48.681538
34.718673	8.693894	4.729795	3.210432	41.13226	48.681538
34.718673	8.693894	4.729795	3.210432	41.13226	48.681538
670.958088	153.584382	8.998233	7.609729	687.6735	694.9137
670.958088	153.584382	8.998233	7.609729	687.6735	694.9137
347.2092	62.976766	7.860359	6.173414	501.61877	189.65292
105.676834	24.641857	6.331279	4.909001	101.57361	117.97892
45.156618	15.515491	4.976911	3.908056	62.985184	26.060883
118.075068	37.243241	6.39837	5.346498	171.3472	106.83917
112.401943	17.65939	6.017158	4.296464	67.890594	20.416769
1016.649677	349.748819	9.535393	8.525675	1075.6887	535.75146
200.250137	56.473994	7.219275	6.065074	270.4142	261.10397
200.250137	56.473994	7.219275	6.065074	270.4142	261.10397
54.691326	10.253475	5.388484	3.225521	56.643303	45.873966
650.278798	148.67072	8.953466	7.525507	643.547	582.5828
130.941685	40.135175	6.657748	5.640889	141.30655	157.15843
307.607698	67.404581	7.771018	6.401119	334.92435	486.07544
307.607698	67.404581	7.771018	6.401119	334.92435	486.07544
307.607698	67.404581	7.771018	6.401119	334.92435	486.07544
307.607698	67.404581	7.771018	6.401119	334.92435	486.07544
307.607698	67.404581	7.771018	6.401119	334.92435	486.07544
307.607698	67.404581	7.771018	6.401119	334.92435	486.07544
2650.854283	667.652432	10.862157	9.677118	2059.2402	2272.2295
2650.854283	667.652432	10.862157	9.677118	2059.2402	2272.2295
41.705489	12.697154	5.027897	3.816646	55.127686	50.96505
96.906686	27.137963	6.209877	5.028119	112.02562	135.24818
170.80498	31.765003	7.057467	5.289692	180.13559	176.08327
34.334838	9.132209	4.784097	3.298494	33.29673	34.109123
29.022404	8.65133	4.529321	3.311416	28.798388	23.88744
308.44192	84.478032	7.775983	6.677881	216.58078	625.24164
52.19061	14.464938	5.346019	4.098216	60.947124	68.019806
1215.345877	22.299157	8.907627	4.688974	1681.6914	182.93506
76.527576	8.347788	5.296491	3.176219	26.096722	10.908359
3661.29385	522.971403	11.257388	9.38924	3994.498	6142.8564
250.49974	60.941516	7.521707	6.137938	339.528	330.8167
50.219098	8.265752	5.063076	3.123642	40.549023	14.131311
274.958873	45.278357	7.025504	5.472248	461.56604	742.6537
86.640568	22.520849	6.029166	4.642732	88.305466	126.22627
86.640568	22.520849	6.029166	4.642732	88.305466	126.22627
31.711949	9.711804	4.577057	3.518953	30.815144	32.34446
110.24791	28.915263	6.399284	5.159363	112.85112	117.76573
43.362756	14.569394	5.063392	4.02901	49.90696	39.355293
34.867152	7.511489	4.787151	3.171021	40.2603	33.625107
34.867152	7.511489	4.787151	3.171021	40.2603	33.625107
44.088035	14.890173	4.9889	3.930646	77.88084	42.26025
1188.701717	341.115922	9.799786	8.694745	1399.874	1351.6241
1188.701717	341.115922	9.799786	8.694745	1399.874	1351.6241
1188.701717	341.115922	9.799786	8.694745	1399.874	1351.6241
1188.701717	341.115922	9.799786	8.694745	1399.874	1351.6241
268.31491	9.491934	6.390406	3.08453	61.008656	16.17564
323.833545	72.544101	7.803659	6.478926	223.73245	168.5467
514.911167	95.712944	8.562426	6.836607	694.18097	692.7842
80.941143	5.650376	5.73081	2.581733	129.7209	39.128223

16.8551	32.251022	33.56408	46.90952	5.9317	4.9999995
14.640571	20.277452	14.553369	16.183523	4.9999995	5.6978
1625.586	1823.2882	1135.1058	1589.5685	510.08627	283.74625
54.569244	57.39496	75.07382	81.014534	23.057703	10.371439
54.569244	57.39496	75.07382	81.014534	23.057703	10.371439
54.569244	57.39496	75.07382	81.014534	23.057703	10.371439
54.569244	57.39496	75.07382	81.014534	23.057703	10.371439
54.569244	57.39496	75.07382	81.014534	23.057703	10.371439
232.33975	307.42783	118.67549	216.97047	70.04434	47.07543
23.05387	40.734394	19.241108	35.46887	11.201732	4.9999995
23.05387	40.734394	19.241108	35.46887	11.201732	4.9999995
23.05387	40.734394	19.241108	35.46887	11.201732	4.9999995
668.75635	723.6797	526.21893	724.50635	157.97737	154.35742
668.75635	723.6797	526.21893	724.50635	157.97737	154.35742
699.2438	145.22778	278.44733	269.0646	75.437515	45.582508
60.131676	100.34588	110.6484	143.38252	25.886337	33.522892
83.172905	55.09082	15.284919	28.344997	18.012571	6.536799
187.83969	114.88747	43.43944	84.09744	47.681206	26.72182
119.116486	264.35788	35.185577	167.44435	13.667409	12.193449
1258.6003	1308.7903	743.5634	1177.5039	351.22183	103.374374
180.56555	200.7017	94.69683	194.01857	77.64741	51.732
180.56555	200.7017	94.69683	194.01857	77.64741	51.732
47.00952	36.144638	68.97457	73.50196	4.9999995	4.9999995
601.1407	689.1999	588.81305	796.38934	152.85103	112.463905
135.99663	87.97118	133.26082	129.9565	47.364567	38.84547
131.6688	194.40936	356.2197	342.34854	66.9885	88.603966
131.6688	194.40936	356.2197	342.34854	66.9885	88.603966
131.6688	194.40936	356.2197	342.34854	66.9885	88.603966
131.6688	194.40936	356.2197	342.34854	66.9885	88.603966
131.6688	194.40936	356.2197	342.34854	66.9885	88.603966
131.6688	194.40936	356.2197	342.34854	66.9885	88.603966
1642.2494	4285.3066	1983.8239	3662.2761	551.3971	314.89
1642.2494	4285.3066	1983.8239	3662.2761	551.3971	314.89
33.009315	37.571056	35.308098	38.251728	10.818241	8.468103
64.28922	77.103905	89.495674	103.27752	26.17869	36.62752
176.12343	142.12689	150.58508	199.77562	35.4599	33.812145
35.45471	35.477825	32.328995	35.341644	7.62253	4.9999995
26.28726	37.088074	21.832447	36.240814	7.799296	9.7194805
247.63095	182.35034	290.78256	288.06525	86.74403	45.696003
48.710976	57.438816	38.650307	39.376633	12.303448	9.948744
2973.0486	47.7859	1354.6466	1051.9677	17.695988	25.00003
34.988617	215.92072	43.66661	127.58443	7.357041	4.9999995
1594.3597	1780.6747	4295.491	4159.8833	525.84265	468.84515
297.89545	122.28673	202.30779	210.16377	36.370785	36.88919
55.070248	24.543531	83.37892	83.641556	5.3744483	4.9999995
70.94139	34.768696	186.93358	152.88983	69.397705	104.83638
70.4396	101.34652	44.38505	89.1405	19.530653	10.077979
70.4396	101.34652	44.38505	89.1405	19.530653	10.077979
16.907341	54.843758	19.327581	36.03341	7.062478	8.032354
65.65369	101.45879	116.48138	147.27675	25.376774	28.671265
43.54297	52.42647	21.31461	53.630234	21.28663	13.219311
38.012466	28.020136	29.71823	39.566673	6.651694	7.2578444
38.012466	28.020136	29.71823	39.566673	6.651694	7.2578444
59.961018	43.09852	21.611227	19.716356	18.298338	5.8172097
1253.7899	1166.8506	821.3006	1138.7711	353.526	185.4167
1253.7899	1166.8506	821.3006	1138.7711	353.526	185.4167
1253.7899	1166.8506	821.3006	1138.7711	353.526	185.4167
1253.7899	1166.8506	821.3006	1138.7711	353.526	185.4167
94.87556	933.6107	29.112125	475.10678	4.9999995	4.9999995
204.44832	639.54364	234.23422	472.49594	55.61591	65.90955
470.00665	278.20428	506.5902	447.7007	92.84358	48.679302
177.07391	38.19937	46.372948	55.15151	5.439171	4.9999995

11.361469	6.038408	7.659488	4.9999995	5.1884604	5.753668
8.982451	4.9999995	4.9999995	5.2766743	3.8205779	4.14771
772.824	221.76556	285.93115	406.48224	10.583677	10.761944
31.714602	14.61185	20.16885	11.931257	5.570574	6.050665
31.714602	14.61185	20.16885	11.931257	5.570574	6.050665
31.714602	14.61185	20.16885	11.931257	5.570574	6.050665
31.714602	14.61185	20.16885	11.931257	5.570574	6.050665
31.714602	14.61185	20.16885	11.931257	5.570574	6.050665
138.29358	52.355648	23.618233	106.14529	7.8612537	7.559444
11.843279	6.4537134	11.605099	6.0595393	4.8995256	5.476765
11.843279	6.4537134	11.605099	6.0595393	4.8995256	5.476765
11.843279	6.4537134	11.605099	6.0595393	4.8995256	5.476765
142.5774	130.99821	196.63225	138.96364	8.9556465	9.116504
142.5774	130.99821	196.63225	138.96364	8.9556465	9.116504
125.89549	47.41436	40.41776	43.112965	8.488871	7.324397
25.192959	25.712206	17.210077	20.326672	6.218515	6.6592035
37.51885	6.7215695	17.036522	7.266637	5.520516	4.6544566
72.14134	14.617041	34.786674	27.511364	6.9682217	6.521834
8.279044	36.79544	12.64665	22.374348	5.6255145	4.3227577
851.89984	202.22154	332.91678	256.85855	9.596456	8.756732
86.68817	35.258255	37.51167	50.00646	7.6208763	7.764816
86.68817	35.258255	37.51167	50.00646	7.6208763	7.764816
8.791377	4.9999995	23.298748	14.430729	5.3618145	5.3962736
212.47934	119.2367	177.40019	117.593155	8.856418	8.876472
46.941406	35.420345	39.645477	32.593784	6.691194	7.057556
55.270905	80.759514	55.681976	57.122627	7.9342217	8.613312
55.270905	80.759514	55.681976	57.122627	7.9342217	8.613312
55.270905	80.759514	55.681976	57.122627	7.9342217	8.613312
55.270905	80.759514	55.681976	57.122627	7.9342217	8.613312
55.270905	80.759514	55.681976	57.122627	7.9342217	8.613312
55.270905	80.759514	55.681976	57.122627	7.9342217	8.613312
735.9117	616.70325	1116.5596	670.45294	10.53047	10.729852
735.9117	616.70325	1116.5596	670.45294	10.53047	10.729852
26.364466	8.095316	12.014589	10.422209	5.3189397	5.5381618
15.641069	35.31418	29.925968	19.14035	6.3592005	6.8468814
26.758799	42.833782	26.182262	25.543129	7.036479	7.2189813
11.72257	7.368085	10.295169	12.7849	4.5944915	5.0130816
11.90632	8.146295	4.9999995	9.336588	4.3953185	4.535953
130.5667	78.02035	89.55344	76.28767	7.306882	8.972078
16.758894	14.600421	21.943161	11.2349615	5.471082	5.9140425
7.6397343	28.453693	30.911163	24.094334	10.244089	7.2733502
9.378756	12.469851	5.4306126	10.45047	4.2485285	3.5126534
565.263	401.43088	731.8774	444.56934	11.4742365	12.122962
45.06956	55.3383	118.064384	73.91688	7.9539247	8.087202
12.972341	11.268029	5.219436	9.760259	4.878085	3.8519087
26.724142	36.02392	13.0935	21.594498	8.373123	9.21453
18.39479	15.065028	44.130295	27.926348	6.011666	6.7500806
18.39479	15.065028	44.130295	27.926348	6.011666	6.7500806
14.10939	7.7896695	9.376892	11.900041	4.4894514	4.9411626
31.586996	32.61732	33.102028	22.137194	6.3684483	6.656224
22.789928	14.815331	7.954725	7.350442	5.178852	5.1954083
9.506561	6.451747	9.326593	5.874494	4.869189	4.9926424
9.506561	6.451747	9.326593	5.874494	4.869189	4.9926424
32.23415	6.561763	12.95513	13.474449	5.830849	5.2916236
483.38516	228.78009	504.32852	291.25906	9.982735	10.0338955
483.38516	228.78009	504.32852	291.25906	9.982735	10.0338955
483.38516	228.78009	504.32852	291.25906	9.982735	10.0338955
483.38516	228.78009	504.32852	291.25906	9.982735	10.0338955
5.4040866	24.268799	4.9999995	12.278718	5.472554	4.032456
58.642868	122.534615	55.119324	77.44234	7.354245	7.1611257
159.13542	83.93907	108.931274	80.74902	8.967152	9.111512
4.9999995	4.9999995	8.463089	4.9999995	6.5717063	5.1868286

3.756371	4.5664573	4.9209423	5.0643563	2.7717695	2.4279919
3.5475922	3.8226523	3.7907338	3.5955896	2.3467326	3.2481415
10.30017	10.513674	9.88764	10.051797	9.267637	9.013053
5.4549794	5.4589586	6.024882	5.8248787	4.7685995	4.1600113
5.4549794	5.4589586	6.024882	5.8248787	4.7685995	4.1600113
5.4549794	5.4589586	6.024882	5.8248787	4.7685995	4.1600113
5.4549794	5.4589586	6.024882	5.8248787	4.7685995	4.1600113
5.4549794	5.4589586	6.024882	5.8248787	4.7685995	4.1600113
7.5354524	7.9422145	6.669238	7.2237377	6.3626533	6.4054785
4.2165093	4.9297	4.169465	4.6868076	3.7166622	2.4279919
4.2165093	4.9297	4.169465	4.6868076	3.7166622	2.4279919
4.2165093	4.9297	4.169465	4.6868076	3.7166622	2.4279919
9.019658	9.191443	8.78512	8.921026	7.5460916	8.121407
9.019658	9.191443	8.78512	8.921026	7.5460916	8.121407
9.093034	6.8550367	7.876892	7.5239215	6.461682	6.3575416
5.5986714	6.2975616	6.5729055	6.640818	4.9361496	5.904049
6.0606613	5.3980017	3.8577545	4.3700776	4.408697	3.4658408
7.232593	6.510322	5.2818365	5.87541	5.8075056	5.5724835
6.576168	7.731335	4.9836664	6.863506	4.000017	4.4020104
9.933219	10.031924	9.276599	9.617431	8.717515	7.5399895
7.1697836	7.331037	6.352173	7.0769625	6.501225	6.5415435
7.1697836	7.331037	6.352173	7.0769625	6.501225	6.5415435
5.2364244	4.7426925	5.9062	5.687501	2.3467326	2.4279919
8.863566	9.124042	8.941606	9.058694	7.496756	7.6624255
6.7632737	6.104297	6.8325105	6.497655	5.7973332	6.118871
6.7161136	7.2840753	8.218967	7.8594213	6.303389	7.317497
6.7161136	7.2840753	8.218967	7.8594213	6.303389	7.317497
6.7161136	7.2840753	8.218967	7.8594213	6.303389	7.317497
6.7161136	7.2840753	8.218967	7.8594213	6.303389	7.317497
6.7161136	7.2840753	8.218967	7.8594213	6.303389	7.317497
6.7161136	7.2840753	8.218967	7.8594213	6.303389	7.317497
10.31841	11.708961	10.655523	11.229724	9.383503	9.154838
10.31841	11.708961	10.655523	11.229724	9.383503	9.154838
4.732257	4.8015223	4.9886355	4.787864	3.6624749	3.8515859
5.7011576	5.9060836	6.2758226	6.170115	4.953069	6.0431924
7.131299	6.8253646	7.016437	7.1162405	5.3816147	5.9182405
4.831638	4.712561	4.870487	4.68232	3.124553	2.4279919
4.4054723	4.78062	4.344045	4.7145166	3.161067	4.0606484
7.6211963	7.1873198	7.9418917	7.6265297	6.668021	6.361072
5.2894835	5.4600086	5.116288	4.8252106	3.8508828	4.0965295
11.161579	5.1810617	10.13089	9.454793	4.383066	5.474477
4.8133607	7.445708	5.289249	6.4694476	3.0713778	2.4279919
10.273834	10.48608	11.766797	11.420417	9.314806	9.747459
7.883682	6.6030097	7.4227605	7.1796627	5.42145	6.0528355
5.4688582	4.134693	6.1779957	5.866915	2.63139	2.4279919
5.836652	4.6824737	7.3133464	6.732897	6.350574	7.5572824
5.8269167	6.314351	5.311865	5.9601197	4.5239863	4.1154904
5.8269167	6.314351	5.311865	5.9601197	4.5239863	4.1154904
3.762033	5.3890414	4.174975	4.7056756	3.0139115	3.7706938
5.731268	6.316223	6.646162	6.67738	4.9103575	5.678822
5.1209946	5.3206434	4.31089	5.2535625	4.6503224	4.526578
4.9343333	4.3432484	4.7512555	4.8322387	2.9290867	3.622164
4.9343333	4.3432484	4.7512555	4.8322387	2.9290867	3.622164
5.5946236	5.0176053	4.330019	3.8686814	4.428994	3.280585
9.927347	9.87209	9.416045	9.5666065	8.72701	8.386008
9.927347	9.87209	9.416045	9.5666065	8.72701	8.386008
9.927347	9.87209	9.416045	9.5666065	8.72701	8.386008
9.927347	9.87209	9.416045	9.5666065	8.72701	8.386008
6.246205	9.54743	4.7249584	8.318831	2.3467326	2.4279919
7.361637	9.006942	7.626415	8.31159	6.035231	6.899699
8.52641	7.803236	8.728725	8.237523	6.767985	6.4538713
7.138297	4.8281054	5.366543	5.293379	2.6458135	2.4279919

3.6012063	2.6997633	3.2333555	2.352752
3.2878168	2.3408759	2.3618152	2.671153
9.542218	8.201783	8.407669	9.205354
5.0014496	4.00406	4.598633	3.8780541
5.0014496	4.00406	4.598633	3.8780541
5.0014496	4.00406	4.598633	3.8780541
5.0014496	4.00406	4.598633	3.8780541
5.0014496	4.00406	4.598633	3.8780541
7.0668764	5.985796	4.8330617	7.1965575
3.654477	2.792818	3.8112333	2.8594089
3.654477	2.792818	3.8112333	2.8594089
3.654477	2.792818	3.8112333	2.8594089
7.114	7.4012246	7.866164	7.609486
7.114	7.4012246	7.866164	7.609486
6.93584	5.8271317	5.6087933	5.849493
4.6782026	4.8758636	4.3766584	4.6830854
5.2322984	2.8495529	4.3662977	3.1256516
6.145634	4.004725	5.3939104	5.1547284
3.1797569	5.4333124	3.9357905	4.8278956
9.677465	8.060068	8.613576	8.545436
6.4086394	5.3698177	5.4982743	6.0709443
6.4086394	5.3698177	5.4982743	6.0709443
3.2567592	2.3408759	4.8164535	4.164312
7.677998	7.254087	7.7133527	7.348425
5.552009	5.3766575	5.580455	5.4200087
5.7815433	6.6631455	6.064305	6.276834
5.7815433	6.6631455	6.064305	6.276834
5.7815433	6.6631455	6.064305	6.276834
5.7815433	6.6631455	6.064305	6.276834
5.7815433	6.6631455	6.064305	6.276834
5.7815433	6.6631455	6.064305	6.276834
9.467373	9.735577	10.373614	9.947801
9.467373	9.735577	10.373614	9.947801
4.7432575	3.106084	3.8631744	3.6733012
4.031947	5.3725133	5.1794367	4.588553
4.7644362	5.6615515	4.9800606	5.0322485
3.6432493	2.9727907	3.6427283	3.979651
3.6623552	3.1157691	2.3618152	3.506844
6.9852676	6.6092434	6.73819	6.705492
4.125649	4.002897	4.7289863	3.78435
3.0751512	5.0359163	5.223381	4.9418535
3.3456976	3.756605	2.779275	3.6763659
9.088825	9.098459	9.746593	9.339301
5.491871	6.0714536	7.132695	6.6573205
3.7749643	3.6037025	2.729733	3.5740688
4.762749	5.402174	3.9852204	4.7754884
4.255297	4.0515842	5.732545	5.1774917
4.255297	4.0515842	5.732545	5.1774917
3.8896964	3.0499225	3.5148478	3.8746445
4.9963174	5.240079	5.3195934	4.811007
4.5414867	4.025834	3.2858248	3.144012
3.3615243	2.792315	3.5072186	2.813815
3.3615243	2.792315	3.5072186	2.813815
5.0232916	2.8144748	3.9718282	4.0647054
8.873232	8.247959	9.211855	8.722403
8.873232	8.247959	9.211855	8.722403
8.873232	8.247959	9.211855	8.722403
8.873232	8.247959	9.211855	8.722403
2.6664345	4.7837806	2.3618152	3.9204235
5.860233	7.2986503	6.0498543	6.729886
7.2669153	6.7183876	7.023465	6.78902
2.350709	2.3408759	3.3722532	2.352752

ASHGA5P056040	0.000593543	0.016411961	3.3850043	up	noncoding
ASHGA5P044370	0.006202958	0.050241924	3.8241574	up	noncoding
ASHGA5P044370	0.006202958	0.050241924	3.8241574	up	noncoding
ASHGA5P022599	0.000411884	0.014498655	15.7475623	up	noncoding
ASHGA5P030420	1.45369E-05	0.004434727	2.1715569	up	noncoding
ASHGA5P037800	0.001315764	0.024205782	3.4845925	up	noncoding
ASHGA5P040385	0.011093531	0.065002686	3.0581461	up	noncoding
ASHGA5P040385	0.011093531	0.065002686	3.0581461	up	noncoding
ASHGA5P040385	0.011093531	0.065002686	3.0581461	up	noncoding
ASHGA5P032705	0.048615314	0.143206684	9.1650282	up	noncoding
ASHGA5P043889	0.009262779	0.059833628	2.0353458	up	noncoding
ASHGA5P047975	0.004334763	0.041729925	2.7486954	up	noncoding
ASHGA5P000521	0.017741106	0.080853294	2.1509967	up	noncoding
ASHGA5P054916	7.34484E-05	0.008283845	2.7423079	up	noncoding
ASHGA5P029332	0.027056606	0.102679848	2.2137161	up	noncoding
ASHGA5P056970	0.002374431	0.031849965	3.4884686	up	noncoding
ASHGA5P049233	0.007664987	0.054952989	4.0235158	up	noncoding
ASHGA5P033347	0.006747962	0.052096554	2.5192794	up	noncoding
ASHGA5P033347	0.006747962	0.052096554	2.5192794	up	noncoding
ASHGA5P033347	0.006747962	0.052096554	2.5192794	up	noncoding
ASHGA5P022745	0.014994338	0.074989017	2.3312742	up	noncoding
ASHGA5P022745	0.014994338	0.074989017	2.3312742	up	noncoding
ASHGA5P043681	0.000116929	0.009923756	2.040575	up	noncoding
ASHGA5P035417	0.004292029	0.041572876	5.7821429	up	noncoding
ASHGA5P028373	0.015276203	0.075655452	2.0767107	up	noncoding
ASHGA5P057025	0.004930716	0.04447418	2.7443821	up	noncoding
ASHGA5P036743	0.020220443	0.087085259	3.2252967	up	noncoding
ASHGA5P044347	0.005719939	0.048087879	5.4860566	up	noncoding
ASHGA5P031524	0.0009559	0.021023728	2.1617731	up	noncoding
ASHGA5P031524	0.0009559	0.021023728	2.1617731	up	noncoding
ASHGA5P031777	0.003411008	0.037690616	2.0189083	up	noncoding
ASHGA5P054921	0.000347607	0.013409132	2.5253194	up	noncoding
ASHGA5P054921	0.000347607	0.013409132	2.5253194	up	noncoding
ASHGA5P054921	0.000347607	0.013409132	2.5253194	up	noncoding
ASHGA5P035595	0.029745787	0.108120501	2.0921658	up	noncoding
ASHGA5P052792	9.34312E-05	0.009080764	3.6311692	up	noncoding
ASHGA5P037958	7.669E-05	0.008348603	5.0953451	up	noncoding
ASHGA5P020759	0.004080202	0.040799661	2.3681042	up	noncoding
ASHGA5P020759	0.004080202	0.040799661	2.3681042	up	noncoding
ASHGA5P020759	0.004080202	0.040799661	2.3681042	up	noncoding
ASHGA5P029767	0.034952198	0.118300274	2.2888647	up	noncoding
ASHGA5P049520	0.000569043	0.016120398	3.6350821	up	noncoding
ASHGA5P020681	0.026121898	0.100677786	4.1816753	up	noncoding
ASHGA5P013324	0.001716208	0.027747135	2.0458104	up	noncoding
ASHGA5P013324	0.001716208	0.027747135	2.0458104	up	noncoding
ASHGA5P013324	0.001716208	0.027747135	2.0458104	up	noncoding
ASHGA5P013324	0.001716208	0.027747135	2.0458104	up	noncoding
ASHGA5P013324	0.001716208	0.027747135	2.0458104	up	noncoding
ASHGA5P041731	0.002980125	0.03525836	5.115651	up	noncoding
ASHGA5P044053	0.003886196	0.039968017	2.6621515	up	noncoding
ASHGA5P054938	0.005147417	0.045573729	3.103416	up	noncoding
ASHGA5P040769	0.009322987	0.059922247	2.0076979	up	noncoding
ASHGA5P025909	0.003987611	0.040458123	2.0977174	up	noncoding
ASHGA5P028273	0.013197221	0.070372979	2.650688	up	noncoding
ASHGA5P028273	0.013197221	0.070372979	2.650688	up	noncoding
ASHGA5P028273	0.013197221	0.070372979	2.650688	up	noncoding
ASHGA5P033138	0.043375686	0.133429762	2.0137079	up	noncoding
ASHGA5P030955	0.008002374	0.056265282	2.7795156	up	noncoding
ASHGA5P030955	0.008002374	0.056265282	2.7795156	up	noncoding
ASHGA5P018873	1.08983E-05	0.004010044	2.8051811	up	noncoding
ASHGA5P014887	0.01045986	0.063149534	4.5721492	up	noncoding
ASHGA5P014887	0.01045986	0.063149534	4.5721492	up	noncoding

ENST00000555377	RP11-72M17.1	GENCODE	286	chr14	-
ENST00000566000	RP11-219B4.7	GENCODE	1264	chr8	-
ENST00000566000	RP11-219B4.7	GENCODE	1264	chr8	-
ENST00000559432	CRNDE	GENCODE	587	chr16	-
TCONS_00017282	XLOC_008100	LincRNAs identified	1287	chrX	-
ENST00000565162	AC004463.6	GENCODE	1438	chr22	+
ENST00000507251	CTD-2203K17.1	GENCODE	537	chr5	-
ENST00000507251	CTD-2203K17.1	GENCODE	537	chr5	-
ENST00000507251	CTD-2203K17.1	GENCODE	537	chr5	-
ENST00000431729	RP11-191N8.2	GENCODE	423	chr1	-
uc010llg.1	BC150500	UCSC_knowngene	478	chr7	+
ENST00000424948	RP1-92O14.3	GENCODE	661	chr1	-
chr8:20622500-2063	chr8:20622500-2063	LincRNAs identified	11950	chr8	-
ENST00000535076	SNHG1	GENCODE	849	chr11	-
ENST00000555776	RP11-61O1.1	GENCODE	542	chr14	-
TCONS_00011696	XLOC_005175	LincRNAs identified	438	chr6	+
TCONS_00014161	XLOC_006144	LincRNAs identified	579	chr7	+
ENST00000579037	RP11-661C3.2	GENCODE	321	chr17	+
ENST00000579037	RP11-661C3.2	GENCODE	321	chr17	+
ENST00000579037	RP11-661C3.2	GENCODE	321	chr17	+
ENST00000561968	CRYM-AS1	GENCODE	559	chr16	+
ENST00000561968	CRYM-AS1	GENCODE	559	chr16	+
uc003tqy.3	CO9	UCSC_knowngene	505	chr7	+
ENST00000442456	AC007405.4	GENCODE	2026	chr2	-
ENST00000544419	RP5-944M2.2	GENCODE	480	chr12	+
TCONS_00012063	XLOC_005592	LincRNAs identified	346	chr6	-
TCONS_00026722	XLOC_012857	LincRNAs identified	275	chr18	-
ENST00000522183	RP11-697M17.1	GENCODE	833	chr8	-
ENST00000564919	RP11-77H9.5	GENCODE	409	chr16	+
ENST00000564919	RP11-77H9.5	GENCODE	409	chr16	+
ENST00000566383	RP11-44F14.2	GENCODE	584	chr16	+
ENST00000539921	SNHG1	GENCODE	1071	chr11	-
ENST00000539921	SNHG1	GENCODE	1071	chr11	-
ENST00000539921	SNHG1	GENCODE	1071	chr11	-
uc001bgg.1	AK025975	UCSC_knowngene	3565	chr1	+
ENST00000427501	SNHG5	GENCODE	680	chr6	-
AW262946		LincRNAs identified	426	chr13	-
NR_033193	ZFAND1	RefSeq	2212	chr8	-
NR_033193	ZFAND1	RefSeq	2212	chr8	-
NR_033193	ZFAND1	RefSeq	2212	chr8	-
ENST00000521812	MEG3	GENCODE	635	chr14	+
NR_045024	C17orf76-AS1	RefSeq	642	chr17	+
ENST00000518376	RP11-1136L8.1	GENCODE	872	chr8	-
NR_028135	KCNMB3	RefSeq	2141	chr3	-
NR_028135	KCNMB3	RefSeq	2141	chr3	-
NR_028135	KCNMB3	RefSeq	2141	chr3	-
NR_028135	KCNMB3	RefSeq	2141	chr3	-
NR_028135	KCNMB3	RefSeq	2141	chr3	-
ENST00000457253	XXbac-BPG308K3.5	GENCODE	709	chr6	-
uc003won.1	BC107568	UCSC_knowngene	932	chr7_gl000195_rand	+
ENST00000544553	RP11-783K16.5	GENCODE	391	chr11	+
CB269930		LincRNAs identified	418	chr1	-
uc002lau.3	LOC284260	UCSC_knowngene	1847	chr18	+
uc001trc.1	BC064974	UCSC_knowngene	1828	chr12	+
uc001trc.1	BC064974	UCSC_knowngene	1828	chr12	+
uc001trc.1	BC064974	UCSC_knowngene	1828	chr12	+
TCONS_00028208	XLOC_013553	LincRNAs identified	3392	chr20	+
ENST00000572706	RP11-485G7.5	GENCODE	505	chr16	-
ENST00000572706	RP11-485G7.5	GENCODE	505	chr16	-
NR_003605	ZNFX1-AS1	RefSeq	695	chr20	+
ENST00000414386	RP11-328M4.2	GENCODE	419	chr6	-
ENST00000414386	RP11-328M4.2	GENCODE	419	chr6	-

66961361	66963052		ATAAAATAGATCTCAGGCTTATGTA'		intergenic
86088033	86089297		TACAACAGCATTAACTAGTAAAGCT		bidirectional
86088033	86089297		TACAACAGCATTAACTAGTAAAGCT		bidirectional
54952977	54962628	uc010vhb.2	AGATTCTGAAGA'	643911	intergenic
2484083	2488088	TCONS_00017281	TCCGTGTGGAGAGAGGGCATAAAG/		intergenic
19158907	19160345		CCTAAGACCCTGTGCAGGGAATCTG		intergenic
33424130	33440725		GCCAGTGTTTGGAATCGACTGGGCT		bidirectional
33424130	33440725		GCCAGTGTTTGGAATCGACTGGGCT		bidirectional
33424130	33440725		GCCAGTGTTTGGAATCGACTGGGCT		bidirectional
222001007	222014008		GGCAGATCACCAAAGAACTCAAGAT		intergenic
127700597	127701075		TACCTGGAAAGCAGTGCATGTATCA		intron sense-overlapp
43820354	43824329		TTAAATCCTACACGAGAGTCGTATT'		bidirectional
20578220	20590170		AAATAGTGGCCTCTCCTTCTAGAAA		intergenic
62619519	62621168		TCTGATTTTCTGA	23642	intergenic
98534576	98671480		TTCTTCTCCCGGATTTTTCTTTCAAT'		intergenic
12322519	12335036		ACTTGTCATGCAAACTCTGTTCCTTC		intergenic
73668831	73669533		TTTTCCAGATTGCTCTGAATGTCCTA		intergenic
71239231	71240845		AAGTACAAGATAACGATGAATTCTG		intron sense-overlapp
71239231	71240845		AAGTACAAGATAACGATGAATTCTG		intron sense-overlapp
71239231	71240845		AAGTACAAGATAACGATGAATTCTG		intron sense-overlapp
21312201	21328207		ACCCAAAAGGAGTTGAACTTCCTCT		intronic antisense
21312201	21328207		ACCCAAAAGGAGTTGAACTTCCTCT		intronic antisense
55840871	55841376	NM_004577	CAATTAAACACCTTTACTCTTAGGA		intergenic
171579718	171627276		CATGTATTTTTCCCACTGATGTTCAC		intergenic
126910535	126918554		CCCTCTCCAAGCGTGTTAATAAAAT		intergenic
5047436	5054329	TCONS_00012062	CTGGATGAAGTACAGCTCTGAAGTC		intergenic
57053838	57056221		AAAGCCTTGATGGTCCAGCCCTCGA		intergenic
76135937	76236976	uc003yao.3	CTTCAAAATAGCTATTTTAATTTTGC		intergenic
8947168	8948204		TCTTTTCTGGATGGATCGGGACAAA		natural antisense
8947168	8948204		TCTTTTCTGGATGGATCGGGACAAA		natural antisense
53407404	53418171		AACACCTTAGGTGAATACCGAGCTC		intergenic
62619459	62622708	ENST00000539975,	ATTTTTTCAGATC	23642	bidirectional
62619459	62622708	ENST00000539975,	ATTTTTTCAGATC	23642	bidirectional
62619459	62622708	ENST00000539975,	ATTTTTTCAGATC	23642	bidirectional
23243782	23247347		CATTCACTCACAACCAGCCTTTTTGC		intergenic
86386799	86387711	ENST00000431043	GACTAGCAGCTTCATTTTGAAGTAG		intergenic
28393227	28393643		CCAGTCTAGGTTTATGCCAGACATT(		intergenic
82613565	82633539		CACTAACTTGAC/	79752	exon sense-overlappi
82613565	82633539		CACTAACTTGAC/	79752	exon sense-overlappi
82613565	82633539		CACTAACTTGAC/	79752	exon sense-overlappi
101298400	101302560	ENST00000452120	CTTACCCTGGCGC	55384	intergenic
16342300	16344525	ENST00000460249,	CCAAGAGAAGGT	125144	intergenic
128742439	128746213		CCTATGGAGAACCGAGACACAAAAC		intergenic
178957536	178977679		AATGTAAAAGCG'	27094	exon sense-overlappi
178957536	178977679		AATGTAAAAGCG'	27094	exon sense-overlappi
178957536	178977679		AATGTAAAAGCG'	27094	exon sense-overlappi
178957536	178977679		AATGTAAAAGCG'	27094	exon sense-overlappi
178957536	178977679		AATGTAAAAGCG'	27094	exon sense-overlappi
28805645	28806783		CGTCGCCAAATTGACGAGCTCATTA		intergenic
137966	141360		AAGGAAAGAAGGTGTGTTTTGCCTG		intergenic
64013435	64015689		CTGCATTTCCTCAGAAGGCTGTAAC		intronic antisense
153487636	153488055		AATTCTGATCATTCTCTGCAGCATGC		intergenic
39766634	40271389		TTTATAGGAGATGGACCTTCTCCTA		intergenic
110939418	110941246		AATTTCTCTCTCATGACTTGTTGTGA		exon sense-overlappi
110939418	110941246		AATTTCTCTCTCATGACTTGTTGTGA		natural antisense
110939418	110941246		AATTTCTCTCTCATGACTTGTTGTGA		natural antisense
47129834	47133716		TTCCTAGAATACACATTATCCCCAG		intergenic
11438207	11439054	ENST00000572913	GCGTGGATGCAGATCACCTACAGAC		intronic antisense
11438207	11439054	ENST00000572913	GCGTGGATGCAGATCACCTACAGAC		intronic antisense
47894714	47905795		GAGCGGTTTGGT'	441951	natural antisense
41491632	41513914		ACGTGGGGAGAAGACGTGAGGCGG		bidirectional
41491632	41513914		ACGTGGGGAGAAGACGTGAGGCGG		bidirectional

NM_001083588	E2F5	transcription factor E	+	86089618	86126753
NM_001951	E2F5	transcription factor E	+	86089618	86126753
NM_001258437	TARS	threonine--tRNA liga	+	33440801	33468196
NM_001258438	TARS	threonine--tRNA liga	+	33440881	33468196
NM_152295	TARS	threonine--tRNA liga	+	33440881	33468196
NM_014390	SND1	staphylococcal nucle	+	127292201	127732659
NM_001255	CDC20	cell division cycle pr	+	43824625	43828873
NM_001100621	C17orf80	uncharacterized prote	+	71228775	71245095
NM_001100622	C17orf80	uncharacterized prote	+	71228775	71241483
NM_017941	C17orf80	uncharacterized prote	+	71228775	71245095
NM_001014444	CRYM	thiomorpholine-carbo	-	21269838	21314404
NM_001888	CRYM	thiomorpholine-carbo	-	21269838	21314404
NM_001042476	CARHSP1	calcium-regulated he	-	8946801	8962248
NM_014316	CARHSP1	calcium-regulated he	-	8946801	8962863
NM_001012662	SLC3A2	4F2 cell-surface antig	+	62623483	62656355
NM_001012664	SLC3A2	4F2 cell-surface antig	+	62623483	62656355
NM_002394	SLC3A2	4F2 cell-surface antig	+	62623483	62656355
NM_001170796	ZFAND1	AN1-type zinc finger	-	82613565	82633539
NM_001170797	ZFAND1	AN1-type zinc finger	-	82615148	82633539
NM_024699	ZFAND1	AN1-type zinc finger	-	82613565	82633539
NM_001163677	KCNMB3	calcium-activated po	-	178957536	178984838
NM_014407	KCNMB3	calcium-activated po	-	178960553	178969403
NM_171828	KCNMB3	calcium-activated po	-	178960593	178984838
NM_171829	KCNMB3	calcium-activated po	-	178960553	178977679
NM_171830	KCNMB3	calcium-activated po	-	178960553	178969645
NM_138689	PPP1R14B	protein phosphatase 1	-	64011950	64014413
NM_152442	RAD9B	cell cycle checkpoint	+	110940004	110969891
NM_016226	VPS29	vacuolar protein sorti	-	110929329	110939916
NM_057180	VPS29	vacuolar protein sorti	-	110929329	110939916
ENST00000381820	RMI2	RMI2, RecQ mediate	+	11410635	11445585
ENST00000572173	RMI2	RMI2, RecQ mediate	+	11343505	11445612
NM_021035	ZNFX1	NFX1-type zinc fing	-	47862438	47894756
NM_001012426	FOXP4	forkhead box protein	+	41514163	41570122
NM_001012427	FOXP4	forkhead box protein	+	41514163	41570122

1618.9195	262.339625	10.111807	8.35265	2104.2886	2901.4353
31.723381	5	4.298621	2.363479	17.13327	17.813341
31.723381	5	4.298621	2.363479	17.13327	17.813341
2729.035967	100.030867	10.737284	6.760227	2034.6936	603.3609
3469.42945	924.28275	11.333155	10.214425	3662.8499	3815.0286
519.465797	87.841063	8.530637	6.729647	366.44388	338.92502
71.737949	20.739598	5.704649	4.091992	88.529465	36.3292
71.737949	20.739598	5.704649	4.091992	88.529465	36.3292
71.737949	20.739598	5.704649	4.091992	88.529465	36.3292
298.650764	12.113269	6.905054	3.708914	236.48227	8.894643
33.210147	12.002232	4.700394	3.67512	44.24674	37.71664
48.786393	11.734265	5.191888	3.733141	53.9724	22.294762
149.840512	47.487162	6.810961	5.705955	185.81056	186.83902
253.242768	58.296739	7.568486	6.113095	227.38388	199.8161
27.026553	10.566668	4.420744	3.274274	38.29485	22.736473
52.0114	8.764409	5.131016	3.328423	40.844135	31.747396
231.518498	39.582923	7.310523	5.302067	143.85498	345.15118
74.983198	27.979359	5.777331	4.44432	105.6531	66.63724
74.983198	27.979359	5.777331	4.44432	105.6531	66.63724
74.983198	27.979359	5.777331	4.44432	105.6531	66.63724
54.559849	15.65677	5.392696	4.171577	64.290054	71.06122
54.559849	15.65677	5.392696	4.171577	64.290054	71.06122
24781.38067	7183.216167	14.040146	13.01117	16274.697	11547.056
129.486578	12.586477	6.457797	3.926193	127.45136	62.325577
56.048644	19.195185	5.413783	4.359483	70.111786	76.148285
22.422238	6.185258	4.066898	2.610416	27.758102	40.186024
389.105417	86.89057	8.072127	6.382695	450.80524	547.16455
1031.589208	199.714247	9.367676	6.911906	1102.169	196.41245
49.80663	15.830693	5.302868	4.190653	59.76152	42.471844
49.80663	15.830693	5.302868	4.190653	59.76152	42.471844
32.266829	10.175817	4.625304	3.611728	34.52872	47.335224
174.690402	43.89686	7.056174	5.719708	173.22546	165.0632
174.690402	43.89686	7.056174	5.719708	173.22546	165.0632
174.690402	43.89686	7.056174	5.719708	173.22546	165.0632
64.50954	17.874329	5.368178	4.30318	88.943794	32.93287
1924.87315	308.903853	10.488011	8.627577	2024.627	1791.2313
37.82921	5.445983	4.861681	2.512501	52.569588	23.710636
2368.702433	549.148935	10.709108	9.465375	1897.5563	1458.5419
2368.702433	549.148935	10.709108	9.465375	1897.5563	1458.5419
2368.702433	549.148935	10.709108	9.465375	1897.5563	1458.5419
225.66806	54.317453	7.207258	6.012626	318.34482	454.6232
1407.781932	209.924955	9.896628	8.03464	1585.9286	2458.969
188.177339	28.177514	7.002098	4.938017	269.45743	211.65163
163.00749	48.934394	6.972859	5.940186	151.37888	152.00891
163.00749	48.934394	6.972859	5.940186	151.37888	152.00891
163.00749	48.934394	6.972859	5.940186	151.37888	152.00891
163.00749	48.934394	6.972859	5.940186	151.37888	152.00891
163.00749	48.934394	6.972859	5.940186	151.37888	152.00891
52.417835	5.825891	5.045911	2.690993	51.90862	73.06636
2104.71625	520.364073	10.610727	9.198134	2217.2979	1980.9501
1825.297127	337.610982	10.190347	8.556489	2436.1965	2750.3032
47.118603	16.149734	5.180857	4.175315	52.92627	59.073006
39.004981	13.009643	4.962138	3.893318	41.860023	42.156918
151.156558	34.163843	6.732473	5.326106	158.50299	108.41011
151.156558	34.163843	6.732473	5.326106	158.50299	108.41011
151.156558	34.163843	6.732473	5.326106	158.50299	108.41011
990.463313	247.328368	9.233797	8.223943	1387.1831	2116.4756
52.71805	12.218524	5.296848	3.822015	57.555443	80.45047
52.71805	12.218524	5.296848	3.822015	57.555443	80.45047
3230.228267	623.183223	11.145688	9.657594	3699.8901	5177.1865
679.765795	85.14315	8.80545	6.612578	1053.9535	1244.8535
679.765795	85.14315	8.80545	6.612578	1053.9535	1244.8535

1097.629	1525.4426	711.915	1372.8065	244.5615	266.36566
13.915919	85.555824	11.69844	44.22349	4.9999995	4.9999995
13.915919	85.555824	11.69844	44.22349	4.9999995	4.9999995
2748.3386	5227.087	1963.7413	3796.9944	86.061195	68.0867
3178.3132	3412.757	2833.3145	3914.3135	1075.6653	797.18414
389.89536	930.6297	381.63275	709.26807	78.28988	49.03814
136.11949	53.62543	50.25066	65.57345	23.74774	4.9999995
136.11949	53.62543	50.25066	65.57345	23.74774	4.9999995
136.11949	53.62543	50.25066	65.57345	23.74774	4.9999995
440.2243	43.835293	569.48004	492.98804	19.716278	18.245802
30.414648	23.11506	30.998959	32.768837	17.549286	8.654367
66.84649	34.08966	58.219593	57.295452	14.053141	11.094371
110.041306	81.334724	168.0709	166.94656	41.315205	20.228199
238.63812	161.19594	349.79965	342.62292	47.86084	43.289394
27.807753	29.828344	20.727026	22.764874	15.009498	4.9999995
50.40181	16.80438	95.24234	77.028336	5.2379613	10.401559
197.30775	83.46931	387.4402	231.88757	46.918453	16.852772
127.985275	49.70292	41.41702	58.50363	44.942913	9.074251
127.985275	49.70292	41.41702	58.50363	44.942913	9.074251
127.985275	49.70292	41.41702	58.50363	44.942913	9.074251
44.78246	61.50396	31.434263	54.287136	15.6625595	8.708936
44.78246	61.50396	31.434263	54.287136	15.6625595	8.708936
18678.31	28155.197	35186.016	38847.008	5165.4546	1714.602
156.96039	225.77856	37.49144	166.91214	12.90472	12.87347
46.93244	34.107895	56.601418	52.39004	27.525679	9.601515
15.663509	16.890411	15.239091	18.796291	12.111548	4.9999995
235.21834	135.82593	504.57626	461.04218	112.96613	44.807762
1892.1351	1038.682	787.0278	1173.1089	313.7765	15.836058
48.270954	61.657406	41.35399	45.324066	20.041502	7.6154137
48.270954	61.657406	41.35399	45.324066	20.041502	7.6154137
17.674799	37.140945	23.966444	32.95484	9.189742	10.98802
154.78699	114.32738	215.95612	224.78326	51.62463	43.55814
154.78699	114.32738	215.95612	224.78326	51.62463	43.55814
154.78699	114.32738	215.95612	224.78326	51.62463	43.55814
161.86378	30.053051	34.095837	39.167908	22.176622	5.714157
2023.7184	2148.008	1396.0842	2165.57	303.93674	249.84442
53.045147	34.86541	24.68291	38.10157	5.061469	4.9999995
1976.5459	3716.345	1764.2201	3399.0054	616.9821	484.6839
1976.5459	3716.345	1764.2201	3399.0054	616.9821	484.6839
1976.5459	3716.345	1764.2201	3399.0054	616.9821	484.6839
105.54912	80.50785	205.60881	189.37456	59.593803	36.003574
617.07104	720.80035	1568.8315	1495.0911	149.7709	187.80077
272.50293	209.6519	48.074223	117.72592	21.449911	30.25274
134.03429	202.43822	126.65777	211.52687	51.53218	54.55114
134.03429	202.43822	126.65777	211.52687	51.53218	54.55114
134.03429	202.43822	126.65777	211.52687	51.53218	54.55114
134.03429	202.43822	126.65777	211.52687	51.53218	54.55114
134.03429	202.43822	126.65777	211.52687	51.53218	54.55114
17.319313	15.4301605	85.892654	70.8899	6.188349	8.767001
2382.302	2459.4045	1435.489	2152.854	566.71466	195.36674
1600.8632	363.54736	2055.2896	1745.5829	472.2814	292.2322
49.222446	28.61416	43.85022	49.025517	20.999214	9.214676
39.302776	41.03944	34.98738	34.68335	15.04539	7.249063
141.28922	285.4441	52.91247	160.38046	25.194042	15.109979
141.28922	285.4441	52.91247	160.38046	25.194042	15.109979
141.28922	285.4441	52.91247	160.38046	25.194042	15.109979
452.80505	249.59193	958.2098	778.5144	318.88336	198.31015
23.626837	50.900356	50.403275	53.371918	9.577639	7.2754292
23.626837	50.900356	50.403275	53.371918	9.577639	7.2754292
1759.3752	3442.3928	2148.0125	3154.5125	628.3699	676.8368
741.25977	334.68777	322.47833	381.3619	76.07163	116.274124
741.25977	334.68777	322.47833	381.3619	76.07163	116.274124

187.25656	442.89038	182.21568	250.74797	10.560705	11.078003
4.9999995	4.9999995	4.9999995	4.9999995	3.626508	4.15219
4.9999995	4.9999995	4.9999995	4.9999995	3.626508	4.15219
81.02024	223.02692	29.667559	112.32259	10.505876	8.922743
1306.1084	860.4668	809.4441	696.82776	11.353091	11.4803095
125.09459	72.86224	120.210815	81.55071	8.0526285	8.120192
46.406925	4.9999995	27.85439	16.428537	6.016551	5.091117
46.406925	4.9999995	27.85439	16.428537	6.016551	5.091117
46.406925	4.9999995	27.85439	16.428537	6.016551	5.091117
6.3264046	10.50435	4.9999995	12.886781	7.4327593	3.255536
23.722738	6.7766004	9.49986	5.8105416	5.0100737	5.1415067
20.643576	6.767854	10.2371	7.6095476	5.2870874	4.441683
101.06403	33.931587	53.430958	34.952995	7.0825233	7.3042655
48.98447	39.13927	105.44821	65.05825	7.3776712	7.3933673
19.906694	4.9999995	13.48382	4.9999995	4.7928348	4.466393
5.4916024	5.532404	14.672351	11.250579	4.8894477	4.915486
101.63322	21.32186	28.541845	22.229387	6.7189436	8.150463
83.0843	12.04145	6.744853	11.988389	6.276982	5.8839192
83.0843	12.04145	6.744853	11.988389	6.276982	5.8839192
83.0843	12.04145	6.744853	11.988389	6.276982	5.8839192
9.598226	14.81401	27.05291	18.103981	5.548009	5.972224
9.598226	14.81401	27.05291	18.103981	5.548009	5.972224
8591.55	7161.6157	12427.543	8038.5317	13.527157	13.046157
10.524141	12.680081	17.26646	9.269992	6.544166	5.7970476
37.911335	12.98444	16.27762	10.87052	5.672883	6.0635896
4.9999995	4.9999995	4.9999995	4.9999995	4.3425303	5.221648
238.57013	43.03125	42.56751	39.40064	8.340542	8.78552
648.0203	29.960062	123.86549	66.82707	9.632255	7.372594
23.8241	15.030681	13.410601	15.061862	5.443587	5.297964
23.8241	15.030681	13.410601	15.061862	5.443587	5.297964
12.786059	12.510451	7.677003	7.9036283	4.647783	5.4383473
51.07383	26.149918	59.420742	31.553898	6.9846425	7.1248007
51.07383	26.149918	59.420742	31.553898	6.9846425	7.1248007
51.07383	26.149918	59.420742	31.553898	6.9846425	7.1248007
24.864458	13.947139	22.09406	18.449535	6.0225396	4.965386
347.76086	264.2424	419.0752	268.5635	10.500728	10.405479
4.9999995	7.614431	4.9999995	4.9999995	5.2485094	4.526578
734.2601	555.6099	490.12146	413.23615	10.409206	10.1362505
734.2601	555.6099	490.12146	413.23615	10.409206	10.1362505
734.2601	555.6099	490.12146	413.23615	10.409206	10.1362505
97.29504	46.278873	42.348827	44.384598	7.8598895	8.52151
215.21484	170.41788	318.69202	217.65332	10.16291	10.843684
6.3661065	52.94368	24.282045	33.7706	7.614466	7.470888
54.971794	47.08346	45.02015	40.44764	6.7857003	7.0109687
54.971794	47.08346	45.02015	40.44764	6.7857003	7.0109687
54.971794	47.08346	45.02015	40.44764	6.7857003	7.0109687
54.971794	47.08346	45.02015	40.44764	6.7857003	7.0109687
54.971794	47.08346	45.02015	40.44764	6.7857003	7.0109687
4.9999995	4.9999995	4.9999995	4.9999995	5.2322044	6.0072627
992.81714	275.69547	680.68787	410.90256	10.637513	10.535038
693.2391	169.62485	238.20753	160.08081	10.765995	11.013427
26.917606	13.80181	17.50175	8.463349	5.2574396	5.7232103
22.275015	11.21692	8.713397	13.558071	4.92615	5.288127
35.80072	43.399963	49.208797	36.26956	6.850599	6.5413547
35.80072	43.399963	49.208797	36.26956	6.850599	6.5413547
35.80072	43.399963	49.208797	36.26956	6.850599	6.5413547
424.36426	149.82433	213.99434	178.59377	9.967988	10.629704
18.663992	12.420909	15.664891	9.708283	5.384713	6.1372123
18.663992	12.420909	15.664891	9.708283	5.384713	6.1372123
488.4312	801.43964	542.6534	601.3684	11.374494	11.874908
28.370453	63.914978	123.1409	103.086815	9.568935	9.920622
28.370453	63.914978	123.1409	103.086815	9.568935	9.920622

9.727071	10.256634	9.21145	9.836981	8.19243	8.922047
3.4712727	6.0635896	3.4931533	4.9850144	2.3467326	2.4279919
3.4712727	6.0635896	3.4931533	4.9850144	2.3467326	2.4279919
11.055225	12.01908	10.641948	11.278831	6.6556563	6.942311
11.253813	11.399617	11.1805	11.331601	10.374018	10.500271
8.256207	9.541262	8.319926	8.893606	6.5142684	6.4654284
6.764355	5.355869	5.474269	5.525732	4.811007	2.4279919
6.764355	5.355869	5.474269	5.525732	4.811007	2.4279919
6.764355	5.355869	5.474269	5.525732	4.811007	2.4279919
8.433738	5.043697	8.894863	8.369729	4.5369043	5.011071
4.618368	4.041284	4.8119617	4.5791698	4.3711762	3.8884676
5.755429	4.6513805	5.675479	5.3402696	4.040421	4.26334
6.463109	5.9885716	7.1676574	6.859638	5.6024175	5.1689596
7.574728	7.0110326	8.193386	7.860731	5.812874	6.281397
4.4878187	4.4438443	4.2696924	4.063881	4.140906	2.4279919
5.339797	3.5207248	6.362803	5.7578397	2.5981882	4.164884
7.309578	6.028187	8.344078	7.3118896	5.781161	4.890941
6.6784725	5.242817	5.214876	5.3669186	5.720097	3.956422
6.6784725	5.242817	5.214876	5.3669186	5.720097	3.956422
6.6784725	5.242817	5.214876	5.3669186	5.720097	3.956422
5.1656566	5.566763	4.8322387	5.2712846	4.1987853	3.8973804
5.1656566	5.566763	4.8322387	5.2712846	4.1987853	3.8973804
13.924151	14.477176	14.691475	14.574759	12.655313	11.6324625
6.9594364	7.510168	5.0766845	6.8592806	3.9159763	4.483424
5.2342877	4.65289	5.636147	5.222901	5.0186114	4.041816
3.6479502	3.528763	3.8534138	3.8070812	3.8283544	2.4279919
7.554172	6.75786	8.721255	8.273411	7.05524	6.3301992
10.529305	9.704905	9.357468	9.609529	8.564204	4.8031573
5.2758613	5.5704274	5.21203	5.0173407	4.559358	3.6882637
5.2758613	5.5704274	5.21203	5.0173407	4.559358	3.6882637
3.8226523	4.7827826	4.472847	4.587409	3.413261	4.247084
6.9397388	6.501886	7.511914	7.274063	5.9257536	6.290818
6.9397388	6.501886	7.511914	7.274063	5.9257536	6.290818
6.9397388	6.501886	7.511914	7.274063	5.9257536	6.290818
7.0055604	4.4554214	4.941365	4.8187933	4.7074065	3.254265
10.629704	10.736275	10.172247	10.483632	8.515199	8.821781
5.410706	4.687648	4.5133567	4.7832866	2.5640197	2.4279919
10.587137	11.514311	10.490754	11.116988	9.555412	9.785257
10.587137	11.514311	10.490754	11.116988	9.555412	9.785257
10.587137	11.514311	10.490754	11.116988	9.555412	9.785257
6.404019	5.972224	7.4453163	7.0405917	6.1351995	6.015731
8.896035	9.185014	10.337179	9.9549465	7.4649067	8.407342
7.755659	7.397591	5.4155555	6.3584294	4.660527	5.7585306
6.7419257	7.344145	6.763552	7.1908607	5.923102	6.620828
6.7419257	7.344145	6.763552	7.1908607	5.923102	6.620828
6.7419257	7.344145	6.763552	7.1908607	5.923102	6.620828
6.7419257	7.344145	6.763552	7.1908607	5.923102	6.620828
6.7419257	7.344145	6.763552	7.1908607	5.923102	6.620828
3.7938292	3.3852472	6.2194715	5.6374507	2.83208	3.9077265
10.875775	10.9322405	10.208466	10.475327	9.423069	8.470265
10.280668	8.181859	10.720492	10.179638	9.150122	9.053056
5.304887	4.3778663	5.2947197	5.127019	4.631235	3.9798257
4.9819813	4.9431906	4.9767184	4.6566615	4.144236	3.6200628
6.8194356	7.8401413	5.5431128	6.800193	4.8980455	4.732089
6.8194356	7.8401413	5.5431128	6.800193	4.8980455	4.732089
6.8194356	7.8401413	5.5431128	6.800193	4.8980455	4.732089
8.47346	7.6548643	9.6525545	9.024214	8.587746	8.489169
4.253834	5.2777257	5.4796605	5.2479434	3.478808	3.6240544
4.253834	5.2777257	5.4796605	5.2479434	3.478808	3.6240544
10.419105	11.409957	10.782239	11.013427	9.58526	10.256634
9.185387	8.069192	8.083456	8.005108	6.4734306	7.7106805
9.185387	8.069192	8.083456	8.005108	6.4734306	7.7106805

7.50101	9.238177	7.757298	8.504936
2.350709	2.3408759	2.3618152	2.352752
2.350709	2.3408759	2.3618152	2.352752
6.30517	8.210383	5.168317	7.279526
10.288283	10.22388	9.895008	10.005093
6.9282484	6.5080023	7.158849	6.8030853
5.5353613	2.3408759	5.075209	4.361504
5.5353613	2.3408759	5.075209	4.361504
5.5353613	2.3408759	5.075209	4.361504
2.8514273	3.4979916	2.3618152	3.994277
4.5967603	2.8618555	3.5339692	2.7984915
4.40739	2.8589454	3.6353405	3.1934094
6.621921	5.3061304	6.0082607	5.5280437
5.6131773	5.525881	6.976121	6.4691224
4.358217	2.3408759	4.0249	2.352752
2.6839373	2.5896928	4.14717	3.7866628
6.6288233	4.5820193	5.1119375	4.817517
6.3448944	3.7032332	3.0564768	3.884795
6.3448944	3.7032332	3.0564768	3.884795
6.3448944	3.7032332	3.0564768	3.884795
3.3755584	4.02563	5.0290437	4.503066
3.3755584	4.02563	5.0290437	4.503066
13.027055	13.278634	13.8879	13.585656
3.4992163	3.781071	4.381456	3.4960139
5.2458887	3.8185854	4.2967763	3.735219
2.350709	2.3408759	2.3618152	2.352752
7.8448186	5.6697598	5.685433	5.710718
9.284413	5.1153426	7.198279	6.506043
4.601352	4.0480566	4.0174313	4.2294583
4.601352	4.0480566	4.0174313	4.2294583
3.7574043	3.7616313	3.235806	3.2551823
5.6682925	4.8995256	6.159394	5.374466
5.6682925	4.8995256	6.159394	5.374466
5.6682925	4.8995256	6.159394	5.374466
4.6603103	3.9279792	4.739351	4.5297704
8.388277	8.478105	8.953883	8.608215
2.350709	3.0177178	2.3618152	2.352752
9.464466	9.580778	9.169953	9.236385
9.464466	9.580778	9.169953	9.236385
9.464466	9.580778	9.169953	9.236385
6.5651445	5.7898073	5.6769366	5.8929386
7.6942043	7.7867117	8.557705	8.296971
2.8592703	6.001797	4.872952	5.4750257
5.7719064	5.816449	5.7611837	5.7476487
5.7719064	5.816449	5.7611837	5.7476487
5.7719064	5.816449	5.7611837	5.7476487
5.7719064	5.816449	5.7611837	5.7476487
5.7719064	5.816449	5.7611837	5.7476487
2.350709	2.3408759	2.3618152	2.352752
9.8901825	8.544217	9.638668	9.222402
9.383112	7.779081	8.149844	7.8237214
4.7728124	3.9104486	4.4020104	3.3555567
4.5102587	3.598264	3.4145653	4.07252
5.1659017	5.683706	5.8925796	5.5843134
5.1659017	5.683706	5.8925796	5.5843134
5.1659017	5.683706	5.8925796	5.5843134
8.681981	7.600642	7.9956307	7.9884896
4.2714405	3.7510884	4.241205	3.5654917
4.2714405	3.7510884	4.241205	3.5654917
8.8879595	10.117644	9.314806	9.783263
4.848854	6.2990246	7.1931014	7.150374
4.848854	6.2990246	7.1931014	7.150374

ASHGA5P014887	0.01045986	0.063149534	4.5721492	up	noncoding
ASHGA5P045880	0.00845124	0.057522562	2.2049152	up	noncoding
ASHGA5P022359	0.002575394	0.032855824	2.1335703	up	noncoding
ASHGA5P045028	0.001185203	0.023096268	3.6820409	up	noncoding
ASHGA5P041906	0.004152119	0.041138537	2.1642458	up	noncoding
ASHGA5P041906	0.004152119	0.041138537	2.1642458	up	noncoding
ASHGA5P041906	0.004152119	0.041138537	2.1642458	up	noncoding
ASHGA5P048422	0.00056374	0.016101946	2.398143	up	noncoding
ASHGA5P030640	0.005314763	0.046390935	2.2626943	up	noncoding
ASHGA5P017388	0.017539755	0.080495745	2.6182577	up	noncoding
ASHGA5P040638	0.003954456	0.040405952	2.3926077	up	noncoding
ASHGA5P040638	0.003954456	0.040405952	2.3926077	up	noncoding
ASHGA5P021404	0.002531392	0.032654253	2.283424	up	noncoding
ASHGA5P028900	0.008657065	0.058001887	3.4027216	up	noncoding
ASHGA5P023339	0.000272626	0.012302262	2.247572	up	noncoding
ASHGA5P052689	0.021627649	0.090752239	2.5822204	up	noncoding
ASHGA5P030387	0.013690177	0.071698418	2.6646257	up	noncoding
ASHGA5P029552	0.00356323	0.038481895	3.6347998	up	noncoding
ASHGA5P026041	0.005125704	0.045497848	2.8395486	up	noncoding
ASHGA5P019506	0.011724241	0.066689086	2.1534914	up	noncoding
ASHGA5P048553	0.001667504	0.027358136	2.1344125	up	noncoding
ASHGA5P030947	0.000810408	0.019071379	2.4392689	up	noncoding
ASHGA5P000116	0.002134637	0.030485504	4.7372513	up	noncoding
ASHGA5P025995	0.005934312	0.049073045	2.3549067	up	noncoding
ASHGA5P025995	0.005934312	0.049073045	2.3549067	up	noncoding
ASHGA5P025995	0.005934312	0.049073045	2.3549067	up	noncoding
ASHGA5P025995	0.005934312	0.049073045	2.3549067	up	noncoding
ASHGA5P025995	0.005934312	0.049073045	2.3549067	up	noncoding
ASHGA5P025995	0.005934312	0.049073045	2.3549067	up	noncoding
ASHGA5P025995	0.005934312	0.049073045	2.3549067	up	noncoding
ASHGA5P044743	0.007955137	0.056185826	4.3426706	up	noncoding
ASHGA5P038215	0.016804637	0.078960288	4.0145475	up	noncoding
ASHGA5P034343	0.001885708	0.02911661	2.7280626	up	noncoding
ASHGA5P057892	0.020518111	0.087886164	2.4538737	up	noncoding
ASHGA5P027696	0.003683154	0.038896709	17.9711102	up	noncoding
ASHGA5P033503	0.000952622	0.021003113	2.8749459	up	noncoding
ASHGA5P048336	0.016356369	0.078078429	2.4249653	up	noncoding
ASHGA5P044072	0.01224925	0.067744715	2.6136247	up	noncoding
ASHGA5P023567	0.004670435	0.043513602	2.7280627	up	noncoding
ASHGA5P037228	0.018730408	0.08321024	2.8138237	up	noncoding
ASHGA5P053573	0.020897704	0.088917985	2.2305383	up	noncoding
ASHGA5P053262	0.001060396	0.022031747	2.176224	up	noncoding
ASHGA5P014028	0.007035556	0.052947146	2.5393138	up	noncoding
ASHGA5P042495	0.020348649	0.087506775	2.0889793	up	noncoding
ASHGA5P047650	0.028431403	0.105108746	2.2473286	up	noncoding
ASHGA5P022821	0.000398784	0.014223693	3.0100697	up	noncoding
ASHGA5P019234	0.002520538	0.03258252	2.2386059	up	noncoding
ASHGA5P019234	0.002520538	0.03258252	2.2386059	up	noncoding
ASHGA5P030541	0.003046383	0.03574339	2.7813412	up	noncoding
ASHGA5P030541	0.003046383	0.03574339	2.7813412	up	noncoding
ASHGA5P044573	0.004732978	0.043738988	3.4631541	up	noncoding
ASHGA5P018515	0.001431581	0.025327629	3.1072061	up	noncoding
ASHGA5P018515	0.001431581	0.025327629	3.1072061	up	noncoding
ASHGA5P018515	0.001431581	0.025327629	3.1072061	up	noncoding
ASHGA5P033644	0.048777581	0.143521005	2.8582194	up	noncoding
ASHGA5P044229	0.00201138	0.029606317	2.5626929	up	noncoding
ASHGA5P028560	0.001862294	0.028981608	3.4395242	up	noncoding
ASHGA5P043238	1.95683E-05	0.004838692	2.9105228	up	noncoding
ASHGA5P043238	1.95683E-05	0.004838692	2.9105228	up	noncoding
ASHGA5P043238	1.95683E-05	0.004838692	2.9105228	up	noncoding
ASHGA5P044200	8.3709E-06	0.004010044	3.5830757	up	noncoding
ASHGA5P044202	0.002154038	0.030562731	3.6857986	up	noncoding

ENST00000414386	RP11-328M4.2	GENCODE	419	chr6	-
TCONS_00001692	XLOC_001050	LincRNAs identified	451	chr1	-
ENST00000555186	CHEK2P2	GENCODE	864	chr15	+
ENST00000520913	PVT1	GENCODE	584	chr8	+
ENST00000423099	RP11-505P4.6	GENCODE	370	chr6	-
ENST00000423099	RP11-505P4.6	GENCODE	370	chr6	-
ENST00000423099	RP11-505P4.6	GENCODE	370	chr6	-
ENST00000532422	RP11-677I18.3	GENCODE	826	chr11	-
ENST00000562313	RP11-226L15.5	GENCODE	1842	chr1	-
ENST00000449351	SNHG11	GENCODE	954	chr20	+
ENST00000503529	CTC-210G5.1	GENCODE	579	chr5	-
ENST00000503529	CTC-210G5.1	GENCODE	579	chr5	-
ENST00000531887	OR5G5P	GENCODE	163	chr11	-
NR_027350	MIR17HG	RefSeq	5018	chr13	+
ENST00000578831	RP11-739L10.1	GENCODE	570	chr18	-
NR_044997	HCG25	RefSeq	703	chr6	+
ENST00000507787	HERC2P9	GENCODE	1023	chr15	+
ENST00000555969	RP11-589M4.1	GENCODE	898	chr14	+
ENST00000564354	RP11-680F20.10	GENCODE	808	chr11	-
NR_051968	MRPS2	RefSeq	1714	chr9	+
ENST00000566448	CHEK2P2	GENCODE	670	chr15	+
ENST00000566787	RP11-27M24.3	GENCODE	502	chr16	-
BG185325		LincRNAs identified	236	chr8	+
NR_049781	VRK2	RefSeq	1916	chr2	+
NR_049781	VRK2	RefSeq	1916	chr2	+
NR_049781	VRK2	RefSeq	1916	chr2	+
NR_049781	VRK2	RefSeq	1916	chr2	+
NR_049781	VRK2	RefSeq	1916	chr2	+
NR_049781	VRK2	RefSeq	1916	chr2	+
NR_049781	VRK2	RefSeq	1916	chr2	+
ENST00000412855	RP11-396C23.2	GENCODE	407	chr1	+
uc001czn.3	BC042048	UCSC_knowngene	528	chr1	+
TCONS_00003752	XLOC_001550	LincRNAs identified	930	chr2	+
TCONS_00023349	XLOC_011211	LincRNAs identified	393	chr15	+
ENST00000397340	RP11-474D1.3	GENCODE	3528	chr12	-
ENST00000581725	RP11-146G7.3	GENCODE	1902	chr18	-
ENST00000571090	RP11-111M22.3	GENCODE	2608	chr11	-
ENST00000520570	RP11-134O21.1	GENCODE	582	chr8	-
ENST00000584686	SNHG15	GENCODE	541	chr7	-
ENST00000446404	AP000402.3	GENCODE	540	chr21	-
ENST00000440959	RP5-1180C10.2	GENCODE	2547	chr1	-
ENST00000437450	AC002467.5	GENCODE	670	chr7	+
uc001cjy.3	LOC100132774	UCSC_knowngene	924	chr1	-
TCONS_00020749	XLOC_010037	LincRNAs identified	1077	chr12	-
ENST00000442188	RP11-107I14.2	GENCODE	382	chr10	+
NR_037620	ORC6	RefSeq	1703	chr16	+
NR_033835	PIGA	RefSeq	3270	chrX	-
NR_033835	PIGA	RefSeq	3270	chrX	-
ENST00000559672	RP11-108K3.2	GENCODE	794	chr15	+
ENST00000559672	RP11-108K3.2	GENCODE	794	chr15	+
ENST00000517411	RP13-582O9.5	GENCODE	645	chr8	-
NR_033231	SMPD4	RefSeq	3777	chr2	-
NR_033231	SMPD4	RefSeq	3777	chr2	-
NR_033231	SMPD4	RefSeq	3777	chr2	-
ENST00000579923	RP11-106E15.1	GENCODE	302	chr18	-
ENST00000524133	RP11-360L9.7	GENCODE	570	chr8	-
uc001vdv.3	DLEU2	UCSC_knowngene	972	chr13	-
ENST00000412754	RP11-126L15.4	GENCODE	369	chr7	-
ENST00000412754	RP11-126L15.4	GENCODE	369	chr7	-
ENST00000412754	RP11-126L15.4	GENCODE	369	chr7	-
ENST00000520431	RP11-527N22.2	GENCODE	578	chr8	-
ENST00000519691	RP11-150O12.3	GENCODE	2379	chr8	-

41491632	41513914		ACGTGGGGAGAAGACGTGAGGCGG(	bidirectional
157019845	157021546		CCGTCAGCTATTCAATCAAGCATTG.	intergenic
20487996	20496811		GGCAGAATAGAT' 646096	intergenic
128902891	128944941		AGACATGGTACC' 5820	intergenic
74201747	74202465		GAGAGCTCTGCAAATATATGAAAGT	natural antisense
74201747	74202465		GAGAGCTCTGCAAATATATGAAAGT	natural antisense
74201747	74202465		GAGAGCTCTGCAAATATATGAAAGT	natural antisense
105956716	105972649		TCCTAAGAGCAACTGCAACAAAATG	natural antisense
159994742	159996584		GCTGTTCTGGCACTGTAAACTTGAA(	intergenic
37075343	37079564	ENST00000440918	CCCAAGGCCGGC(128439	intergenic
121772948	121814775		GGTTTTTGGATATGAATTTGTCATT/	intronic antisense
121772948	121814775		GGTTTTTGGATATGAATTTGTCATT/	intronic antisense
56569551	56587123		TTTATTATAGTGGTGTGATCATCTTC	intergenic
92000073	92006829		GGAAAGAAAGCA 407975	intergenic
20279443	20513727		TCCGGGTGGGGCAGAAGAATCTTTT	intronic antisense
33217312	33222667		CAGCAGCCTCCG(414765	natural antisense
28834637	28836269		AAAGTCGTGTAG(440248	intergenic
53241954	53244458		CAGAGGACCTTAGATGTCCCACTAT	natural antisense
125742925	125744293		ATTTTTTTGTGGTGTTTGTCTTATTG	intergenic
138392476	138396519		AACCTGTAAGGT' 51116	exon sense-overlappi
20488734	20496839		TGAATTTTAGATT 646096	intergenic
10608698	10622059		CGGCATAAAGTAGAACATTCTTACA	intergenic
122348053	122424931		CCACATTGGAACAAGGCTTCTTAAG	intergenic
58273776	58387055		TCCCCACAAATA/ 7444	exon sense-overlappi
58273776	58387055		TCCCCACAAATA/ 7444	exon sense-overlappi
58273776	58387055		TCCCCACAAATA/ 7444	exon sense-overlappi
58273776	58387055		TCCCCACAAATA/ 7444	exon sense-overlappi
58273776	58387055		TCCCCACAAATA/ 7444	exon sense-overlappi
58273776	58387055		TCCCCACAAATA/ 7444	natural antisense
58273776	58387055		TCCCCACAAATA/ 7444	natural antisense
226274581	226277993	uc001hpx.3	GCAAACAAATCTTTACTTGATGAGA	intergenic
60238466	60254501		ATGTCAAAATTCCCTTCTCCATAGG'	intergenic
85190234	85194331		AAGAGTCCGTATGCTGGAAAAGCAC	intergenic
36743228	36777325		AAAGCAAAAGAAACCAGCATATCAT	intergenic
130517998	130526887	ENST00000538806,	CAGATATTTAGATAATATTTTCCCA/	intergenic
6919494	6929804		CAAGAAGAAAGCTTCCCTACACATT	intergenic
76153116	76155724		GTACACCTTGTTACAGAGCTCAGAA	bidirectional
2558973	2585957		GCATTTTGCCCGTTTGTCTGTCATTC	intergenic
45023445	45026267	ENST00000584327	AAGCAGTCTTCA(285958	intergenic
26258989	26275070		GCACTGCCTTGATTGTACACGCCCT'	intergenic
180241736	180245359		GAGAACAGTTATGATTCTTTGCAAA	natural antisense
107383246	107384360		CTCTAAGAGGTCAATGTAATAATCT'	exon sense-overlappi
44165355	44173012	ENST00000398804	TCCAGAAGCAGT(100132774	natural antisense
29294263	29301212		CAACCAGGAAGTGCTGAAAAACGG(	intergenic
103070774	103071262		TCACATTCCCTAAATACTAATGGCA(	intergenic
46723557	46732306		GGGCCCTCAGGA 23594	exon sense-overlappi
15337572	15353676		CATTGCTCAGGG/ 5277	exon sense-overlappi
15337572	15353676		CATTGCTCAGGG/ 5277	exon sense-overlappi
51600433	51614193		AAAGGAAGAGAAACCCATCAACAT(	intronic antisense
51600433	51614193		AAAGGAAGAGAAACCCATCAACAT(	intronic antisense
144362335	144363830	ENST00000523031	GCTTGAAATTCCAAGGTCGATTTTCT	intergenic
130908964	130939330		TCCAGCAAAGTG(55627	exon sense-overlappi
130908964	130939330		TCCAGCAAAGTG(55627	exon sense-overlappi
130908964	130939330		TCCAGCAAAGTG(55627	exon sense-overlappi
66420415	66421820		GCACGAAAAGTGTAAGAATTGACAT	intronic antisense
41397899	41402563		AATTTACTGTGGTTTTAGAGGATGT.	natural antisense
50618047	50619368	ENST00000235290,	CTTGAACCCTTTT 8847	intergenic
100434935	100450238		TACGGGCGGGTCGTCCTCTCCACCA'	bidirectional
100434935	100450238		TACGGGCGGGTCGTCCTCTCCACCA'	bidirectional
100434935	100450238		TACGGGCGGGTCGTCCTCTCCACCA'	bidirectional
37262956	37264242		GTGACAGAGTTTGCATAGAGATGGC	intergenic
37454997	37457376		CAGAACAAATGCATGCAAACCTTGC	intergenic

NM_138457	FOXP4	forkhead box protein	+	41514163	41570122
NM_001123226	MTO1	protein MTO1 homo	+	74171453	74211179
NM_012123	MTO1	protein MTO1 homo	+	74171453	74211179
NM_133645	MTO1	protein MTO1 homo	+	74171453	74211179
NM_015423	AASDHPPT	L-aminoadipate-semi	+	105948291	105969419
NM_001242935	SNCAIP	synphilin-1 isoform 1	+	121647819	121799794
NM_005460	SNCAIP	synphilin-1 isoform 1	+	121647819	121799794
NM_002894	RBBP8	DNA endonuclease F	+	20513294	20606449
NM_022553	VPS52	vacuolar protein sorti	-	33218048	33239662
NM_198066	GNPNAT1	glucosamine 6-phosp	-	53241910	53258386
NM_016034	MRPS2	28S ribosomal protei	+	138392476	138396519
NM_001130480	VRK2	serine/threonine-prot	+	58273776	58387055
NM_001130481	VRK2	serine/threonine-prot	+	58273846	58387055
NM_001130482	VRK2	serine/threonine-prot	+	58273776	58387055
NM_001130483	VRK2	serine/threonine-prot	+	58273776	58387055
NM_006296	VRK2	serine/threonine-prot	+	58273776	58387055
NM_001114636	FANCL	E3 ubiquitin-protein	-	58386377	58468515
NM_018062	FANCL	E3 ubiquitin-protein	-	58386377	58468515
NM_020193	C11orf30	protein EMSY	+	76156068	76262589
NM_033343	LHX4	LIM/homeobox prote	+	180199432	180244188
NM_024814	CBLL1	E3 ubiquitin-protein	+	107384278	107402083
NM_014663	KDM4A	lysine-specific demet	+	44115796	44171189
NM_014321	ORC6	origin recognition co	+	46723557	46732306
NM_002641	PIGA	phosphatidylinositol	-	15337572	15353676
NM_020473	PIGA	phosphatidylinositol	-	15337572	15353676
NM_000103	CYP19A1	cytochrome P450 19.	-	51500253	51630795
NM_031226	CYP19A1	cytochrome P450 19.	-	51500253	51630795
NM_001171083	SMPD4	sphingomyelin phosp	-	130908964	130939330
NM_017751	SMPD4	sphingomyelin phosp	-	130908964	130939330
NM_017951	SMPD4	sphingomyelin phosp	-	130908964	130939330
NM_001093729	CCDC102B	coiled-coil domain-c	+	66382490	66722426
NM_032336	GINS4	DNA replication com	+	41386724	41402565
NM_001267812	SLC12A9	solute carrier family	+	100450340	100463160
NM_001267814	SLC12A9	solute carrier family	+	100450340	100463160
NM_020246	SLC12A9	solute carrier family	+	100450340	100464634

679.765795	85.14315	8.80545	6.612578	1053.9535	1244.8535
50.374123	16.149314	5.292639	4.151916	60.142967	64.14926
1042.7363	296.725387	9.621102	8.527832	1142.3381	1118.856
2077.956883	319.763082	10.506151	8.625646	1822.0737	1507.1025
139.022275	37.641169	6.488675	5.37481	143.12788	232.10596
139.022275	37.641169	6.488675	5.37481	143.12788	232.10596
139.022275	37.641169	6.488675	5.37481	143.12788	232.10596
42.444531	11.704979	5.061332	3.799415	48.35843	43.66983
2364.1251	651.026203	10.741553	9.563511	2372.2085	1999.5952
377.998985	101.261505	8.108989	6.720382	306.33218	492.21564
807.940238	239.097911	9.111008	7.852424	784.28406	304.41248
807.940238	239.097911	9.111008	7.852424	784.28406	304.41248
61.847301	18.182869	5.564838	4.373639	82.47011	69.746
1675.992742	272.054042	10.19032	8.423631	2412.0322	2464.4675
130.518609	36.338358	6.650301	5.481933	161.61023	151.73343
70.887411	19.901299	5.681934	4.313322	80.13581	89.317665
162.962123	48.253804	6.926574	5.512641	206.71596	244.68762
115.812298	21.037967	6.458941	4.597065	96.599106	98.24008
29.657885	7.109764	4.356514	2.850852	34.89477	7.5787725
170.256477	48.636952	6.990521	5.883844	192.83769	223.39728
947.169117	268.997583	9.485969	8.39213	983.9219	1016.94806
176.443362	45.737336	7.088831	5.802383	175.34848	142.4207
50.218072	7.808878	5.147569	2.903519	28.828463	15.312428
364.604675	90.991815	8.073304	6.837634	352.58942	192.00157
364.604675	90.991815	8.073304	6.837634	352.58942	192.00157
364.604675	90.991815	8.073304	6.837634	352.58942	192.00157
364.604675	90.991815	8.073304	6.837634	352.58942	192.00157
364.604675	90.991815	8.073304	6.837634	352.58942	192.00157
364.604675	90.991815	8.073304	6.837634	352.58942	192.00157
364.604675	90.991815	8.073304	6.837634	352.58942	192.00157
859.944907	119.652114	9.272537	7.153954	1077.5099	724.1927
129.591356	17.315431	6.30651	4.301272	138.58441	34.847008
36.944746	9.222667	4.847715	3.399838	32.991272	27.714941
31.987092	9.332176	4.619948	3.324887	43.035675	33.926487
1498.786032	94.616238	9.463438	5.29583	2659.6238	4270.3755
176.730079	34.586455	6.682532	5.158997	221.48785	33.37344
93.340735	29.515703	6.178098	4.900134	82.66092	66.77338
102.227439	20.956216	6.033007	4.646955	150.73607	197.08679
1078.6394	227.666518	9.605766	8.15789	1215.5621	1598.3105
39.14571	7.840596	4.61303	3.120499	66.25631	80.30266
26.819003	8.567705	4.362501	3.20511	25.665838	25.610258
27.784145	8.274089	4.423925	3.302098	38.00504	26.222681
85.646296	20.711472	5.953243	4.608804	124.7942	94.2576
141.075249	42.28462	6.6952	5.632401	179.30476	242.94312
30.912255	12.167679	4.603345	3.435134	44.08478	28.414165
94.546347	20.546574	6.198516	4.608719	97.54164	94.64154
48.788466	14.583297	5.249569	4.086968	52.461105	34.567715
48.788466	14.583297	5.249569	4.086968	52.461105	34.567715
1355.85455	231.953763	9.629662	8.153882	1895.3737	3271.0457
1355.85455	231.953763	9.629662	8.153882	1895.3737	3271.0457
2586.481717	423.647847	10.821391	9.029304	2213.0234	2083.9465
85.117596	17.411954	5.972205	4.336587	110.15943	135.04347
85.117596	17.411954	5.972205	4.336587	110.15943	135.04347
85.117596	17.411954	5.972205	4.336587	110.15943	135.04347
37.456537	7.83041	4.456925	2.941809	30.703587	4.9999995
27.867089	6.78966	4.413724	3.056063	20.81631	18.631714
676.785223	103.399277	8.735659	6.95345	1078.0128	539.2249
461.743705	94.792184	8.438256	6.896978	555.7028	550.7101
461.743705	94.792184	8.438256	6.896978	555.7028	550.7101
461.743705	94.792184	8.438256	6.896978	555.7028	550.7101
22.962252	5	4.204678	2.363479	20.09248	22.170746
7923.217117	1215.774767	12.442371	10.560394	10877.105	11812.968

741.25977	334.68777	322.47833	381.3619	76.07163	116.274124
48.186977	55.6933	32.95054	41.121696	19.533312	8.973085
865.06415	1162.1503	820.87885	1147.1304	226.48688	169.99866
2097.524	3770.5967	1037.4742	2232.9702	273.0964	326.62878
33.312992	215.0929	60.972008	149.52191	31.236282	36.373775
33.312992	215.0929	60.972008	149.52191	31.236282	36.373775
33.312992	215.0929	60.972008	149.52191	31.236282	36.373775
34.765915	46.441833	29.817976	51.6132	12.73324	9.016196
2248.502	3422.2996	1362.2003	2779.945	729.446	313.77896
297.0118	201.42094	544.01135	427.002	113.41691	34.35386
977.28937	418.91592	1175.6917	1187.0479	342.9245	54.360767
977.28937	418.91592	1175.6917	1187.0479	342.9245	54.360767
63.38044	71.36145	31.041702	53.084103	23.722464	8.98363
1783.996	728.08795	1280.4832	1386.8896	247.9306	257.6948
95.774376	124.04309	99.69502	150.25551	40.975346	27.347624
46.511715	30.890625	94.90651	83.56214	23.605902	14.228391
143.68169	96.18614	134.52533	151.976	66.15984	26.70026
79.18693	159.8894	95.24857	165.7097	27.028278	14.222089
40.30842	19.398357	23.16812	52.598873	6.2542086	4.9999995
77.30818	195.1234	154.71353	178.15878	51.48749	38.005444
792.8191	1040.7556	744.02264	1104.5474	207.27559	158.24315
163.40251	153.14838	183.8502	240.4899	30.68631	32.88291
64.59719	79.67975	41.844433	71.046165	8.634929	4.9999995
482.87863	277.10233	392.6686	490.3875	90.3978	77.76285
482.87863	277.10233	392.6686	490.3875	90.3978	77.76285
482.87863	277.10233	392.6686	490.3875	90.3978	77.76285
482.87863	277.10233	392.6686	490.3875	90.3978	77.76285
482.87863	277.10233	392.6686	490.3875	90.3978	77.76285
482.87863	277.10233	392.6686	490.3875	90.3978	77.76285
482.87863	277.10233	392.6686	490.3875	90.3978	77.76285
1040.0919	374.30807	851.51697	1092.0499	143.42285	177.247
197.16302	246.9714	27.82079	132.16151	26.44223	10.390111
29.05639	51.436054	29.703184	50.766632	6.285104	6.7972593
28.733326	42.911194	14.83793	28.477938	4.9999995	5.4958086
672.09125	288.74033	544.20416	557.68115	177.45503	317.88177
387.88788	50.361794	205.88	161.38951	41.223923	8.843164
102.348305	110.121605	77.78452	120.35568	28.403433	5.2174864
98.47845	24.936659	76.53856	65.588104	25.049782	16.00239
1043.2642	1073.7195	543.5009	997.4792	221.00288	262.10852
18.994326	11.773661	32.07835	25.468952	9.155903	6.6837025
15.608109	40.895817	17.686213	35.447784	7.114459	4.9999995
32.9016	16.316732	26.382065	26.876749	8.275476	7.5881114
131.6809	45.071735	51.49704	66.5763	25.067299	9.930493
93.53277	102.2273	106.924805	121.51874	38.997658	23.443058
28.175505	23.58911	32.277412	28.932556	19.973608	5.0696683
63.264385	119.143295	87.31611	105.37111	29.394674	11.672572
60.456924	40.799595	41.34646	63.098995	16.131779	13.206341
60.456924	40.799595	41.34646	63.098995	16.131779	13.206341
311.23187	694.1433	949.29193	1014.0408	263.8068	373.31693
311.23187	694.1433	949.29193	1014.0408	263.8068	373.31693
1870.9268	4398.2285	1534.3047	3418.4604	491.65048	413.0122
65.26337	46.296223	70.98844	82.95464	21.79773	10.9557705
65.26337	46.296223	70.98844	82.95464	21.79773	10.9557705
65.26337	46.296223	70.98844	82.95464	21.79773	10.9557705
61.113564	14.903361	60.502163	52.51655	13.370808	4.9999995
17.308737	38.939735	34.822544	36.683495	7.708559	5.8793364
1447.4685	264.02887	341.40817	390.5681	121.8491	58.854282
453.26834	479.99564	280.25775	450.5276	107.008804	69.86095
453.26834	479.99564	280.25775	450.5276	107.008804	69.86095
453.26834	479.99564	280.25775	450.5276	107.008804	69.86095
20.63054	30.39274	22.338518	22.148487	4.9999995	4.9999995
8661.685	4960.5103	4836.5044	6390.53	1036.653	768.6137

28.370453	63.914978	123.1409	103.086815	9.568935	9.920622
31.794104	11.081131	9.683162	15.831091	5.4512715	5.832843
281.10678	338.2549	438.3374	326.1677	9.68511	9.77977
200.87605	550.39606	221.85207	345.72913	10.352169	10.177752
22.115263	83.314545	14.690829	38.11632	6.7098274	7.60107
22.115263	83.314545	14.690829	38.11632	6.7098274	7.60107
22.115263	83.314545	14.690829	38.11632	6.7098274	7.60107
17.35589	10.560021	11.176979	9.387548	5.13557	5.333505
1380.9827	457.01743	576.66315	448.26898	10.731882	10.548714
244.87872	60.03534	85.41136	69.47284	7.8063107	8.630488
521.6628	88.97867	288.24203	138.4187	9.1556835	7.9789886
521.6628	88.97867	288.24203	138.4187	9.1556835	7.9789886
26.731184	17.69621	10.476661	21.487064	5.9139695	5.948052
191.33873	437.3221	241.98842	256.0496	10.753808	10.847031
48.542282	29.574648	36.406166	35.18408	6.880004	7.009277
49.585896	8.819269	13.8707	9.297635	5.8713107	6.2758226
109.4663	11.5949	53.20358	22.397943	7.238327	7.6682215
33.776894	11.980461	21.985449	17.234629	6.1462817	6.40302
13.085819	4.9999995	8.318557	4.9999995	4.664701	3.0539074
65.347206	32.490585	61.951355	42.53963	7.1401796	7.547325
250.26782	317.87463	379.35953	300.96478	9.474733	9.648809
36.395702	58.342857	61.25263	54.86361	6.9998484	6.923353
16.442488	6.7758503	4.9999995	4.9999995	4.396659	3.955556
82.38914	88.61263	122.17741	84.61106	8.007133	7.3420224
82.38914	88.61263	122.17741	84.61106	8.007133	7.3420224
82.38914	88.61263	122.17741	84.61106	8.007133	7.3420224
82.38914	88.61263	122.17741	84.61106	8.007133	7.3420224
82.38914	88.61263	122.17741	84.61106	8.007133	7.3420224
82.38914	88.61263	122.17741	84.61106	8.007133	7.3420224
82.38914	88.61263	122.17741	84.61106	8.007133	7.3420224
67.000175	166.61488	78.10673	85.52105	9.598484	9.17943
24.58446	11.205129	18.875132	12.395521	6.6602583	5.039991
16.93047	8.208804	7.6584325	9.455935	4.583099	4.737006
18.23903	9.178678	10.370259	7.709279	4.9687037	5.0050797
4.9999995	45.394318	4.9999995	16.96631	10.890528	11.626885
58.986202	19.060303	50.550507	28.85463	7.34337	4.983366
44.898758	22.779152	47.025387	28.769999	5.9166517	5.8860703
27.171923	17.36898	25.60485	14.539369	6.779146	7.376218
120.13419	315.06595	210.0192	237.66837	9.774508	10.251916
12.697461	4.9999995	4.9999995	8.506513	5.591161	6.134455
12.860089	9.249876	10.00439	7.1774154	4.2230234	4.630823
9.092795	7.0647845	11.67079	5.952575	4.7836666	4.6623034
24.543499	21.60845	24.653778	18.465311	6.5128684	6.344739
55.027435	29.022873	68.19404	39.022655	7.0309734	7.65796
29.363583	4.9999995	8.599217	4.9999995	5.004211	4.7705865
19.3434	17.511803	23.989704	21.367292	6.1590247	6.3500853
22.237162	9.38642	16.81343	9.7246475	5.2455544	5.028482
22.237162	9.38642	16.81343	9.7246475	5.2455544	5.028482
154.46819	262.72156	149.45012	187.95898	10.40648	11.253372
154.46819	262.72156	149.45012	187.95898	10.40648	11.253372
668.5521	244.4995	424.9709	299.2019	10.635328	10.609558
26.610575	15.52134	18.577496	11.008811	6.334403	6.844835
26.610575	15.52134	18.577496	11.008811	6.334403	6.844835
26.610575	15.52134	18.577496	11.008811	6.334403	6.844835
11.53463	7.077025	4.9999995	4.9999995	4.4840164	2.3499577
6.58295	6.525096	6.75845	7.2835674	3.9207904	4.2088876
170.00421	81.154564	106.604996	81.92851	9.599325	8.765872
108.119514	111.761086	86.22131	85.78144	8.636986	8.795517
108.119514	111.761086	86.22131	85.78144	8.636986	8.795517
108.119514	111.761086	86.22131	85.78144	8.636986	8.795517
4.9999995	4.9999995	4.9999995	4.9999995	3.868803	4.433914
1501.12	721.8174	1963.2157	1303.2288	12.937954	13.073466

9.185387	8.069192	8.083456	8.005108	6.4734306	7.7106805
5.27341	5.4151297	4.899445	4.883735	4.524444	3.940665
9.403176	9.866453	9.415103	9.577	8.075701	8.256011
10.67865	11.535351	9.762024	10.530962	8.353953	9.211855
4.745681	7.439549	5.7393994	6.6965203	5.199497	6.033301
4.745681	7.439549	5.7393994	6.6965203	5.199497	6.033301
4.745681	7.439549	5.7393994	6.6965203	5.199497	6.033301
4.8057127	5.1352983	4.7566676	5.20124	3.8975403	3.9470742
10.787763	11.401575	10.136795	10.842588	9.811741	9.149743
7.8802357	7.3366446	8.834486	8.165771	7.06087	5.941716
9.560576	8.402716	9.935831	9.632255	8.687116	6.614456
9.560576	8.402716	9.935831	9.632255	8.687116	6.614456
5.6802406	5.7919316	4.814247	5.2405853	4.8094206	3.9419389
10.439191	9.201448	10.049246	9.851195	8.212599	8.866066
6.2588153	6.6233706	6.4259686	6.704368	5.5922737	5.6084337
5.219773	4.5024333	6.356907	5.8653555	4.8020067	4.6423035
6.8427434	6.2388854	6.848012	6.723254	6.2843747	5.5719695
5.990555	7.0019712	6.3630323	6.848785	4.994567	4.641411
5.0170918	3.7510884	4.4243255	5.227968	2.844999	2.4279919
5.958491	7.289172	7.0561333	6.951828	5.922295	6.089942
9.280705	9.708057	9.2780285	9.525483	7.940966	8.155844
7.0180564	6.9317765	7.2939515	7.366003	5.1729603	5.8777623
5.7079835	5.9564347	5.2283545	5.6404276	3.3201697	2.4279919
8.564828	7.7982454	8.364308	8.363289	6.7256374	7.1320114
8.564828	7.7982454	8.364308	8.363289	6.7256374	7.1320114
8.564828	7.7982454	8.364308	8.363289	6.7256374	7.1320114
8.564828	7.7982454	8.364308	8.363289	6.7256374	7.1320114
8.564828	7.7982454	8.364308	8.363289	6.7256374	7.1320114
8.564828	7.7982454	8.364308	8.363289	6.7256374	7.1320114
8.564828	7.7982454	8.364308	8.363289	6.7256374	7.1320114
9.647693	8.228717	9.475051	9.505845	7.395313	8.313027
7.308626	7.641995	4.668123	6.5200653	4.9652867	4.162794
4.552782	5.2899013	4.750262	5.1732373	2.8509417	3.5248582
4.5374103	5.0115256	3.819745	4.3772216	2.3467326	3.19566
9.027562	7.8594213	8.834897	8.541335	7.72486	9.171236
8.250728	5.2615566	7.447236	6.8089333	5.5998073	3.919333
6.3615	6.43821	6.0764217	6.3897324	5.0632734	3.111527
6.301192	4.1620407	6.053564	5.525881	4.88923	4.819709
9.653972	9.747459	8.833395	9.373349	8.039367	8.896035
3.9279792	2.9514513	4.8580565	4.21508	3.408402	3.500977
3.6418579	4.937386	4.0559616	4.685957	3.0235991	2.4279919
4.7282124	3.4752138	4.6017704	4.292384	3.2548506	3.6831756
6.7164097	5.0883117	5.508582	5.5485444	4.890824	4.0939226
6.224653	6.3266964	6.525436	6.4054785	5.517062	5.380506
4.5068436	4.0704474	4.8689246	4.3990583	4.553592	3.0711236
5.676843	6.5643873	6.2416773	6.1990805	5.1091943	4.3354993
5.60712	4.93216	5.2118726	5.472224	4.2443795	4.5249004
5.60712	4.93216	5.2118726	5.472224	4.2443795	4.5249004
7.9459085	9.134197	9.641011	9.397005	8.302611	9.413319
7.9459085	9.134197	9.641011	9.397005	8.302611	9.413319
10.51125	11.745006	10.304794	11.12241	9.2082615	9.553489
5.72143	5.130092	5.947675	5.8547955	4.6839733	4.24366
5.72143	5.130092	5.947675	5.8547955	4.6839733	4.24366
5.72143	5.130092	5.947675	5.8547955	4.6839733	4.24366
5.6231155	3.330637	5.7285686	5.225256	3.9688778	2.4279919
3.7928545	4.8600335	4.967521	4.732257	3.141049	3.3003044
10.121954	7.7294717	8.1610365	8.036295	7.161021	6.7297325
8.474561	8.592521	7.884934	8.245016	6.9781785	6.9742694
8.474561	8.592521	7.884934	8.245016	6.9781785	6.9742694
8.474561	8.592521	7.884934	8.245016	6.9781785	6.9742694
4.048906	4.47694	4.376144	4.0233607	2.3467326	2.4279919
12.73972	11.940308	11.934742	12.028038	10.32528	10.446188

4.848854	6.2990246	7.1931014	7.150374
5.0043287	3.5806754	3.556489	4.304893
8.080805	8.847735	9.015858	8.890884
7.600642	9.568935	8.047681	8.970808
4.5011563	6.7064342	4.1490726	5.6594
4.5011563	6.7064342	4.1490726	5.6594
4.5011563	6.7064342	4.1490726	5.6594
4.169465	3.506844	3.759072	3.516492
10.377842	9.284052	9.40276	9.354929
7.886544	6.1977954	6.669768	6.5656
8.981082	6.8084383	8.419452	7.6040025
8.981082	6.8084383	8.419452	7.6040025
4.7632113	4.2889414	3.6699626	4.7683587
7.5304184	9.221022	8.172513	8.539166
5.599554	5.0947185	5.4592195	5.5374002
5.630499	3.238737	4.0658145	3.5005684
6.739199	3.6484337	6.001797	4.830072
5.0909204	3.6937294	4.731419	4.430343
3.788291	2.3408759	3.3502028	2.352752
6.0102873	5.234422	6.2192883	5.826829
7.9146185	8.760494	8.806071	8.774788
5.1906757	6.1569686	6.202991	6.212938
4.097216	2.8611693	2.3618152	2.352752
6.332508	6.8006306	7.1805944	6.8544245
6.332508	6.8006306	7.1805944	6.8544245
6.332508	6.8006306	7.1805944	6.8544245
6.332508	6.8006306	7.1805944	6.8544245
6.332508	6.8006306	7.1805944	6.8544245
6.332508	6.8006306	7.1805944	6.8544245
6.332508	6.8006306	7.1805944	6.8544245
6.0461674	7.751947	6.5465245	6.870746
4.644471	3.5963895	4.5057125	3.9329805
4.138937	3.1255329	3.2329185	3.5258389
4.2433863	3.2912536	3.6561031	3.2161844
2.350709	5.7582207	2.3618152	4.4081416
5.868784	4.4054723	5.930689	5.2298956
5.4863777	4.6850863	5.829446	5.225091
4.786666	4.2621455	4.9485474	4.1754317
6.8683367	8.749801	7.9668875	8.426911
3.7477627	2.3408759	2.3618152	3.3631585
3.7651324	3.3054376	3.6030655	3.105431
3.303122	2.9155302	3.8195217	2.8363883
4.6426964	4.5999384	4.8945465	4.530895
5.77373	5.066552	6.358503	5.6980553
4.896122	2.3408759	3.3963394	2.352752
4.3209305	4.2729287	4.854783	4.758981
4.5079484	3.3295732	4.3466597	3.5683482
4.5079484	3.3295732	4.3466597	3.5683482
7.220653	8.467757	7.451296	8.067653
7.220653	8.467757	7.451296	8.067653
9.3244505	8.356528	8.96983	8.7632675
4.755535	4.097975	4.484213	3.7541666
4.755535	4.097975	4.484213	3.7541666
4.755535	4.097975	4.484213	3.7541666
3.622365	2.9170492	2.3618152	2.352752
2.8987508	2.8076916	3.0597517	3.128833
7.360831	6.669672	6.9915404	6.8079033
6.7205853	7.150624	6.6840997	6.8741083
6.7205853	7.150624	6.6840997	6.8741083
6.7205853	7.150624	6.6840997	6.8741083
2.350709	2.3408759	2.3618152	2.352752
10.4938545	9.963567	11.194374	10.939101

ASHGA5P019373	0.002738397	0.033929075	2.3428346	up	noncoding
ASHGA5P019373	0.002738397	0.033929075	2.3428346	up	noncoding
ASHGA5P018940	0.019803701	0.085931879	2.0340309	up	noncoding
ASHGA5P014614	0.002420678	0.032008799	3.6441374	up	noncoding
ASHGA5P015663	0.041012225	0.129441514	3.4355488	up	noncoding
ASHGA5P058458	0.008577715	0.057726152	3.5564035	up	noncoding
ASHGA5P032685	0.029294936	0.10706631	2.1214872	up	noncoding
ASHGA5P026851	0.020468167	0.08778085	2.6445963	up	noncoding
ASHGA5P046404	0.007977332	0.05622546	2.2981537	up	noncoding
ASHGA5P017787	0.033522754	0.115469453	3.2284453	up	noncoding
ASHGA5P023257	1.42878E-06	0.001921139	3.0236657	up	noncoding
ASHGA5P044810	0.000921575	0.020476941	4.4989534	up	noncoding
ASHGA5P021330	0.029048836	0.106504196	2.1351048	up	noncoding
ASHGA5P051211	0.00062236	0.016906436	3.7948895	up	noncoding
ASHGA5P012461	1.06339E-05	0.004010044	4.4208286	up	noncoding
ASHGA5P012461	1.06339E-05	0.004010044	4.4208286	up	noncoding
ASHGA5P012461	1.06339E-05	0.004010044	4.4208286	up	noncoding
ASHGA5P012461	1.06339E-05	0.004010044	4.4208286	up	noncoding
ASHGA5P012461	1.06339E-05	0.004010044	4.4208286	up	noncoding
ASHGA5P033100	0.002853873	0.034516291	4.0149355	up	noncoding
ASHGA5P022833	0.017277622	0.080040435	2.1335774	up	noncoding
ASHGA5P056071	0.002733859	0.033921403	2.2160055	up	noncoding
ASHGA5P033340	0.035027063	0.118437866	2.2019658	up	noncoding
ASHGA5P033340	0.035027063	0.118437866	2.2019658	up	noncoding
ASHGA5P033151	0.001708576	0.027664864	2.3790617	up	noncoding
ASHGA5P031531	0.000386926	0.014011107	2.8542567	up	noncoding
ASHGA5P052947	0.013639077	0.071680418	2.5427593	up	noncoding
ASHGA5P031299	0.000296051	0.012593001	3.6369143	up	noncoding
ASHGA5P031299	0.000296051	0.012593001	3.6369143	up	noncoding
ASHGA5P000575	0.002595719	0.032877969	3.0687617	up	noncoding
ASHGA5P058494	0.029493368	0.107451211	2.0349105	up	noncoding
ASHGA5P035977	0.004748664	0.043793775	2.1161031	up	noncoding
ASHGA5P053967	0.00537183	0.046607024	5.0191242	up	noncoding

NR_024485	LOC100130093	RefSeq	2400	chr1	+
NR_024485	LOC100130093	RefSeq	2400	chr1	+
ENST00000475441	SNHG12	GENCODE	444	chr1	-
ENST00000411824	RP11-93B14.5	GENCODE	809	chr20	-
ENST00000422345	RP11-108M9.1	GENCODE	735	chr1	-
uc003tda.1	DPY19L1P2	UCSC_knowngene	1166	chr7	+
ENST00000572730	RP11-334C17.5	GENCODE	750	chr17	-
AW450413		LincRNAs identified	431	chr7	-
TCONS_00001386	XLOC_000698	LincRNAs identified	430	chr1	-
ENST00000445739	AC011322.1	GENCODE	546	chr3	-
ENST00000576197	RP11-1197K16.2	GENCODE	401	chr17	+
ENST00000518942	RP11-110G21.1	GENCODE	816	chr8	+
NR_024596	C11orf73	RefSeq	1129	chr11	+
ENST00000414022	TMEM191A	GENCODE	466	chr22	+
NR_034181	SUV39H2	RefSeq	3002	chr10	+
NR_034181	SUV39H2	RefSeq	3002	chr10	+
NR_034181	SUV39H2	RefSeq	3002	chr10	+
NR_034181	SUV39H2	RefSeq	3002	chr10	+
NR_034181	SUV39H2	RefSeq	3002	chr10	+
ENST00000429227	RP5-1061H20.4	GENCODE	868	chr1	-
NR_040000	LOC339166	RefSeq	1851	chr17	+
TCONS_00027655	XLOC_013195	LincRNAs identified	928	chr19	-
ENST00000583916	RP11-171G2.1	GENCODE	624	chr17	+
ENST00000583916	RP11-171G2.1	GENCODE	624	chr17	+
NR_026680	MGC57346	RefSeq	2110	chr17	+
ENST00000535363	RP11-27M24.1	GENCODE	533	chr16	+
NR_033960	LOC442497	RefSeq	2247	chr7	+
ENST00000456293	GAS5	GENCODE	583	chr1	-
ENST00000456293	GAS5	GENCODE	583	chr1	-
CX166999		LincRNAs identified	638	chr1	+
uc004azr.1	DQ673971	UCSC_knowngene	818	chr9	-
ENST00000577528	RP4-594I10.3	GENCODE	1445	chr1	-
uc004dgk.1	AK098783	UCSC_knowngene	1961	chrX	-

227916239	227922055		TCAGGCCCAGGT(	100130093	natural antisense
227916239	227922055		TCAGGCCCAGGT(	100130093	natural antisense
28905049	28907741	ENST00000461832,	ACTTAAGCAGAT(	85028	natural antisense
61294370	61298076		GCACCACCAGTGTCGCTGACTTGAA		natural antisense
17177815	17179624	ENST00000457465	CACAGGGCAGTGTCTTCTTGGTACT.		intergenic
32865913	32878356		AGTGAACTATTACATTCTAGAAAAG		intron sense-overlapp
78177631	78179748		GAGAGTGTTGTTAGCTCGGATTTAT'		natural antisense
25895917	25896427		TAATCATGAAGACGAGGGACGAACT		intergenic
9509252	9514916		TTCAACAAGATTGTCTGGTGCAAAG		intergenic
196710760	196713813		CACTGAAGGGTGTGTTTTAGAACCC		intergenic
80938957	80939614		AGGAAATGAGGCTGGCTGAAAAAT(		intronic antisense
52811884	52859165		TTGCATTGAAGGACACGGTCTTACG		bidirectional
86014397	86056985		CATAATCCTGGT(	51501	exon sense-overlappi
21057260	21058542		GGCTGTTCGGCG(	84222	intergenic
14920781	14946304	NM_024670	GCGCGAGGAGGA	79723	exon sense-overlappi
14920781	14946304	NM_024670	GCGCGAGGAGGA	79723	exon sense-overlappi
14920781	14946304	NM_024670	GCGCGAGGAGGA	79723	exon sense-overlappi
14920781	14946304	NM_024670	GCGCGAGGAGGA	79723	exon sense-overlappi
14920781	14946304	NM_024670	GCGCGAGGAGGA	79723	exon sense-overlappi
229394027	229406750		TCTGTGATTCGAGTATCAGGAAATC		bidirectional
5675553	5834016		AATGACCAAGGT(	339166	exon sense-overlappi
1246145	1248181		GACCAGATGCCCAGAGCCAGTCTAA		intergenic
70835177	70835857		CATTCAGAAGATACCTTTGGTCTCT(		intronic antisense
70835177	70835857		CATTCAGAAGATACCTTTGGTCTCT(		intronic antisense
43697711	43715329	NR_027295	GGTGCAAAGAGG	401884	exon sense-overlappi
10623296	10625939		ATGTATTGCTTGAGGGGGTCGATCT		natural antisense
419390	422845		GAACAGGACTGA	442497	intergenic
173834386	173837129	ENST00000421068,	CCTCCTAGTGCTC	60674	bidirectional
173834386	173837129	ENST00000421068,	CCTCCTAGTGCTC	60674	bidirectional
38893048	38911529		ACTGTGGAAGGGTTTGAATCACCTT		intergenic
102121666	102132150	uc004azm.1, uc004a:	TTTCAGCTTCCTGGATTCATATCTGT		intergenic
24865291	24882515		GATTTTTCTTATTCACTGTTTCCCAT		bidirectional
45707326	45710447		TGCATATTAAGATCCTTTTTCAACA/		intergenic

NM_001161465	JMJD4	jmjC domain-contain	-	227918889	227923112
NM_023007	JMJD4	jmjC domain-contain	-	227918889	227923112
NM_017846	TRNAU1AP	tRNA selenocysteine	+	28879528	28905057
NM_016354	SLCO4A1	solute carrier organic	+	61273796	61303647
ENST00000404479	AVL9	AVL9 homolog (S. c	+	32535321	33078516
NM_024110	CARD14	caspase recruitment c	+	78152306	78183130
NM_001009905	B3GNTL1	UDP-GlcNAc:betaGa	-	80901666	81009686
NM_052937	PCMTD1	protein-L-isoaspartat	-	52730139	52811735
NM_016401	C11orf73	protein Hikeshi	+	86013252	86056985
NM_001193424	SUV39H2	histone-lysine N-met	+	14920781	14946304
NM_001193425	SUV39H2	histone-lysine N-met	+	14920864	14946304
NM_001193426	SUV39H2	histone-lysine N-met	+	14920781	14946304
NM_001193427	SUV39H2	histone-lysine N-met	+	14920864	14946304
NM_024670	SUV39H2	histone-lysine N-met	+	14920898	14946304
NM_004578	RAB4A	ras-related protein Ra	+	229406878	229440518
ENST00000573619	WSCD1	WSC domain contair	+	5676245	5984112
NM_001159770	SLC39A11	zinc transporter ZIP1	-	70642084	71088853
NM_139177	SLC39A11	zinc transporter ZIP1	-	70642084	71088853
NM_001256299	CRHR1	corticotropin-releasir	+	43697709	43913194
NM_001424	EMP2	epithelial membrane	-	10622278	10674539
NM_001122770	ZBTB37	zinc finger and BTB	+	173837492	173855774
NM_032522	ZBTB37	zinc finger and BTB	+	173837492	173842778
NM_001010980	NCMAP	uncharacterized prote	+	24882566	24935818

40.500956	11.943798	4.965411	3.737156	52.853024	56.941704
40.500956	11.943798	4.965411	3.737156	52.853024	56.941704
4525.755317	1372.823263	11.69951	10.675169	4685.418	4225.252
228.68474	33.68042	7.258025	5.392447	215.07085	199.1935
5239.6446	1074.59441	11.675376	9.894835	4839.7534	6018.0938
686.750932	125.462064	8.954229	7.12381	718.98773	354.50372
32.440137	12.33765	4.682919	3.597843	31.861757	29.47595
227.27035	46.022683	7.227503	5.824456	224.642	272.81384
61.326045	21.152975	5.56518	4.364705	83.68519	56.033085
56.621784	13.585416	5.241243	3.550404	64.81521	109.29993
55.642785	11.969827	5.412245	3.815946	72.308655	64.64433
56.824107	9.93667	5.392416	3.222827	77.34342	66.6551
2882.602967	761.723647	11.012283	9.917976	2415.849	2560.362
1469.77025	245.45113	10.08499	8.160932	2222.9377	1508.6127
219.107397	30.328249	7.371805	5.227489	204.71576	242.61075
219.107397	30.328249	7.371805	5.227489	204.71576	242.61075
219.107397	30.328249	7.371805	5.227489	204.71576	242.61075
219.107397	30.328249	7.371805	5.227489	204.71576	242.61075
219.107397	30.328249	7.371805	5.227489	204.71576	242.61075
175.186672	28.265112	7.030258	5.024881	168.753	141.20831
796.009218	249.189818	9.203284	8.110009	976.88245	883.1299
342.42225	88.973117	7.947711	6.79975	444.37747	592.18915
23.505641	6.993801	4.076864	2.938072	31.406464	20.28476
23.505641	6.993801	4.076864	2.938072	31.406464	20.28476
181.093453	47.441144	7.072927	5.822534	128.14688	110.58029
1414.816417	287.999573	10.045144	8.532029	1449.646	1391.5143
73.905846	34.189844	5.456441	4.110046	104.80111	32.80204
375.579753	63.780864	8.126867	6.264152	361.8695	288.62698
375.579753	63.780864	8.126867	6.264152	361.8695	288.62698
76.34809	18.100856	5.872657	4.255	106.422104	88.34934
16.791217	6.409846	3.660263	2.635298	19.749952	13.683208
80.306446	24.619154	5.90578	4.82437	82.78576	40.72563
231.598767	33.075758	7.418235	5.0908	166.90111	129.2711

36.556732	30.987158	32.63396	33.03316	13.775548	8.998206
36.556732	30.987158	32.63396	33.03316	13.775548	8.998206
4276.9663	5785.933	3131.987	5048.9756	1089.9519	546.33545
193.00731	442.65533	54.62179	267.55966	38.44713	30.429731
2441.1633	1419.2574	8057.1357	8662.464	1557.1019	335.60474
889.2016	421.06644	858.8308	877.9153	73.69416	91.78642
28.328756	43.380272	22.480162	39.113926	16.87987	4.9999995
141.16748	436.48572	54.10884	234.40422	45.564476	28.86494
68.8391	40.920113	51.57502	66.90376	27.815317	7.442774
33.933575	17.580803	53.52517	60.576015	23.756086	7.2761927
41.703423	74.31433	32.993706	47.892265	16.720263	10.537569
79.8143	28.079943	47.04305	42.008827	12.982498	4.9999995
2167.9807	1936.3533	3941.2068	4273.866	808.34735	1087.0299
1302.8403	1130.1927	1199.4506	1454.5875	218.28319	99.9612
127.96569	210.77133	221.98329	306.59756	27.304884	26.528
127.96569	210.77133	221.98329	306.59756	27.304884	26.528
127.96569	210.77133	221.98329	306.59756	27.304884	26.528
127.96569	210.77133	221.98329	306.59756	27.304884	26.528
127.96569	210.77133	221.98329	306.59756	27.304884	26.528
162.36284	273.56094	95.20284	210.0321	17.29351	16.79644
646.63306	461.9818	869.22626	938.20184	354.1168	142.47131
228.94998	282.57693	201.70215	304.73782	106.27458	65.65163
12.28259	18.01087	13.44482	45.604343	7.65543	10.166669
12.28259	18.01087	13.44482	45.604343	7.65543	10.166669
158.70377	277.84424	164.50287	246.78267	27.320656	33.59238
1462.4191	940.196	1581.1323	1663.9908	246.71678	241.50421
183.95993	17.52742	56.85093	47.493645	49.42177	4.9999995
377.5106	534.2651	228.76686	462.43948	74.20928	35.921265
377.5106	534.2651	228.76686	462.43948	74.20928	35.921265
58.851444	58.088947	62.85982	83.516884	30.303598	9.512895
17.764189	26.776838	5.0840125	17.6891	13.45908	4.9999995
107.57542	110.36242	46.368107	94.02134	35.435215	11.642201
191.1726	248.86342	308.20877	345.1756	51.06151	19.163164

22.859386	11.53359	7.189952	7.3061037	5.255651	5.676667
22.859386	11.53359	7.189952	7.3061037	5.255651	5.676667
1724.245	939.46783	2408.0684	1528.871	11.719634	11.611698
29.902328	42.67526	28.882719	31.74535	7.298137	7.389969
3179.5447	357.9537	629.97986	387.38156	11.753469	12.095535
55.946312	105.99121	277.0589	148.29538	9.020507	8.183102
20.04135	18.106232	5.4726562	8.525795	4.535043	4.818328
35.911198	55.363327	55.70854	54.723618	7.360831	7.824805
52.66212	14.09968	13.274098	11.623861	5.934437	5.656571
34.07824	5.6153636	4.9999995	5.786614	5.55934	6.5527864
9.134722	18.057482	7.180349	10.18858	5.7174973	5.8430877
16.801533	4.9999995	14.835991	4.9999995	5.819184	5.884157
442.21634	896.29443	663.1015	673.35236	10.756419	10.895147
401.8254	182.86548	368.10522	201.66629	10.643144	10.180511
29.53738	30.850822	41.26858	26.47983	7.2250257	7.6567955
29.53738	30.850822	41.26858	26.47983	7.2250257	7.6567955
29.53738	30.850822	41.26858	26.47983	7.2250257	7.6567955
29.53738	30.850822	41.26858	26.47983	7.2250257	7.6567955
29.53738	30.850822	41.26858	26.47983	7.2250257	7.6567955
20.49962	26.86792	50.44372	37.68946	6.943407	6.91127
534.9008	169.44739	141.11533	153.08728	9.463007	9.449045
106.847084	75.30096	96.51035	83.2541	8.323768	8.89725
9.140706	4.9999995	4.9999995	4.9999995	4.515896	4.3150325
9.140706	4.9999995	4.9999995	4.9999995	4.515896	4.3150325
32.32705	63.26309	68.31715	59.82654	6.551623	6.568746
263.4591	298.68555	383.17953	294.45227	10.0338955	10.072842
126.15086	4.9999995	11.29016	8.276276	6.2662735	4.958408
95.08665	53.814205	69.747345	53.906437	8.037419	7.902903
95.08665	53.814205	69.747345	53.906437	8.037419	7.902903
33.17379	10.899619	8.639275	16.07596	6.287452	6.261116
4.9999995	4.9999995	4.9999995	4.9999995	3.844818	3.8105896
35.185776	20.791143	24.703844	19.956747	5.91938	5.2409825
70.4647	26.570868	16.143408	15.050899	6.924693	6.783414

4.876491	4.5086327	4.8840795	4.590945	4.010683	3.9438212
4.876491	4.5086327	4.8840795	4.590945	4.010683	3.9438212
11.710215	12.158989	11.321924	11.674601	10.389388	9.9533205
7.274791	8.480724	5.5888686	7.5156593	5.4991455	5.7658873
10.912378	10.150345	12.6610155	12.479512	10.909376	9.251125
9.430572	8.409232	9.490204	9.191759	6.431789	7.369813
4.515004	5.028482	4.3837647	4.8168936	4.3111634	2.4279919
6.818125	8.460256	5.5743656	7.326638	5.740194	5.6903105
5.794947	4.9389505	5.510723	5.5554514	5.0341306	3.6569169
4.7693167	3.592714	5.5577474	5.4155555	4.8120947	3.6244895
5.0626903	5.8556795	4.900607	5.0939074	4.295729	4.1842256
6.0035667	4.3466597	5.388265	4.912665	3.9233885	2.4279919
10.73358	10.591292	11.638906	11.458352	9.958928	10.964233
9.987953	9.820278	9.962795	9.91526	8.018048	7.49937
6.6782455	7.4070687	7.547723	7.7159743	5.0081215	5.56461
6.6782455	7.4070687	7.547723	7.7159743	5.0081215	5.56461
6.6782455	7.4070687	7.547723	7.7159743	5.0081215	5.56461
6.6782455	7.4070687	7.547723	7.7159743	5.0081215	5.56461
6.6782455	7.4070687	7.547723	7.7159743	5.0081215	5.56461
7.0105543	7.7754326	6.3622093	7.1786737	4.3499465	4.8860598
8.972414	8.536714	9.508995	9.289527	8.728452	8.004814
7.5159082	7.8234415	7.4187264	7.707172	6.9689827	6.8927608
3.290321	3.631518	3.6823473	5.026067	3.131765	4.1291914
3.290321	3.631518	3.6823473	5.026067	3.131765	4.1291914
6.976121	7.80188	7.135289	7.4039035	5.0094213	5.9085116
10.142613	9.557951	10.348855	10.11471	8.204572	8.767781
7.2014375	3.5869246	5.6418333	5.0837708	5.8599296	2.4279919
8.21139	8.74	7.593223	8.276265	6.439118	6.012279
8.21139	8.74	7.593223	8.276265	6.439118	6.012279
5.563604	5.4782276	5.781289	5.8642516	5.155034	4.0269637
3.830756	4.2785006	2.4739645	3.7229505	3.977643	2.4279919
6.431594	6.440709	5.3660274	6.035989	5.3808203	4.3314667
7.2576933	7.6520634	8.020663	7.870885	5.9098897	5.084915

4.546173	3.6406584	3.1468709	3.134729
4.546173	3.6406584	3.1468709	3.134729
10.692127	10.365895	11.487516	11.162765
4.9195175	5.6548414	5.1307707	5.3845224
11.603841	8.9389	9.529076	9.136693
5.7958097	7.076746	8.364308	7.7043962
4.368747	4.323081	2.789473	3.3666031
5.1700954	6.0718365	6.0653067	6.2089925
5.7095904	3.945613	4.0054483	3.8365288
5.1029253	2.6075516	2.3618152	2.7935462
3.309942	4.319272	3.1445363	3.6419725
4.128713	2.3408759	4.1632395	2.352752
8.748426	10.28197	9.600977	9.9533205
8.606754	7.9015408	8.762093	8.177788
4.9027133	5.1563654	5.6408777	5.092244
4.9027133	5.1563654	5.6408777	5.092244
4.9027133	5.1563654	5.6408777	5.092244
4.9027133	5.1563654	5.6408777	5.092244
4.9027133	5.1563654	5.6408777	5.092244
4.3985877	4.944685	5.926359	5.643648
9.015858	7.776702	7.377167	7.7570624
6.703638	6.5554075	6.8498535	6.827855
3.31203	2.3408759	2.3618152	2.352752
3.31203	2.3408759	2.3618152	2.352752
5.0268035	6.2824507	6.3613963	6.346623
7.988207	8.673475	8.821267	8.736874
6.939135	2.3408759	3.7711673	3.3211787
6.531945	6.027666	6.388048	6.1858544
6.531945	6.027666	6.388048	6.1858544
5.0630255	3.5547388	3.402621	4.3276176
2.350709	2.3408759	2.3618152	2.352752
5.1439953	4.5420656	4.8974514	4.650423
6.1098304	4.92615	4.2860208	4.227992
